# 12th European Headache Federation Congress jointly with 32nd National Congress of the Italian Society for the Study of Headaches: Florence, Italy. 28-30 September 2018

**DOI:** 10.1186/s10194-018-0900-0

**Published:** 2018-09-20

## EHF Invited Speakers

### S1 KATP channels

#### Mohammad Al-Mahdi Al-Karagholi (mahdi.alkaragholi@gmail.com)

##### Danish Headache Center, Department of Neurology, Faculty of Health and Medical Sciences, University of Copenhagen, Denmark

This abstract was not included as it has been previously published [1].

**Reference**

[1] Al-Karagholi MAM, Hansen JM, Severinsen J, Jansen-Olesen I, Ashina M. The KATP channel in migraine pathophysiology: a novel therapeutic target for migraine. J Headache Pain. 2017; 18(1):90. doi: 10.1186/s10194-017-0800-8.

### S2 The secondary headaches: a *cul de sac* for the headache expert ?

#### Christian Lampl (Christian.Lampl@ordensklinikum.at)

##### Headache Medical Center, Seilerstätte, Ordensklinikum Linz Barmherzige Schwestern, Austria

**Background**

According to the new ICHD 3 diagnostic criteria a de novo headache occurring with another disorder recognized to be capable of causing it is always diagnosed as secondary. For example, when a new headache occurs for the first time in close temporal relation to trauma or injury to the head and/or neck, it is coded as a “*secondary headache attributed to the trauma or injury*”. However, it may be possible, that this headache phenomenologically appears to be a primary headache. But, when a pre-existing headache with the characteristics of a primary headache disorder becomes chronic or is made significantly worse in close temporal relation to such trauma or injury, both the initial (primary) headache diagnosis and a diagnosis of “*Headache attributed to trauma or injury to the head and/or neck (or one of its types or subtypes)*” should be given. In other words, since headache is very prevalent, it can occur simultaneously with another disorder with and without a causal relation. Primary or secondary headache or both – clinically/scientifically spoken a dead end? These entities are a challenging diagnostic problem as can be primary or secondary and the etiologies for secondary cases differ depending on the headache type. Secondary headache can be definitely diagnosed only when solid evidence exists from published scientific studies that the disorder is capable of causing headache. Scientific evidence can come from large clinical studies observing close temporal relationships between the disorder and headache outcomes after treatment of the disorder, or from smaller studies using advanced scanning methods, blood tests or other paraclinical tests.

**Conclusion**

As all secondary headache disorders can be associated with a wide range of underlying etiologies such as infection, anatomical abnormalities, trauma, and immunological disease or sleep disorders, it is possible that these underlying pathophysiological processes generate long-standing activation of nociceptive mechanisms involved in headache. These can lead to chronification and refractoriness of the headache symptomatology.

**References**

1. Headache Classification Committee of the International Headache Society (IHS) (2018) The international classification of headache disorders, 3rd edition. Cephalalgia 38:1–211

### S3 Hormonal contraceptives: how they impact on migraine course

#### Simona Sacco (simona.sacco@univaq.it)

##### Department of Applied Clinical Sciences and Biotechnology, Section of Neurology, University of L’Aquila, L’Aquila, Italy

The role of female hormones in the pathogenesis of migraine is well-recognized [1,2]. Migraine is more prevalent in women than in men, it usually starts after puberty and in many women improves during pregnancy and after the menopause [1,3,4]. The menstrual phase of the female cycle represents a trigger for migraine attacks in many women [1,4]. Additionally, exogenous hormones may change the course of migraine by inducing de novo migraine, inducing de novo aura, worsening previous migraine but also improving migraine particularly those attacks related to menstruation [5,6]. Several attempts were made to manipulate the female hormonal cycle to try to improve migraine. A working group including headache experts, gynaecologists, stroke experts, and epidemiologists developed a first consensus document about the safety of hormonal contraceptives in female migraineurs of reproductive age [7]. Currently, no formal guidelines specifically address hormonal treatment of migraine. A further consensus document was developed by representatives of the European Headache Federation and the European Society of Hormonal Contraceptives and Reproductive Health. The aim of this consensus document is to provide recommendations on the management of migraine with the use of estrogens and progestogens in women of reproductive age. We systematically reviewed data about the effect of exogenous estrogens and progestogens on the course of migraine during reproductive age. Thereafter a consensus procedure among international experts was undertaken to develop statements to support clinical decision making, in terms of possible effects on migraine course of exogenous estrogens and progestogens and on possible treatment of headache associated with the use or with the withdrawal of hormones. Overall, quality of current evidence is low. Recommendations are developed for all the compounds with available evidence including the conventional 21/7 combined hormonal contraception, the desogestrel only oral pill, combined oral contraceptives with shortened pill-free interval, combined oral contraceptives with estradiol supplementation during the pill-free interval, extended regimen of combined hormonal contraceptive with pill or patch, combined hormonal contraceptive vaginal ring, transdermal estradiol supplementation with gel, transdermal estradiol supplementation with patch, subcutaneous estrogen implant with cyclical oral progestogen. As the quality of available data is poor, further research is needed on this topic to improve the knowledge about the use of estrogens and progestogens in women with migraine. There is a need for better management of headaches related to the use of hormones or their withdrawal.

**References**

1. MacGregor EA (2014) Oestrogen and attacks of migraine with and without aura. Lancet Neurol 3:354-361.

2. Vetvik KG, MacGregor EA (2017) Sex differences in the epidemiology, clinical features, and pathophysiology of migraine. Lancet Neurol 16:76-87.

3. Ripa P, Ornello R, Degan D, Tiseo C, Stewart J, Pistoia F, Carolei A, Sacco S (2015) Migraine in menopausal women: a systematic review. Int J Womens Health 7:773-782.

4. Sacco S, Ricci S, Deagn D, Carolei A (2012) Migraine in women: the role of hormones and their impact on vascular diseases. J Headache Pain 13:177-189.

5. Loder EW, Buse DW, Golub JR (2005) Headache and combination estrogen-progestin oral contraceptives: integrating evidence, guidelines, and clinical practice. Headache 45:224-231.

6. Warhurst S, Rofe CJ, Brew BJ, Bateson D, McGeechan K, Merki-Feld GS, Garrick R, Tomlinson SE (2018) Effectiveness of the progestin-only pill for migraine treatment in women: a systematic review and meta-analysis. Cephalalgia 38:754-764.

8. Sacco S, Merki-Feld GS, Ægidius KL, Bitzer J, Canonico M, Kurth T, Lampl C, Lidegaard Ø, MacGregor EA, MaassenVanDenBrink A, Mitsikostas DD, Nappi RE, Ntaios G, Sandset PM, Martelletti P; European Headache Federation (EHF) and the European Society of Contraception and Reproductive Health (ESC) (2017) HCs and risk of ischemic stroke in women with migraine: a consensus statement from the European Headache Federation (EHF) and the European Society of Contraception and Reproductive Health (ESC). J Headache Pain 18:108

### S4 EHF Guidelines on the use of CGRP(r) MAbs in migraine

#### Simona Sacco (simona.sacco@univaq.it)

##### Department of Applied Clinical Sciences and Biotechnology, Section of Neurology, University of L’Aquila, L’Aquila, Italy

Great enthusiasm is preceding the marketing of new treatments for migraine prevention which act on the calcitonin gene-related peptide (CGRP) pathway. This offsets the lack of significant novelties in treatment of migraine for many years since in the 80s triptans were marketed. Factors that contribute to the increasing enthusiasm are that those new treatments are, as triptans, migraine specific whereas all the other available preventive drugs were developed for indications other than migraine and have an unclear mechanism of action considering migraine pathophysiology. Poor tolerability and side effects which may particularly troublesome for patients are important limitations of available preventive treatments [1]. Efficacy is not always as expected by patients. Those factors are the main responsible of medication discontinuation and poor adherence which is very often observed with available treatments [2-4]. From a physician perspective, comorbidities limit the possibility of using those drugs in some patients.

We have now, four monoclonal antibodies (mAb) available acting on the CGRP pathway: one targeting the CGRP receptor (erenumab) and three targeting the CGRP peptide (galcanezumab, eptinezumab, and fremanezumab).

The European Headache Federation (EHF) set up a panel of experts to develop evidence-based guideline to clinicians for the management of episodic and chronic migraine with mAb acting on the CGRP or on its receptor. The guidelines included recommendations about safety and efficacy for each of the available drug and suggestions on how to use them in daily clinical practice. Guidelines were developed using the GRADE approach where feasible. Additionally, experts’ suggestions were provided for clinical questions which were considered relevant for the use of mAb acting on the CGRP. Consensus among experts was reached by using the Delphi method.

The EHF on the use of mAb acting on the CGRP will hopefully represent a useful tool for the use of those drugs in the clinical practice. The guidelines will be systematically updated as far as new evidence about those drugs will become available.

**References**

1. Blumenfeld AM, Bloudek LM, Becker WJ, Buse DC, Varon SF, Maglinte GA, Wilcox TK, Kawata AK, Lipton RB (2013) Patterns of use and reasons for discontinuation of prophylactic medications for episodic migraine and chronic migraine: results from the second international burden of migraine study (IBMS-II). Headache 53:644-655.

2. Berger A, Bloudek LM, Varon SF, Oster G (2012) Adherence with migraine prophylaxis in clinical practice. Pain Pract 12:541-549.

3. Diamond S, Bigal ME, Silberstein S, Loder E, Reed M, Lipton RB (2007) Patterns of diagnosis and acute and preventive treatment for migraine in the United States: results from the American Migraine Prevalence and Prevention study. Headache 47:355-363.

4. Loder EW, Rizzoli P (2011) Tolerance and loss of beneficial effect during migraine prophylaxis: clinical considerations. Headache 51:1336-1345.

### S5 Novel Insight into Post Traumatic Headache: Lessons learned from Blast Exposed Civilians

#### Gal Ifergane (gal.ifergane@gmail.com)

##### Department of Neurology, Division of Brain Medicine, Soroka University Medical Center, Ben-Gurion University of the Negev, Beer-Sheva, Israel

Although extremely common, Post Traumatic Headache (PTH) deserves little attention in Headache medicine and in research. The strict criteria for PTH, defining its onset less than 7 days from a head trauma and the lack of specific headache phenotype limit the ability to study clinical samples. The limited ability to evaluate traumatic exposure, , and the association of PTH with multi symptom syndromes e.g. Post Concussion Syndrome (PCS) or Post Traumatic Stress Disorder (PTSD), further complicate the study PTH.

We had the opportunity to study the Neuro Psychiatric sequelae in civilians residing next to missile impact sites. This population allows us to define the level of exposure to explosion as direct (participants residing in direct line of sight from the explosion), and indirect (residing in 500m radius of explosion but without direct line to the explosion).

Brain MRI scans of 42 participants with blast exposure (direct or indirect) exhibited significant white-mater diffusion alterations, as compared with 16 non-exposed participants. Notwithstanding, individuals with direct exposure demonstrated differential white matter diffusion characteristics as compared to individuals with indirect exposure.

Clinical evaluation using semi-structured interviews of 289 exposed participants (direct exposure 58, 20%) demonstrated that neither PTSD, nor PCS were associated with the degree of blast exposure. Interestingly, they were associated with the emotional experience of fear and helplessness felt in the explosion.

Active headache was reported by 109 participants (38%) who expressed higher PCS and PTSD symptomatology and poorer sleep quality compared to participants without active headache. Headache phenotype (migraine or tension) and severity were similarly not associated with the degree of blast exposure.

Our data suggests that while MRI diffusion abnormalities are exposure related, headache, as well as PCS and PTSD are not necessarily so, and that the emotional experience plays an important role in its development.

### S6 Imaging inflammation in animal models of migraine

#### Aaron Schain, Agustin Melo-Carrillo, Andrew Strassman, Rami Burstein

##### Department of Anesthesia, Critical Care and Pain Medicine, Beth Israel Deaconess Medical Center, Harvard Medical School, Boston, MA

###### **Correspondence:** Aaron Schain (aschain@bidmc.harvard.edu)

Inflammation is likely to have a key role in the pathophysiology of migraine. NSAIDs and anti-inflammatory agents have success in its treatment, and inflammatory molecules such as nitric oxide, CGRP, and cytokines are able to recreate migraine pain. Our lab has shown that cortical spreading depression (CSD), the neural correlate of migraine aura, can activate rodent dural nociceptors. Because CSD is an event that takes place within the brain, yet dural nociceptors lie outside, there must be some chain of events linking the two environments, which likely involves inflammation.

To better understand how inflammation might be involved in CSD’s activation of dural nociceptors, we developed an *in vivo* imaging strategy that allows us to follow inflammatory processes that begin in the brain and end in the dura. We used a state-of-the-art 2-photon microscope to create three-dimensional images through the lightly thinned skulls of mice that allow us to see all the layers of the meninges and the superficial cortex at cellular resolution. We used genetically modified mice to study the shape and behavior of macrophages and dendritic cells, immune cells that are very sensitive to changes in inflammatory environment, in each meningeal layer before and after propagation of CSD.

We found that both cell types had predictable characteristics that were modified by CSD. Macrophages at baseline had large dynamic phagocytic cytoplasmic extensions that were pulled into the cell body post CSD. This happened first in macrophages within the brain, and 10-20 minutes later with the macrophages outside the brain, on the dura. A subset of dendritic cells was found to be highly migratory at baseline, but stopped immediately post CSD. These cells were able to migrate freely within the subarachnoid space near the pia and the dura. Notably both macrophages and dendritic cells exhibiting these behaviors were found adjacent to or in contact with TRPV1-positive fibers in the dura.

Our findings raise the possibility that activation of immune cells in the pia and dura after CSD may be involved in the activation of meningeal nociceptors, potentially by altering the environment in which nociceptors reside – a condition shown previously as sufficient for activation of nociceptors. Furthermore, our study underscores the ability of in vivo imaging to connect inflammatory processes within the brain to outside the brain.

### S7 What have we learned from cellular and circuit mechanisms of the primary brain dysfunctions that cause familial hemiplegic migraine?

#### Daniela Pietrobon^1,2^ (daniela.pietrobon@unipd.it)

##### ^1^Department of Biomedical Sciences and Padova Neuroscience Center University of Padova, 35121 Padova, Italy; ^2^CNR Institute of Neuroscience, 35121 Padova, Italy

Migraine is much more than an episodic headache disorder. It is a complex brain disorder, characterized by a global dysfunction in multisensory information processing. The headache is associated with one or more symptoms indicating amplification of sensory gain and, in the interictal period, migraineurs show several alterations in sensory information processing. In a third of migraineurs, the headache is preceded by transient sensory disturbances, the so called migraine aura, whose neurophysiological correlate is cortical spreading depression (CSD). In animal studies CSD can activate the trigeminovascular pain network and hence the headache mechanisms. The molecular, cellular and network mechanisms of the primary brain dysfunctions that underlie migraine onset, susceptibility to CSD and altered interictal sensory processing remain largely unknown and are major open issues in the neurobiology of migraine. To tackle these questions we studied the functional consequences of mutations causing familial hemiplegic migraine (FHM), a rare monogenic subtype of migraine with aura, in knock-in mouse models carrying either a FHM type 1 (FHM1) or a FHM type 2 (FHM2) mutation. FHM1 is caused by gain-of-function mutations in the neuronal Ca_V_2.1 channel, a voltage-gated calcium channel that plays a dominant role in controlling neurotransmitter release at brain excitatory and inhibitory synapses. FHM2 is caused by loss-of-function mutations in the glial α2 Na/K-ATPase. Both FHM1 and FHM2 mouse models have a lower threshold for induction of experimental CSD and a higher velocity of CSD propagation. We investigated the mechanisms underlying the facilitation of experimental CSD in FHM1 and FHM2 knock-in mice by studying synaptic transmission at different cortical excitatory and inhibitory synapses and the rates of glutamate and K+ clearance during neuronal activity in slices of somatosensory cortex. We will summarize some of our findings supporting the conclusions that i) excessive cortical excitatory synaptic transmission and excessive activation of NMDA receptors, due either to enhanced glutamate release in FHM1 or impaired glutamate clearance in FHM2, are the main mechanisms underlying facilitation of experimental CSD in the genetic migraine models; ii) the dynamic regulation of the excitatory-inhibitory balance during thalamocortical activity is dysfunctional in FHM1 mice. The data from our genetic migraine models are consistent with the hypothesis that a reduced ability to dynamically maintain an appropriate excitatory/inhibitory balance in specific brain networks may underlie the dysfunctional sensory information processing in migraine and, in certain conditions, may favor CSD ignition and the onset of a migraine attack.

### S8 What is the evidence for role of inflammation in migraine? Why do NSAIDs work so effectively in acute treatment

#### M. Ashina (ashina@dadlnet.dk)

##### Danish Headache Center, Department of Neurology, Rigshospitalet Glostrup, University of Copenhagen, Denmark

In 1968, the first study linking prostaglandins and migraine pathogenesis was conducted and reported that an intravenous injection of prostaglandin E1 induced a migraine-like headache. The mechanism of action of NSAIDs is primarily inhibition of prostaglandin formation and inflammation. Aspirin and other NSAIDs are widely used drugs for acute treatment of migraine and comparative randomized clinical trials demonstrated efficacy similar to triptans. Preclinical migraine models have suggested that migraine pain is associated with local inflammation in the meninges involving mast cells and subsequent activation of trigeminal nociceptive neurons. However, definitive evidence for inflammation in dura or perivascular space in migraine patients is still lacking.

### S9 Emerging targets for treatment – apart from CGRP – PAC1 receptor and PACAP

#### Luise Haulund Vollesen

##### Danish Headache Centre and Department of Neurology, Rigshospitalet Glostrup, Faculty of Health and Medical Sciences, University of Copenhagen, Denmark

Pituitary adenylate cyclase-activating peptide-38 (PACAP38), a vasoactive signaling molecule involved in parasympathetic and sensory signaling, belongs to the glucagon/secretin superfamily of peptides and is found in several key structures of interest in migraine pathophysiology such as the trigeminovascular system. PACAP38 provokes migraine attacks in migraine patients without aura. Interestingly, the structurally related vasoactive intestinal peptide (VIP) does not induce migraine. Both PACAP38 and VIP have affinity for at least three different receptors: the VPAC1, VPAC2, and PAC1 receptors. However, PACAP38 shows higher affinity on the PAC1 receptor than VIP. Studies suggested three potential targets of interest in migraine treatment; the PAC1 receptors, PACAP38 itself, or a de novo receptor. An ongoing phase II trial will reveal whether targeting the PAC1 receptor shows clinical efficacy in migraine treatment.

### S10 Counting cost: disability and lost productivity

#### T. J. Steiner (t.steiner@imperial.ac.uk)

##### Norwegian University of Science and Technology, Trondheim, Norway

Headache disorders are now recognized as the second cause of disability worldwide, after low back pain. Among specific diseases, migraine is top cause in people under 50. Disability, especially during the productive years up to age 50, translates into lost productivity and thence into financial cost, which is very high wherever it has been measured.

Empirical evidence for these statements comes from various sources. Population-based studies estimate headache *prevalence*. The Global Burden of Disease (GBD) studies multiply population prevalence, mean proportion of time in the ictal state (essentially a product of attack frequency and duration) and a disability weight (DW) reflecting the loss of health attributed, through a very large global consultation, to the ictal state. The result, expressed in years lived with disability (YLDs), is reported as *disability* but, as a measure of lost health, might more accurately be considered as *impairment*. A minority of population-based studies have simultaneously reported lost productive time, using measures such as the MIDAS (Migraine Disability Assessment) instrument. These, too, have reported their results as *disability*, although they better reflect *behavioural response to impairment* (there is, unless headache is very bad, an element of choice in working or not working with headache). Lost productive time is not a measure of disability, although it might be expected to correlate strongly with disability. However, it is not clear that an independent measure of headache-attributed disability exists.

Whether arising from impairment or disability, and whatever external factors influence it, lost productive time is an important consequence of headache, assumed to reduce output, with financial penalty. It would be helpful to understand the relationships between these.

This presentation takes examples from population-based studies conducted by *Lifting The Burden*, using individual-participant data obtained contemporaneously to describe symptom severity (impairment), YLDs and lost productive time. Focusing on migraine, it explores the relationships between these variables, and shows that past assumptions may not have been correct. There are implications for cost-effectiveness analyses that value interventions according to their expected impact on indirect financial costs.

### S11 The migraine brain is inheritably hyper-responsive

#### Magis Delphine (dmagis@chuliege.be)

##### Headache Research Unit, University Department of Neurology CHR, Liège, Belgium

Increased responsivity to environmental stimuli is associated with migraine attacks but is also reported interictally. The way the migraine brain responds to sensory stimuli has been intriguing researchers for a long time. Electrophysiology provides an evaluation of neural responses to different stimuli and thus constitutes one of the most direct methods of sensory processing analysis. Neurophysiological studies demonstrated three functional features of migraine patients compared to healthy controls: habituation modifications, cortical dysexcitability and abnormal functional connexions and circuits within the CNS (chiefly thalamo-cortical dysrhythmia). Whether these (controversial) peculiarities are genetically determined or only result from a favourable environment can be a matter of debate. Both genetic and environmental factors may actually influence the electrophysiological behaviour of each subject and therefore partially account for the differences observed between subgroups. This presentation will review the arguments in favour of an inherited hyperresponsivity of the migraine brain.

### S12 Seizure activates the trigeminovascular system differently than CSD: Implications to differences between post-ictal headache and migraine

#### Agustin Melo Carrillo (amelo1@bidmc.harvard.edu)

##### Department of Anesthesia, Critical Care and Pain Medicine. Beth Israel Deaconess Medical Center. Harvard Medical School, Boston, MA, USA

**Summary**

Seizures affect 50 million people worldwide and are followed by headache in up to 50% of patients. These headaches are called Post-Ictal Headaches (PIH), they are commonly migraineous in nature, can last many hours, be very painful and since they strikes repetitively, they drive patients to endure a significant hardship. Given that seizures occur in the cortex, we hypothesized that PIH are intracranial at origin and accordingly, aimed in this study to determine whether and how seizures (both generalized and focal) activate peripheral and central neurons of the meningeal sensory pathway.

**Aim of investigation**

To investigate the mechanism of post-ictal headache.

**Methods**

Single-unit electrophysiological techniques were used to study the physiological properties and response profile of C- and Aδ meningeal nociceptors and high-threshold (HT) and wide-dynamic range (WDR) neurons in the spinal trigeminal nucleus – in response to occurrence of seizure underneath their dural receptive fields. Cortical electrodes were used to trace the magnitude, extent and progression of epileptiform seizure.

**Results**

The induction of seizure triggered prolonged activation in both meningeal nociceptors and central trigeminovascular neurons. In the ganglion, activity began to increase minutes after the seizure reached their receptive fields and remained elevated for as long the seizure activity continued. In the medullary dorsal horn, neurons exhibited a biphasic response following seizure onset that consisted of an initial inhibition of the spontaneous activity followed by a gradual increase in activity that remained above baseline for at least 2 hours after seizure onset. In separate experiments, we created a focal seizure that was restricted to either the parietal or occipital cortex. Induction of focal seizure in the parietal cortex did not produce any changes in neuronal activity, but induction of focal seizure in the occipital cortex produced the same neuronal changes that we observed with the generalized seizure.

**Conclusions**

This study provides evidence for activation of the trigeminovascular pathway by generalized and focal seizures. The importance of this study is that it uses a model of abnormal cortical activity to which there are ample of evidence in human subjects suffering epilepsy (and PIH), and thus, it bypass the bitter debate over the validity of studying the headache phase of migraine using the controversial cortical spreading depression model – to which evidence for actual occurrence in humans are scarce.

**Acknowledgments**

NIH Grant: RO1-NS094198.

### S13 Cortical Spreading Depression as a trigger for migraine headache

#### Rami Burstein

##### Department of Anesthesia and Critical Care, Beth Israel Deaconess Medical Center

Cortical spreading depression (CSD) has long been implicated in migraine attacks that begin with visual aura. Over the years, it was hotly debated whether aura can trigger a headache. This lecture will focus on data that show that a single wave of CSD can trigger long-lasting activation and sensitization of peripheral and central trigeminovascular neurons in 2 manners. In the first, neurons become activated 10-40 minutes after occurrence of CSD and in the second they become activated immediately. These findings will be discussed in the context of the headache and the possible role activation of peripheral trigeminovascular neurons play in migraine chronification.

The process by which CSD, a cortical event that occurs within the blood brain barrier (BBB), results in nociceptor activation outside the BBB is complex and likely mediated by multiple molecules and cells. This lecture will also present up-to-date data on the possible mechanisms and processes that occur between the cortical aura and the nociceptor activation.

### S14 Migraine onset in childhood is a genetic disease

#### Andrew Hershey (Andrew.Hershey@cchmc.org)

##### Cincinnati Children’s Hospital Medical Center, 3333 Burnet Avenue, MLC 2015, Cincinnati, OH 45229,USA

Migraine has long been noted to “run in families.” Studies have suggested a strong familial pattern that is influenced by environmental contributions. In this talk, I will review the population genetic studies suggesting migraine as a genetic condition, including family and twin studies. These observations have been further advanced by genetic tools including genome wide association studies (GWAS), individual gene identification studies – especially for hemiplegic migraine, polymorphism identification, and more recently by whole exome sequencing. This can be combined with genomic expression to identify pathways that contribute to the genetic lode and the phenotypic development of migraine. All these techniques and their stages in leading to the understanding of migraine as a genetic disease will be discussed.

### S15 Providing care: Cost-effective and affordable

#### Michela Tinelli (m.tinelli@lse.ac.uk)

##### Personal Social Services Research Unit, London School of Economics (LSE), Houghton Street, London WC2A 2AE, UK

**Introduction and objectives**: Headache disorders are real illnesses, often causing lifelong disabilities. Migraine, tension-type headache (TTH) and medication-overuse headache (MOH) are of major public-health importance: collectively, they are the 2nd highest cause of disability in populations throughout the world, leading to much lost productivity and very high indirect costs (>€100 billion per year in EU). The Value of Treatment (VoT) Project is a timely and ground-breaking initiative of the European Brain Council (EBC) in collaboration with the LSE, *Lifting The Burden* (LTB), European Headache Federation and other partner institutions. It argues that optimizing interventions in brain disorders can bring not only positive outcomes for patients but also economic gains for society. As part of the VoT project, the headache economic case study estimated cost/effectiveness of implementing structured headache services (SHSs) based in primary care and supported by educational initiatives aimed at both patients and health-care providers.

**Methods**: We modelled cost-effectiveness of SHSs delivering treatments for each of the headache types (migraine, TTH, MOH), with efficacy known from randomized controlled trials. Three health-care systems – of Russia, Spain and Luxemburg – brought different experiences of health service delivery and financing into the model. We made annual (short-term) and 5-year cost estimates from health-care provider and societal perspectives (2017 figures, euros). We expressed effectiveness as healthy life years (HLYs) gained, and cost-effectiveness as incremental cost-effectiveness ratios (ICERs) (cost to be invested/HLY gained).

**Results**: In short-term modelling from the health-care provider perspective, the intervention is cost effective overall and across headache types – well below WHO framework thresholds. Over 5 years, the intervention is even more cost effective. Results are consistent across health-care systems. From the societal perspective, the intervention is not only cost-effective but also cost-saving over 1 year and 5 years, for all types of headache and across health-care systems. The greater the country’s wage levels, the greater are the economic savings for society (Luxemburg > Spain > Russia).

**Discussion**: For the first time, the effectiveness and cost effectiveness of introducing hypothetical SHSs in Europe was evaluated across health-care systems. Our results study showed that such services, based in primary care and supported by patient and provider education, are effective and cost-effective solutions to headache disorders and the disability they cause. From the health-care provider perspective, cost-effectiveness is least (ICERs greatest) for TTH because of its much lower disability weight compared with those for migraine and MOH. In practice, structured headache services will not discriminate: they must manage all headache types; however, people with TTH are least likely to require them.

**Acknowledgments**

This study was part of the EBC-led Value of Treatment Project. The headache economic study team included Michela Tinelli *^1^, Timothy Steiner*^2^, Matilde Leonardi^3^, Dimos Mitsikostas^4^, Koen Paemeleire^4^, Elena Ruiz de la Torre^5,6^. ^1^The London School of Economics and Political Science; ^2^*Lifting The Burden*; ^3^European Brain Council (EBC) WHO Liaison; ^4^European Headache Federation; ^5^European Federation of Neurological Associations (EFNA); ^6^European Headache Alliance (EHA). *These authors contributed equally.

### S16 Expression of CGRP family of neuropeptides in the trigeminal system

#### Lars Edvinsson^1,2^, Karin Warfvinge^1,2^

##### ^1^Departments of Internal Medicine, Lund University Hospital, Lund, Sweden; ^2^Department of Clinical Experimental Research, Copenhagen University Hospital, Glostrup, Denmark.

###### **Correspondence:** Lars Edvinsson (lars.edvinsson@med.lu.se)

**Background.** The calcitonin gene-related peptide (CGRP) family of neuropeptides consists besides CGRP of adrenomedullin, amylin and calcitonin (CT). They share receptor components and structure, and have several similar biological actions. The receptors consist of either calcitonin receptor like-receptor (CLR) or calcitonin receptor (CTR) which for function needs an accessory protein, receptor activity-modifying proteins (RAMPs). These define the receptor subtype and can interact and produce different effects via signal transduction and receptor trafficking. It is by now well known that CGRP has a pivotal role in primary headaches but the role of the other members of the CGRP family of peptides and their receptors is not known. Here, we describe the expression of these molecules in the trigeminal ganglion (TG) to understand the role they may have in headaches.

**Methods.** Single or double immunohistochemistry were applied on frozen sections of rat trigeminal ganglia using primary antibodies against CGRP, adrenomedullin, amylin, CT, RAMP1/2/3, CLR and CTR.

**Results**. CGRP and CT showed similar results with expression in TG small to medium-sized neurons. Immunoreactive fibers were also found. Adrenomedullin immunoreactivity was found in the satellite glial cells (SGC) and on the fibers, probably staining of the myelinating cells. Amylin was found in the cytoplasm in many TG neurons and in some SGC.

When it comes to the receptor components, many large neurons showed immunoreactivity to both CLR and RAMP1, and also to fibers, most likely neuronal processes. In addition, SGCs were immunoreactive to the two receptor components. RAMP2 and RAMP3 were expressed in all nuclei. CTR was found in almost all cells, both neurons and SGCs.

**Conclusion**. Several of the diverse biological actions of the CGRP family of peptides are clinically relevant. Our findings demonstrate the specific ligand and receptor sites in the rat TG, highlighting recognition mechanisms to facilitate drug development.

**Key words**. CGRP peptides family, ligands, receptors

### S17 Managing secondary headaches with a multimodal expertise

#### Henrik Winther Schytz (henrikschytz@hotmail.com)

##### Danish Headache Center, Department of Neurology, Rigshospitalet Glostrup, Denmark

Secondary headaches are often a huge challenge to clinicians at all levels and there is a general lack of evidence-based managment of secondary headaches. At the Danish Headache Center 30 % of the patients treated suffer from secondary headaches with medication overuse headache being the most frequent. The lecture managing secondary headaches with a multimodal expertise will present the approach to managing secondary headaches such as medication-overuse headache, post-traumatic headache, headache attributed to spontaneous intracranial hypotension and headache attributed to idiopathic intracranial hypertension. Perspectives on how to approach the patient in tertiary headache center will be presented as well.

### S18 Hormonal contraceptives in women with migraine: pros and cons

#### E. Anne MacGregor (anne@annemacgregor.com)

##### Barts Health NHS Trust, London UK

Since migraine is most prevalent during women of reproductive age, many women with migraine will be using contraception. Further, since some migraine medication is contraindicated in pregnancy, for women with migraine using such medication, it is important that contraception is highly effective and does not adversely affect migraine. Indeed, irrespective of the need for contraception, several contraceptive strategies may also benefit migraine, particularly for women who experience exacerbation of migraine perimenstrually. Menstruation is associated with two independent migraine triggers – estrogen ‘withdrawal’ which typically occurs premenstrually, and prostaglandin release, often associated with menorrhagia and/or dsymenorrhoea. Understanding how the different methods of hormonal contraception affect either one, or both, of these potential mechanisms is useful for management.

Hormonal methods are the most popular methods of contraception and include combined hormonal contraceptives (combined oral contraceptives [COC], contraceptive vaginal ring [CVR], and combined transdermal patch [CTP]) and progestogen-only methods (progestogen-only pill [POP], subdermal implant [SDI], intramuscular/subcutaneous depot medroxyprogesterone acetate [DMPA], and levonorgestrel intrauterine system [LNG-IUS]), all of which, with the exception of DMPA, are immediately reversible.

Combined hormonal contraceptives contain estrogen, usually synthetic ethinylestradiol, and a progestogen. The addition of ethinylestradiol provides greater ovarian suppression and better cycle control than progestogen-only methods, although the incidence of breakthrough bleeding increases as the dose of ethinylestradiol is reduced. The older first- and second-generation progestogens, derived from testosterone, tend to exhibit androgenic actions, counterbalancing the estrogenic activity. Newer third- and fourth-generation progestins have been designed to have neutral androgenic or estrogenic actions. Traditional regimens include a 7-day contraceptive-free interval (CFI) following 21-days of hormones in order to mimic a monthly menstrual bleed. Hormone ‘withdrawal’ can also occur *during* hormone treatment as recruited follicles undergo atresia, resulting in a drop in endogenous estradiol levels. Continuous or extended regimens with a shorted CFI have the potential to reduce the frequency and severity of estrogen-withdrawal symptoms.

The desogestrel POP and implant inhibit ovulation but do not predictably inhibit ovarian activity. This can result in fluctuating estrogen levels and unscheduled bleeding. DMPA inhibits ovarian activity and bleeding, although it can take 6-9 months before bleeding is fully suppressed. The LNG-IUS allows the normal menstrual hormone cycle fluctuations but maintains a thin endometrium, reducing menstrual blood loss and prostaglandin release.

The efficacy, risk and non-contraceptive benefits of each of these methods will be discussed.

### S19 Cortical spreading depression as mechanism of the migraine aura: new mechanisms

#### Martin Lauritzen (martin.johannes.lauritzen@regionh.dk)

##### Department of Clinical Neurophysiology, Rigshospitalet and Institute for Neuroscience, University of Copenhagen, Glostrup, DK-2600, Denmark

Cortical spreading depression and depolarization waves (CSD) are associated with dramatic failure of brain ion homeostasis, efflux of excitatory amino acids from nerve cells, increased energy metabolism and changes in cerebral blood flow (CBF). There is strong clinical and experimental evidence to suggest that CSD is involved in the mechanism of migraine, stroke, subarachnoid hemorrhage and traumatic brain injury. The implications of these findings are wide-spread and suggest that intrinsic brain mechanisms have the potential to worsen the outcome of cerebrovascular episodes or brain trauma. The consequences of these intrinsic mechanisms are intimately linked to the composition of the brain extracellular microenvironment and to the level of brain perfusion and in consequence brain energy supply. This presentation summarizes the evidence provided by novel experiments in rodents using twophoton microscopy imaging which reveal new mechanisms of CSD of importance for our perception of CSD as a pathophysiological mechanism of migraine and for a group of acute neurological disorders. Specifically, the talk will summarize new mechanisms that implicates involvement of organelles in neurons and the cortical microcirculation at the level of brain capillaries in CSD. The findings are likely to have implications for how we understand migraine with aura and for new strategies for migraine treatment.

### S20 Pain regulation by non-neuronal cells and inflammation

#### Ru-Rong Ji (ru-rong.ji@duke.edu)

##### Center for Translational Pain Medicine, Departments of Anesthesiology and Neurobiology, Duke University Medical Center, Durham, North Carolina, 27710, USA

Acute pain is protective and a cardinal feature of inflammation. Chronic pain after tissue and nerve injury is detrimental and associated with chronic inflammation. Accumulating evidence suggests that non-neuronal cells such as immune cells and glial cells play active roles in the pathogenesis of pain via neuron-immune and neuro-glial interactions. Neuroinflammation, as characterized by infiltration of immune cells, activation of glial cells, and production of inflammatory mediators in the peripheral and central nervous system, plays an important role in the induction and maintenance of chronic pain. I will discuss distinct roles of inflammation, neurogenic inflammation, and neuroinflammation in the induction and maintenance of pain including headache. I will also discuss how macrophages and specialized pro-resolving mediators promote the resolution of inflammation and pain.

### S21 Occipital Migraine and the Cerebellum

#### Rodrigo Noseda, Rami Burstein

##### Department of Anesthesia, Critical Care & Pain Medicine, Beth Israel Deaconess Medical Center, Harvard Medical School, Boston MA, USA

###### **Correspondence:** Rodrigo Noseda (rnoseda@bidmc.harvard.edu)

The neural substrate of occipital headache in migraine and other headaches is little understood. In theory, this type of headache could emerge extracranially from tension, stiffness or tenderness in neck and shoulder muscles or intracranially from activation of meningeal nociceptors in the dura overlying the cerebellum, of the posterior fossa. That the cerebellum is affected in migraineurs is convincingly demonstrated by multiple imaging studies showing altered activation and connectivity of the cerebellum in the migraine brain. Yet, little is known about the role it may play in the initiation of headache. Anecdotally, patients whose migraine headaches involve mainly or exclusively the occipital region appear to experience vertigo – a symptom attributed mainly to the cerebellum – more than patients whose migraine are restricted to the periorbital and frontal regions of the head. Given the above scenario, we sought to identify the anatomical origin and trajectory of the sensory innervation of the cerebellar dura, characterize the functional properties of neurons processing nociceptive input from this area, and determine their susceptibility to activation and sensitization by inflammatory agents.

In this presentation, we will revisit current understanding of the causes of occipital headache, discuss potential pathological scenarios for its occurrence, and provide anatomical data obtained in the rat that shows in unprecedented detail the nerve trajectories and terminal sensory innervation of the intracranial dura overlying the cerebellum by C2-3 upper cervical DRGs. We will also provide electrophysiological data from central neurons processing nociceptive input from the posterior dura under experimental pathological conditions. These novel anatomical and functional data will be discussed in the context of occipital migraine.

### S22 The migraine brain is not hyper-responsive

#### Trond Sand^1,2^ (trond.sand@ntnu.no)

##### ^1^Department of Neuromedicine and Movement Science, Faculty of Medicine and Health Sciences, NTNU - Norwegian University of Science and Technology, Trondheim, Norway; ^2^Department of Neurology and Clinical Neurophysiology, St. Olavs hospital HF, Trondheim University Hospital, 7006, Trondheim, Norway

Obligatory and common symptoms like phonophobia, photophobia and allodynia clearly suggest neural hypersensitivity during a migraine attack. Reliable premonitory symptoms like photophobia are also reported in the preictal phase 24 hours before headache. However, the variety of migraine triggers is huge, and the most reliable triggers are unspecific like “sleep” and “stress”. Hence, even from a clinical viewpoint it is more uncertain if an interictal CNS-hyper-responsitivity exists.

The extremely well-known theory about “deficient interictal CNS-habituation in migraine” has regretably not been confirmed in fully blinded visual evoked potential (VEP) studies, neither in Norway nor in the Netherlands. It is of course very important to blind both recording and analysis to achieve a reliable confirmation of initial and preliminary evidence.

Another measure of CNS-hyperexcitability is transcranial magnetic stimulation (TMS). Again, results from TMS-studies in migraine are widely divergent, although this variability is often neglected in the literature. Also,TMS is not a natural sensory stimulus, and the method does not convey information about “responsitivity” defined as a sensory stimulus-response function.

Different modalities may have different excitability and responsitivity. It will be of great interest to test if thalamocortical somatosensory excitability changes, measured with somatosensory evoked potential (SEP) and pain evoked potential methods can be confirmed to be abnormal also with fully blinded methodology. It is also of great interest to use other EEG-based methods like event-related synchronization/desynchronization to confirm if frequency.specific rhythm responsivity is abnormal in interictal and preictal phases.

The simplistic idea about a general hyper- or hypo-responsitivity for the whole brain should be abandoned. Instead, it is likely that states of hypo-, normal, and hyper-excitability occur in succession and differs among various brain networks. Indeed, excessive variability within and between different networks may be hypothesized as the most probable abnormality in migraine. Another challenge is to identify new methods that are able to reliably measure stimulus-response functions in different CNS network subsystem in humans.

### S23 MRI studies can predict progression of disease years before it occurs

#### Roberta Messina, Maria A. Rocca, Massimo Filippi

##### Neuroimaging Research Unit, Institute of Experimental Neurology, Division of Neuroscience, San Raffaele Scientific Institute, Vita-Salute San Raffaele University, Milan, Italy

###### **Correspondence:** Roberta Messina (messina.roberta@hsr.it)

Over the last two decades, a series of neuroimaging studies has changed the way we understand the pathophysiology of migraine. Conventional and advanced magnetic resonance imaging (MRI) techniques have been applied extensively to the study of migraine patients, both in the course of an acute attack and during the interictal phase. It is now accepted that migraine should be viewed as a complex brain network disorder that involves multiple cortical, subcortical and brainstem regions, rather than a purely vascular disorder.

Numerous studies have consistently demonstrated that patients with migraine experience not only abnormalities of function but also important and distributed structural abnormalities of the brain gray matter (GM) and white matter (WM). In particular, a selective involvement of key brain regions and networks involved in multisensory processing, including pain, has been shown to occur in the brain of migraineurs. Whether such alterations represent a predisposing trait or are the consequence of the recurrence of headache attacks is still a matter of debate. Many studies have indicated that structural brain abnormalities in migraineurs might be dynamic, since they differ according to the migraine phase, attack frequency and disease duration. Recent longitudinal studies revealed that the migraine brain changes over time and different pathophysiological mechanisms are associated to disease severity. Other investigations have shown morphometric alterations that are not influenced by migraine patients’ clinical characteristics and revealed that GM volume abnormalities are detectable from the early stages of the disease, thus representing a potential migraine biomarker.

Numerous conventional MRI studies have also described an increased risk of harbouring brain WM hyperintensities in patients with migraine, that might be influenced by headache frequency, disease duration and the presence of aura. All of this is critical not only to improve the understanding of migraine pathophysiology, but also to develop imaging biomarkers to be applied in treatment trials of new experimental drugs.

### S24 Three dimensional approch for DDI evaluation in Precision Medicine

#### Leda Marina Pomes^1^, Giovanna Gentile^2^, Maurizio Simmaco^2^, Marina Borro^2^, Paolo Martelletti^3,4,5,6^

##### ^1^Residency Program in Laboratory Medicine, Gabriele d’Annunzio University, Chieti, Italy; ^2^Department of Neurosciences, Mental Health and Sensory Organs, Sapienza University, Rome Italy; ^2^Department of Clinical and Molecular Medicine, Sapienza University, Rome Italy; ^3^Residency Program in Internal Medicine, Sapienza University, Rome, Italy; ^4^Master in Headache Medicine, Sapienza University, Rome, Italy; ^5^Internal Medicine and Emergency Medicine Unit, Sant’Andrea Hospital, Rome, Italy; ^6^Regional Referral Headache Center, Sant’Andrea Hospital, Rome, Italy

###### **Correspondence:** Paolo Martelletti (paolo.martelletti@uniroma1.it)

The evidence of multiple comorbidities in migraine population, especially at cardio-cerebrovascular, psychiatric, metabolic and musculoskeletal levels, has led to an increase in the observation in clinical practice of patients undergoing multiple pharmacological therapies. The use of different drugs in the same migraine patient significantly increases the risk of potentially serious adverse events (AEs). As a consequence, the development and implementation in clinical practice of precision medicine addresses this increased clinical risk, allowing to evaluate in advance the possible drug/drug interactions (DDIs) and guiding the clinical prescription and monitoring. We have also to consider that in a real-world scenario each migraine patient might also suffer from other (episodic or chronic) not comorbid pathologies that require additional therapies. These adjunctive treatments may result in additional DDIs and AEs risking to produce a decrease in the clinical efficacy of prescribed drug(s).

The evidence of daily polypharmacy (≥5 different drugs) in headache patients have been recently reported in a large cross-sectional study and this misuse occurs in 40.7% of this cohort. The chronic headache patients’ subset of this cohort reached an awesome 58.8% for ≥5 drugs and 5% for ≥10 drugs administration at the same time.

Considering these complex drug-drug cross-interactions the application of personalized medicine can help the clinician to evaluate the individual profile of the subject and according to it to proceed to a specific therapeutic strategy not competing with the same metabolic pathway.

The more drugs are present in the polytherapy, the more possible interactions are established. Notably, these kind of DDIs are largely predictable using open source, knowledge-based reporting the ADME profile for each drug. A useful one is Transformer (http.//bioinformatic.charite.de/transformer), which provides information on DDIs based on drug transport and metabolism of over 3000 drugs and more than 350 foods and supplements. Transformer analyzes an input drug list comparing the interactions (substrate, induction or inhibition) existing between each drug and different metabolic enzymes and, through a color code, highlights potential unfavorable DDIs; furthermore, through a drop-down menu, it allows to compare the metabolic route of equivalent drugs on the same screen, providing a tool to make an alternative prescription choice. Another web – based database that provides updated information about drug specific ADMEs proteins (enzymes and transporters) and the pharmacological interactions that can occur in polytherapies is DrugBank (http://www.drugbank.ca/). It returns information about the metabolism and mechanism of action of 10523 molecules with pharmacological action.

As pharmacogenomics sciences revealed that genes encoding many important DMEs are characterized by the presence of functional polymorphisms affecting the enzyme activity level and thus the expected PKs profile of drugs interacting with such enzymes, this genomic information started to be used to adjust the prediction of drug safety and tolerability made using a simple DDIs checker.

### S25 The EMA-EHF Documento for migraine

#### Efstratia Vatzaki^1*^, Sabine Strauss^2^, Jean-Michel Dogne^3^, Juan Garcia Burgos^1^, Thomas Girard^1^ and Paolo Martelletti^4^

##### ^1^European Medicine Agency, London, UK; ^2^Medicines Evaluation Board, The Netherlands; ^3^Head of the Department of Pharmacy, Namur Thrombosis and Hemostasis Centre - Narilis University of Namur, Belgium; ^4^European Headache Federation, Rome, Italy

###### **Correspondence:** Efstratia Vatzaki (Efstratia.Vatzaki@ema.europa.eu)

Migraine is a common and burdensome neurological condition which affects mainly female patients during their childbearing years. Valproate has been widely used for the prophylaxis of migraine attacks and is also included in the main European Guidelines. Previous (2014) European recommendations on limiting the use of valproate in women of childbearing age did not achieve their objective in terms of limiting the use of valproate in women of child bearing age and raising awareness regarding the hazardous effect of valproate to children exposed *in utero*. The teratogenic and foetotoxic effects of valproate are well documented, and more recent studies show that there is an even greater neurodevelopmental risk to children exposed to valproate in the womb. The latest 2018 European review from the European Medicines Agency, with the active participation of the European Headache Federation, concluded that not enough has been done to mitigate the risks associated with *in utero* exposure to valproate. The review called for more extensive restrictions to the conditions for prescribing, better public awareness, and a more effective education campaign in migrainous women.

**Keywords**: migraine prophylaxis, valproate, pregnancy, teratogenic risk, foetotoxic effect, neurodevelopmental retard, pregnancy prevention programme, clinical recommendations, education, public awareness.

### S26 Precision medicine general concept in clinical pratice

#### Marina Borro, Giovanna Gentile, Maurizio Simmaco

##### Department of Neurosciences, Mental Health and Sensory Organs, Faculty of Medicine and Psychology, Sapienza University of Roma and Sant'Andrea Hospital of Rome, Laboratory of Clinical Biochemistry

###### **Correspondence:** Maurizio Simmaco (maurizio.simmaco@uniroma1.it)

Inefficacy/safety of pharmacological treatment remain main challenges for healthcare systems. The biological inter-individual variability in drug response is a multi-factorial phenomenon modified by both static factors, as the genomic background, and by dynamic factors, as ageing, life-style, environment. Furthermore, polypharmacy presents the peculiar risk of drug-drug interactions which could alter the drug pharmacokinetics and pharmacodynamics, causing adverse effects or low response.

The personal genomic profile is a “static” contributor of the expressed phenotype, which is determined by a plethora of dynamic factors. The advent of modern technologies, as Next Generation Sequencing and mass spectrometry, has allowed to deeply characterize genomic profiles as well as molecular signatures that are a clue to define more exactly the clinical phenotype of the patient.

We developed a strategy aimed to make actionable the clinical use of genomics and other molecular signatures. The foundation of this strategy is the high-level integration of molecular data with clinical and environmental data. Taking advantage from a multi-disciplinal environment, a new cultural model in medical practice has taken root at the Sant’Andrea Hospital of Rome, allowing step by step establishment of a patient-centered care model, embedded in the workflow of the Italian National Health System.

### S27 Correlation of migraine attacks with neck pain and tension

#### E Anne MacGregor^1^, Stephen Donoghue^2^, Marina Vives-Mestres^2^

##### ^1^Barts Health NHS Trust, London UK; ^2^Curelator Inc., Cambridge MA, USA

###### **Correspondence:** E Anne MacGregor (anne@annemacgregor.com)

**Background:** Neck pain associated with migraine attacks is common and usually first noticed close to time of headache onset [1,2]. However, because it can occur prior to headache, it can be perceived as a migraine trigger. We used a digital platform (Curelator Headache®) to determine 1) how many individuals *suspect* neck pain/tension as a migraine trigger and 2) for how many individuals an association between neck pain/tension and migraine attacks can be identified statistically.

**Methods:** Individuals with migraine registered to use Curelator Headache® (now called N1-Headache®) and answered questions about which factors they suspect contribute to attack occurrence, including neck pain/tension, and rate their importance (0=none; 10=maximal). They then used Curelator Headache® daily for 90 days, entering details of headaches and factors possibly associated with attack occurrence: presence of neck pain/tension was determined by a Yes/No response to the daily question ‘Did you notice neck pain or tension (today)?’. After 90 days all factors were analyzed and for each individual the association between neck pain/tension and migraine attacks was determined via a univariate Cox proportional hazard model [3] using a) all data and b) relating attack occurrence with *previous* day neck pain data (lagged analysis).

**Results:** Table 1 shows statistical associations between neck pain/tension and attack risk according to the degree of suspicion as a trigger in 774 individuals (non-lagged analysis).

Neck pain/tension was associated with increased risk of attacks (‘trigger’) in about one-fifth of individuals, but never found as a ‘protector’. There was an association between degree of neck pain/tension *suspicion* as a trigger and frequency of statistical confirmation of an association with attacks. Not analyzed individuals had insufficient data for analysis, due to constant or low variability in neck pain/tension (128: 46.9%), low number of attacks or convergence problems in the Cox model. In the lagged analysis, only 4.9% of individuals had an association between attacks and preceding day neck pain/tension.

**Conclusion:** Neck pain/tension was widely suspected as a migraine trigger. In one-third of individuals with adequate data there was a statistical association between same day neck pain/tension and migraine headache. However, neck pain/tension the day *before* headache started was much less commonly correlated with attacks: this supports the view that neck pain/tension is more likely a migraine symptom rather than a trigger.

**References**

1. Calhoun AH *et al.* Headache 2010;50:1273-1277

2. Lampl C *et al.* J Headache Pain 2015;16:80-84

3. Peris F *et al*. Cephalalgia 2017; 37(5): 452-463

**Table 1 (abstract S27).** See text for description

**Suspected? →**	Not suspected	Low (levels 1-3)	Medium (levels 4-6)	High (levels 7-10)	No answer	**TOTAL** ***n (%)***
**Association? ↓**						
Associated ‘trigger’	19	13	31	92	6	161 (20.8)
Associated ‘protector’	0	0	0	0	0	0 (0)
No association	56	40	81	147	16	340 (43.9)
Not analyzed	72	37	47	99	18	273 (35.3)
**TOTAL** ***n (%)***	147 (19)	90 (11.6)	159 (20.5)	338 (43.7)	40 (5.2)	774

### S28 Brainstem aura is not real

#### Anne Ducros (a-ducros@chu-montpellier.fr)

##### University of Montpellier, and Headache Centre, Neurology department, Montpellier University Hospital, France

According to ICHD-3, migraine with brainstem aura is defined as a migraine with aura including at least two of the following symptoms: dysarthria, vertigo, tinnitus, hypacusis, diplopia, ataxia and/or decreased level of consciousness.

Whereas the typical migraine aura symptoms are thought to be the clinical consequence of cortical spreading depression (CSD), the mechanisms of migraine with brainstem aura remain debated. One hypothesis could be that “brainstem” aura does not originate in the brainstem but in the cerebral cortex.

Even though any symptom of brainstem aura can be observed in patients with brainstem lesions, numerous clinical observations and functional mapping data show that these latter symptoms can also originate within the cortex. Vertigo can result from a vestibular cortex dysfunction while tinnitus and hypacusis can originate within the auditory cortex. Diplopia can reflect a parieto-occipital involvement. Dysarthria can be caused by dysfunctions located in precentral gyri. Ataxia can reflect abnormal processing of vestibular, sensory or visual inputs by the parietal lobe. Alteration of consciousness can be caused by abnormal neural activation within specific consciousness networks that include prefrontal and posterior parietal cortices.

Any symptom of so-called brainstem aura can originate within the cortex. Based on these data, we suggest that brainstem aura could have a cortical origin. A potential cortical origin of “brainstem” aura would explain the frequent association between symptoms of brainstem and of typical aura during attacks and the frequency of “brainstem” aura symptoms in hemiplegic migraine. Moreover, a cortical origin of any aura symptom (e.g typical, brainstem-like and hemiplegic) would fit with the theory of CSD as the underlying mechanism of migraine aura. In conclusion, patients with brainstem aura are real, but migraine with brainstem aura is not real and could be called migraine with brainstem-like aura.

### S29 Migraine with brainstem aura does exist

#### Jes Olesen (jes.olesen@regionh.dk)

##### Rigshospitalet and university of Copenhagen, Denmark

Migraine with brainstem aura has been a recognized entity for decades. It was first described by Bickerstaf as basilar artery migraine. He believed that it was caused by spasms of the basilar artery. At the time of the first international headache classification in 1988 it was clear that spasms do not explain any kind of migraine. The diagnosis was therefore renamed basilar migraine. In ICHD-2 it was called basilar-type migraine in order to get further away from the basilar artery and finally, in ICHD-3, it was renamed migraine with brainstem aura. Formally applying the diagnostic criteria of the ICHD-II to a large material of systematically phenotyped migraine with aura patients resulted in a surprisingly high number patients who fulfilled the criteria [1]. While formally migraine with brain stem aura thus exists and formally is quite common, we as experts do not believe that it is common. On the other hand we have identified a number of patients at the Danish Headache Center who undoubtedly have brainstem symptoms as an aura of migraine and we shall present some cases.

**References**

[1] Li, D., A.F., C., & J., O. (2015, August). Field-testing of the ICHD-3 beta/proposed ICD-11 diagnostic criteria for migraine with aura. *Cephalalgia*, pp. 748-756.

### S30 Manual Approach to Tension-Type Headache: Is this a Reliable Approach?

#### César Fernández-de-las-Peñas (cesar.fernandez@urjc.es)

##### Departamento de Fisioterapia, Terapia Ocupacional, Rehabilitación y Medicina Física. Universidad Rey Juan Carlos, Alcorcón, Madrid, Spain

The etiology of tension type headache (TTH) is not completely understood yet. Current evidence suggests a potential role of muscle referred pain, particularly trigger points (TrPs) in the development and maintenance of pain and sensitization mechanisms in TTH. In addition, other factors such as mood disorders or sleep disturbances are also involved in the chronification process. Understanding the integration of musculoskeletal disorders may help to develop better management regimes for patients with TTH. The current lecture will provide updated discussion of the clinical reasoning for application of physical manual therapy approaches in TTH since different therapeutic options are currently proposed for these patients. Current evidence supports the concept that not all manual therapies are equally effective for patients with TTH and that the effectiveness of these interventions will depend on a proper clinical reasoning. Since the pathogenesis of TTH is mainly associated to muscular impairments, manual therapies should be targeted to muscle trigger points and other related tissues. This could explain the inconsistent results in the current literature in relation to effectiveness of manual therapy for TTH. In addition, multimodal approaches including different interventions are more effective than isolated therapeutic approaches for TTH since they can focus on both peripheral and central mechanisms. All these topics will be discussed in this lecture.

### S31 Differences and similarities observed in functional imaging of headache studies

#### Anders Hougaard (ahougaard@dadlnet.dk)

##### Danish Headache Center & Dept. of Neurology, Righospitalet Glostrup, University of Copenhagen, Denmark

Neuroimaging studies have substantially enhanced our possibilities for investigating pathophysiological mechanisms of primary headache disorders and experimental head pain. In recent years, an immense number of imaging studies in headache have been published, often with diverging findings. New technologies and imaging data analysis methods are being developed at an increasing speed. Future studies have the potential not only to advance our understanding of headache-relevant disease mechanisms, but also to improve diagnostics, to elucidate the effects of treatment, and to monitor treatment effects in an objective and non-invasive way.

This presentation focuses on highlights of studies using positron emission tomography, functional MRI, MR angiography, and MR spectrometry and possible future directions of this field of research

### S32 Migraine in childhood is an environmental disease - focus on the role of epigenetic

#### G. Natalucci^1^, N. Faedda^2^, G. Turturo^1^, V. Guidetti^1^

##### ^1^Department of Human Neuroscience, “Sapienza” University of Rome, Rome, Italy; ^2^Behavioural Neuroscience, Department of Human Neuroscience, “Sapienza” University of Rome, Rome, Italy

###### **Correspondence:** V. Guidetti (vincenzo.guidetti@uniroma1.it)

In 1942 Conrad Waddington defined for the first time the term “epigenetics”. Synthetically, it refers to environmental factors and events that can affect gene expression or chromatin structure, for example trough post-translational changes of the tails of histone proteins, DNA methylation or RNA-associated silencing, resulting in changes in DNA structure without altering the genetic code. In this contest, several recent theories have supposed that in a disease like migraine, epigenetic mechanisms may explicate how non-genetic endogenous and exogenous factors and possibly influence frequency, intensity and genesis of migraine. In fact, while there are several evidences and studies on the role of specific genes on migraine’s development, the impact of biological, neural, molecular and environmental factors is still uncertain and difficult to scientifically assess without significant *bias* [1]. Some studies have demonstrated, for example, that hormonal changes, especially in women, sleep deprivation, skipping meals and stress can have a high influence on the different expression of migraine attacks. Specifically, it seems that female hormones, through epigenetic mechanism, can change the balance between inhibitory and excitatory neurotransmission, increasing excitatory neuronal activity. Other important factors that can have an epigenetic influence are early stressing experiences. It has been demonstrated a high prevalence of headache and migraine and substantial changes on neural circuits in adults who experienced a type of abuse in their life (sexual, emotional, physical or domestic abuse) [2]. In fact, many researchers have found how early negative adversity may have long-term effects on hypothalamic–pituitary–adrenal (HPA) axis function and immune system activity through epigenetic modifications, and these mechanisms may explain how attack frequency is modulated by non-genetic factors. Moreover, experiences like childhood maltreatment can lead to severe migraine manifestations which can be also more refractory to treatment. Early negative life experiences and female hormones are shared factors by both migraine and depression. Studies on animal models demonstrated how aberrant epigenetic gene regulation may be associated with the pathophysiology of mood disorders and consequently, they could be the same pathways of headache and migraine. So, causal pathways shared between migraine and its comorbid disorders may be moderated by epigenetic mechanisms [3]. Because of prophylactic medication used to prevent migraine is only effective in half of the patients, more studies on epigenetic migraine mechanism may provide a new conception of migraine pharmacotherapy, specifically acting to modulate chromatin structure at migraine features.

**References**

1) Hershey AD, Faedda N. Guidetti V. “Epigenetics” In: Guidetti V., Arruda M., Ozge A. (Eds) “Headache and Comorbidities in Childhood and Adolescence” Springer International Publishing 2017 page 31-37 ISBN 978-3-319-54726-8

2) Tietjen GE Childhood Maltreatment and Headache Disorders. Curr Pain Headache Rep. 2016 Apr;20(4):26. doi: 10.1007/s11916-016-0554-z.

3) Eising E, A Datson N, van den Maagdenberg AM, Ferrari MD. Epigenetic mechanisms in migraine: a promising avenue? BMC Med. 2013 Feb 4;11:26. doi: 10.1186/1741-7015-11-26. Review.

### S33 Sports and headache

#### Randolph W. Evans

##### Department of Neurology, Baylor College of Medicine, Houston, Texas

Sports are commonly associated with headaches. Concussions can lead to post-traumatic migraines, tension- type headaches, medication overuse, occipital neuralgia, and cervicogenic. Minor head injury can trigger migraine with aura (footballer's migraine). The usual acute and preventive treatments can be considered for the phenotype. Neurologists treating collegiate and professional athletes should be familiar with prohibited substances for treatment as listed by the World Anti-Doping Agency. Non-traumatic headaches associated with sports include the following: exertional, cough (weightlifter's), and external compression headaches.

### S34 Post-traumatic headache is a unique headache disorder

#### Mattias Linde (mattias.linde@ntnu.no)

##### Department of Neuromedicine and Movement Science, NTNU Norwegian University of Science and Technology, Trondheim, Norway

According to the new version of the International Classification of Headache Disorders (ICHD-3), persistent headache attributed to traumatic injury to the head (HAIH) is among the most common headache disorders. Epidemiological evidence to support this statement has, however, been lacking. Only two previous studies using non-injured comparison groups have investigated the causal relationship between head injury and headache, and they show conflicting results.

Since HAIH does not differ from migraine and tension-type headache (TTH) based on clinical characteristics, some experts are disputing its existence, arguing that it may be nothing other than a primary headache being misattributed to a head injury

The speaker will present the findings of the so far largest controlled, population-based study on HAIH with known pre-trauma headache status. More than one thousand traumas were classified according to the Head Trauma Severity Scale (HISS). Adjusting for potential confounders, it can now be concluded that HAIH is a true secondary headache entity. Prognostic markers at the time of admission, headache phenotypes, and dose-response relationships will be reviewed.

### S35 Fifty years of Norwegian contributions to headache research

#### L. J. Stovner (lars.stovner@ntnu.no)

##### Norwegian Advisoy Unit on Headaches, Department of Neuromedicine and Movement Science, Norwegian University of Science and Technology (NTNU), and St. Olavs Hospital, Trondheim, Norway

For a small country, Norway has made substantial contributions to the headache field. The founder of this tradition is without doubt professor Ottar Sjaastad, who from he started his work in the sixties, described several new headache entities (Chronic paroxysmal hemicranias, Hemicrania continua and SUNCT), was among the founding fathers of IHS and the Scandinavian Migraine Society, was the founding editor of Cephalagia which he edited for almost 10 years. In his older days performed the Vågå study, in which he personally interviewed almost all 1800 inhabitants in a Norwegian community. In the same period, professor Tor-Erik Widerøe made the first proper trials proving that a beta-blocker (propranolol) was affective as migraine preventative. In the same tradition, professor Harald Schrader made the observations leading to the development of lisinioril and later candesartan as migraine prophylactics. The large epidemiological survey in Nord-Trøndelag county (the HUNT study) from around 1995 became an arena for headache epidemiological studies, through the work of professors John-Anker Zwart, Knut Hagen, Gunnar Bovim and myself. This tradition in epidmiology, together with the establishment of The Norwegian National Headache centre from the start of the 2000, has made the headache group at the Norwegian University of Science and Technology in Trondheim the academic basis for the NGO Lifting the Burden, running the Global campaign against headache. This has also led to the work to demonstrate the huge societal impact of headache, as collaborators of the Global Burden of Disease project. The group is now performing innovative research for treating chronic cluster headache and migraine.

### S36 Correlation of migraine attacks with neck pain and tension

#### E. Anne MacGregor^1^, Stephen Donoghue^2^, Marina Vives-Mestres^2^

##### ^1^Barts Health NHS Trust, London UK, ^2^Curelator Inc., Cambridge MA, USA

###### **Correspondence:** E. Anne MacGregor

**Background:** Repetitive yawning is a common premonitory migraine symptom [1,2]. In contrast to other common premonitory symptoms such as neck pain and tiredness, repetitive yawning is more specific and has a high predictive value for a migraine attack [3]. We used a digital platform (Curelator Headache® - now called N1-Headache®) to determine 1) how many individuals recorded excessive yawning and 2) for how many individuals an association between excessive yawning and migraine attacks can be identified statistically.

**Methods:** Individuals with migraine registered to use Curelator Headache®. They then used this daily, entering details of headaches and factors possibly associated with attack occurrence: presence of yawning was determined by a Yes/No response to the daily question ‘Did you notice excessive yawning (today)?’. To be eligible for analysis, data must include 90 tracked days or more, at least five migraine attacks, more than 50 answers to the yawning question, and excessive yawning must be reported on at least 5% of days, but not all days. After 90 days all factors were analysed and for each individual the association between excessive yawning and migraine occurrence was determined via a univariate Cox proportional hazard model [4].

**Results:** Of 852 individuals with migraine, 285 (33.5%) were eligible for analysis. Excessive yawning was associated with increased risk of migraine attack in 72 (25.3%), with decreased risk in 4 (1.4%) and no significant within-person association was identified in 189 (66.3%). Risk could not be assessed in 20 (7%) due to convergence problems in the Cox model. Of the 72 with increased risk, the median hazard ratio was 3 (IQR=4-2.2) meaning that, for them, when yawning is present the occurrence of migraine is about three times the rate per unit time as when there is no yawning.

**Conclusion:** In some individuals excessive yawning is a discernible symptom that is a sensitive predictor of migraine. Early identification of migraine provides an opportunity for early intervention. Future studies can assess effective strategies during the premonitory stage to abort an attack.

**References**

1. Laurell K et al. Cephalalgia 2016; 36(10): 951-959

2. Guven B et al. Headache 2018; 58(2): 210-216

3. Giffin NJ et al. Neurology 2003; 60(6): 935-940

4. Peris F et al Cephalalgia 2017; 37(5): 452-463

## SISC Invited Speakers

### S37 Comorbidities of Migraine - Sleep Disorders

#### Catello Vollono (lvol@libero.it)

##### Department of Neurology, Catholic University, Fondazione Policlinico Universitario Agostino Gemelli IRCCS, Rome, Italy

**Background**

Migraine is a pleomorphic relapsing, remitting disorders characterized by recurrent attacks that can be triggered or precipitated by a large number of internal and external factors. The relationship between sleep and migraine has always been known, but current knowledge on the exact nature of the link between migraine and sleep remains incomplete and unclear.

In other primary headaches, such as Hypnic Headache and Cluster Headache, sleep studies has shown dysfunction of arousal mechanisms, associated with the brain's ability to process exogenous and endogenous stimuli during sleep.

Different sleep studies were performed in order to define also the complex link between sleep and migraine.

**Results**

Fragmentation of sleep, insomnia and hypersomnia all showed relationships with migraine. Primary sleep disorders such as insomnia, hypersomnia, sleep breathing disorders are all associated with primary headaches, may cause headache, can worsen migraine and are risk factors for migraine chronification. Medical, psychiatric and rheumatic diseases are associated with sleep disorders and migraine.

Migraine is associated with modifications in sleep: circadian changes in the sleep-wake rhythm [1], modifications of the ultradian rhythm (more frequent than 24h, and therefore shorter than a day, the alternation of NREM / REM phases in the sleep cycles) [2], changes in arousals mechanisms [3].

Sleep-related migraine is characterized by a marked reduction in the CAP rate in NREM sleep and a lower incidence of 'cortical' arousal in REM sleep (cortical hypoarousability) that could predispose to the appearance of migraine episodes during sleep [4].

In migraineurs the autonomic balance during sleep changes more evidently than in controls and the oscillations of the arousal levels change in a concordant manner [5].

**Conclusions**

Migraine has close clinical correlation with sleep and sleep disorders. Moreover, widespread pathophysiological links have been shown between migraine and sleep, expression of reduced effectiveness of the mechanisms of processing of the incoming stimuli.

The study of sleep/wake pattern in order to know and try to change the influence of biological rhythms, sleep and trigger factors on migraine can allow the clinicians to control the frequency and intensity of attacks and, above all, to avoid the chronicity of the disorder.

**References**

[1] Ahn AH, Goadsby PJ. Migraine and sleep: new connections. Cerebrum. 2013 Dec 1;2013:15

[2] Jennum P, Jensen R. Sleep and headache. Sleep Med Rev. 2002 Dec;6(6):471-9

[3] Bruni O, Russo PM, Violani C, Guidetti V. Sleep and migraine: an actigraphic study. Cephalalgia. 2004 Feb;24(2):134-9

[4] Della Marca G, Vollono C, Rubino M, Di Trapani G, Mariotti P, Tonali PA. Dysfunction of arousal systems in sleep-related migraine without aura. Cephalalgia. 2006 Jul;26(7):857-64

[5] Vollono C, Gnoni V, Testani E, Dittoni S, Losurdo A, Colicchio S, Di Blasi C, Mazza S, Farina B, Della Marca G. Heart rate variability in sleep-related migraine without aura. J Clin Sleep Med. 2013 Jul 15;9(7):707-14.

### S38 Nutraceuticals and headache

#### Lidia Savi (lsavi@cittadellasalute.to.it)

##### Headache Center LBS, Lugano, 6900, Switzerland

Headache disorders are in many cases inadequately managed with the existing treatments. Also, given the myriad side effects of traditional prescriptions medications, there is an increasing demand for “natural” treatment like vitamins, minerals, herbal preparations and food supplements.

The term “ nutraceutical “ was created by Defelice in 1979 and is derived from “nutrition” and “pharmaceutical” industries in the idea that some foods can be effective in treating diseases.

In the last years the use of nutraceuticals is expanding in patients with headache and particularly with migraine. The National Health Interview Study reported that 49,5% of the patients suffering from migraine use nutraceutical products and do not discuss this with their health care providers while only 33.9% of general population use them, .

Headache Therapy Guidelines of Scientific Societies from different countries (USA, Italy, England, Switzerland , etc) consider these products with sometimes conflicting information. Although the understanding of migraine pathophysiology has increased dramatically in recent years, the exact etiology remains to be defined. The current prevailing theory is based on a hyperexcitable “trigeminovascular complex” in patients who are genetically predisposed to migraine. In these people, there is a lowered threshold for migraine attacks and vulnerability to environmental triggers. In susceptible individuals, the trigeminovascular neurons release neurotransmitters, such as calcitonin gene-related peptide and substance P, when headache triggers are encountered. This lead to vasodilation, mast cells degranulation, increased vascular permeability and meningeal edema, resulting in neurogenic inflammation. This nociceptive information is transmitted from the trigeminal nerve to the brainstem nuclei, thalamic nuclei, and the cortex, where migraine pain is ultimately perceived.

Although the mechanisms of action of these molecules in migraine prevention are poorly known, their effects on vessel wall, neurons, as well as on specific neurotransmitters involved in migraine pathophysiology have been hypothesized based on numerous pre-clinical observations.

Many different nutraceuticals are currently used in migraine prophylaxis other than in patients who do not want a pharmacological traditional therapy, in those cases when the pharmacological therapy cannot be used or it is better to avoid it (children, old patients using many different drugs, severe liver or renal diseases, pregnancy, etc), but in any case is important to know the possible even rare side-effects of each one of these and when it is better to avoid them.

### S39 Community pharmacies as epidemiological sentinels of headache

#### Paola Brusa^1,2^, Gianni Allais^3,8^, Cecilia Scarinzi^4^, Francesca Baratta^1^, Marco Parente^1^, Sara Rolando^3^, Roberto Gnavi^4^, Teresa Spadea^4^, Giuseppe Costa^4,^ Chiara Benedetto^3^, Massimo Mana^5,6^, Mario Giaccone^2,7^, Andrea Mandelli^7^, Gian Camillo Manzoni^8^, Gennaro Bussone^8^

##### ^1^Department of Science and Technology of Drugs, University of Turin, via Pietro Giuria 9, 10125, Turin, Italy; ^2^Order of Pharmacists of Turin, Via Sant'Anselmo, 14, 10125, Turin, Italy; ^3^Department of Surgical Sciences, Women’s Headache Center, University of Turin, Via Ventimiglia 3, 10126, Turin, Italy; ^4^Epidemiology Unit, ASL TO3, Via Sabaudia 164, 10095, Grugliasco (Turin), Italy; ^5^ATF Informatics, Via Cascina Colombaro 56, 12100, Cuneo, Italy; ^6^Federfarma Piemonte, Via Sant'Anselmo, 14, 10125, Turin, Italy; ^7^FOFI, Federation of the Orders of Italian Pharmacists, Via Palestro 75, 00185, Rome, Italy; ^8^FI.CEF Onlus, Italian Headache Foundation, via Celoria 11, 20133 Milan, Italy

###### **Correspondence:** Paola Brusa (paola.brusa@unito.it)

**Background**. A study was carried out in Italy with the objective of analysing the patterns of use of drugs in subjects who entered a pharmacy requesting OTC medications in order to treat a headache attack. In this context, the objective is to analyse the association of headaches with socio-demographic and clinical characteristics and with the pathway of care followed by the patients.

**Methods**. a questionnaire was administered to subjects who entered a pharmacy requesting self-medication for a headache attack. The questionnaire consisted of 14 items covering socio-demographic factors, pathway of care and clinical information. The clinical section included the items of the ID Migraine TM Screener (ID-MS), resulting in a four-level variable that classifies patients as suffering from definite migraine, probable migraine, unlikely migraine, or other headaches, based on symptoms, occurrence and severity of headaches. A single national online training course was employed to prepare the pharmacists in order to ensure that the data were collected as homogeneously as possible. At the end of the training course, each pharmacist had to pass a final test before being enrolled in the project.

**Results**. Out of 610 trained pharmacists, 445 collected at least one questionnaire, 4424 questionnaires had been correctly completed. The ID-MS shows a strong association with gender. The prevalence of definite migraines was significantly higher among the two lower educational levels.

Our study, confirming data found in investigations carried out among the general population, indicates that about half of headache sufferers and about a third of migraineurs do not consider their condition as a disease and tend to self-medicate, which leads to inappropriate treatment of the condition.

**Conclusions**. The pharmacy can be a valuable observatory for the study of headaches and can be the first important step in seeking to build a good management relationship for subjects with headache.

**Refrences**

1. Brusa P, Parente M, Allais G et al. Community pharmacies as epidemiological sentinels of headache: first experience in Italy Neurol Sci 2017; 38(Suppl 1): 15-20.

2. Lipton RB, Dodick D, Sadovsky R et al. A self-administered screener for migraine in primary care. The ID MigraineTM validation study. Neurology 2003; 61: 375–382

3. Rapoport AM, Bigal ME. ID-migraine. Neurol Sci 2004; 25:S258–S260

4. Headache Classification Committee of the International Headache Society: The international classification of headache disorders, 3rd edition (beta version). Cephalalgia 2013, 33:629-808.

5. Manzoni GC, Bonavita V, Bussone G, Cortelli P, Narbone MC, Cevoli S, D’Amico D, De Simone R, Torelli P on behalf of ANIRCEF (Associazione Neurologica Italiana Ricerca Cefalee): Chronic migraine classification: current knowledge and future perspectives. J Headache Pain 2011, 12:585-592.

### S40 Digital health and clinical decision support : the HealthSOAF project and the Calabria Headache Network

#### Rosario Iannacchero^1^, Carmela Mastrandrea^1^, Domenico Conforti^2^

##### ^1^Regional Headache Centre , Neurology Division “ Pugliese-Ciaccio “ Hospital Catanzaro Italy; ^2^ Department of Mechanical Energy Management Engineering, University of Calabria Rende Italy

###### **Correspondence:** Rosario Iannacchero (centrocefaleeaopc@gmail.com)

**Introduction:** Good clinical governance of headache implies efficient and accessible diagnostic and therapeutic pathways involving different levels of health care. Headache syndromes are often diagnosed and treated inadequately. Information and communication technology (ICT) can play a key role in improving access to treatment and, from the clinical management perspective, in increasing the levels of quality , efficiency and prevention.The HealthSOAF ( Service-Oriented Architecture Framework ) is a networking and interoperability technology platform, designed to assist healthcare decision making and improve access to care at appropriate levels. The Headache Network operating in the Italian region Calabria provide the HealthSOAF platform with its first real tes bed in Europe, with the aim of helping clinicians working at different levels of health care to correctly diagnose , manage and refer headache patients.

**Materials and Methods :** Between December 2014 and June 2015 primary care general practitioners in Catanzaro Lido, Borgia and Soverato , three secondary care neurologists, and one multidisciplinary tertiary care team from the Headache Centre the Pugliese-Ciaccio Hospital in Catanzaro , used the pilot client software and accessed the HealthSOAF platform.In the period considered , GP recruited 197 patients with headache diagnoses made with the support of techonological platform : 19 ( 9,64%) had a suspected diagnosis of secondary headache and were referred to emergency room ; 74 ( 37,56% ) were diagnosed with episodic primary headache and managed exclusively by GP at primary headache level; 36 ( 18,27% ), also with episodic primary headache, were managed by both GP and outpatient neurologists , again in the primary setting ; 68 patients ( 34,52% ) were sent to the reference headache centre. The preliminary data from the pilot study , showing an approximately 50% reduction in inappropriate referrals to the hospital reference centre ( 15,42% vs 7,35% ), indicate enhanced diagnostic accuracy and appropriateness of referrals within the coded diagnostic, therapeutic and care pathways.

**Conclusion:** The use of ICT support in clinical decision making and management processes is a valuable aid in clinical practice. The HealthSOAF project is the first at a national level and among the first at international level to study , define, test and validate a new approach in the software development cycle, designed to guarantee interoperability of distributed and highly heterogeneous health information system , of the kind that are increasingly necessary to support continuity of care , diagnostic processes , integrated therapeutic care , prevention activities and public health protection.

**References**

1. Commision of the European Communities: An action plan for European e-Health Area 2004

2 European Commision , Horizon 2020 . The EU Framework Programme for Research and Innovation

### S41 Headaches and cranio-cervico-mandibular disorders: EBP manual therapy

#### Riccardo Rosa^1,2^ (clinicadelmalditesta@gmail.com)

##### ^1^La Sapienza University of Roma, Italy; ^2^Clinica del Mal di Testa, via campania 37, Roma, 00161, Italy

Headaches are one of the most disabling disorders [1]. The 3rd edition of the International Classification of Headache Disorders (ICHD-III) describes the diagnostic criteria of primary, secondary and other headache disorder types. Interestingly, Migraine and Cervicogenic Headache (CGH) , Tension Type Headache (TTH), Headache attributed to temporo-mandibular disorder (TMD), Headache attributed to cervical myofascial pain and Occipital Neuralgia share similarities in some criteria and clinical presentation. Moreover, Neck Pain associated disorders (NAD) and Temporo-Mandibular Disorders (TMD) are a very common presentation in primary headache population as Migraine and Tension Type Headache [2,3]. Moreover, recent knowledge have suggested that physical examination for provocative procedures should be done on each patient with sidelocked headaches as many of these headaches may closely mimic primary headaches [4]. There have been identified eleven physical tests to properly assess cervical disorders. When these dysfunctions are present, they support a reciprocal interaction between the trigeminal and the cervical systems as a trait symptom in migraine [6,7,8]. The ICHD-III also does recommend the use of diagnostic criteria evolved by the International RDC/TMD Consortium Network and Orofacial Pain Special Interest Group to assess disorder involving structures in the temporomandibular region contributing to primary headache [9,10]. In this presentation, an evidence based protocol of manual tests and rehabilitation proposal it will be provided by a physiotherapist to assess and treat musculoskeletal disorders associated to the most common primary headaches as Migraine and Tension Type Headache. Moreover, the integration of this examination and approach in a multidisciplinary team it will be discussed [11].

**References**

1. Stovner LJ. Migraine prophylaxis with drugs influencing the reninangiotensinsystem. *Eur J Neurol*. 2007;14(7):713-4. doi:10.1111/j.1468-1331.2007.01760.x.

2. Ashina S, Bendtsen L, Lyngberg AC, Lipton RB, Hajiyeva N, Jensen R. Prevalence of neck pain in migraine and tension-type headache: a population study. *Cephalalgia*. 2015;35(3):211-9. doi:10.1177/0333102414535110.

3. Tomaz-Morais JF, Lucena LB, Mota IA, Pereira AK, Lucena BT, Castro RD,Alves GA. Temporomandibular disorders is more prevalent among patients with primary headaches in a tertiary outpatient clinic. *Arq Neuropsiquiatr*. 2015 Nov;73(11):913-7. doi: 10.1590/0004-282X20150145.

4. Prakash S, Rathore C. Side-locked headache: an algorithm based approach. *The Journal of Headache and Pain* 2016; 17:95

5. Luedtke K, Boissonnault W, Caspersen N, Castien R, Chaibi A, Falla D et al. International consensus on the most useful physical examination tests used by physiotherapists for patients with headache: A Delphi study. Man Ther. 2016;23:17-24. doi:10.1016/j.math.2016.02.010.

6. Luedtke K, Starke W, May A. Musculoskeletal dysfunction in migraine patients. Cephalalgia. 2017; Jan 1:333102417716934. doi:10.1177/0333102417716934.Headache Classification Committee of the International Headache Society (IHS). The International Classification of Headache Disorders, 3rd edition (beta version) 2013 Jul;33(9):629-808. doi: 10.1177/0333102413485658.

7. Luedtke and May A. Stratifying migraine patients based on dynamic pain provocation over the upper cervical spine. *The Journal of Headache and Pain* (2017) 18:97 DOI 10.1186/s10194-017-0808-0

8. Headache Classification Committee of the International Headache Society (IHS). The International Classification of Headache Disorders, 3rd edition (beta version) 2013 Jul;33(9):629-808. doi: 10.1177/0333102413485658.

9. Schiffman E, Ohrbach R, Truelove E, et al. Diagnostic Criteria for Temporomandibular Disorders (DC/TMD) for Clinical and Research Applications: Recommendations of the International RDC/TMD Consortium Network and Orofacial Pain Special Interest Group. *Journal of oral & facial pain and headache*. 2014;28(1):6-27.

10. Garrigòs-Pedròn M, La Touche R, Navarro-Desentre P, Gracia-Naya M, Segura-Ortì E. Effects of a Physical Therapy Protocol in Patients with Chronic Migraine and Temporomandibular Disorders: A Randomized, Single-Blinded, Clinical Trial. *J Oral Facial Pain headache***.** 2018 Spring;32(2):137-150. doi:10.11607/ofph.1912.

### S42 Spontaneous intracranial hypotension headache

#### Enrico Ferrante (enricoferrante@libero.it)

##### Neurology Department San Carlo Hospital- Potenza

Schaltenbrand, in 1938 first described spontaneous intracranial hypotension (SIH). In 1991 Mokri, published the first report on diffuse pachymeningeal enhancement (DPE) in SIH. In 1993 Ferrante, published the first Italian report on SIH. SIH results from spinal CSF leaks, sometimes asociated with underlying connective tissue disorders. The decrease in CSF volume is compensated by an increase in intracranial venous blood volume according to the Monro-Kellie doctrine. The associated prevalence and incidence has been estimated at 2–5:100,000. Female are affected twice to fifth as often as males. The peak incidence is in 30–50 year [1]. The triggers for the symptoms can be trivial or minor; however, no clear precipitant was identified in most cases. Orthostatic headache (OH), generally occipital-nuchal bilaterally, worsening during Valsalva manoeuvres, is the main symptom of SIH. In our case series over 400 cases we have observed about 70% of cases with cochlear-vestibular signs associated with OH and seven patients who had only a Valsalva manoeuvre headache. The diagnostic criteria for SIH are described in the International Classification of Headache Disorders, (ICHD) 3rd edition. Brain CT is often normal while brain MRI generally shows the indirect SIH signs (DPE, subdural fluid collections, venous structures engorgement, pituitary hyperemia, and sagittal sagging). Spinal MRI may show cervical pachymeningeal enhancement, spinal epidural collection, or fluid collection in cervical soft tissues, meningeal diverticula, dilated nerve root sleeves, and epidural venous plexus engorgement. MRI myelography is more sensitive to identify a spinal CSF leak. CT myelography should be reserved for patients without identified leak on spinal MRI, who have failed to respond to two or three EBPs, and in whom a targeted EBP at the site of CSF leak is being considered. In 15–30% of cases, SIH is resolved spontaneously or with conservative treatment (bed rets and overhydration) within a period of 1–2 weeks. The success rate with each EBP is variable (30% -90%) [2]. In our experience, the EBP success depends on the adequate volume of blood injected, which has to be sufficient to fill the epidural space, and on strict bed rest in Trendelenburg position for at least 8 hours after the procedure. Targeted EBP in the upper thoracic or cervical regions or surgical approaches could be useful if two or three attempts at a lumbar EBP have failed and the CSF leak site is located.

**References**

1) Mokri B. Spontaneous Intracranial Hypotension. Continuum (Minneap Minn) 2015;21(4):1086–1108.

2) Ferrante E, Arpino I, Citterio A, Wetzl R, Savino A. Epidural blood patch in Trendelenburg position pre–medicated with acetazolamide to treat spontaneous intracranial hypotension. Eur J Neurol 2010, 17: 715–19.

### S43 The standard of care for chronic migraine in lombardia SISC centres: results of a collaborative study

#### Paola Merlo^1*^, Valentina Rebecchi^2^, Natascia Ghiotto^3^, Lucia Princiotta Cariddi^2^, Fabio Antonaci^4,5^, Andrea Giorgetti^6^, Franco Di Palma^7^, Edgard Matta^8^, Giorgio Dalla Volta^9^, Grazia Sances^3^ on behalf of SISC - Società Italiana per lo Studio delle Cefalee - Sezione Lombardia, Italy

##### ^1^UO Neurologia - Centro Cefalee, Humanitas Gavazzeni, Bergamo; ^2^Centro Cefalee UOC Neurologia –Varese - ASST Settelaghi – Univ. Insubria; ^3^Headache Science Centre, National Neurologic Institute C. Mondino, Pavia; ^4^UC Neurologia Speciale d'Urgenza, Istituto Neurologico Nazionale, Pavia; ^5^Dipartimento di Scienze del Sistema Nervoso e del Comportamento Università di Pavia; ^6^Centro Cefalee, Dipartimento di Neuroscienze H di Legnano ASST Ovest milanese; ^7^Centro Cefalee UOC Neurologia della ASST Lariana - Ospedale S. Anna, Como; ^8^Centro Cefalee UOC Neurologia ASST Bergamo ovest; ^9^Centro Cefalee UO Neurologia - Istituto Clinico Città di Brescia, Brescia

###### **Correspondence:** Paola Merlo (paola.merlo@gavazzeni.it)

**Background**

Although the diagnosis of Chronic Migraine with and without Medication Overuse (CM/CM-MOH) are based on well-established criteria (ICHD-III β) the definition of international guidelines for its management is still work in progress, especially as far as the long term management and the sustainability of the cost/benefit ratio are concerned. Thus, the present observational study was aimed to evaluate methods for CM management among different headache structures with a common cultural background and SISC standards (local resources, structural standards, operators qualification).

**Methods**

This study involved different neurological structures belonging to SISC-Lombardia and operating in or for Italian National Health System, that boast a documented practice in headache management, adherence to the locally approved protocols for CM, open access to detoxification programs and to onabotulinumtoxin A treatment. As far as the patients are concerned, they had to have a history of CM for at least 2 years prior to screening and to be willing and able to return to the clinic for the follow-up evaluations.

**Results**

We enrolled a population of 1940 patients over one year: 1140 (58.7%) had been diagnosed with CM and 800 (41.2%) with CM-MOH. In the CM-MOH group 685 patients (85.6%) underwent pharmacological detoxification treatment during the previous year. There are no great differences in the assessment methods and treatment plans among the structures: headache diaries are used everywhere (100%), while disability assessments are scored with MIDAS and HIT-6 questionnaires in 62.5% and 50% respectively. A protocol for detoxification was performed as inpatients (87.5%) or outpatients (12.5%). The average duration of detoxification was 5-7 days. Pharmacological methods for detoxification and the treatment of rebound headache showed some differences in the kind of drugs. In 3 structures (37.5%) a prophylaxis treatment with Onabotulinumtoxin A every 3 months followed detoxification (122 of CM-MOH, 15.25%). The follow-up methods were not homogeneous and showed controversial results; the same methods and parameters were used to evaluate the effectiveness of treatment but with different scheduling: the follow up period implied a 3 (37.5% of the Centres), 6 (25%) or 12 (37.5%) months evaluation. In one centre the follow-up included 3-6-12 months clinical controls for each patient. A strong variability was found in the response to treatments: the detoxification and prophylaxis treatment were effective from 20 to 97%; onabotulinumtoxin A alone was effective from 40% to 70%.

**Conclusion**

Our data showed a similar clinical approach in management of CM/CM-MOH. On the other hand the different therapeutic methods showed a lower concordance both for the clinical follow-up and to the evaluation of the treatments among the various structures. There was more agreement in the Onabotulinumtoxin A efficacy among the different centres.

**References**

1. MA Giamberardino, MD Dimos-Dimitrios Mitsikostas, MD Paolo Martelletti. Update on medication-overuse headache and its treatment. Curr Treat Options Neurol. 2015; 17:37

2. C Chiang, TJ Schwedt, S Wang, DW Dodick. Treatment of medication-overuse headache: a systematic review. Cephalagia 2016; 36(4): 371.

3. C Tassorelli, R Jensen, M Allena, R De Icco, G Sances, Z Katsarava, M Lainez, JA Leston, R Fadic, S Spadafora, M Pagani, G Nappi and the COMOESTAS Consortium. A consensus protocol for the management of medication-overuse headache: Evaluation in a multicentric, multinational study

4. C Tassorelli, G Sances, M Avenali, R De Icco, D Martinelli, V Bitetto, G Nappi, G Sandrini. Botulinum toxin for chronic migraine: Clinical trials and technical aspects. Toxicon. 2018 Jun 1;147:111-115.

### S44 Migraine pathophysiology

#### Gianluca Coppola (gianluca.coppola@gmail.com)

##### Sapienza University of Rome Polo Pontino, Department of Medico-Surgical Sciences and Biotechnologies, Latina, Italy

Presently, migraine is considered a brain disorder. Many independent research groups have observed that the brain of migraine patients abnormally processes information of any sensory modality. These functional abnormalities are under continuous fluctuations following the migraine cycle and the frequency of the attacks and are under the influence of endogenous and environmental factors. Recent evidence provided by modern neuroimaging techniques tends to show that plastic (mostly reversible) changes in brain metabolism, connectivity, and micro-/macro- structure accompany functional abnormalities. However, whatever the origin of these cerebral morpho-functional abnormalities, migraine clinical manifestation requires ignition of the central and peripheral trigeminal system. The heterogeneous clinical presentation of visual migraine aura symptoms runs in parallel with heterogeneous BOLD fMRI responses within the visual areas. Malfunctioning descending pain control systems in the frontal cortex and brainstem, and abnormal thalamic and hypothalamic connectivity- alone or in combination- seem to be major permissive interictal factors for the preictal cascade of events that leads to sequential sensitization of first- and/or second-order trigeminovascular nociceptors resulting in transient (episodic migraine) or persistent (in CM) central sensitization.

### S45 The use of a phytotherapic compound containing Tanacetum Parthenium and Andrographis, in combination with CoQ10 and Riboflavin, for migraine prophylaxis: a randomized double blind versus placebo clinical trial

#### Cherubino Di Lorenzo (cherub@inwind.it)

##### IRCCS Fondazione Don Carlo Gnocchi, Milan, Italy

**Background.** The most of migraineurs patients referred to a headache clinic for a specialist consultation is characterized by a middle to high frequency of migraine attacks. These patients need prophylactic treatments to reduce the burden of disease in terms of attacks, days with headache, and symptomatic drugs consumption. So far, pharmacologic prophylactic treatments are characterized by a sub-optimal efficacy due to the high number of patients that do not respond to the treatments, and the elevated incidence of side effects. To match the patient needs, waiting for the next generation treatments, the use of herbal medicine is very common among migraine population. In the last years, phytoextracts of feverfew (tanacetum partenium) were studied and adopted to treat migraineurs. In particular, there is a fixed combination of Tanacetum Parthenium, Andrographis, Coenzyme Q10, and Riboflavin, that is widely used in Italy. In order to verify the efficacy of that association, we designed a randomized double blind versus placebo clinical trial. Here we present the preliminary results of our study.

**Results.** Twenty-four patients were enrolled and randomly assigned to receive the verum or the placebo treatment for 3 months. Each treatment kit, blinded by a unique code that was coupled to each patient, was composed by 120 pills, enough for 3 months: (1 pill b.i.d. in the first month, 1 per day in the second and third month). Eleven of twelve patients assigned to the verum arm of the study improved their headache, with a responder rate (reduction of at least 50% of headaches) of 60%. On the contrary, only 4/12 patients that received the placebo treatment improved their headache, with a 0% responder rate. No side effects were reported.

**Conclusions.** The traditional use of herbal medicine is as old as the history of medicine and that ancient practice is present in almost all cultures. Our results show that the examined combination is effective in the migraine prophylaxis if compared to placebo and safe if compared with data about synthetic drugs, as they are reported in literature.

### S46 Migraine and Metabolic Syndrome

#### Cherubino Di Lorenzo (cherub@inwind.it)

##### IRCCS Fondazione Don Carlo Gnocchi, Milan, Italy

**Background.** Although migraine is a neurological disorder in which a peripheral trigemino-vascular activation is elicited in response to central sensitization processes, several studies payed attention on metabolic features among migraineurs, and their influence on migraine clinical characteristics.

**Results.** By a literature review, it is possible to assess that MetS is more prevalent in migraineurs (21.8% with aura, 16.8% without aura) than in controls (14.5%), and that it is related to the development of a chronic form of headache, the so-called ‘medication overuse headache’. These data are consistent to epidemiological studies that evidenced an increased risk to develop cerebrovascular disorders (CVDs) in migraineurs patients (both with and without aura), since CVDs are the more expected outcome of MetS. It is well known that obesity and hypertension are risk factors for migraine development and its chronification, while their improvement leads to a migraine improvement. Besides, the most of attention was recently payed to the oral glucose tolerance test to examine insulin sensitivity. In migraineurs patients, after glucose load, there is a significant increase of both plasmatic insulin and glucose concentrations in comparison with controls, while is common to find in migraineurs a very late hypoglycemic response that may trigger the migraine attack. The role of hypoglycemia, one of the early characteristics of the so-called insulin-resistance, is indirectly confirmed by the observation that the development of diabetes (characterized by a state of stable hyperglycemia) seems to be protective against migraine attacks. The insulin-resistance is characterized by an alteration of insulin receptor signaling that has shown to be also altered in migraineurs. All these metabolic abnormalities are related to the low-grade inflammation that could, in turn, be related to release of inflammatory cytokines (among these, the CGRP) that lead to the migraine attack.

**Conclusions.** One of the theories to explain the high prevalence of MetS among the population is the so called “thrifty gene theory”. According to this theory, the high levels of well-being of western population are related to an increase of nutritional intake that has stressed the metabolism of people genetically fitted to survive in famine, leading to MetS development. If common genetic and biological backgrounds underpin both migraine and MetS, according to this theory is possible to explain the high prevalence of migraine among general population.

### S47 Migraine and Ketogenic Diet

#### Francesco Pierelli^1^, Gianluca Coppola^2^, Cherubino Di Lorenzo^3^

##### ^1^INM Neuromed IRCCS, Pozzilli (IS), Italy; ^2^G. B. Bietti Foundation-IRCCS, Department of Neurophysiology of Vision and Neurophthalmology, Rome, Italy; ^3^IRCCS Fondazione Don Carlo Gnocchi, Milan, Italy

###### **Correspondence:** Francesco Pierelli; Cherubino Di Lorenzo (cherub@inwind.it)

**Background.** Ketogenic diet (KD) is a dietetic regimen characterized by a very low intake of carbohydrate in order to induce the metabolic ketosis (the physiologic endogenous production of ketones) to replace sugars as energetic substrate for the brain. Ketones are a very efficient fuel for the brain, since they induce to an increase of ATP production with a reduction of oxidative stress related to mitochondrial activity. On the other hand, they have an anti-inflammatory effect and are able to modulate the cortical excitability by their direct and indirect GABAergic effects. Several different types of KDs wer developed across the decades, all characterized by the low glycemic intake. They could be normo-caloric (rich in fats that have to replace the missing carbohydrate in the caloric balance), or hypocaloric (poor in fats to induce the lepidic metabolism from adipose tissue). Since 1921 this diet was proposed to treat drug resistant epilepsy in children. Even if in 1928 and in 1930 two papers reported about the efficacy of KDs in migraineurs patients, they did not meet the expected success, since at that time several symptomatic treatments were already available. Only in the recent years, the awareness about symptomatic overuse related troubles, and the efforts to reduce the number of treatment resistant patients, seems to rekindle the interest in this procedure.

**Results.** Our group started to study the application of KDs in the field of headache since the 2009, using both normo-caloric and hypocaloric, according to patients’ exigences. We found the KD an useful tool in the treatment of patients with migraine, both in episodic and chronic form, and in cluster headache patients. On the contrary, KD seems to be ineffective in tension type and cervicogenic forms of headache.

**Conclusions.** According to early literature reports, KDs seem to be a useful opportunity to improve the quality of life of our patients. One possible application is, for instance, the weight reduction (that could improve *per se* migraine), maybe induced by some prophylactic treatments. Another opportunity given by KD is the improvement of headache in drug-resistant patients. As future perspective, a there is a wide interest in the use of exogenous ketones, the so called “ketogenic diet in pills”.

### S48 Headache attributed to disorder of homoeostasis

#### Federico Mainardi (fmainardi@iol.it)

##### Headache Centre, Neurological Division, SS Giovanni e Paolo Hospital, Venice, Italy

As in the previous version, chapter 10 of the current International Classification of Headache Disorders (ICHD 3) [1] is dedicated to the forms of headache attributed to disorders of homeostasis. All the nosographic entities of the provisional ICHD 3 beta version (2013) [2] are confirmed in this final edition, both in their digit code and in their diagnostic criteria.

The main rehash of chapter 10 was performed in the passage from ICHD-1 (1988) [3] to ICHD-2 (2003) [4], when the chapter was extensively remodeled. Hypoxic and hypercapnic headaches were unified in a unique headline (10.1), including *High altitude headache* (10.1.1), *Diving headache* (10.1.2) and *Sleep apnoea headache* (10.1.3). Moreover, *Headache attributed to arterial hypertension* and its subtypes, previously collocated in chapter 6 - *Headaches associated to vascular disorders*, were moved at the third digit of the first level (10.3), between *Dyalisis headache* (10.2) and *Headache attributed to hypothyroidism* (10.4). *Headache attributed to fasting* (10.5) substituted the older denomination “*Hypoglicemia”*, and the new *Cardiac cephalalgia* (10.6), a rare occurrence characterized by a close temporal relationship between the onset of headache and cardiac ischemia, appeared for the first time.

In 2013, the provisional ICHD 3 [2] confirmed the structure of the chapter. The main modifications regarded the merger of *Headache attributed to pre-eclampsia* and *eclampsia* in an unique digit (10.3.4), the disappearance of *Headache attributed to acute pressor response to an exogenous agent*, and the addition of two new entities.

The first one, *Headache attributed to aeroplane travel* (10.1.2), placed together with other forms of headache attributed to hypoxia and/or hypercapnia, is characterized by very severe unilateral pain attacks that appear during take-off or, more frequently, landing of the aeroplane, lasting up to 30 min after the ascent or descent of the vehicle is completed. Recently, the coexistence of headache attacks with the same features but triggered by the rapid ascent after free/scuba diving and/or the descent from high mountain altitude has been described and suggests a possible common causal mechanism, that is an imbalance between intrasinusal and external air pressure [5].

*Headache attributed to autonomic dysreflexia* (10.3.5), the second new entry, appears in the subchapter dedicated to *Headache attributed to arterial hypertension*. These sudden-onset severe headache attacks, accompanied by symptoms of autonomic dysfunction and triggered by stimuli of visceral or somatic origin (including bladder or bowel distension, pressure ulcers, trauma or surgical diagnostic procedures), affect patients with spinal cord injury.

**References**

1. Headache Classification Committee of the International Headache Society (IHS). The International Classification of Headache Disorders, 3^rd^ edition. Cephalalgia 2018; 38: 1-211

2. Headache Classification Committee of the International Headache Society. The International Classification of Headache Disorders: 3^rd^ edition (beta version). *Cephalalgia*. 2013; 33: 629-808

3. Headache Classification Committee of the International Headache Society. Classification and diagnostic criteria for headache disorders, cranial neuralgia and facial pain. Cephalalgia 1988;8(suppl 7):1-96.

4. Headache Classification Subcommittee of the International Headache Society: The International Classification of Headache Disorders, 2nd edn. Cephalalgia 2004;24(Suppl 1):1-160.

5. Mainardi F, Maggioni F, Zanchin G. Aeroplane headache, mountain descent headache, diving ascent headache. Three subtypes of headache attributed to imbalance between intrasinusal and external air pressure? Cephalalgia 2018;38:1119-1127. Epub ahead of print 8 August 2017. DOI: 10.1177/033310417724154.

### S49 Chronic primary headaches

#### Paolo Rossi (paolo.rossi9079@gmail.com)

##### INI Grottaferrata

According to the ICHD-3 classification primary headaches may exist in episodic and chronic form. Chronic headaches have a significantly greater effect on quality of life than episodic headaches. Chronic headaches patients are more difficult to manage and more prone to miss work or have decreased productivity. They have more comorbidity including psychiatric disorders and use more health resources than the episodic ones. The most prevalent subtypes of chronic headaches are chronic migraine and chronic tension-type headaches. Migraine and tension-type headaches are defined as chronic when the headache occur on more than 15 days/month for more than 3 months. In both forms the chronification of the headache is usually the result of a process of transformation under the influence of pain system sensitizing factors such as recurring untreated headaches, medication overuse, comorbid pain or psychiatric disorders and others. When the headache is chronic from the onset and very soon unremitting the diagnosis should be oriented to the New Daily Persistent Headache a syndrome that should be rapidly identified because can transform in a highly refractory headache. This review will focus on some relevant unanswered question on chronic headaches: 1) Is the new classification of the pain disorders helpful for the management of these patients ? 2) are the mechanism of transformation of the headache into a chronic form a potential therapeutic target ? 3) is the comorbidity a potential therapeutic target ?

### S50 Nocturnal onset headache

#### Carlo Lisotto (carlo.lisotto@aas5.sanita.fvg.it)

##### Headache Centre, Department of Neurology, Azienda Sanitaria Friuli Occidentale, Pordenone, Italy

Headache and sleep disturbances represent some of the most commonly reported disorders in clinical practice, causing considerable individual disability and socio-economical burden. The link between headache and sleep includes five different possible types of relationship : 1. headache disorders occurring exclusively or mainly during sleep; 2. sleep disorders directly causing headache; 3. sleep disturbances caused by headache; 4. comorbidity of sleep disturbances and headache disorders; 5. underlying disorders leading both to headache and sleep disturbances [1]. A preferential or exclusive occurrence of attacks at night-time is well documented in some primary headaches, such as migraine, cluster headache (CH) and hypnic headache (HH). In case of recent-onset nocturnal headaches, it is mandatory to rule out a secondary form, in particular intracranial hypertension and uncontrolled arterial hypertension. The occurrence of attacks at night-time or in the early morning has been extensively ascertained in migraine without aura. The preferential emergence of migraine attacks during sleep and/or upon awakening progressively increases in relation to the age of patients. The percentage of subjects with sleep-related migraine was found to be 16% in patients aged 20-30 and 58% in migraineurs aged 60-70, respectively [2]. The association between CH and sleep has been long recognised, due to the extremely frequent occurrence of attacks during nocturnal sleep. In the majority of patients attacks occur with “clock-like regularity” at particular times during 24 hours, often awakening patients from sleep. Approximately 75% of CH attacks occur between 9 pm and 10 am. Attacks usually occur about 90 minutes after the patient falls asleep, which coincides with the first REM sleep, although this is less apparent in chronic CH [3]. HH is a primary headache that is characterized by recurring headache attacks developing only during sleep (occurring mostly between 2 am and 4 am), generally in the elderly population. HH is significantly more common in women. The pain is usually mild to moderate, but may be severe, whereas the duration of attacks ranges from 15 to 240 minutes. Most HHs are bilateral and the most mentioned location of pain are the fronto-temporal area or holocranial/diffuse. The natural course of HH remains unknown. Some patients show an episodic course, while other subjects present and episodic-relapsing remitting trend over time. A history of migraine is common in HH patients, possibly due to a common pathophysiological predisposition. In some patients HH appears to be as an evolution from a pre-existing migraine condition over time.

**References**

1. Freedom T, Evans RW. Headache and sleep. Headache 2013;53: 1358-1366.

2. Gori S, Lucchesi C, Morelli N, Maestri M, Bonanni E, Murri L. Sleep-related migraine occurrence increases with aging. Acta Neurol Belg 2012;112: 183-187.

3. Rains JC, Poceta JS. Sleep-related headaches. Neurol Clin 2012;30: 1285-1298.

### S51 Headache and Physiotherapy

#### César Fernández-de-las-Peñas (cesar.fernandez@urjc.es)

##### Departamento de Fisioterapia, Terapia Ocupacional, Rehabilitación y Medicina Física, Universidad Rey Juan Carlos, Alcorcón, Madrid, Spain

Headache is the medical problem most commonly observed by neurologists. Non-pharmacological treatments, particularly physical therapy, are commonly demanded by individuals with headaches, but their scientific evidence of effectiveness is conflicting. The current lecture will provide an updated discussion on what is supported by current scientific evidence about physical therapy for tension type headache (TTH), migraine, or cervicogenic headache (CeH) and which gaps there still may be in our understanding of these interventions. Today, there are several physical therapies including spinal joint manipulation/mobilization, soft tissue interventions, therapeutic exercises and needling therapies that are proposed to be effective for the management of headaches. Current evidence has shown that the effectiveness of these interventions will depend on proper clinical reasoning since not all interventions are equally effective for all headache pain conditions. For instance, since the pathogenesis of TTH is associated to musculoskeletal disorders, particularly muscular impairments, manual therapies targeting muscle trigger points and other related tissues may be effective for this headache. On the contrary, the evidence of physiotherapy in migraine is less robust than for TTH, which seems to be expected since migraine pathogenesis involves activation of sub-cortical structures and the trigemino-vascular system. Finally, since CeH may be more related to upper cervical spine, spinal manipulations or mobilizations should be the therapeutic options. In fact, the inconsistent results in the current literature can be related to the fact that maybe not all therapeutic interventions are appropriate for all patients with TTH, migraine or CeH, or maybe not all individuals with a particular headache will benefit from a specific intervention. In addition, multimodal approaches including different interventions are more effective than isolated therapeutic approaches for with TTH, migraine and CeH. All these topics will be further discussed in this plenary lecture.

### S52 Migraine and Psychiatric Comorbidity

#### Maria Pia Prudenzano

##### Headache Center, “L. Amaducci” Clinic, Department of Basic Medical Sciences, Neurosciences and Sense Organs, University of Bari, Italy

Most of the studies concerning psychiatric comorbidity in migraine have focused on anxiety and depression disorders. According to these investigations results, migraineurs are two to four more likely to suffer from depression than non-migraineurs. When considering anxiety disorders, panic disorder and generalised anxiety disorder have been found more frequently in migraine than in non-migraine individuals. Rates of psychiatric comorbidity are higher in chronic when compared with episodic migraine. More recently also other psychiatric disorders have gained attention. A meta-analysis found a migraine prevalence of 34,8% in subjects with bipolar disorder. Post-Traumatic Stress Disorder was found to be more prevalent in migraine patients than in the general population. Psychiatric comorbidity is associated with more severe migraine symptoms and disability. Longitudinal studies demonstrated that the comorbidity with anxiety and depression may be a risk for migraine chronification. Available data supports a bidirectional relationship between migraine and depression. Several other theories have been proposed to explain psychiatric comorbidity of migraine. Migraine and comorbid disorders might share a genetic or environmental risk factor producing a brain state which predisposes to both disorders. Neurotransmitter dysfunctions, inflammatory activity, hormonal influences, sensitization of both sensory and emotional brain network might all contribute. Neuroimaging studies in migraineurs show structural, functional and connectivity abnormalities of brain regions playing an role in emotional response to pain, affectivity and mood. Similar findings were obtained with analogous methods in people with psychiatric disorders. Further investigations are needed to determine if such imaging findings might express a brain state predisposing to both migraine and psychiatric comorbidity, if they might be the result of recurrent migraine attacks enhancing emotional responses and leading to psychiatric comorbidity or if the psychiatric disease might induce both these brain changes and altered emotional responses to painful stimuli. Comorbid psychiatric conditions should always be screened by appropriate diagnostic interviews and tools before planning a treatment for a patient with migraine. In the case of psychiatric comorbidity, drugs that are known to be effective for migraine prevention but also to worsen the comorbid psychiatric disorder should be avoided (i.e. topiramate in migraine and depression). Drugs able to control both disorders, possibly in monotherapy, should be preferred (i.e. propranolol in migraine and mild anxiety). The migraine treatment plan might need to be discussed and coordinated with a psychiatrist. An integrated non pharmacologic and a behavioural therapy may be combined with drug therapy to obtain a synergistic effect with enduring benefits.

**References**

1. Buse DC, Silberstein SD, Manack AN, Papapetropoulos S, Lipton RB. Psychiatric comorbidities of episodic and chronic migraine. J Neurol. 2013; 260(8):1960-9.

2. Seng EK, Seng CD. Understanding migraine and psychiatric comorbidity. Curr Opin Neurol. 2016;29(3):309-13

3. Minen MT, Begasse De Dhaem O, Kroon Van Diest A, Powers S, Schwedt TJ, Lipton R, Silbersweig D. Migraine and its psychiatric comorbidities. J Neurol Neurosurg Psychiatry. 2016 Jul;87(7):741-9

## EHF Oral Presentation

### O1 New pharmacology, expression profiles and probes of CGRP receptors

#### Debbie L. Hay^1^ (dl.hay@auckland.ac.nz)

##### School of Biological Sciences, The University of Auckland, Auckland, New Zealand

There is high interest in blocking the actions of the neuropeptide calcitonin gene-related peptide (CGRP) for the treatment of migraine and other headache disorders. Small molecule antagonists and antibodies that target CGRP itself or a CGRP receptor have been developed for clinical use. Most efforts have focussed on the canonical CGRP receptor, comprising the calcitonin receptor-like receptor (CLR) with receptor activity-modifying protein 1 (RAMP1). However, CGRP can potently activate a closely-related receptor; the AMY_1_ receptor, which is a complex of RAMP1 with the calcitonin receptor (CTR). Very few studies have directly compared the pharmacology and expression profile of both of these receptors and the relevance of the AMY_1_ receptor to CGRP biology is still an open question.

We profiled the affinity of a peptide antagonist (CGRP_8-37_) and four small molecule antagonists (olcegepant, telcagepant, MK-3207 and rimagepant) at both CGRP-responsive receptors. Selectivity of these antagonists between these two receptors varied but was generally lower than expected. We developed a novel high affinity peptide antagonist of both CGRP-responsive receptors to use as a probe for global blockade of CGRP receptor activity. We also expanded the molecular toolbox for studying both CGRP receptors through the synthesis of fluorescent peptides and validation of antibodies for CTR and RAMP1. Using these tools, we have been probing the brain for AMY_1_ receptor expression.

The data show that many CGRP receptor antagonists are not highly selective for the CLR/RAMP1 CGRP receptor and have significant affinity at the CTR/RAMP1 AMY_1_ CGRP-responsive receptor. Our studies are providing new insights into the pharmacology and spatial relationships of CGRP receptors in physiologically relevant tissues, and collectively suggest that the AMY_1_ receptor could be of importance to CGRP activity in migraine.

### O2 Efficacy, Safety, and Tolerability of Orally Administered Atogepant for the Prevention of Episodic Migraine: Results from a Phase 2b/3 Study

#### Peter J. Goadsby^1^ David W. Dodick,^2^ Joel M. Trugman,^3^ Michelle Finnegan,^3^ Hassan Lakkis,^3^ Kaifeng Lu,^3^ Armin Szegedi^3^

##### ^1^NIHR-Wellcome Trust King’s Clinical Research Facility, King’s College London, UK; ^2^Mayo Clinic, Phoenix, AZ, USA; ^3^Allergan plc, Madison, NJ, USA

###### **Correpondence:** Peter J. Goadsby (kuang_amy@allergan.com)

**Objectives:** Atogepant is a novel, oral CGRP receptor antagonist in development for the prevention of migraine. This study evaluated the efficacy, safety, and tolerability of atogepant versus placebo for the prevention of episodic migraine.

**Methods:** Multicenter, randomized, double-blind, placebo-controlled, parallel-group study (NCT02848326). Adult patients with a history of migraine, with or without aura, were included. Patients with 4-14 migraine days in the 28-day baseline period were randomized 2:1:2:1:2:1 to placebo, atogepant 10 mg QD, 30 mg QD, 30 mg BID, 60 mg QD, or 60 mg BID, respectively, and treated for 12 weeks for the prevention of episodic migraine. Primary efficacy endpoint was change from baseline in mean monthly migraine days across the 12-week treatment period. Safety and tolerability were evaluated.

**Results:** Of patients randomized (n=834), 825 were in the safety population, and 795 were included in the primary efficacy population. Mean age was 40.1 years; majority white (76.1%), female (86.5%), and had not taken preventive treatment for migraine in the past (n=593, 71.9%). At baseline, patients reported an average 7.67 (SD=2.49) migraine days. Mean change in migraine days across the 12-week treatment period (adjusted p-values for comparisons versus placebo): placebo (-2.85), atogepant 10 mg QD (-4.00, p=0.0236), 30 mg QD (-3.76, p=0.0390), 60 mg QD (-3.55, p=0.0390), 30 mg BID (-4.23, p=0.0034), 60 mg BID (-4.14, p=0.0031). A total of 480 patients (58.2%) reported treatment-emergent adverse events (AEs); 170 (20.6%) were considered treatment-related. The most common treatment-emergent AEs were nausea, fatigue, constipation, upper respiratory tract infection, nasopharyngitis, urinary tract infection, and blood creatine phosphokinase increase (reported in >5% of patients in at least one treatment group). Seven patients (0.8%) reported serious AEs; none were considered related to treatment. Following daily dosing, 11 cases of ALT/AST elevations ≥3x the upper limit of normal were reported; the number of cases were balanced across treatment groups (placebo [n=3], 10mg QD [n=2], 30mg QD [n=1], 60mg QD [n=3], 30mg BID [n=1], 60mg BID [n=1]).

**Conclusions:** All 5 atogepant treatment arms showed statistically significant differences from placebo in reductions from baseline in mean migraine days across the 12-week treatment period. Reductions in mean migraine days and treatment differences versus placebo were clinically relevant. Atogepant was well-tolerated with no treatment-related serious adverse events.

### O3 Alterations in CGRP and PACAP38 levels in cluster headache

#### Agneta Snoer^1&^, Anne Luise Haulund Vollesen^1&^, Rasmus Paulin Beske^1^, Song Guo^1^, Jan Hoffmann^2^, Jan Fahrenkrug^3^, Niklas Rye Jørgesen^4,5^, Torben Martinussen^6^, Rigmor Højland Jensen^1^, Messoud Ashina^1^

##### ^1^Danish Headache Center and Department of Neurology, Rigshospitalet Glostrup, Faculty of Health and Medical Sciences, University of Copenhagen, Denmark; ^2^Department of Systems Neuroscience, University Medical Center Hamburg-Eppendorf, Hamburg, Germany; ^3^Department of Clinical Biochemistry, Bispebjerg Hospital, University of Copenhagen, Denmark^; 4^Department of Clinical Biochemistry, Rigshospitalet Glostrup, Denmark^; 5^OPEN, Odense Patient Data Explorative Network, Odense University Hospital/Institute of Clinical Research, University of Southern Denmark, Odense, Denmark^; 6^Section of Biostatistics, University of Copenhagen, Denmark

###### **Correspondence:** Messoud Ashina (ashina@dadlnet.dk)

& The first two authors contributed equally to the study

**Background and aim:** A key element in the cluster headache (CH) attack is activation of the trigemino-autonomic reflex of which the vasoactive peptides calcitonin gene-related peptide (CGRP), vasoactive intestinal polypeptide (VIP) and pituitary adenylate cyclase-activating polypeptide-38 (PACAP38) are known mediators. Here, we investigated these peptides during CGRP induced CH attacks.

**Methods:** We included patients with episodic CH in bout (eCHa)-, in remission (eCHr) and chronic patients (cCH). The study was conducted as a randomized, double-blind, placebo controlled, two-way cross-over study during which we measured CGRP, VIP and PACAP38 at baseline, in response to CGRP and placebo infusion. In addition, we compared our baseline findings with historical data on migraine patients and healthy controls.

The study was approved by the Regional Committee on Health Research Ethics of the Capital Region (H-15006836) and registered at clinicaltrials.gov (identifier NCT02466334).

**Results:** In total 31 (9 eCHa, 9 eCHr and 13 cCH) patients completed the study. Blood samples from 11 CGRP induced CH attacks were collected. At baseline CGRP levels were significantly higher in eCHr patients compared to cCH patients (100.6 ± 36.3 pmol/l vs. 65.9 ± 30.5 pmol/l, p=0.0114), and post-hoc analyses revealed significantly higher CGRP levels in eCHa (88.4 ± 29.1 pmol/l, p<0.0001), eCHr (p<0.0001) and cCH (p=0.0202) compared to controls (44.8 ± 11.4 pmol/l). PACAP38 levels in eCHa patients were significantly higher compared to cCH patients (4.0 ± 0.8 pmol/l vs. 3.3 ± 0.7 pmol/l, p=0.0326). We found no change in PACAP-38 levels in response CGRP infusion or to CH attacks. There was no difference in baseline levels of VIP in between CH group. CGRP infusion caused a significant increase in VIP (p<0.0001), but no additional increase was seen in response to attack.

**Conclusion:** This is the first study to investigate CGRP, PACAP38 and VIP in all three disease states. The lower baseline levels of CGRP in cCH patients suggest basic pathophysiological differences in between phenotypes.

### O4 Medication overuse in a post-hoc analysis of phase 3 placebo-controlled studies of galcanezumab in the prevention of episodic and chronic migraine

#### Sheena K. Aurora, Paula A Morrow, Dustin D Ruff, Eric Pearlman

##### Eli Lilly and Company, Indianapolis, IN 46285, USA

###### **Correspondence:** Sheena K. Aurora (sheena.aurora@lilly.com)

**Background:** Galcanezumab, a humanised monoclonal antibody that selectively binds to the calcitonin gene-related peptide, was superior to placebo in the prevention of episodic and chronic migraine in three phase 3 studies. Medication overuse is common among migraine patients. This study investigated medication overuse in a post-hoc analysis of phase 3 placebo-controlled studies of galcanezumab.

**Methods:** This post-hoc analysis comprised EVOLVE-1 and -2 (pooled), and REGAIN, which were phase 3, double-blind, randomised, placebo-controlled studies in patients with episodic migraine (4 to 14 monthly migraine headache days [MHDs]; EVOLVE-1 and -2) and chronic migraine (≥15 monthly MHDs per month for >3 months; REGAIN). Patients in each study were randomised 2:1:1 to monthly subcutaneous injections of placebo or galcanezumab 120 or 240 mg/month for 3-6 months. Headache medication overuse was collected in the electronic patient-reported outcome diary and determined using criteria adapted from the International Classification of Headache Disorders third edition. Mean changes in MHDs and the proportion of patients with medication overuse after randomisation were estimated via mixed modelling.

**Results:** The number of patients with baseline medication overuse in the placebo, galcanezumab 120-mg or galcanezumab 240-mg groups, respectively, was: 298 (39.5%), 140 (18.8%), and 139 (19.5%) for EVOLVE-1 and -2, and 353 (79.5%), 178 (66.0%), and 177 (62.0%) for REGAIN. Both galcanezumab doses demonstrated a statistically significant improvement compared with placebo (p<0.001) for overall least square mean change in monthly MHD in patients with baseline medication overuse (EVOLVE-1 and -2: placebo = -3.07; galcanezumab 120 mg = -5.89; galcanezumab 240 mg = -5.42; REGAIN: placebo = -2.46; galcanezumab 120 mg = -5.29; galcanezumab 240 mg = -4.74). Patients with baseline medication overuse had significantly lower average monthly medication overuse rates for both galcanezumab doses relative to placebo (p<0.001) in all three studies (EVOLVE-1 and -2: placebo = 39.5%; galcanezumab 120 mg = 18.8%; galcanezumab 240 mg = 19.5%; REGAIN: placebo = 79.5%; galcanezumab 120 mg = 66.0%; galcanezumab 240 mg = 62.0%). These findings were consistent with those in patients without baseline medication overuse, as well as in the overall intent-to-treat population in all three studies.

**Conclusions:** Both doses of galcanezumab significantly improved mean monthly MHDs compared with placebo in patients with baseline medication overuse. Average monthly medication overuse decreased with galcanezumab compared with placebo in patients with baseline medication overuse. Galcanezumab is at least as efficacious in patients who overuse acute medications as in those who do not.

**Trial registration:** ClinicalTrials.gov Identifiers: NCT02614183, NCT02614196, NCT02614261

**Ethics approval**

This study was conducted in accordance with the International Conference on Harmonization Guidelines for Good Clinical Practice, the Declaration of Helsinki, and with approval by each institution’s ethical review board. Patients provided written informed consent.

### O5 Study CGAL: a placebo-controlled study of galcanezumab in patients with episodic cluster headache: results from the 8-week double-blind treatment phase

#### James M. Martinez^1^, Peter J. Goadsby^2,3^, David Dodick^4^, Jennifer N. Bardos^1^, Tina M. Myers Oakes^1^, Brian A. Millen^1^, Chunmei Zhou^1^, Sherie A. Dowsett^1^, Sheena K. Aurora^1^, Jyun Yan Yang^1^, Robert R. Conley^1,5^

##### ^1^Eli Lilly and Company, Indianapolis, IN, USA; ^2^University of California, San Francisco, CA, USA; ^3^NIHR-Wellcome Trust King’s Clinical Research Facility, King’s College London, London, UK; ^4^Mayo Clinic, Phoenix, AZ, USA; ^5^University of Maryland School of Medicine, Baltimore, MD, USA

###### **Correpondence:** Sherie A. Dowsett (dowsett_sherie_a@lilly.com)

**Objective:** We assessed the efficacy and safety of galcanezumab, a humanized monoclonal antibody that selectively binds to calcitonin gene-related peptide (CGRP), in individuals with episodic cluster headache.

**Methods:** This study comprised a screening period; a prospective baseline period; an 8-week, double-blind, placebo-controlled treatment period; and a washout period. We present findings from the double-blind treatment period. Participants were randomized 1:1 to galcanezumab 300 mg (N=49) or placebo (N=57) subcutaneously (SC) once monthly. The primary endpoint was overall mean change from baseline in weekly cluster headache attack frequency across Weeks 1- 3. The key (gated) secondary endpoint was the proportion of participants achieving a reduction from baseline of ≥50% in weekly cluster headache attack frequency at Week 3.

**Results:** The mean change in weekly cluster headache attack frequency across Weeks 1-3 was -8.7 for galcanezumab versus -5.2 for placebo (treatment groups difference in mean change, -3.5 [95% CI -6.7, -0.2]; p=0.036). The percentage of participants achieving ≥50% reduction in weekly cluster headache attack frequency at Week 3 was 76% for galcanezumab versus 57% for placebo (p=0.04). Four participants (8%) in the galcanezumab group discontinued during the double-blind period versus 12 (21%) in placebo. In the placebo group, 8 (14%) discontinued due to lack of efficacy versus 1 (2%) with galcanezumab (p=0.036). There were no clinically meaningful differences between treatment groups on tolerability or safety parameters except for a greater incidence of injection site pain with galcanezumab versus placebo (8.2% vs 0%, p=0.043).

**Conclusion:** In individuals with episodic cluster headache, galcanezumab reduced the weekly cluster headache attack frequency across Weeks 1-3 and resulted in a greater percentage achieving a ≥50% reduction in the weekly cluster headache attack frequency at Week 3. The safety profile of galcanezumab in this population was similar to that seen previously in patients with episodic or chronic migraine.

### O6 Migraine with prolonged aura: a rare and peculiar condition? Results form a prospective diary-aided study

#### Michele Viana^1^, Grazia Sances G^1^, Mattias Linde^2^, Giuseppe Nappi^1^, Peter Goadsby^3^ and Cristina Tassorelli^1,4^.

##### ^1^Headache Science Center, C. Mondino National Neurological Institute, Pavia, Italy; ^2^Department of Neuroscience, Norwegian University of Science and Technology, Trondheim, Norway; ^3^Headache Group – NIHR-Wellcome Trust Clinical Research Facility, King’s College London, London, UK; ^4^Department of Brain and Behavioral Sciences, University of Pavia

###### **Correspondence:** Michele Viana (michele.viana@ymail.com)

**Introduction:** Migraine with prolonged aura (PA - defined as an aura including at least one symptom for >1hr) it is considered to be a rare phenomena. Indeed, while the first version of the ICHD included migraine with PA, the subsequent two versions of the ICHD removed PA from the classification. Those auras with at least one symptom lasting more than 60 minutes and less than 7 days are classified as “probable migraine with aura (PA)”. The term “probable” used in such classification indicates suspicion as to whether the symptom is migraine aura and we feel it does not help to categorise auras of a longer duration. Moreover there is limited literature on PA with no prospective studies. The aim of this study is to characterize prospectively the phenotype and prevalence of PA.

**Results:** Two hundred and twenty-four patients suffering from migraine with aura were recruited from the Headache Centers of Pavia and Trondheim. Patients prospectively described, on an *ad hoc* diary, each aura symptom (AS), the duration of AS and headache, and headache features. Seventy-two patients recorded three consecutive auras in their diaries. 19 (26%) of patients suffered at least one PA. Out of 216 recorded auras, 38 (17%) were PAs. We compared PAs with non-PAs with respect to 20 features (Table 1); PAs were characterized by a higher number of non-visual symptoms (non-VS) (p<0.001). No other differences were found. We obtained similar results when we compared auras with at least one symptom with a duration of >2 hrs (n=23) or >4 hrs (n=14) with the the others (n=193 and n=202 respectively). Five per cent of aura symptoms were longer than four hours.

**Conclusion**: PAs are quite common representing 17% of all auras and occurring in 26% of patients. PAs phenotypically are similar to other auras except for a higher number of non-visual symptoms (non-VSs). This latter finding is not surprising if we consider that an AS with a longer duration is likely related to a cortical spreading depression (CSD) that proceeds along a longer path on the respective brain area. Such CSD therefore will involve more easily other adjacent brain areas, conferring a higher number of non-VSs to PA. The substantial phenotypical similarities between PA and the other auras is maintained also when we increase the limit of duration to 2 and/or 4 hrs.

**Table 1 (abstract O6).** Characteristics of PA (auras with at least 1 symptoms lasting >1 h) and NON-PA

Variable	PROLONGED AURAS (PA)	NON-PA	Sig.
*Number*	38	178	
Visual symptoms (VS)	37 (97)	175 (98)	0.69
• DVP	19 (50)	77 (43)	0.44
• Positive	23 (60)	119 (67)	0.45
• Negative	12 (31)	70 (39)	0.37
• Number of elementary visual disturbance^ per aura	1.97 (1.05)	1.94 (0.93)	0.83
Sensory symptoms (SS)	26 (68)	49 (27)	**<0.0001**
Dysphasic symptoms (DS)	12 (31)	10 (7)	**<0.0001**
Number of symptoms (VS, SS, DS)/aura			**<0.0001**
• 1	10 (26)	127 (71)	
• 2	19 (50)	46 (26)	
• 3	9 (23)	5 (3)	
Time relation between aura symptoms in one aura			0.28
• B* starts simultaneously with A*	11 (42)	14 (27)	
• B starts during A	11 (42)	18 (37)	
• B starts when A ceased	2 (8)	5 (10)	
• B starts after an interval of time after A has ceased	2 (8)	12 (24)	
Headache	38 (100)	159 (83)	0.10
• Started before/together with Aura (n=157)	7 (22)	27 (21)	0.88
• Intensity (n=192)	2.3 (0.8)	2.2 (0.8)	0.59
• Unilateral pain (n=192)	27 (75)	100 (64)	0.21
• Throbbing pain (n=187)	17 (47)	71 (47)	0.98
• Pain aggravated by physical activity (n=189)	27 (73)	98 (65)	0.32
• Associated symptom: nausea (n=192)	23 (62)	101 (65)	0.73
• Associated symptom: vomiting (n=189)	8 (23)	25 (16)	0.35
• Associated symptom: photophobia (n=192)	29 (78)	126 (81)	0.68
• Associated symptom: phonophobia (n=191)	23 (62)	99 (64)	0.81
• Associated symptom: osmophobia (n=189)	6 (43)	53 (34)	0.31

Data are presented as means (SD) for continuous data and as n (% of column) for categorical data. DVP: disturbances of visual perception (i.e. blurred/foggy vision, ‘like looking through heat waves or water’, deformed images,…). . **^**In the analysis, the authors dissected every visual into elementary disturbance as reported by Viana et al. 2017 (*Cephalalgia* 37: 979–989). *A and B can be referred to 1st and 2nd aura symptoms or 2nd and 3rd aura symptoms (67 auras had at least 2 symptoms, 14 auras had 3 symptoms). When two symptoms started simultaneously, we designated the first completing symptom as A

### O7 Ten factors associated to migraine chronification: a cross-sectional study on 318 long-term migraine sufferers

#### Michele Viana^1^, Sara Bottiroli^1^, Grazia Sances^1^, Giuseppe Nappi^1^, Cristina Tassorelli^1,2^

##### ^1^Headache Science Center, National Neurological Institute C. Mondino; ^2^Department of Brain and Behavioural Sciences, University of Pavia, Pavia, Italy

###### **Correspondence:** Michele Viana (michele.viana@ymail.com)

**Introduction**: Factors implicated in the evolution of episodic migraine (EM) into chronic migraine (CM) are mostly elusive. Medication overuse (MO) is considered to be one of the main determinants, but other possible clinical and psychological factors can play a role. The aim of this study was to identify some of these factors.

Method: We enrolled consecutive migraine patients in two groups: long history of episodic migraine (EM) and CM with medication overuse (CM-MO) and compared their clinical (n=49) and psychological variables (n=8) in a cross-sectional study. The study was approved by the Local Ethic Committee (on 5^th^ March 2014) and informed consent was obtained from all the patients.

**Results:** Three hundred and eighteen patients were enrolled, of which 156 were EM and 162 were CM-MO patients. The mean age was 42.1±10.3, 80.8% were female. The duration of migraine (before CM-MO onset in the CM-MO group) was 24.6 years in EM and 24.0 years in CM-MO (p=0.57). After the multivariate analysis (Table 1), the factors associated to CM-MO were: age of onset of migraine (earlier), use of at least one migraine preventive medication, marital status (married or separated/divorced/widowed marital status versus unmarried), physical inactivity, history of depression, insomnia associated to use of hypnotics (versus absence of insomnia), previous use of migraine preventive medications, traumatic head injuries, snoring, use of combined oral contraceptives and childhood traumas.

**Conclusion:** We considered several aspects that may be involved in the development of CM-MO. A multivariate analysis identified ten factors belonging to five different areas (migraine history, socio-demographic/lifestyle habits, medical history, gynaecological and psychological factors), meaning that CM-MO onset is likely influenced by a complex mixture of factors. This information is useful when planning strategies to prevent and manage CM-MO.

**Funding**

This work was supported by grants of the Italian Ministry of Health to RC 2013-2015.

**Table 1 (abstract O7).** Second multivariate logistic regression equations performed for predicting the tendency to be a CM-MO versus EM patient

Variable	Sign	OR 95% CI
SOCIO-DEMOGRAPHIC and LIFESTYLE VARIABLES		
Marital Status		
- Single		Ref
- Married	**0.002**	3.65 (1.63-8.19)
- Separated/divorced/Widowed	**0.031**	4.19 (1.13-15.47)
Physical Activity	**0.029**	0.42 (0.19-0.91)
MIGRAINE CHARACTERISTICS		
Age of onset	**0.016**	0.94 (0.89-0.98)
Use of at least one migraine preventive medication (whenever for EM, before chronification + MO onset for CM-MO)	**0.014**	2.36 (1.18-4.71)
OBSTETRICIAN AND GYNECOLOGICAL HISTORY		
Combined Oral Contraceptives (previous/current use vs absent use)	**0.031**	3.38 (1.10-10.3)
FAMILY HYSTORY		
Family history for headache	0.082	2.63 (0.88-7.86)
MEDICAL HYSTORY		
Depression	**0.012**	2.91 (1.25-6.73)
Traumatic head injuries	**0.002**	3.54 (1.57-7.99)
Insomnia		
- no		Ref
- only insomnia	0.94	1.02 (0.47-2.20)
- insomnia + use of hypnotic drugs	**0.006**	5.59 (1.65-18.93)
Snoring	**0.036**	2.24 (1.05-4.79)
PSYCHOLOGICAL VARIABLES		
Childhood Trauma Questionnaire (total score)	**0.012**	1.48 (1.09-2.02)

### **O8 Long-term effects of the non-paralytic botulinum toxin A molecule BiTox in migraine animal models**

#### Ramla AbuukarAbdullahi^1,2^, Joseph O. Lloyd^1^, Martyn G Jones^1^, Giorgio Lambru^2^, Adnan Al-Kaisy^3^, Bazbek Davletov^4^, Anna P. Andreou^1,2^

##### ^1^Headache Research, Wolfson CARD, King's College London; ^2^Headache Centre, Guy's and St Thomas' NHS Trust; ^3^Pain Management and Neuromodulation Centre, Guy's and St Thomas' NHS Trust; ^4^Department of Biomedical Science, University of Sheffield

###### **Correspondence:** Anna P. Andreou (anna.andreou@headache-research.com)

***Background***

*Although botulinum* toxin A is an established preventive treatment for migraine, its toxicity and the unwanted muscle paralysis are major limitations of the achieved efficacy. Recombinant botulinum Clostridial chimeras that lack this paralytic effect, have been recently developed. Of them, BiTox preserves its inhibitory actions on neurotransmitter release from sensory neurons, while it lacks muscle paralytic effects. In this project we aimed to investigate the long-term actions of BiTox in the trigeminal ganglia and trigeminocervical system (TCC) in migraine animal models.

**Methods**

In male rats, Bitox (200 ng) or saline were injected over the peri-orbital areas (100 nl). Seven days later, mechanical (von Frey) and electrical trigeminovascular activation thresholds were assessed bilaterally on first order neurons in the trigeminal ganglia by means of extracellular electrophysiology, by a researcher blinded to experimental groups. In a separate set of experiments, seven days following injections, the superior sagittal sinus was electrically stimulated and TCC tissue was collected and processed for the presence of Fos -positive cells, using a standard immunohistichemistry protocol. Cell counting was performed by a researcher blinded to experimental groups.

**Results**

In first order neurons in the trigeminal ganglia, BiTox significantly increased the mechanical thresholds of Aδ-fibers compared to saline (*P* < 0. 005). Electrical activation thresholds, assessed as the minimum voltage required to induce evoked action potentials, were significantly increased in the BiTox treated group compared to saline in both Aδ- and C-fibers (*P* < 0. 005). The number of Fos-positive cells was significantly lower in the TCC tissue collected from animals treated with BiTox, compared to the saline treated group (*P* < 0. 05).

**Conclusion**

Non-paralytic botulinum-like molecules can be important modulators of trigeminovascular nociceptive processing, offering a promising and significant advancement in the preventive therapeutic options for migraine patients.

**Ethics Approval**

All experiments were conducted under UK Home Office project licence in accordance with the Animal (Scientific Procedures) Act (1986) and conformed to the ARRIVE guidelines. All researchers involved in the conduction of experiments were personal licence holders and approval was granted by the King’s College London Animal Welfare and Ethical Review Board.

### O9 White matter microstructure changes in migraine: a diffusional kurtosis imaging study

#### Sait Ashina^1^, Bettina Conti^2^, Benjamin Ades-Aron^2^, Yvonne Lui^2^, Mia Minen^3^, Dmitry Novikov^2^, Timothy Shepherd^2^, and Els Fieremans^2^

##### ^1^Departements of Neurology, Anesthesia, Critical Care and Pain Medicine, Harvard Medical School, Beth Israel Deaconess Medical Center, Boston, MA, USA; ^2^Department of Radiology, New York University School of Medicine, NYU Langone Medical Center, New York, NY, USA; ^3^Department of Neurology, New York University School of Medicine, NYU Langone Medical Center, New York, NY, USA

###### **Correspondence:** Sait Ashina (sashina@bidmc.harvard.edu)

**OBJECTIVES**: Migraine can be associated with increased risk for stroke and white matter (WM) abnormalities, though the linking mechanisms remain unknown. Diffusion MRI is a powerful method to study microstructural changes in WM non-invasively. Diffusion tensor imaging (DTI) studies have revealed conflicting whether WM integrity is altered in migraine patients. We investigated the normal-appearing WM in migraineurs using diffusional kurtosis imaging (DKI), a clinically-feasible extension of DTI, which also examines the non-Gaussian diffusion effects of water known to occur in the brain.

**METHODS**: The local institutional review board approved a retrospective analysis of 3-T MRI diffusion data (1.7x1.7mm in-plane resolution, 3.0mm slice-thickness,50 slices, b = 0, 250, 1000, & 2000 s/mm^2^ along 84 diffusion directions, TR/TE = 3500/95ms) of 49 migraine patients (age range 21-65, mean 40.65 +/- 12.77 years; 8 male) and 37 non-migraine controls (age range 18-64, mean 41.16 +/- 13.11 years; 11 male). Migraine diagnosis was assigned using ICHD-3 criteria. Patients were also divided in 2 groups: episodic migraine (EM) and chronic migraine (CM). Diffusion data was processed to generate parameter maps of standard DTI metrics (mean, radial, axial diffusivity and fractional anisotropy), as well as mean, radial and axial kurtosis (MK, RK & AK). Using Tract-Based Spatial Statistics (TBSS), skeletonized voxel-wise analysis was performed to identify areas of differences between the groups for the diffusion metrics using FSL's 'randomise' feature while co-varying for age and gender.

**RESULTS**: Of the 49 migraine patients, 20 reported chronic migraine (CM) (age range 21-58, mean 40.05 +/- 11.84 years; 4 men), and 29 reported episodic migraines (EM) (age range 22-65, mean 41.07 +/- 13.60 years; 4 men). Sixteen patients, with age ranging from 22 to 65 (mean 36.87 +/- 13.32; 3 men), reported migraine with aura. TBSS analysis revealed significantly (*p* < 0.05) decreased RK and increased AK in migraineurs compared to controls. Secondary TBSS analysis also found significantly increased AK, and decreased RK in EM compared to controls, while we found no differences between CM and controls, or between EM and CM. TBSS also found increased AK and MK between migraineurs with aura (MA) and without aura (MO), while RK was decreased in both MA and MO compared to controls and MA. No changes were found in any DTI metric.

**CONCLUSION**: This cross-sectional study found DKI parameters being more sensitive than DTI parameters in detecting WM alterations in migraineurs compared to controls. Changes were found in the genu corpus callosum, corona radiata and internal capsule, regions previously (with DTI) shown to be affected in migraine. Remarkably, the observed increase in AK in migraineurs versus controls is the same directional change (increase), though to a smaller extent, as seen in acute stroke infarcts compared to normal appearing white matter, and potentially could be explained here by changes in the intra-axonal environment. The decrease in RK, on the other hand, may suggest myelin breakdown or atrophy. A prospective longitudinal study to explore the role of DKI in predicting migraine course, disease progression and development of comorbidities is warranted.

### O10 Meningeal contribution to migraine pain: a 3T magnetic resonance angiography study

#### Sabrina Khan^1*^, Faisal Mohammad Amin^1^, Casper Emil Christensen^1^, Hashmat Ghanizada^1^, Samaira Younis^1^, Anne Christine Rye Andersen^1^, Patrick J. H. de Koning^2^, Henrik B. W. Larsson^3^, Messoud Ashina^1^

##### ^1^Danish Headache Center and Department of Neurology, Rigshospitalet Glostrup, Faculty of Health and Medical Sciences, University of Copenhagen, Denmark; ^2^Division of Image Processing, Department of Radiology, Leiden University Medical Center, Leiden, Netherlands; ^3^Functional Imaging Unit, Department of Clinical Physiology, Nuclear Medicine and PET, Rigshospitalet, Faculty of Health and Medical Sciences, University of Copenhagen, Denmark

###### **Correspondence:** Sabrina Khan (sksabrinakhan@gmail.com)

The origin of migraine pain is unknown but possibly implicates the dura mater, which is pain sensitive in proximity to the meningeal arteries. Therefore, subtle changes in vessel caliber on the head pain side could reflect activation of dural perivascular nociceptors that leads to migraine headache. To test this hypothesis, we measured circumference changes of cranial arteries in patients with cilostazol-induced unilateral migraine without aura using 3T high-resolution magnetic resonance angiography (MRA). The middle meningeal artery (MMA) was of key interest, as it is the main supply of the dura mater. We also measured the superficial temporal (STA) and external carotid (ECA) arteries as additional extracranial segments, and the middle cerebral (MCA), the cerebral and cavernous parts of the internal carotid (ICA_cerebral_ and ICA_cavernous_), and the basilar (BA) arteries as intracranial arterial segments. MRA scans were performed at baseline, migraine onset, after sumatriptan, and ≥27 hours after migraine onset.A total of 30 patients underwent MRA scans, of which 26 patients developed unilateral attacks of migraine without aura and were included in the final analysis. Eleven patients treated their migraine with sumatriptan while the remaining 15 patients did not treat their attacks with analgesics or triptans.

At migraine onset, only MMA exhibited greater circumference increase on the pain side (0.24 ± 0.37 mm) compared to the non-pain side (0.06 ± 0.38 mm) (p=0.002). None of the remaining arteries revealed any pain-side specific changes in circumference (p>0.05), but exhibited bilateral dilation. Sumatriptan constricted all extra-cerebral arteries (p<0.05). In the late phase of migraine, we found sustained bilateral dilation of MMA.

In conclusion, onset of migraine is associated with increase in MMA circumference specific to the head pain side. Our findings suggest that vasodilation of MMA may be a surrogate marker for activation of dural perivascular nociceptors, indicating a meningeal site of migraine headache.

### O11 Migraine induction with calcitonin gene-related peptide in patients from erenumab trials

#### Casper Emil Christensen^&^; Samaira Younis^&^; Marie Deen; Sabrina Khan; Hashmat Ghanizada; Messoud Ashina

##### ^1^Danish Headache Center and Department of Neurology, Rigshospitalet Glostrup, Faculty of Health and Medical Sciences, University of Copenhagen, Copenhagen, Denmark

###### **Correspondence:** Messoud Ashina (ashina@dadlnet.dk)

^**&**^ These authors contributed equally to this work.

**Background:** Erenumab has recently been approved by the US Food and Drug Administration as a monoclonal antibody against the calcitonin gene-related peptide (CGRP) receptor for migraine-specific preventive treatment. Identifying those patients with the greatest potential to benefit from erenumab treatment could have a major impact on clinical practice. CGRP provokes migraine attacks and the question is whether hypersensitivity to CGRP infusion might be a predictor of erenumab efficacy, serving as a biomarker of treatment efficiency.

**Objective:** To explore a possible correlation between individual efficacy of anti-CGRP treatment and susceptibility to migraine induction by CGRP.

**Methods:** Thirteen migraine patients, previously enrolled in erenumab anti-CGRP receptor monoclonal antibody trials, received CGRP in a double-blind, placebo-controlled, randomized design to investigate their susceptibility to migraine induction. A standardized questionnaire was used to assess efficacy of antibody treatment. The patients were stratified into groups of high responders and poor responders.

**Results:** Ten high responders and three poor responders were included. CGRP induced migraine-like attacks in ten (77%) patients, whereof two were poor responders, compared to none after placebo (p=0.002). The area under the curve for headache intensity was greater after CGRP, compared to placebo, at 0–90 min (p=0.009), and 2–12 h (p=0.014). The median peak headache intensity score was 5 (5–9) after CGRP, compared to 2 (0–4) after placebo (p=0.004).

**Conclusions:** Patients with an excellent effect of erenumab are highly susceptible to CGRP provocation. A large-scale prospective CGRP provocation study in patients should confirm whether hypersensitivity to CGRP could be a biomarker for predicting antibody treatment efficacy.

**Trials Registration number:** ClinicalTrials.gov identifier: NCT03481400.

**Ethics approval and consent**

The study was approved by the Ethics Committee of the Capital Region of Denmark (H-16014580) and is registered at ClinicalTrials.gov (NCT03481400). All participants provided written informed consent to participate in accordance with the Declaration of Helsinki of 1964, with later revisions.

### O12 Incidence, clinical characteristics and long-term course of headache in patients with stroke (DMKG multicenter study)

#### Thomas Dresler^1,2^, Sarah Dietrich^3^, Andrea Düring^4^, Daniel Rothkirch^5^, Filipp Filippopulos^6^, Ozan Eren^6^, Andreas Straube^6^, Stephan Zierz^3^, Gudrun Goßrau^4^, Torsten Kraya^3^

##### ^1^Department of Psychiatry and Psychotherapy, University of Tuebingen, Tuebingen, Germany; ^2^LEAD Graduate School & Research Network, University of Tuebingen, Tübingen, Germany; ^3^Department of Neurology, Martin-Luther-University Halle-Wittenberg, Halle/ Saale, Germany; ^4^University Pain Center, University of Dresden (coop.: Elblandklinikum, Meißen), Germany; ^5^Department of Neurology, BG Clinic Bergmannstrost, Halle/ Saale, Germany; ^6^Department of Neurology, Ludwig-Maximilians-University Munich, Munich-Grosshadern, Germany

###### **Correspondence:** Thomas Dresler (thomas.dresler@med.uni-tuebingen.de)

**Background**:

Post stroke headache (symptomatic headache, ICHD 6.1-6.2.) has not been investigated in Germany. According to previous European and American studies it is a common accompanying symptom. Nevertheless, other stroke symptoms (e.g., palsy, aphasia) are dominating, clinical assessments and treatments focus on acute therapy. However, headache is an essential symptom in subarachnoid bleeding or cerebral venous thrombosis and it is unclear which risk factors modulate symptoms and occurrence of headache in stroke [1,2]. Therefore, we planned a prospective multicenter register study to record the history of the patient, the characteristic of headache symptoms and long-term prognosis, as well as stroke characteristics.

**Methods**:

Patients were included within 24 hours after onset of stroke symptoms and interviewed for headache from day 1 to 3 with a newly generated questionnaire according to ICHD-3 beta [3]. Follow-up data were collected after months 3, 6 and 12. The study was approved by the study centers’ ethics committees and all patients gave their informed consent.

**Results**:

707 stroke patients were included. Diagnoses were: ischemic stroke (67%), TIA (transient ischemic attack, 22%), hemorrhagic stroke (5%), SAH (subarachnoid hemorrhage, 0.8%), cerebral venous thrombosis (0.5%) other diagnoses than stroke (4.7%).

40% complained about headache on at least one of the first three days (46% in females, 36% in males, p<0.02). Headache affected 38% of patients with ischemic stroke and 47% with TIA. The most common stroke affected vessel was the middle cerebral artery (62%). Headache frequency decreased from day 1 to 3. Headache intensity was low in 19% (NRS 1-2), moderate in 67% (NRS 3-6), and high in 14% (NRS 7-10). Headache was most often frontal (70%), followed by occipital (42%). 72% of the patients with previous headache complained about headache on day 1 to 3. Average follow-up response rates were about 55%.

**Discussion**:

Headache prevalence was higher than previously reported (25-35%, [4,5]). One explanation could be that the patients were consequently asked on the stroke unit. Headache prevalence was still that high, when excluding strokes associated with headache (hemorrhagic stroke, cerebral venous thrombosis, SAH). Risk factors are being female and having a history of primary headaches. We conclude that headache is a frequent, yet often unrecognized symptom in stroke which needs specific attention.

**References**:

1. Bederson JB, Connolly ES Jr, Batjer HH, Dacey RG, Dion JE, Diringer MN, Duldner JE Jr, Harbaugh RE, Patel AB, Rosenwasser RH; American Heart Association Guidelines for the management of aneurysmal subarachnoid hemorrhage: a statement for healthcare professionals from a special writing group of the Stroke Council, American Heart Association. Stroke. 2009; 40(3):994-1025.

2. Carolei A, Sacco S. Headache attributed to stroke, TIA, intracerebral haemorrhage, or vascular malformation. Handb Clin Neurol. 2010; 97:517–528.

3. Headache Classification Committee of the International Headache Society (IHS). The International Classification of Headache Disorders, 3rd edition (beta version). Cephalalgia. 2013, 33(9):629-808.

4. Hansen AP, Marcussen NS, Klit H, Kasch H, Jensen TS, Finnerup NB. Pain following stroke: a prospective study. Eur J Pain. 2012; 16(8):1128–1136.

5. Wasay M, Kojan S, Dai AI, Bobustuc G, Sheikh Z. Headache in Cerebral Venous Thrombosis: incidence, pattern and location in 200 consecutive patients. J Headache Pain. 2010; 11(2):137–139.

### O13 Headache in transient ischemic attacks

#### Elena R. Lebedeva^1,2^, Natalia M. Gurary^3^, Jes Olesen^4^

##### ^1^Department of Neurology and Neurosurgery, the Ural State Medical University, 620000, Yekaterinburg, Russia; ^2^International Headache Center “Europe-Asia”, Yekaterinburg, 620144, Russia,cosomos@k66.ru; ^3^Medical Union “New Hospital”, Yekaterinburg, 620000, Russia; ^4^Danish Headache Center, Department of Neurology, Rigshospitalet-Glostrup, University of Copenhagen, Copenhagen, 2600, Denmark

###### **Correspondence:** Elena R. Lebedeva (elenalebedeva1971@gmail.com)

**Background:** Headache is a common feature in acute cerebrovascular disease but no studies have evaluated the prevalence of specific headache types in patients with transient ischemic attacks (TIA). The purpose of the present study was to analyze all headaches within the last year and the last week before TIA and at the time of TIA.

**Methods:** Eligible patients with TIA (n=120, mean age 56.1, females 55%) had focal brain or retinal ischemia with resolution of symptoms within 24 hours without presence of new infarction on MRI with DWI (n=112) or CT (n=8). All patients were evaluated within one day of admission by a single neurologist. As a control group we used patients (n=192, mean age 58.7, females 64%) admitted with diagnoses “lumbago”, “lumbar spine osteochondrosis” or “gastrointestinal ulcer”.

**Results:** One-year prevalence of migraine without aura was significantly higher in TIA patients than in controls: 20.8% and 7.8% respectively (p=0.002, OR 3.1, 95% CI 1.6-6.2). 22 patients (18.3%) had sentinel or warning headache within the last week before TIA. At the time of TIA a new type of headache was observed in 16 patients (13.3%). No controls had a new type of headache. 12 of these 16 patients had migraine-like headache, 8 patients had tension-type-like headache and one patient thunderclap headache. Posterior circulation TIA was associated with headaches within last week before TIA and at the time of TIA much more frequently than anterior circulation TIA.

**Conclusions:** The one year prevalence of migraine was significantly higher in TIA patients than in controls and so was the prevalence of headache within the last week before TIA and at the time of TIA. A previous headache that worsens and a new type of headache can be a warning of impending TIA.

### O14 OPTIMIZATION OF VAGUS NERVE STIMULATION EFFICACY ON CORTICAL SPREADING DEPRESSION

#### Andreia Lopes de Morais^1,3^, Tsubasa Takizawa^1^, Inge Mulder^1^, Tao Qin, BS^1^, Bruce Simon^4^, Rubem Carlos Araujo Guedes^3^, Cenk Ayata^1,2^

##### ^1^Department of Radiology, Massachusetts General Hospital, Boston, MA, USA; ^2^Department of Neurology, Massachusetts General Hospital, Boston, MA, USA; ^3^Department of Nutrition, UFPE, Recife, Pernambuco, Brazil; ^4^Electrocore LLC, Basking Ridge, NJ, USA

###### **Correnspondence:** Cenk Ayata (cayata@mgh.harvard.edu)

**Introduction**: Vagus nerve stimulation (VNS) acutely suppresses cortical spreading depression (CSD) susceptibility, which is a clinically-relevant animal model to screen for migraine therapies. We aimed to optimize VNS efficacy on CSD using various stimulation protocols.

**Methods**: VNS (1ms pulse of 5kHz sine waves at 25 Hz) was delivered non-invasively (nVNS) using cutaneous bipolar disc electrodes. Stimulus trains were generated using a customized stimulator modified from the current gammaCore nVNS device (electroCore LLC, Basking Ridge, NJ). Systemic physiology was monitored during the stimulation. CSD susceptibility was evaluated 40 min after the last stimulation. *Experiment 1* determined the optimal nVNS protocol among sham (N=8), 1x2-min (N=5), 2x2-min 5 min apart (N=8), 3x2-min 5 min apart (N=9), and 1x6-min (N=8) stimulation protocols. *Experiment 2* evaluated chronic daily nVNS for 4 weeks using the most efficient protocol from *Experiment 1*. Daily femoral nerve stimulation (nFNS) was used as control.

**Results**: Among the studied protocols 2x2-min 5 min apart was the most efficient in suppress CSD susceptibility. This protocol increased electrical threshold by 68% and decreased in 22% KCl- induced CSD frequency. Other protocols yielded weaker efficacy (Fig. 1). Chronic nVNS was responsible for 68% increase in electrical CSD threshold and 35% decrease in KCl-induced CSD frequency when compared to nFNS.

**Discussion**: Our results suggest that chronic nVNS may be more effective than the acute stimulation in suppressing CSD.

**Ethics Approval**

Experiments were carried out in accordance with the Guide for Care and Use of Laboratory Animals (NIH Publication No. 85-23, 1996), and approved by the institutional review board.

**Fig. 1 (abstract O14).**
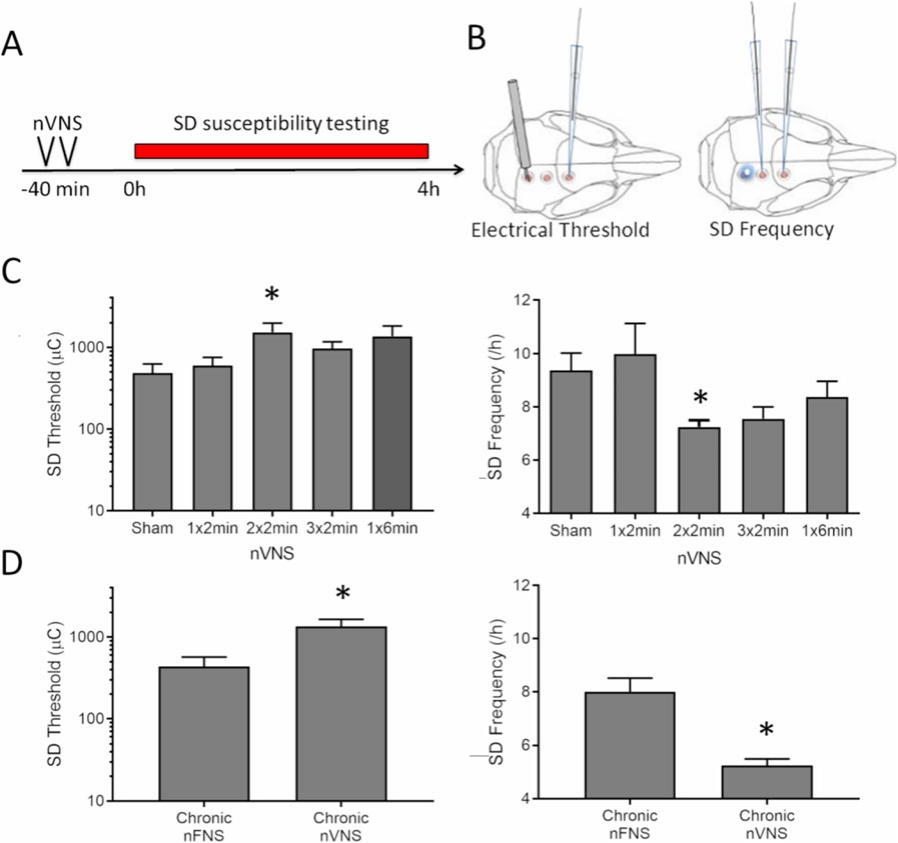
Summary of the data. A) Experimental timeline. B) Electrical Threshold and KCl-induced repetitive SDs assessment. C) Effects of different nVNS protocol on SD susceptibility. D) Effects of 4 weeks daily nVNS on SD susceptibility. Graphs shows Mean ± SE.

### O15 DNA methylation pattern of *CALCA* gene in patients with migraine

#### Elisa Rubino^1^, Silvia Boschi^1,2^, Alessandro Vacca^1^, Salvatore Gentile^3^, Lorenzo Pinessi^3^, Maria Teresa Giordana^1,3^, Innocenzo Rainero^1,3^

##### ^1^Department of Neuroscience “Rita Levi Montalcini”, University of Torino, Italy; ^2^Department of Neuroscience, Psychology, Drug Research and Child Health (NEUROFARBA), University of Florence, Italy; ^3^Department of Neuroscience and Mental Health, AOU Città della Salute e della Scienza di Torino, Italy

###### **Correpondence:** Elisa Rubino (elisa.rubino@unito.it)

**Background.** Migraine is a common neurological disorder characterized by intense, recurrent or chronic pain, representing a cause of extreme disability for the affected subjects. For complex disorders as migraine, both genetic and environmental components play an important role in disease pathogenesis. Epigenetic markers are heritable changes in phenotype or gene expression in the absence of changes in DNA sequence. DNA methylation is the most common type of epigenetic modification, and plays a key role in several disorders as cancer, neurodegenerative disorders, and aging. Changes in global DNA methylation have been associated with environmental factors. To date, little is known about DNA methylation in migraine. Several studies have shown that the neuropeptide calcitonin gene–related peptide (CGRP), encoded by *CALCA* gene, has a key role in migraine pathogenesis, and successful new drugs for treatment of migraine target CGRP. The aim of the study was to screen DNA methylation of *CALCA* gene in patients with migraine.

**Methods.** Twenty-two patients with migraine (F/M 15/7; mean age± SD: 39.7± 13.4 years) and 20 controls (F/M 12/8; mean age± SD: 40.5± 14.8 years) were recruited for the study at the Department of Neuroscience, University of Torino. The diagnosis of migraine was made according to the ICHD-III beta version criteria. Genomic DNA was extracted from peripheral blood. Cytosine-to-thymine conversion by sodium bisulfite and DNA purification were performed using EZ DNA Methylation™ Kit (Zymo Research, Orange, CA, USA). The genomic region around the CpG islands in the promoter region (from -582 to -138 bp) of *CALCA* gene was amplified. Sequencing was performed on an ABI Prism 3130 DNA sequencer.

**Results.** DNA methylation of *CALCA* gene in both patients and controls was more pronounced at 5’ flanking region. No overall difference was found in the global methylation of *CALCA* in patients with migraine and controls. Interestingly, stratification analysis showed that in migraineurs the methylation level was lower in 2 out of 6 analysed CpG islands (CpG -302, p=0.04, and -256, p=0.04, respectively).

**Conclusions.** This study provides the first evidence that DNA methylation of *CALCA* gene promoter could play a role in migraine. Global DNA methylation of *CALCA* gene in migraineurs does not differ from controls, However, DNA methylation status in two CpG islands of the promoter region is lower in patients with migraine. Further studies with larger sample size are needed in order to confirm these preliminary results.

### O16 The impact of chronic headache on workdays, unemployment and disutility in the general population

#### Espen Saxhaug Kristoffersen^1,2^, Knut Stavem^3,4,5^, Christofer Lundqvist^1,3,5,6^, Michael Bjørn Russell^1,3^

##### ^1^Head and Neck Research Group, Research Centre, Akershus University Hospital, Lørenskog; ^2^Department of General Practice, Institute of Health and Society, University of Oslo, Oslo ^3^Institute of Clinical Medicine, Campus Akershus University Hospital, University of Oslo, Nordbyhagen; ^4^Department of Pulmonary Medicine, Medical Division, Akershus University Hospital, Lørenskog ^5^HØKH, Research Centre, Akershus University Hospital, Lørenskog; ^6^Department of Neurology, Akershus University Hospital, Lørenskog, Norway

###### **Correspondence:** Espen Saxhaug Kristoffersen (e.s.kristoffersen@medisin.uio.no)

**Introduction**

Data on the socioeconomic burden of chronic headache (≥15days/last month or ≥180 days/year) is lacking. This study investigated the impact of chronic headache on sickness absence, unemployment and disutility in the general population in Norway.

**Methods**

30,000 persons aged 30─44 from the general population were screened for chronic headache. The International Classification of Headache Disorders was used. We analysed the association of chronic headache with lost workdays, days with >50% reduced productivity, sick leave, unemployment and disutility, as assesses with the SF6D in separate regression analyses.

**Results**

Eighty-three% (427/516) of the eligible participants completed the data on workdays and utility. They reported a mean of 9.7 (SD 24.8) workdays lost over the last 3 months, because of headache. The mean disutility score (1-SF6D score) was 0.41. Thirty-three% were on long term (>1 year) sick leave. The odd ratio (OR) for being on sick leave was 1.9(95% CI 1.1-3.2,p=0.017) for those with secondary compared with primary chronic headache. Similarly, the OR for increased number of workdays lost to headache was 3.5(1.8-6.5,p<0.001) and for unemployment 1.7(1.0-2.9,p=0.07), for those with secondary compared with primary chronic headache. Secondary chronic headache, high headache frequency and high psychological distress were significantly associated with higher disutility score.

**Conclusions**

The burden of chronic headache in the general population is substantial with high rates of lost workdays and disutility.

**Ethics approval and consent to participate**

The Regional Committee for Medical Research Ethics and the Norwegian Social Science Data Services approved the study. All participants gave informed consent.

### O17 Phase 3 studies (EVOLVE-1 & EVOLVE-2) of galcanezumab in episodic migraine: subgroup analyses of efficacy by low- versus high-frequency of migraine headaches

#### Stephen Silberstein^1^, Virginia L Stauffer^2^, Katie Day^2^, Qi Zhang^2^, Sarah Lipsius^3^, Maria-Carmen Wilson^4^

##### ^1^Thomas Jefferson University, Philadelphia, Pennsylvania, 19102, USA; ^2^Lilly Research Laboratories, Lilly Corporate Center, Indianapolis, Indiana, 46285, USA; ^3^Syneos Health, Raleigh, North Carolina, 27604, USA; ^4^Ochsner Health System, Covington, Louisiana, 70433, USA

###### **Correspondence:** Stephen Silberstein; Virginia L Stauffer (stauffer_virginia@lilly.com)

**Objective:** To investigate the efficacy of galcanezumab in episodic migraine (EM) by subgroups of low- versus high-frequency of migraine headaches.

**Methods:** Data were pooled from two phase 3 randomized trials. Headaches were tracked via an electronic patient-reported outcome system, and randomization was stratified by low-frequency (LFEM; 4-7 monthly migraine headache days [MHDs]) or high-frequency (HFEM; 8-14 monthly MHDs). Patients were 18-65 years old and experienced 4-14 MHDs, with ≥2 monthly migraine attacks, determined during the prospective 1-month baseline period. Patients had history of migraine for ≥1 year prior onset, and onset was before age 50. Subgroup analysis of efficacy data, including functional impact and disability measures, were conducted for LFEM and HFEM subgroups with a linear or generalized linear mixed model repeated measures approach.

**Results:** For intent-to-treat patients (N=1773), mean age was 41.3 years, most patients were white (75%), female (85%), and HFEM was present in 66% of patients. There were no statistically significant (p<.05) subgroup-by-treatment interactions for all measures. In both the LFEM and HFEM subgroups, the least square mean (LSM) change differences from baseline in monthly MHDs and monthly MHDs with acute medication use compared with placebo were statistically significantly reduced for galcanezumab 120-mg and 240-mg (Tables 1 and 2). In both LFEM and HFEM subgroups, the mean percentage of patients with ≥50%, ≥75%, and 100% reduction from baseline in overall monthly MHDs during treatment was statistically significantly greater in both galcanezumab dose-groups compared with placebo (p<.001). Galcanezumab treatment statistically significantly improved the Migraine-Specific Quality of Life Questionnaire (**MSQ**) role function-restrictive domain score compared to placebo (p<.001) and the migraine disability assessment (MIDAS) total score compared with placebo (Table 3) for patients in both LFEM and HFEM subgroups.

**Conclusions:** Overall, treatment effects of both doses of galcanezumab were similar with regard to reduction in monthly MHDs, improved functioning, and reduced disability for both LFEM and HFEM subgroups. The percentage of patients with ≥50%, ≥75%, and 100% reduction from baseline in MHDs were similar between LFEM and HFEM subgroups.

**Trial Registration:** NCT02614183, NCT02614196

Ethics approval: These studies were approved by the appropriate institutional review board for each of the study sites. They were all conducted according to Good Clinical Practice and the Declaration of Helsinki guidelines

**Table 1 (abstract O17).** Monthly migraine headache days, average of months 1-6

	LFEM					HFEM					
Treatment	Baseline mean^a^ (SD)	N	LSM change from baseline ± SE	LSM change difference ± SE	p-value^b^	Baseline mean^a^ (SD)	N	LSM change from baseline ± SE	LSM change difference ± SE	p-value^b^	p-value^c^
Placebo	5.8 (1.1)	295	-0.9 ± 0.2			10.9 (2.0)	580	-3.4 ± 0.2			
Galca 120mg	5.8 (1.1)	150	-2.8 ± 0.3	-1.8 ± 0.3	<.001	10.9 (2.0)	286	-5.4 ± 0.3	-2.0 ± 0.3	<.001	.642
Galca 240mg	5.8 (1.2)	145	-2.3 ± 0.3	-1.4 ± 0.3	<.001	10.7 (2.0)	283	-5.5 ± 0.3	-2.1 ± 0.3	<.001	.101

*Galca* galcanezumab, *N* number of subjects who have a non-missing baseline value and at least one post-baseline value;

^**a**^Based upon subjects with a non-missing baseline value; ^**b**^p-value vs. placebo; ^**c**^Treatment-by-subgroup interaction p-value vs. placebo

**Table 2 (abstract O17).** Monthly migraine headache days with acute medication use, average of months 1-6

	LFEM					HFEM					
Treatment	Baseline mean^a^ (SD)	N	LSM change from baseline ± SE	LSM change difference ± SE	p-value^b^	Baseline mean^a^ (SD)	N	LSM change from baseline ± SE	LSM change difference ± SE	p-value^b^	p-value^c^
Placebo	4.8 (1.7)	295	-0.8 ± 0.2			8.9 (3.3)	580	-2.7 ± 0.2			
Galca 120mg	4.8 (1.8)	150	-2.4 ± 0.2	-1.7 ± 0.2	<.001	8.8 (3.4)	286	-4.6 ± 0.2	-1.9 ± 0.2	<.001	.493
Galca 240mg	4.9 (1.7)	145	-2.1 ± 0.2	-1.4 ± 0.2	<.001	8.7 (3.1)	283	-4.6 ± 0.2	-1.9 ± 0.2	<.001	.152

*Galca* galcanezumab, *N* number of subjects who have a non-missing baseline value and at least one post-baseline value**;**

^a^Based upon subjects with a non-missing baseline value; ^**b**^ p-value vs. placebo; ^**c**^Treatment-by-subgroup interaction p-value vs. placebo

**Table 3 (abstract O17).** Migraine disability assessment total score, month 6

	LFEM					HFEM					
Treatment	Baseline mean^a^ (SD)	N	LSM change from baseline ± SE	LSM change difference ± SE	p-value^b^	Baseline mean^a^ (SD)	N	LSM change from baseline ± SE	LSM change difference ± SE	p-value^b^	p-value^c^
Placebo	25.7 (22.6)	241	-9.8 ± 1.4			36.9 (31.5)	483	-15.1 ± 1.2			
Galca 120mg	25.1 (21.8)	126	-18.6 ± 1.8	-8.8 ± 1.9	<.001	35.3 (30.2)	254	-22.3 ± 1.5	-7.2 ± 1.5	<.001	.557
Galca 240mg	28.7 (24.6)	124	-15.9 ± 1.8	-6.2 ± 1.9	.001	37.2 (29.7)	241	-22.1 ± 1.5	-7.0 ± 1.6	<.001	.737

*Galca* galcanezumab, *N* number of subjects who have a non-missing baseline value and at least one post-baseline value**;**

^**a**^Based upon subjects with a non-missing baseline value; ^**b**^p-value vs. placebo; ^**c**^Treatment-by-subgroup interaction p-value vs. placebo

### O18 Persistence of effect of galcanezumab in patients with episodic or chronic migraine: phase 3, randomized, double-blind, placebo-controlled EVOLVE-1, EVOLVE-2 and REGAIN studies

#### Sheena K. Aurora, Qi Zhang^1*^, Virginia L. Stauffer^1^

#### Eli Lilly and Company, Indianapolis, IN, USA

##### **Correspondence:** Qi Zhang (zhang_qi_x1@lilly.com)

**Objectives**: To describe the persistence of effect following treatment with galcanezumab in adult patients with episodic or chronic migraine.

**Methods**: Data from two parallel studies (EVOLVE-1=NCT02614183, EVOLVE-2=NCT02614196) of patients with episodic migraine (between 4 and 14 migraine headache days [MHD] and at least 2 migraine attacks per month during baseline) and one study (REGAIN= NCT02614261) of patients with chronic migraine (headache ≥15 days/month for >3 months, with features of migraine headache ≥8 days/month at baseline) were analyzed. In all three studies, patients randomized in a 1:1:2 ratio received a subcutaneous (SC) injection of galcanezumab at 120 mg/month or 240 mg/month or a SC placebo. Persistence of effect during the double-blind phase was evaluated based on a comparison of the percentages of galcanezumab- and placebo-treated patients with maintenance of ≥50% response (defined as ≥50% reduction from baseline in monthly MHDs) for at least 3 and 6 consecutive months for the episodic study and 3 months for the chronic study. Logistic regression analyses were used for between treatment comparisons. The studies were approved by the appropriate Institutional Review Board for each study site.

**Results**: A total of 1773 adult patients with episodic migraine (n=444 for galcanezumab 120 mg; n=435 for galcanezumab 240 mg; n=894 for placebo for two episodic studies pooled) and 1113 patients with chronic migraine (n=278 for galcanezumab 120 mg; n=277 for galcanezumab 240 mg; n=558 for placebo) were evaluated. In patients with episodic migraine, significantly higher percentages of patients maintained ≥50% response for at least 3 consecutive months in the galcanezumab 120 mg (41.5%; p<.001) and 240 mg (41.1%; p<.001) groups or for 6 consecutive months (19.0% and 20.8%, respectively; p<.001) compared with the placebo group (21.4% at 3 months and 8.0% at 6 months). In patients with chronic migraine, significantly higher percentages of patients in the galcanezumab 120 mg (16.8%) and 240 mg (14.6%) groups maintained ≥50% response for all 3 months of the double-blind treatment phase compared with placebo (6.3%; all p<.001).

**Conclusions**: Treatment with galcanezumab 120 mg or 240 mg demonstrated statistically significant and clinically meaningful persistence of effect in patients with episodic migraine (at least 3 and 6 consecutive months) and in patients with chronic migraine (for 3 months).

### O19 Efficacy of galcanezumab in patients who failed prior preventive treatments for migraine: results from EVOLVE-1, EVOLVE-2 and REGAIN studies

#### Grazia Dell’Agnello^1^, Antje Tockhorn-Heidenreich^2^, Qi Zhang^3^, Dustin D. Ruff^3^, Eric M. Pearlman^3^, Sheena K. Aurora^3^, Janet H. Ford^3^

##### ^1^Eli Lilly SpA, Sesto Fiorentino, Italy; ^2^Eli Lilly and Company Limited, Erl Wood Manor, Windlesham, Surrey, UK; ^3^Eli Lilly and Company, Indianapolis, IN, USA

**Background**

Galcanezumab (GMB) is a humanised monoclonal antibody against calcitonin gene-related peptide under development for prevention of migraine. Objective of this post-hoc analysis of three Phase 3 studies of GMB was to assess if there were any differential treatment effects in patients who had failed ≥2 previous preventives due to all-cause (efficacy and/or safety/tolerability) versus those who had not.

**Methods**

EVOLVE-1 (NCT02614183), EVOLVE-2 (NCT02614196) and REGAIN (NCT02614261) were Phase 3, randomised, double-blind, placebo-controlled studies in patients with episodic (EVOLVE-1/2) or chronic (REGAIN) migraine. Patients were randomised 2:1:1 to receive placebo (PBO), GMB_120mg or GMB_240mg during double-blind treatment period lasting 6 months (EVOLVE-1/2) or 3 months (REGAIN). Post-hoc analyses were conducted for change from baseline in the number of monthly migraine headache days (MHD) and ≥50% response (reduction in the number of MHDs) for patients who failed ≥2 prior preventive treatments or preventive treatment classes. Subgroup-by-treatment interactions were calculated using linear or generalised linear mixed models.

**Results**

In the integrated analysis of EVOLVE studies (PBO=90, GMB_120mg=51, GMB_240mg=45) and in the REGAIN study (PBO=175, GMB_120mg=72, GMB_240mg=104), GMB_120/240mg statistically significantly improved overall mean reduction from baseline of monthly MHD compared with PBO (EVOLVE 1/2 p<0.001; and REGAIN p<0.01). For the subgroup who failed prior preventives, mean monthly reductions from baseline (MHD [Standard Error]) in EVOLVE 1/2 were: PBO: 0.46 (0.64); GMB_120mg: 3.06 (0.74); GMB_240mg: 3.83 (0.80); and in REGAIN were: PBO: 1.01 (0.54); GMB_120mg: 5.35 (0.71); GMB_240mg: 2.77 (0.66). Significant treatment-by-subgroup interactions were seen for GMB_240mg (EVOLVE-1/2) and for GMB_120mg (REGAIN) suggesting better efficacy compared with PBO for these doses in patients who failed prior preventives. Estimated mean proportions (SE) of patients with ≥50% response were significantly higher compared with PBO (EVOLVE-1/2, PBO: 0.225 [0.033]; GMB_120mg: 0.536 [0.050]; GMB_240mg: 0.611 [0.053], p<0.001;), REGAIN, PBO: 0.094 [0.019]; GMB_120mg: 0.295 [0.047]; p<0.001 and GMB_240mg: 0.186 [0.033]; p<0.01). The results are consistent also when considering failure due to all-cause for ≥2 previous treatment classes.

**Conclusions**

GMB_120/240mg is efficacious compared with PBO in reducing monthly MHDs in both in the overall population and the patients who failed ≥2 prior preventive treatments. Treatment-by-subgroup interactions may be driven by lower PBO response in patients who failed prior preventive treatments, as magnitude of change for GMB-treated patients were similar in both subgroups.

**Trial Registration**: Each study was approved by a central Ethics Review Board and registered on ClincalTrials.gov (NCT02614183 (EVOLVE-1); NCT02614196 (EVOLVE-2); NCT02614261 (REGAIN); NCT02614287 (Study CGAJ)).

### O20 Cephalic and extracephalic neurophysiological effects of botulinum toxin type A treatment in chronic migraine

#### Gianluca Coppola^1*^, Francesca Cortese^1^, Davide Di Lenola^1^, Cherubino Di Lorenzo^2^, Vincenzo Parisi^3^, Francesco Pierelli^1,4^.

##### ^1^Sapienza University of Rome Polo Pontino, Department of Medico-Surgical Sciences and Biotechnologies, Latina, Italy; ^2^Don Carlo Gnocchi Onlus Foundation, Milan, Italy; ^3^G.B. Bietti Foundation IRCCS, Research Unit of Neurophysiology of Vision and Neurophthalmology, Rome, Italy; ^4^IRCCS-Neuromed, Pozzilli (IS), Italy

###### **Correspondence:** Gianluca Coppola (gianluca.coppola@gmail.com)

**Background**

Injection botulinum toxin type A (BTX-A) has been approved for the treatment of chronic migraine (CM). Although different studies have shown that this treatment is highly effective and safe, the neurophysiological mechanisms underlying its clinical efficacy are still debated widely. This study assessed the segmental, suprasegmental, cephalic, and extra-cephalic effects of BTX-A injection in a group of patients with CM.

**Materials and methods**

We assessed the excitability of the trigeminal system in a group of 13 CM patients (11 with and 2 without medication overuse), by simultaneously recording the blink reflex (nBR), the trigemino-cervical reflex (nTCR), and the pain-related evoked potential (PREP), following stimulation of the right supraorbital nerve with a nociception specific concentric electrode. Further, we recorded the non-noxious somatosensory evoked potentials (SSEPs) amplitude and habituation to verify the influence of BTX-A prophylaxis on the cortical excitability in extracephalic sensory areas. Neurophysiological measurements were recorded before (T0), and 1 month (T1) and 3 months (T3) after BTX-A injections.

**Results**

At month 3, BTX-A significantly reduced the mean monthly headache days, severity of headache (1-3), and the mean monthly tablet intake (all p=<0.001). A significant increase in pain threshold, but not in perception threshold was noted 3 months after treatment compared to baseline (T0=8.3 mA, T2=12.0 mA; p=0.04). Despite a non-significant variation of the 1^st^ nBR and nTCR amplitude blocks, we found that the initial nBR and nTCR lack of habituation was replaced by normal habituating response at 3 months after treatment (p=0.005 for nBR, p<0.05 for nTCR). There were no variations in the initial PREP and SSEP after BTX-A, despite a trend for an increased habituation for PREP and lack of SSEP amplitudes.

**Conclusions**

This is the first study to show that the clinical improvement induced by a single session of BTX-A injection in CM patients may be attributed to the neurophysiological changes that occur at the brainstem and that BTX-A may have an active in modulating the habituation of subcortical trigemino-cervical circuits. Further, our findings suggest that the responsiveness to a single session of BTX-A may be related to the blockage of sensitization of the nociceptive neurons in the dorsal horn, without an evident involvement of cortical circuitries.

### O21 Idiopathic Intracranial Hypertension: Consensus Guidelines on Management

#### S. P. Mollan^1,2^, B. Davis^3^, N. C. Silver^4^, S. Shaw^5^, C. Malucci^6,7^, B. R. Wakerley^8,9^, A. Krishnan^4^, S. V. Chavda^10^, S. Ramalingam^10^, J. Edwards^11,12^, K. Hemmings^13^, M. Williamson^13^, M. A. Burdon^2^, G. Hassan-smith^1,13^, K. Digre^14^, G. T. Liu^15^, R. H. Jensen^16^, A. J. Sinclair^1,2,13,17^

##### ^1^Neurometabolism, Institute of Metabolism and Systems Research, University of Birmingham, Edgbaston, B15 2TT, UK; ^2^Birmingham Neuro-Ophthalmology, Queen Elizabeth Hospital, Birmingham, B15 2WB, UK; ^3^Department of Neurology, University Hospital North Midlands NHS Trust, Stoke-on-Trent, ST4 6QG; ^4^Department of Neurology, The Walton Centre NHS Foundation Trust, Liverpool, L9 7LJ, UK; ^5^Department of Neurosurgery, University Hospital North Midlands NHS Trust, Royal Stoke University Hospital, Newcastle Road, Stoke-on-Trent, ST4 6QG, UK; ^6^Department of Neurosurgery, The Walton Centre NHS Foundation Trust, Liverpool, L9 7LJ, UK; ^7^Department of Paediatric Neurosurgery, Alder Hey Children's NHS Foundation Trust, Liverpool L12 2AP, UK; ^8^Department of Neurology, Gloucestershire Hospitals NHS Foundation Trust, GL53 7AG, UK; ^9^Nuffield Department of Clinical Neurosciences, Oxford OX3 9DU, UK; ^10^Department of Neuroradiology, University Hospitals Birmingham, Queen Elizabeth Hospital, Birmingham, B15 2WB, UK; ^11^Department of Neurology, Sandwell and West Birmingham NHS Trust, City Hospital, Dudley Road, Birmingham, UK; ^12^Department of Neurology, University Hospitals Birmingham, Queen Elizabeth Hospital, Birmingham, B15 2WB, UK; ^13^IIH-UK, Tyne & Wear, NE38 7JX, UK; ^14^Departments of Ophthalmology and Neurology, Moran Eye Center, University of Utah, Salt Lake City, Utah, USA; ^15^Neuro-ophthalmology Services, Children's Hospital of Philadelphia and Hospital of the University of Pennsylvania, Philadelphia, PA 19104, USA; ^16^Danish Headache Center, Department of Neurology, Rigshospitalet-Glostrup, University of Copenhagen, Denmark; ^17^Centre for Endocrinology, Diabetes and Metabolism, Birmingham Health Partners, B15 2TH, UK

###### **Correspondence:** S. P. Mollan (soozmollan@doctors.org.uk)

**Background**

Idiopathic intracranial Hypertension (IIH) is commonly associated with obesity, younger age and females. Patients present acutely to many different specialities, and often through the course of their disease will have multiple acute visits. Headache is the major morbidity in IIH.

**Objectives**

The aim was to capture interdisciplinary expertise from a large group of clinicians, reflecting practice from across the UK and further, to inform subsequent development of a national consensus guidance for optimal management of Idiopathic Intracranial Hypertension.

**Methods**

Between September 2015 and October 2017 a specialist interest group including neurology, neurosurgery, neuro-radiology, ophthalmology, nursing, primary care doctors, and patient representatives met. A comprehensive systematic literature review was performed to assemble the foundations for the working group. Population, Interventions, Controls and Outcomes (PICO) questions were defined and through a large Delphi group exercise, expertise was captured from a wide-reaching group of clinicians reflecting practice from across the United Kingdom and internationally. The statements were then critically reviewed key opinion leaders and by Association of British Neurologists, British Association for the Study of Headache, the Society of British Neurological Surgeons and the Royal College of Ophthalmologists.

**Results**

Over twenty questions were constructed: One based on the diagnostic principles for optimal investigation of papilloedema and twenty-one for the management of IIH. 3 main principles were identified: 1, to treat the underlying disease; 2, to protect the vision and 3, to minimise the headache morbidity. Statements presented provide insight to uncertainties in IIH where research opportunities exist.

Evaluation of the headache phenotype was found to be essential, so that targeted treatment can be used and help identification of medication overuse headache. Acute exacerbation of headache often leads to re-investigation with lumbar puncture, and the collective expert opinion reflected that lumbar puncture only provides temporary relief, can lead in some to longer term complications and exacerbation of headache. In those with acute exacerbation of headache, optic nerve examination is essential and in those found not to have papilloedema, investigation with LP and brain imaging is not required as long as no other secondary causes of headache are suspected.

**Conclusions**

In collaboration with many different specialists, professions and patient representatives we have developed guidance statements for the investigation and treatment of adult IIH. This is the first consensus guidance for optimal management of IIH.

### O22 Long-term outcomes after reversible cerebral vasoconstriction syndrome: recurrence, cardiovascular events and mortality in a prospective follow-up study

#### Rosalie Boitet^1^, Solène De Gaalon^2^, Grégory Marin^3^, Claire Duflos^3^, Caroline Roos^2^, Jérôme Mawet^2^, Cécilia Burcin^2^, Ursula Fiedler^2^, Marie-Germaine Bousser^4^ et Anne Ducros^1^

##### ^1^Neurology Department & ^3^Statistics, Montpellier University Hospital, Montpellier; ^2^Emergency Headache Centre & ^4^Neurology Department, Lariboisière Hospital, APHP, Paris, France

###### **Correspondence:** Rosalie Boitet (rosalie.boitet@gmail.com)

**Introduction:** Long-term outcomes >5 years after reversible cerebral vasoconstriction syndrome (RCVS) have not been previously studied.

**Objective:** To determine rates of recurrent thunderclap headache, recurrent RCVS, incident stroke, and cardiovascular mortality (CVM) after a first RCVS (RCVS1).

**Methods:** Of 173 RCVS patients recruited from 2004 to 2013 in Lariboisière, 172 completed a follow-up >6 months and were included in this study. Follow-up visits were clinical at 1, 3, 6, 12 and 18 months after RCVS, thereafter by consultations, phone calls, emails or letters with a final contact between March 2017 and February 2018.

**Results:** Of the 172 patients who completed a mean follow-up of 110 ± 40 months (range 6–196), 28 had a new thunderclap-like headache, and 10 (5.8%) had a confirmed recurrent RCVS (0.65 per 100 person-years, 95% CI 3.29 – 11.54). The second RCVS occurred a mean of 78 ± 46 (median 81, interquartile range 35 – 93) months after the first. One woman had a third RCVS (0.6%). The only two significant independent predictors of recurrent RCVS were exertion as a trigger for the RCVS1 (OR = 6, 95% CI 1.5–23.7) and having a newly diagnosed hypertension after RCVS1 (OR = 4.8; 95% CI 1.3–19.2). Of the 7 women who delivered during follow-up, one (14%) had a recurrent RCVS in postpartum. One 46 years-old man (0.6%) had a sudden cardiac death 5 years after RCVS1. One patient had a stroke (0.6%) during her recurrent RCVS.

**Conclusions**: RCVS patients have a low but significant risk of recurrent RCVS and CVM. Exertion as a trigger for RCVS1 and developing hypertension after RCVS1 are potential predictors of recurrent RCVS.

### O23 Complete detoxification is the most effective in reducing disability in patients with medication-overuse headache: A randomized controlled open-label trial

#### Mia Nielsen, Louise N. Carlsen, Rigmor H. Jensen, Signe B. Munksgaard, Ida M. S. Engelstoft, Lars Bendtsen

##### Danish Headache Center, Neurological Clinic, Rigshospitalet, Glostrup, Denmark

###### **Correspondence:** Louise N. Carlsen (louise.ninett.carlsen@regionh.dk)

**Background**: Complete stop of acute medication and/or migraine medication for treatment of medication-overuse headache (MOH) has previously been reported more effective in reducing headache days and migraine days per month compared with restricted intake of acute medication. It is well known that treatment of MOH reduces disability and increases quality of life for patients.

**Aim**: To compare changes in disability and quality of life between two detoxification programs for medication-overuse headache (MOH).

**Methods**: In a prospective, outpatient study patients with MOH were randomized to program A (two months with no acute analgesics or migraine-medications) or program B (two months with acute medications restricted to two days/week). At 6 and 12 months follow-up, disability and headache burden were measured by Headache Under Response to Treatment-8 (HURT-8) and HURT-3, respectively. Quality of life was estimated by EUROHIS QOL 8-item (QOL-8) at 2, 6 and 12 months follow-up.

**Results**: We included 72 MOH patients with a primary migraine and/or tension-type headache diagnosis. Fifty-nine patients completed detoxification. At 12 month-follow-up, 41 completed HURT and 38 patients EUROHIS QOL-8. At month 12, HURT-8 was reduced by 24.5% in program A and 7.1% in program B (p=0.027). HURT-3 in program A was reduced by 33.3% versus 3.1 % in program B (p=0.005). At 12 months, QOL-8 was increased by 8.2% in total, without any significant difference between the programs (p=0.297). However, there was significant difference in favor of program A in QOL-8 at 2 months (p=0.006).

**Conclusion**: Both detoxification programs reduced disability and improved quality of life. Detoxification without acute medication was the most effective in reducing headache burden and disability.

**Trial registration:** Clinicaltrials.gov (NCT02903329).

**Ethical approval**

The study was approved by the Regional Ethical Committee in Denmark (H-1-2012- 105 116)

### O24 The economic and humanistic burden of episodic and chronic migraine in Europe

#### Hicham Benhaddi^1^; Timothy Fitzgerald^2^; Sophie McCabe^3^; Ruth Zeidman^3^

##### ^1^Teva Pharmaceuticals, Wilrijk, Belgium; ^2^Teva Pharmaceuticals, Frazer, Pennsylvania, USA; ^3^Covance Market Access, London, UK

###### **Correspondence:** Hicham Benhaddi (mhowell@hcg-int.com)

**BACKGROUND:**

Migraine is a debilitating neurological disorder characterized by attacks that may last 4–72 hours, with a high burden in Europeans.

**OBJECTIVE:**

This systematic literature review examined the clinical, humanistic, and economic burden associated with chronic and episodic migraine (CM and EM, respectively) in Europe.

**METHODS:**

Literature searches and evidence screening were structured according to the PICOS (population, intervention, comparators, outcomes, and study types) framework. Reviews and original observational studies in adults (≥18 years) with EM (<15 headache days per month) or CM (≥15 headache days with ≥8 migraine days per month) were included. Searches focused on resource utilization, treatment costs, productivity, quality of life (including generic and migraine-specific instruments and functioning), and utility outcomes (published 2007– February 1, 2018; geographical limitation: United Kingdom, France, Germany, Spain, Italy, the Netherlands, Poland, Denmark, Finland, Iceland, Norway and Sweden). Searches included: Embase, MEDLINE, and the Cochrane Library databases; specialty medicine associations; and health technology assessment agency websites.

**RESULTS:**

Analysis included 68 publications. Data from the World Health Organization indicated that in Europe, migraine burden weighed higher than that of epilepsy, multiple sclerosis, and Parkinson’s disease. Up to 57% of individuals with migraine report severe disability, and many find treatments ineffective. Nausea and/or vomiting occurred in up to 74% of individuals with migraine. Depression and/or anxiety occur up to three times more often in individuals with migraine than in the general population. People with migraine have reported poorer health-related quality of life than those without migraine, which worsens with increasing migraine attack frequency. Europeans with migraine perceive that it has a negative impact on work (up to 76%), family situations, leisure time, studies, sexual life, social position, love, financial situation, career, and friendships. The prevalence of migraine is highest for men and women during their peak years of economic productivity (ages 25–55 years). In Europe, the estimated total annual cost was up to €111 billion (2008–2009), of which 72%–98% was indirect costs (two-thirds of indirect costs due to reduced productivity). Annual direct costs for CM were up to four times higher than those for EM.

**CONCLUSIONS:**

This research demonstrates that migraine has a substantial humanistic and economic burden on Europeans and affects all aspects of life.

### O25 Headache outcome measures in medically refractory chronic migraine patients treated with OnabotulinumtoxinA

#### Lagrata Susie^1^, Ahmed Maha^1^, Miller Sarah^1,2^, Matharu Manjit^1,2^

##### ^1^Headache Group, National Hospital for Neurology and Neurosurgery, Queen Square, London; ^2^Institute of Neurology, University College London, National Hospital for Neurology and Neurosurgery, Queen Square, London

###### **Correspondence:** Matharu Manjit (m.matharu@uclmail.net)

**Introduction**

OnabotulinumtoxinA is standard care of management for chronic migraine (CM). Few studies on the use of OnabotulinumtoxinA on CM have identified factors associated with a positive response to OnabotulinumtoxinA treatment. There are currently no data on outcome measures that might predict subjective perceived outcome (SPO) to OnabotulinumtoxinA in reported by patients with medically-refractory chronic migraine (rCM).

**Aim**

To identify components of headache characteristics (frequency, intensity, duration) or disability score (Headache Impact Test-6, HIT-6) that predicts SPO.

**Method**

100 patients who had completed at least two full treatment cycles and had at least six months follow up were identified. Data on SPO, clinical and headache characteristics were collected prospectively on all patients using headache diaries, feedback forms and validated disability scores. Variables analysed for predictors of SPO were headache characteristics (frequency, intensity and duration) and disability score (HIT-6) using multivariate analysis.

**Results**

Response rates are shown in Fig. 1. Multivariate analysis showed change in pain intensity (p<0.001) and pain duration (p=0.022) were significantly positively associated with SPO. Change in headache days (p=0.294) and HIT-6 score (p=0.321) were not significantly positively associated with SPO. Table 1 shows results of multivariate analysis.

**Conclusion**

Our data suggest that improvement in pain intensity and headache duration predicts SPO to onabotulinumtoxinA in rCM. The results of this study suggest that for rCM the use of pain intensity and headache duration appear to be the more appropriate outcome measure to assess. The current NICE guidelines which state that response should be assessed using headache days alone may not be appropriate for rCM.

**Fig. 1 (abstract O25).**
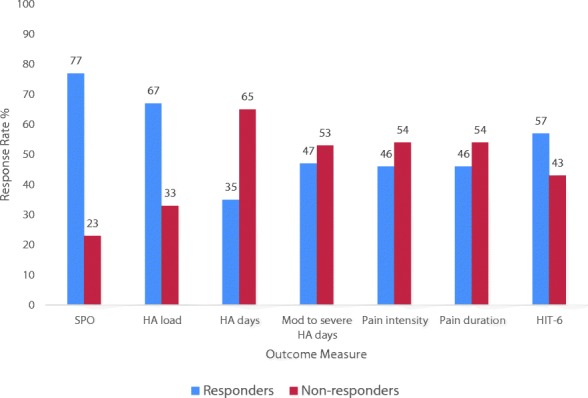
Response rate for each outcome measure*.* Responders ≥ 30% improvement on each outcome measure; ≥3 HIT-6 points improvement. HA: headache, SPO: subjective perceived outcome

**Table 1 (abstract O25).** Results of univariate and multivariate analysis

Variables	Univariable Analysis r^2^	Multivariable Analysis β, Standard error	p=value
Change in headache days	0.292	1.126, 0.120	0.294
Change in pain intensity	0.488	0.539, 0.090	<0.001
Change in pain duration	0.303	0.194, 0.083	0.022
HIT-6 points	0.130	0.216, 0.217	0.321

Dependent Variable: Patient subjective perceived outcome (SPO)

### O26 Searching for a neural signature of hallodynia in migraine: a volumetric MRI study

#### Francesca Pistoia^1^, Claudia Marsecano^2^, Antonio Carolei^1^, Riccardo Cornia^1^, Luana Evangelista^1^, Alessandra Splendiani^2^, Simona Sacco^1^

##### ^1^Neurological Institute, Department of Applied Clinical Science and Biotechnology, University of L’Aquila, L’Aquila, Italy; ^2^Radiology section, Department of Applied Clinical Science and Biotechnology, University of L’Aquila, L’Aquila, Italy

###### **Correspondence:** Francesca Pistoia (francesca.pistoia@univaq.it)

**Background**

Allodynia is a phenomenon of central pain sensitization, characterized by the occurrence of a painful sensation following the administration of innocuous stimuli like a light touch. It occurs frequently in patients with migraine and it seems to be associated with the duration and severity of the disease [1-2]. The aim of the present study was to investigate the presence of structural brain changes in migraine patients with hallodynia as compared to migraine patients without hallodynia.

**Materials and methods**

Consecutive eligible patients referring to our Regional Headache Center within a six-month period and with a diagnosis of migraine were screened for the inclusion in the study. Hallodynia was investigated through the Allodynia Symptom Checklist [3]. All patients underwent a neuroradiological assessment through a 3 Tesla Magnetic resonance Imaging (MRI) scanner (Discovery MR750w). MRI images were analyzed through the Freesurfer 6.0 software to evaluate volume cortical differences between the two groups. Groups’ analyses were performed through the QDec software for cortical volumes (level of significance fixed at p 0.01)

**Results**

Sixteen women with clinical signs of hallodynia (mean age±SD 45.2±9.2) were included and compared to 16 women not showing hallodynia (mean age±SD 44.8±8.8). The two groups were comparable referring to disease duration (19.3±13.9 vs 20.8±9.6, respectively; p>0.05) and number of days with migraine per month (mean±SD 7.0±4.8 vs 8.0±5.8, respectively; p>0.05). Migraine patients with hallodynia showed a selective cortical volume loss involving the pars triangularis (p< 0.001), the rostral anterior cingulate cortex (p< 0.001), the precuneus (p< 0.005) and the rostral middle frontal cortex (p< 0.005) in the left hemisphere, and the precuneus in the right hemisphere (p< 0.005). No correlation was found between the migraine duration and the extent of volumetric decrease in patients with allodynia.

**Conclusions**

We found a selective cortical volume loss associated with the phenomenon of hallodynia in some areas, which are functionally linked to the experience of pain. Specifically, the anterior cingulate cortex has been previously reported to have a role in registering the pain intensity and in modulating the individual emotional reaction to pain [4]. Similarly, the activity of the precuneus may be linked to the perception of the environment, the cue reactivity and the affective responses to pain [5]. Further studies are necessary to establish whether the above areas belong to critical hub networks influencing the development of hallodynia and whether they can become the target of specific pharmacological and behavioral therapies [6].

**References**

1. Levinsky Y, Zeharia A, Eidlitz-Markus T. Cephalic cutaneous allodynia in children and adolescents with migraine of short duration: A retrospective cohort study. Cephalalgia. 2018 Jan 1:333102418776018.
2. Tietjen GE, Brandes JL, Peterlin BL, Eloff A, Dafer RM, Stein MR, Drexler E, Martin VT, Hutchinson S, Aurora SK, Recober A, Herial NA, Utley C, White L, Khuder SA. Allodynia in migraine: association with comorbid pain conditions. Headache 2009;49:1333-44.
3. Lipton RB, Bigal ME, Ashina S, Burstein R, Silberstein S, Reed ML, Serrano D, Stewart WF. Cutaneous allodynia in the migraine population. Ann Neurol 2008;63:148-158.
4. Barthas F, Sellmeijer J, Hugel S, Waltisperger E, Barrot M, Yalcin I. The anterior cingulate cortex is a critical hub for pain-induced depression. Biol Psychiatry 2015;77:236-245.
5. Cavanna AE, Trimble MR. The precuneus: a review of its functional anatomy and behavioural correlates. Brain 2006;129:564-583.
6. Pistoia F, Sacco S, Carolei A. Behavioral therapy for chronic migraine. Curr Pain Headache Rep 2013;17:304.

### O27 Dopaminergic symptoms in migraine: a case series on 1148 patients

#### P. Barbanti^1^, P. Ferroni^2,3^, G. Egeo^1^, L. Fofi^1^, C. Aurilia^1^

##### ^1^Headache and Pain Unit IRCCS San Raffaele Pisana, Rome, Italy; ^2^Department of Human Sciences and Quality of Life Promotion, San Raffaele Roma Open University, Rome, Italy; ^3^Interinstitutional Multidisciplinary Biobank (BioBIM), IRCCS San Raffaele Pisana, Rome, Italy

###### **Correpondence:** P. Barbanti (piero.barbanti@sanraffaele.it)

**Background**: Dopamine (DA) plays a major role in migraine pathogenesis as suggested by clinical, genetic, biochemical and pharmacological evidence. Some migraineurs show intense dopaminergic symptoms (DAs) during the attack and might represent a distinctive endophenotype. The present study is aimed to assess frequency and characteristics of DAs in migraineurs during the different attack phases.

**Methods**: We studied all patients affected by episodic and chronic migraine consecutively seen at our Headache and Pain Unit from 1 April 2017 to 31 March 2018. Following a careful physical and neurological examination, all patients were evaluated with face-to face interviews using a semi-structured questionnaire addressing three main issues: 1) life-style, behavioral and socio-demographic factors; 2) comorbidities and concomitant medications; and 3) clinical migraine features encompassing family history, disease duration, site, quality and intensity of pain, attack duration and frequency, presence, type and duration of aura, prodromes, accompanying symptoms, postdromes, DAs, allodynia, unilateral cranial parasympathetic symptoms, triggers and alleviating factors, previous and current acute or preventive treatments, patients’satisfaction with triptans. The presence of DAs was determined by asking the following question: “During prodromes, headache stage or postdromes do you also have at least one of the following symptoms: yawning, somnolence, fatigue, severe nausea, vomiting, mood changes or diuresis?”.

**Results:** We studied 1148 migraine patients (F/M: 902/246; without aura, MwA: 679; with aura, MA: 66; MwA + MA: 37; chronic migraine, CM: 366; medication overuse headache, MOH: 284). A total of 374 patients (32.6%) reported the presence of ≥1 DAs during the attack: 143 patients with DAs (DAs+) (38.2%) reported 1 DAs, 107 (28.6%) 2 DAs and 124 (33.2%) ≥3 DAs. The most frequent DAs was yawning (64.4%) followed by somnolence (58.6%), severe nausea (42%), vomiting (27.3%), fatigue (17%), mood changes (7%) and diuresis (3%). DAs mostly occurred during headache stage (54%), less frequently during prodromes (33.7%) or postdromes (27%). Migraineurs with and without DAs did not differ for life-style, behavioral and socio-demographic factors and other migraine variables. Stepwise logistic regression analysis revealed that DAs+ patients had more frequently long-lasting attacks (p <0.0001), osmophobia (p<0.0001), allodynia (p=0.0077), and unilateral autonomic symptoms (p=0.0426) than general migraine population.

**Conclusions:** More than 1/3 or migraineurs show DAs, mostly yawing and somnolence, especially during the headache phase. Their attacks are longer and more frequently associated with allodynia, osmophobia and unilateral autonomic symptoms than general migraine population. DAs+ patients could represent a migraine endophenotype.

### O28 Non-invasive vagus nerve stimulation (nVNS) for the acute treatment of episodic migraine: additional findings from the randomised, sham-controlled, double-blind PRESTO trial

#### Paolo Martelletti^1^, Licia Grazzi^2^, Giulia Pierangeli^3^, Innocenzo Rainero^4^, Pierangelo Geppetti^5^, Anna Ambrosini^6^, Paola Sarchielli^7^, Piero Barbanti^8^, Cristina Tassorelli^9,10^, Eric Liebler^11^, Marina de Tommaso^12^

##### ^1^Department of Clinical and Molecular Medicine, Sapienza University, Rome, Italy; ^2^Headache Center, Carlo Besta Neurological Institute and Foundation, Milano, Italy; ^3^Istituto di Ricovero e Cura a Carattere Scientifico (IRCCS) Istituto delle Scienze Neurologiche di Bologna, Bologna, Italy; ^4^Department of Neuroscience, University of Turin, Turin, Italy; ^5^Headache Centre, University Hospital of Careggi, Florence, Italy; ^6^IRCCS Neuromed, Pozzilli (IS), Italy; ^7^Neurologic Clinic, Santa Maria della Misericordia Hospital, Perugia, Italy; ^8^Headache and Pain Unit, IRCCS San Raffaele Pisana, Rome, Italy; ^9^Headache Science Centre, IRCCS C. Mondino Foundation, Pavia, Italy; ^10^University of Pavia, Pavia, Italy; ^11^electroCore, LLC, Basking Ridge, New Jersey, USA; ^12^Neurophysiology and Pain Unit, University of Bari Aldo Moro, Bari, Italy

###### **Correspondence:** Paolo Martelletti (paolo.martelletti@uniroma1.it)

**Background:** The randomised sham-controlled PRESTO trial provided Class I evidence that for patients with an episodic migraine, non-invasive vagus nerve stimulation (nVNS; gammaCore®) significantly increases the probability of having mild pain or being pain-free 2 hours post‑stimulation. Here, we used a range of end points beyond those previously reported to evaluate the consistency and durability of the efficacy of nVNS in PRESTO.

**Methods:** The methods for PRESTO were previously reported [1]. Additional end points reported here include the percentages of all treated attacks that were aborted (pain-free) and of those with pain relief (decrease from moderate or severe pain to mild or no pain) at 30, 60, and 120 minutes, mean change in pain score at 30, 60, and 120 minutes, sustained pain-free and pain relief response rates through 24 and 48 hours, and acute medication use.

**Results:** Response rates at 120 minutes for all treated attacks were significantly higher with nVNS than with sham for pain freedom (22.9% vs 14.8%; *p*=0.026) and pain relief (35.2% vs 24.4%; *p*=0.018). Superiority of nVNS vs sham was also shown at 60 minutes. Mean changes in pain score from baseline to 120 minutes were –0.62 (nVNS) vs –0.23 (sham) for the first attack (*p*=0.011) and –0.50 (nVNS) vs –0.28 (sham) for all attacks (*p*=0.057), with similar results seen at 60 minutes. Sustained response rates were high in both treatment groups (24 h: ≥75%; 48 h, ≥58%) for the first attack and all attacks. Acute medications used per attack decreased from the run-in period (0.86) to the double-blind period (nVNS, 0.45; sham, 0.55; *p*=0.054). All results from the nVNS group in the double-blind period were consistent with those from the open-label period.

**Conclusions:** These findings from the PRESTO trial further support the efficacy and reliability of acute nVNS for migraine. nVNS was effective when measured by first attack or all attacks, suggesting that nVNS is an attractive option for treating multiple attacks while reducing the need for current acute medications and any associated adverse events or concerns of overuse.

**Acknowledgements**

PRESTO was sponsored by electroCore, LLC. We present this abstract on behalf of the PRESTO Study Group.

**Trial registration:** NCT02686034

**Ethics approval**

PRESTO was approved by the local ethics committee for each study site.

**Reference**

1. Tassorelli C, Grazzi L, de Tommaso M, et al. Non-invasive vagus nerve stimulation as acute therapy for migraine: the randomized PRESTO study. *Neurology*. In press.

**Author Disclosures:**

**P. Martelletti** has received research grants, advisory board fees, or travel fees from ACRAF; Allergan S.p.A.; Amgen Inc.; electroCore, LLC; Novartis AG; and Teva Pharmaceutical Industries Ltd.

**L. Grazzi** has received consultancy and advisory fees from Allergan S.p.A. and electroCore, LLC.

**G. Pierangeli** has nothing to disclose.

**I. Rainero** has received consultancy fees from electroCore, LLC, and Mylan N.V. and research grants from the European Commission – Horizon 2020. He is also a principal investigator for RCTs sponsored by Axovant Sciences Ltd. and TauRx Pharmaceuticals Ltd.

**P. Geppetti** has received consultancy fees from Allergan S.p.A.; electroCore, LLC; Evidera; Novartis AG; Pfizer Inc.; and Sanofi S.p.A. and research grants from Chiesi Farmaceutici S.p.A. He is also a principal investigator for RCTs sponsored by Eli Lilly and Company; Novartis AG; and Teva Pharmaceutical Industries Ltd.

**A. Ambrosini** has received consultancy fees from Almirall, S.A., and travel grants from Allergan S.p.A. and Almirall, S.A.

**P. Sarchielli** has received clinical study fees from Allergan S.p.A.

**P. Barbanti** has received consultancy fees from Allergan S.p.A.; electroCore, LLC; Janssen Pharmaceuticals, Inc.; Lusofarmaco; and Visufarma and advisory fees from Abbott Laboratories; Merck & Co., Inc.; Novartis AG; and Teva Pharmaceutical Industries Ltd. He is also a principal investigator for RCTs sponsored by Alder BioPharmaceuticals Inc.; Eli Lilly and Company; GlaxoSmithKline Pharmaceuticals Ltd.; and Teva Pharmaceutical Industries Ltd.

**C. Tassorelli** has received consultancy fees from Allergan S.p.A.; electroCore, LLC; Eli Lilly and Company; and Novartis AG and research grants from the European Commission and the Italian Ministry of Health. She is also a principal investigator or collaborator for RCTs sponsored by Alder BioPharmaceuticals Inc.; Eli Lilly and Company; and Teva Pharmaceutical Industries Ltd.

**E. Liebler** is an employee of electroCore, LLC, and receives stock ownership.

**M. de Tommaso** has received advisory fees from Allergan S.p.A.; Neopharmed; and Pfizer Inc.

### O29 Tocilizumab in patients with giant cell arteritis: results from a Phase 3 randomized controlled trial

#### Susan P. Mollan^1*^, Katie Tuckwell^2^, Sophie Dimonaco^2^, Micki Klearman^3^, Neil Collinson^2^, John H. Stone^4^

##### ^1^Birmingham Neuro-Ophthalmology Unit, Department of Ophthalmology, University Hospitals Birmingham NHS Trust, Queen Elizabeth Hospital Birmingham, Birmingham, United Kingdom; ^2^Roche Products Ltd., Welwyn Garden City, United Kingdom; ^3^Genentech, South San Francisco, California; ^4^Massachusetts General Hospital Rheumatology Unit, Harvard Medical School, Boston, Massachusetts, United States

###### **Correspondence:** Susan P. Mollan (susan.mollan@uhb.nhs.uk)

**Introduction:** The efficacy and safety of tocilizumab (TCZ), an IL-6 receptor-α inhibitor, was evaluated in patients with giant cell arteritis (GCA) in GiACTA, a randomized, double-blind, placebo-controlled trial with blinded glucocorticoid regimens of variable dose and duration^1^. Data for the week-52 primary outcome measurement are presented.

**Methods:** Patients aged ≥50 years had active GCA previously confirmed by temporal artery biopsy or cross-sectional imaging and documented acute-phase reactant elevation attributable to GCA. Randomization was stratified by baseline prednisone dose (≤30 or >30 mg/day) as selected by the investigator (20-60 mg/day). Patients were randomized 1:1:2:1 to 4 groups: short-course prednisone (SCP) or long-course prednisone (LCP) (26-week or 52-week prednisone taper + weekly subcutaneous [SC] placebo, respectively); or weekly (TCZ-QW) or every other week (TCZ-Q2W) SC TCZ 162 mg + 26-week prednisone taper. Prednisone doses <20 mg/day were blinded. Patients who flared or could not adhere to the protocol-defined tapering schedule received open-label prednisone escape therapy but continued on double-blind TCZ/placebo. Sustained remission was defined as the absence of flare at week 52, normalization of C-reactive protein after week 12, and adherence to the protocol-defined prednisone taper. The primary and key secondary endpoint was the proportion of patients in sustained remission, comparing TCZ groups with the SCP and LCP groups (significance level, 0.005). A dose hierarchy of statistical testing was implemented. Prednisone exposure was a secondary endpoint.

**Results:** Among 251 patients randomized, the mean ± SD age was 69 ± 8.2 years, and 75% were female. A total of 56% and 53.1% of patients in the TCZ-QW and TCZ-Q2W groups, respectively, achieved sustained remission compared with 14% in the SCP group (*p*< 0.0001; primary endpoint) and 17.6% in the LCP group (*p*≤ 0.0002; key secondary endpoint). The median cumulative steroid exposure was 1862.0 in both TCZ groups, and 3296.0, and 3817.5, in the SCP and LCP groups, respectively. The incidence of adverse events (AEs) was similar among the 4 groups. Serious AEs were reported in 15.0%, 14.3%, 22.0%, and 25.5% of TCZ-QW, TCZ-Q2W, SCP, and LCP patients, respectively. No deaths or new vision loss occurred.

**Conclusion:** TCZ plus 26-week prednisone taper was superior to SCP and LCP tapers for sustained remission at 52 weeks. TCZ plus prednisone also led to a substantial reduction in the cumulative prednisone doses required to control GCA.

**Trial registration:** NCT01791153 / EudraCT 2011-006022-25. This study was funded by F. Hoffmann-La Roche Ltd.

**Ethics approval**

Protocol approval was obtained from institutional review boards, ethics committees, and/or regulatory authorities in accordance with the Declaration of Helsinki and Good Clinical Practice was followed.

**Reference**

1. Stone J.H, Tuckwell K, Dimonaco S, et al. N Engl J Med 2017;377:317-28.

### O30 Response to lasmiditan for acute treatment of migraine based on prior response to triptan therapy

#### Kerry Knievel^1^, Louise Lombard^2^, Andrew Buchanan^2^, Simin Baygani^2^, Joel Raskin^2^, Joshua Tobin^3^

##### ^1^Barrow Neurological Institute, Phoenix, AZ 85013, USA; ^2^Eli Lilly and Company, Indianapolis, IN 46285, USA; ^3^21^st^ Century Neurology, Xenoscience, Phoenix, AZ 85013, USA

###### **Correspondence:** Kerry Knievel; Joshua Tobin (josh.bytheway@rxcomms.com)

**Background**: In the American Migraine Prevalence and Prevention Study, >40% of patients with episodic migraine had unmet acute treatment needs with current therapies, including lack of efficacy and intolerance; those with ≥1 unmet need were more likely to have used triptans in the past 3 months [1]. Lasmiditan, a novel, central nervous system penetrant, highly selective 5-hydroxytryptamine_1F_ receptor agonist, has demonstrated superiority to placebo in the acute treatment of migraine in adults. In two Phase 3 studies, SAMURAI (NCT02439320) and SPARTAN (NCT02605174), the percentage of patients who were migraine pain-free 2 hours post-first dose was significantly greater with lasmiditan 200mg and 100mg (all comparisons p<0.001 vs placebo) taken within 4 hours of a single migraine attack. This post-hoc analysis determined whether response to lasmiditan differed according to prior triptan therapy response in participants in SAMURAI and SPARTAN.

**Methods**: Both studies included patients with moderate/severe migraine disability (MIDAS score ≥11). Current analyses considered combined data from participants reporting triptan use within 3 months prior to screening randomized to receive lasmiditan 100mg or 200mg, or placebo, as first dose. At baseline, patients rated themselves as good, poor or nonresponders prior to triptan therapy. To determine whether therapeutic benefit varied according to prior triptan response, treatment-by-subgroup analyses of 2-hour outcomes used a logistic regression model, including study, treatment, rescue medication use and triptan responder subgroup (good vs poor/nonresponder). Significance for interaction was defined as p<0.1.

**Results**: In combined analyses, there was no evidence that the benefit (on headache pain freedom, most bothersome symptom [MBS] freedom, headache pain relief) of lasmiditan 200mg versus placebo varied significantly between triptan responder subgroups. A significant differential benefit of lasmiditan 100mg over placebo favouring poor/nonresponders vs good responders was seen for headache pain freedom (odds ratio 4.5 vs 1.8, p=0.056) and MBS freedom (odds ratio 3.2 vs 1.5, p=0.042). Risk relative to placebo of experiencing a treatment-emergent adverse event was significantly lower for poor/nonresponders vs good responders for each lasmiditan dose. Significant differences between subgroups were not consistently seen in individual studies.

**Conclusion**: Therapeutic benefit of lasmiditan in patients with moderate/severe migraine disability was generally unaffected by prior triptan therapy response. Lasmiditan offers a possible alternative migraine therapy option for patients regardless of prior response to triptans.

**References**

1. Lipton RB, Buse DC, Serrano D, et al. Examination of unmet treatment needs among persons with episodic migraine: results of the American Migraine Prevalence and Prevention (AMPP) Study. *Headache* 2013;53:1300-11.

### O31 Effect of galcanezumab on severity and symptoms of migraine in phase 3 trials in patients with episodic or chronic migraine

#### Kathleen A. Day^1^, Michael Ament^2^, Virginia L. Stauffer^1^, Vladimir Skljarevski^1^, Qi Zhang^1^, Eric M. Pearlman^1^, Sheena K Aurora^1^

##### ^1^Eli Lilly and Company, Indianapolis, IN 46285 USA; ^2^Ament Headache Center, Denver, CO, 80206, USA

###### **Correspondence:** Virginia L. Stauffer (stauffer_virginia@lilly.com)

**Objective**: Galcanezumab, a humanized monoclonal antibody that binds CGRP, has demonstrated in multiple studies, a significant reduction in monthly migraine headache days compared to placebo. Here, we present data from 3 randomized clinical trials in patients with migraine showing that galcanezumab alleviates severity and symptoms of migraine.

**Methods**: EVOLVE-1 and EVOLVE-2 were 6-month double-blind studies conducted in North America and globally, respectively, in male and female patients 18–65 years of age with episodic migraine (i.e., 4–14 monthly migraine headache days [MHDs]). REGAIN was a global study with a 3-month double-blind phase in patients with chronic migraine (≥15 headache days [HDs] per month, where ≥8 met criteria for migraine). For the 3 studies, patients were randomized 2:1:1 to subcutaneous monthly injections of placebo, or 120 or 240 mg of galcanezumab. Patients randomized to the 120 mg galcanezumab group received a loading dose of 240 mg at randomization. Patients recorded headache characteristics, duration, and severity, as well as presence of nausea and vomiting, photophobia, phonophobia, and aura with each headache attack in an electronic patient-reported outcome (ePRO) diary. The reported parameters were changes from baseline in moderate to severe monthly HDs, mean severity of remaining MHDs, and monthly MHDs with nausea and/or vomiting, photophobia and phonophobia, aura, and prodromal symptoms other than aura. Rating for migraine severity was 1=mild, 2=moderate, and 3=severe. Analyses were conducted on the intent-to-treat population and those who had a non-missing baseline value and at least one post-baseline value. Change from baseline in severity and symptoms parameters were analyzed using a linear mixed model repeated measures approach.

**Results**: With the exception of “Mean severity of remaining MHDs” in EVOLVE-1 and “Monthly MHDs with aura” in REGAIN, both doses of galcanezumab were statistically significantly superior to placebo (p<.05) in reducing migraine symptoms and severity (Table 1).

**Conclusions**: Galcanezumab was superior to placebo in reducing the mean severity of remaining migraine headaches and in reducing the numbers of monthly moderate to severe headaches in patients with episodic or chronic migraine. Galcanezumab also reduced the frequency of associated symptoms of migraine, with the exception of aura, which was reduced in patients with episodic, but not chronic, migraine. Overall, along with the previously reported reductions in monthly MHDs, galcanezumab can alleviate potentially disabling symptoms in patients with migraine.

**Trial registration**: ClinicalTrials.gov identifiers NCT02614183 (EVOLVE-1), NCT02614196 (EVOLVE-2), and NCT02614261 (REGAIN)

**Ethics approval**

These studies were approved by the appropriate institutional review board for each of the study sites. They were all conducted according to Good Clinical Practice and the Declaration of Helsinki guidelines.

**Table 1 (abstract O31).** Summary of overall results

Δ from BL in:		EVOLVE-1 Galcanezumab		EVOLVE-2 Galcanezumab		REGAIN Galcanezumab	
		120 mg (N=210)	240 mg (N=208)	120 mg (N=226)	240 mg (N=220)	120 mg (N=273)	240 mg (N=274)
Moderate to severe monthly HDs	Δ (SE) vs. BL (PBO)	-2.89 (0.21)		-2.31 (0.18)		-2.92 (0.33)	
	Δ (SE) vs. BL	-4.24 (0.25)	-4.15 (0.25)	-3.90 (0.22)	-3.78 (0.22)	-4.90 (0.40)	-4.64 (0.39)
	Δ (SE) vs. PBO	-1.35 (0.23)	-1.26 (0.24)	-1.59 (0.23)	-1.47 (0.24)	-1.98 (0.38)	-1.72 (0.38)
	95% CI vs. PBO	(-1.81, -0.89)	(-1.72, -0.80)	(-2.05, -1.13)	(-1.93, -1.01)	(-2.73, -1.23)	(-2.47, -0.97)
	p-value vs. PBO	<.001	<.001	<.001	<.001	<.001	<.001
Mean severity of remaining MHDs	Δ (SE) vs. BL (PBO)	-0.17 (0.02)		-0.15 (0.02)		-0.12 (0.02)	
	Δ (SE) vs. BL	-0.19 (0.03)	-0.22 (0.03)	-0.20 (0.03)	-0.22 (0.03)	-0.19 (0.02)	-0.19 (0.02)
	Δ (SE) vs. PBO	-0.02 (0.03)	-0.05 (0.03)	-0.06 (0.03)	-0.08 (0.03)	-0.07 (0.02)	-0.07 (0.02)
	95% CI vs. PBO	(-0.07, 0.03)	(-0.10, 0.01)	(-0.11, -0.01)	(-0.13, -0.03)	(-0.11, -0.03)	(-0.11, -0.03)
	p-value vs. PBO	.447	.086	.031	.004	<.001	<.001
Monthly MHDs with nausea and/or vomiting	Δ (SE) vs. BL (PBO)	-1.17 (0.16)		-0.87 (0.13)		-1.92 (0.27)	
	Δ (SE) vs. BL	-1.91 (0.19)	-2.05 (0.19)	-2.02 (0.17)	-1.77 (0.17)	-3.13 (0.33)	-3.20 (0.33)
	Δ (SE) vs. PBO	-0.74 (0.18)	-0.88 (0.18)	-1.14 (0.18)	-0.90 (0.18)	-1.21 (0.32)	-1.28 (0.31)
	95% CL for Δ	(-1.10, -0.39)	(-1.23, -0.52)	-0.90 (0.18))	(-1.25, -0.55)	(-1.82, -0.59)	(-1.90, -0.66)
	p-value vs. PBO	<.001	<.001	<.001	<.001	<.001	<.001
Monthly MHDs with photophobia and phonophobia	Δ (SE) vs. BL (PBO)	-2.10 (0.23)		-1.47 (0.19)		-2.25 (0.36)	
	Δ (SE) vs. BL	-3.50 (0.27)	-3.54 (0.27)	-3.22 (0.24)	-3.00 (0.24)	-3.81 (0.43)	-3.58 (0.43)
	Δ (SE) vs. PBO	-1.39 (0.26)	-1.43 (0.26)	-1.76 (0.25)	-1.53 (0.25)	-1.56 (0.41)	-1.33 (0.41)
	95% CI vs. PBO	(-1.90, -0.89)	(-1.94, -0.93)	(-2.25, -1.27)	(-2.02, -1.04)	(-2.37, -0.75)	(-2.14, -0.52)
	p-value vs. PBO	<.001	<.001	<.001	<.001	<.001	.001
Monthly MHDs with aura	Δ (SE) vs. BL (PBO)	-0.96 (0.12)		-0.97 (0.12)		-1.42 (0.24)	
	Δ (SE) vs. BL	-1.39 (0.14)	-1.38 (0.15)	-1.45 (0.15)	-1.44 (0.16)	-1.40 (0.29)	-1.89 (0.29)
	Δ (SE) vs. PBO	-0.43 (0.14)	-0.42 (0.14)	-0.48 (0.17)	-0.48 (0.17)	0.03 (0.28)	-0.47 (0.28)
	95% CI vs. PBO	(-0.70, -0.16)	(-0.70, -0.15)	(-0.81, -0.16)	(-0.80, -0.15)	(-0.53, 0.58)	(-1.02, 0.09)
	p-value vs. PBO	.002	.002	.004	.004	.922	.098
Monthly MHDs with prodromal symptoms other than aura	Δ (SE) vs. BL (PBO)	-1.23 (0.14)		-1.01 (0.14)		-1.15 (0.28)	
	Δ (SE) vs. BL	-1.83 (0.17)	-1.69 (0.17)	-1.84 (0.17)	-1.74 (0.17)	-1.81 (0.34)	-2.18 (0.33)
	Δ (SE) vs. PBO	-0.61 (0.17)	-0.46 (0.17)	-0.83 (0.18)	-0.72 (0.18)	-0.66 (0.32)	-1.03 (0.32)
	95% CI vs. PBO	(-0.93, -0.28)	(-0.79, -0.14)	(-1.18, -0.47)	(-1.08, -0.36)	(-1.29, -0.02)	(-1.67, -0.40)
	p-value vs. PBO	<.001	. 006	<.001	<.001	.042	.001

*Abbreviations: Δ* change in least squares mean; *BL* baseline, *CI* confidence interval, *HDs* headache days, *MHDs* migraine headache days, *N* number of intent-to-treat patients who have non-missing baseline value and at least one post-baseline value; N’s differed slightly for mean severity of remaining MHDs. *PBO* placebo, *SE* standard error

Shaded cells represent change from baseline for placebo groups; N=425, 450, and 538 for EVOLVE-1, EVOLVE-2, and REGAIN, respectively

### O32 Factors associated with significant reduction in migraine headache days: a post hoc analysis of phase 3 placebo-controlled trials of patients with episodic and chronic migraine treated with galcanezumab

#### Sheena K. Aurora, Dustin D. Ruff, Qi Zhang, Eric M. Pearlman

##### Lilly Corporate Center, Eli Lilly and Company, Indianapolis, IN, USA

###### **Correspondence:** Qi Zhang (zhang_qi_x1@lilly.com)

**Background**: Migraine remains undertreated with few approved preventive medications available. Galcanezumab, a humanized monoclonal antibody that binds to calcitonin gene-related peptide (CGRP), was developed for the prevention of migraine.

**Objectives**: To determine if previously seen galcanezumab response predictors are revalidated in phase III episodic and chronic migraine studies.

**Methods**: This *post hoc* analysis of 3 randomized, double-blind, placebo-controlled Phase 3 studies evaluated the effects of galcanezumab in patients aged 18-65 years. Results of two episodic migraine (EM) studies (NCT02614183, NCT02614196) that enrolled patients with a baseline of 4-14 migraine headache days (MHD) per month were pooled. One chronic migraine (CM) study (NCT02614261) enrolled patients with ≥15 headache days per month, of which 8 had migraine-like features. In all studies, patients were randomized 2:1:1 to placebo, galcanezumab 120 mg, or galcanezumab 240 mg given subcutaneously once monthly. Three possible predictors of clinical response – prior triptan use, migraine history ≥20 years, and history of failure to preventive treatments – were evaluated as subgroup analyses of 50% response rate (RR) (≥50% reduction in number of MHD). The overall treatment-by-subgroup interaction p-values for the double-blind phase (EM: 6 months; CM: 3 months) are reported with a 2-sided significance level of 0.10. The studies were approved by the appropriate Institutional Review Board for each study site.

**Results**: Baseline characteristics were generally consistent across the 3 studies, with the exception of mean headache days (EM: 10.67 (n=1773) vs. CM: 21.44 (n=1113)) and MHD (9.13 vs. 19.41). Both galcanezumab doses were associated with significantly higher 50% RRs than placebo in most of the examined subgroups for EM and CM. For the 120 mg dose vs. placebo in the EM and CM studies, a greater treatment effect was seen for those with vs. without history of failure of ≥1 preventive treatment (treatment-by-subgroup interaction EM: p=0.012; CM: p<0.001), and for those who used vs. did not use a triptan at baseline (EM: p<0.001; CM: p=0.062). Migraine diagnosis ≥20 years previously was predictive of response to the 120 mg dose for individuals with EM (p=0.056) but not for those with CM (p=0.206).

**Conclusion**: Analysis of this large data set expands previous findings and suggests that there may be some predictors of clinical response to galcanezumab compared with placebo in EM and CM, primarily driven by placebo response fluctuations, as the magnitude of 50% RR with galcanezumab was similar in all subgroups but was lower and varied with placebo.

### O33 Effect of galcanezumab on possible menstrual-related migraine: exploratory analyses results from EVOLVE-1, EVOLVE-2 and REGAIN

#### Maria S. Fernandes,^1^ Holland C. Detke,^1^ Qi Zhang,^1^ Jelena M. Pavlovic,^2^ Sheena K. Aurora^1^

##### ^1^Eli Lilly and Company, Indianapolis, IN 46285 USA; ^2^Albert Einstein College of Medicine, Montefiore Headache Center, Bronx, NY 10461USA

###### **Correspondence:** Sheena K. Aurora (sheena.aurora@lilly.com)

**Background:** Migraine is a common neurologic disease that affects more than 36 million people in the Unites States, being more prevalent in females of reproductive age. Attacks of migraine in more than 50% of women correlate with menstrual cycle hormonal fluctuations, and are typically more severe and difficult to manage with conventional therapies. Menstrual migraine attacks are defined as attacks occurring during the 5-day perimenstrual interval (-2 to +3, where first day of bleeding is defined as +1). These exploratory post-hoc analyses focus on the effect of galcanezumab, a Calcitonin gene-related peptide (CGRP) monoclonal antibody studied for the prevention of chronic and episodic migraine, on the incidence and severity of menstrual migraine attacks.

**Methods:** Post-hoc analyses were performed using data from 3 double-blind, placebo (PBO)-controlled, Phase 3 studies in patients aged 18-65 years with episodic (EVOLVE-1 & EVOLVE-2) or chronic migraine (REGAIN). A total of 2,886 patients (858 EVOLVE-1, 915 EVOLVE-2, 1113 REGAIN) randomly received 120mg (with 240mg loading dose at first month) or 240mg galcanezumab (GMB) or placebo (PBO), which was administered subcutaneously once/month for 6 months in EVOLVE-1&2 and for 3 months in REGAIN. Menstruation was assessed on a daily basis via self-reported diary, as well as headache characteristics, duration, and severity.

A menstrual-related migraine headache day (MRMHD) is defined as a headache with migraine characteristics (definition adapted from the standard IHS ICHD-3 beta definition) within the 5-day perimenstrual period (-2 to +3). Exploratory analyses included intent-to-treat (ITT) patients who had >0 MRMHD during a one-month baseline period and who were females with menstrual periods. Using a negative binomial repeated measures model, the number of MRMHDs per 30-day period was estimated each month and overall across 6 months for pooled EVOLVE-1 & EVOLVE-2 data, and across 3 months for REGAIN.

**Results:** For ITT patients with >0 MRMHD, baseline mean number of MRMHDs were 2.4, 2.4 and 2.6 for 120mg GMB, 240mg GMB, and PBO, respectively, for pooled EVOLVE-1 & EVOLVE-2 studies (n=650). Corresponding values were 4.0, 4.5, and 4.4 days, respectively, for REGAIN (n=407). Statistically significantly lower incidence of MRMHDs per 30-day period was observed for both GMB doses compared with PBO overall across 6 months for pooled EVOLVE-1 & EVOLVE-2 and across 3 months for REGAIN (Table 1).

**Conclusion:** Galcanezumab, given monthly, was effective in reducing migraine headache days during the perimenstrual period.

The studies were approved by a central Ethics Review Board and registered on ClinTrials.gov (NCT02614183, NCT02614196, and NCT02614261).

**Table 1 (abstract O33).** Estimated Number of Menstrual-Related Migraine Headache Days

Period	Treatment	N	Estimated Number of MRMHDs per 30 day Period (SE)	95% CI	Rate Ratio per 30 day period	95% CI	P-value
ITT patients with baseline number of MRMHDs >0 for Episodic Migraine							
Average of Month 1 to 6	PBO	321	1.58 (0.13)	1.34, 1.86			
	120mg	170	1.16 (0.12)	0.96, 1.41	0.74	0.64, 0.85	<0.001
	240mg	159	1.12 (0.12)	0.90, 1.38	0.71	0.60, 0.83	<0.001
ITT patients with baseline number of MRMHDs >0 for Chronic Migraine							
Average of Month 1 to 3	PBO	198	3.52 (0.18)	3.18, 3.90			
	120mg	112	2.58 (0.21)	2.58, 3.40	0.84	0.73, 0.97	0.015
	240mg	97	3.01 (0.20)	2.64, 3.42	0.85	0.75, 0.98	0.021

*MRMHD* Menstrual-Related Migraine Headache Days, *CI* confidence interval, *ITT* intent-to-treat, *MM* menstrual migraine, *PBO* placebo, *SE* standard error

### O34 Primary angiitis of the central nervous system and reversible cerebral vasoconstriction syndrome: a comparative study of two cohorts

#### Hubert de Boysson^1^, Jean-Jacques Parienti^2^, Jérôme Mawet^3^, Caroline Arquizan^4^, Grégoire Boulouis^5^, Cécilia Burcin^3^, Olivier Naggara^6^, Mathieu Zuber^7^, Emmanuel Touzé^8^, Achille Aouba^1^, Marie-Germaine Bousser^9^, Christian Pagnoux^10^, Anne Ducros^5^

##### ^1^Internal Medicine, Caen University Hospital, Caen, France; ^2^Biostatistics, Caen University Hospital, France; ^3^Emergency Headache Centre, Lariboisière Hospital, APHP, Paris, France; ^5^Neurology, Montpellier University Hospital, Montpellier, France; ^6^Neuroradiology, Centre Hospitalier Sainte-Anne, Paris-Descartes University, Paris, France; ^7^Vascular Neurology - Saint Joseph Hospital, Paris, France; ^8^Neurology, Caen University Hospital, France; ^9^Neurology, Lariboisière Hospital, APHP, Paris, France; ^10^Rheumatology, Mount Sinai Hospital, Toronto, Canada

###### **Correspondence:** Anne Ducros (a-ducros@chu-montpellier.fr)

Distinguishing primary angiitis of the central nervous system (PACNS) and reversible cerebral vasoconstriction syndrome (RCVS) can remain a challenge in clinical practice.

We compared two large French cohorts of patients with biopsy or angiographically-proven PACNS (n=110) to patients with angiographically-proven RCVS (n=173) in order to better define differences at onset.

Patients with RCVS were more often women (71% versus 47% in PACNS, p<0.0001) and more often had a past history of migraine (32% versus 7%, p<0.0001). While headaches, especially thunderclap headaches, were more frequent in RCVS (99% versus 54% in PACNS and 94% versus 3% in PACNS, respectively, both p<0.0001), all other neurological symptoms (focal deficits, seizures, vigilance impairment) were more frequent in PACNS (all symptoms, p<0.0001), even when focusing only on angiography-diagnosed PACNS patients or on RCVS patients with abnormal brain imaging.

Brain MRI was abnormal in every PACNS patient, but only in 53 (31%) RCVS patients (p<0.0001). Acute ischemic stroke was more frequent in PACNS than in RCVS (76% versus 8%, p<0.0001). While intracerebral hemorrhage was more frequent in PACNS (20% versus 9% in RCVS, p=0.006), subarachnoid hemorrhage predominated in RCVS (27% versus 16% in PACNS, p=0.04). In a multivariate analysis, female sex and thunderclap headache were the two strongest variables associated with RCVS, whereas motor deficit and acute brain infarction were the two strongest variables associated with PACNS.

The clinical context (female sex, past history of migraine) and the characteristics of headache (thunderclap) combined with brain MRI features enable to distinguish RCVS from PACNS.

### O35 VALIDATION OF THE ITALIAN VERSION OF THE “IDENTIFY CHRONIC MIGRAINE” SCREENING TOOL: THE IT-EC-ID

#### Simona Sacco^1^, Silvia Benemei^2^, Sabina Cevoli^3,4,^ Gianluca Coppola^5^, Pietro Cortelli^3,4^, Francesco De Cesaris^2^, Roberto De Icco^6^, Cristiano De Marco^7^, Cherubino Di Lorenzo^5^, Luana Evangelista^1^, Pierangelo Geppetti^2^, Alessia Manni^8^, Andrea Negro^7^, Giulia Pierangeli^3,4^, Francesco Pierelli^9^, Lanfranco Pellesi^10^, Luigi Alberto Pini^10^, Francesca Pistoia^1^, Maria Pia Prudenzano^8^, Antonio Russo^11^, Grazia Sances^6^, Valentina Taranta^1^, Cristina Tassorelli^6,12^, Gioacchino Tedeschi^11^, Maria Trojano^8^, Paolo Martelletti^7^

##### ^1^Department of Applied Clinical Sciences and Biotechnology, Section of Neurology, University of L’Aquila, L’Aquila, Italy; ^2^Department of Health Sciences, Section of Clinical Pharmacology and Oncology, University of Florence, Florence, Italy; ^3^IRCCS Institute of Neurological Science of Bologna, Bologna, Italy; ^4^Department of Biomedical and Neuromotor Sciences, University of Bologna, Bologna, Italy; ^5^Department of Medico-Surgical Sciences and Biotechnologies, Sapienza University Polo Pontino, Latina, Italy; ^6^Headache Science Center, IRCCS C. Mondino Foundation, Pavia, Italy; ^7^Department of Clinical and Molecular Medicine, Regional Referral Headache Centre, Sant'Andrea Hospital, Sapienza University, Rome, Italy; ^8^Department of Basic Medical Sciences, Neurosciences and Sense Organs, University of Bari, Italy; ^9^IRCCS NEUROMED, Pozzilli (IS), Italy; ^10^Headache and Drug Abuse Research Centre, Policlinico Hospital, University of Modena e Reggio Emilia, Modena, Italy; ^11^Department of Medical, Surgical, Neurological, Metabolic and Aging Sciences, Second University of Naples, Napoli, Italy; ^12^Department of Brain and Behavioral Sciences, University of Pavia, Pavia, Italy

###### **Correspondence:** Simona Sacco (simona.sacco@univaq.it)

**Background**

Chronic migraine (CM) is an underdiagnosed and undertreated condition. Tools to improve CM detection by health care professionals may enhance case finding and promote proper referral to dedicated care. Recently, the self-administered tool ‘Identify Chronic Migraine (ID-CM)’ proved to be a reliable instrument to identify patients likely to suffer from CM [1]. An Italian version of the ID-CM (IT-EC-CM) was developed according to a standardized methodology [2]. The aim of the present study was to validate IT-EC-ID on a sample of headache sufferers seeking consultation at tertiary headache centres in Italy.

**Material and Methods**

We included all consecutive patients, aged 18 years or older, consulting 9 selected tertiary Headache Centers for a first in-person visit for the presence of headache. We excluded patients who had language barrier, or any other condition, including cognitive disturbances that could affect the capability of filling the IT-EC-ID. Patients, self-filled the IT-EC-ID before the in-person visit. Thereafter, a clinical diagnosis, according to the International Classification of Headache Disorders (ICHD), III revision beta version, was assigned by a headache expert blinded to the IT-EC-ID results. This diagnosis was used as the gold standard for the validation of the diagnosis assigned by the IT-EC-ID. The study was approved by the local Ethic Committees of each of the participating centers.

**Results**

From November 2017 to April 2018, we included 532 subjects with headache (81% women, mean age 42.0±13.0). According to the clinical diagnosis, 209 (39.3%) of them suffered with CM. We excluded 39 patients (1 unwilling to complete the IT-EC-ID and 38 with incomplete IT-EC-ID). Among the 493 patients, eligible for the validation procedure, we had 185 patients diagnosed with CM according to ICHD-III and 218 patients with possible CM according to the IT-EC-ID. True positive cases were 150, true negative 240, false positive were 68, and false negative were 35. The sensitivity for the IT-ID-CM was 81.1% with a specificity was 77.8%, a negative predictive value of 86.0%, and a positive predictive value of 70.9%. Among the false positive, we found 73.7% of the patients with medication overuse without CM, 54.5% of patients with chronic tension type headache, 30.0% of patients with episodic tension type headache, and 14.3% of patients with migraine.

**Conclusions**

The sensitivity and specificity achieved by IT-EC-ID compares favorably with those achieved by the ID-CM, indicating that it is a reliable tool for identifying CM patients and for excluding patients with other headache forms. This study is still ongoing and an electronic version of the IT-EC-ID is being validated in the selected headache centers. The further step will be to test the reliability of the IT-ID-CM in the primary care setting and the feasibility of its use in the identification of subjects with CM for fast-track access to dedicated care.

**Acknowledgements**

*This project was supported by an unconditional grant from Allergan Italy to the Fondazione Italiana per lo Studio delle Cefalee Onlus.*

**References**

1. Lipton RB, Serrano D, Buse DW, Pavlovic JM, Blumenfeld AM, Dodick DW, Aurora SK, Becker WJ, Diener H-S, Wang S-J, Vincent MB, Hindiyeh NA, Starling AJ, Gillard PJ, Varon SF, Reed ML (2016) Improving the detection of chronic migraine: Development and validation of Identify Chronic Migraine (ID-CM). Cephalalgia 36:203–215.

2. Sacco S, Benemei S, Cevoli S, Coppola G, Cortelli P, De Cesaris F, De Marco C, Di Lorenzo C, Evangelista L, Geppetti P, Negro A, Pierangeli G, Pierelli F, Pini LA, Pistoia F, Russo A, Tassorelli C, Tedeschi G, Martelletti P. Development of the Italian version of the “identify chronic migraine” (IT-ID-CM). J Headache Pain 2017;18(Suppl 1):P43.

### O36 Migraine progression in natural subgroups of migraine based on comorbidities and concomitant conditions: results of the Chronic Migraine Epidemiology and Outcomes (CaMEO) study

#### Richard B. Lipton^1^, Vincent T. Martin^2,^ Michael L. Reed^3,^ Kristina M. Fanning,^3^ Aubrey Manack Adams,^4^ Dawn C. Buse,^1^

##### ^1^Department of Neurology, Albert Einstein College of Medicine, Bronx, NY, 10461, USA; ^2^University of Cincinnati Headache and Facial Pain Center, University of Cincinnati College of Medicine, Cincinnati, OH, 45267, USA; ^3^Vedanta Research, Chapel Hill, NC, 27517, USA; ^4^Allergan plc, Irvine, CA, 92612, USA

###### **Correspondence:** Richard B. Lipton (Richard.Lipton@einstein.yu.edu)

**Background:** Prior research has identified 8 natural subgroups of migraine based on profiles of comorbidities from the CaMEO Study. We tested the hypothesis that these subgroups differ in prognosis as measured by progression to chronic migraine (CM).

**Methods:** Participants from the prospective, web-based baseline CaMEO Study (ClinicalTrials.gov, NCT01648530) were identified using quota sampling. Episodic migraine (EM) and CM were distinguished. Based on respondents’ self-report, 8 subgroups were identified using latent class analysis: Most Comorbidities, Respiratory/Psychiatric (Resp/Psych), Respiratory/Pain (Resp/Pain), Respiratory, Psychiatric, Cardiovascular, Pain, and Fewest Comorbidities. Modelling approaches included forward stepwise analysis of time to CM onset in individuals with EM at baseline across 7 comorbid classes, using Fewest Comorbidities as reference. Covariates including age, gender, income, body mass index, race, Migraine Disability Assessment (MIDAS), Migraine Symptom Severity Scale (MSSS), allodynia, and medication overuse at baseline were added to the model starting with the variable providing the most significant improvement in model fit, and continuing until no further improvement was observed. The study received ethical approval from the Institutional Review Board of the Albert Einstein College of Medicine.

**Results:** In the analysis population (n=8658), MIDAS was the first variable added to the forward stepwise model, as it provided the most statistically significant improvement in fit. Medication overuse was included as step 2, followed by comorbid class variable, allodynia, income, age, gender and MSSS (Table 1). BMI and race did not further improve fit and were not added. In the final model, Most Comorbidities had the highest risk of progression to CM, 3 times higher than Fewest Comorbidities (HR, 3.01 [95% CI: 2.17, 4.18]). Addition of age tended to increase the HR for all comorbid classes; Most Comorbidities increased from 2.49 (95% CI: 1.83, 3.39) before addition of age to 3.02 after (95% CI: 2.17, 4.20).

**Conclusions:** Identified comorbid classes of migraine are associated with risk of progression from EM to CM. Understanding the biological differences among these subgroups may help minimize migraine disease progression and optimize management.

**Table 1 (abstract O36).** Forward stepwise model* for the discrete time hazard to chronic migraine onset in comorbid classes of migraine in individuals with episodic migraine at baseline

	Hazard Ratio (95% CI)*					
LCA Class	Step 3 (Comorbid Class)	Step 4 (Allodynia)	Step 5 (Income ≥50k)	Step 6 (Age)	Step 7 (Gender)	Step 8 (MSSS)
Most Comorbidities	2.80 (2.06, 3.80)	2.59 (1.91, 3.53)	2.49 (1.83, 3.39)	3.02 (2.17, 4.20)	3.07 (2.21, 4.27)	3.01^†^ (2.17, 4.18)
Resp/Psych	1.86 (1.39, 2.47)	1.75 (1.31, 2.33)	1.75 (1.32, 2.34)	1.87 (1.40, 2.49)	1.93 (1.44, 2.58)	1.87^†^ (1.40, 2.50)
Resp/Pain	2.25 (1.68, 3.02)	2.13 (1.59, 2.86)	2.21 (1.64, 2.96)	2.61 (1.91, 3.57)	2.63 (1.92, 3.60)	2.57^†^ (1.88, 3.51)
Respiratory	1.40 (1.07, 1.82)	1.33 (1.01, 1.74)	1.35 (1.03, 1.76)	1.40 (1.07, 1.83)	1.42 (1.09, 1.87)	1.41^‡^ (1.07, 1.84)
Psych	2.22 (1.63, 3.03)	2.15 (1.58, 2.94)	2.10 (1.54, 2.87)	2.09 (1.53, 2.85)	2.12 (1.55, 2.89)	2.07^†^ (1.52, 2.83)
Cardiovascular	1.21 (0.84, 1.74)	1.21 (0.84, 1.74)	1.25 (0.87, 1.80)	1.46 (1.00, 2.13)	1.40 (0.96, 2.04)	1.41 (0.96, 2.05)
Pain	1.35 (0.93, 1.97)	1.30 (0.89, 1.90)	1.32 (0.91, 1.93)	1.48 (1.01, 2.17)	1.42 (0.96, 2.09)	1.41 (0.96, 2.07)

*LCA* latent class analysis, *MSSS* Migraine Symptom Severity Scale, *Resp* respiratory, *Psych* psychiatric

*Step 1 added MIDAS and Step 2 added medication overuse and are not included in this table as the comorbid class only enters at Step 3.

^†^P≤0.05, compared with the “Fewest Comorbidities” class.

^‡^P≤0.001, compared with the “Fewest Comorbidities” class

### O37 Complete detoxification is the most feasible treatment for medication-overuse headache: A randomized controlled open-label trial

#### Ida M. S. Engelstoft, Louise N. Carlsen, Signe B. Munksgaard, Mia Nielsen, Rigmor H. Jensen, Lars Bendtsen

##### Danish Headache Center, Neurological Clinic, Rigshospitalet, Glostrup, Denmark

###### **Correspondence:** Louise N. Carlsen (louise.ninett.carlsen@regionh.dk)

**Background**: Complete stop of acute medication and/or migraine medication for treatment of medication-overuse headache (MOH) has previously been reported more effective in reducing headache days and migraine days per month compared with restricted intake of acute medication. However, it is unknown whether complete stop or restricted intake is the most feasible treatment for patients.

**Objective:** To investigate if feasibility of detoxification in medication-overuse headache (MOH) is different between complete stop of acute medication and restricted intake, and if reductions in headache-related medication dependence, anxiety and depression differ between the treatments.

**Methods:** MOH patients were included in a prospective, outpatient study and randomized to two months detoxification with either no analgesics or acute migraine-medication (program A) or acute medication restricted to two days/week (program B). After 6 and 12 months of treatment, patients graded feasibility of detoxification. Psychological dependence was measured by Severity of Dependence Scale (SDS), while anxiety and depression were measured by Hospital Anxiety and Depression Scale (HADS).

**Results**: We included 72 MOH patients with primary migraine and/or tension-type headache. Forty-nine completed detoxification and the SDS-questionnaire at 12-months follow-up, and the feasibility of detoxification was significantly higher in program A compared to program B (*p* <0.001). At 12 months, the psychological dependence was reduced by 44% in program A compared to 26% in program B (*p* = 0.053), while anxiety score was reduced by 32% and 11%, respectively (*p* = 0.048).

**Conclusion:** Detoxification with complete stop of acute medication was more feasible and also the most effective in reducing headache-related anxiety.

**Trial registration:** Clinicaltrials.gov (NCT02903329)

**Ethical approval**

The study was approved by the Regional Ethical Committee in Denmark (H-1-2012- 105 116)

### O38 Impact of fremanezumab on response rates, acute medication use, and disability in patients with episodic migraine who have failed at least one prior migraine preventive medication

#### Paul K. Winner^1^; Rashmi B. Halker Singh^2^; Joshua M. Cohen^3^; Ronghua Yang^3^; Paul P. Yeung^3^; Verena Ramirez Campos^4^

##### ^1^Premiere Research Institute, West Palm Beach, Florida, USA; ^2^Mayo Clinic, Phoenix, Arizona, USA; ^3^Teva Pharmaceuticals, Frazer, Pennsylvania, USA; ^4^Teva Pharmaceuticals, Buenos Aires, Argentina

###### **Correspondence:** Paul K. Winner (khokenson@hcg-int.com)

**BACKGROUND**:

Preventive medication is recommended for episodic migraine (EM) patients with ≥4 headache days per month. Fremanezumab, a fully humanized monoclonal antibody (IgG2a) that selectively targets calcitonin gene-related peptide (CGRP), is efficacious in preventing EM, but its effectiveness in patients who failed previous preventive medications is unknown.

**OBJECTIVE:**

To assess the effects of fremanezumab on response rates, acute headache medication use, and disability in EM patients who failed at least one prior preventive migraine medication.

**METHODS:**

In this Phase 3, multicenter, randomized, double-blind, placebo-controlled study, patients were randomized 1:1:1 to receive subcutaneous injections of fremanezumab quarterly (675 mg at baseline and placebo at Weeks 4 and 8), fremanezumab monthly (225 mg at baseline, Weeks 4 and 8), or placebo (at baseline, Weeks 4 and 8) over a 12-week treatment period. Analyses were performed in patients who failed at least one prior preventive migraine medication (due to lack of efficacy or intolerability) using Cochran–Mantel–Haenszel test or an analysis of covariance model. Endpoints included the proportion of patients with ≥50% reduction in the monthly average number of migraine days, mean change from baseline in the monthly average number of days of acute headache medication use, and mean change from baseline in the Migraine Disability Assessment (MIDAS) score during the 12-week treatment period.

**RESULTS:**

The subgroup who failed at least one prior migraine preventive therapy included 58 fremanezumab quarterly, 65 fremanezumab monthly, and 63 placebo patients. A greater proportion of patients who received fremanezumab had a ≥50% reduction in the monthly average number of migraine days during the treatment period (quarterly: 38%, *P*=0.0100; monthly: 43%, *P*=0.0010) compared with placebo (17%). Fremanezumab significantly reduced from baseline the monthly average number of days of any acute headache medication use during the treatment period (quarterly [least-squares mean change ± standard error]: –3.1±0.5 days; monthly: –3.4±0.5 days) compared with placebo (–1.1±0.5 days; both, *P*<0.0001). Fremanezumab significantly improved disability from baseline, based on the change in MIDAS score during the treatment period (quarterly: –24.5±3.7, *P*=0.0006; monthly: –26.8±3.7, *P*<0.0001) compared with placebo (–11.1±3.4).

**CONCLUSIONS:**

Among EM patients who failed at least one prior preventive migraine medication, fremanezumab treatment was efficacious, reduced acute headache medication use, and improved disability, with effect sizes greater than those seen in the overall trial population.

**TRIAL REGISTRATION**: ClinicalTrials.gov, NCT02629861

**ETHICS APPROVAL**

The study was approved by relevant independent ethics committees or institutional review boards, according to national or local regulations.

### O39 Impact of fremanezumab on response rates, migraine days, and acute medication use in patients with chronic migraine who have failed at least one prior migraine preventive medication

#### Stephen D. Silberstein^1^, Jessica Ailani^2^, Joshua M. Cohen^3^, Ronghua Yang^3^, Paul P. Yeung^3^, Verena Ramirez Campos^4^

##### ^1^Jefferson Headache Center, Thomas Jefferson University, Philadelphia, Pennsylvania, USA; ^2^Medstar Georgetown University Hospital, Washington, District of Columbia, USA; ^3^Teva Pharmaceuticals, Frazer, Pennsylvania, USA; ^4^Teva Pharmaceuticals, Buenos Aires, Argentina

**BACKGROUND:**

Fremanezumab, a fully humanized monoclonal antibody (IgG2a) that selectively targets calcitonin gene-related peptide (CGRP), is efficacious in preventing chronic migraine (CM), but its effectiveness in patients who have failed previous preventive medications is unknown.

**OBJECTIVE:**

To assess the effects of fremanezumab on response rates, migraine days and acute headache medication use in patients with CM who failed one prior preventive migraine medication.

**METHODS:**

In this Phase 3, multicenter, randomized, double-blind, placebo-controlled study, patients were randomized 1:1:1 to receive subcutaneous injections of fremanezumab quarterly (675 mg at baseline and placebo at Weeks 4 and 8), fremanezumab monthly (675 mg at baseline and 225 mg at Weeks 4 and 8), or placebo (at Baseline, Weeks 4 and 8) over a 12-week treatment period. Analyses were performed in patients who failed at least one prior preventive migraine medication (due to lack of efficacy or intolerability) using a Cochran-Mantel-Haenszel test or an analysis of covariance model. Endpoints included the proportion of patients with a ≥50% reduction in headache days of at least moderate severity, mean change from baseline in the monthly average number of migraine days and mean change from baseline in the number of days of acute headache medication use during the 12-week treatment period.

**RESULTS:**

The subgroup of patients who failed at least one prior preventive migraine therapy included 130 fremanezumab quarterly, 141 fremanezumab monthly, and 136 placebo patients. More patients who received fremanezumab experienced a ≥50% reduction in headache days of at least moderate severity (quarterly: 28%, *P*<0.0001; monthly: 38%, *P*<0.0001) during the treatment period than did those given placebo (8%). Fremanezumab treatment reduced the monthly average number of migraine days during the 12-week treatment period ([least-squares mean change ± standard error]: fremanezumab quarterly, –4.2±0.6 days, *P*=0.0050; fremanezumab monthly, –4.8±0.5 days, *P*<0.0001) compared with placebo (–2.4±0.6 days). Fremanezumab significantly reduced the monthly average number of days from baseline of any acute headache medication use during the treatment period (fremanezumab quarterly, –3.4±0.5 days; fremanezumab monthly, –4.1±0.5 days; both, *P*<0.0001) compared with placebo (–1.2±0.5 days).

**CONCLUSIONS**:

Among patients with CM who have failed at least one prior preventive migraine medication, fremanezumab was efficacious, with effect sizes in excess of those seen in the overall trial population. Fremanezumab offers clinical benefits to these potentially difficult-to-treat patients.

**TRIAL REGISTRATION**: ClinicalTrials.gov, NCT02621931

**ETHICS APPROVAL**

The study was approved by relevant independent ethics committees or institutional review boards, according to national or local regulations.

### O40 Efficacy of fremanezumab in migraine patients who have failed at least one prior migraine preventive medication

#### Peter McAllister^1^, David W. Dodick^2^, Joshua M. Cohen^3^, Ronghua Yang^3^, Paul P. Yeung^3^, Verena Ramirez Campos^4^

##### ^1^New England Institute for Neurology and Headache, Stamford, Connecticut, USA; ^2^Department of Neurology, Mayo Clinic, Phoenix, Arizona, USA; ^3^Teva Pharmaceuticals, Frazer, Pennsylvania, USA; ^4^Teva Pharmaceuticals, Buenos Aires, Argentina

###### **Correspondence:** Peter McAllister (jpotuzak@hcg-int.com)

**BACKGROUND:**

Fremanezumab, a fully humanized monoclonal antibody (IgG2a) that selectively targets calcitonin gene-related peptide (CGRP), has been shown to be effective in the prevention of chronic migraine (CM) and episodic migraine (EM).

**OBJECTIVE:**

To assess the efficacy of fremanezumab in migraine patients who failed at least one prior preventive migraine medication.

**METHODS:**

Fremanezumab was studied in two Phase 3, multicenter, randomized, double-blind, placebo-controlled, parallel-group trials. Patients with CM or EM (confirmed during a 28-day pre-treatment baseline period) received subcutaneous injections of fremanezumab quarterly (675 mg at baseline and placebo at Weeks 4 and 8), monthly (CM: 675 mg at baseline and 225 mg at Weeks 4 and 8; EM: 225 mg at baseline and Weeks 4 and 8), or placebo (at baseline and Weeks 4 and 8) over a 12-week treatment period, with a final evaluation 4 weeks after the last dose of the study drug. Mean changes from baseline in the monthly average number of headache days of at least moderate severity or the monthly average number of migraine days during the 12-week treatment period were assessed in patients who failed at least one prior migraine preventive medication due to lack of efficacy or intolerability. Analyses were performed in the intent-to-treat population using an analysis of covariance (ANCOVA) model.

**RESULTS:**

In CM patients, fremanezumab yielded greater reductions in the monthly average number of headache days of at least moderate severity (quarterly [n=130] [least-squares mean change ± standard error]: –4.0±0.47, *P*<0.0001; monthly [n=141]: –4.6±0.46, *P*<0.0001) compared with placebo (n=136; –1.9±0.49). There were similar reductions in the monthly average number of migraine days (quarterly: –4.2±0.55, *P*=0.005; monthly: –4.8±0.53, *P*<0.0001) compared with placebo (–2.4±0.56).

In EM patients, fremanezumab yielded greater reductions in the monthly average number of headache days of at least moderate severity (quarterly [n=58]: –3.0±0.51, *P*<0.0001; monthly [n=65]: –3.2±0.49, *P*<0.0001) compared with placebo (n=63; –0.7±0.47). There were similar reductions in the monthly average number of migraine days (quarterly: –3.3±0.61, *P*=0.0015; monthly: –3.8±0.59, *P*<0.0001) compared with placebo (–1.3±0.57).*P*-values stated are compared with placebo.

**CONCLUSIONS:**

Fremanezumab was efficacious in migraine patients who failed at least one prior migraine preventive medication, a potentially difficult-to-treat population. Effect sizes in this subgroup were greater than those in the overall trial population.

**TRIAL REGISTRATION**: ClinicalTrials.gov NCT02621931 and NCT02629861

**ETHICS APPROVAL**

The study was approved by all relevant independent ethics committees or institutional review boards, according to national or local regulations.

### O41 Cluster headache is not associated with sleep apnea or specific sleep stages

#### Nunu Lund^1^; Mads Barloese^2^; Agneta Snoer^1^; Anja Petersen^1^; Poul Jørgen Jennum^3^; Rigmor H. Jensen^1^

##### ^1^Danish Headache Center, Department of Neurology, Rigshospitalet Glostrup, University of Copenhagen, Denmark; ^2^Department of Clinical Physiology, Nuclear Medicine and PET, Rigshospitalet Glostrup, University of Copenhagen, Denmark; ^3^Danish Center for Sleep Medicine, Dept. of Neurophysiology, Rigshospitalet – Glostrup, University of Copenhagen, Denmark

###### **Correspondence:** Nunu Lund (nunu.lund@regionh.dk)

**Background and aim:** Cluster headache (CH) is characterized by severe, unilateral attacks of pain and by a high nocturnal attack burden. Sleep quality remains significantly lower in patients more than one year after the last CH attack compared with healthy controls. Further, sleep parameters are shown to be altered in active CH patients. The primary aim of this study was to compare the macrostructure of sleep in eCH patients in both disease phases and to compare eCH patients with controls.

**Method**: ECH patients, aged 18-65 years, diagnosed according to the International Classification of Headache Disorders 2^nd^edition, were admitted for gold standard polysomnography at the Danish Center for Sleep Medicine, preferably both in bout and remission. The macrostructure of sleep including arousals, breathing parameters, limb movements (LMs) and periodic limb movements (PLMs) were compared with 25 age-, sex- and BMI-matched healthy controls. The study was approved by the ethics committee of the Capital Region of Denmark (H-7-2014-020) and the protocol was published at clinical trials.gov (NCT03439722).

**Results**: There were no differences in any of the sleep parameters for patients in bout (n=32) compared with patients in remission (n=23). Less than half of the patients in bout (14 out of 32 patients, 43.8%) suffered from 21 attacks in total during the polysomnography. Attacks were not related to specific sleep stages (N1: 2/21 attacks, N2: 6/21 attacks, N3: 5/21 attacks, REM: 7/10 attacks).

eCH patients had longer latency (18.9 vs. 11.7 minutes, p<0.05) and lower efficiency (84.4 vs. 86.5, p<0.05) compared with controls, but fewer PLMs (0.67 vs. 1.30 hour^-1^, p<0.05). Finally, the sleep apnea index was similar in both groups (9.63 vs. 7.76 hour^-1^, p=0.7674).

**Conclusion:** This is the first study that systematically investigates eCH patients with full polysomnography in both bout and remission and the largest study comparing eCH patients with controls. The observed sleep disturbances were not associated with the bout but rather seem to be the manifestation of a persisting, underlying pathology. Finally, the prevalence of sleep apnea was comparable in all groups and attacks were not related to specific sleep stages.

### O42 Real-world preventative drug management of Chronic Migraine among Spanish Neurologists

#### D. García-Azorin^1^, S. Santos Lasaosa^2^, A. B. Gago-Veiga^3^, J. Viguera Romero^4^, A. L. Guerrero-Peral^1^, P. Pozo-Rosich^5,6^.

##### ^1^Headache Unit, Neurology Department. Hospital Universitario Clínico de Valladolid. Valladolid, Spain; ^2^Headache monographic consult, Hospital Clínico Universitario Lozano Blesa, Zaragoza, Spain; ^3^Headache consult, Neurology Department. Hospital Universitario de la Princesa, Madrid, Spain; ^4^Headache consult. Neuroscience unit. Hospital Universitario Virgen Macarena, Seville, Spain; ^1^Headache Unit, Neurology Department. Hospital Universitario Clínico de Valladolid. Valladolid, Spain; ^5^Headache Unit. Neurology Department. Hospital Universitari Vall d’Hebron, Barcelona, Spain; ^6^Headache Research Group. Vall d’Hebron Institut de Recerca (VHIR), Barcelona, Spain

###### **Correspondence:** D. García-Azorin (davilink@hotmail.com)

**Background:**

In migraine, the therapeutic preventive drug arsenal is varied. When prescribing both Guidelines and patient characteristics are taken into account. In Spain, the use of preventive therapies seems to be heterogeneous.

The objective of this study was to evaluate real-life clinical prescribing practice amongst neurologists in Spain

**Methods:**

Observational descriptive study done with a survey by Neurologists of the Spanish Neurological Society (SEN). Neurologists who participated were divided into Headache Specialists or not. The following data was collected: socio-demographic data,; preventive treatment and choices different migraine sub-types, and their personal perception of efficacy and tolerability to different drugs.

**Results:**

We analyzed 152 surveys from neurologists around our country. From them: 43.4% were female, 53.3% <40 years, and 34.9% were interested in headache .

In regards to preventive treatment choice; in chronic migraine topiramate (57%) amytriptiline (17.9%) and beta-blockers (14.6%), whereas in episodic migraine the preferred drugs were beta-blockers (47.7%), topiramate (21.5%) and amytriptiline (13.4%).

Regarding perceived efficacy, topiramate was considered the best option in chronic migraine (42.7%) followed by onabotulinumtoxinA (25.5%) and amitryptiline (22.4%). In episodic migraine, neurologist preferred topiramate (43.7%) and beta-blockers (30.3%).

Regarding the duration of preventive therapy when improvement was achieved, when treating episodic migraine 43.5% of the surveyed neurologists recommended 3 months and 39.5% preferred 6 months. When they treated chronic migraine, 20.4% of neurologists recommended 3 months, 42.1% 6 months, 12.5% 9 months and 22.4% preferred to maintain treatment during 12 months.

When considering onabotulinumtoxinA treatment, the number of prior therapeutical failures was cero in 7.2% of surveyed, one in 5.9%, two in 44.1%, three in 30.9%, and four or more in 11.9%. The increase of OnabotulinumtoxinA dose up to 195 UI was considered by 51% of neurologists after a first ineffective procedure, by 42.2% after two injections, and by 83% after a third infiltration. Surveyed colleagues admitted to take into account in their decisions mainly patient comorbidities (70.2%) rather than guidelines (13.9%).

**Conclusions:**

Initial management of Migraine among Spanish Neurologists is made with the preventative drugs which are considered as first choices in most of the guidelines. Management of episodic migraine differed from chronic migraine, both in the order or drugs and the perception of the most effective therapy.

### O43 Effect of the H_1_-antihistamine clemastine on PACAP38 induced migraine

Luise Haulund Vollesen^1^, Song Guo^1^, Malene Rohr Andersen^2^, Messoud Ashina^1^

^1^Danish Headache Centre and Department of Neurology, Rigshospitalet Glostrup, Faculty of Health and Medical Sciences, University of Copenhagen, Denmark; ^2^Department of Clinical Biochemistry, Herlev and Gentofte Hospital, Gentofte, Denmark

**Correspondence:** Messoud Ashina (ashina@dadlnet.dk)

**Objective** To investigate the effect of the H_1_-antihistamine clemastine on the migraine inducing abilities of pituitary adenylate cyclase activating peptide-38 (PACAP38).

**Methods** We conducted a double-blind, randomized, placebo controlled two-way cross-over study. Twenty migraine without aura patients were randomly allocated to receive bolus clemastine 2 mg (1 mg/ml) or bolus saline 2 ml intravenously over 2 min on two study days. Following each bolus injection 10 pmol/kg/min of PACAP38 was administered intravenously over 20 min. We recorded migraine/headache characteristics every 10 min until 90 min after infusion start and collected blood to investigate mast cell degranulation and the inflammation markers tryptase and tumor necrosis factor-alpha (TNF-alpha) before and after infusion of PACAP38.

**Results** After clemastine pretreatment 5/20 participants developed a migraine-like attack in response to a PACAP38 infusion compared to 9/20 after placebo pretreatment (P=0.288). Following clemastine pretreatment 15/20 participants reported headache in response to a PACAP38 infusion, whereas 19/20 participants did so following placebo pretreatment (P=0.221). We found no difference in area under the curve 12 h (AUC_12h_) for headache intensity between the two experimental days (P=0.481). We found no difference in AUC_180min_ for tryptase (P = 0.525) or TNF-alpha (P = 0.487) between clemastine and placebo pretreatment days.

**Conclusion** H1-antihistamine, clemastine, failed to prevent migraine or headache after PACAP38 infusion thus making a role for histamine release or mast cell degranulation in PACAP38 induced migraine less likely.

### O44 CSF pressure fluctuations as a marker of isolated CSF hypertension in headache sufferers

#### L. Rapisarda^1,2^, G. Demonte^1^, F. Tosto^1^, M. Curcio^1^, B. Vescio^5^, C. Bombardieri ^3^, D. Mangialavori^4^, U. Aguglia^2^, A. Gambardella^2^, F. Bono^1,2^

##### ^1^Headache Center, and Institutes of Neurology^2^, Neuroradiology^3^, Ophthalmology^4^, Department of Medical and Surgical Sciences, Magna Græcia University of Catanzaro, Italy*;*^5^Neuroimaging Research Unit, Institute of Bioimaging and Molecular Physiology, National Research Council, Catanzaro, Italy

###### **Correspondence:** G. Demonte (g.dem.1990@gmail.com)

**Background**

It is needed to identify the characteristics and the pressure-related features of isolated cerebrospinal fluid hypertension for differentiating headache sufferers with isolated cerebrospinal fluid hypertension from those with primary headache disorder.

**Patients and Methods**

In this prospective study patients with refractory chronic headaches suspected of having cerebrospinal fluid-pressure elevation without papilledema or sixth nerve palsy and non-headache controls underwent 1-hour lumbar cerebrospinal fluid pressure monitoring via a spinal puncture needle.

**Results**

We recruited 148 consecutive headache patients and 16 non-headache controls. Lumbar cerebrospinal fluid pressure monitoring showed high pressure and abnormal pressure fluctuations in 93 (63 %) patients with headache: 37 of these patients with the most abnormal pressure parameters (opening pressure above 250 mm H_2_O, mean pressure 301 mm H_2_O, mean peak pressure 398 mm H_2_O, and severe abnormal pressure fluctuations) had the most severe headaches and associated symptoms (nocturnal headache, postural headache, transient visual obscuration); 56 patients with the less abnormal pressure parameters (opening pressure between 200 and 250 mm H_2_O, mean pressure 228 mm H_2_O, mean peak pressure 316 mm H_2_O, and abnormal pressure fluctuations) had less severe headaches and associated symptoms.

**Conclusions**

Nocturnal headache and postural headache are the most common characteristics of isolated cerebrospinal fluid hypertension. Abnormal pressure fluctuations are associated with symptomatic high cerebrospinal fluid pressure, and they differentiate headache sufferers with isolated cerebrospinal fluid hypertension from those with primary headache disorder.

### O45 The practical and clinical utility of non-invasive vagus nerve stimulation (nVNS) as an acute treatment for episodic migraine: a post hoc analysis of the randomised sham-controlled PRESTO trial

#### Licia Grazzi^1^; Cristina Tassorelli^2,3^; Marina de Tommaso^4^; Giulia Pierangeli^5^; Paolo Martelletti^6^; Innocenzo Rainero^7^; Pierangelo Geppetti^8^; Anna Ambrosini^9^; Paola Sarchielli^10^; Eric Liebler^11^; Piero Barbanti^12^

##### ^1^Headache Center, Carlo Besta Neurological Institute and Foundation, Milano, Italy; ^2^Headache Science Centre, Istituto di Ricovero e Cura a Carattere Scientifico (IRCCS) C. Mondino Foundation, Pavia, Italy; ^3^University of Pavia, Pavia, Italy; ^4^Neurophysiology and Pain Unit, University of Bari Aldo Moro, Bari, Italy; ^5^IRCCS Istituto delle Scienze Neurologiche di Bologna, Bologna, Italy; ^6^Department of Clinical and Molecular Medicine, Sapienza University, Rome, Italy; ^7^Department of Neuroscience, University of Turin, Turin, Italy; ^8^Headache Centre, University Hospital of Careggi, Florence, Italy; ^9^IRCCS Neuromed, Pozzilli (IS), Italy; ^10^Neurologic Clinic, Santa Maria della Misericordia Hospital, Perugia, Italy; ^11^electroCore, LLC, Basking Ridge, New Jersey, USA; ^12^Headache and Pain Unit, IRCCS San Raffaele Pisana, Rome, Italy

###### **Correspondence:** Licia Grazzi (licia.grazzi@istituto-besta.it)

**Background:** The multicentre, double-blind, randomised, sham-controlled PRESTO trial provided Class I evidence that for patients with an episodic migraine, non-invasive vagus nerve stimulation (nVNS; gammaCore®) significantly increases the probability of having mild pain or being pain-free 2 hours post‑stimulation [1]. Here, we aimed to reveal further insights into the practical and clinical utility of nVNS in PRESTO by evaluating the ability of this therapy to provide clinically meaningful reductions in pain while reducing the need for rescue medication.

**Methods:** The PRESTO study consisted of a 4‑week run-in period (individualised treatment regimens), a 4-week double-blind period of randomly assigned nVNS or sham treatment, and a 4-week open-label period wherein all patients received nVNS. In this post hoc analysis of PRESTO, the percentage of patients with a ≥1-point reduction in pain score (0, *no pain*; 1, *mild pain*; 2, *moderate pain*; 3, *severe pain*) for their first treated attack and the percentage of all treated attacks achieving this reduction were evaluated. Rescue medication use at any time point was also assessed for both the first attack and all attacks.

**Results:** The percentage of patients with a ≥1‑point decrease in pain was significantly higher with nVNS (n=120) than with sham (n=123) for the first attack at 30 minutes (nVNS, 32.2%; sham, 18.5%; *p*=0.020), 60 minutes (nVNS, 38.8%; sham, 24.0%; *p*=0.017), and 120 minutes (nVNS, 46.8%; sham, 26.2%; *p*=0.002). Results were similar when evaluating this end point as a percentage of all attacks. Rescue medication use was significantly lower in the nVNS group than in the sham group for both the first attack (nVNS, 40.7%; sham, 58.2%; *p*=0.013) and all attacks (nVNS, 47.7%; sham, 62.7%; *p*=0.008).

**Conclusions:** Findings from this post hoc analysis reinforce the practical and clinical utility of nVNS for episodic migraine. Patients using nVNS acutely benefited from consistent ≥1-point pain reductions and a decreased need for rescue medication. The flexibility of nVNS offers the ability to treat multiple attacks without increasing exposure to acute medications and pharmacologic adverse events.

**Acknowledgements**

PRESTO was sponsored by electroCore, LLC. We present this abstract on behalf of the PRESTO Study Group.

**Trial registration:** NCT02686034

**Ethics approval**

PRESTO was approved by the local ethics committee for each study site.

**Reference**

1. Tassorelli C, Grazzi L, de Tommaso M, et al. Non-invasive vagus nerve stimulation as acute therapy for migraine: the randomized PRESTO study. *Neurology*. In press.

**Author Disclosures:**

**Dr. Grazzi** has received consultancy and advisory fees from Allergan S.p.A. and electroCore, LLC.

**Prof. Tassorelli** has received consultancy fees from Allergan S.p.A.; electroCore, LLC; Eli Lilly and Company; and Novartis AG and research grants from the European Commission and the Italian Ministry of Health. She is also a principal investigator or collaborator for RCTs sponsored by Alder BioPharmaceuticals Inc.; Eli Lilly and Company; and Teva Pharmaceutical Industries Ltd.

**Prof. de Tommaso** has received advisory fees from Allergan S.p.A.; Neopharmed; and Pfizer Inc.

**Dr. Pierangeli** has nothing to disclose.

**Prof. Martelletti** has received research grants, advisory board fees, or travel fees from ACRAF; Allergan S.p.A.; Amgen Inc.; electroCore, LLC; Novartis AG; and Teva Pharmaceutical Industries Ltd.

**Prof. Rainero** has received consultancy fees from electroCore, LLC, and Mylan N.V. and research grants from the European Commission -- Horizon 2020. He is also a principal investigator for RCTs sponsored by Axovant Sciences Ltd. and TauRx Pharmaceuticals Ltd.

**Prof. Geppetti** has received consultancy fees from Allergan S.p.A.; electroCore, LLC; Evidera; Novartis AG; Pfizer Inc.; and Sanofi S.p.A. and research grants from Chiesi Farmaceutici S.p.A. He is also a principal investigator for RCTs sponsored by Eli Lilly and Company; Novartis AG; and Teva Pharmaceutical Industries Ltd.

**Dr. Ambrosini** has received consultancy fees from Almirall, S.A., and travel grants from Allergan S.p.A. and Almirall, S.A.

**Prof. Sarchielli** has received clinical study fees from Allergan S.p.A.

**Mr. Liebler** is an employee of electroCore, LLC, and receives stock ownership.

**Prof. Barbanti** has received consultancy fees from Allergan S.p.A.; electroCore, LLC; Janssen Pharmaceuticals, Inc.; Lusofarmaco; and Visufarma and advisory fees from Abbott Laboratories and Merck & Co., Inc.

## SISC Oral Presentation

### O46 Headache as presenting symptom of neurosarcoidosis

#### C. Lisotto^1^, L. Toma^2^, E. Mampreso^3^, G. Zanchin^4^

##### ^1^Headache Centre, Department of Neurology, Pordenone, Italy, ^2^Department of Neurology, Ferrara, Italy, ^3^Headache Centre, Department of Neurology, Piove di Sacco, Italy ^4^Headache Centre, Department of Neurosciences, Padua, Italy

###### **Correspondence:** C. Lisotto (carlo.lisotto@aas5.sanita.fvg.it)

**Objectives** Sarcoidosis is a multi-organ granulomatous disease of unknown aetiology, characterized pathologically by multiple non-caseating granulomata in the absence of a defined infective or toxic trigger. Sarcoidosis involving the nervous system (the so-called neurosarcoidosis) is infrequent and headache may be the presenting symptom [1]. The diagnosis of headache attributed to neurosarcoidosis is challenging and requires particular attention from headache specialists.

**Materials and methods** The medical records of patients admitted in the past 15 years to our Department of Neurology for recent-onset headache with a final diagnosis of neurosarcoidosis were retrospectively reviewed. The diagnosis of headache attributed to neurosarcoidosis was made according to the International Classification of Headache Disorders, 3^rd^ edition (ICHD-3) [2].

**Results** Four patients, two males and two females, mean age at observation 40 years (range 31-49), were included in our review. In all the subjects headache was the onset symptom, occurred acutely or subacutely. Two patients (one male and one female) reported headache as the only symptom; the pain was diffuse, severe, non-pulsating, with daily or nearly-daily occurrence, mimicking tension-type headache. In the remaining two cases the headache was unilateral, periorbital, intense and sharp, associated with third cranial nerve paralysis, resembling Tolosa-Hunt syndrome. All the patients underwent brain magnetic resonance imaging (MRI) and cerebrospinal fluid (CSF) examination. MRI showed non-enhancing periventricular white matter lesions, enhancement of the leptomeninges with predilection for suprasellar and frontal basal meninges and involvement of cavernous sinus ipsilateral to pain in the patients with third cranial nerve palsy. CSF examination revealed lymphocytic pleocytosis and elevated protein. All the patients were treated with oral prednisone, starting from 1 mg/kg daily, with rather rapid clinical improvement. In three patients chest radiography was abnormal, showing bihilar lymphadenopathy; on bronchoalveolar lavage CD4:CD8 lymphocyte ratio was more than 3:5:1.

**Discussion** The headache in our patients was clinically similar to tension-type headache in two cases and to Tolosa-Hunt syndrome in the other two. ICHD-3 criteria for headache attributed to neurosarcoidosis imply that the clinical features of this secondary headache have a wide range of presentations. As for differential diagnosis versus Tolosa-Hunt syndrome, our patients obtained a remission within 10-14 days, a longer time than 72 hours for pain and paresis resolution, as required by ICHD-3 diagnostic criteria for this syndrome.

**Conclusions** Headache may rarely herald the diagnosis of neurosarcoidosis. New case series continue to broaden the phenotype of neurosarcoidosis, reinforcing the need for a systematic approach to diagnosis and management.

**References**

1. Radwan W, Lucke-Wold B, Robadi IA, Gyure K, Roberts T, Bhatia S. Neurosarcoidosis: unusual presentations and consideration for diagnosis and management. Postgrad Med J 2017;93:401-5.

2. Headache Classification Committee of the International Headache Society (IHS). The International Classification of Headache Disorders, 3rd edition. Cephalalgia 2018;38:1-211.

3. Ibitoye RT, Wilkins A, Scolding NJ. Neurosarcoidosis: a clinical approach to diagnosis and management. J Neurol 2017;264:1023-8.

### O47 Association of Tanacethum Parthenium, 5 - hydroxy tryptophan and magnesium ( Aurastop) versus Mg tablet impact on aura phenomena and its evolution : an observational study

#### Lidia Savi (lsavi@cittadellasalute.to.it)

##### Headache Center LBS, Lugano, Switzerland

**BACKGROUND:**

A new phytotherapic combination of Tanacethum Parthenium (150 mg), 5 - hydroxy tryptophan (20 mg) and magnesium (185 mg) (Aurastop ®) is now available for migraneous patients. The three components act on the four main mechanisms involved in the pathophysiology of migraine with aura: cortical Spreading Depression, sensitization of trigeminal vascular system, central sensitization and activation of “migraine generator” at the brainstem’s level. Since many years Magnesium is well known to interact with the aura phenomena and migraine itself.With this study we want to compare the efficacy on the aura phenomenon and its disability of the combination of Tanacethum Parthenium, 5 - hydroxy tryptophan and magnesium versus magnesium alone, when taken at the beginning of the aura.

**MATERIALS AND METHODS:** We selected from the Headache Center LBS of Lugano (CH) a population of 60 patients aged from 18 to 60 years (mean 32 years ), 31 women and 29 men, suffering from migraine with aura, not assuming migraine preventive therapy. They have to refer of an aura with a duration of at least 20 minutes to be included. We gave to the patients a form where they have to describe the aura features of the 4 aura episodes following the administration of 1 tablet of Aurastop at the beginning of the aura and 1 tablet at the beginning of the headache ( if present ) in the first 2 aura and 1 tablet of magnsium 2,25 gr in the same modality at the 3° and 4° episodes of aura. Patients were evaluated for duration and disability of the aura, need and response to their habitual analgesic drug

**RESULTS:** A reduction in duration greater than 50% in 54 patients versus 6 and of disability >50% in 52 patients against 8 were observed respectively after taking Aurastop or Magnesium alone

.Furthermore 30% of the aurastop group did not have to take pain reliever after the aura as the headache intensity was more tolerable, only 5% of the magnesium group. We also noted a marked improvement in the benefit of the usual pain killer in 30 patients that used aurastop .

**CONCLUSIONS:** By the fact that this combination pass very quickly the ematoencephalic barrier Aurastop has been shown to have a quick impact on the evolution of aura reducing the duration and disability of symptoms than the magnesium alone.

### O48 Hemicrania continua-like headache revealed a subacute internal carotid artery dissection in patient with unrecognized connective tissue disorder

#### Silvia Ricci, Gaetano Salomone, Francesca Rossi, Alberto Polo

##### Neurology department- Mater Salutis Hospital- ULSS Scaligera, Verona, Italy

###### **Correspondence:** Silvia Ricci (silv.ricci@yahoo.it)

**Background** It is widely accepted that internal carotid (ICA) dissection could simulate a cluster headache attack. Conversely, clinical features resembling hemicrania continua (HC) occurring after cervical artery dissection have rarely been reported in the literature. We described the case of a patient who developed typical HC-like headache after carotid artery dissection.

**Case-presentation** On February 2018, a 43 year-old man presented to our emergency department because of the onset, four days before, of severe continuos right trigeminoautonomic cephalgia with Horner’s syndrome and elevated blood pressure. His familiar and personal medical history were unremarkable except for the cluster headache involving the left side few years ago. Neurological examination revealed acute headache fulfilling all IHS criteria for HC (apart from the time criterion) unresponsive to habitual medication for migraine associated with right tongue deviation. The routine laboratory test, CT brain scan and ultrasound examination of neck vessels were normal. Indomethacin 200mg i.v improved the headache but we decided to admit the patient to our department because of the continuity of pain. Over the next two days there was complete relief with oral indomethacin 200 mg per day and an improvement of the Horner’s syndrome, nevertheless the patient developed progressive dysphagia, dysphonia, and weak left-turning of the head suggesting 9th through 12th cranial nerve palsy. He underwent brain magnetic resonance imaging (MRI) with MR angiography of head and neck that showed a right ICA dissection with extension into the petrous segment and intramural hematoma causing mass effect upon the internal jugular vein; no hyperintensity was found in DWI sequences . The patient was started on acetylsalicylic acid 100 mg daily. An extended CT angiography showed extensive luminal irregularities in the main renal arteries, with aneurysm formations and irregularities of iliac vessels. Due to his new diagnosis of arterial hypertension and the other findings we assumed the possibility of a connective tissue disorder and we performed a genetic counseling with test for Ehlers-Danlos syndrome variants. So far, the results received were negative but other test are still ongoing and fibromuscolar dysplasia (FMD) is strongly considered.

**Conclusion** ICA dissection may result in an HC-like headache syndrome. The history of cluster headache, a specific response to indomethacin and the absence of neurological focal signs does not rule out dissection as underlying pathology. Screening for connective tissue disorder and extracranial manifestations of FMD should be considered even if the brain vasculature is normal.

**Consent for publication:** Informed consent was obtained from patient for publication.

**References**

1- Ashkenazi A, Abbas MA, Sharma DK, et al. Hemicrania continua-like headache associated with internal carotid artery dissection may respond to indomethacin. Headache 2007; 47: 127–130.

2- Headache Classification Committee of the International Headache Society (IHS). The International Classification of Headache Disorders, 3rd edition (beta version). Cephalalgia 2013; 33: 629–808.

3- O’Connor S, Poria N and Gornik H. Fibromuscular dysplasia: An update for the headache clinician. Headache 2015; 55: 748–755

### O49 Neurophysiological correlates of clinical improvement after Greater Occipital Nerve (GON) Block in Chronic Migraine: relevance for chronic migraine pathophysiology

#### Alessandro Viganò^1,2^, Maria Claudia Torrieri^3^, Massimiliano Toscano^1,4^, Francesca Puledda^5^, Barbara Petolicchio^1^, Tullia Sasso D’Elia^2^, Angela Verzina^6^, Sonia Ruggiero^1^, Marta Altieri^1^, Edoardo Vicenzini^1^, Jean Schoenen^7^, Vittorio Di Piero^1,8^

##### ^1^Headache Centre & Neurocritical Care Unit. Department of Human Neurosciences, Sapienza – University of Rome, Rome, Italy; ^2^Molecular and Cellular Networks Lab. Department of Anatomy, Histology, Forensic medicine and Orthopaedics, Sapienza – University of Rome, Rome, Italy; ^3^Rita Levi Montalcini Department of Neuroscience, Città della Salute e della Scienza, Turin, Italy; ^4^Department of Neurology – Fatebenefratelli Hospital – Rome, Italy; ^5^Headache Group, Department of Basic and Clinical Neuroscience, King's College London, and NIHR-Wellcome Trust King's Clinical Research Facility, Wellcome Foundation Building, King's College Hospital, London, SE5 9PJ, UK; ^6^Department of Neurology, University of Perugia, Perugia, Italy; ^7^Headache Research Unit. Department of Neurology, University of Liège, Citadelle Hospital, Liège, Belgium; ^8^University Consortium for Adaptive Disorders and Head pain – UCADH, Pavia, Italy

###### **Correspondence:** Alessandro Viganò (alessandro.vigano@uniroma1.it)

**Background:**

Therapeutic management of Chronic Migraine (CM), often associated with Medication Overuse Headache (MOH), is chiefly empirical, as no biomarker predicting or correlating with clinical efficacy is available to address therapeutic choices. The present study searched for neurophysiological correlates of Greater Occipital Nerve Block (GON-B) effects in CM.

**Results:**

We recruited 17 CM women, of whom 12 with MOH, and 19 female healthy volunteers (HV). Patients had no preventive treatment since at least 3 months. After a 30-day baseline, they received a bilateral betamethasone-lidocaine GON-B of which the therapeutic effect was assessed 1 month later. Habituation of visual evoked potentials (VEP) and intensity dependence of auditory evoked potentials (IDAP) were recorded before and 1 week after the GON-B. At baseline, CM patients had normal VEP habituation, but a steeper IDAP value than HV (p=0.009), suggestive of a lower serotonergic tone. GON-B significantly reduced the number of total headache days per month (-34.9%; p=0.003). Eight out 17CM patients reversed to episodic migraine and medication overuse resolved in 11 out of 12 patients. One week after the GON-B VEP habituation tended to be reduced (p=0.09) and became inferior to that of HV (p=0.03) like in episodic migraine, while the IDAP slope significantly flattened (p=0.008). GON-B-induced reduction in headache days positively correlated with IDAP slope decrease (rho=0.51, p=0.03).

**Conclusions:**

GON-B may be effective in the treatment of CM, with or without MOH. The pre-treatment IDAP increase is compatible with a weak central serotonergic tone, which is strengthened after GON-B, suggesting that serotonergic mechanisms may play a role in CM and its reversion to episodic migraine. Since the degree of posttreatment IDAP decrease is correlated with clinical improvement, IDAP might be potentially useful as an early predictor of GON-B efficacy.

### O50 Short-Term Psychodynamic Psychotherapy versus OnabotulinumtoxinA as preventive therapy in Chronic Migraine: a real world study

#### M. Alessiani^1^, B. Petolicchio^1,2^, A. Viganò^1^, R. Di Giambattista^3^, M. Altieri^1^, E. Gilliéron^3^,V. Di Piero^1,2^

##### ^1^Department of Human Neuroscience, Sapienza University of Rome, 00185 - Rome, Italy; ^2^ “Enzo Borzomati” Pain Medicine Unit – University Hospital Policlinico Umberto I, 00161-Rome, Italy; ^3^ Istituto Europeo di Psicoterapia Psicoanalitica (IREP), 00187 - Rome, Italy

###### **Correspondence:** B. Petolicchio (barbara.petolicchio@uniroma1.it)

**Background**

The preventive treatment for chronic migraine (CM) is difficult and often complicated by analgesics overuse and poor compliance.

Previously, we showed that short-term psychodynamic psychotherapy (STPP), alone or with pharmacological therapies, improved the clinical outcome, the analgesics overuse withdrawal and reduces long-term relapse rate of CM [1,2].

Since OnabotulinumtoxinA (BoNT-A) is one of the most effective options for CM [3,4], we investigated the effect of STPP versus BoNT-A as preventive treatment in a real world CM population, with and without medication overuse headache (MOH).

**Results**

We consecutively recruited, CM patients who underwent STPP or BoNT-A treatment according to clinical judgment of the attending headache specialist.

STPP consists of 4 exploratory meetings, followed by 8 meetings of freudian-inspired psychotherapy [5]. At the end of the STPP (90 days), if appropriate, a pharmacological therapy was added. BoNT-A was administered according to PREEMPT protocol [3,4]. No additional pharmacological therapies were allowed.

At 90, 180 and 270 days, evaluations were made on the treatment effectiveness by an investigator blinded of the assigned treatment. Ninthy-eight patients with CM (64% with MOH) were treated with STPP and 54 (59% with MOH) with BoNT-A. At baseline, BoNT-A patients had a significant (p <0.001) higher attack rates, more failed preventive therapies, more years of illness and chronicity, and were older.

The first appropriate follow-up to evaluate STPP efficacy is at 90d whereas for BoNT-A is after 180d. At these times, the episodic pattern remission rate was 53% (52/98) for STPP and 33% (18/54) for BoNT-A treatment. A pharmacological therapy was added in 27 patients of the STPP group.

With respect to baseline, at 270d STPP and BoNT-A groups showed a significant reduction of headache days (-14,9±0,3 vs –10,8±3,3) and analgesics intake (-12,2±10,3 vs –11,6±13,4, pills/month), respectively (Figs.1 and 2). In both groups, a high headache frequency at baseline (>25 days/month) was a significant negative prognostic factor for remission to an episodic pattern (p<0.05). Dropout rate was lower in BoNT-A group than STPP one (11% vs. 29%, p<0.05).

**Conclusion**

In the real world, both treatments with STPP, alone or combined with drug therapy, and BoNT-A were effective for treating patients with CM, with or without MOH. Treatment with BoNT-A is the physician preferred treatment for patients with more severe CM. Furthermore, the effectiveness of the STPP occurs earlier than BoNT-A but with a higher dropout rate.

**Ethics approval**

The study was approved by Policlinico Umberto I Ethical Board N°4604.

**Reference**

1. Altieri M, Di Giambattista R, Di Clemente L, Fagiolo D, Tarolla E, Mercurio A, Vicentini E, Tarsitani L, Lenzi GL, Biondi M, Di Piero V. Combined pharmacological and short term psychotherapy for probabile medication overuse headache: a pilot study. Cephalalgia 2009;29(3): 29-39

2. Petolicchio B, Viganò A, Di Giambattista R, Squitieri M, Zanoletti N, Tortora D’Amato P, Spensierato A, Baldassarre M, Di Piero V. Short-term psychodynamic psychotherapy versus pharmacological treatment in chronic headache: an observational study. J Headache Pain 2013; 14 (S13-S41): 31

3. Aurora SK, Dodick DW, Turkel CC, et al. OnabotulinumtoxinA for treatment of chronic migraine: results from the double-blind, randomized, placebo-controlled phase of the PREEMPT 1 trial. Cephalalgia. 2010;30(7):793–803. 57.

4. Diener HC, Dodick DW, Aurora SK, et al. OnabotulinumtoxinA for treatment of chronic migraine: results from the double-blind, randomized, placebo-controlled phase of the PREEMPT 2 trial. Cephalalgia. 2010;30(7):804–814

5. Gilliéron E. Setting and motivation in brief psychotherapy. PsychotherPsychosom 1987; 47:105–12

**Fig. 1 (abstract O50).**
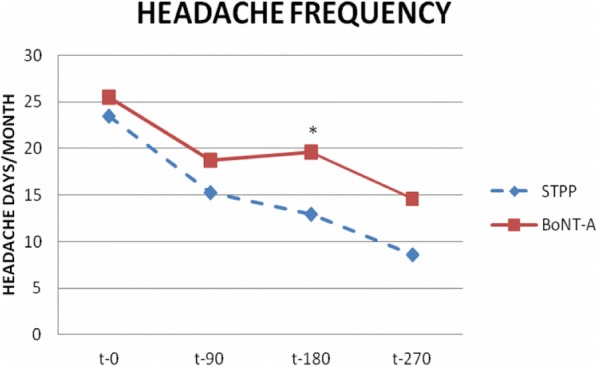
Headache days per month analgesics (p<0.001)

**Fig. 2 (abstract O50).**
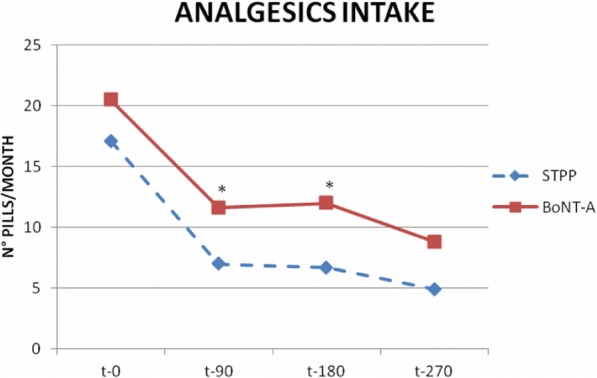
Monthly intake of (p<0.01)

### O51 Resting-state between-networks functional connectivity is abnormal in chronic migraine patients

#### Gianluca Coppola^1*^, Barbara Petolicchio^2^, Antonio Di Renzo^1^, Emanuele Tinelli^2^, Cherubino Di Lorenzo^3^, Vincenzo Parisi^1^, Mariano Serrao^4^, Valentina Calistri^2^, Stefano Tardioli^2^, Gaia Cartocci^2^, Francesca Caramia^2^, Vittorio Di Piero^2^, and Francesco Pierelli^4,5^

##### ^1^G.B. Bietti Foundation IRCCS, Research Unit of Neurophysiology of Vision and Neurophthalmology, Rome, Italy; ^2^Sapienza University of Rome, Department of Neurology and Psychiatry, Rome, Italy; ^3^Don Carlo Gnocchi Onlus Foundation, Milan, Italy; ^4^Sapienza University of Rome Polo Pontino, Department of Medico-Surgical Sciences and Biotechnologies, Latina, Italy; ^5^IRCCS-Neuromed, Pozzilli (IS), Italy

###### **Correspondence:** Gianluca Coppola (gianluca.coppola@gmail.com)

**Background**

The functional connectivity (FC) between default mode network (DMN), the executive control network (ECN), and the dorsal/ventral attention systems was found to be abnormal using resting state functional magnetic resonance imaging (RS-fMRI) in episodic migraine depending on migraine phase (ictal/interictal) and the frequency of the attacks. Here, we investigated RS between networks connectivity using independent component analysis (ICA) in chronic migraine (CM) patients.

**Materials and methods**

Twenty patients with untreated de-novo chronic migraine (CM) underwent 3T MRI scans and were compared to a group of 20 healthy controls (HC). We used MRI to collect RS data among three selected resting state networks, identified using group ICA: the DMN, the ECN, and the dorsal attention system (DAS).

**Results**

Compared to HCs, CM patients showed significant reduced functional connectivity between the DMN and the ECN. Moreover, in patients, the DAS showed significant stronger FC with the DMN and weaker FC with the ECN. The severity of headache attacks was correlated positively with the strength of DAS connectivity, and negatively with the strength of ECN connectivity.

**Conclusions**

These results suggest that the brain of CM patients is characterized by a large-scale reorganization at the level of the functional networks. Our data further suggest that the severity of migraine pain is associated with proportional inverse pattern of frontal executive and dorsal attentive networks connectivity.

### O52 New daily persistent headache in a pediatric cohort

#### Laura Papetti, Barbara Battan, Romina Moavero, Giorgia Sforza, Samuela Tarantino, Massimiliano Valeriani

##### Headache Center Bambino Gesù Children Hospital, Rome Italy

###### **Correspondence:** Laura Papetti (laura.papetti@opbg.net)

**Introduction**

Primary new daily persistent headache (NDPH) is a rare disorder of children and adults defined by the onset of daily headaches with distinct and clearly-remembered onset, with pain becoming continuous and unremitting within 24 hours and present for >3 months. The pain lacks characteristic features, and may be migraine-like or tension-type-like, or have elements of both. Our aim was to investigate the clinical features of NDPH in a cohort of pediatric patients.

**Methods**

We retrospectively reviewed the charts of patients attending the Headache Centre of Bambino Gesú Children from the last ten years with history of persistent daily headache. The ICHD-III criteria were used for diagnosis. Statistical analysis was conducted by SPPS version 22.0 and χ2 test was used to study possible correlations between: - NDPH and population features (age and sex); - NDPH and headache qualitative features; - NDPH and response to prophylactic therapies.

**Results**

We included 377 patients with CPH (66.4% female, 33.6% male, age between 0 and 18 years). The frequency of NADPH was 13% (49/377). We did not find significant differences between the frequency of NADPH in males (42.9%) and females (57.1%). In relation to age we found that NDAPH is less common in the age group of 7-10 years (p<0.05).

Regarding the features of the pain we did not find significant differences compared to the other forms of chronic headache for the quality of pain (throbbing or gravating), and the presence of photophobia (59.2% vs 60.7%, p>0.05) and phonophobia (63.3% vs 70.1%, p>0.05). However we found a low frequency of nausea and vomiting in the NADPH population (28.6% vs 48.2%, p<0.05).

We found that 75% of patients have an onset of the symptoms in the winter months (November-February), respect the remaining months of the year when the incidence is very low (p<0.05).

Our results show that 29 (30.6%) out of 49 NADPH CPH received a prophylactic therapy. Among them, 26 patients received amitriptyline, 4 patients topiramate, one patient L-5 hydroxytryptophan, and one patient flunarizine. Positive response to therapy (reduction of attacks by at least 50% in a month) was detected in 30.6% of patients, while no outcome data were obtained from 63.3% of cases. Amitriptyline showed the highest efficacy (p<0.05).

**Conclusions**

Our results show that the incidence of NASDH in children with daily headache is 13%. In general, the onset occurs in the winter months and this is probably related to the increase in requests for school activities. Qualitative characteristics as for adults are variable, migrainous or tension type. The most effective drug is amitriptyline, although the number of patients who received other types of drugs is very low Furthermore, the number of patients for whom there is an absence of follow-up data is very high and for this reason the efficacy data are not conclusive.

### O53 Clinical report of nineteen Italian patients with nummular headache

#### Lanfranco Pellesi^1^, Silvia Benemei^2^, Sabina Cevoli^3^, Valentina Favoni^3^, Chiara Lupi^2^, Edoardo Mampreso^4^, Antonio Russo^5^, Simona Guerzoni^1^

##### ^1^Medical Toxicology, Headache and Drug Abuse Centre, University of Modena e Reggio Emilia, Modena, Italy; ^2^Headache Centre, Careggi University Hospital, Department of Health Sciences, University of Florence, Florence, Italy; ^3^IRCCS Institute of Neurological Sciences of Bologna, Bologna, Italy; ^4^Headache Centre, Headache Centre, Neurology - Euganea, Padova Health Unit, Padua, Italy; ^5^Headache Centre, Department of Medical, Surgical, Neurological, Metabolic and Ageing Sciences, University of Campania “Luigi Vanvitelli”, Naples, Italy

###### **Correspondence:** Lanfranco Pellesi (lanfranco.pellesi@gmail.com)

**Background**

Nummular headache, also known as coin-shaped headache, is a rare headache disorder described by a small circumscribed painful area of the scalp. In the last decades, the description of numerous case reports has motivated its inclusion among the primary headaches, in the International Classification of Headache Disorders [1]. Since its initial description, approximately 280 cases have been reported in literature. Here, we report a case series of 19 patients (10 men, 9 women), fifteen of which were retrospectively identified with the RegistRare Network, a collaborative group of seven Italian Headache Centres [2]. The remaining four patients were identified in a later period of time. Data are summarized in Table 1.

**Results**

Nummular headache was episodic (<15 days/month) in four patients and chronic (>15 days/month) in fifteen patients. Headache was appeared around 47 years (range, 18-63) and lasted approximately 11.8 years (range, 0.01-47). The pain was mainly unilateral (84% of cases). The temporal region was the most common location, followed by occipital and parietal areas. Pain is mostly described as stabbing (32%), pressing and tightening (32%) or throbbing (21%). Pain intensity can vary widely; the mean intensity, measured with Numerical Rating Scale, was 6.0 (range, 3-10). Nonsteroidal anti-inflammatory drugs (NSAIDs) were the most prescribed acute treatments, they were effective in 69% of patients. Pregabalin and amitriptyline were the most effective prophylaxis therapies (in 80% and 75% of patients, respectively).

**Conclusions**

Our series of cases reconfirm previous findings. Location, quality and intensity of pain are highly heterogeneous, whereas NSAIDs, pregabalin and amitriptyline were the most prescribed and effective therapies.

**Ethics approval**

Each participating centre received the approval of the competent Ethics Committee (for the Coordinating Centre, Careggi Hospital Headache Centre, Florence, approval #10976 of the Area Vasta Centro Section of the Tuscany Region Ethics Committee) before commencing any procedures.

**References**

1. Headache Classification Committee of the International Headache Society (IHS). The International Classification of Headache Disorders, 3rd edition. Cephalalgia 2018; 38 (1): 1-211.

2. Lupi C, Evangelista L, Favoni V, Granato A, Negro A, Pellesi L, Ornello R, Russo A, Cevoli S, Guerzoni S, Benemei S. Rare primary headaches in Italian tertiary Headache Centres: Three year nationwide retrospective data from the RegistRare Network. Cephalalgia. 2018 Jan 1: 333102418768824.

**Table 1 (abstract O53).** Clinical profiles of nummular headache patients

Patient	Sex	Age (years)	Age of onset (years)	Duration (years)	Episodic	Chronic	Location	Accompanying symptoms	Quality of Pain	NRS
1	M	42	36	6		X	Right, P	Conjunctival lacrimation	Stabbing	3
2	M	63	20	43		X	Right, O	Confusion	Persistent	5
3	F	65	63	2		X	Left, V	Tinnitus and dizziness	Stabbing	8
4	M	52	52	0.01	X		V	Not present	Stinging and stabbing	4
5	F	33	33	0.25		X	Right, P	Photophobia and nausea	Throbbing	8
6	F	40	40	0.01	X		Left, O	Dizziness	Stabbing	8
7	M	39	39	0.01	X		O	Dizziness	Stabbing	9
8	M	55	52	3		X	Left, O	Not present	Stabbing	8
9	F	54	53	1		X	Left, T	Not present	Throbbing	5
10	F	58	57	1		X	V	Not present	Pressing and tightening	2
11	F	35	33	2		X	Right, T	Not present	Pressing and tightening	3
12	M	44	18	26		X	Left, P	Not present	Stinging	5
13	M	36	36	0.33		X	Right, O	Not present	Throbbing	7
14	M	30	18	12		X	Left, T	Conjunctival injection and ptosis	Pressing and tightening	5
15	F	40	40	0.17	X		Left, T	Not present	Throbbing	7
16	M	67	20	47		X	Right, V	Not present	Pressing and tightening	5
17	F	63	63	3		X	Left, T	Not present	Pressing and tightening	6
18	F	25	25	6		X	Right, T	Not present	Pressing and tightening	5
19	M	43	43	1		X	Right, T	Facial redness	Stinging	10

*NRS* Numerical Rating Scale

## EHF Poster Presentation

### P1 The cost of migraine in Greece: a half billion euros disease

#### Panagiotis Stafylas^1^, Marianthi Karaiskou^2^, Eirini Tsiamaki^3^, Maria Kalogeropoulou^4^, Dimos-Dimitrios Mitsikostas^5^

##### ^1^HTA Consultant, HealThink, Thessaloniki, Greece; ^2^HTA Consultant, HealThink, Thessaloniki, Greece; ^3^Medical Advisor, Therapeutic Area Neuroscience, Novartis Hellas SACI, Athens, Greece; ^4^Health Economics Manager, Novartis Hellas SACI, Athens, Greece; ^5^Associate Professor of Neurology, National and Kapodistrian University of Athens, Athens, Greece

###### **Correspondence:** Maria Kalogeropoulou (maria.kalogeropoulou@novartis.com)

**Background:** Migraine is a common disabling disease that affects about one out of six to one out of five adults, most often female at productive age. The objective of this analysis is to approximate the economic burden of migraine in Greece.

**Methods:** A cost-of-Illness analysis was conducted to estimate direct and indirect costs for the management of migraine in Greece. Data on epidemiology and resource use were obtained from international literature and were validated by Greek clinical expert to cover evidence gaps. Unit costs were taken from officially published Greek sources (Ministry of Health, Social Insurance Funds -SIFs). Direct cost inputs included drug acquisition and other treatment related costs such as physician visits, hospitalization, lab and imaging tests. The results of a national survey of migraineurs in Greece reporting days absent from work (absenteeism) and days with reduced productivity (presenteeism) were used for the calculation of the indirect costs. The costs are estimated in euros with reference year 2017. The perspective of the analysis is societal.

**Results:** Assuming that the prevalence of migraine in Greek adults is 13.3%, it is estimated that about 1,208,210 adults suffer from migraine in Greece. Based on these data, the total direct cost for the management of migraine in Greece is €301,969,046. The total indirect costs due to lost productivity has been estimated at €145.085.134 (€22,919,208 and €122,165,926 due to absenteeism and presenteeism respectively). Concerning the direct costs, medications account for 12%, other therapeutic choices for 6%, physicians visits for 74%, hospitalization for 8%, other costs less than 1%. It is very interesting that from the total direct cost, about 83% represent out-of-pocket expenses. Consequently, the economic burden of migraine in Greece for the year 2017 (including both direct and indirect costs) was about €447,054,179.

**Conclusion:** The total economic burden of migraine in Greece has been estimated at about half billion euros for 2017. About one third is due to productivity loss mainly because of reduced productivity while symptomatic. About 74% of direct costs concern visits to primary care physicians and neurologists and 18% pharmaceutical or alternative treatments. It must be noticed that about 83% of the direct costs represent out-of-pocket expenses.

### P2 Single-pulse transcranial magnetic stimulation (sTMS) for the treatment of migraine: a prospective real world experience

#### Lambru Giorgio^1^, Hill Bethany^1^, Lloyd Joseph^2^, Al-Kaisy Adnan^3^, Andreou Anna P^1,2^

##### ^1^The Headache Centre, Guy’s and St Thomas’ NHS Foundation Trust, London, UK; ^2^Wolfson CARD, King’s College London, London, UK; ^3^Pain and Neuromodulation Academic Research Centre, Guy’s and St Thomas’ NHS Foundation Trust, London, UK

###### **Correspondence:** Lambru Giorgio (giorgiolambru@gmail.com)

**Objectives**: Single pulse transcranial magnetic stimulation (sTMS) is a non-invasive neuromodulation technique which has been approved in 2014 by the National Institute for Health and Care Excellence (NICE) for the acute and preventive treatment of migraine. However, its effectiveness in a real world NHS service has not been explored yet. The Headache Centre, Guy’s and St Thomas’ NHS Trust is currently the only NHS service commissioned to offer sTMS to migraine patients. Here we present our interim results.

**Methods**: This is an open-label prospective clinical audit. It aims to evaluate the effectiveness of sTMS as a non-pharmacological modality for the treatment of migraine with and without aura in a real world setting. The audit is ongoing. We present here the outcome of the first 44 consecutive treated patients with chronic or high frequency episodic migraine. Audit inclusion criteria were a documented diagnosis of chronic migraine documented in a headache diary and patients willingness in filling a headache diary and HIT-6 score, which were used to collect clinical outcomes. Change in headache days, migraine days and HIT-6 at 3 months of treatment compared to baseline were analysed. Adverse events and treatment compliance were also collected.

**Results**: Forty-two migraine patients (11 with aura, 31 without aura) treated with sTMS were analysed. Twenty patients (47.6%) received sTMS after failing Botox^®^ therapy, hence were considered refractory to medical treatments. At baseline, patients displayed an average of 14.7 headache days (HD)/month, 11.1 migraine days (MD)/month and HIT-6 score of 63.3. Following 3-month trial, 28 patients (64%) obtained a clinically meaningful benefit (- 2.7 MD/month and -5.4 points on HIT-6 score) hence continued the treatment. Seventeen patients (36%) did not benefit from the therapy and discontinued the treatment. Of those, the majority were Botox non-responders. At 6 months 1 out of 28 responders stopped the treatment due to lack of effect durability. Amongst responders, five patients continued sTMS treatment for 12 months, 10 for nine months and 12 for six months. Treatment compliance was satisfactory with sTMS used up to eight pulses three times a day. Side effects were minor and include, worsening of the headache (n = 3), transient mild dizziness during the treatment (n = 1) and scalp tenderness (n = 2).

**Conclusion**: sTMS may constitute an effective and well tolerated preventive treatment option for difficult-to-treat high frequency/chronic migraine patients in a real world setting. Since sTMS is less costly than Botox^®^ on the NHS, it could be included as one of the three preventive treatment to offer to chronic migraine patients prior to Botox^®^.

### P3 The impact of fremanezumab on symptoms associated with migraine in patients with episodic migraine

#### Jan L. Brandes^1^; Paul P. Yeung^2^; Ernesto Aycardi^2^; Ronghua Yang^2^; Yuju Ma^2^; Joshua M. Cohen^2^

##### ^1^Nashville Neuroscience Group, Vanderbilt University, Department of Neurology, Nashville, Tennessee, USA; ^2^Teva Pharmaceuticals, Frazer, Pennsylvania, USA

###### **Correspondence:** Jan L. Brandes (jtuck@hcg-int.com)

**OBJECTIVES**: Non–headache symptoms (nausea, vomiting, photophobia and phonophobia) are included in the International Classification of Headache Disorders, third edition (beta version) (ICHD-3 beta) criteria for migraine. Fremanezumab, a fully humanized monoclonal antibody (IgG2a) that selectively targets calcitonin gene-related peptide (CGRP), reduced the number of migraine days in EM patients. We assessed the effect of fremanezumab on nausea or vomiting, and photophobia and phonophobia in EM patients.

**METHODS**: In this multicenter, randomized, double-blind, placebo-controlled, Phase 3 study, patients with EM were randomized 1:1:1 to receive subcutaneous fremanezumab quarterly (675 mg at baseline, placebo at Weeks 4 and 8), fremanezumab monthly (225 mg at baseline, Weeks 4 and 8), or placebo over a 12-week treatment period. Exploratory endpoints included mean change from baseline in the monthly average number of days with nausea or vomiting, and days with photophobia and/or phonophobia during the 12-week period after the first dose of study drug. Analyses were performed in the full analysis set (all randomized patients who received ≥1 dose of study drug and had ≥10 days of post-baseline efficacy assessments on the primary endpoint). The data were analyzed using both the analysis of covariance approach, with baseline number of days with nausea or vomiting, or photophobia and phonophobia, and years since onset of migraines as covariates, and the Wilcoxon rank-sum test.

**RESULTS**: Fremanezumab treatment yielded greater reductions from baseline in the monthly number of days with nausea or vomiting during the 12-week treatment period (quarterly [least-squares mean ± standard error]: –1.9±0.19 days, *P*=0.0314; monthly: –2.1±0.19 days, *P*=0.0008) compared with placebo (–1.4±0.19 days). Reductions in nausea or vomiting were seen as early as Week 4 (quarterly: –1.7±0.21 days, *P*=0.0046; monthly: –1.9±0.21 days, *P*=0.0002) compared with placebo (–1.0±0.21 days). Fremanezumab treatment also yielded greater reductions from baseline in the number of days with photophobia and phonophobia during the 12-week treatment period (quarterly: –2.2±0.21 days, *P*=0.0038; monthly: –2.4±0.21 days, *P*=0.0001) compared with placebo (–1.5±0.21 days). Significant reductions in days with photophobia and phonophobia were seen as early as Week 4 (quarterly: –2.0±0.23 days, *P*=0.0003; monthly: –2.2±0.23 days, *P*<0.0001) compared with placebo (–1.0±0.23 days).

**CONCLUSIONS**: In patients with EM, fremanezumab treatment rapidly improved non–head pain symptoms associated with migraine, including nausea or vomiting, and photophobia and phonophobia.

**TRIAL REGISTRATION**: ClinicalTrials.gov, NCT02629861

**ETHICS APPROVAL**

The study was approved by all relevant independent ethics committees or institutional review boards, according to national or local regulations.

### P4 The impact of fremanezumab on symptoms associated with migraine in patients with chronic migraine

#### Peter McAllister^1^, Paul P. Yeung^2^, Ernesto Aycardi^2^, Ronghua Yang^2^, Yuju Ma^2^, Joshua M. Cohen^2^

##### ^1^New England Institute for Neurology and Headache, Stamford, Connecticut, USA; ^2^Teva Pharmaceuticals, Frazer, Pennsylvania, USA

###### **Correspondence:** Peter McAllister (pwong@hcg-int.com)

**OBJECTIVES**: The International Classification of Headache Disorders, third edition (beta version) (ICHD-3 beta) criteria for migraine include nausea, vomiting, photophobia, and phonophobia symptoms. Fremanezumab, a fully humanized monoclonal antibody (IgG2a) that selectively targets calcitonin gene-related peptide (CGRP), reduced the frequency and severity of headaches in patients with chronic migraine (CM). We assessed the effect of fremanezumab versus placebo on nausea or vomiting, and photophobia and phonophobia, in patients with CM.

**METHODS**: In this multicenter, randomized, double-blind, placebo-controlled, Phase 3 study, patients with CM were randomized 1:1:1 to receive subcutaneous injections of fremanezumab quarterly (675 mg at baseline, placebo at Weeks 4 and 8), fremanezumab monthly (675 mg at baseline, 225 mg at Weeks 4 and 8), or placebo (at baseline, Weeks 4 and 8) over a 12-week treatment period. Exploratory endpoints included the mean change from baseline in the monthly average number of days with nausea or vomiting, and days with photophobia and phonophobia during the 12-week period after the first dose of study drug. Analyses were performed in the full analysis set (all randomized patients who received ≥1 dose of study drug and had ≥10 days of post-baseline efficacy assessments on the primary endpoint) using analysis of covariance (with baseline number of days with the symptom, and years since onset of migraines as covariates) and the Wilcoxon rank-sum test.

**RESULTS**: Fremanezumab treatment yielded greater reductions from baseline in the monthly number of days with nausea or vomiting during the 12-week treatment period (quarterly [least-squares mean ± standard error]: –3.3±0.29 days, *P*=0.0009; monthly: –3.2±0.28 days, *P*=0.0019) compared with placebo (–2.2±0.29 days). Significant reductions in nausea or vomiting were seen as early as Week 4 (quarterly: –3.2±0.30 days, *P*<0.0001; monthly: –2.9±0.29 days, *P*=0.0014) versus placebo (–1.9±0.29 days). Fremanezumab treatment also yielded greater reductions from baseline in the number of days with photophobia and phonophobia during the 12-week treatment period (quarterly: –3.5±0.32 days, *P*=0.0025; monthly: –3.7±0.32 days, *P*=0.0001) versus placebo (–2.4±0.32 days). Reductions in days with photophobia and phonophobia were seen as early as Week 4 (quarterly: –3.5±0.33 days, *P*<0.0001; monthly: –3.5±0.32 days, *P*<0.0001) versus placebo (–2.1±0.33 days).

**CONCLUSIONS**: Fremanezumab treatment rapidly improved non–head pain symptoms associated with migraine, including nausea or vomiting, and photophobia and phonophobia, in patients with CM.

**TRIAL REGISTRATION**: ClinicalTrials.gov, NCT02638103

**ETHICS APPROVAL**

The study was approved by all relevant independent ethics committees or institutional review boards, according to national or local regulations.

### P5 Long-term impact of fremanezumab on response rates, acute headache medication use, and disability in patients with episodic migraine: interim results of a 1-year study

#### Jan L. Brandes^1*^; Paul P. Yeung^2^; Joshua M. Cohen^2^; Sanjay K. Gandhi^2^; Timothy Fitzgerald^2^ ; Ronghua Yang,^2^; Yuju Ma^2^; Ernesto Aycardi^2^

##### ^1^Nashville Neuroscience Group, Nashville, Tennessee; ^2^Teva Pharmaceuticals, Frazer, Pennsylvania, USA

###### **Correspondence:** Jan L. Brandes (MCoakley@hcg-int.com)

**BACKGROUND**: Fremanezumab, a fully humanized monoclonal antibody (IgG2a) that selectively targets calcitonin gene-related peptide (CGRP), has demonstrated efficacy in preventing episodic migraine (EM) in 3-month studies; this analysis evaluates its long-term effects.

**OBJECTIVE**: To investigate the long-term effect of fremanezumab on response, acute headache medication and disability in adults with EM.

**METHODS**: This 52-week, multicenter, randomized, double-blind, parallel-group study evaluated the long-term safety, tolerability and efficacy of fremanezumab in adults with migraine; disability was assessed using the Migraine Disability Assessment (MIDAS). Most patients rolled over from a pivotal EM study, but some patients enrolled directly into this long-term study. Patients were assigned to one of two subcutaneous dose groups: (1) monthly dosing: 225 mg doses of fremanezumab every month, or (2) quarterly dosing: 675 mg doses of fremanezumab every 3 months. Percentage of patients achieving ≥50% reduction in monthly average number of migraine days, the mean change from baseline in the monthly number of days of use of any acute headache medications, and the mean change from baseline in MIDAS score were assessed for both doses.

**RESULTS:** This study enrolled 780 EM patients. The mean change in monthly number of migraine days from baseline to Month 1 was –4.6 days for the monthly treatment group and –4.9 days for the quarterly group. The proportion of patients achieving ≥50% reduction in monthly average number of migraine days at Month 6 was 61% with monthly dosing, and 65% with quarterly dosing. The mean change in monthly number of days of use of any acute headache medications from baseline to Month 6 in patients with EM was –4.1 days in the monthly group and –4.3 days in the quarterly group. The change from baseline in the MIDAS disability score in patients with EM was similar in both treatment groups at Month 6; disability scores decreased by 27.1 and 27.3 at Month 6 in the monthly and quarterly treatment groups, respectively. For a subset of patients who completed the entire 12-month treatment period, data available at the cutoff date indicated that the response achieved at Month 6 was maintained throughout the treatment period.

**CONCLUSION**: Efficacy and disability data from this interim analysis indicated that the efficacy observed at Month 1 was maintained during the remainder of the study.

**TRIAL REGISTRATION**: ClinicalTrials.gov, NCT02638103

**ETHICS APPROVAL**

The study was approved by all relevant independent ethics committees or institutional review boards, according to national or local regulations.

### P6 Overview of fremanezumab pooled safety data from placebo-controlled phase 2 and 3 studies

#### Stephen D. Silberstein^1^, Nicola Faulhaber^2^, Xiaoping Ning^3^, Paul P. Yeung^3^, Jimmy Schiemann^3^, Ronghua Yang^3^, Yuju Ma^3^, Ernesto Aycardi^3^

##### ^1^Thomas Jefferson University, Philadelphia, Pennsylvania, USA; ^2^Teva Pharmaceuticals, Ulm, Germany; ^3^Teva Pharmaceuticals, Frazer, Pennsylvania, USA

###### **Correspondence:** Stephen D. Silberstein (abormes@hcg-int.com)

**BACKGROUND**: Fremanezumab, a fully humanized monoclonal antibody (IgG2a) that selectively targets calcitonin gene-related peptide (CGRP), has been shown to be effective in the prevention of episodic migraine (EM) or chronic migraine (CM).

**OBJECTIVE**: To summarize the safety profile of fremanezumab based on all placebo-controlled studies in patients with migraine.

**METHODS**: Fremanezumab has been studied in four placebo-controlled studies in patients with migraine, including two Phase 2 and two Phase 3 studies. Each study was a 16-week, multicenter, randomized, double-blind, placebo-controlled, parallel-group study to compare the efficacy, safety, and tolerability of fremanezumab and placebo in adults with EM or CM. The studies evaluated fremanezumab at the proposed subcutaneous doses of 225 mg monthly (CM patients received a starting dose of 675 mg), 675 mg quarterly, and at two higher doses (675 mg monthly and 900 mg monthly) for 3 months.

**RESULTS:** Most patients who received fremanezumab (N=1702) or placebo (N=861) were female (87%), with mean age of 41.4 years (range = 18 to 70 years), respectively. Serious adverse events (AEs) and AEs leading to discontinuation occurred infrequently, with similar incidences in patients who received fremanezumab (1% and 2%, respectively) versus patients who received placebo (2% for both subsets). The most common AEs in the placebo-controlled studies were injection-site reactions, including induration and erythema, which tended to be transient, mild and slightly more frequent in patients who received fremanezumab versus those given placebo. Upper respiratory tract infection and nasopharyngitis, both reported with similar incidence in patients who received either fremanezumab or placebo, were the next most frequently reported AEs. Cardiovascular AEs occurred infrequently and with a similar incidence in both fremanezumab and placebo groups. No signal for hepatoxicity was observed. No anaphylaxis or severe hypersensitivity occurred, and only three patients (two on placebo, and one on fremanezumab) had AEs of drug hypersensitivity of mild or moderate severity. None of these events was serious, and all resolved with steroid and/or antihistamine treatment. Incidence of antidrug antibody (ADA) formation was low, and there were no AEs related to ADA or neutralizing antibody development.

**CONCLUSION**: Four placebo-controlled studies demonstrate that fremanezumab, at the proposed monthly and quarterly dose regimens, is an efficacious and generally safe and well-tolerated preventive therapy.

**TRIAL REGISTRATION**: ClinicalTrials.gov, NCT02621931, NCT02629861, NCT02021773, NCT02025556

**ETHICS APPROVAL**

The study was approved by all relevant independent ethics committees or institutional review boards, according to national or local regulations.

### P7 Reversion of patients with chronic migraine to an episodic migraine classification with fremanezumab treatment

#### Joshua M. Cohen^1^; Kristen Bibeau^1^; Maja Galic^2^; Michael J. Seminerio^1^; Verena Ramirez Campos^3^; Rashmi B. Halker Singh^4^; Jessica Ailani^5^

##### ^1^Teva Pharmaceuticals, Frazer, Pennsylvania, USA; ^2^Teva Pharmaceuticals, Amsterdam, The Netherlands; ^3^Teva Pharmaceuticals, Buenos Aires, Argentina; ^4^Mayo Clinic, Phoenix, Arizona, USA; ^5^Medstar Georgetown University Hospital, Washington, District of Columbia, USA

###### **Correspondence:** Joshua M. Cohen (jbanigan@hcg-int.com)

**OBJECTIVE:** To evaluate the effect of fremanezumab on reversion from chronic migraine (CM) to episodic migraine (EM).

**BACKGROUND:** CM and EM are clinically, functionally, and anatomically differentiated, with evidence suggesting that they may be separate conditions. Furthermore, patients with CM usually have more comorbid conditions and more-frequent medication overuse, which complicates their clinical management. Fremanezumab, a fully humanized monoclonal antibody (IgG2a) that selectively targets calcitonin gene-related peptide (CGRP), has demonstrated efficacy in migraine prevention.

**DESIGN/METHODS:** In this Phase 3, multicenter, randomized, double-blind, placebo-controlled, parallel-group study, adults with prospectively confirmed CM (≥15 headache days and ≥8 migraine days per month) were randomized 1:1:1 to subcutaneous injections of fremanezumab quarterly (675 mg at baseline; placebo at Weeks 4 and 8), fremanezumab monthly (675 mg at baseline; 225 mg at Weeks 4 and 8), or matching placebo over a 12-week treatment period. *Post hoc* analyses evaluated the proportion of patients who reverted from CM to EM, defined as patients who had ≥15 headache days per month at baseline (28-day pre-treatment period) and then had <15 headache days per month in all 3 months of the treatment period.

**RESULTS:** In an analysis of the 1130 CM patients randomized in this trial (quarterly, N=376; monthly, N=379; placebo, N=375), significantly more fremanezumab-treated patients reverted from having ≥15 headache days per month at baseline to <15 headache days per month in Months 1, 2, and 3 (quarterly: 121 patients [32%]; monthly: 133 patients [35%]) than those who received placebo (86 patients [23%]; both, *P*≤0.002). On average, these fremanezumab-treated patients had 18–19 headache days per month at baseline and showed reductions to 6–9 headache days during any month in the treatment period, representing up to an approximately 70% reduction in headache days.

**CONCLUSIONS:** Along with its efficacy as a migraine preventive treatment, fremanezumab demonstrated the potential benefit for reversion from CM to EM.

**TRIAL REGISTRATION**: ClinicalTrials.gov, NCT02621931, NCT02629861

**ETHICS APPROVAL**

The study was approved by all relevant independent ethics committees or institutional review boards, according to national or local regulations.

**Disclosures:**

**Joshua M. Cohen**: Employee of Teva Pharmaceuticals.

**Kristen Bibeau**: Former employee of Teva Pharmaceuticals.

**Maja Galic**: Employee of Teva Pharmaceuticals.

**Michael J. Seminerio**: Employee of Teva Pharmaceuticals.

**Verena Ramirez Campos**: Employee of Teva Pharmaceuticals.

**Rashmi B. Halker Singh**: Received honoraria from *Current Neurology and Neuroscience Reports*, MedLink, and Amgen.

**Jessica Ailani:** Received honoraria from Allergan (speaking/consulting), Avanir (speaking), Eli Lilly (speaking/advisory board), Teva Pharmaceuticals (advisory board), Promius (speaking), *Current Pain and Headache Reports* (section editor), Theranica (clinical trials).

### P8 Efficacy of fremanezumab in patients with chronic migraine with or without concomitant use of preventive medication

#### Peter J. Goadsby^1*^, David W. Dodick^2^, Stephen D. Silberstein^3^, Paul P. Yeung^4^, Tricia Blankenbiller^4^, Xiaoping Ning^4^, Ronghua Yang^4^, Yuju Ma^4^, Ernesto Aycardi^4^, Marcelo E. Bigal^4^

##### ^1^NIHR-Wellcome Trust King’s Clinical Research Facility, King’s College, London, UK; ^2^Mayo Clinic, Phoenix, Arizona, USA; ^3^Jefferson Headache Center, Thomas Jefferson University, Philadelphia, Pennsylvania, USA; ^4^Teva Pharmaceuticals, Frazer, Pennsylvania, USA

###### **Correspondence:** Peter J. Goadsby (srieger@hcg-int.com)

**OBJECTIVE:** To investigate the efficacy of fremanezumab in chronic migraine (CM) patients with or without concomitant use of preventive medication.

**BACKGROUND:** Some patients with CM may take more than one preventive medication. Fremanezumab, a fully humanized monoclonal antibody (IgG2a) that selectively targets calcitonin gene-related peptide (CGRP), has demonstrated efficacy in migraine prevention.

**DESIGN/METHODS:** In this Phase 3, randomized, double-blind, placebo-controlled, parallel-group study, eligible patients with prospectively confirmed CM (≥15 headache days and ≥8 migraine days per month) were randomized 1:1:1 to receive subcutaneous injections of fremanezumab quarterly (675 mg at baseline; placebo at Weeks 4 and 8), fremanezumab monthly (675 mg at baseline; 225 mg at Weeks 4 and 8) or placebo at each time point over a 12-week treatment period. Changes from baseline were assessed in the monthly average number of headache days of at least moderate severity, and in migraine days in patients with or without concomitant preventive medication.

**RESULTS:** Analyses included 239 patients receiving one concomitant preventive medication (quarterly, *N*=77; monthly, *N*=85; placebo, *N*=77) and 882 patients receiving none (quarterly, *N*=298; monthly, *N*=290; placebo, *N*=294). During the 12-week treatment period, fremanezumab reduced from baseline the mean number of monthly headache days of at least moderate severity versus placebo in patients receiving concomitant preventive medication (quarterly: –3.8±0.61; monthly: –4.5±0.57; placebo: –2.5±0.61), reaching significance with monthly dosing (*P*=0.003). Reductions were also significant for fremanezumab quarterly and monthly in those not receiving concomitant preventive medication (quarterly: –4.6±0.33; monthly: –4.9±0.33; placebo: –2.7±0.33; both, *P*<0.0001). These reductions were observed as early as 4 weeks after initiation of fremanezumab monthly in patients receiving concomitant preventive medication (*P*=0.028); similarly early reductions occurred with fremanezumab monthly and quarterly in patients not receiving concomitant preventive medication (*P*<0.0001). There were also fewer migraine days with both fremanezumab regimens.

**CONCLUSIONS:** Fremanezumab demonstrated efficacy in patients with CM, regardless of concomitant preventive medication use.

**TRIAL REGISTRATION**: ClinicalTrials.gov, NCT02621931

**ETHICS APPROVAL**

The study was approved by all relevant independent ethics committees or institutional review boards, according to national or local regulations.

**Disclosures:**

**Peter Goadsby**: Personal fees from Teva Pharmaceuticals during the conduct of the study. He receives personal fees from Akita Biomedical, Alder Biopharmaceuticals, Avanir Pharma, Cipla Ltd, Dr. Reddy's Laboratories, ElectroCore LLC, Novartis, Pfizer Inc, Quest Diagnostics, Scion, MedicoLegal, UptoDate, and Oxford University Press. He receives grants and personal fees from Allergan, Amgen, Eli Lilly and Company, and eNeura Inc. He receives personal fees and other from Trigemina Inc. He reports work, personal fees from Journal Watch, and Massachusetts Medical Society, outside the submitted work. He has a patent on magnetic stimulation for headache licensed to eNeura.

**David W. Dodick**: Provides consultation to Acorda, Allergan, Amgen, Alder, Dr. Reddy’s, Merck, Promius, eNeura, Eli Lilly & Company, Insys, Autonomic Technologies, Teva, Xenon, Tonix, Trigemina, Boston Scientific, GBS, Colucid, Zosano, Laydenburg Thalmann, Biocentric, Biohaven, Magellan, Charleston Laboratories, Pfizer. Royalties: Oxford University Press and Cambridge University Press (Book Royalty). He receives editorial/honoraria from UpToDate. He receives honoraria/publishing or honoraria/royalties from Chameleon Communications, Medscape, WebMD, Academy for Continued Healthcare Learning, Haymarket Medical Education, Global Scientific Communications, HealthLogix, Academy for Continued Healthcare Learning, MeetingLogiX, Wiley Blackwell, Oxford University Press, Cambridge University Press. Stock/options: GBS/Nocira, Epien, and Mobile Health. He has a consulting use agreement with NAS. He has a board position at King-Devick Inc.

**Stephen D. Silberstein**: Provides consultation to Alder, Allergan, Amgen, Avanir, Curelater Inc., Depomed, Dr. Reddy’s Laboratories, Ensured Inc., ElectroCore Medical LLC, INSYS Therapeutics, Lilly USA LLC, Supernus Pharmaceuticals Inc., Teva Pharmaceuticals, Theranica, and Trigemina Inc.

**Paul P. Yeung**: Employee of Teva Pharmaceuticals.

**Tricia Blankenbiller**: Former employee of Teva Pharmaceuticals.

**Xiaoping Ning:** Employee of Teva Pharmaceuticals.

**Ronghua Yang**: Employee of Teva Pharmaceuticals.

**Yuju Ma**: Employee of Teva Pharmaceuticals.

**Ernesto Aycardi**: Former employee of Teva Pharmaceuticals.

**Marcelo E. Bigal**: Former employee of Teva Pharmaceuticals.

### P9 The Effect of Cognitive Behavioural Therapy on Self-Efficacy and Headache Frequency, in Chronic Migraine

#### Ayhan Bingöl, Derya Uludüz, Özlem Mercan, Yasemin Akıncı, Duygu Deringöl, Sabahattin Saip, Aksel Siva

##### Department of Neurology, Istanbul University Cerrahpasa Faculty of Medicine, Istanbul, Turkey, 34096

###### **Correspondence:** Özlem Mercan (ozlemmercan3@gmail.com)

**Background:** Chronic migraine (CM) is still an underdiagnosed and undertreated headache disorder even though the incidence and prevalence of recurrent headaches have considerably increased over the last decades. CM patients suffer more prominent disease related decrease of quality of life, occupational and educational absenteeism. CM have also burden of disease with direct and indirect costs on population and healthcare systems. Treatment of CM is not only essential to recover quality of life of sufferers, but also could provide lessening of burden of disease and economical costs. Unfortunately refractory CM is much more disabling, debilitating and challenging. Psychosocial interventions such as cognitive-behavioral therapy can improve the pain, disability and impaired quality of life and can be as cost effective as medication in refractory CM. Patients with refractory CM with medication overuse headache were studied and the results of the CBT treatment were reported.

**Methods:** Fifty patients with refractory CM and medication overuse headache were included in the study. Patients with psychiatric comorbidity such as depression or anxiety were excluded and twenty patients out of fifty were received CBT treatment. CBT treatment consists four modules, patients underwent weekly CBT sessions lasting 45 minutes for 3 months. Headache spesific self efficacy scale and patient pain attitute and belief scale were administered before and after the CBT treatment.

**Results:** The mean age of the patients was 41±9.4 years and 13 patients were female (65%). Seventy-five percent had a history of trauma. %90 of the patients had an attitude of organic belief rather than psychological belief according to pain attitute and belief scale. Self efficacy scale mean score (SD) was 66.0±10.8 before CBT treatment and 104.9±9.2 after CBT. Headache frequency was 19.0±4.7 before CBT and 1.4±2.2 after the treatment. Decrease in the headache frequency was associated with the increase of self efficacy scores.

**Conclusions**: A well structured CBT treatment including life style changes in CM patients was found to be superior to medical treatment alone for improving pain frequency even in patients without psychiatric comorbidity. The efficacy is thought to be related with the increase of self efficacy scores.

### P10 Efficacy of fremanezumab in patients with chronic migraine and comorbid moderate to moderately severe depression

#### Joshua M. Cohen^1^, Paul P. Yeung^1^, Ernesto Aycardi^1^, Marcelo E. Bigal^1^, Ronghua Yang^1^, Kristen Bibeau^1^, Maja Galic^2^, Michael J. Seminerio^1^, Richard B. Lipton^3^, Dawn C. Buse^3^

##### ^1^Teva Pharmaceuticals, Frazer, Pennsylvania, USA; ^2^Teva Pharmaceuticals, Amsterdam, The Netherlands; ^3^Albert Einstein College of Medicine and Montefiore Medical Center, Bronx, New York, USA

###### **Correspondence:** Joshua M. Cohen (mmathew@hcg-int.com)

**OBJECTIVE:** To evaluate the efficacy of fremanezumab on migraine symptoms and depression in patients with chronic migraine (CM) and comorbid moderate to moderately severe depression.

**BACKGROUND:** Depression is common in CM and contributes to the already substantial burden of disease. Fremanezumab, a fully humanized monoclonal antibody (IgG2a) that selectively targets calcitonin gene-related peptide (CGRP), has demonstrated efficacy in migraine prevention.

**DESIGN/METHODS:** In this Phase 3, multicenter, randomized, double-blind, placebo-controlled, parallel-group study, eligible patients aged 18–70, with prospectively confirmed CM (≥15 headache days and ≥8 migraine days per month) were randomized 1:1:1 to receive subcutaneous injections of fremanezumab quarterly (675 mg at baseline; placebo at Weeks 4 and 8), fremanezumab monthly (675 mg at baseline; 225 mg at Weeks 4 and 8), or matching placebo over a 12-week treatment period. *Post hoc* analyses evaluated changes in headache and migraine frequency and depression in patients with moderate to moderately severe depression (score of 10–19 on the 9-item Patient Health Questionnaire [PHQ-9]) at baseline.

**RESULTS:** Almost 20% (219/1130) of randomized patients had moderate to moderately severe depression at baseline (quarterly, n=74; monthly, n=88; placebo, n=57). As in the overall study population, fremanezumab-treated patients in this subgroup had significant reductions from baseline in the mean number of monthly headache days of at least moderate severity (quarterly: –5.4±0.79; monthly: –5.6±0.75) versus those who received placebo (–2.2±0.84) during the 12-week treatment period (both, *P*<0.001), with effects observed as early as Week 4 (*P*<0.0001). Similar treatment differences were observed for change in the mean number of migraine days (*P*<0.001). Fremanezumab also reduced the mean PHQ-9 score from baseline to Week 12 (quarterly: –10.5±0.68; monthly: –9.5±0.63) versus placebo (–8.7±0.71); the quarterly group reached significance (*P*<0.05).

**CONCLUSIONS:** Fremanezumab demonstrated efficacy in preventive treatment of CM in patients with comorbid moderate to moderately severe depression, reducing migraine and headache frequency and improving depression.

**TRIAL REGISTRATION**: ClinicalTrials.gov, NCT02621931

**ETHICS APPROVAL**

The study was approved by all relevant independent ethics committees or institutional review boards, according to national or local regulations.

**Disclosures:**

**Joshua M. Cohen**: Employee of Teva Pharmaceuticals.

**Paul P. Yeung**: Employee of Teva Pharmaceuticals.

**Ernesto Aycardi**: Former employee of Teva Pharmaceuticals.

**Marcelo E. Bigal**: Former employee of Teva Pharmaceuticals.

**Ronghua Yang**: Employee of Teva Pharmaceuticals.

**Kristen Bibeau**: Former employee of Teva Pharmaceuticals.

**Maja Galic**: Employee of Teva Pharmaceuticals.

**Michael J. Seminerio**: Employee of Teva Pharmaceuticals.

**Richard B. Lipton**: Consultant to Teva Pharmaceuticals.

**Dawn C. Buse**: Consultant to Amgen, Allergan, Avanir, Biohaven, Eli Lilly and Promeius.

### P11 Achievement of response over time with fremanezumab in the treatment of chronic and episodic migraine

#### Stephen D. Silberstein^1^, Richard B. Lipton^2^, Merle L. Diamond^3^, Joshua M. Cohen^4^, Ronghua Yang^4^, Bo Jiang^4^

##### ^1^Jefferson Headache Center, Thomas Jefferson University, Philadelphia, Pennsylvania, USA; ^2^Albert Einstein College of Medicine, Bronx, New York, USA; ^3^Diamond Headache Clinic, Chicago, Illinois, USA; ^4^Teva Pharmaceuticals, Frazer, Pennsylvania, USA

###### **Correspondence:** Stephen D. Silberstein (jtuck@hcg-int.com)

**OBJECTIVES:** The long-term efficacy of monoclonal antibodies that selectively target calcitonin gene-related peptide (CGRP) in patients with early treatment failure is not well characterized. Based on data from Phase 3 trials in episodic (EM) and chronic migraine (CM) of fremanezumab, a fully humanized monoclonal antibody (IgG2a) that selectively targets CGRP, we assessed long-term treatment response rates in patients with early treatment failure.

**METHODS:** This multicenter, randomized, double-blind, parallel-group, long-term study, included patients who completed either 12-week Phase 3 study (HALO CM or HALO EM). Patients continued on treatment from the 12-week studies, receiving either subcutaneous fremanezumab quarterly (675 mg every 3 months), fremanezumab monthly (CM: 675 mg at baseline and 225 mg every month; EM: 225 mg every month) over a 12-month treatment period. The percentage of patients with a reduction in migraine days (response rates) >40% at Months 6 and 9 among patients with low response rates (<40%) at Month 1 was assessed in patients who received active treatment in the 12-week studies.

**RESULTS:** CM patients with <20% reduction in migraine days at Month 1 had >40% response rates of 29% (58/197) at Month 6 and 43% (35/81) at Month 9. Patients with <20% reduction at Month 3 had >40% response rates of 18% (32/176) at Month 6 and 30% (21/69) at Month 9. Patients with <40% reduction at Month 1 had >40% response rates of 36% (99/272) at Month 6 and 51% (55/108) at Month 9. Patients with <40% reduction at Month 3 had >40% response rates of 28% (72/253) at Month 6 and 41% (41/101) at Month 9.

EM patients with <20% reduction in migraine days at Month 1 had >40% response rates of 53% (53/100) at Month 6 and 62% (26/42) at Month 9. Patients with <20% reduction at Month 3 had >40% response rates of 41% (34/83) at Month 6 and 47% (15/32) at Month 9. Patients with <40% reduction at Month 1 had >40% response rates of 57% (92/162) at Month 6 and 63% (45/72) at Month 9. Patients with <40% reduction at Month 3 had >40% response rates of 46% (62/135) at Month 6 and 61% (33/54) at Month 9.

**CONCLUSIONS:** Failure to achieve an early response to fremanezumab does not predict failure at later time points.

**TRIAL REGISTRATION**: ClinicalTrials.gov, NCT02638103

**ETHICS APPROVAL**

The study was approved by all relevant independent ethics committees or institutional review boards, according to national or local regulations.

### P12 The impact of fremanezumab on medication overuse in patients with chronic migraine

#### Stephen D. Silberstein^1^, Sait Ashina^2^, Zaza Katsarava^3^, Kristen Bibeau^4^, Michael J. Seminerio^4^, Danielle E. Harlow^4^, Joshua M. Cohen^4^

##### ^1^Jefferson Headache Center, Thomas Jefferson University, Philadelphia, Pennsylvania, USA; ^2^Beth Israel Deaconess Medical Center Comprehensive Headache Center, Harvard Medical School, Boston, Massachusetts, USA; ^3^University of Essen, Unna, Germany; ^4^Teva Pharmaceuticals, Frazer, Pennsylvania, USA

###### **Correspondence:** Stephen D. Silberstein (khokenson@hcg-int.com)

**OBJECTIVES**: Overuse of acute or symptomatic headache medications (triptans, ergot derivatives, opioids, and combination analgesics) can cause medication overuse headache (MOH), which often accompanies chronic migraine (CM). Fremanezumab, a fully humanized monoclonal antibody (IgG2a) that selectively targets calcitonin gene-related peptide (CGRP), reduced the frequency and severity of headaches in CM patients. We assessed the effect of fremanezumab on medication overuse and acute headache medication use in CM patients.

**METHODS**: In this multicenter, randomized, double-blind, placebo-controlled, Phase 3 study, CM patients CM were randomized 1:1:1 to receive subcutaneous fremanezumab quarterly (675 mg at baseline, and placebo at Weeks 4 and 8), fremanezumab monthly (675 mg at baseline, and 225 mg at Weeks 4 and 8), or placebo over a 12-week treatment period. We assessed the proportion of patients who reverted from overusing medications at baseline (use of acute headache medication on ≥15 days, use of migraine-specific acute medication on ≥10 days, or use of combination medications for headache on ≥10 days during the 28-day baseline period) to not overusing medications at Week 12, and the change from baseline in the number of days of acute headache medication use among these patients. Analyses were performed in the full analysis set (all randomized patients who received ≥1 dose of study drug and had ≥10 days of post-baseline efficacy assessments on the primary endpoint).

**RESULTS**: Among patients with medication overuse at baseline (quarterly n=201; monthly n=198; placebo n=188), more fremanezumab-treated patients reported no medication overuse during the 12-week treatment period (quarterly: 111/201 patients [55%], *P*=0.0389; monthly: 120/198 patients [61%], *P*=0.0024) than those who received placebo (87/188 patients [46%]). This response was seen as early as Week 4 (quarterly: 102/201 patients [51%], *P*=0.0091; monthly: 107/198 patients [54%], *P*=0.0014; vs placebo: 73/188 patients [39%]). Among patients who responded (quarterly n=111; monthly n=120; placebo n=87), the baseline number of days with medication overuse was similar across treatment groups (quarterly [mean ± standard error]: 16.6±0.32 days; monthly: 16.7±0.33 days; placebo: 16.6±0.35). Within this population, fremanezumab treatment reduced the days of acute headache medication use over the treatment period (quarterly: –9.0±0.41 days, *P*=0.0017; monthly: –8.9±0.41 days, *P*=0.0040) versus those who received placebo (–7.1±0.46 days).

**CONCLUSIONS**: Fremanezumab treatment was associated with reduced overuse of acute medications and fewer days using acute medications.

**TRIAL REGISTRATION**: ClinicalTrials.gov, NCT02621931

**ETHICS APPROVAL**

The study was approved by all relevant independent ethics committees or institutional review boards, according to national or local regulations.

### P13 Work productivity amongst those with migraine: results from the My Migraine Voice survey

#### Todd J. Schwedt^1^, Rebeca Quintana^2^, Veruska Carboni^3^, Paolo Martelletti^4, 5^, Michel Lanteri-Minet^6^, Hans-Christoph Diener^7^, Annik K.-Laflamme^8^, Elena Ruiz de la Torre^9^, Audrey Craven^10^, Annette Vangaa Rasmussen^11^, Donna Walsh^12^, Simon Evans^13^, Paula Dumas^14^, Rachel Fink^8^, Angela Fiorin^8^, Stephanie Ribbe^8^, Pamela Vo^8^

##### ^1^Neurology Arizona, Mayo Clinic, Phoenix, United States; ^2^GFK, Madrid, Spain; ^3^GfK Health, Basel, Switzerland; ^4^Department of Clinical and Molecular Medicine, Sapienza University of Rome, Rome, Italy; ^5^EHF; ^6^Département d’Evaluation et Traitement de la Douleur, Centre Hospitalo-Universitaire de Nice, -, France; ^7^Department of Neurology and Headache Center, University Duisburg-Essen, -, Germany; ^8^ovartis Pharma AG, Basel, Switzerland; ^9^European Headache Alliance, Brussels, Belgium; ^10^Migraine Association Ireland, Dublin, Ireland ^11^Rigshospitalet Glostrup, Copenhagen, Denmark; ^12^European Federation of Neurological Associations, Brussels, Belgium; ^13^Migraine Action, Leicester, United Kingdom; ^14^Migraine Again, United States

###### **Correspondence:** Todd J. Schwedt (Schwedt.Todd@mayo.edu)

**Objectives**

Migraine-induced disability results in substantial economic and societal burden globally [1]. However, limited evidence exists for those with migraine who have used preventive medication. As part of the worldwide My Migraine Voice survey, this study aimed to describe the impact of migraine on work and activity impairment amongst migraine individuals who suffer from at least 4 monthly migraine days (MMDs) and reported use of preventive medication.

**Methods**

A cross-sectional study was conducted using an online worldwide survey of migraine patients from 31 countries across Africa, America, Asia, and Europe, recruited via online panels and patient organizations. Study participants were adult patients (≥18 years) who reported ≥4 MMDs over the 3 months preceding the time of the survey (September 2017- February 2018), with pre-specified 90% of them having reported having used preventive migraine treatments. The impact of migraine on work productivity and activities during the past seven days (prior to survey completion) was evaluated using the work productivity and activity impairment (WPAI) questionnaire and was compared among treatment naive, no prior treatment failure (TF), 1 TF, and ≥2 TF patient subgroups.

**Results**

A total of 11,266 migraine patients with at least 4MMDs responded to the survey (75% women, mean age: 39 years old). Migraine patients reported overall a reduction of 13% in their working time (absenteeism), 48% in productivity while working (presenteeism), and 52% in both overall work productivity (absenteeism and presenteeism combined) and daily activities due to migraine. Descriptive analysis of results by prior treatment showed that all WPAI outcomes are impacted by migraine especially in those who have failed 2 or more prior prophylactic treatments.

**Conclusion**

This large worldwide study shows that migraine is associated with work productivity and activity impairment especially in those patients who have experienced two or more treatment failures.

**Ethics approval**

Data was handled confidentially and anonymity of respondents was maintained throughout the study. Participants’ consent was obtained prior to participation in the survey.

**Funding**

This study was funded by Novartis Pharma AG, Basel, Switzerland

**References**

1. Steiner TJ, Stovner LJ, Vos T, Jensen R, Katsarava Z. Migraine is first cause of disability in under 50s: will health politicians now take notice? The Journal of Headache and Pain. 2018;19(1):17.

### P14 Headache following mild traumatic brain injury (MTBI) in a population-based, controlled, longitudinal study

#### Lena H. Nordhaug^1^, Mattias Linde^1^, Turid Follestad^2^ Toril Skandsen^1^, Anne Vik^1^

##### ^1^Department of Neuromedicine and Movement Science, Faculty of Medicine and Health Sciences, Norwegian University of Science and Technology, Postbox 8905, 7491 Trondheim, Norway; ^2^Department of Public Health and Nursing, Faculty of Medicine and Health Sciences, Norwegian University of Science and Technology, Trondheim, Norway

###### **Correspondence:** Lena H. Nordhaug (lena.h.nordhaug@ntnu.no)

**Background**

Headache is the most frequent symptom following mild traumatic brain injury (MTBI), but the majority of data published are from hospital admissions only. We explored both hospitalized and non-hospitalized subjects with MTBI and compared them to a healthy control group and a control group with orthopaedic injuries to examine whether the MTBI group had an increase in headache suffering following the head injury.

**Methods**

This was a population-based, controlled, longitudinal cohort study. Patients were recruited from the emergency departments at a level 1 trauma-center and a municipal outpatient-clinic. Information regarding the participants´ headache status was collected through questionnaires at baseline (with information on headache suffering during the last 12 months), 3 and 12 months after the MTBI. We used generalized linear mixed model to examine whether there was an interaction effect between the three groups over time regarding headache status (headache yes/no).

The Regional committee for research ethics approved the study. Participants, or parents of participants < 18 years, gave written consent.

**Results**

378 MTBI patients were included. The control groups consisted of 83 healthy controls and 82 orthopedic controls. Mean age in the MTBI group was 31.2 years (SD ±13.0). The majority of the participants was male (65%) and 69% were treated without hospital admittance. There was a significant interaction between time and participant group (p = 0.042). Figure 1 shows the trajectories of log odds for headache for the three participants groups as reported in the three questionnaires. The increase in odds of headache from pre-injury status to 3 months was significantly larger for the MTBI group than orthopedic controls (ratio of OR 4.25, 95% CI 1.46-12.40), and healthy controls (ratio of OR 3.53, 95% CI 1.08 – 11.59). The change in odds of headache from pre-injury status to 12 months, and from 3 months to 12 months, did however not differ between the groups. In the MTBI group the odds of headache increased significantly from pre-injury to 3 months (OR 8.62, 95% CI 4.90-15.17) and 12 months (OR 3.73, 95% CI 2.19 – 6.33), but a significant decrease in odds of headache from 3 months to 12 months was observed (OR 0.43, 95% CI 0.25-0.74).

**Conclusions**

There was a significantly larger increase in odds of headache from pre-injury status to 3 months for the MTBI group compared to orthopedic and healthy controls, but the change in odds of headache from pre-injury status to 12 months did not differ between the groups.

**Fig. 1 (abstract P14).**
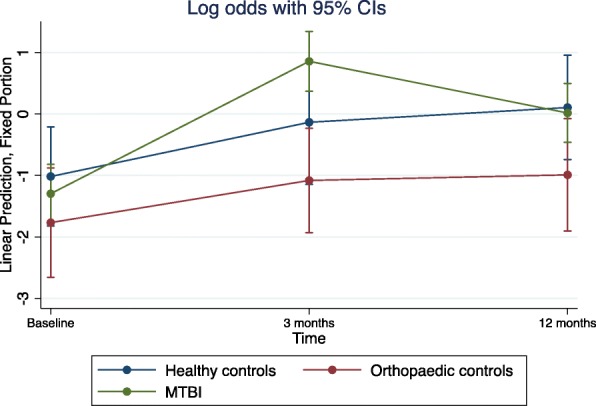
Log odds with 95% CI for headache pre-injury, 3 months and 12 months for all three participant groups

### P15 A randomised, double-blind, placebo-controlled study of erenumab safety in patients with stable angina

#### Christophe Depre^1^, Lubomir Antalik^2^, Amaal Starling^3^, Michael Koren^4^, Osaro Eisele^1^, Daniel D. Mikol^1^

##### ^1^Amgen Inc., Thousand Oaks, CA, USA; ^2^Regional Hospital, Cardiological Department, Slovakia; ^3^Mayo Clinic, Scottsdale, AZ, USA; ^4^Jacksonville Center for Clinical Research, Jacksonville, FL, USA

###### **Correspondence:** Lubomir Antalik (antalik@nspbr.sk)

**Background**

During myocardial ischaemia, cardiac sensory nerves release a number of vasodilatory and cytoprotective mediators, including the calcitonin gene-related peptide (CGRP). Erenumab, a fully human anti-CGRP receptor antibody approved as a preventive treatment for migraine by the US FDA. We previously reported that erenumab did not adversely affect exercise time in an at-risk population of patients with stable angina. Here we provide additional analyses of erenumab effects on cardiovascular function and haemodynamics in a high-risk population of patients with stable angina.

**Methods**

In this double-blind, placebo-controlled study in patients with stable angina due to documented coronary artery disease (CAD), patients were randomised 1:1 to a single intravenous (IV) infusion of erenumab 140 mg or placebo, stratified based on baseline total exercise time average (<7 minutes or ≥7 minutes) of two qualifying exercise treadmill tests (ETTs) performed during screening. Following IV study drug administration, an ETT was conducted on Day 1. Safety follow-up visits occurred every 2–4 weeks for 12 weeks. The primary endpoint, previously reported, was change from baseline in exercise duration. Safety analyses included blood pressure (BP), heart rate (HR), laboratory assessments, vital signs, electrocardiograms, and monitoring of adverse events (AEs).

**Results**

Demographics and baseline disease characteristics of the 89 enrolled patients, balanced between treatment groups, were representative of a high-risk patient population: 100% had cardiovascular disease, ~40% had history of myocardial infarction, and 24% had history of cerebrovascular or peripheral arterial disease. At baseline, mean (standard deviation) systolic (SBP) and diastolic blood pressure (DBP; mmHg) was 132.1 (18.3) and 80.0 (11.8) in the placebo group and 129.3 (12.0) and 77.8 (13.6) in the erenumab group. Mean baseline heart rate (beats/min) was 70.5 (13.4) and 69.6 (10.0), respectively. ETT and last measured post-ETT SBP, DBP and HR are presented (Table 1). The mean changes from baseline in SBP, DBP and HR during the safety follow-up period were similar between the two groups.

Adverse events, reported by 32% and 27% of patients in placebo and erenumab groups, were mostly singularly reported. One serious AE (atrial fibrillation with placebo) was reported. No clinically significant changes in serum chemistry or haematology laboratory values were observed. No deaths occurred during the study.

**Conclusions**

These results demonstrate that erenumab has no adverse haemodynamic effects during exercise in patients with CAD and stable angina and does not impact functional recovery, providing reassurance that CGRP receptor blockade does not negatively impact cardiovascular function in patients with CAD.

**Table 1 (abstract P15).** Outcome measures

Parameter	Erenumab 140 mg	Placebo
ETT		
Peak SBP	172.1 (22.9)	172.2 (22.4)
Peak DBP	90.1 (15.1)	90.3 (15.4)
Peak heart rate	122.2 (19.7)	122.6 (22.0)
Post-ETT		
SBP	134.5 (15.2)	135.0 (18.5)
DBP	76.2 (9.3)	79.2 (11.6)
Heart rate	73.3 (8.7)	78.8 (13.7)

*ETT* exercise treadmill tests, *DBP* diastolic blood pressure, *SBP* systolic blood pressure

### P16 Burden and patient-reported outcomes of migraine patients with prior prophylactic treatment failure: Study design of the multinational BECOME study

#### Paolo Martelletti^1^, Christian Lucas^2^, Patricia Pozo-Rosich^3^, David Watson^4^, Charly Gaul^5^, Shannon Ritter^6^, Josefin Snellman^6^

##### ^1^Department of Clinical and Molecular Medicine, Sapienza University of Rome, Sant'Andrea Hospital, Via di Grottarossa, Rome, Italy; ^2^Hopital Salengro, CHRU de Lille, Service de Neurochirurgie, Lille Cedex, France; ^3^Headache Unit, Neurology Department, Vall d'Hebron University Hospital, Barcelona, and Headache Research Group, Vall d’Hebron Institute of Research (VHIR), Universitat Autonoma of Barcelona, Spain; ^4^Hamilton Medical Group, Aberdeen, Scotland; ^5^Migraine and Headache Clinic Königstein, Königstein im Taunus, Germany; ^6^Novartis Pharma AG, Basel, Switzerland

###### **Correspondence:** Paolo Martelletti (paolo.martelletti@uniroma1.it)

**Background**

Migraine is the most prevalent primary headache disorder in tertiary care and can impair a patient’s quality of life. However, there is limited data available on the disease burden and quality of life in migraine patients with prior treatment failure from European countries.

**Objectives**

The primary objective of the BECOME study is to describe the proportion of migraine patients with at least one prior prophylactic treatment failure. Other objectives include assessment of impact of migraine on quality of life in this population and estimate the resulting healthcare resource utilisation (HRU).

**Methods**

BECOME is a multicentre, prospective, non-interventional study, being conducted simultaneously in two parts over three consecutive months in 167 headache clinics across 17 European countries and Israel in adult migraine patients (aged 18–65 years) who have failed more than one preventive treatment within the last 5 years. The study protocol was approved by the IRB/IEC and was in accordance with the Declaration of Helsiniki. In Part 1 of the study, the proportion of migraine patients is determined by weekly collection of site-specific data for a period of 3 months from patients visiting (Visit 0) the individual healthcare centres as an in- or out-patient (Fig. 1). For the cross-sectional Part 2, patients from Part 1, identified by investigators according to local routine clinical practice, who are willing to complete a set of questionnaires and provide study participation consent are invited for a subsequent study visit (Visit 1) within 14 days from Visit 0. A set of questionnaires are used to collect patient-specific data on disease characteristics, patient-reported outcomes, burden of disease, and HRU (Fig. 1). A sample size of 2462 patients will be included in Part 2 to give an overall error rate of 1.6% (half-width of 95% confidence interval), under the assumption that 80% of patients discontinue at least one therapy.

**Conclusions**

The outcome of the study will provide proportion of migraine patients with at least one prior treatment failure visiting the headache clinics in Europe and Israel, and describe patient characteristics and perspectives during disease management in real-life setting. This information can help in treatment decisions for patients with limited treatment options.

**Fig. 1 (abstract P16).**
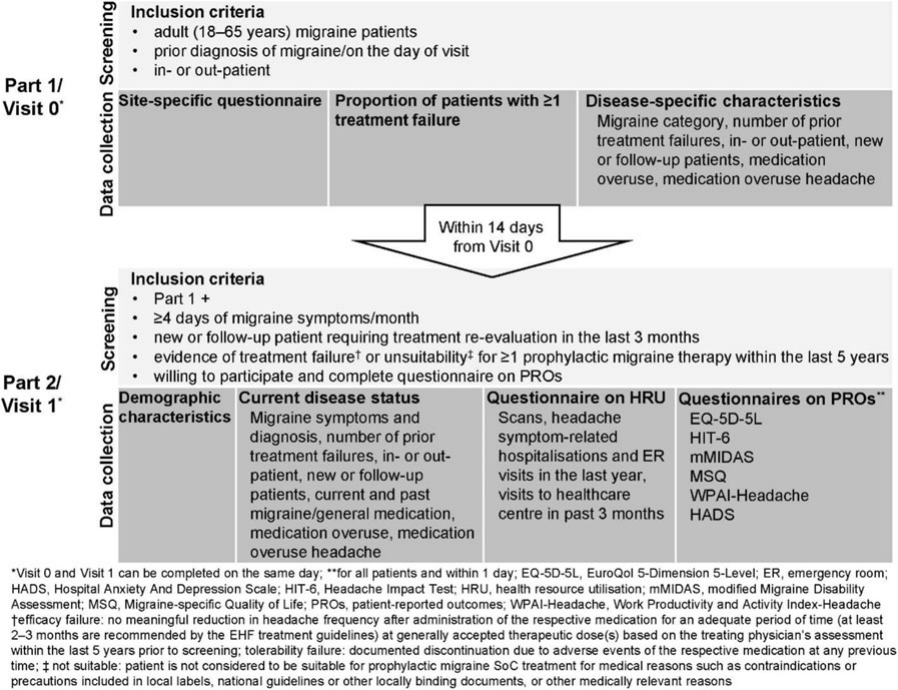
BECOME study design and data collection during Parts 1 and 2

### P17 My Migraine Voice: a worldwide survey of 11,266 migraine patients

#### Elena Ruiz de la Torre^1^, Rebeca Quintana^2^, Veruska Carboni^3^, Paolo Martelletti^4,5^, Todd J. Schwedt^6^, Michel Lanteri-Minet^7^, Hans-Christoph Diener^8^, Annik K.-Laflamme^9^, Annette Vangaa Rasmussen^10^, Donna Walsh^11^, Audrey Craven^12^, Simon Evans^13^, Paula Dumas^14^, Rachel Fink^9^, Angela Fiorin^9^, Stephanie Ribbe^9^, Pamela Vo^9^

##### ^1^European Headache Alliance, Brussels, Belgium; ^2^GFK, Madrid, Spain; ^3^GfK Health, Basel, Switzerland; ^4^Department of Clinical and Molecular Medicine, Sapienza University of Rome, Rome, Italy; ^5^EHF; ^6^Neurology Arizona, Mayo Clinic, Phoenix, United States; ^7^Département d’Evaluation et Traitement de la Douleur, Centre Hospitalo-Universitaire de Nice, France,; ^8^Department of Neurology and Headache Center, University Duisburg-Essen, -, Germany; ^9^Novartis Pharma AG, Basel, Switzerland; ^10^Rigshospitalet Glostrup, Copenhagen, Denmark; ^11^European Federation of Neurological Associations, Brussels, Belgium; ^12^Migraine Association Ireland, Dublin, Ireland; ^13^Migraine Action, Leicester, United Kingdom; ^14^Migraine Again, United States

###### **Correspondence:** Elena Ruiz de la Torre (elena@europeanheadachealliance.org)

**Introduction**

The *My Migraine Voice* survey was conducted to assess migraine characteristics and describe the current real-world burden and impact of living with migraine from clinical, personal, and economic perspectives amongst those with at least 4 monthly migraine days (MMDs). This analysis reports on survey respondents’ demographics, migraine characteristics, use of migraine therapies, and association with other chronic conditions.

**Methods**

A worldwide cross-sectional study was conducted using a 30-minute online survey of adults with migraine recruited from 31 countries across Africa, America, Asia and Europe via online panels and patient organizations. To be included, participants had to report >=4 MMDs in the 3 months preceding survey administration (September 2017-February 2018). High-need patients were prioritized by ensuring 90% of patients had reported preventive migraine treatment use (pre-specified).

**Results**

A total of 11,266 individuals with migraine participated (75% female, mean age=39 years). Of all respondents, 47% were employed full-time, 56% married, 63% had children and 54% had migraine family history. Patients had had migraine for 11.6 years on average (27% for >20 years), 54% received a diagnosis within 6 months of consulting for their symptoms (19% within >=2 years), and 83% reported taking prescription medications for acute treatment (51% also take OTC drugs). Respondents reported 3.3 other chronic conditions on average (top 3: anxiety, insomnia/sleep disorders, and depression).

**Conclusion**

This large worldwide study constitutes a rich source of data to further describe this economically active population with prior treatment usage and with personal, social and professional commitments, and high healthcare use.

**Ethics approval**

Data was handled confidentially and anonymity of respondents was maintained throughout the study. Participants’ consent was obtained prior to participation in the survey.

**Funding**

This study was funded by Novartis Pharma AG, Basel Switzerland

### P18 Economic burden of migraine: healthcare resource utilization in the My Migraine Voice survey

#### Paolo Martelletti^1,2^, Rebeca Quintana^3^, Veruska Carboni^4^, Todd J. Schwedt^5^, Michel Lanteri-Minet^6^, Hans- Christoph Diener^7^, Annik K.-Laflamme^8^, Elena Ruiz de la Torre^9^, Audrey Craven^10^, Simon Evans^11^, Donna Walsh^12^, Annette Vangaa Rasmussen^13^, Paula Dumas^14^, Rachel Fink^8^, Angela Fiorin^8^, Stephanie Ribbe^8^, Pamela Vo^8^

##### ^1^Department of Clinical and Molecular Medicine, Sapienza University of Rome, Rome, Italy; ^2^EHF; ^3^GFK, Madrid, Spain; ^4^GfK Health, Basel, Switzerland; ^5^Neurology Arizona, Mayo Clinic, Phoenix, United States; ^6^Département d’Evaluation et Traitement de la Douleur, Centre Hospitalo-Universitaire de Nice, France; ^7^Department of Neurology and Headache Center, University Duisburg-Essen, Germany; ^8^Novartis Pharma AG, Basel, Switzerland ^9^European Headache Alliance, Brussels, Belgium; ^10^Migraine Association Ireland, Dublin, Ireland; ^11^Migraine Action, Leicester, United Kingdom; ^12^European Federation of Neurological Associations, Brussels, Belgium; ^13^Rigshospitalet Glostrup, Copenhagen, Denmark; ^14^Migraine Again, United States

###### **Correspondence:** Paolo Martelletti (paolo.martelletti@uniroma1.it)

**Introduction**

Migraine is a distinct neurological disease ranking among the top causes of disability globally [1]. This study aimed to evaluate the real-world healthcare resource utilization due to migraine, particularly among individuals who suffer from at least 4 monthly migraine days (MMDs).

**Methods**

A cross-sectional study was conducted using an online survey of migraine patients in 31 countries across Africa, America, Asia and Europe, recruited via online panels and patient organizations. Study participants were adults (>=18 years) who reported having >=4 MMDs over the 3 months preceding the time of the survey (September 2017-February 2018), with pre-specified 90% among those having reported having used preventive migraine treatment.

**Results**

A total of 11,266 migraine patients responded to the survey (75% women, mean age: 39 years old). Migraine patients with at least 4 MMDs reported visiting different healthcare professionals (HCP) in a 6- month period due to their migraine. In the previous 12 months, 38% of patients visited the Emergency room (ER) an average 3.3 times, and 23% stayed in hospital overnight for an average of 3.2 days. Brain scans were performed on 58% of patients on average 2.1 times. These proportions were both higher in Turkey, India, Brazil, Indonesia, Poland, USA, Portugal, Russia, and also for migraine patients who have failed >=2 prophylactic treatments (Table 1).

**Conclusion**

Migraine patients (>= 4MMD) reported a high rate of HCP, ER visits and overnight stays in the hospital due to migraine; results indicate that the burden is higher among those who have failed a prophylactic migraine treatment and this trend increased with the number of prophylactic treatment failures.

**Ethics approval**

Data was handled confidentially and anonymity of respondents was maintained throughout the study. Participants’ consent was obtained prior to participation in the survey.

**Funding**

This study was funded by Novartis Pharma AG, Basel, Switzerland

**Table 1 (abstract P18).** Resource utilization in previous 12 months in migraine individuals

	Overall (N=11266)	No prior prophylactic treatment	No prophylactic TF	1 prophylactic TF	2 or more prophylactic TFs
Brain scan					
Proportion of patients (%)	58%	29%	46%	56%	68%
Average number of scans	2.1	1.7	1.8	1.7	2.3
ER visits					
Proportion of patients (%)	38%	17%	28%	28%	46%
Average number of visits	3.3	2.9	2.5	2.7	3.5
Overnight hospital stay in past 12 months					
Proportion of patients (%)	23%	8%	14%	15%	29%
Average nights of stay	3.2	2.6	2.6	2.3	3.4

*ER* emergency room, TF treatment failure; Data refer to 12 months prior to survey completion; survey took place between September 2017 and February 2018 in 31 countries

**References:**

1. Steiner TJ, Stovner LJ, Vos T, Jensen R, Katsarava Z. Migraine is first cause of disability in under 50s: will health politicians now take notice? The Journal of Headache and Pain. 2018;19(1):17.

### P19 Effect of galcanezumab following double-blind treatment in patients with migraine: results from EVOLVE-1 and EVOLVE-2

#### Qi Zhang, Paula A. Morrow, Virginia L. Stauffer, Vladimir Skljarevski, Eric M. Pearlman, Sheena K Aurora

##### Eli Lilly and Company, Indianapolis, IN 46285 USA

**Correspondence:** Virginia L. Stauffer (stauffer_virginia@lilly.com)

**Objective**: Galcanezumab, a humanized monoclonal antibody that binds calcitonin gene-related peptide, has a half-life of 27 days so the effect can persist after the last injection. Here, we examine the efficacy and safety data from the post-treatment period of the phase 3 studies in patients with episodic migraine.

**Methods**: Patients aged 18-65 years with 4 to 14 baseline number of migraine headache days were enrolled into two double-blind, placebo-controlled, migraine prevention studies. The studies randomized 858 patients (EVOLVE-1) and 915 patients (EVOLVE-2) to receive galcanezumab 120mg (with 240mg loading dose at first month) or 240mg galcanezumab or placebo, which was administered subcutaneously once/month for 6 months. After completion or discontinuation of the 6-month treatment period, patients were to enter a 4-month post-treatment (PT) period without receiving galcanezumab or placebo. Efficacy (daily headache diary) and safety measures including post-treatment emergent adverse events (AEs) were collected. Change from baseline in number of migraine headache days (MHDs) over the 10 months of study were analyzed with mixed model repeated measures analysis. Time to first loss of 50% response were analyzed with Kaplan-Meier method with log rank test.

**Results**: Among 740 (EVOLVE-1) and 830 (EVOLVE-2) patients entered in the 4 month PT period, 95.1% (EVOLVE-1) and 96.0% (EVOLVE-2) completed the PT period. For change from baseline in monthly MHDs over the 10 months of study, the differences between each galcanezumab dose group and placebo were statistically significant at each month (smaller differences at months 7 to 10), with the exception of one galcanezumab dose group at month 10 for both studies (Figs. 1 and 2). There were not statistically significant differences between treatment groups for time to first loss of 50% response. By last month of PT period, about 50% patients across all treatment groups had first loss of 50% response in both studies. The only PT emergent AEs that were >1.0% were viral upper respiratory tract infection (URTI). There were no discontinuations due to AEs during the PT period for both studies.

**Conclusion**: Both treatment groups continued to have reduction in MHD frequency compared to baseline, but the treatment differences were lower once glacanezumab was stopped. No new safety signals emerged following cessation of treatment with galcanezumab.

**Trial registration**: ClinicalTrials.gov identifiers NCT02614183 (EVOLVE-1) and NCT02614196 (EVOLVE-2)

**Ethics approval**

These studies were approved by the appropriate institutional review board for each of the study sites. They were all conducted according to Good Clinical Practice and the Declaration of Helsinki guidelines.

**Fig. 1 (abstract P19).**
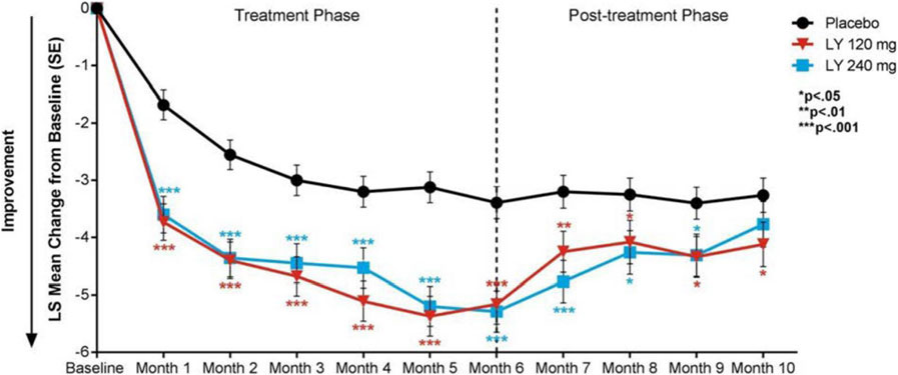
Change from baseline in the number of monthly migraine headache days during 6 months treatment and 4 month post-treatment period for EVOLVE-1

**Fig. 2 (abstract P19).**
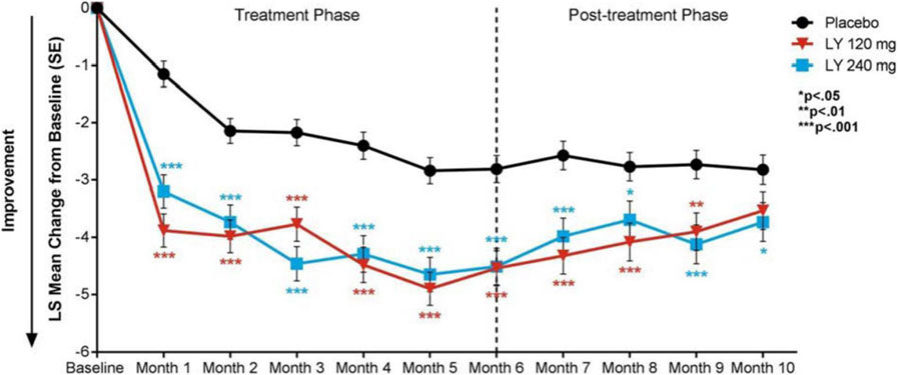
Change from baseline in the number of monthly migraine headache days during 6 months treatment and 4 month post-treatment period for EVOLVE-2

### P20 Sustained efficacy over 1 year of treatment with erenumab: results from the extension phase of the STRIVE study in episodic migraine

#### Peter J. Goadsby^1^, Uwe Reuter^2^, Yngve Hallström^3^, Gregor Broessner^4^, Jo H Bonner^5^, Feng Zhang^6^, Sandhya Sapra^6^, Denise E Chou^6^, Jan Klatt^7^, Hernan Picard^6^, Robert A Lenz^6^, Daniel D Mikol^6^

##### ^1^NIHR-Wellcome Trust King’s Clinical Research Facility, King’s College Hospital, London, UK; ^2^Department of Neurology, Charité Universitätsmedizin Berlin, Berlin, Germany; ^3^Neuro Center, St Görans Hospital, Stockholm, Sweden; ^4^Department of Neurology, Headache Outpatient Clinic, Medical University of Innsbruck, Innsbruck, Austria; ^5^Mercy Research, Saint Louis, MO, USA; ^6^Amgen Inc., Thousand Oaks, CA, USA; ^7^Novartis Pharma AG., Basel, Switzerland

###### **Correspondence:** Peter J. Goadsby (peter.goadsby@kcl.ac.uk)

**Background**

To assess efficacy and safety of erenumab in the 28-week, dose-blinded, active treatment phase (ATP) of the Phase 3 STRIVE study (NCT02456740).

**Methods**

955 patients were randomised (1:1:1) during the 24-week double-blind treatment phase (DBTP) of the STRIVE study to placebo, erenumab 70mg or 140mg administered subcutaneously, once-monthly. At the end of 24 weeks DBTP, 845 patients were re-randomised (1:1) to receive erenumab 70mg or 140mg (during the 28-week dose-blinded ATP). Monthly migraine days (MMDs), monthly acute migraine-specific medication treatment days (MSMDs), >=50% responder rates in MMD, and safety were assessed.

**Results**

At Week 52, patients receiving 140mg or 70mg during the ATP showed changes from baseline/Week 24 in MMD of −4.64/−1.78 and −4.22/−1.10, respectively (Table 1). For patients switching from placebo to 140mg or 70mg, change from baseline/Week 24 in MMD was −4.50/−2.86 and −3.71/−2.18, recapitulating the numerically greater efficacy observed for 140mg vs 70mg in the DBTP. For patients increasing dose from 70mg to 140mg during the ATP, the change from Week 24 to 52 in MMD was −1.82; and −0.07 for patients decreasing dose from 140mg to 70mg. Overall, numerically greater efficacy was observed for patients receiving erenumab 140mg vs 70mg during the ATP. Safety of erenumab in the ATP was similar to that observed in the DBTP and in prior studies.

**Conclusion**

Over 52 weeks, erenumab provides sustained efficacy in prevention of episodic migraine and a safety profile comparable to placebo as observed in prior studies.

**Table 1 (abstract P20).** Outcome measures

Outcome measures	Erenumab 140 mg (ATP)(N=424)	Erenumab 70 mg (ATP)(N=421)
MMD		
Baseline MMD, mean (SD)	8.23 (2.43)	8.34 (2.48)
Change from baseline to Week 52	−4.64 (0.19)	−4.22 (0.22)
Change from Week 24 to Week 52 (ATP)	−1.78 (0.19)	−1.10 (0.21)
Proportion of patients achieving >=50% reduction from baseline in MMD at Week 52, n/N1 (%)	239/368 (64.9)	225/369 (61.0)
MSMD		
Baseline MSMD, mean (SD)	3.49 (3.49)	3.60 (3.41)
Change from baseline to Week 52	−2.00 (0.15)	−1.75 (0.14)
Change from Week 24 to Week 52 (ATP)	−0.98 (0.13)	−0.72 (0.14)

Data are presented as mean (SE) unless specified

*ATP* active treatment phase, *MMD* monthly migraine days, *MSMD* monthly acute migraine-specific medication treatment days, *SE* standard error, *SD* standard deviation

N1, number of subjects with non-missing percentage change from baseline; % = n/N1 *100;

### P21 Efficacy outcomes in responder and nonresponder patients with episodic migraine treated preventively with erenumab in the STRIVE study

#### Gregor Brössner^1^, Uwe Reuter^2^, Jo H Bonner^3^, David W Dodick^4^, Hernan Picard^5^, Shihua Wen^6^, Shannon Ritter^6^, Jan Klatt^7^, Daniel D Mikol^5^

##### ^1^Headache Outpatient Clinic, Medizinische Universität Innsbruck, Innsbruck, Austria; ^2^Charité Universitätsmedizin Berlin, Berlin, Germany; ^3^Mercy Clinic Neurology and Headache Center, Saint Louis, MO, USA; ^4^Mayo Clinic, Scottsdale, AZ, USA; ^5^Amgen Inc., Thousand Oaks, CA, USA; ^6^Novartis Pharmaceuticals Corporation, East Hanover, NJ, USA; ^7^Novartis Pharma AG, Basel, Switzerland

###### **Correspondence:** Uwe Reuter (Uwe.Reuter@charite.de)

**Background**

Erenumab is a fully human anti-calcitonin gene-related peptide receptor antibody recently approved in the United States and recommended for approval in Europe as a preventive treatment for migraine. STRIVE study demonstrated safety and efficacy of erenumab (70 mg and 140 mg) in patients with episodic migraine (EM) [1]. In clinical practice, patients achieving/not achieving sufficient response to treatment are likely to continue/discontinue treatment; we sought to contextualise the actual treatment benefit among patients achieving (R subgroup) or not achieving (NR subgroup) response at the ≥50% threshold.To evaluate change from baseline in monthly migraine days (MMD), migraine-specific medication treatment days (MSMD), and Migraine Physical Function Impact Diary domain scores for everyday activity (MPFID-EA) and physical impact (MPFID-PI) in R and NR subgroups in STRIVE.

**Methods**

Patients (N=955; aged 18–65 years) with ≥4 and <15 migraine days per month were randomised (1:1:1) to receive subcutaneous erenumab 70 mg, 140 mg, or placebo once monthly for 6 months. The primary endpoint was change from baseline in MMD. The proportion of ≥50% Rs and change from baseline in MSMD, MPFID-EA, and MPFID-PI were prespecified secondary endpoints. All endpoints were evaluated by averaging the monthly treatment effect over Months 4, 5, and 6 of the double-blind treatment phase.

**Results**

In the overall study population, the odds of being a ≥50% R on erenumab were 2.13 (70 mg) and 2.81 (140 mg) compared with placebo. Baseline characteristics were similar in both subgroups (Table 1). In the R subgroup, there was a mean change of −6.0 to −6.1 MMD from a baseline of 8.2 to 8.3. For this, and for the efficacy measures MSMD, MPFID-EA, and MPFID-PI, change from baseline was 2.7- to 7.5-fold greater in Rs than in NRs and 1.4- to 1.9-fold greater in Rs than in the overall population (Table 1). Mean MPFID-EA and MPFID-PI domain scores were reduced by 7–10 points in Rs (differences of ≥3–5 points have been shown to be clinically meaningful).

**Table 1 (abstract P21).** Primary and secondary outcome measures in erenumab-treated responder and nonresponder subgroups in STRIVE

*Erenumab 70 mg*	*Responders (n=135),* *baseline*	*Responders,* *change*	*Non responders (n=161), baseline*	*Non responders change*	*Overall* *(n=312),* *baseline*	*Overall,* *change (mean±SE)*
**MMD**	8.3 ±2.5	−6.1 ±2.1	8.3 ±2.4	−1.1 ±2.8	8.3 ±2.5	−3.4 ±0.2
MSMD	2.9 ±3.3	−2.0 ±2.4	3.8 ±3.5	−0.4 ±2.0	3.2 ±3.4	−1.1 ±0.1
MPFID-EA	13.5 ±8.5	−9.9 ±6.8	14.1 ±8.9	−2.4 ±7.0	14.0 ±8.9	−5.8 ±0.5
MPFID-PI	11.8 ±9.3	−8.4 ±7.00	12.7 ±9.6	−1.1 ±7.7	12.6 ±9.7	−4.4 ±0.5
*Erenumab 140 mg*	*Responders (n=159),* *baseline*	*Responders,* *change*	*Non responders (n=143), baseline*	*Non responders change*	*Overall* *(n=318),* *baseline*	*Overall,* *Change (mean±SE)*
**MMD**	8.2 ±2.4	−6.0 ±2.1	8.5 ±2.5	−1.4 ±2.3	8.3 ±2.5	−3.8 ±0.2
MSMD	3.2 ±3.4	−2.3 ±2.6	3.6 ±3.5	−0.9 ±1.7	3.4 ±3.5	−1.6 ±0.1
MPFID-EA	12.6 ±7.9	−8.9 ±6.8	13.6 ±8.6	−2.5 ±7.0	13.0 ±8.2	−5.8 ±0.4
MPFID-PI	11.5 ±8.3	−7.6 ±7.0	12.8 ±9.7	−1.8 ±8.00	12.0 ±9.0	−4.8 ±0.5

Data are mean ±standard deviation, except as indicated.

*MMD* monthly migraine days, *MSMD* migraine-specific medication treatment days, *MPFID-EA* Migraine Physical Function Impact Diary domain scores for everyday activity, *MPFID-PI* MPFID-physical impairment, *SE* standard error

**Conclusion**

Among patients with EM who achieved ≥50% reduction from baseline in MMD when treated with erenumab (70 mg, 43%; 140 mg, 50%), there were substantial, clinically relevant reductions in the frequency of MMD, MSMD and MPFID scores compared with NRs and the overall patient population. The odds of responding were numerically greater with the 140 mg dose than with the 70 mg dose.

### P22 Efficacy of erenumab in patients with chronic migraine achieving ≥50% response: Subgroup analysis of a double-blind, randomised study

#### David Dolezil^1^, Jan Klatt^2^, Sunfa Cheng^3^, Feng Zhang^3^, Shihua Wen^4^, Shannon Ritter^4^, Daniel D Mikol^3^

##### ^1^Prague Headache Center, DADO MEDICAL s.r.o., Prague, Czech Republic; ^2^Novartis Pharma AG, Basel, Switzerland; ^3^Amgen Inc., Thousand Oaks, CA, USA; ^4^Novartis Pharmaceuticals Corporation, East Hanover, NJ, USA

###### **Correspondence:** Jan Klatt (jan.klatt@novartis.com)

**Background**

Erenumab is a fully human anti-CGRP receptor antibody approved as a preventive treatment for migraine by the US FDA. A 12-week, randomised, double-blind, placebo-controlled study demonstrated efficacy of erenumab (70 mg and 140 mg) in patients with chronic migraine (CM). At Week 12, a greater proportion of patients treated with erenumab achieved ≥50% reduction in MMDs vs placebo (140 mg: 41.2%; 70 mg: 39.9%; placebo: 23.5%). Since in clinical practice, patients achieving/not achieving sufficient response to treatment are likely to continue/discontinue treatment, we sought to contextualise the actual treatment benefit among patients achieving (or not) response at the ≥50% threshold.

**Methods**

Patients (N=667; aged 18-65 years, inclusive) with CM (≥15 headache days/month; ≥8 migraine days/month) were randomised (2:2:3) to receive subcutaneous, once-monthly erenumab 70 mg, 140 mg or placebo. In this subgroup analysis, responders/non-responders were defined by the threshold of ≥50% reduction in MMD, and outcomes were change from baseline to Week 12 in: MMDs, migraine-specific medication treatment days (MSMD), the Headache Impact Test (HIT-6™) scores, Migraine Disability Assessment (MIDAS) scores, and Migraine-Specific Questionnaire (MSQ) scores.

**Results**

Mean (SD) baseline MMDs in the overall study population were 18.0 (4.6). The baseline MMDs in responders and non-responders were comparable. Greater reductions were observed in MMDs in responders (140 mg: −12.5; 70 mg: −12.2) vs non-responders (140 mg: −2.2; 70 mg: −2.6) at both erenumab doses. Similarly, both doses of erenumab showed greater improvements in terms of MSMDs, HIT-6, total MIDAS and MSQ scores in responders than the non-responders (Table 1). Across all outcome measures, change from baseline was 50-100% greater in responders than the overall population and 2-6 times (70 mg: 2-4 folds; 140 mg: 3-6 folds) greater in responders vs non-responders.

**Conclusion**

Among the 39.9%/41.2% of patients with CM treated with erenumab 70 mg/140 mg in this study who achieved ≥50% reduction MMD, there were substantial reductions in the frequency of migraines, the use of migraine-specific medication, and disability as assessed by the HIT-6, MIDAS and MSQ scores compared with non-responders and with the overall patient population. These findings may help to provide context for setting realistic patient expectations for response to treatment with erenumab.

**Table 1 (abstract P22).** Effect of erenumab in patients achieving ≥50% response in MMDs vs non-responders and overall population

Outcome measures	Erenumab 70 mg			Erenumab 140 mg		
	≥50% Responders (N=75)	Non-responders (N=113)	Overall population (N=188)	≥50% Responders (N = 77)	Non-responders (N =110)	Overall population (N=187)
Change from baseline to Week 12						
MMD	−12.2 (2.9)	−2.6 (4.3)	−6.6 (6.1)	−12.5 (4.6)	−2.2 (4.4)	−6.5 (6.8)
MSMD	−5.2 (5.2)	−1.8 (4.1)	−3.3 (4.9)	−6.9 (5.6)	−2.4 (3.9)	−4.3 (5.2)
HIT-6	−10.0 (7.6)	−2.8 (5.1)	−5.7 (7.2)	−10.7 (8.0)	−1.7 (5.1)	−5.5 (7.9)
MIDAS total score	−29.1 (45.4)	−12.6 (41.5)	−19.5 (43.8)	−35.0 (45.2)	−5.8 (38.4)	−18.1 (43.7)
MSQ-RFP	23.0 (19.4)	6.0 (19.8)	13.0 (21.3)	25.7 (23.2)	4.8 (16.2)	13.7 (22.1)
MSQ-RFR	29.7 (18.6)	9.4 (19.8)	17.7 (21.7)	32.7 (23.7)	8.2 (18.5)	18.7 (24.1)
MSQ-EF	30.5 (25.4)	11.1 (26.7)	19.1 (27.8)	33.3 (27.2)	6.3 (19.9)	17.8 (26.8)

Data are expressed as mean (SD)

*HIT-6* Headache Impact Test (higher score indicates worse outcome), *MIDAS* Migraine Disability Assessment (higher score indicates worse outcomes), *MMD* monthly migraine days, *MSQ* Migraine-Specific Quality-of-Life Questionnaire (higher scores indicate better outcomes), *MSQ-RFR* Migraine-Specific Quality-of-Life Questionnaire role function-restrictive, *MSQ-RFP* Migraine-Specific Quality-of-Life Questionnaire role function-preventive, *MSQ-EF* Migraine-Specific Quality-of-Life Questionnaire emotional functioning, *SD* standard deviation

### P23 Long-term safety and tolerability of erenumab: Three-plus year results from an ongoing open-label extension study in episodic migraine

#### Messoud Ashina^1*,^ Peter J. Goadsby^2,^ Uwe Reuter^3,^ Stephen Silberstein^4,^ David Dodick^5,^ Gregory A. Rippon^6^, Jan Klatt^7^, Feng Zhang^6^, Sunfa Cheng^6^, Daniel D. Mikol^6^

##### ^1^Department of Neurology, Danish Headache Center, Rigshospitalet Glostrup, Faculty of Health and Medical Sciences, University of Copenhagen, Copenhagen, Denmark; ^2^NIHR-Wellcome Trust King’s Clinical Research Facility, King’s College London, UK; ^3^Department of Neurology, Charité Universitätsmedizin Berlin, Berlin, Germany; ^4^Jefferson Headache Center, Thomas Jefferson University, Philadelphia, PA, USA; ^5^Department of Neurology, Mayo Clinic, Scottsdale, AZ, USA; ^6^Amgen Inc., Thousand Oaks, CA, USA; 7Novartis Pharma AG, Basel, Switzerland

###### **Correspondence:** Messoud Ashina (ashina@dadlnet.dk)

**Background**: To assess long-term safety and tolerability of erenumab (human anti-CGRP receptor antibody) in patients with migraine after ≥3 years of treatment. Previously published 3-month placebo-controlled and 1-year open-label clinical trial data have provided information on efficacy and safety of erenumab.

**Methods**: Interim analysis from an ongoing 5-year open-label extension (OLE) after all patients completed ≥3 years in the OLE or discontinued the study. Following a 12-week double-blind, placebo-controlled study of erenumab (7 mg, 21 mg, or 70 mg) in adults with episodic migraine, patients could enroll in the OLE, initially receiving erenumab 70 mg monthly. A protocol amendment increased the dosage to 140 mg monthly to assess long-term safety of the higher dose. Safety and tolerability were assessed by monitoring adverse events (AEs), electrocardiograms, laboratory assessments, and vital signs.

**Results**: Of 383 patients enrolled in the OLE, at data cutoff 235 (61.3%) remained in study, all received 140-mg for ≥1 year. Median (Q1, Q3) exposure (70 mg or 140 mg) for all patients enrolled was 3.2 (1.3, 3.4) years. For those continuing in the study, exposure ranged from 3.0 to 3.9 years. Exposure-adjusted AE rate was 132.0/100-patient-years (142.0 prior to and 128.1 following dosage increase). Most frequent AEs (≥4.0/100-patient-years) were viral upper respiratory tract infection, upper respiratory tract infection, sinusitis, influenza, and back pain. Exposure-adjusted serious AE (SAE) rates were 4.2/100-patient-years. One death, which occurred during the first year of the OLE (prior to protocol amendment), was previously reported and was confounded by comorbidities. There was no increase in cardiovascular events over time and no meaningful changes in systolic/diastolic blood pressure or heart rate up to 3.3-years’ follow-up.

**Conclusions**: In this first, and so far only, long-term study of a CGRP-pathway antibody, erenumab was found to be safe and well tolerated with a spectrum and rate of AEs consistent with shorter-term placebo-controlled studies and no dose-dependent AEs.

### P24 Assessment of the long-term safety and efficacy of erenumab during open-label treatment of subjects with chronic migraine

#### Stewart Tepper^1^, Messoud Ashina^2^, Uwe Reuter^3^, Jan L Brandes^4^, David Doležil^5^, Stephen Silberstein^6^, Paul Winner^7^, Feng Zhang^8^, Sunfa Cheng^9^, Daniel Mikol^9^

##### ^1^Geisel School of Medicine at Dartmouth, Hanover, NH, USA; ^2^Department of Neurology, Danish Headache Center, Rigshospitalet Glostrup, University of Copenhagen, Copenhagen, Denmark; ^3^Department of Neurology, Charité Universitätsmedizin Berlin, Berlin, Germany; ^4^Nashville Neuroscience Group and Vanderbilt University Department of Neurology, Nashville, TN, USA; ^5^DADO MEDICAL sro, Prague Headache Center, Prague, Czech Republic; ^6^Jefferson Headache Center, Thomas Jefferson University, Philadelphia, PA, USA; ^7^Premiere Research Institute, Nova Southeastern University, West Palm Beach, FL, USA; ^8^Global Bio statistical Science, Amgen Inc, Thousand Oaks, CA, USA; ^9^Global Development, Amgen Inc, Thousand Oaks, CA, USA

###### **Correspondence:** Uwe Reuter (Uwe.Reuter@charite.de)

**Background**

Erenumab, fully human antibody, has demonstrated efficacy and safety in migraine prevention studies (NCT02066415, NCT02456740). Chronic migraine (CM), the most prevalent type of headache in tertiary care, may require long-term preventive therapy. Here, we report the results from a 1-year open-label extension (OLE) study (NCT02174861) of patients completing a 12-week, placebo-controlled study of erenumab for CM.

**Methods**

In the parent study, CM subjects were randomised (3:2:2) to placebo, erenumab 70 mg, and 140 mg for the 12-week double-blind treatment phase (DBTP), following which eligible subjects could enrol in the 52-week OLE, initially receiving erenumab 70 mg monthly. A protocol amendment increased dose to 140-mg for subjects who had not yet completed Week-28 visit, to assess long-term safety (primary endpoint) of higher dose. Safety was assessed by monitoring adverse events (AEs), electrocardiograms (ECG), laboratory assessments, and vital signs. Secondary endpoints were change from baseline to Week 52 in monthly migraine days (MMD), monthly acute migraine-specific medication days (MSMD), monthly cumulative hours of headache, and proportion of patients achieving ≥50% reduction in MMD. A *post-hoc* analysis on efficacy data, based on last dose received was conducted for subjects who completed the OLE treatment.

**Results**

Of the 609 subjects who were enrolled to OLE study 451 (74.1%) completed the study. Among these, 350 subjects received erenumab 70 mg; 60 received 140 mg; and 199 increased their dose from 70 mg to 140 mg by Week 28. Most subjects were women (83.6%); mean (range) age of patients was 42.5 (18–66) years. Overall, 398/609 (65.4%) subjects reported at least one AE during the OLE for an exposure-adjusted incidence rate of 126.3/100 subject-years. Most frequent AEs (>2.0/100-subject-years) were viral upper respiratory tract infection, upper respiratory tract infection, sinusitis, arthralgia, and migraine (Table 1). Exposure-adjusted treatment-related AE and SAE rates were 20.5 and 3.8/100-subject-years, respectively. There were no clinically meaningful changes from baseline in laboratory values, vital signs, ECG findings, blood pressure or heart rate at any post baseline time point, which appears to be consistent with the DBTP of the parent study. Sustained efficacy was noted for both erenumab doses. Based on the last dose received, a numerically greater benefit in terms of efficacy was observed with erenumab 140 mg compared with the 70 mg dose at both Weeks 40 and 52 in completers (Table 2).

**Conclusions**

Long-term safety data in CM was consistent with the known safety profile of erenumab. Efficacy was sustained throughout the study.

**Table 1 (abstract P24).** Data on primary and secondary outcomes Safety: Exposure-adjusted Subject Incidence Rates of Treatment-emergent Adverse Events Occurring in > 2/100 subject-years for total erenumab (Safety Analysis Set)

Adverse event	Erenumab 70 mg/140 mg (N=609), n (%) / e [r]
Viral upper respiratory tract infection	96 (15.8) / 586.0 [16.4]
Upper respiratory tract infection	45 (7.4) / 624.5 [7.2]
Sinusitis	44 (7.2) / 620.4 [7.1]
Arthralgia	27 (4.4) / 636.7 [4.2]
Migraine	26 (4.3) / 638.5 [4.1]

% = n / N x 100; N = number of subjects exposed to erenumab in OLE; n = number of subjects reporting at least 1 occurrence of an adverse event; r = exposure-adjusted subject rate per 100 subject-years (n / e x 100)

e = sum across all subjects, the total time at risk in the study in years

**Table 2 (abstract P24).** Efficacy ─ 52-week OLE completers

Outcome	Time-point	Erenumab 70-mg (OLE last dose received) (N=266)	Erenumab 140-mg (OLE last dose received) (N=203)
Change from baseline in MMD, mean (95% CI)	Baseline	17.92 (17.38, 18.45)	17.79 (17.16, 18.43)
	Week 40	-7.81 (-8.64, -6.97 ) (n=228)	-9.96 (-10.91, -9.00 ) (n=187)
	Week 52	-8.49 (-9.35, -7.63) (n=214)	-10.48 (-11.52, -9.43) (n=165)
Change from baseline in MSMD, mean (95% CI)	Baseline	10.22 (9.39, 11.06)	8.32 (7.28, 9.35)
	Week 40	-4.68 (-5.33, -4.04) (n=228)	-4.59 (-5.32, -3.85) (n=187)
	Week 52	-5.05 (-5.76, -4.34) (n=214)	-4.97 (-5.81, -4.13) (n=165)
Change from baseline in MSMD in subjects taking MSM, mean (95% CI)	Baseline	12.40 (11.67, 13.13)	11.35 (10.30, 12.40)
	Week 40	-5.63 (-6.33, -4.93)	-6.15 (-7.00, -5.30)
	Week 52	-6.16 (-6.93, -5.40)	-6.71 (-7.67, -5.75)
≥50% responder rate, N1(%)	Week 40	108 (47.4) (n=228)	126 (67.4) (n=187)
	Week 52	114 (53.3) (n=214)	111 (67.3) (n=165)

*CI* confidence interval, *OLE* open label extension, *MMD* monthly migraine days, *MSMD* migraine-specific medication treatment days, *N* number of subjects in the analysis set, *N1* number of responders at corresponding visit, *n* number of subjects with observed data

### P25 Immunogenicity findings from Phase 3 galcanezumab trials in patients with episodic or chronic migraine

#### James M. Martinez, Sandra Garce, Greg Anglin, Michael Hodsdon, William Kielbasa, Brian A Moser, Eric Pearlman

##### Eli Lilly and Company, Indianapolis, IN, USA

###### **Correspondence:** James M. Martinez (martinez_james_michael@lilly.com)

**Background:** To evaluate the immunogenicity profile of galcanezumab, a humanized monoclonal antibody that selectively binds calcitonin gene-related peptide (CGRP) and inhibits its activity, in patients with episodic or chronic migraine.

**Methods:** The Phase 3 migraine program for galcanezumab consisted of 4 studies: the 6-month double-blind (DB), placebo (PBO)-controlled (DB/PC) EVOLVE-1 and EVOLVE-2 studies in episodic migraine, the 3-month DB/PC REGAIN study in chronic migraine (with optional 9-month open-label [OL] extension), and the 12-month OL Study CGAJ in chronic and episodic migraine. Immunogenicity was analyzed using data from the baseline and DB phases of EVOLVE-1, EVOLVE-2, and REGAIN, and from the baseline and OL phases of Study CGAJ. Analyses assessed the incidence of antidrug antibodies (ADA) at baseline, treatment-emergent ADA (TE ADA) and neutralizing ADA (NAb), as well as the time to first occurrence of TE ADA. The effect of ADA titer on both pharmacokinetics (PK) and pharmacodynamics (PD) was assessed from measurements of serum galcanezumab concentrations and plasma CGRP concentrations, respectively. The relationship between ADA status and efficacy was explored using average change in monthly migraine headache day (MHD) in galcanezumab-treated patients. Safety analyses assessed the potential relationship between TE ADA and hypersensitivity events or adverse events (AEs) related to injection sites.

**Results:** The percentage of patients with ADA present at baseline ranged from 6.20% - 11.21% in the galcanezumab group and 5.92% - 8.35% in the PBO group across the 4 studies. The incidence of TE ADA during the specified treatment periods across the 4 studies ranged from 2.57% - 12.40% in the galcanezumab group and 0.45% - 1.66% in the PBO group. The majority of TE ADA in galcanezumab-treated patients were detected approximately 3-6 months after the first dose of study drug. Overall, the observed ADA titer in patients did not impact galcanezumab concentrations, CGRP concentrations, or the efficacy profile of galcanezumab. Hypersensitivity events and AEs related to injection sites were examined in detail, and there was no evidence that such events were mediated by TE ADA.

**Conclusion:** These analyses from the Phase 3 migraine program characterize the immunogenicity profile of galcanezumab treatment in patients with episodic and chronic migraine. In these analyses, immunogenicity did not impact galcanezumab concentrations, CGRP concentrations, or the efficacy profile of galcanezumab. Additionally, TE ADA did not appear to mediate the occurrence of hypersensitivity events and AEs related to injection sites.

**Ethics approval and consent to participate**

Each study has been approved by the relevant institutional Ethics Board. Informed consent to publish has been obtained from all patients who participated in the above studies

**Trial Registration**: NCT02614183 (EVOLVE-1); NCT02614196 (EVOLVE-2); NCT02614261 (REGAIN); NCT02614287 (Study CGAJ).

### P26 Lack of visual paired associative short-term plasticity in migraine patients between attacks

#### Gianluca Coppola^1^, Chiara Abagnale^1^, Federico Ranieri^2^, Clarissa Elizabeth Centurioni^1^, Gabriella Musumeci^2^, Fioravante Capone^2^, Giovanni Di Pino^2^, Vincenzo Parisi^3^, Vincenzo Di Lazzaro^2^, Francesco Pierelli^1,4^

##### ^1^Sapienza University of Rome Polo Pontino, Department of medico-surgical sciences and biotechnologies, Latina, Italy; ^2^Research Unit of Neurology, Neurophysiology, Neurobiology, Department of Medicine, Università Campus Bio-Medico, Rome, Italy; ^3^G.B. Bietti Foundation IRCCS, Research Unit of Neurophysiology of Vision and Neurophthalmology, Rome, Italy; ^4^IRCCS Neuromed, Pozzilli (IS), Italy

###### **Correspondence:** Gianluca Coppola (gianluca.coppola@gmail.com)

**Background**

In healthy controls (HCs), we recently observed that the same time-dependent paired-associative plasticity rules found within the sensorimotor system are valid for the visual system. With the same paradigm of stimulation, here, we have verified whether dysfunctioning associative plasticity might characterize the visual system of episodic migraine without aura (MO) patients, where abnormalities in both inhibitory and excitatory paired-associative sensorimotor plasticity have been previously observed in between attacks (1).

**Materials and methods**

In 15 HCs and in 12 MO patients between attacks, we performed a visual paired associative stimulation (vPAS) protocol by coupling 90 black-and-white checkerboard pattern reversals with low-frequency TMS pulses over the occipital cortex at 2 interstimulus intervals in separate sessions by subtracting or adding 25ms to the visual evoked potential (VEP) P100 latency. We recorded VEPs (600 sweeps) before, after, and 10-min later each vPAS session. VEPs were partitioned in 6 blocks of 100 sweeps. We analysed VEP N1-P1 first block amplitude and delayed habituation.

**Results**

While vPAS-25 significantly enhanced and vPAS+25 reduced VEP amplitude habituation in HCs, they both did not significantly change VEP amplitude habituation in MO between attacks.

**Conclusions**

We provide for the first-time evidence for lack of excitability depressing and enhancing short-term associative plasticity mechanisms within the visual system in migraine between attacks.

**References**

1. Pierelli F, Iacovelli E, Bracaglia M, Serrao M, Coppola G. Abnormal sensorimotor plasticity in migraine without aura patients. Pain. 2013 Sep;154(9):1738-42.

### P27 Effect of OnabotulinumtoxinA on the Frequency and Impact of Headaches in Patients with Chronic Migraine with or without a History of Acute Pain Medication Overuse: Results of the COMPEL Study

#### Stewart J. Tepper^1*^ Maria-Carmen Wilson^2^ John F. Rothrock^3^ Amelia Orejudos^4^ Aubrey Manack Adams^4^ Andrew M. Blumenfeld^5^

##### ^1^Neurology Department, Headache Center, Dartmouth-Hitchcock Medical Center, Lebanon, NH, 03748, USA; ^2^Ochsner Health System, Covington, LA, 70433, USA; ^3^Department of Neurology, George Washington School of Medicine, Washington, DC 20037, USA; ^4^Allergan plc, Irvine, CA, 92612, USA; ^5^Headache Center of Southern California, The Neurology Center, Carlsbad, CA, 92024, USA

###### **Correspondence:** Stewart J. Tepper (Stewart.J.Tepper@Dartmouth.edu)

**Background**: Overuse of acute pain medication by people with chronic migraine (CM) can increase the frequency and intensity of headache. This subanalysis of COMPEL Study data evaluates the relative effect of onabotulinumtoxinA on the frequency and impact of headaches in patients with CM based on history of acute pain medication overuse (MO).

**Methods:** The 108-wk, multicenter, open-label COMPEL Study (ClinicalTrials.gov, NCT01516892) enrolled adults with CM receiving onabotulinumtoxinA 155 U for 9 treatments. Patients completed a daily diary recording headache days for 28 days before the baseline visit and at intervals following treatments 2, 5, 7, and 9. A 6-item Headache Impact Test Questionnaire (HIT-6) was completed at every administration visit. History of MO was defined as use of acute pain medication ≥ 2 times/week in any week with diary data on ≥5 days during the 4-week screening period. Efficacy variables included mean change from baseline in overall number of headache days, number of moderate/severe headache days, and HIT-6 total score at weeks 60 (after 5 treatments) and 108 (after 9 treatments). Observed data are reported. The study received ethical approval from the Institutional Review Board or Independent Ethics Committee at each site.

**Results**: 716 patients were enrolled, 715 of whom had ≥ 1 efficacy analysis and comprised the intention-to-treat (ITT) population: 456 (63.7%) ITT patients had a history of acute MO, 259 (36.3%) did not. Throughout the study period onabotulinumtoxinA treatment showed similar efficacy in both groups, including similar reductions in headache frequency and number of moderate to severe headaches (Table 1). At week 60, 53.3% of patients with a history of MO had a ≥50% reduction in headache days from baseline, as did 55.5% of patients with no history of MO. Additional improvement was seen at week 108: ≥50% reductions were documented in 58.7% of patients with a history of MO and 69.1% of those without. Mean change from baseline (SD) in HIT-6 scores were similar for patients with and without a history of MO at week 60 (−6.8 [6.7] and –6.7 [6.2], respectively) and week 108 (–7.0 [7.19] and –7.2 [7.3], respectively).

**Conclusions**: These results suggest that onabotulinumtoxinA has similar efficacy in patients with or without a history of medication overuse, and that reductions in headache frequency are sustained over time.

**Table 1 (abstract P27).** Mean change from baseline for headache day frequency and number of moderate/severe headaches in onabotulinumtoxinA-treated patients with and without a history of medication overuse

Variable	Medication Overuse (n=456)	No Medication Overuse (n=259)
Headache day frequency, d, mean (SD)		
Week 60	–10.1 (6.8)	–10.3 (7.6)
Week 108	–11.4 (7.2)	–12.5 (7.5)
Moderate/severe headache days, d, mean (SD)		
Week 60	–8.8 (6.1)	–8.7 (6.2)
Week 108	–10.1 (7.0)	–10.0 (7.2)

### P28 Body Mass Index and its relationship with disability, severity, duration and frequency of headaches in female migraine patients

#### Mansouerh Togha, Faraidoon Haghdoost, Faezeh Khorsha, Soodeh Razeghi Jahromi, Zeinab Ghorbani

##### Headache Department, Iranian Center of neurological Research, Tehran University of Medical Sciences, Tehran, Iran

###### **Correspondence:** Faraidoon Haghdoost (faraidoon haghdoost)

**Objectives:** Migraine is a highly prevalent and debilitating neurological disorder. It is most common between the ages 20 and 45, with women predominantly. Several evidences have shown that increased Body Mass Index (BMI) is associated with increased frequency and severity of migraine headaches. The aims of the current study were to evaluate the relation of BMI with disability, severity, frequency and duration of headaches in female migraine patients.

**Methods:** This cross-sectional study evaluated the characteristics of migraine attacks and also MIDAS (Migraine Disability Assessment) score in female migrainures. The diagnosis of migraine was based on ICHD-3 beta criteria. The data on migraine attack characteristics; duration of each attack, frequency, and severity; was recorded on the patients’ diary form that designed by the senior investigator. Visual Analog Scale (VAS), a linear measure of zero without headache to 10 the severest attack, was used for headache severity. Also validated and translated Iranian version of MIDAS questionnaire, a valid and reliable short questionnaire for assessment of headache related disability, was fulfilled by the patients. Height and weight in order to calculate the BMI were measured. BMI was calculated as the weight in kilograms divided by the height in meters squared. Pearson correlation coefficient was used to assess the correlations. Informed consent to publish has been obtained from this patient.

**Results:** In the current study, 170 female migraine patients with the Mean (±SD) age of 34.03±8.03 were enrolled. Of the participants, 67.1% were married, 62.9% were educated at university level and 52.1% declared the association of headaches with menstruation. Mean (±SD) BMI, total MIDAS score, VAS and frequency of headaches were 25.40±4.30, 12.23±6.94, 5.82±2.15 and 9.94±0.64 respectively. BMI was significantly correlated with MIDAS total score (r=0.594, P<0.001), VAS (r=0.516, P<0.001) and frequency of headaches (r=0.500, P<0.001). The correlations remained significant after adjustment for age. No significant correlation was found between BMI and duration of each migraine attacks (r=0.093, P=0.229).

**Conclusion:** This study revealed an association between body mass index and disability, severity and frequency of headaches in female migraine patients. On the other hand, no association was found between headache durations and BMI.

**Key words:** Body mass index, Disability, Headache duration, Headache frequency, Migraine, Visual Analog Scale

### P29 Effect of propranolol in a non-invasive human model of trigeminovascular activation

#### Eloísa Rubio-Beltran, Rianne M. Schoon, Jeffrey vd Berg, Jorie Versmissen, A.H. Jan Danser, Anton H. van den Meiracker, Antoinette MaassenVanDenBrink

##### Division of Pharmacology, Dept. of Internal Medicine, Erasmus University Medical Center, Rotterdam, The Netherlands

###### **Correspondence:** Eloísa Rubio-Beltran (a.rubiobeltran@erasmusmc.nl)

Propranolol is a β-adrenoceptor antagonist that is used for the prophylactic treatment of migraine since many years. However, the mechanism of action of propranolol in preventing migraine attacks has not yet been elucidated. Both a central action, as well as a vascular action (preventing β adrenoceptor‑mediated vasodilatation) have been suggested. In our study, we set out to assess whether propranolol has an inhibitory effect on the trigeminovascular system in our human forehead perfusion model.

We investigated the effect of propranolol (80 mg, 90 min after oral administration, corresponding to the T_max_) on the rise of dermal blood flow (DBF) of the forehead skin (innervated by the trigeminal nerve) by capsaicin application (0.6 mg/ml) and electrical stimulation(0.2-1.0 mA) before and after placebo (grapefruit juice) and propranolol (oral solution with tangerine taste, diluted in grapefruit juice) in a randomized, double-blind, placebo controlled cross-over study, including healthy males (n=11, age±SD: 28±10 yrs) and females (n=11, 25±3 yrs). In addition to the skin dermal blood flow, systolic (SBP) and diastolic blood pressure (DBP) and heart rate (HR) were recorded. As studies were performed in a double-blind manner, the investigator performing the blood flow studies was also blinded for the blood pressure and HR measurements. The study was approved by Medical Ethics Committee from Erasmus Medical Center (MEC 2016-196).

Preliminary results show a significant decrease in SBP (mean±SEM: 109±1 mm Hg vs. 105±2 mm Hg; p<0.001), DBP (mean±SEM: 63±1 mm Hg vs. 61±1 mm Hg; p<0.05) and HR (mean±SEM: 65±2 bpm vs. 58±2 bpm; p<0.001) after propranolol, but not after placebo (p>0.05). Furthermore, DBF responses to capsaicin (mean±SEM: 512.72±33.14 A.U.) were attenuated after propranolol (mean±SEM: 465.67±35.12 A.U., p<0.05) but no after placebo (mean±SEM: 504.8±40.46 A.U.). DBF responses to electrical stimulation were not modified by either placebo or propranolol. When sexes were analyzed separately, propranolol had no effect on the DBF responses to capsaicin in females (mean±SEM: 544.4±32.27 A.U. vs. 535.7±33.22 A.U.; p>0.05), whereas it significantly attenuated the response to capsaicin in males (mean±SEM: 472±54.57 A.U. vs. 394.7±51.09 A.U.; p<0.05).

In conclusion, propranolol 80 mg inhibits the capsaicin-induced increases in DBF, suggesting a modulation of the trigeminovascular system. Moreover, this effect seems to be independent from its direct cardiovascular effects, and was different between sexes, as significant changes were only observed in male subjects. More studies are required to elucidate the mechanism behind this modulation of the trigeminovascular system.

### P30 Effects of subthreshold single pulse Transcranial Magnetic Stimulation (sTMS) on activity of hypothalamic A11 region

#### J. Lloyd^1^, M Jones^2,3^, S McMahon^2^, R Abuukar Abdullahi^1,4^, AP Andreou^1,4^

##### ^1^Headache Research-Wolfson CARD, King’s College London, London, UK; ^2^Neurorestoration Department, Wolfson Centre for Age-Related Diseases, King’s College London, London, UK; ^3^Zenith Neuroteck Ltd, London, UK; ^4^Headache Centre, Guy’s and St Thomas’s NHS Trust, King’s Health Partners, London, UK

###### **Correspondence:** J. Lloyd (k1633995@kcl.ac.uk)

**Objectives**

Migraine pathophysiology has been shown to involve altered activity of hypothalamic region. The dopaminergic A11 nucleus appears to be of particular interest. Single-pulse transcranial magnetic stimulation (sTMS) is a non-invasive neuromodulation technique shown to be a successful acute preventative treatment for migraine patients. sTMS uses a single magnetic pulse of 170 μs duration to induce weak electrical currents to the cortex via electromagnetic induction. The aim of this experiment was to investigate if sTMS could affect the neuronal activity of dopaminergic cells in the A11 nucleus.

**Methods**

All procedures were performed under a UK Home Office Licence in accordance to the 1986 Animal (Scientific Procedures) Act in anaesthetised male adult Sprague-Dawley rats. Tungsten microelectrodes were used for extracellular recordings from the A11 nucleus. Spontaneous neuronal activity of the A11 nucleus was recorded following induction of a cortical spreading depression (CSD) induced by pinprick at the occipital cortex, or following application of sTMS pulses (~1.1T) applied to the visual cortex from a custom made sTMS coil (11 mm diameter; rise time 170 us). In the latter group, an sTMS pulse was applied every 10 min. Post-hoc spike analysis was used to isolate dopaminergic firing and data were compared to baseline spontaneous firing.

**Results**

A single 600 V sTMS pulse was found to have a significant effect on spontaneous dopaminergic firing of the A11 region. This effect was seen within a minute of application of the pulse (t (17) = 3.129, p<.05). In 50% of the cells recorded sTMS facilitated spontaneous firing and in the remaining it inhibited neuronal firing. In addition, repeating the sTMS pulse was found to have a cumulative effect on the firing of the A11 nucleus.

Likewise in the first minute following application of Cortical Spreading Depression there was a reduction in A11 nucleus activity (t (6) = 3.021, p<.5), which returned to baseline within 10 min.

**Conclusions**

CSD, as a vast cortical event, can alter the spontaneous neuronal firing in the A11 nucleus, suggesting at least an indirect cortical-subcortical networks communication. A single TMS pulse, as a cortical treatment, can alter the as well the firing rate of the dopaminergic A11 nucleus, suggesting that such a treatment could indirectly influence hypothalamic regions believed to be involved in the triggering of a migraine attack. Such alterations in the hypothalamic region be sTMS may be involved in the preventive mechanism of sTMS in migraine.

### P31 Intracranial pressure: a comparison of the non-invasive HeadSense monitor vs. lumbar pressure measurement

#### Jeppe Hvedstrup^1^, Aleksandra Radojicic^2,3^, Walid Moudrou^4^, Martin W Herklots^5^, Anton Wert^6^, Manfred Holzgraefe^6^, Mark Obermann^6,7^, Guus G Schoonman^5^, Rigmor H Jensen^2^, Henrik W Schytz^1^

##### ^1^Headache Diagnostic Laboratory, Danish Headache Center and Department of Neurology, Rigshospitalet-Glostrup, faculty of health and sciences, university of Copenhagen, Denmark; ^2^Danish Headache Center and Department of Neurology, Rigshospitalet-Glostrup, Faculty of Health and Medical Sciences, University of Copenhagen, Denmark; ^3^Neurology Clinic, Clinical Center of Serbia, Belgrade, Serbia; ^4^Department of Neurology, Maastad Hospital, Rotterdam, The Netherlands ^5^Department of Neurology, Elsabeth-Tweesteden Hospital, Tilburg, The Netherlands ^6^Center of Neurology, Asklepios Hospitals Schildautal, Seesen, Germany; ^7^Department of Neurology, university Hospital Essen, University of Duisburg-Essen, Germany

###### **Correspondence:** Jeppe Hvedstrup (jeppe.hvedstrup.mann@regionh.dk)

**Background:** Intracranial pressure (ICP) is essential in monitoring and as a tool to diagnose intracranial hypertension. A method for measuring non-invasive ICP (nICP) has been developed and showed promising results in intensive care patients when comparing the method to invasive ICP monitoring. The nICP uses mixed transcranial acoustic (TCA) signals generated via an acoustic signal transmitted through the cranium and detected in the opposite ear using an acoustic sensor.

**Objective:** To compare nICP with conventional lumbar puncture opening pressure (LP-ICP) in patients investigated at neurologic departments.

**Hypothesis:** nICP values using mixed TCA method correlate with LP-ICP values.

**Design:** A multicenter study of patients undergoing lumbar puncture for diagnostic purpose at neurological departments were included. Each patient underwent LP-ICP and nICP with HeadSense^©^ equipment. The HeadSense nICP measurements were conducted with the patients’ head in a 30-degree tilt and in supine position right before and after the lumbar puncture. All the collectors of the mixed TCA nICP data were blinded to the LP-ICP data. The primary endpoint was the correlation between the nICP in the supine position before the lumbar puncture and the LP-ICP measurement.

**Results:** No correlation between the supine nICP before the lumbar puncture and the LP-ICP was found (*r* = -0.211 & *P* = 0.358). The nICP in 30-degree head-tilt correlated with the supine nICP before the lumbar puncture (r = 0.830 & *P* < 0.01). The supine nICP before and after the lumbar puncture did not correlate (*r* =0.056, *P* = 0.831). Furthermore, nICP showed values below 15 mmHg in the three patients with LP-ICP over 20 mmHg.

**Conclusion:** This is the first study to compare nICP using mixed TCA signals with LP-ICP in a neurological department setting. The nICP signals were not reliable and high ICP values were missed with the new nICP method. Thus, further development of the mixed TCA nICP is warranted.

### P32 Triptans use in post-dural puncture headache

#### Federico Mainardi^1^, Giorgio Zanchin^2^, Carlo Lisotto^3^, Ferdinando Maggioni^2^

##### ^1^Headache Centre, Neurological Division, SS Giovanni e Paolo Hospital, Venice, Italy; ^2^Headache Centre, Department of Neurosciences, Padua University, Padua, Italy; ^3^Headache Centre, San Vito al Tagliamento hospital, San Vito al Tagliamento, Italy

###### **Correspondence:** Federico Mainardi (fmainardi@iol.it)

**Introduction**: Post-dural puncture headache (PDH) is classified in chapter 7 - *Headache attributed to non-vascular disorders* as a subtype of *Headache attributed to low cerebrospinal fluid pressure* (code 7.2) in the current International Classification of Headache Disorders 3 ed (ICHD 3) [1]. The occurrence of PDH widely ranges from 1 to 40%, being linked to variable as needle gauge and orientation and operator skill level [2]. Accepted standard treatment for PDH is lacking. Albeit maintenance of the supine position as long as PDH lasts and mild analgesics when necessary are usually sufficient to control the pain, some patients require additional strategies. The use of triptans in treating PDH has been proposed without conclusive evidence of efficacy.

**Material and methods**: We reviewed the literature on the topic with the aim to assess the efficacy of triptans in the acute and prophylactic treatment of PDH. Triptans were used as abortive therapy in six papers [3-8], whereas two papers investigated their efficacy as a preventive option [9,10].

**Results**: *Acute treatment*: in a series of 30 patients, Zolmitritpan 2.5 mg resulted significatively superior versus placebo in relieving PDH at 6 (z:60%, p:36%;),12 (z:70%, p:46%) and 24 hours (z:86%, p:63%) [3]. Sumatriptan 6 mg sc was effective in 9 up to 14 patients who developed PLH [4-8]; in those with a positive response, headache recurred within 24 h in three cases. *Prophylactic treatment*: Frovatriptan 2.5 mg/die for 5 days was administrated in 50 patients after lumbar puncture; among them, a mild isolate headache episode was referred in five cases in the first day of treatment, while a persistent headache for the first 2 days was reported by two patients. In the second study, sumatriptan 25 mg 4 doses/day resulted superior to placebo in preventing the occurrence of PSD in the next 48 h from the induction of spinal anesthesia [10].

**Conclusion**: Albeit no definitive data are available, triptans appear to be more useful in the prophylaxis of PDH than in its acute treatment. More studies on the topic are needed.

**References**

1. Headache Classification Committee of the International Headache Society (IHS). The International Classification of Headache Disorders, 3^rd^ edition. Cephalalgia 2018; 38: 1-211.

2. Basurto Ona X, Osorio D, Bonfill Cosp X. Drug therapy for treating post-dural puncture headache. *Cochrane Database of Systematic Reviews* 2015, Issue 7. Art. No.: CD007887. DOI:10.1002/14651858.CD007887.pub3.

3. Riaz A, Rao ASK, Sharif A. Zolmitriptan is effective in relieving post-dural puncture headache in young parturients. Anesth Pain & Intensive Care 2014; 18: 147-151.

4. Carp H, Singh PJ, Vadhera R, Jayaram A. Effects of the Serotonin-Receptor Agonist Sumatriptan on Postdural Puncture Headache: Report of Six Cases. Anesth Analg 1994; 79: 180-182.

5. Connelly NR, Parker RK, Rahimi A, Gibson CS. Sumatriptan in Patients with Postdural Pouncture Headache. Headache 2000; 40: 316-319.

6. Hodgson C, Roiberg-Henry A. The use of sumatriptan in the treatment of postdural headache. Anaesthesia 1997; 52: 808.

7. Lhuissier C, Mercier FJ, Dounas M, Benhamou D. Sumatriptan: an alternative to epidural blood patch? Anaesthesia 1996; 51: 1078.

8. Sprigge JS. The use of sumatriptan in the treatment of postdural puncture headache after accidental lumbar puncture complicated a blood patch procedure. Anaesthesia 1999; 54: 95-86.

9. Bussone G, Tullo V, d’Onofrio F, Petretta V, Curone M, Frediani F, Tonini C, Omboni S. Frovatriptan for the prevention of postdural puncture headache. Cephalalgia 2007; 27: 809-813.

10. Ghanei M, Rahmanian K, Jahromi AS, Sahraei R. Effect of Sumatriptan on Postdural Puncture Headache. Biomedical & Pharmacology Journal 2016; 9: 735-738.

### P33 A case of autoimmune encephalitis preceded by posterior reversible encephalopathy syndrome and reversible cerebral vasoconstriction syndrome

#### Jaeho Kim, Mi Ji Lee

##### Department of Neurology, Samsung Medical Center, Sungkyunkwan University School of Medicine, Seoul, Republic of Korea

###### **Correspondence:** Mi Ji Lee (mirony.lee@gmail.com)

**Background & Significance**: Posterior reversible encephalopathy syndrome (PRES) is typically characterized by headache, altered mental functioning, seizures, and visual loss associated with imaging findings of bilateral subcortical and cortical edema with a predominantly posterior distribution. We report a case of patient of Autoimmune encephalitis preceded by PRES and reversible cerebral vasoconstriction syndrome (RCVS).

**Case**: A 31-year-old woman presented with thunderclap headache after 1 week of paroxetine and alprazolam treatment due to new onset psychiatric symptoms. Brain MRI showed concomitant PRES and RCVS. After 1 week of admission, she developed repeated generalized tonic-clonic seizures. In addition to epileptic seizures, she had memory impairment, mood fluctuation, dullness, and psychiatric symptoms. Follow up brain MRI showed a marked improvement of PRES and RCVS, but persistent hyperintensities and swelling in the bilateral medial temporal areas. The patient underwent CSF autoimmune antibody test, which revealed anti-LGI1 antibody. Finally, she was diagnosed with LGI1 autoimmune encephalitis. After treated with steroid and antiepileptic drug, her symptoms have been improved, and she returned to work.

**Conclusions**: Autoimmune encephalitis can present with PRES and RCVS. Persistent encephalopathic symptoms, imaging abnormalities in medial temporal lobes, and autoantibody testing can serve as clues suggesting autoimmune encephalitis. Appropriate treatment such as immunotherapy and/or antiepileptic treatment can lead to excellent neurological recovery.

**Consent for publication**: Informed consent to publish has been obtained from this patient

**Fig. 1 (abstract P33).**
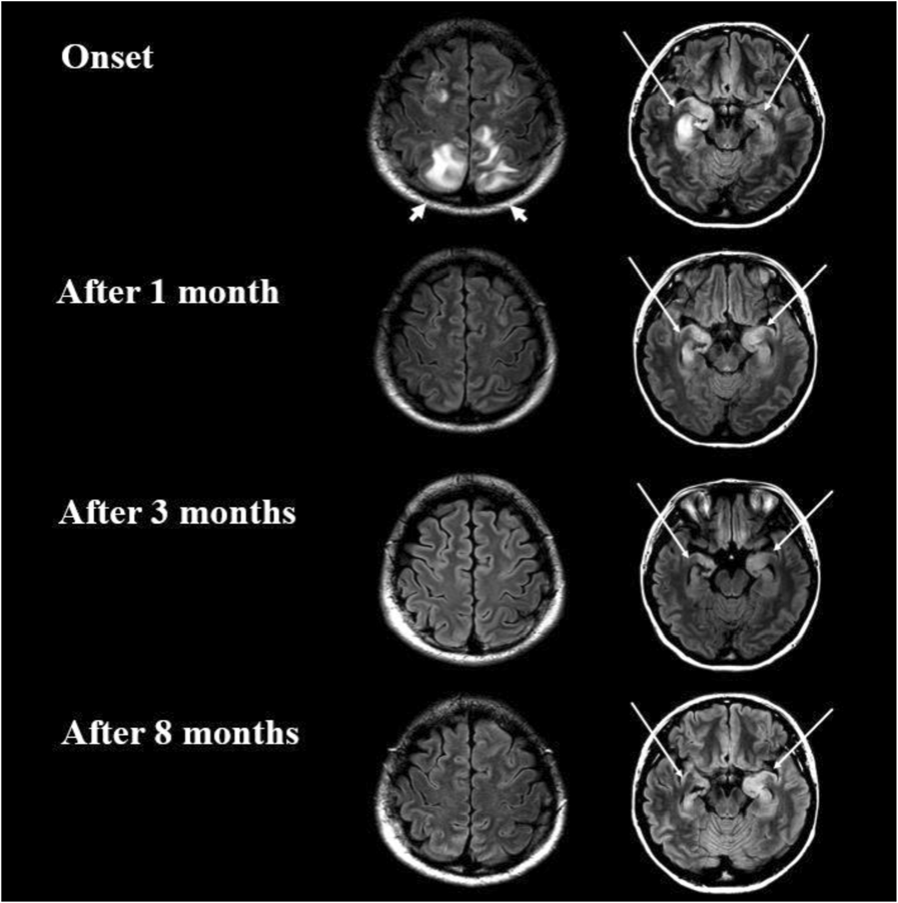
Brain MR findings show PRES (short arrows) and persistent high signal change in bilateral hippocampi and progressive atrophy in the right medial temporal structures (long arrows).

### P34 New insights in post-traumatic cluster headache through a cohort study

#### Lou Grangeon^1,2^, Emer O’Connor^1^, Layan Akijian^1^, Thanh Mai Pham Ngoc^3^, Manjit Matharu^1^

##### ^1^Headache Group, Institute of Neurology and The National Hospital for Neurology and Neurosurgery, Queen Square, London; ^2^Department of Neurology, Rouen University Hospital, 76031, Rouen, France; ^3^Mathematics Institute of Orsay, Paris Sud University, CNRS, 91405 Orsay, France

###### **Correspondence:** Manjit Matharu (m.matharu@uclmail.net)

**Background:** Very few cases of cluster headache (CH), one of the most painful conditions known to humans, have been reported following head injury, leading to loss of work capacity and significant fiscal consequences. This study attempts to investigate the characteristics of post-traumatic CH (PTCH) and to compare its severity to primary CH

**Methods:** A retrospective cohort study was conducted in a tertiary headache centre at the National Hospital for Neurology and Neurosurgery (Queen Square, London, UK) between 2007 and 2017. All consecutive patients diagnosed with chronic or episodic CH that developed within 7 days of head trauma were assessed. A control cohort of 553 CH patients

included all patients who attended the headache clinic during the same period and who fulfilled the criteria for primary CH without any previous history of head trauma. Demographics of PTCH patients, characteristics of PTCH attack, concomitant headache, response to treatment and cause and mechanism of head trauma. Multivariate analysis was performed using logistic regression and resorting to the powerful Elastic net algorithm for variable selection.

**Results:** 26 PTCH patients were identified. Approximately 84% were diagnosed with chronic CH and 55% responded poorly to preventive treatment. Five patients suffered from concomitant chronic migraine, four of whom developed it after head trauma as well. The CH attacks were ipsilateral to the injury in all patients. According to multivariate analyses, significant association was found between PTCH and familial history of CH (OR 2.32; 95% CI, 1.4 - 3.8), chronic form (OR 1.53; 95% CI, 1.0 – 2.2), parietal location (OR 3.9; 95% CI,

2.5 – 6.1), and presence of eye oedema during attacks (OR 1.53, 95% CI, 1.0 – 2.2). PTCH patients were at a higher risk of being intractable to acute (OR 2.1, 95% CI, 1.0 – 4.6) and preventive (OR 4.9, 95% CI, 3.0 – 8.2) treatment and of suffering from associated chronic migraine (OR 5.59; 95% CI, 3.0 - 10.4).

**Conclusion:** This largest series of PTCH defines it as a unique entity with specific evolutive profile. After comparison to a large cohort of primary CH, we demonstrated that PTCH is more severe with more chronic forms, marked autonomic features, higher risk of intractability to treatment and associated chronic migraine in patients with family history of CH. This highlights the requirement for individualized care.

**Fig. 1 (abstract P34).**
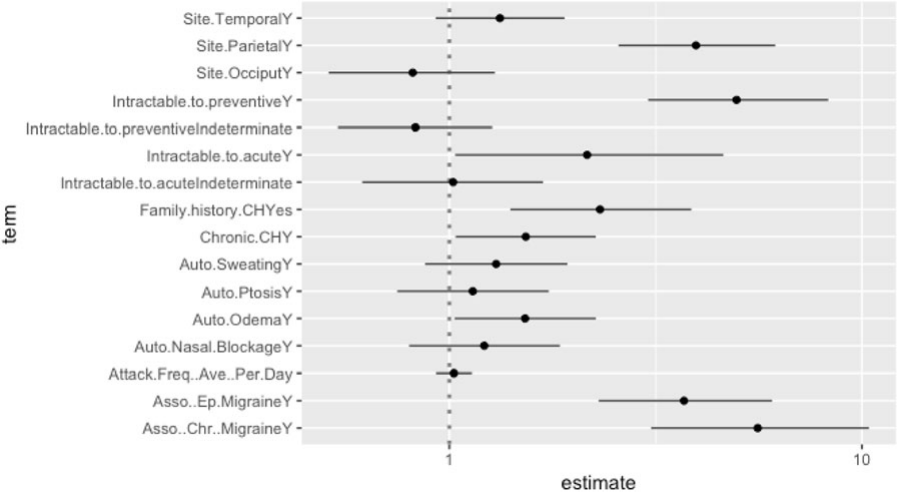
Estimate odds-ratio of each selected item entered into the logistic regression model (n=16)

**Table 1 (abstract P34).** Characteristics of PTCH according to multivariate logistic regression model

Predictive factor	OR	95% CI	p value
Family History of CH	2.32	1.41 - 3.86	0.001
Chronic CH	1.53	1.04 - 2.26	0.032
Parietal location	3.96	2.57 - 6.17	<0.001
Presence of eye oedema	1.53	1.03 - 2.27	0.035
Associated Chronic Migraine	5.59	3.08 - 10.40	< 0.001
Associated Episodic Migraine	3.71	2.30 - 6.05	< 0.001
Intractable to Acute Treatment	2.16	1.03 - 4.61	0.043
Intractable to Preventive Treatment	4.97	3.03 - 8.28	< 0.001

*CI* Confidence interval, *OR* Odds-ratio

### P35 The relevance of associated symptoms of migraine according to migraine subtypes: A Clinical study

#### Aynur Özge^1^, Osman Özgür Yalın^2^, Derya Uludüz^3^, Özlem Mercan^3^, Mehmet Ali Sungur^4^, Aksel Siva^3^

##### ^1^Mersin University School of Medicine, Professor, Neurology Department, Mersin, Turkey; ^2^University of Health Sciences, Istanbul Training and Research Hospital, MD, Neurology Department, İstanbul, Turkey; ^3^Istanbul University Cerrahpasa School of Medicine, Associate Professor, Neurology Department,İstanbul, Turkey; ^4^Düzce University School of Medicine, Professor, Biostatistics Department, Düzce, Turkey

###### **Correspondence:** Osman Özgür Yalın (osmanozguryalin@yahoo.com)

**Background:** Migraine is a primary headache disorder with various associated symptoms. Nausea, vomiting, photophobia, phonophobia, osmophobia and allodynia can be present with different frequency in migraine subtypes. Clinical symptoms such as allodynia and osmophobia are not routinely evaluated during clinical visits even though their diagnostic and therapeutic implications are known. We aimed to observe the association with clinical symptoms and headace intensity and frequency in patients with migraine subtypes.

**Methods:** The study was based on the Turkish Headache Database Study group electronically recorded patient data. Database includes about 20.000 patient records from tertiary headache clinics since 20 years. Diagnosis of headache is based on the International Classification of Headache Disorders (ICHD) 3 beta. Patients were classified according to ICHD as; episodic migraine and chronic migrane with or without aura. Patients were asked if they experienced photophobia, phonophobia, osmophobia and allodynia during migraine attack. The results were compared within each subgroups.

**Results:** Totally 1935 patients were enrolled. Chronic migraine was diagnosed in 24.8%, episodic migraine in 75.2%, migraine without aura in %60.7 and migraine with aura in 39.3% of the patients. Osmophobia and allodynia were significantly more frequent among chronic migraine patients (p=0.001). The presence of photophobia and phonophobia were not differ in subgroups except photophobia was more frequent in patients with migraine with aura (p<0.001). We analyzed effects of associated symptoms within chronic migraine patients. Photophobia and phonophobia were positively correlated with the headache frequency, severity and headache duration within the group, whereas no correlation was found for osmophobia and allodynia.

**Conclusions:** We found an association between allodynia, osmophobia and headache chronicity. Awareness of those symptoms and understanding of revolution of migraine in the term of time will provide new insight for the management of disease. Future research should further elucidate these relationships and focus on prevention of migraine patients from chronification.

### P36 Descriptive analysis of a population with chronic migraine in a tertiary Headache Center

#### Ana-Inês Martins, Joana Ramos-Lopes, Pedro Lopes, Bruno Silva, Sónia Batista, Lívia Sousa, Isabel Luzeiro

##### Neurology Department, Coimbra University and Hospital Centre, Coimbra, Portugal

###### **Correspondence:** Ana-Inês Martins (ana.inesm@hotmail.com)

**Background**: Chronic migraine (CM) is the 5th most prevalent disease and has a massive impact in individual quality-of-life and global economy. Although there are several international studies about this topic, in Portuguese population that analysis is scarce. We aim to describe a population of chronic migraine patients followed in a tertiary Headache Center.

Patients diagnosed with CM and followed in our tertiary hospital center were selected and posteriorly interviewed.

**Results**: A total of 201 patients were enrolled, mean age 44.98 (±13.20) years, 190 females (94.5%), mean BMI 24.30 (±3.59). Of those, 50.7% (N=102) lived in an urban area and 152 patients were professionally active. The mean age of symptoms onset was 22.71 (±9.82) years and the diagnosis of CM was made on average at 32.05 (±12.05) years, which was mainly established by a Neurologist (72.5%). At the time of diagnose, mean days of headache per month in the previous year was 17.82 (±4.73). After initiating preventing treatment mean days of headache per month decreased to 8.82 (±5.10). Phonophotophobia was the most common symptom accompanying migraine (90.2%). Moreover, in our sample there was a loss of 58 days from paid work in the preceding 3 months, a mean of 0.29 per person. Acute phase treatment comprised mainly non-steroidal inflammatory drugs, which are used for 82.6% of patients, followed by triptans (43.3%) and analgesics (32.3%). Regarding preventive treatment, the majority of patients are under two or more different pharmacological classes (57.2%, N=115). Most patients are under an anti-epileptic drug (50.7%,N=102). The second most used preventive drug class is beta-blockers (38.3%, N=77), closely followed by selective serotonin re-uptake inhibitors (35.3%, N=71). The least used classes are toxin botulinum (32.1%, N=64) and tricycle antidepressants (28.9%, N= 58).

**Conclusions**: Our work adds further evidence of epidemiological and clinical data about CM in Portugal. Prevention of migraine chronification is essential and requires adequate treatment of individual migraine attacks, early initiation of preventive medication and avoiding analgesic overuse.

**Ethics Approval**

The study was approved by Coimbra University and Hospital Centre Institutution‘s Ethics Board, approval number CHUC-106-17.

### P37 WHITE MATTER LESIONS IN CHRONIC MIGRAINE ARE RELATED WITH MOLECULAR MARKERS OF INFLAMMATION AND BLOOD BRAIN BARRIER DISRUPTION

#### C. Dominguez^1^, M. Saavedra^1^, X. Rodríguez-Osorio^1^, T. Sobrino^2^, F Campos^2^, J. Castillo^2^, R. Leira^1,2^

##### ^1^Servicio de Neurología, Hospital Clínico Universitario de Santiago de Compostela, Santiago de Compostela, 15706, Spain; ^2^Laboratorio de Investigación de Neurociencias Clínicas, Instituto de Investigación Sanitaria (IDIS). Santiago de Compostela, 15706, Spain

###### **Correspondence:** C. Dominguez (claravivero@hotmail.com)

**Background:** White matter lessions (WML) have been described in migraine patients, but their origin and role in migraine pathophysiology is still under discussion. In this study we aim to determine the relation between WML and levels of molecular markers of inflammation, endothelial dysfunction, blood brain barrier disruption and brain damage in chronic migraine (CM).

**Methods:** Prospective study including 62 patients with CM (IHS 2013) (41.4±11.1 years old; 89,5% women). The study was approved by the Galicia Autonomic Investigation Ethics Board (number 2016/079). All subjects underwent a clinical, molecular and neuroimaging protocol, including an 3T MRI study (T1,T2,T2* and FLAIR) . White matter hyperintensities were analysed and clasified in number (<3, 4-6, >6) and location (subcortical, periventricular or other). Plasma levels of biomarkers of inflammation: interleukin 6 (IL-6), interleukin 10 (IL-10), high-sensitivity C-reactive protein (hs-CRP), calcitonin gene-related peptide (CGRP); endothelial dysfunction: pentraxin 3 (PTX3), soluble TNF-like weak inducer of apoptosis (sTWEAK); blood-brain barrier disruption: cellular fibronectin

(cFN) and brain damage: s100 calcium binding protein (s100B) and neuron-specific enolase (SNE) were determined by ELISA in peripheral blood during interictal periods.

**Results:** 37 patients with CM (59.6%) showed WML in MRI. WML were related with higher levels of IL-6 (9.2±2.9 vs 7.5 ±2.8 pg/mL; p=0.001), cFN (17.4 ±6.2 vs 10.9 ±5.2 microgr/mL p=0.008 ) and SNE (18.7 ±5.1 vs 15.9 ±4.8 ng/mL). Logistic regression analysis showed an independent correlation between WML and levels of IL-6 (OR 1.2 95%CI 1.0-1.5; p=0.049) and cFN (OR 1.1, 95% CI 1.0-1,2; p=0.021).

**Conclusions**: High levels of biomarkers of inflammation and BBB disruption are associated with WML in patients with CM, suggesting a role of these mechanisms in white matter damage in CM.

**Funding**

This work was funded by project PI15/01578; from the State Plan of I+D+I 2013-2016 and co-funded by the ISCIII-Subdirección General de Evaluación y Fomento de la Investigación el Fondo Europeo de Desarrollo Regional (FEDER).

### P38 IRON DEPOSITS IN PERIAQUEDUCTAL GRAY MATTER ARE RELATED WITH POOR RESPONSE TO ONABOTULINUMTOXIN A IN CHRONIC MIGRAINE

#### C. Dominguez^1^, M. Saavedra^1^, X. Rodríguez-Osorio^1^, C. Villalba^2^, P. Ramos-Cabrer^3^, T. Sobrino^4^, F Campos^4^, J. Castillo^4^, R. Leira^1,4^

##### ^1^Servicio de Neurología, Hospital Clínico Universitario de Santiago de Compostela, Santiago de Compostela, 15706, Spain; ^2^Servicio de Radiología, Hospital Clínico Universitario de Santiago de Compostela, Santiago de Compostela, 15706, Spain; ^3^Molecular Imaging Unit. CIC biomaGUNE. Donostia. Ikerbasque, Basque Foundation for Science. Bilbao , Spain; ^4^Laboratorio de Investigación de Neurociencias Clínicas, Instituto de Investigación Sanitaria (IDIS). Santiago de Compostela, 15706, Spain

###### **Correspondence:** C. Dominguez (claravivero@hotmail.com)

**Background:** Response to treatment with Onabotulinumtoxin A (OnabotA) in chronic migraine (CM) has been related with several clinical features and molecular markers. Our aim is to determine if there is any relation between MRI evidence of iron deposits in deep brain nuclei and periaqueductal grey matter (PAG) and efficacy of treatment with OnabotA.

**Methods:** Prospective study including 62 patients with CM (IHS 2013) (41.4±11.1 years old; 89,5% women) that were treated with OnabotA ( PREEMPT protocol). The study was approved by the Galicia Autonomic Investigation Ethics Board (number 2016/085). All subjects underwent a 3T MRI (T1,T2,T2* and FLAIR) at baseline. Volume of hipointense areas in deep brain nuclei and PAG was measured. Patients were classified in responders (reduction of more than 50% in headache frequency) and non-responders (reduction of less than 50% in headache frequency) after one year of treatment.

**Result**s: 47 patients (75.8%) responded to treatment with OnabotA. Non-responders showed larger volumes of iron in *globus pallidus (GP)* (2495,5 ± 1852,3 vs 1691,7±1000,3 microl; p=0.004) and PGA (405,7±55.1 vs 325,8±64.1 microl; p=0.001) when compared to responders. There was a negative correlation between volume of iron deposits in GP (r -0.270; p=0.040) and PGA (r -0..430; p<0.001) and response to OnabotA. After performing a logistic regression analysis, iron deposits in PGA were independently associated with poor response to OnabotA (OR 0.973 [0.955-0.991 95% IC; p=0.04).

**Conclusions**: Larger iron deposits in PGA are related with poor response to treatment with OnabotA in CM.

**Funding**

This work was funded by project PI15/01578; from the State Plan of I+D+I 2013-2016 and co-funded by the ISCIII-Subdirección General de Evaluación y Fomento de la Investigación el Fondo Europeo de Desarrollo Regional (FEDER).

### P39 Economic impact of migraine in the EU5: a matched analysis of the NHWS 2017 data on work productivity and healthcare resource use

#### Michael J. Doane^1^, Pamela Vo^2^, Aikaterini Bilitou^3^, Juanzhi Fang^4^, Annik K-Laflamme^2^, Shaloo Gupta^5^

##### ^1^Kantar Health, Horsham, Pennsylvania, USA, 19044; ^2^Novartis Pharma AG, Kohlenstrasse, Basel, Switzerland, 4056;^3^Novartis Global Services Centre, Dublin, Ireland, D04A9N6; ^4^Novartis Pharmaceuticals Corporation, East Hanover, New Jersey, USA, 07936; ^5^Kantar Health, Madison Ave, New York, USA, 10010

###### **Correspondence:** Pamela Vo (pamela.vo@novartis.com)

**Introduction**

Migraine is a distinct neurological disease ranking among the top causes of disability globally [1].

**Objectives**

This study aimed to describe the incremental burden of migraine on work productivity and healthcare resource utilization (HRU) in those suffering from ≥4 monthly headache days (HDs) in Europe.

**Methods**

A retrospective, cross-sectional analysis of the 2017 National Health and Wellness Survey (NHWS) data from the EU5 (France, Germany, Italy, Spain, and UK) was conducted. Outcomes from respondents who self-reported a doctor diagnosis of migraine, experienced at least one migraine during the prior month and overall ≥4 HDs during the prior month, were stratified by HD frequency (i.e., 4-7, 8-14 and ≥15 HDs) and matched by propensity scores within each subgroup and country using sociodemographic characteristics to respondents without migraine (controls). Work and activity impairment was assessed via Work Productivity and Activity Impairment Questionnaire – General Health version (WPAI-GH), and HRU via healthcare provider (HCP) visits, emergency room (ER) visits, and hospitalizations during the prior 6 months of survey completion. Mann-Whitney U tests were used for continuous and Chi-square tests were used for categorical variables to determine significant differences between subgroups.

**Results**

Analyses of the propensity score-matched sample of 1569 respondents with migraine (4-7, 8-14 and ≥15HDs/month) showed that a significantly higher proportion of patients reported at least one visit to a HCP, neurologist, ER or being hospitalized in the prior 6 months compared with matched controls (Table 1). WPAI outcomes were also significantly impacted across all migraine subgroups compared with controls.

**Conclusions**

Migraine patients across all migraine frequency subgroups reported significantly higher HRU and work impairment compared with matched non-migraine controls. This study highlights the economic implications of migraine to the healthcare system and society.

**Ethics Approval**

The NHWS received approval from the Pearl Institutional Review Board. All NHWS respondents provided informed consent prior to participating.

**References**

1. Steiner TJ, Stovner LJ, Vos T, Jensen R, Katsarava Z. Migraine is *first* cause of disability in under 50s: will health politicians now take notice? *The Journal of Headache and Pain*. 2018;19(1):17.

**Table 1 (abstract P39).** HRU and WPAI outcomes after propensity score matched analysis*

	Non-migraine (N=1,569)	4-7 HDs (N=783)	8-14 HDs (N=429)	>=15 HDs (N=357)
HRU in the past 6 months	% proportion of patients with at least 1 visit (N)			
Visited any HCP	^85.1%^a ^(1336)^	^96.0%^b ^(752)^	^95.6%^b ^(410)^	^94.1%^b ^(336)^
Visited ER	^11.9%^a ^(187)^	^23.0%^b,c ^(180)^	^21.7%^b ^(93)^	^27.7%^c ^(99)^
Visited Neurologist	^4.2%^a ^(66)^	^12.4%^b ^(97)^	^14.5%^b ^(62)^	^24.4%^c ^(87)^
Hospitalized	^7.8%^a ^(123)^	^11.9%^b ^(93)^	^14.9%^b ^(64)^	^15.4%^b ^(55)^
WPAI outcome in the past 7 days	Average % work impairment* (N)			
Absenteeism	^8.47%^a ^(870)^	^11.28%^b ^(447)^	^13.34%^b,c ^(264)^	^19.13%^c ^(166)^
Presenteeism	^20.54%^a ^(849)^	^32.11%^b ^(431)^	^33.74%^b ^(254)^	^43.51%^c ^(154)^
Overall Work Impairment	^22.62%^a ^(841)^	^34.77%^b ^(427)^	^36.95%^b ^(251)^	^47.20%^c ^(154)^

*Propensity matching was conducted using patient demographic variables identified in pre-matched bivariate results (i.e., age, gender, employment status, marital status, income, education, smoking, alcohol use, body mass index, exercise and the Charlson comorbidity index). Subscripts refer to pairwise comparisons using chi-square tests for HRU and Mann-Whitney U tests for WPAI outcomes. If subscripts are different between categories, then statistical significance (p<.05) is indicated.

*These metrics are only reported of those currently employed; Non-Migraine (N=957), 4-7 HDs (N=481), 8-14 HDs (N=281) and

>=15 HDs (N=176)

### P40 Humanistic burden of migraine in the EU5: a matched analysis of the NHWS 2017

#### Michael J. Doane^1^, Pamela Vo^2^, Aikaterini Bilitou^3^, Juanzhi Fang^4^, Annik K-Laflamme^2^, Shaloo Gupta^5^

##### ^1^Kantar Health, Horsham, Pennsylvania, USA, 19044; ^2^Novartis Pharma AG, Kohlenstrasse, Basel, Switzerland, 4056; ^3^Novartis Global Services Centre, Dublin, Ireland, D04A9N6; ^4^Novartis Pharmaceuticals Corporation, East Hanover, New Jersey, USA, 07936; ^5^Kantar Health, Madison Ave, New York, USA, 10010

###### **Correspondence:** Pamela Vo (pamela.vo@novartis.com)

**Introduction**

Migraine is a distinct neurological disease ranking among the top causes of disability globally and affecting multiple domains of life for individuals [1].

**Objectives**

The purpose of this study was to describe the incremental burden of migraine on health- related quality of life (HRQoL) in those suffering from migraine of ≥4 monthly headache days (HDs) compared with matched controls in Europe (EU5; France, Germany, Italy, Spain, and UK).

**Methods**

A retrospective, cross-sectional analysis was conducted using patient-reported data from the 2017 EU5 National Health and Wellness Survey (NHWS). Outcomes from 1569 adult respondents, who self-reported a doctor diagnosis of migraine, experiencing at least one migraine in the prior month, and overall ≥4 HDs during the prior month, were stratified by HD frequency (i.e., 4-7, 8-14 and ≥15 HDs) and matched by propensity scores to 1569 respondents without migraine (controls) within each HD subgroup and country using sociodemographic characteristics. HRQoL was assessed via SF-36v2 and EQ-5D. Independent samples t-tests were used for the pairwise comparison of the outcomes across subgroups.

**Results**

All HRQoL outcomes assessed after propensity score matching in 783 respondents with 4-7 HDs, 429 respondents with 8-14 HDs and 357 respondents with ≥15 HDs were significantly lower compared with outcomes of 1569 control respondents without migraine (Table 1).

**Conclusions**

Individuals with migraine across migraine frequency subgroups report significantly worse HRQoL compared with those without migraine. Results highlight the burden that exists across the spectrum of migraine patients who may be eligible for preventive treatment.

**Ethics Approval**

The NHWS received approval from the Pearl Institutional Review Board. All NHWS respondents provided informed consent prior to participating.

**Table 1 (abstract P40).** HRQoL outcomes in NHWS respondents with migraine versus matched controls*

	Non-migraine controls (N=1,569)	4-7 HDs (N=783)	8-14 HDs (N=429)	≥15 HDs (N=357)
	Mean (SD)	Mean (SD)	Mean (SD)	Mean (SD)
Mental Component Summary Score	45.17^a^	41.02^b^	40.03^b^	36.52^c^
Physical Component Summary Score	50.87^a^	48.11^b^	47.15^b^	42.69^c^
SF-6D Utility Score	0.70	0.64	0.63	0.58
EQ-5D Index	0.83	0.75	0.73	0.58^c^
Health Status, EQ VAS	74.22^a^	67.13^b^	64.24^c^	52.26^d^

*Propensity matching was conducted using patient demographic variables identified in pre-matched bivariate results (i.e., age, gender, employment status, marital status, income, education, smoking, alcohol use, body mass index, exercise and the Charlson comorbidity index). Subscripts refer to pairwise comparisons using independent samples t-tests between subgroups. Values in the same row that do not share the same subscript are significantly different at p<0 .05

**References**

1. Steiner TJ, Stovner LJ, Vos T, Jensen R, Katsarava Z. Migraine is *first* cause of disability in under 50s: will health politicians now take notice? *The Journal of Headache and Pain*. 2018;19(1):17.

### P41 Phase 3 studies (SAMURAI, SPARTAN) of lasmiditan compared to placebo for acute treatment of migraine

#### Linda A. Wietecha^1^, Bernice Kuca^2^, Josephine Asafu-Adjei^1^, Sheena K. Aurora^1^

##### ^1^Eli Lilly and Company, Indianapolis, IN; ^2^CoLucid Pharmaceuticals, Inc., a wholly owned subsidiary of Eli Lilly and Company

###### **Correspondence:** Linda A. Wietecha (wietecha_linda_a@lilly.com)

**Background:** Lasmiditan is a novel centrally acting serotonin (5-HT_1F_) agonist that lacks vasoconstrictive activity.

**Objective:** Efficacy and safety findings from two pivotal Phase 3 studies of lasmiditan for acute treatment of migraine are reported here.

**Methods:** SAMURAI (NCT02439320) and SPARTAN (NCT02605174) were Phase 3, randomized, double-blind, placebo-controlled studies. Inclusion criteria included Migraine Disability Assessment Score ≥11 (moderate disability) and 3–8 migraine attacks per month. Patients were randomized to a first dose of treatment (SAMURAI, 1:1:1 ratio of lasmiditan 200/100 mg or placebo, SPARTAN, 1:1:1:1 ratio of lasmiditan 200/100/50 mg or placebo) which was taken within 4 hours of migraine onset (moderate severity or worse and not improving). For rescue or recurrence, patients took a randomly assigned second dose of the previously assigned lasmiditan dose or placebo. The primary and key secondary analyses compared the proportions of patients in the lasmiditan 200-mg group with the placebo group who were headache pain-free and who were most bothersome symptom (MBS)-free at 2 hours post-first dose, respectively. Treatment-emergent adverse events (TEAEs) were used to assess safety. Logistic regression was used for comparisons. The studies were approved by the appropriate Institutional Review Board for each study site.

**Results:** At 2 hours post-first dose, significantly greater proportions of patients (p<0.001) were headache pain-free (lasmiditan 200 mg: SAMURAI 32.2%, SPARTAN 38.8%; placebo: SAMURAI 15.3%, SPARTAN 21.3%) and MBS-free (lasmiditan 200 mg: SAMURAI 40.7%, SPARTAN 48.7%; placebo: SAMURAI 29.5%, SPARTAN 33.5%) with lasmiditan 200 mg compared with placebo. For both endpoints, significance was also noted for other lasmiditan dose groups (100 mg, 50 mg) compared to placebo. The most frequently reported TEAEs with lasmiditan (≥2% and greater than placebo) after the first dose were dizziness, paresthesia, somnolence, fatigue, nausea, and lethargy, and most events were mild-to-moderate in severity.

**Conclusion:** The primary and key secondary endpoints were met and safety outcomes were consistent across the two Phase 3 studies.

### P42 Conversion from chronic to episodic migraine with erenumab, a specific inhibitor of the calcitonin gene-related peptide receptor

#### Richard B. Lipton^1^, Stewart J. Tepper^2^, Stephen Silberstein^3^, David Kudrow^4^, Messoud Ashina^5^, Uwe Reuter^6^, David Dodick^7^, Feng Zhang^8^, Gregory A. Rippon^8^, Daniel D. Mikol^8^

##### ^1^Department of Neurology, Albert Einstein College of Medicine and Montefiore Medical Center, Bronx, NY, USA; ^2^Geisel School of Medicine at Dartmouth, Hanover, NH, USA; ^3^Thomas Jefferson University, Philadelphia, PA, USA; ^4^California Medical Clinic for Headache, Santa Monica, CA, USA; ^5^Department of Neurology, Danish Headache Center, Rigshospitalet Glostrup, Faculty of Health and Medical Sciences, University of Copenhagen, Copenhagen, Denmark; ^6^Department of Neurology, Charité Universitätsmedizin Berlin, Berlin, Germany; ^7^Mayo Clinic, Phoenix, AZ, USA; ^8^Amgen Inc., Thousand Oaks, CA, USA

###### **Correspondence:** Richard B. Lipton (richard.lipton@einstein.yu.edu)

**Background**

Patients with migraine are classified into episodic migraine (EM: <15 headache days/month) and chronic migraine (CM: ≥15 headache days/month). Over time, migraine patients may move from EM to CM and from CM to EM. Erenumab is a fully human monoclonal antibody that specifically inhibits the canonical calcitonin gene-related peptide receptor and was developed as a preventive migraine therapy. Erenumab has been shown to significantly reduce the number of monthly migraine days versus placebo in patients with CM and EM. As conversion to EM is a treatment goal for patients with CM, this analysis of a pivotal CM study assessed the rate of conversion to EM during short-term erenumab treatment.

**Methods**

This is a post hoc analysis of a pivotal, randomised, double-blind, placebo-controlled trial of erenumab in CM. Patients aged 18–65 years with a history of CM were randomised 2:1:1 to receive placebo, erenumab 70 or 140 mg once every 4 weeks for 12 weeks. Migraine headache information was captured daily via an electronic diary throughout the double-blind phase. Numbers and percentages of erenumab-treated patients who converted to EM were calculated and compared with placebo within each 4-week period of the 12-week double-blind phase, as well as over the entire 12 weeks based on average monthly headache days. Adjusted odds ratios (ORs) and p values were obtained from a Cochran-Mantel-Haenszel test after missing data were imputed as nonresponse. Nominal statistical significance was determined when p<0.05 without adjustment for multiplicity.

**Results**

Demographics and baseline clinical characteristics were well balanced among groups. Based on average monthly headache days over the 12-week double-blind phase, patients receiving erenumab were significantly more likely to convert to EM than patients receiving placebo (OR: 2.31; 95% confidence interval [CI]: 1.57, 3.38; p<0.001 for 70 mg erenumab, and OR: 2.10; 95% CI: 1.44, 3.08; p<0.001 for 140 mg erenumab). Higher rates of conversion to EM were also observed at 4, 8, and 12 weeks (Fig. 1).

**Conclusions**

Conversion to EM is an important treatment goal for patients with CM. Over 12 weeks, erenumab significantly increased the odds of converting from CM to EM, with conversion to EM occurring early during treatment. Future studies should assess the rate and persistence of conversion of CM to EM over the long term.

**Fig. 1 (abstract P42).**
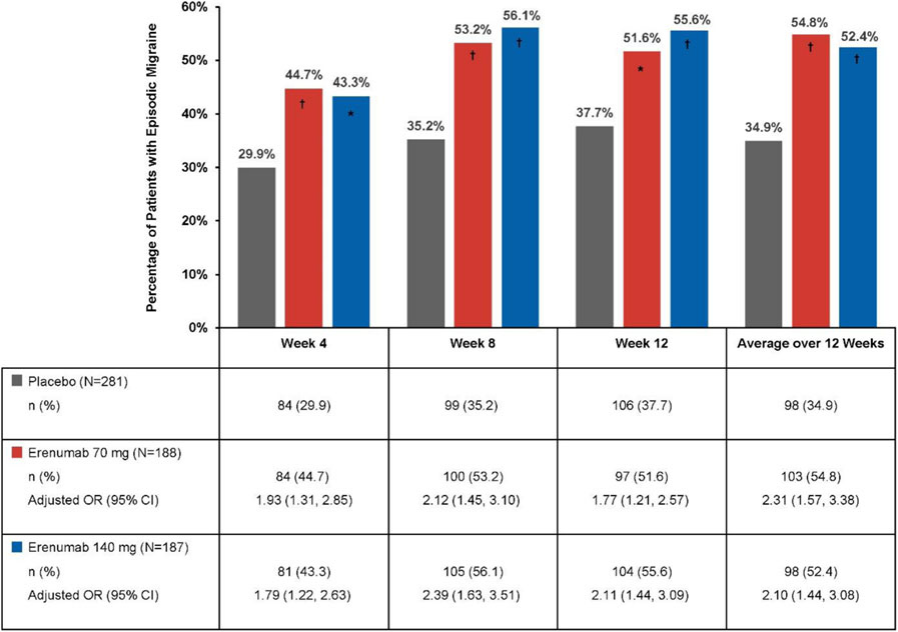
Conversion to episodic migraine during the 12-week double-blind phase**.** Odds ratio (OR) was obtained from a Cochran-Mantel-Haenszel test and stratified by region and medication overuse. Each erenumab dose group was compared with placebo. ^†^p<0.001, *p=0.003. CI, confidence interval.

### P43 Erenumab impact on patient-reported outcomes in chronic migraine in the presence of acute medication overuse

#### Stewart J Tepper^1^, Messoud Ashina^2^, Jan Klatt^3^, Pooja Desai^4^, Feng Zhang^4^, Sunfa Cheng^4^, Daniel D Mikol^4^

##### ^1^Geisel School of Medicine at Dartmouth, Hanover, NH, USA; ^2^Department of Neurology, Danish Headache Center, Rigshospitalet Glostrup, Faculty of Health and Medical Sciences, University of Copenhagen, Copenhagen, Denmark; ^3^Novartis Pharma AG, Basel, Switzerland; ^4^Amgen Inc., Thousand Oaks, CA, USA

###### **Correspondence:** Messoud Ashina (ashina@dadlnet.dk)

**Background**

Erenumab is a fully human anti-CGRP receptor monoclonal antibody approved as a preventive treatment for migraine by the US Food and Drug Administration. Erenumab has demonstrated clinically relevant efficacy in chronic migraine (CM), including in the presence of acute medication overuse (MO) at baseline (≥15 days/month on simple analgesics or ≥10 days/month on triptans, combination of therapies, or ergotamine derivatives). The objective of this study was to evaluate the efficacy of erenumab on patient-reported outcomes (PROs) in patients with CM in the presence of MO.

**Methods**

This was a subgroup analysis of data from a pivotal study of erenumab in patients with CM (≥15 headache days/month over 3 months with ≥8 migraine days/month). Patients were randomised to receive erenumab (70 or 140 mg once monthly) or placebo for 3 months, stratified by region and presence/absence of MO during the 4-week baseline period. Subgroups with MO were assessed for change from baseline at Month 3 on the Headache Impact Test (HIT-6), Migraine Disability Assessment (MIDAS), and Migraine-Specific Quality-of-Life Questionnaire (MSQ). No formal hypothesis was tested.

**Results**

Of 667 patients randomised, 41% (n=274) met MO criteria. Baseline PRO scores were similar among treatment arms. Erenumab 70 and 140 mg treatment showed greater reductions from baseline than placebo in HIT-6 total score at Month 3 in both MO and non-MO subgroups. The treatment differences (TD) exceeded the established group-level minimally important difference (MID) for HIT-6 total score (≥2.3 point reduction). Reductions from baseline to Month 3 in MIDAS total scores were greater with erenumab compared with placebo. The TD in MIDAS total score were >5 days of improvement over the last 3 months, a clinically meaningful change. At Month 3, changes from baseline were greater with erenumab treatment compared with placebo in each MSQ domain score in both subgroups. The observed differences between treatment groups exceeded the respective MIDs for the MSQ-RFR (≥3.2) and MSQ-EF (≥7.5) domain scores (Table 1).

**Conclusions**

Erenumab provided a consistent benefit across multiple measures of social and psychological impact, disability and health-related quality of life in the presence of MO in patients with CM. Change from baseline in all of the PROs studied showed clinically meaningful improvements. The observed improvement in quality of life is likely due to reduction of monthly migraine days and reduction of acute medication overuse. These data further support the use of erenumab in patients with CM, including those with MO.

**Table 1 (abstract P43).** Patient-reported outcomes at Month 3 in patients with chronic migraine by baseline medication overuse status

	Medication overuse			Without medication overuse		
	Placebo (N=113)	Erenumab 70 mg (N=77)	Erenumab 140 mg (N=78)	Placebo (N=168)	Erenumab 70 mg (N=111)	Erenumab 140 mg (N=109)
HIT-6 total score (range: 36–78)						
Baseline score, mean (SD)	63.8 (5.0)	63.9 (4.4)	63.3 (4.8)	63.0 (5.3)	63.0 (5.4)	62.2 (6.2)
Change from baseline	−2.9 (−4.1, −1.7)	−5.2 (−6.7, −3.7)	−5.4 (−6.9, −3.9)	−3.4 (−4.4, −2.3)	−6.0 (−7.2, −4.6)	−5.8 (−7.1, −4.6)
Difference from placebo		−2.3 (−4.2, −0.3)	−2.5 (−4.4, −0.6)		−2.6 (−4.2, −1.0)	−2.5 (−4.1, −0.8)
MIDAS total score						
Baseline score, mean (SD)	71.6 (52.7)	69.3 (43.4)	66.6 (55.7)	65.5 (58.2)	63.3 (47.5)	56.8 (49.4)
Change from baseline	−3.6 (−12.0, 4.8)	−22.0 (−32.0, −12.1)	−16.1 (−26.1, −6.1)	−11.2 (−17.0, −5.5)	−18.40 (−25.2, 11.6)	−23.5 (−30.3, −16.6)
Difference from placebo		−18.5 (−31.4, −5.5)	−12.5 (−25.4, 0.4)		−7.2 (−16.1, 1.7)	−12.2 (−21.1, −3.3)
MSQ-RFR (range: 0–100)						
Baseline score, mean (SD)	41.3 (17.4)	42.9 (16.5)	43.7 (17.3)	43.8 (17.7)	46.0 (19.4)	46.8 (20.3)
Change from baseline	11.7 (8.0, 15.4)	17.1 (12.6, 21.5)	17.4 (13.0, 21.9)	12.0 (9.0, 15.1)	18.4 (14.7, 22.1)	20.6 (16.8, 24.3)
Difference from placebo		5.4 (−0.4, 11.2)	5.7 (0.0, 11.51)		6.4 (1.6, 11.2)	8.5 (3.7, 13.3)
MSQ-RFP (range: 0–100)						
Baseline score, mean (SD)	60.8 (19.5)	63.8 (21.6)	61.1 (20.0)	59.9 (20.4)	60.6 (21.6)	64.2 (21.8)
Change from baseline	7.7 (4.4, 11.1)	11.6 (7.6, 15.6)	10.5 (6.5, 14.4)	10.0 (7.3, 12.7)	14.2 (11.0, 17.5)	16.7 (13.4, 20.0)
Difference from placebo		3.9 (−1.3, 9.0)	2.7 (−2.4, 7.9)		4.3 (0.0, 8.5)	6.8 (2.5, 11.0)
MSQ-EF (range: 0–100)						
Baseline score, mean (SD)	53.8 (24.3)	50.8 (23.9)	55.4 (24.8)	52.4 (26.7)	55.6 (26.1)	57.7 (27.9)
Change from baseline	8.2 (4.2, 12.2)	17.1 (12.3, 21.9)	15.9 (11.1, 20.7)	11.3 (7.9, 14.8)	19.4 (15.3, 23.5)	21.2 (17.1, 25.4)
Difference from placebo		8.9 (2.7, 15.1)	7.7 (1.5, 13.9)		8.1 (2.7, 13.4)	9.9 (4.5, 15.3)

Data represent LS mean (95% CI) unless otherwise indicated.

*CI* confidence interval, *HIT-6* Headache Impact Test (higher score indicates worse outcome), *LS* least squares, *MIDAS* Migraine Disability Assessment (higher score indicates worse outcome), *MSQ* Migraine-Specific Quality-of-Life Questionnaire (higher score indicates better outcome), *MSQ-EF* Migraine-Specific Quality-of-Life Questionnaire emotional functioning, *MSQ-RFP* Migraine-Specific Quality-of-Life Questionnaire role function-preventive, *MSQ-RFR* Migraine-Specific Quality-of-Life Questionnaire role function-restrictive, *N* number of patients in the analysis set, *SD* standard deviation

### P44 Long-term efficacy of erenumab in patients with chronic migraine who failed prior prophylactic treatment

#### Messoud Ashina^1^, Stewart J Tepper^2^, Jan L Brandes^3^, Uwe Reuter^4^, Guy Boudreau^5^, David Doležil^6^, Jan Klatt^7^, Feng Zhang^8^, Sunfa Cheng^8^, Daniel D Mikol^8^

##### ^1^Department of Neurology, Danish Headache Center, Rigshospitalet Glostrup, Faculty of Health and Medical Sciences, University of Copenhagen, Copenhagen, Denmark; ^2^Geisel School of Medicine at Dartmouth, Hanover, NH, USA; ^3^Nashville Neuroscience Group, Nashville, TN, USA; ^4^Department of Neurology, Charité Universitätsmedizin Berlin, Berlin, Germany; ^5^Centre de Traitement Neurologique, Montreal, QC, Canada; ^6^Prague Headache Center, DADO MEDICAL s.r.o., Prague, Czech Republic; ^7^Novartis Pharma AG, Basel, Switzerland; ^8^Amgen Inc., Thousand Oaks, CA, USA

###### **Correspondence:** Messoud Ashina (ashina@dadlnet.dk)

**Background**

Erenumab is a fully human anti-CGRP receptor monoclonal antibody approved as a preventive treatment for migraine by the US Food and Drug Administration. In a pivotal placebo-controlled study of adults with chronic migraine (CM; NCT02066415), erenumab reduced the number of monthly migraine days (MMD) with a safety profile similar to placebo. A subsequent open-label extension (OLE) study (NCT02174861) characterised the long-term efficacy and safety of erenumab. We performed a subgroup analysis of patients in the OLE who had failed ≥1 prophylactic treatment due to lack of efficacy and/or poor tolerability.

**Methods**

The parent study enrolled 667 adult patients with CM. Efficacy endpoints were change from parent study baseline (BL) in MMD, monthly acute migraine-specific medication treatment days (MSMD), and proportion of patients with ≥50% response (≥50% reduction from parent study BL in MMD). Data were summarised by visit for the treatment failure (TF) subgroup and at Weeks 40 and 52 for patients who completed 52 weeks of erenumab treatment. Safety was summarised for the TF subgroup based on the dose received when adverse events (AEs) occurred.

**Results**

Of 416 TF patients who enrolled in the OLE with ≥1 measurement of MMD post-BL, 326 completed 52 weeks of the OLE. For the TF subgroup, the mean (standard deviation) BL MMD and MSMD were 18.4 (4.5) and 10.8 (7.1) days, respectively. Erenumab treatment in the OLE resulted in a sustained reduction in MMD and MSMD, and achievement of ≥50% response in most patients at Week 52 (Table 1). For patients who completed 52 weeks of erenumab, the BL MMD was 18.1 (4.3) and 18.2 (4.6), and the BL MSMD was 11.5 (6.5) and 9.6 (7.6) for the 70 and 140 mg dose groups, respectively. Reductions in MMD and MSMD and the percentage of patients who achieved a ≥50% response were greater with 140 versus 70 mg at Weeks 40 and 52 (Table 1). In the TF subgroup, the subject incidence of AEs was 60.3% (70 mg) and 66.7% (140 mg), and for serious AEs was 3.1% (70 mg) and 5.2% (140 mg), consistent with the overall study population.

**Conclusions**

In CM patients who had previously failed prophylactic treatment(s), erenumab 70 and 140 mg monthly led to a sustained reduction in MMD and MSMD, and more than half of patients achieved a ≥50% response. The therapeutic effect of erenumab was greater in the 140 mg dose group and after 52 weeks of treatment.

**Table 1 (abstract P44).** Long-term efficacy with 52-week open-label erenumab treatment after 12-week double-blind treatment in patients with chronic migraine

	Treatment failure subgroup			52-week OLE completers by last dose received			
	Erenumab 70 mg/140 mgN=416			Erenumab 70 mgN=189^a^		Erenumab 140 mgN=137^a^	
OLE visit weeks	Week 12n=395	Week 40n=300	Week 52n=261	Week 40n=166	Week 52n=150	Week 40n=124	Week 52n=109
Change from BL^b^ in MMD, mean (SE)	−6.84 (0.32)	−7.75 (0.38)	−8.58 (0.41)	−7.16 (0.48)	−7.86 (0.52)	−8.58 (0.62)	−9.66 (0.65)
Change from BL^b^ in MSMD, mean (SE)	−4.17 (0.26)	−4.78 (0.29)	−5.56 (0.34)	−4.75 (0.38)	−5.47 (0.43)	−4.93 (0.47)	−5.75 (0.54)
Percentage of patients with ≥50% response^c^(95% CI)	40 (35, 44)	50 (44, 56)	55 (49, 61)	43 (36, 51)	49 (41, 57)	60 (51, 68)	63 (54, 72)

^a^Based on the final dose of erenumab received. Patients in the erenumab 140 mg group received ≥3 months of erenumab 140 mg at Week 40 and ≥6 months of erenumab 140 mg at Week 52. ^b^Parent study baseline. ^c^≥50% reduction in MMD from parent study baseline

BL, baseline; CI, confidence interval; MMD, monthly migraine days; MSMD, monthly acute migraine-specific medication treatment days; N, number of patients enrolled in the OLE and who failed ≥1 prophylactic treatment; n, number of patients with observed data at each time point; OLE, open-label extension; SE, standard error

### P45 Changes in patient functioning and disability: results from two phase 3 double-blind placebo-controlled clinical trials evaluating galcanezumab for episodic migraine prevention (EVOLVE-1 and EVOLVE-2)

#### Janet H. Ford; David W. Ayer; Qi Zhang; Jeffrey N. Carter; Vladimir Skljarevski; Sheena K. Aurora

##### Eli Lilly and Company, Indianapolis, IN, USA

###### **Correspondence:** Jeffrey N. Carter (carter_jeffrey_n@lilly.com)

**OBJECTIVE:** To evaluate changes from baseline in migraine-specific patient-reported outcomes for measures of daily functioning and disability among patients treated with galcanezumab, a humanized monoclonal antibody that binds to CGRP, or placebo in two clinical studies (ClinicalTrials.gov NCT02614183 and NCT02614196).

**METHODS:** Patients with episodic migraine (4-14 monthly migraine headache days) were treated (monthly subcutaneous injections) with galcanezumab (EVOLVE-1: 120-mg N=210, 240-mg N=208; EVOLVE-2: 120-mg N=226, 240-mg N=220), or placebo (EVOLVE-1 N=425; EVOLVE-2 N=450) during six months of treatment. The Migraine-Specific Quality of Life Questionnaire v2.1 (MSQ; higher scores indicate better functioning) was used to measure the impact of migraine on daily functioning in three domains (Role Function-Restrictive [RFR], Role Function-Preventive [RFP], Emotional Function [EF]), and the Migraine Disability Assessment (MIDAS; lower scores indicate less disability) was used to quantify headache-related disability. Both were collected at baseline and during treatment (MSQ=monthly; MIDAS=Month 3 and 6). Changes from baseline in MSQ (Month 4-6 average) and MIDAS (Month 6) scores were analyzed with mixed models repeated measures. Both studies were approved by the appropriate Institutional Review Board for each study site.

**RESULTS:** No statistically significant differences between treatment groups were observed for most baseline scores, specifically MSQ Total score (EVOLVE-2: 120-mg=59.6; 240-mg=58.0; placebo=58.0), and MIDAS Total score (EVOLVE-1: 120-mg=32.9; 240-mg=36.1; placebo=31.8; EVOLVE-2: 120-mg=30.9; 240-mg=32.8; placebo=34.3). In EVOLVE-1, the mean baseline MSQ Total scores were statistically different among galcanezumab versus placebo (120-mg=56.7; 240-mg=54.6; placebo= 59.6 both *P*<.001); similar statistical differences were observed across the individual MSQ domains. Differences of least squares (LS) mean change from baseline for galcanezumab (120-mg, and 240-mg, respectively) compared with placebo in MSQ Total score were: EVOLVE-1=7.3 and 6.7 (both *P*<.001); EVOLVE-2=8.5 and 7.3 (both *P*<.001). Significant differences (*P<*.001 for both galcanezumab dose groups compared with placebo) were also observed at Month 1 in both studies, and across the three MSQ domains (RFR, RFP, EF). Differences of LS mean change from baseline for galcanezumab (120-mg and 240-mg, respectively) compared with placebo in MIDAS Total score were: EVOLVE-1=-6.3 (*P*<.001) and -5.2 (*P*=.002); EVOLVE-2=-9.2 and -8.2 (both *P*<.001). In EVOLVE-2, each individual item score of MIDAS overall achieved statistically significant improvement (*P*<.05) for both galcanezumab doses compared with placebo; statistically significant improvements were observed for most individual items in EVOLVE-1 for both doses compared with placebo.

**CONCLUSIONS:** Patients treated with galcanezumab reported statistically significant and clinically meaningful improvements in daily functioning, and decreased disability compared with those patients who received placebo.

### P46 100% response rate to galcanezumab in patients with episodic migraine: randomized, double-blind, placebo-controlled studies

#### Abraham Jim Nagy^1^, Eric Pearlman^2^, Dustin Ruff^2^, Kathleen Day^2^, Noah Rosen^3^

##### ^1^Nevada Headache Institute, Las Vegas, NV, 89113, USA; ^2^Eli Lilly and Company, and/or one of its subsidiaries, Indianapolis, IN, 46285, USA; ^3^Hofstra Northwell School of Medicine at Hofstra University, Department of Neurology, Hempstead, NY, 11549, USA

###### **Correspondence:** Eric Pearlman (eric.pearlman@lilly.com)

**Objective:** To characterize adult patients with episodic migraine headache who achieved 100% response to galcanezumab treatment.

**Methods**: The proportions of patients with 100% response (100% reduction from baseline in monthly MHD) were calculated for each month from 2 double-blind, 6‑month galcanezumab studies in patients with episodic migraine (4 to 14 MHD and ≥2 migraine attacks per month at baseline). Patients were randomized (1:1:2) to monthly subcutaneous galcanezumab 120 mg (after 240 mg initial loading dose) or 240 mg or placebo. A generalized linear mixed model with effects for baseline MHD, treatment, month, and treatment-by-month interaction was used to estimate the mean monthly response rate. The studies were approved by a central Ethics Review Board and registered with ClinicalTrials.gov (NCT02614183 and NCT02614196).

**Results**: The analysis included 1739 patients treated with galcanezumab 120 mg (n=436) or 240 mg (n=428) or placebo (n=875). The mean monthly 100% response rate on an average month in the 6-month double-blind phase was greater for galcanezumab 120 mg (13.5%) and 240 mg (14.3%) groups versus placebo (5.9%) with odds ratios of 2.5 (95% confidence interval [CI] 1.9, 3.2) and 2.6 (95% CI 2.0, 3.4), respectively (p<0.001). The rate of 100% monthly response increased at each month over the 6-month double-blind phase with higher rates for galcanezumab dose groups (9% to 21%) than placebo (2% to 10%) (p<0.001). Evaluation of 100% response by the number of months showed greater proportions of galcanezumab-treated patients in either dose group, compared to placebo, were able to achieve a 100% response (p<0.05) though few patients had ≥4 months of 100% response. The proportions of patients with 100% response were greater in the last 3 months of treatment. Considering the average number days between migraine attacks across the 6-month period (not just during the times of 100% response), the duration of migraine headache free periods in the galcanezumab groups was 29 days for those with at least 1 month of 100% response and 55 days for those with at least 3 months of 100% response– around 6 to 11 times the mean gap of 5 days observed at baseline.

**Conclusion**: More than a third of patients with episodic migraine headache treated with galcanezumab 120 mg or 240 mg achieved 100% response for at least 1 month. More patients had 100% monthly response in the last 3 months of the 6-month double-blind period. For those with 100% response of at least 1 month, the average time between attacks for the entire treatment period was nearly 1 month and approached 2 months for patients with 3 or more months of 100% response.

**Trial Registration:** ClinicalTrials.gov NCT02614183 and NCT02614196

### P47 Diagnostic screeners for migraine: a methodological caveat

#### C. Ertsey^1^, É. Csépány^2^, M. Magyar^1,2^, T. Gyüre^2^, Bozsik Gy^2^, M. Tóth^3^

##### ^1^Department of Neurology, Semmelweis University, Budapest, Hungary; ^2^János Szentágothai Doctoral School of Neurosciences, Semmelweis University, Budapest, Hungary; ^3^Department of Neurology, Vaszary Kolos Hospital, Esztergom, Hungary

###### **Correspondence:** C. Ertsey (konfreg1@bcdtravel.hu)

**Introduction**: Validated migraine screening tools are widely used for both research and clinical purposes. While evaluating the performance of two such instruments, the ID-Migraine (ID-M) and the Migraine Diagnostic (MDX) questionnaires in tertiary headache centres, we found a source of false positive cases.

**Methods**: Consecutive patients presenting at two Hungarian headache centres completed the questionnaires during their outpatient visits. The patients' results were confronted with the clinical diagnosis according to the ICHD-3beta diagnostic criteria. The questionnaires' sensitivity, specificity, positve and negative predictive value as well as their misclassification error were calculated.

**Results**: A total of 405 patients completed both questionnaires. 247 patients had only one type of headache, whereas 158 patients had at least 2 different headache diagnoses. The clinical diagnosis of migraine was made in 324 patients (200 had only migraine, and 124 had at least another type of headache beside migraine). Tension type headache (TTH) alone was diagnosed in 50 patients, cluster headache (CH) alone in 23, while 8 patients had other headaches (ICHD groups 4 and 11). Both questionnaires' performance was adequate: the sensitivity and specificity of the ID-M were 0.95 and 0.44, and those of the MDX were 0.95 and 0.57, respectively. Among the 23 CH patients 21 (91%) were false positive for migraine according to their ID-M scores and 20 (87%) according to their MDX scores. This corresponds to 5.9% and 5.8% of all positive ID-M and MDX cases, respectively.

**Conclusion**: In this study, the overwhelming majority of CH patients had positive ID-M and MDX scores. In tertiary centres, and also in special populations (such as headahce self-help groups) CH patients may be overrepresented and might be overlooked. In the case of non-representative sampling studies using migraine diagnostic screeners as the sole source of diagnosis may have a significant number of false positive patients.

### P48 Comparison of personality traits and dependence behavior between MOH patients and illicit drug users

#### Baraldi Carlo, Pellesi Lanfranco, Guerzoni Simona, Cainazzo Maria Michela, Lo Castro Flavia, Pini Luigi Alberto

##### Medical Toxicology-Headache and Drug Abuse Centre, Department of Diagnostic Medicine, Clinical and Public Health, University of Modena and Reggio Emilia, Via del Pozzo 71, 41124, Modena, Italy.

###### **Correspondence:** Baraldi Carlo (infocarlo.baraldi@gmail.com)

**Background**

Chronic migraine usually leads to the excessive consumption of acute medications, generating a secondary headache called medication overuse headache (MOH). MOH pathogenesis has not been clarified yet, but personality traits seems to have a role in it. Moreover, illicit drug users have been compared with MOH patients, trying to find out clinical overlaps. Despite this, is also proved that the different the overused drug is and the different is the response to detoxification treatment, as well as the eventual relapse into it. The focus of this study is to explore the difference in the Leeds Dependence Questionnaire as well as personality traits between patients suffering for MOH and patients affected by illicit drug use.

**Methods**

A questionnaire containing the Leeds dependence questionnaire, the ‘International Personality Item Pool (IPIP) and the Symptom Checklist-90-R was filled out by patients enrolled during their recovery in Modena Headache and Drug Abuse Research Center- Medical Toxicology Unit, between 2015 and 2017. Mean values of the above-mentioned questionnaires were compared using a one-way analysis of variance followed by the Tuckey-Kramer post-hoc comparison test. These comparisons were made between illicit drug users and 4 categories defined by the overuse drug by headache people (triptans, NSAIDs, opioids and associations).

**Results**

The Leeds dependence questionnaire was significantly higher in illicit drug users compared with triptan users and NSAIDs ones (17±6.08 vs. 9.39±5.5 for triptans and vs. 10.9±7.08, respectively). Moreover, IPIP analysis showed that illicit drug users have a greater somatizations than triptan and NSAIDs users (1.03±0.08 vs. 0.56±0.4 for triptans and vs. 0.66±0.52, respectively). Furthermore, a greater mean of the sub-voice hostility of the IPIP was seen in illicit drug users rather than in triptan users. No other differences between illicit drug users and MOH patients were seen.

**Conclusion**

Personality differences are present especially for triptan overusers versus illicit drug users; in particular, illicit drug users showed a higher grade of dependence and higher depressive as well as hostility traits than triptan overuses. It should be highlighted that no differences were seen between other drugs’ users and illicit drug users. These observations can suggest that overuses of headache acute medications other than triptans have a higher degree of dependence towards their overuse drug, as they are psychologically more similar to illicit drug abusers than triptan overusers.

### P49 VALIDITY AND RELIABILITY TURKISH HEADACHE IMPACT TEST (HIT-6) IN PATIENTS WITH EPISODIC AND CHRONIC MIGRAINE

#### P. Yalınay Dikmen^1^, M. Bozdag^2^, M. Gunes^3^, S. Kosak^1^, B. Tasdelen^4^, D. Uludüz^3^, A. Ozge^2^

##### ^1^Acıbadem University, School of Medicine, Department of Neurology, Istanbul, Turkey; ^2^Mersin University, Faculty of Medicine, Department of Neurology, Mersin, Turkey; ^3^Istanbul University, Cerrahpasa Faculty of Medicine, Department of Neurology, Istanbul, Turkey; ^4^Mersin University, Faculty of Medicine, Department of Biostatistics and Medical Informatics, Mersin, Turkey

###### **Correspondence:** P. Yalınay Dikmen (pinar.yalinay@acibadem.com)

**Background:** The Headache Impact Test (HIT-6) is a self-report questionnaire designed to evaluate the impact of headache on quality of life in both clinical research and practice. The aim of this study is to assess the comprehensibility, internal consistency, patient-physician reliability and validity of Turkish version of HIT-6 questionnaire in patients with episodic and chronic migraine.

**Methods:** Migraine patients applying to three Neurology Clinics in Turkey were evaluated at the baseline (visit 1) and week 4 (visit 2). Patients were randomized 2:1 at the visit 1 as group A and group B. Patients in both group were asked to complete the HIT-6 questionnaire by themselves at the baseline and follow-up visits. Patients in group A were additionally assessed by the physicians who also applied the HIT-6 questionnaire. All patients were also asked to complete Comprehensibility Assessment Form (CAF) at visits 1 and 2. The physician also completed this form for the patients of group A at all visits. The correlation between the total patient-applied HIT-6 scores and the corresponding total physician-applied HIT-6 scores of group A was analyzed to evaluate the patient-physician reliability of the questionnaire.

**Results:** A total of 114 migraine patients (60.5 % female, mean age:35,8±9.2;77 episodic, 37 chronic) were enrolled into the study. Comprehensibility of HIT-6 was evaluated as good for each item. At least 95% of the patients stated that the items were comprehensible. The significant positive correlation (r=0.876; p<0.001) was detected between patient and physician HIT-6 assessments in Group A. Structural validity of HIT was firstly examined using exploratory factor analysis (EFA) and explained variance proportion was acceptable (> 0.60). Then, Confirmatory factor analysis (CFA) was employed in order to ensure consistency of HIT scores and model fit statistics were obtained as good (≥0.90). Internal consistency of HIT-6 was assessed using Cronbach’s alpha and was found at acceptable (>0.75) or excellent (>0.87) levels in both patients and physician applied HIT-6 scores at visits 1 and 2, respectively. Total HIT-6 score showed good test-retest reliability (r=0.68).

**Conclusions:** These results demonstrated that the Turkish translation is equivalent to English version of HIT-6 in terms of internal consistency, test-retest reliability and validity. The Turkish translation of HIT-6 could be reliably used by physicians in assessing the impact of headache in both episodic and chronic migraine patients.

**Ethics approval**

The study received approval by the Ethics Committee of the Acıbadem University, School of Medicine, approval number 2017-16/15.

### P50 Retrospective analysis of peripheral nerve block for the treatment of headaches and cranial neuralgias in the neurology section of a general hospital

#### Vanesa Adell^1^, Jessica García-Alhama^2^, Sonia Jaraba^2^, Joan Prat^3^, Mariano Huerta^2^

##### ^1^Neurology department, IDIBELL, Hospital de Viladecans, Barcelona, Spain; ^2^Neurology department, Hospital de Viladecans, Barcelona, Spain; ^3^Neurology department, Hospital Universitari de Bellvitge, Barcelona, Spain.

###### **Correspondence:** Vanesa Adell (vanesa.adell@bellvitgehospital.cat)

**Purpose:** Anaesthetic block (AB) is a diagnostic and mainly therapeutic technique increasingly used in the treatment of headaches.

**Method**: We conducted a retrospective observational study of activity registered and coded as "peripheral nerve or trigeminal branches block" in the neurology service of our hospital between May 2009 and March 2018. Anaesthetic blocks of cranial and / or facial nerves for the diagnosis or treatment of headache or neuralgia were included. Therapeutic response (TR) was defined as the improvement of pain frequency and / or intensity ≥ 7 days for preventive treatment and ≥ 24 hours for acute treatment.

**Results**: The total number of blocks was 224 carried out in 100 patients (73% women, with an average age of 57.3 ± 15. 2 years old) and 42% patients received > 1 block. The most commonly treated pathologies were: migraine with 75/224 blocks (33.5%, 51/224 episodic and 24/224 chronic migraine), cervicogenic headache 41/224 blocks (18%) and cluster headache 28/224 blocks (12.5%, 20/224 episodic and 8/224 chronic). A total of 168/224 (75%) blocks were performed on the great occipital nerve (GON) on one (30%) or both sides (45%), 34/224 (15%) were performed on GON in combination with other points and 24/224 (10.7%) on the supraorbital nerve (SON). Nerve block with anaesthesia alone was the most used treatment (114/224, 51%) followed by combination of anaesthesia with corticoids (96/224, 43%). Therapeutic response was obtained in 141/224 (62%) of the blocks performed: 29/41 (70%) in cervicogenic headache, 44/75 (58%) in migraine and 16/ 28 (57%) in cluster headache.

**Conclusion**: AB is a therapeutic support technique that can be performed safely in a neurology section of a general hospital as an adjuvant treatment with a high percentage of symptomatic improvement.

### P51 RELATIONSHIP BETWEEN SEVERITY OF MIGRAINE AND VITAMIN D DEFICIENCY: A CASE CONTROL STUDY

#### L. Rapisarda^1^, A.D. Montisano^1^, A. Sarica^2^, G. Demonte^1^, F. Tosto^1^, A. Gambardella^1,2^, F. Bono^1,2^

##### ^1^Headache Center, Institute of Neurology, Department of Medical and Surgical Sciences, Magna Graecia University of Catanzaro, Italy; ^*2*^Neuroscience Research Center, Department of Medical and Surgical Sciences, Magna Graecia University of Catanzaro, Italy

###### **Correspondence:** L. Rapisarda (laura_rapisarda90@yahoo.it)

**Background**

It is well recognized that vitamin D deficiency is involved in a number of neurological disorders. However, if the serum vitamin D levels correlate with severity of migraine remains uncertain [1].

**Materials and methods**

In this prospective study we enrolled 157 patients with primary headache and 62 healhty controls. Each patient underwent a careful neurological evaluation, recording the frequency of headache through a monthly headache diary; we also evaluated pain (VAS), disability (MIDAS, allodynia (ASC-12); depression and anxiety (BDI-II and HARS), and medical overuse. All participants underwent a venous blood sampling for 25-hydroxyvitamin D. It has been considered sufficient 25-hydroxyvitamin D ≥30 ng/ml; insufficient for values 20-30 ng/ml; deficient ˂20 ng/ml.

**Results**

Patients were grouped into 3 groups: Group 1 included 113 patients with chronic migraine (17 M, 96 F; age 41,5±13,4; BMI 25,5±4,2); Group 2 included 44 patients with episodic migraine (8 M, 36 F; age 39,9±12,8; BMI 22,7±2,9); control group included 62 patients (24 M, 38 F; age 40,8±14,6; BMI 24,1±3,7). Serum 25-hydroxyvitamin D deficiency has been reported in headache sufferers and less severely in control group (12,8±5,4; 18,6±6; 22±6; p<0,001). Chronic migraneurs had a higher percentage of medication overuse (68% and 27% respectively, p <0,001), higher disability (MIDAS: 36,5±17,2 vs 9,6±7 p<0,001) and greater psychiatric comorbility (BDI-II: 18,9±8,6 vs 16±7,4, p<0,001; HARS: 19±9 vs 16,2±9,3, p<0,001). No statistically relevant differences were reported among the other clinical parameters. Statistical analysis showed that serum 25-hydroxyvitamin D levels were negative related to headache frequency (Pearson correlation coefficient -0,636; p<0,001). Moreover there was a negative relation even between vitamin D levels and BMI, HARS, BDI-II, MIDAS, VAS (-0,315; -0,406; -0,471; -0,525, -0,4; p<0,001).

**Discussion**

As 25-hydroxyvitamin D receptors (VDR) and 1a-hydroxylase (1a-OHase), are expressed in neurons of dorsal ganglia[2], limbic system, basal ganglia, cerebellum, and cerebral cortex, particularly in prefrontal cortex, cingulate gyrus, hyppocampus, dentate gyrus, substantia nigra, lateral geniculate nuclei, supraoptic and paraventricular nuclei in hypotalamus [3]; recent studies showed that vitamin D is implicated in descending modulation of endogenous pain control. Indeed we speculated that vitamin D deficiency may facilitate headache attacks; moreover it has a role in peripheral and central sensitization which lead to migraine chronification and are responsible for other migraine related phenomena such as allodynia.

**Conclusion**

Our data indicate that severe vitamin D deficiency is associated with higher frequency of headache in migraine patients, suggesting that serum vitamin D levels correlate with severity of migraine.

**REFERENCES:**

1. Prakash et al. Vitamin D in chronic tension-type headache: a case control study. Headache. 2017 Jul;57(7):1096-1108.

2. Tague et al. Vitamin D receptor and enzyme expression in dorsal root ganglia of adult female rats: Modula/on by ovarian hormones. Journal of Chemical Neuroanatomy 41 (2011) 3. Eyles et al. Distribu/on of the Vitamin D receptor and 1a-hydroxylase in human brain. Journal of Chemical Neuroanatomy 29 (2005) 21–30.

### P52 A case-control study of visual-vestibular mismatch in childhood with primary headaches

#### Daniele Monzani^1^, Pellesi Lanfranco^2^, Guerzoni Simona^2^, Cainazzo Maria Michela^2^, Lo Castro Flavia^2^, Baraldi Carlo^2^, Pini L Alberto^2^

##### ^1^Otolaryngology Unit, Department of Diagnostic Medicine, Clinical and Public Health, University of Modena and Reggio Emilia, Via del Pozzo 71, 41124, Modena, Italy; ^2^ Medical Toxicology-Headache and Drug Abuse Centre, Department of Diagnostic Medicine, Clinical and Public Health, University of Modena and Reggio Emilia, Via del Pozzo 71, 41124, Modena, Italy

###### **Correspondence:** Baraldi Carlo (Carloinfocarlo.baraldi@gmail.com)

**Background**

Disorientation, nausea, imbalance, confusion, unsteadiness and dizziness are unpleasant mates of the 40% of migraineurs children. The abovementioned symptoms may be triggered by repetitive visual moving patterns (optic flow stimulation), even in inter-ictal phases and are called “visual vestibular mismatch” (VVM). Bronsky and coworkers found that there were no underlining neurologic or otolaryngology conditions in all children from a Pediatric Vestibular Clinic complaining vestibular symptoms associated with migraine. However, M is frequently triggered by specific visual stimuli such as repetitive patterns, suggesting a central relationships between pain and vestibular pathways. In this work the effect of optokinetic stimulation (OKS) on balance function in migraineurs children suffering for VVM and without vestibular abnormalities was explored to detect a possible central vestibular impairment..

**Methods**

A static posturography examination was performed on 30 children suffering for primary headaches and 22 healthy controls. Children were texted in three different conditions subsequently: with closed eyes (EC), with open eyes (EO) and under a condition of OKS- obtained with a vertical bar in front of their eyes. Posturogram length (L), surface (S) and the mean sway velocity of the center of pressure (V) were recorded under EO, EC and OKS conditions. The EC/EO ratio (Romberg index) was calculated regarding the value of S and the destabilization index (DI) with the values obtained under OKS. One way ANOVA followed by Tukey-Kramer post-hoc comparison test was performed to compare the mean values of the abovementioned parameters during the different conditions. The study was approved by the local Ethical Committee (protocol number: 1921, study code NOC09).

**Results**

S and L were significantly lower in the control group compared with headache sufferers, whilst V showed an opposite trend, both in Eo and EC conditions (all p<0.05). Under OKS a greater variability of explored parameter was seen, but the trend was the same as before, without differerences between the left and right OKS (all p<0.05). RI was not significantly different in any group, neither was the DI.

**Conclusion**

A vestibular impairment is present in children suffering for headaches, even in inter-critic phases. The vestibular frailty of those patients can be attributed to a central impairment of vestibular ways, connecting peripheral vestibular organs with the vestibular cortex. Maybe trigeminal connections with the vestibular ganglion as well as central vestibular pathways may play a role in it.

**Table 1 (abstract P52).** See text for description

**Stabilometric findings**				
*Eyes Open*				
*Parameter*	*C*	*TTH*	*M*	p*
Ellipse Surface	20.134±13.203	339.181±223.8	339.745±195.423	<0.0001
Ellipse Lenght	22.596±10.623	530.733±195.997	548.924±164.403	<0.0001
Mean Sway Velocity	22.76±13.617	12.391±3.64	11.127±3.0396	0.0004
FFTx	24.269±11.488	0.071±0.084	0.047±0.04	<0.0001
FFTy	20.726±10.452	0.0455±0.038	0.0526±0.045	<0.0001
*Eyes Closed*				
*Parameter*	*C*	*TTH*	*M*	*P*
Ellipse Surface	22.927±10.991	407.681±211.192	410.367±208.812	<0.0001
Ellipse Lenght	36.3±78.973	793.875±600.239	728.162±257.888	<0.0001
Mean Sway Velocity	26.292±11.498	17.793±10.976	14.171±5.635	0.01
FFTx	22.132±12.584	0.045±0.039	0.045±0.046	<0.0001
FFTy	20.884±8.691	0.049±0.054	0.038±0.031	<0.0001
Romberg Index	2.157±2.137	1.478±0.634	1.292±0.489	0.155
Destabilisation Index	5.295±4.964	4.119±3.071	2.641±1.067	0.0735
**Stabilometric findings OKN Right and Left**				
*OKN Right*				
*Parameter*	*C*	*TTH*	*M*	p*
Ellipse Surface	21.986±12.159	509.318±303.792	456.057±391.45	<0.0001
Ellipse Lenght	18.531±15.928	683.604±265.269	690.163±151.569	<0.0001
Mean Sway Velocity	22.409±11.745	15.643±4.279	14.046±2.87	0.0053
FFTx	23.562±9.849	0.065±0.092	0.033±0.021	<0.0001
FFTy	19.2102 11.78949	.0681818 .0972438	.0444298 .0327543	<0.0001
*OKN Left*				
*Parameter*	*C*	*TTH*	*M*	P*
Ellipse Surface	29.778±13.215	527.543±291.058	436.288±340.867	<0.001
Ellipse Lenght	1252.205±5381.505	725.216±270.133	708.717±224.549	0.8630
Mean Sway Velocity	22.409±11.745	15.643±4.279	14.046±2.87	0.4209
FFTx	21.458±12.251	0.035±0.013	0.065±0.071	<0.0001
FFTy	24.837±13.58	0.068±0.072	0.042±0.031	<0.0001

*P refers to ANOVA followed by Tuckey-Kramer post-hoc test

### P53 Primary headaches associated with physical exertion

#### Füsun Mayda Domaç, Sergül Zengin, Mehmet Fatih Demir

##### Health Sciences University, Erenköy Mental and Neurological Diseases Training and Research Hospital, Neurology Department, Istanbul, Turkey

###### **Correspondence:** Füsun Mayda Domaç (fusundomac@yahoo.com.tr)

**OBJECTIVE**:Headaches associated with physical exertion is rare and they can be primary or secondary and their etiologies may differ depending on the headache type. Primary headaches are less often seen and pathogenesis is poorly understood. Herein, we report the cases with primary physical exertion related headaches

**MATERIAL AND METHODS**: Headache classification of 473 patients followed up at the Headache outpatient clinic of Health Sciences University, Erenköy Mental and Neurological Diseases Training and Research Hospital were determined due to ICHD-3. Patients with physical exertion headache were investigated detailed neurologic examination, by cranial computerized tomography, cranial magnetic resonance (MR) imaging, cranial MR angiography and venography if necessary. Patients with any secondary reasons for headache were excluded.

**RESULTS**: Fifteen patients (%3.17) had primary headache associated with physical activity. Among these 4 male patients had primary cough headache with sudden onset and short lasting headache in association with coughing localized at occipital region. Primary exercise headache was diagnosed in 4 male patients with bilateral pulsatile headache. Headache has occurred while weight-lifting in 2 patients and in hot weather in 2 of them. Primary headache associated with sexual activity were diagnosed in 4 male and 1 female patients. Headache was usually bilateral, starting with a dull ache and abrupt explosive intensity in association with orgasm. One of the patients had a history of migraine. Two patients were diagnosed as primary thunderclap headache with abrupt onset high intensity headache.

**CONCLUSION**: Primary headaches associated with physical exertion have similar characteristics of secondary headaches. All may occur as a manifestation of a possible underlying, symptomatic etiology, and additional diagnostics should typically be pursued to rule out serious causes.

### P54 Headache burden in the Emergency Room

#### A.Quka^1^,O. Cibuku^1,2^, I.Zekja^1^, I. Xhura^1,2^, J. Kruja^1,2^

##### ^1^Neurology Department, UHC MotherTeresa, Tirana, 1000, Albania; ^2^Neurology, Faculty of Medicine, University of Medicine, Tirana, 1000, Albania

###### **Correspondence:** A.Quka (aidaquka@gmail.com)

**Introduction**: Headache remains an important symptom which leads the patients to the ER service. The aim of this study was to evaluate the burden of the patients presenting with this main symptom and try to evaluate their further management (diagnostic tests, hospitalization rate, causes and treatment) in the ER.

**Methods**: We prospectively collected the data of patients complaining of headache as the main symptom, admitted in the neurological ER unit, UHC Mother Teresa for three consecutive months,January,2018 –March 2018 and evaluated their further management in the ER.

**Results**: 2695 patients were admitted in the neurological ER service, and 22.15% (597) of them complained of headache as the main symptom, 374(62.6%)women and 223 (37.4%) males. Mean age was 49.1±13.4 years. Primary headache was observed in 285 (47.7%)pts: Migraine 151(25.3%)pts, Tension type headache 117 (19.5%)pts, Trigeminal neuralgia in 11(1.8%)pts with and 7(1.1%)other primary headaches . Secondary headaches were observed in 312 (52.3%)pts: Subarachnoidal hemorrhage 35 (5.9%)pts, Other types of stroke 78pts (13.1%), Infections 65(3.8%)pts, Primary brain tumors 15(2.5%)pts, Secondary brain tumors 19 (3.2%)pts, uncontrolled arterial hypertension 51 (8.5%)pts, Trauma and other types of secondary headache 49(8.2%)pts. Brain CT scan was performed in 411(68.8%)pts, brain CTA was performed in 153 (25.6%)pts. Hospitalization rate was 59.1%(353pts). Most pts were treated in the ER with NSAID (85.59%) combined with benzodiazepines (59.5%). Opioids and steroids were used less frequently.

**Conclusions**: Pts presenting with headache as the main primary symptom occupy an important part of the neurological care in the ER service and more than half of them in our study had primary headaches or uncontrolled arterial hypertension. A better identification and follow up of pts with primary headaches, infections and uncontrolled arterial hypertension, by the primary care physician or local neurologist, may lead to reduction in the number of pts presenting in the ER and thus avoid unnecessary tests and reduce the neurologist working time in the ER.

### P55 Prevalence and risk factors of tension-type headache among adolescents in Kharkiv city (Ukraine)

#### Stepanchenko Kostiantyn (kosty0516@gmail.com)

##### Department of neurology and child neurology, Kharkiv Medical Academy of Postgraduate Education, Kharkiv, 61176, Ukraine

**Background.** The Worldwide prevalence of tension-type headache (TTH) an average of 42% in adults. In Ukraine the prevalence of TTH in adolescents is poor documented. Aim of this study – to determine the prevalence of TTH and identify the risk factors among adolescents in Kharkiv.

**Methods.** The scientific work is based on the results of the survey of 2342 adolescents aged 13-18 years in the period of preventive medical examinations in randomly selected secondary schools in Kharkiv. The analysis of the risk factors for the development of TTH was conducted by the questionnaire developed by us. Possible risk factors in the formation of TTH were divided into 4 groups: hereditary, biomedical, psychosocial and welfare.

**Results.** It was found that the prevalence of headache in adolescents in Kharkiv was 80,2%. Girls were more likely to suffer from headache (56,3%). Primary headache was the most common type of headache (72,2%). TTH has taken the main place among primary headaches (61,1%). We observed infrequent episodic TTH in 59,7% of adolescents with TTH, frequent episodic TTH – in 33,8% and chronic TTH – in 6,5% of adolescents with TTH.

The risk factors for development of TTH in adolescents were genetics factors for headache (59%), gastrointestinal (24%) and autonomic nervous system (46%) disorders; pathology of the ante-, intra- and postnatal periods: fetal pathology (51%) and newborn (55,8%); extragenital mother's pathology (27%) (especially for adolescents with chronic TTH (35,5%)), pathology of pregnancy and childbirth (50,5%); syndrome of increased neuro-reflex excitability (46%) and sleep disorders in early childhood; comorbidity (increased excitability (18%), sleep disturbance (27%), gastrointestinal tract diseases (24%)); unfavorable family factors (divorce (23%) and quarrel (27%) of parents); poor adaptation to pre-school (51%) and school education (40%); hypodynamics (prolonged work at the computer (1,4±0,2 hours), drop in visits of sports groups (27,3%), smoking (41%) and alcohol consumption (23%).

**Conclusions.** The frequency of TTH in adolescents in Kharkiv city is 61,1%. The most common form is infrequent episodic TTH. Chronic TTH is observed in 6,5%. It’s revealed that the main risk factors for the development of TTH in adolescents are genetic factors, pathology of the perinatal period, concomitant pathology, unfavorable family factors, hypodynamia, smoking and alcohol consumption. Prognostic tables have been developed for allocating risk groups for the formation and chronization of TTH for the timely administration of treatment and prophylactic measures.

**Ethics approval**

The study was approved by Kharkiv Medical Academy of Postgraduate Education`s Ethics Board, approval number 142.

### P56 Pediatric migraine: A new pediatric migraine-spesific comorbidity index on prognosis

#### Didem Derici Yıldırım^1^, Bahar Taşdelen^1^, Derya Uludüz^2^, Aynur Özge^3^, Osman Özgür Yalın^4^, Saim Yoloğlu^4^

##### ^1^Department of Biostatistics and Medical Informatics, Mersin University Faculty of Medicine, Mersin, Turkey; ^2^Istanbul University, Cerrahpasa School of Medicine, Neurology Department, İstanbul, Turkey; ^3^Mersin University, School of Medicine, Neurology Department, Mersin, Turkey; ^4^Health Sciences University, Istanbul Training and Research Hospital, İstanbul, Turkey

###### **Correspondence:** Osman Özgür Yalın (osmanozguryalin@yahoo.com)

**Objective:** We aimed to develop and validate a comorbidity index to predict prognosis of migraine, based to a longitudinally followed heterogeneous population.

**Methods:** The study has been conducted from a 15-year computer based follow up data as a part of Turkish Headache Database Study group. All patients selected from Mersin University School of Medicine and Cerrahpaşa School of Medicine Neurology department headache centers. First, the latent sub-groups were determined with Group-based trajectory modeling (GBTM) as outcome’s change in time was different for each patient. With the results of GBTM analysis we developed pediatric migraine specific comorbidity index.

**Results:** The study conducted with 481 pediatric migraine patients. Sub-group analysis performed to reveal effects of age, gender, headache attack severity, frequency and duration. Out of all weighting methods to evaluate co-morbidities, the three group model and quadratic form of all groups fitted the data best. After deciding the number of group and functional form, the information criteria and minimum group percentage of weighting methods were compared. The best method was the posterior probabilities obtained from latent class analysis (LCA) taken as weights. We found statistically significant difference in the term of age between subgroups. The third group-which involves more then three comorbidities were significantly older (p=0.004). Headache severity and attack duration did not differ between groups. Headache attack frequency differed between groups, third group headache frequency found significantly higher (p=0.018)

**Conclusion:** In this study we developed a pediatric migraine comorbidity index (p-MCI) to evaluate effect of covariates on migraine course. There are need to studies to predict course of migraine to classifify high risk patient groups.

**Keywords:** Migraine, group-based trajectory modeling, co-morbidity index, prognosis.

### P57 Characteristics of Isolated Headache patients in Cerebral Venous Sinus Thrombosis (CVST): The Results of VENOST - national survey

#### Derya Uludüz^1^, Osman Özgür Yalın^2^, Taşkın Duman^3^, Füsun Mayda Domaç^4^, Şerefnur Öztürk^5^, Vildan Yayla^6^, Ali Yavuz Karahan^7^, Nazire Afşar^8^, Mehmet Ali Sungur^9^, VENOST Study Group

##### ^1^Istanbul University Cerrahpasa School of Medicine, Professor, Neurology Department,İstanbul, Turkey; ^2^University of Health Sciences, Istanbul Training and Research Hospital, Associate Professor, Neurology Department, İstanbul, Turkey; ^3^Hatay Mustafa Kemal University School of Medicine, Professor, Neurology Department, Hatay, Turkey; ^4^University of Health Sciences, Erenköy Training and Research Hospital, Associate Professor, Neurology Department, İstanbul, Turkey; ^5^Selçuk University School of Medicine, Professor, Neurology Department, Konya, Turkey; ^6^University of Health Sciences, İstanbul Sadi Konuk Training and Research Hospital, Professor, Neurology Department, İstanbul, Turkey; ^7^Uşak University School of Medicine, Professor, Neurology Department, Uşak, Turkey; ^8^Acıbadem University School of Medicine, Professor, Neurology Department, İstanbul, Turkey; ^9^ University of Health Sciences, Bakırköy Training and Research Hospital, MD, Neurology Department, İstanbul, Turkey; ^10^Düzce University School of Medicine, Professor, Biostatistics Department, Düzce, Turkey

###### **Correspondence:** Osman Özgür Yalın (osmanozguryalin@yahoo.com)

**Introduction:** Cerebral Venous Sinus Thrombosis (CVST) is a rare cause of stroke in young age with a highly variable clinical presentation. The incidence of CVST was previously underestimated due to lack of detailed neuroimaging evaluation. With the developing radiological technology it’s estimated to account 3-5% of all strokes. Despite advances in the diagnosis of CVST in recent years, diagnosis can be challenging due to intensely variable clinical spectrum. Even worse CVST may present with solely headache. In this study we aimed to reveal characteristics of isolated headache patients diagnosed with CVST.

**Methods:** CVST study is a nationwide, multicenter study comprised 1144 patients, data obtained from the patient follow up files and 35 national stroke centers participated to this study. This study provides largest number of CVST patient data analysis until today to literature.

**Results:** Among 1144 CVST cohort isolated headache presented in 287 (25.1%) patients. Headache symptom onset was frequently acute and headache is severe in the isolated headache group (p<0.001). Transverse sinus thrombosis was more frequently involved (p<0.001) and parenchymal lesions were less common in the isolated headache group (p<0.001). MRI was normal in 79.8% of the patients. Modified Rankin Score on admission and first, second and third months of the follow up were more favorable in isolated headache patients.

**Conclusion:** CVST could present acute, subacute and chronic symptoms. Symptoms can be intracranial hypertension (headache, nausea, visual symptoms etc.), epileptic seizure or focal neurological findings. Various types of clinical presentation is the main cause of late diagnosis. We observed that one fourth of the patients were admitted with isolated headache without any neurological symptoms or findings even without any parenchymal lesions. CVST diagnosis is important in those patients. Prompt diagnosis of CVST is based to sufficient first evaluation of patients according to headache characteristics.

### P58 One-year incidence of Chronic Migraine in Tertiary Headache Outpatient Clinics: A multi-center study

#### Aynur Özge^1^, Derya Uludüz^2^, Osman Özgür Yalın^3^, Seden Demirci^4^, Macit Selekler^5^, Ali Akyol^6^, Şebnem Bıçakçı^7^, Vesile Öztürk^8^, Musa Öztürk^9^, Betül Baykan^10^, Mehmet Ali Sungur^11^, Aksel Siva^2^

##### ^1^Mersin University School of Medicine, Professor, Neurology Department, Mersin, Turkey; ^2^Istanbul University Cerrahpasa School of Medicine, Associate Professor, Neurology Department,İstanbul, Turkey; ^3^University of Health Sciences, Istanbul Training and Research Hospital, MD, Neurology Department, İstanbul, Turkey; ^4^Isparta Süleyman Demirel University School of Medicine, Assistant Professor, Neurology Department, Isparta, Turkey; ^5^Kocaeli University School of Medicine, Professor, Neurology Department, Kocaeli, Turkey; ^6^Aydın University School of Medicine, Professor, Neurology Department, Aydın, Turkey; ^7^Çukurova University School of Medicine, Professor, Neurology Department, Adana, Turkey; ^8^Dokuz Eylül University School of Medicine, Professor, Neurology Department, İzmir, Turkey; ^9^University of Health Sciences, Bakırköy Training and Research Hospital, MD, Neurology Department, İstanbul, Turkey; ^10^İstanbul University School of Medicine, Professor, Neurology Department, İstanbul, Turkey; ^11^Düzce University School of Medicine, Professor, Biostatistics Department, Düzce, Turkey

###### **Correspondence:** Osman Özgür Yalın (osmanozguryalin@yahoo.com)

**Background:** Chronic migraine is a disabling neurological disorder, affecting 0.5-2% of population. Exact understanding of pathophysiological mechanisms are lack and treatment options are limited. Chronic migraine is a disorder that affect individual quality of life, and also affect population with markedly disability at working. It’s also one of the important cause of analgesic overuse, and related with many other indirect costs.

**Aims:** This study is aimed to investigate burden of Chronic Migraine at tertiary Neurology clinics, specifically at headache outpatient clinics. Although there are studies investigating prevalence of migraine, this is the first study in our country investigating incidence of Chronic Migraine in tertiary headache centers.

**Methods:** Ten tertiary Neurology clinics included and 821 patients enrolled to study. Diagnosis based to International Classification of Headache Disorders- 3 beta (ICHD-3 beta). At each center, all consecutive patients of Headache outpatient clinics admitted to study for 1-year. To estimate Chronic Migraine incidence; only new diagnosed patients included to statistical analysis.

**Results:** Eight-hundred and twenty one-patient included to study. 733 patients (89.3%) diagnosed wtih primary headache disorder and 88 patients (10.3%) with secondary headache disorder. Totally study groups’ 66.4% have diagnosed migraine, %18 have tension type headache, and 1.7% have trigeminal autonomic cephalalgias. The Chronic Migraine incidence was estimated as 31.2% in 821 headache clinic patients. We compared clinical features of headache between primary and secondary headache groups. Primary headache disorders were more common at males (p<0.001), patients had longer headache history (years) (p<0.001), and attack duration (p=0.006). Headache days per month was higher at secondary headache disorders patients (p=0.049).

We compared features of chronic and episodic migraine patients. Chronic migraine patients were older (p=0.026). Disease duration was longer and allodynia was more common at chronic migraine group(p<0.001).

Expectantly headache frequency per month was higher in chronic migraine, aura was less commonly observed at chronic migraine patients (p<0.001).

**Conclusion:** This study is conducted with a large, nationally, multi center based headache clinics to evaluated incidence of Chronic Migraine. Our results is concordant with previous reports around the world and reflect high incidence of the disease. Chronic migraine is related with prominent occupational disability and medication overuse. Thus prompt action to diagnose and treat new patients to prevent complications is mandatory.

**Key words:** chronic migraine, incidence, burden, disability.

**Fig. 1 (abstract P58).**
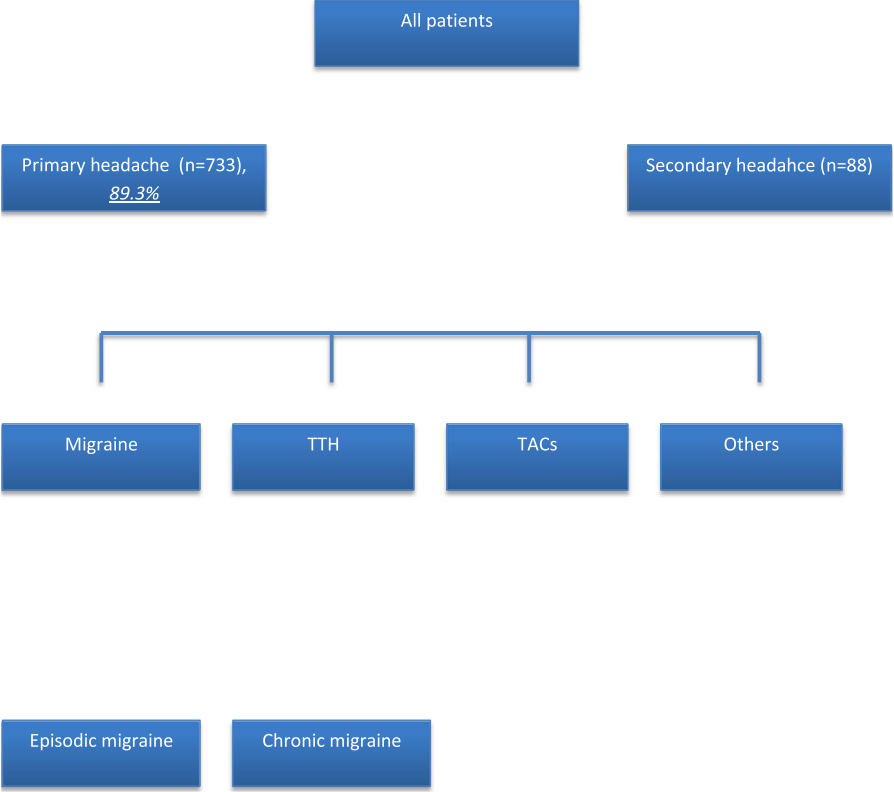
See text for description

### P59 Vestibular Migraine presenting as an acute peripheral vestibulopathy: clinical and vestibular function test profiles

#### Zeljka Calic^1,2,3^, Rachael L Taylor^4^, Allison Young^4^, Leigh M McGarvie^4^, Cecilia Cappelen-Smith^1,2,3^, Denis Cordato^1,2,3^, Miriam S Welgampola^4,5^

##### ^1^Department of Neurophysiology, Liverpool Hospital, 2170, Australia; ^2^South Western Sydney Clinical School, University of New South Wales, Sydney, 2170, Australia; ^3^Ingham Institute for Applied Medical Research, Liverpool, 2170, Australia; ^4^Institute of Clinical Neurosciences, Royal Prince Alfred Hospital, Camperdown, 2006, Australia; ^5^Central Clinical School, Sydney Medical School, University of Sydney, Camperdown, 2006, Australia

###### **Correspondence:** Zeljka Calic (zcalic@hotmail.com)

**Background**

Migraine is considered the chameleon of neurology and can present with headache, vertigo, visual and other sensory symptoms. Vestibular migraine is a common cause of acute spontaneous vertigo lasting minutes to days. We report the clinical, oculographic and vestibular test characteristics of eight subjects who presented with prolonged spontaneous vertigo exceeding 24 hours, initially thought to represent peripheral vestibulopathy.

**Methods**

The diagnosis of vestibular migraine was made according to International Headache Society and the Bárány Society criteria. A history, neuro-otological bedside examination, video head impulse testing (vHIT), cervical and ocular VEMPs (cVEMP/oVEMP) to air-conducted clicks and bone-conducted vibration, subjective visual horizontal (SVH), audiometry and Magnetic Resonance Imaging (MRI) were undertaken.

**Results**

Six subjects presented to the Emergency Room and two to a rapid access neurology outpatient facility with vertigo that exceeded 24 hours in duration. All had a history of previous episodes of vertigo associated with headaches. None had tinnitus or hearing loss. All had primary position unidirectional horizontal spontaneous nystagmus in keeping with a unilateral vestibular dysfunction. All underwent video head impulse testing, and all had horizontal semi-circular canal dysfunction with reduced gain and catch up saccades. CVEMPs were normal in 75% and asymmetric in 25%. OVEMPs were normal in 75% and asymmetric in 25%. SVH showed an abnormal bias in 57% (range 3.3°-5.9°) and normal in 43%. Audiometry and MRI brain were normal in all. Subjects were followed up over 1-7 years with no change in the final diagnosis.

**Summary**

Vestibular Migraine can sometimes present as an acute peripheral vestibulopathy with findings that mimic vestibular neuritis and should be considered in the differential diagnosis of acute prolonged vertigo.

**Ethics approval**

The study was approved by Royal Prince Alfred Hospital Research Ethics and Governance Office, approval number HREC/13/RPAH/591.

### P60 Headache in the Pediatric Emergency Department: proposal of diagnostic and therapeutic algorithm of management

#### Roberta Rossi^1^, Antonia Versace^1^, Barbara Lauria^1^, Giulia Grasso^1^, Emanuele Castagno^1^, Fulvio Ricceri^2,3^, Rosaura Pagliero^1^, Antonio Francesco Urbino^1^

##### ^1^A.O.U. Città della Salute e della Scienza di Torino, Regina Margherita Children’s Hospital, Department of Pediatric Emergency, Pediatric Headache Centre, Turin, Italy; ^2^Unit of Cancer Epidemiology, Department of Medical Sciences, University of Turin, Turin, Italy; ^3^Unit of Epidemiology, Regional Health Service, ASL TO3, Grugliasco (TO), Italy.

###### **Correspondence:** Roberta Rossi (roberta.rossi15@gmail.com)

**Background:** headache is a very frequent cause of access to the Pediatric Emergency Department (ED). The vast majority of headaches referred to the Pediatric ED are benign, due to primary form or secondary to upper airway infections [1]. The main aim of ED pediatricians is to promptly recognize headache secondary to serious organic causes that need a rapid and appropriate treatment. We propose a diagnostic-therapeutic algorithm for the management of headache in a Pediatric ED.

**Methods:** we analyzed the Literature published about pediatric headache in the ED and we select 31 articles.

**Results:** a detailed personal history and an accurate neurological examination are crucial in the evaluation of a child with headache. The clinical red flags associated to major risk of “serious headache” are: visual disturbances, cranial nerve palsy, pupillary abnormalities, nystagmus, ataxia, hyposthenia, strabismus, drowsiness and meningismus [1-3]. Neuroimaging is recommended only in patients with altered neurological examination and it should be considered in presence of a chronic progressive headache [1, 4]. The evaluation and treatment of pain are also very important and they must begin during the triage. In presence of primary headaches it is also important to give specific information to refer to Pediatric Headache Centre (PHC) within few days for the follow-up; the collaboration between ED and PHC is crucial to limit repeated visits [1, 5] (Fig. 1).

**Conclusions:** in the evaluation of pediatric headaches personal history and neurologic examination are crucial and should guide pediatricians to prescription of any diagnostic test. The evaluation and treatment of pain are also essential and the collaboration with PHC allows to ensure the follow-up for these patients and their families.

**References**

[1] Headache in the pediatric emergency department: A 5-year retrospective study. R Rossi, A Versace, B Lauria, G Grasso, E Castagno, F Ricceri, R Pagliero, AF Urbino. Cephalalgia 2018 0(0) 1–8.

[2] Acute Headache in Children and Adolescents Presenting to the Emergency Department DW Lewis, F Qureshi. Headache 2000;40:200-203.

[3] Headache with Focal Neurologic Signs in Children at the Emergency Department. D Massano et al.

J Pediatr 2014;165:376-82.

[4] Management Of Headache In The Pediatric Emergency Department. MJ Alfonzo, K Bechtel, S Babineau. Pediatric Emergency Medicine Practice. 2013. Vol 10, N 7

[5] Clinic and Emergency Room Evaluation and Testing of Headache. BL Nye, TN Ward. Headache 2015

**Fig. 1 (abstract P60).**
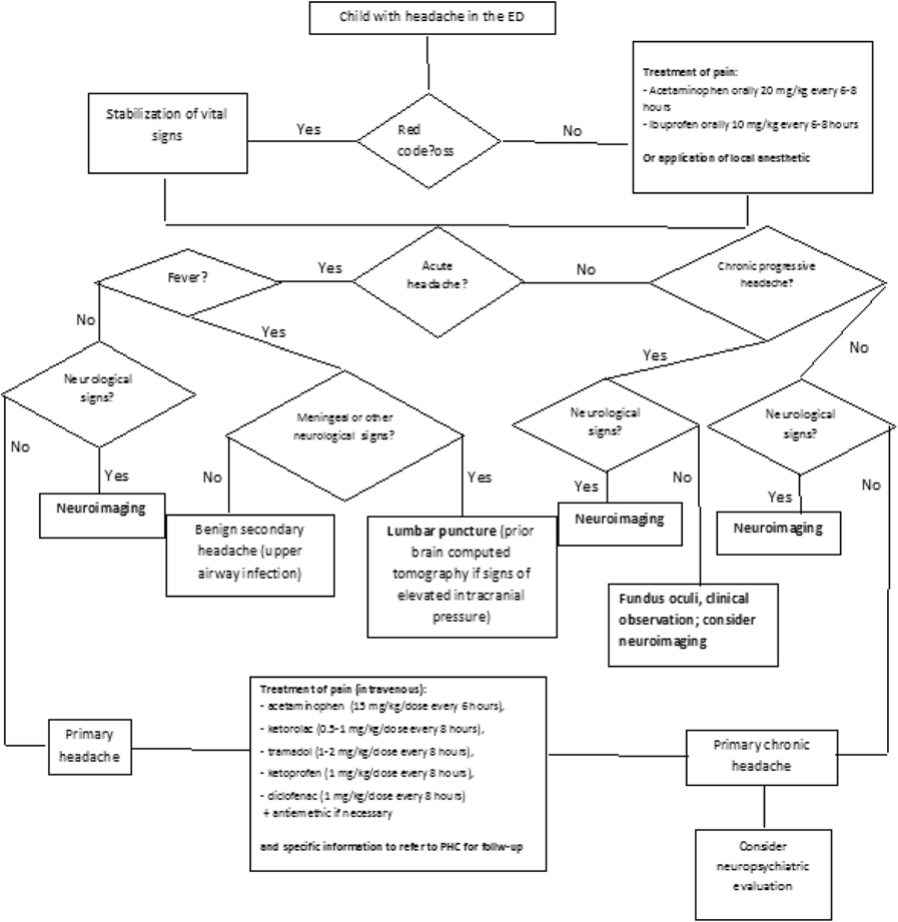
diagnostic-therapeutic algorithm for the management of headache in a Pediatric ED

### P61 Correlation between pediatric primary headache and specific learning disabilities: a case-control study

#### Ausilia Enea^1^, Antonia Versace^1*^, Barbara Lauria^1^, Giulia Grasso^1^, Roberta Rossi^1^, Emanuele Castagno^1^, Stefania Benetti^1^, Veronica Sciannameo^2,3^, Antonio Francesco Urbino^1^

##### ^1^A.O.U. Città della Salute e della Scienza di Torino, Regina Margherita Children’s Hospital, Department of Pediatric Emergency, Pediatric Headache Centre, Turin, Italy; ^2^Unit of Cancer Epidemiology, Department of Medical Sciences, University of Turin, Turin, Italy; ^3^Unit of Epidemiology, Regional Health Service, ASL TO3, Grugliasco (TO), Italy

###### **Correspondence:** Antonia Versace (aversace@cittadellasalute.to.it)

**Objective**: to investigate the connection between primary headache and specific learning disabilities in a case-control study, comparing children respectively with and without primary headache.

**Background**: headache cause substantial impact on physical and mental health in childhood, as well as on school performance and quality of life. Moreover, recent findings underline the connection between recurrent and chronic headache and learning disabilities, which seem to have high prevalence among children with primary headache syndromes, especially migraine.

**Methods**: we studied 202 children aged 3-14 years with primary headache and 202 children aged 3-14 years without headache and/or neurological and chronic disorders. We described epidemiologic and clinical features of both the populations, including specific learning disabilities, their characteristics and their correlation to different kinds of headache and other clinical features.

**Results:** specific learning disabilities were well represented among patients with headache, in particular tension type headache (20%) compared to migraine with aura (7.69%), but their prevalence was not significantly different comparing the overall children with and without headache (5.94% vs 7.92%, p=0.94). Children with specific learning disabilities and headache were significantly older than those without headache (mean age: 11.40 ± 1.62 vs 8.70 ± 2.20 years, respectively; p=0.001) and were mostly males, regardless of headache. We also found a borderline significant correlation between headache and type of specific learning disabilities (p-value=0.05): among children with headache, reading disabilities were more frequent (45.5% vs 12.5%), while writing disabilities were more represented among patients without headache (25% vs 0%). Overall, combined disorders were the most frequent in the two populations (62.5% in patients without headache and 45.5% in others). Specific learning disabilities showed especially during school age (56.25%), but also before school age in a relevant way (25%), among patients with and without headache.

**Conclusions**: we found some connections about the type of headache, age and type of specific learning disabilities. The presence of specific learning disabilities is relevant among children with headache, in particular among children with tension type headache compared to migraine with aura, supporting “the continuum model” about tension type headache and migraine without aura (mixed headaches) as part of a continuum opposed to migraine with aura. Specific learning disabilities frequently show before school age and their early diagnosis can have beneficial effects on school performance as well as on headache attacks, improving the quality of life. Moreover, it is important to evaluate the presence and the type of headache among children with specific learning disabilities.

### P62 Astrocytes contribute to the trigeminal central sensitization and cephalic cutaneous hypersensitivity in rat model of chronic migraine

#### M. Megemont, A. Descheemaeker, R. Dallel, L. Monconduit and P. Luccarini*

##### Université Clermont Auvergne, Inserm, Neuro-Dol, F-63000 Clermont-Ferrand, France

###### **Correspondence:** M. Megemont (philippe.luccarini@uca.fr)

Repeated migraine attacks are associated with maladaptive neural plasticity and lead to chronic headache. Sensitization of the pain pathways could play an important role in migraine’s pathophysiology and in the transition to the chronic forms of the disorder. In recent years, it is increasingly recognized that glial cells in the spinal and *medullary* dorsal horn (MDH), such as astrocytes, are activated in response to peripheral nerve injury or peripheral tissue injury and are involved in central sensitization of nociceptive neural networks (Ji *et al*., 2011, Lefevre *et al*., 2015). Combining behavioral and *in vivo* electrophysiological approaches, this study examined the contribution of astrocytes activity in trigeminal central sensitization and cephalic cutaneous hypersensitivity in a new model of chronic migraine (Dallel *et al*, 2018). We investigated the effect of intracisternal (i.c.) application of D-Amino-Acid-Oxidase (DAAO), a D-Serine degrading enzyme, on cephalic cutaneous sensitivity and on neuronal activation within the MDH in the rat evoked by recurrent administration of systemic administration of isosorbide dinitrate (ISDN), a nitric oxide donor (NO).

Recurrent ISDN injection (10 mg/kg, i.p., 5 injections, 1 per day) induced a persistent cephalic mechanical allodynia in vehicle-treated rats that peaked at 1 h. Conversely, DAAO-treated (0.17 UI in 5μl, i.c.) rats did not develop such cephalic mechanical allodynia. The anti-allodynia effect was significant at 30 min after ISDN administration and lasted at least 4 h (p< 0.0001). Using *in vivo* electrophysiological unitary extracellular recordings, we show that ISDN administration never affected the spontaneous activity of trigeminal wide dynamic range neurons, but facilitated C-fiber-evoked responses in 62% (18/29) of the neurons tested. The facilitation was significant from 30 min and still increased until 120 min (144 ± 3.4 % of baseline, n = 18, *P* < 0.001). Conversely, diffuse noxious inhibitory controls, quantified as an inhibitory effect on MDH neuronal firing during heterotopic immersion of one hindpaw in 49°C bath, remained *unchanged*. Interestingly, DAAO (0.17 UI in 5μl, i.c.) administration before the last ISDN injection *prevented* the *facilitation* by ISDN of *C fiber*-*evoked* activity of wide dynamic range neurons.

These results suggest that astrocytes contribute to the trigeminal central sensitization and cephalic cutaneous hypersensitivity that characterize the migraine progression.

### P63 Withdrawn

### P64 Сortical excitability in chronic migraine patients after OnabotulinumtoxinA preventive treatment

#### Vladlena Shevchenko^1^, Ada Artemenkoˡ, Mikhail Bzhiljanski^2^, Fedor Bushkov^2^, Nikolay Yahnoˡ, Alexey Kurenkov^3^

##### ^1^Research Department of Neurology, Scientific-technological Park of biomedicine, I.M. Sechenov First Moscow State Medical University, Moscow, 119991, Russia; ^2^Rehabilitation centre «Preodolenie», Moscow, Russia, 127083; ^3^Scientific Centre of Children’s Health of the Ministry of Health of the Russian Federation, Moscow, 119991, Russian Federation

###### **Correspondence:** Vladlena Shevchenkoˡ (vladashevchenko@list.ru)

**Background:** Chronic migraine (CM) is a common and disabling disease from the primary headache group, one of the migraine (M) form, characterized by 15 headache days a month or more and at least eight days of them are represented by typical M attacks [1]. The leading role in the CM pathophysiology belongs to cortical hyperexcitability [2,3]. Transcranial magnetic stimulation (TMS) is the most informative and non-invasive tool to investigate the cortical excitability and cortical inhibition processes [4]. OnabotulinumtoxinA (Botox®) is the medication with proven efficacy (level A) in CM preventive therapy. The mechanism of OnabotulinumtoxinA (Botox®) analgesic effect is not fully understood, mainly associated with its peripheral action by blocking neurogenic inflammation and proinflammatory neuropeptides transmission in sensory nerves terminals of the trigemino-cervical system, but possible central effects have not been studied [5].

The **aim** of the study: to investigate the influence of OnabotulinumtoxinA on cortical excitability after CM preventive treatment using TMS.

**Materials and methods:** This open-label, prospective study included 36 patients with CM in the interictal period (mean age 41,9±10,6 years, female/ male 93,2%/6,8%) at the Moscow University neurological clinic. The diagnose of CM was established according to ICHD-III criteria, 2018. Clinical data were obtained from headache diaries: number of headache days per month; number of attacks per month, number of days with taking medications for acute migraine treatment. We determined neurophysiological parameters using diagnostic TMS («MagPro R30» (Medtronic, Copenhagen, Denmark) with figure-of-eight coil: motor cortex thresholds (MT r/l, % of maximal stimulator output) and the cortical silent period duration (CSP r/l, ms) were measured by stimulation of the motor cortex and recording responses from abductor digiti minimi muscles r/l, using electromyograph «Keypoint» (Medtronic, Copenhagen, Denmark). Also, phosphene threshold (PT, % maximal stimulator output) was assessed by visual cortex stimulation with the same coil before and 3 months after OnabotulinumtoxinA (Botox®) injections at a dose of 195 units, according the PREEMPT paradigm [5].

**Results:** Three month after OnabotulinumtoxinA (Botox®) treatment we found the reduction of headache frequency on 11 days (p<0,05), number of attacks per month on 7 days (p<0,05), number of days with taking medications for acute migraine treatment on 13 days (p<0,05). No significant differences were observed in MT r/l and PT (MTd: before treatment 43,5±10,1%, after 44,9±9,3%, p = 0.65, MTs: before 43,9±10,7%, after 44,76±7,63%, p=0,54; PT: before 71,1±17,2%, after 72,4±12,2%, p=0,62). The duration of the CSP r/l increased significantly (CSPd: from 99,1±28,7ms to 120±30,5ms, p ≤ 0.05, CSPs: from 98,8±28,5 to 126,4±36, р≤0,05).

**Conclusion:** The results of our study showed such changes in cortical excitability as an increasing duration of the cortical silent period for both brain hemispheres after Onabotulinumtoxin A treatment what was combined with the headache frequency reduction and improvement in the patients' condition.

**References**

1) Olesen J, et al., International Classification of Headache Disorders: 3rd Edition Lancet Neurol.2018 May; 17(5):396-397.

2) Aurora SK et al.: The brain is hyperexcitable in migraine. Celphalalgia. 2007 Dec;

3) Peter J. Goadsby Pathophysiology of migraine. Ann Indian Acad Neurol. 2012 Aug; 15(Suppl 1): S15–S22.

4) Barker AT, Shields K. Transcranial Magnetic Stimulation: Basic Principles and Clinical Applications in Migraine. Headache. 2017 Mar;

5) Dodick DW et al. PREEMPT Chronic Migraine Study Group. OnabotulinumtoxinA for treatment of chronic migraine: pooled results from the double-blind, randomized, placebo-controlled phases of the PREEMPT clinical program. Headache. 2010 Jun; 50(6):921-36.

### P65 Beyond the headache phase of a migraine attack: closer look at the burden of migraine phases - results from the worldwide My Migraine Voice survey

#### Michel Lanteri-Minet^1^, Rebeca Quintana^2^, Veruska Carboni^3^, Paolo Martelletti^4, 5^, Todd J. Schwedt^6^, Hans- Christoph Diener^7^, Annik K.-Laflamme^8^, Elena Ruiz de la Torre^9^, Audrey Craven^10^, Simon Evans^11^, Donna Walsh^12^, Annette Vangaa Rasmussen^13^, Paula Dumas^14^, Rachel Fink^8^, Angela Fiorin^8^, Stephanie Ribbe^8^, Pamela Vo^8^

##### ^1^Département d’Evaluation et Traitement de la Douleur, Centre Hospitalo-Universitaire de Nice, Nice, France; ^2^GFK, Madrid, Spain; ^3^GfK Health, Basel, Switzerland; ^4^Department of Clinical and Molecular Medicine, Sapienza University of Rome, Rome, Italy; ^5^EHF; ^6^Neurology Arizona, Mayo Clinic, Phoenix, United States; ^7^Department of Neurology and Headache Center, University Duisburg-Essen, Germany; ^8^Novartis Pharma AG, Basel, Switzerland; ^9^European Headache Alliance, Brussels, Belgium; ^10^Migraine Association Ireland, Dublin, Ireland; ^11^Migraine Action, Leicester, United Kingdom; ^12^European Federation of Neurological Associations, Brussels, Belgium; ^13^Rigshospitalet Glostrup, Copenhagen, Denmark; ^14^Migraine Again, United States

###### **Correspondence:** Michel Lanteri-Minet (lanteri-minet.m@chu-nice.fr)

**Introduction**

Limited evidence exists on the quantitative burden associated with premonitory and postdromal phases of the migraine attack. This study aimed to evaluate the burden of migraine as described by patients in these specific migraine phases: premonitory, headache, and postdromal.

**Methods**

My Migraine Voice is a cross-sectional study conducted using an online survey of 11,266 migraine patients (31 countries across Africa, America, Asia and Europe) recruited via online panels and patient organizations. Participants were adult migraine patients per ICHD-3 criteria, who reported having had >=4 migraine days/month in the 3 months preceding survey administration, with pre-specified 90% having reported having used preventive migraine treatment.

**Results**

A total of 11,266 migraine patients responded to the survey and reported that, over the last 3 months, they had on average 9.8 migraine days/month; 37% reported being affected by migraine for >15 years. 44% of respondents’ migraine attack lasted one day or more (19% reported longer than 3 days). Half of patients (49%) reported feeling limited throughout the 3 migraine phases. Almost 1/3 of patients (29%) reported feeling very to extremely limited during the premonitory phase, 71% during the headache phase and 30% in the postdromal phase (Table 1).

**Conclusion**

This study is the first to quantify the burden experienced by patients during the different phases of migraine. While the attack itself caused the highest degree of impairment, the findings demonstrate that the burden of migraine extends beyond the headache phase and is higher for patients who have failed >=1 preventive treatment.

**Ethics approval:**

Data was handled confidentially and anonymity of respondents was maintained throughout the study. Participants’ consent was obtained prior to participation in the survey.

**Funding**

This study was funded by Novartis Pharma AG, Basel Switzerland

**Table 1 (abstract P65).** Description of the migraine phases

Proportion of respondents (%)	Premonitory phase	Headache phase	Postdromal phase
Duration of phase			
< 4 hours	51%	20%	30%
4-24 hours	30%	45%	40%
>24 hours	14%	34%	27%
Not experiencing this phase	6%	1%	3%
Duration of the phase >24 hours in migraine patients with:			
- no failure to migraine preventive treatment	11%	27%	20%
- 1 failure to migraine preventive treatment	11%	29%	23%
- ≥2 failures to migraine preventive treatment	17%	38%	30%
Feeling very to extremely limited during the phase	29%	71%	30%

### P66 Withdrawn

### P67 Carbon monoxide inhalation induces headache but no migraine in patients with migraine without aura

#### Hashmat Ghanizada^1^, Nanna Arngrim^1^, Henrik Winther Schytz^1^, Jes Olesen^1^, Messoud Ashina^1^

##### ^1^Danish Headache Center and Department of Neurology, Rigshospitalet Glostrup, Faculty of Health and Medical Sciences, University of Copenhagen, Valdemar Hansens vej 5, DK-2600 Glostrup, Denmark

###### **Correspondence:** Messoud Ashina (ashina@dadlnet.dk)

This abstract was not included as it has been previously published [1].

**Reference:**

[1] Ghanizada H, Arngrim N, Schytz HW, Olesen J, Ashina M. Carbon monoxide inhalation induces headache but no migraine in patients with migraine without aura. Cephalagia. 2018: 333102418765771. doi: 10.1177/0333102418765771

### P68 Demographic and clinical characteristics of patients with trigeminal neuralgia assisted by neurologists in an Emergency Department

#### Javier Camiña Muñiz, Germán Ferrer Juan, María Magdalena Rosselló Vadell, Antonio José Moreno Rojas, Francisco José Molina Martínez

##### Department of Neurology. Hospital Universitari Son Espases, Carretera de Valldemossa, 79. 07120, Palma, Illes Balears, Spain

###### **Correspondence:** Javier Camiña Muñiz (javier.camina.muniz@gmail.com)

**Background:** Trigeminal neuralgia is characterized by recurrent brief episodes of severe paroxysmal pain often needing medical treatment in Emergency Departments in cases of severe outbreaks of symptoms, with antiepileptic and non-antiepileptic drugs.

**Objectives**: To describe the demographic, clinical and therapeutical characteristics of 100 patients with acute exacerbations of trigeminal neuralgia treated in an Emergency Department. To analyze possible risk factors and predictors of these exacerbations.

**Methods:** We have retrospectively searched for patients with a diagnosis of trigeminal neuralgia by the clinical system of the Emergency Department (Firstnet® of Millennium). Demographic and clinical characteristics of the patients are collected from the clinical history by the Millennium Powerchart® clinical system.

**Results:** One hundred (100) patients with acute exacerbation of trigeminal neuralgia were treated in Emergency Department in 153 episodes between May 2016 and May 2018. They had an average age of 57 years, 64.60% were women and 34.68% needed more than 1 visit over this period. In 16.34% of the cases hospitalization was required for pain management.

**Conclusion:** Around 20% of patients with craniofacial pain assisted by Neurology in our Emergency Department have trigeminal neuralgia. One of the clinical objectives in trigeminal neuralgia patients should be to reduce the probability of admission by optimizing their outpatient treatment.

### P69 Systematic review of the safety and efficacy of Occipital Nerve Stimulation for intractable chronic cluster headache

#### Iris Smet^1^, Jean-Pierre Van Buyten^1^, Michel Lanteri-Minet^2^, Denys Fontaine^3^, Jennifer Tinsley^4^, Brooke Kelley^5^

##### ^1^ Multidisciplinary Pain Centre AZ Nikolaas, Sint -Niklaas, 9100, BE; ^2^ Pain Department CHU Nice, FHU INOVPAIN, Côte Azur University, 06001, Nice, FR; ^3^ Department of Neurosurgery, CHU de Nice, FHU INOVPAIN, University Cote d’Azur, 06001, Nice, FR; ^4^ Medtronic International Trading Sàrl, Clinical Research, Tolochenaz, 1131 CH; ^5^ Medtronic Plc, Clinical Research, Minneapolis, Minnesota, 55432, US

###### **Correspondence:** Jennifer Tinsley (jennifer.tinsley@medtronic.com)

**Background:** Occipital Nerve Stimulation (ONS) consists of applying mild electrical stimulation to the occipital nerves via leads implanted under the skin. An implanted battery generates the stimulation.

**Methods:** A literature search in MEDLINE and EMBASE was conducted to evaluate the safety of ONS (to treat any headache condition) and the performance of the therapy to treat Intractable Chronic Cluster Headache (iCCH). The search used a combination of peripheral nerve locations, and neurostimulation and headache key terms (timeframe 01-Jan-1990 through 30-AUG-2017).

**Results:** Of 820 results, 71 met selection criteria. Twenty-one publications described ONS for iCCH. There were 7 unique studies where performance outcome data could be pooled:

- 61.8% of patients reduced attack frequency by ≥30% (5 studies; 110 patients) and 54.9% reduced by ≥50% (5 studies; 164 patients)
- 39.3% of patients had reduced CH intensity score of ≥30% (5 studies; 56 patients)
- mean 41.8% reduction in attack duration (2 studies; 78 patients)
- 43.1% of patients reduced medications to some extent (7 studies; 188 patients)

Thirteen prospective studies comprising 575 unique patients with an average 37.5 months follow-up were included. A total of 469 adverse events (AEs) were reported with an AE rate (number of AEs/number of subjects) of 81.6% overall (range 12.5-130.6%). Most common events were lead migration (10.1%), persistent pain at device site (11.3%), undesirable change in stimulation (11.3%), infection (7.7%), wound complications (3.7%) and skin erosion (3.5%).

**Conclusions:** While efficacy of ONS for iCCH appears promising, the complication rate shows a need for improved technology and implant techniques.

### P70 Safety data from phase 3 clinical studies comparing galcanezumab and placebo in patients with episodic and chronic migraine

#### Virginia L. Stauffer, Shufang Wang, Mark E. Bangs, Tina M. Oakes, Jeffrey N. Carter, Sheena K. Aurora

##### Lilly Research Laboratories, Indianapolis, IN, USA

###### **Correspodence:** Jeffrey N. Carter (carter_jeffrey_n@lilly.com)

**OBJECTIVE:** To evaluate the safety and tolerability of galcanezumab compared with placebo each given monthly (subcutaneous injection) for up to six months for prevention of migraine.

**METHODS:** Data were integrated from three double-blind clinical studies (EVOLVE-1=NCT02614183; EVOLVE-2=NCT02614196; REGAIN=NCT02614261); two galcanezumab dose-groups (120- and 240-mg) were pooled. Adverse events (AEs) that were treatment-emergent (TEAEs), discontinuation due to AEs (DCAEs), and serious AEs (SAEs) were analyzed. Laboratory results, vital signs, and ECG results were also assessed. Calcitonin gene-related peptide is a potent vasodilator and believed to play a protective role in cardiovascular (CV) health. People with migraine often have CV risk; therefore, patients with comorbid CV disease and risk based on medical history or pre-existing conditions were evaluated. The studies were approved by the appropriate Institutional Review Board for each study site.

**RESULTS:** A total of 1,435 patients were treated with galcanezumab and 1,451 with placebo. At baseline, 18.5% of placebo- and 17.2% of galcanezumab-treated patients reported CV risk factors. During the treatment period, TEAEs occurring in 1.5% or more of galcanezumab-treated patients, more frequently than among placebo-treated patients, and significantly different between galcanezumab and placebo included nasopharyngitis, injection site reaction, injection site erythema, injection site pruritus, and constipation (Table 1). The proportion of DCAEs among galcanezumab-treated patients was low (Table 2), and the proportion of patients who discontinued due to an injection-site related AE was less than 0.5%. None of the TEAEs related to injection site were reported as an SAE, and the majority of patients reported the events as mild or moderate in severity. Fewer than 2.0% of galcanezumab-treated patients reported an SAE (Table 2). There were no significant differences between galcanezumab- and placebo-treated patients in the frequency of serious CV TEAEs, or discontinuations due to CV TEAEs. There were no significant treatment-by-cardiovascular disease subgroup interactions for CV TEAEs. There were no clinically meaningful differences between galcanezumab- and placebo-treated patients in laboratory analytes, vital signs, ECGs, or cardiovascular-related TEAEs.

**CONCLUSION:**

Galcanezumab (120- and 240-mg monthly) demonstrated a favorable safety and tolerability profile for the prevention of episodic and chronic migraine.

**Table 1 (abstract P70).** Treatment-emergent adverse events reported by 1.5% or more patients in any galcanezumab dose-group.

		Galcanezumab		
Event	Placebo N=1451 n (%)	120-mg N=705 n (%)	240-mg N=730 n (%)	Combined N=1435 n (%)
Injection site pain	138 (9.5)	71 (10.1)	85 (11.6)	156 (10.9)
Nasopharyngitis	94 (6.5)	52 (7.4)	31 (4.3)^†^	83 (5.8)
Upper respiratory tract infection	60 (4.1)	31 (4.4)	36 (4.9)	67 (4.7)
Injection site reaction	14 (1.0)	22 (3.1)^‡^	45 (6.2)^‡^	67 (4.7)^‡^
Dizziness	41 (2.8)	20 (2.8)	20 (2.7)	40 (2.8)
Injection site erythema	20 (1.4)	20 (2.8)^†^	29 (4.0)^‡^	49 (3.4)^‡^
Sinusitis	31 (2.1)	20 (2.8)	19 (2.6)	39 (2.7)
Urinary tract infection	33 (2.3)	19 (2.7)	18 (2.5)	37 (2.6)
Fatigue	34 (2.4)	17 (2.4)	16 (2.2)	33 (2.3)
Injection site pruritus	2 (0.1)	15 (2.1)^‡^	24 (3.3)^‡^	39 (2.7)^‡^
Neck pain	21 (1.5)	15 (2.1)	6 (0.8)	21 (1.5)
Abdominal pain	24 (1.7)	13 (1.8)	6 (0.8)	19 (1.3)
Cough	19 (1.3)	12 (1.7)	13 (1.8)	25 (1.7)
Oropharyngeal pain	13 (0.9)	10 (1.4)	12 (1.6)	22 (1.5)
Bronchitis	17 (1.2)	9 (1.3)	11 (1.5)	20 (1.4)
Influenza	34 (2.3)	8 (1.1)	20 (2.7)	28 (2.0)
Constipation	8 (0.6)	7 (1.0)	11 (1.5)^†^	18 (1.3)^†^
Migraine	14 (1.0)	7 (1.0)	12 (1.6)	19 (1.3)

^†^Indicates *P* < .05 compared with placebo

^‡^Indicates *P* < .001 compared with placebo

**Table 2 (abstract P70).** Overview of adverse events.

		Galcanezumab		
Event	Placebo N=1451 n (%)	120-mg N=705 n (%)	240-mg N=730 n (%)	Combined N=1435 n (%)
Deaths	0	0	0	0
SAE	14 (1.0)	12 (1.7)	11 (1.5)	23 (1.6)
DCAE	24 (1.7)	13 (1.8)	22 (3.0)^†^	35 (2.4)
TEAE	827 (57.0)	441 (62.6)^†^	472 (64.7)^‡^	913 (63.6)^‡^

*Abbreviations: SAE* Serious adverse events, *DCAE* discontinuation due to an adverse event, *TEAE* Treatment-emergent adverse events

^†^Indicates *P* < .05 compared with placebo

^‡^Indicates *P* < .001 compared with placebo

### P71 OnabotulinumtoxinA: How and when do our patients improve?

#### A.Alpuente^1,2^, VJ. Gallardo^2^, M.Torres-Ferrús^1,2^, J.Álvarez-Sabín^1^, P.Pozo-Rosich^1^

##### ^1^Headache Unit, Neurology Department, Hospital Universitari Vall d'Hebron, Barcelona, Spain; ^2^Headache Research Group, Vall d'Hebron Research Institute (VHIR), Universitat Autònoma de Barcelona, Barcelona, Spain

###### Correspondence: P.Pozo-Rosich (ppozorosich@yahoo.com)

**Objective:** To analyze the clinical characteristics of a long-term follow-up of patients with migraine in treatment with onabotulinumtoxinA.

**Methods:** A three-year prospective observational study. We included patients diagnosed with chronic migraine (CM) and high-frequency episodic migraine (HFEM) according to ICHD-3 beta. We collected clinical data. A comparative analysis was carried out at 6, 12, 18 and 24 months identifying response variables (frequency, intensity, analgesics intake) according to treatment and diagnosis time.

**Results:** Data was collected from 534 patients (84.6% women, mean age 46.9±12.8 years): 80.1% CM and 19.9% HFEM, 59.9% had medication-overuse (MOH) and 81.2% had severe disability (MIDAS). After 6 months (n=352), average headache frequency improved (-42.0%±39.1 in MC). In the following months, this reduction continued with a stepwise slope: -45.4% ± 34.2 (12 months), -48.6% ± 38.5 (18 months) and 59.4% ± 29.8 (24 months). Likewise, in CM patients, we observed and improvement in intensity greater than 50% during the follow-up (46.9% at 6 months, 50.0% at 12 months, 58.6% at 18 months, 63.9% at 24 months) and analgesics intake (81.7±59.3% at 6 months). There was also an statistically significant improvement in headache frequency, intensity and analgesics intake in HFEM patients.

An improvement in the anxiety and depression scales (HAD) at 6 months of treatment (19.0 ± 1.2 vs. 10.0 ± 5.0 with p-value <0.01, 15.8% ± 5.1% vs 7.8 ± 5.2 with p-value <0.05) was observed, and this remained stable during the rest of follow-up.

During the 24 months of follow-up, 23.2% discontinued treatment because of: clinical improvement (20.9%-CM vs. 34.2%-HFEM), lack of efficacy (46.5%-CM vs. 31.6%-HFEM) and other medical reasons (32.6%-CM vs. 34.2%-HFEM).

**Conclusion:** In our cohort, onabotulinumtoxinA efficacy is significant at 6 months, with further moderate progressive improvement at medium term.

The improvement in CM and HFEM is proportional and significant in terms of headache frequency, intensity, and analgesic intake.

This improvement is correlated with less anxiety and depression.

### P72 The role of foods with a high glycemic index in migraine patients: a real life preliminary study

#### Gianluca Cecchi, Maria Bravo, Paola Di Caprio, Giorgia Botti, Nicoletta Brunelli, Matteo Paolucci, Manon Khazrai, Fabrizio Vernieri, Claudia Altamura

##### Headache and Neurosonology Unit, Neurology Unit, Università Campus Bio-Medico di Roma, Rome, Italy.

###### **Correspondence:** Gianluca Cecchi (gianlucacecchi250489@gmail.com)

**Introduction**

Migraine is one of the main causes of disability according to the WHO. [1] Numerous progresses have been accomplished in the individuation of the subtending pathogenetic mechanisms but there are still lands to be discovered. Different predisposing and trigger factors have been identified, among these an important role is played by an incorrect diet. [2]This study aimed at evaluating retrospectively the relationship between dietary habit, and migraine frequency and disability.

**Methods**

We enrolled 149 consecutive patients (mean age 41 ys SD 13.2, 86% female) affected by Migraine with or without Aura referring to our Headache Centre. All patients underwent anthropometric assessment and filled a Frequency Food Questionnaire (FFQ) to assess their dietary habit and clinical scales (BS11, PPI, BRS6, MIDAS e HIT-6) in the previous three months. All data were analysed with SPSS 25.0. The study was approved by our Local Ethical committee (prot 6.18TS CBM). Informed consent to publish has been obtained from patients.

**Results**

The analysis of baseline data showed average BMI 24.6 ± 4 kg/m^2^ and Waist Circumference 83 cm; mean headache days per month 9.3 ± 7, abortive drugs per month 9.2 ± 7, MIDAS TOT 25.6 ± 33, MIDAS-A TOT 20,7

± 18,6, MIDAS-B TOT 6.3 ± 1.9, HIT6 TOT 63.6 ± 7, BS-11 TOT 7.5 ± 1.9, PPI TOT 3.4 ± 1, BRS 6 TOT 3.7 ± 1.

We observed a correlation between sweet confectionery product (croiassant, biscuits, sweets and spreads) weekly intake and BS-11 (Spearman Rho= 0.178, p= 0.04), and PPI(Spearman Rho= 0.181, p= 0.037), MIDAS- tot (Spearman Rho= 0.189, p= 0.021) and HIT6 (Spearman Rho= 0.231, p= 0.005). Weekly rice consumption had a correlation with MIDAS-B (Spearman Rho=0.223, p=0.01). Likewise the consumption of potatoes had a correletion beetween MIDAS- B (Spearman Rho=0.216, p=0.022) and HIT6 (Spearman Rho=0.200, p=0.034). No other relation were observed between headache frequency and disability and all other foods and drinks.

**Discussion and conclusion**

Literature data hypothesize that changes of blood glucose values may play a role in the pathogenesis of fasting headaches. Martins-Oliveria et all highlighted that glycemic neuroendocrine signaling modulates specific neural networks can alter the transmission of trigeminal nociceptive inputs [3]. Our study supports this hypothesis: patients consuming more frequently high-glycemic foods tend to score higher on disability scales. We can speculate that high-glycemic foods amplify glucose and insulin blood oscillations, producing unstable neuroendocrine signalling.

**References**

1. Global, regional, and national incidence, prevalence, and years lived with disability for 301 acute and chronic diseases and injuries in 188 countries, 1990-2013: a systematic analysis fot the Global Burden of Disease Study 2013. 2015 Aug 22; 386 (9995):743-800.

2. Dalcara T. How does fasting trigger migraine? A hypothesis. Curr Pain Headache Rep. 2013;17(10):368)

3. Martins-Oliveira M, Akerman S, Holland PR, et al (2017) Neuroendocrine signaling modulates specific neural networks relevant to migraine. Neurobiol Dis 101:16–26 . doi: 10.1016/j.nbd.2017.01.005

### P73 Do evidence-based practice guidelines exist to support physiotherapists in the approach of patients with episodic headache?

#### Sarah Mingels^1,2^, Marita Granitzer^1^

##### ^1^REVAL Rehabilitation Research Centre, Biomedical Research Institute, Faculty of Medicine and Life Sciences, Hasselt University, Belgium; ^2^Musculoskeletal Research Unit, Department of Rehabilitation Sciences, Faculty of Kinesiology and Rehabilitation Sciences, Leuven University, Belgium

###### **Correspondence:** Sarah Mingels (sarah.mingels@uhasselt.be)

**Background**

The International Classification of Headache Disorders provides an extensive framework to classify headaches as primary or secondary. Physiotherapy is indicated if neuro-musculoskeletal dysfunctions are assumed to be related to the pathophysiological process. Mostly patients suffering from episodic migraine, cervicogenic and tension-type headache consult physiotherapists. Various interventions such as manual therapy, relaxation and exercise therapy are applied in such patients. Yet, clinical outcomes following physiotherapy tend to vary (Fig. 1).

**Methods**

National and international physiotherapy guidelines concerning the treatment of headache were searched in the databases Pubmed, Web of Science, Pedro and the Cochrane library from January to May 2017. The following Topics or Medical subject heading terms were combined: 'Headache', 'Adult', 'Physiotherapy or Physical Therapy', '(EBM/EBP) Guidelines' and 'Recommendations'. Guidelines as well as meta-analyses and (systematic) reviews in English and Dutch were included.

**Results**

Guidelines focus primarily on the pharmacological management of headache. From only two evidence-based physiotherapy guidelines it was concluded that effectiveness of interventions will depend on clinical reasoning since not all interventions are equally effective for all headache types (Fig. 2).

**Fig. 1 (abstract P73).**
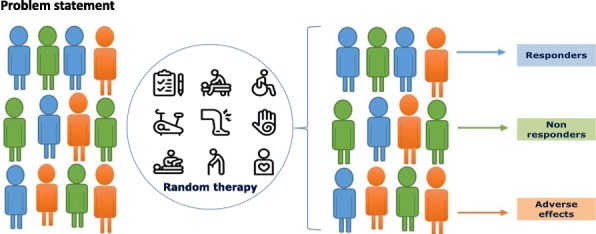
Visualisation of the consequence of random physiotherapeutic interventions in patients with headache

**Fig. 2 (abstract P73).**
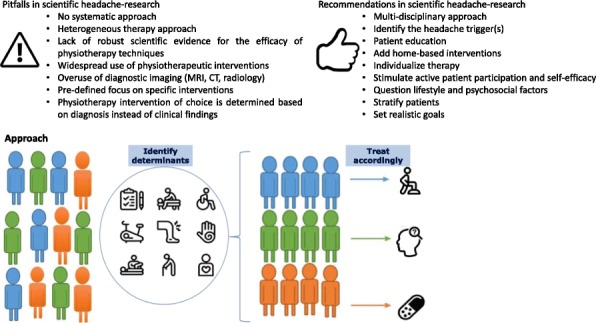
Visualisation of determinant-based physiotherapeutic interventions in patients with headache

### P74 Efficacy and safety of a sequential surgical algorithm in 44 patients with refractory chronic cluster headache

#### Robert Belvís, Marina Guasch, Paula Marrero, Maria Jesus Alvarez, Rodrigo Rodriguez, Joan Molet, Carles Roig

##### Department of Neurology and Department of Neurosurgery. Hospital de la Santa Creu i Sant Pau. Barcelona. Spain

###### **Correspondence:** Robert Belvís (rbelvis@santpau.cat)

Several surgical procedures have been proposed when chronic cluster headache (CCH) becomes refractory (r). We usually perform in order of safety: ipsilateral sphenopalatine ganglion radiofrequencies (SPG-Rf), bilateral occipital nerve stimulation (NOM-S) and deep brain stimulation (DBS).

**Patients.**

We included patients with rCCH according to ICHD3 / EHF criteria between November 2003 - June2018 (mean follow-up: 87.4 months). End-point of efficacy: reduction superior to 50% of headache attacks / day.

**Results.**

We performed 74 SPG-Rf procedures in 44 patients (man: 70.4%, mean age: 41.2y). Average of headache attack / day: 4.4, and mean triptans / day consumption: 3.4. Efficacy of SPG-Rf: 33.3%. SPG-Rf reduced NRS score (10/10 points-to-7/10 points) and triptans consumption (20.5%). Safety: vaso-vagal syncope during the procedure (1) and palatal hypoaesthesia as a sequel (1).

We implanted bilateral NOM-S devices in 22 of the 29 SPG-Rf-refractory patients. Average of headache attack / day: 5.7, main sc sumatriptan / day consumption: 4.4. Efficacy of NOM-S: 50%. NOM-S reduced NRS score (10/10 points-to-6/10 points) and triptans consumption (50%). Three patients (13.6%) have been able to shut down the system. Complications: system infection-4, broken electrode-1, disconnected electrode-1.

Finally, 7 patients (man: 87.7%, mean age: 44.6y) were subjected to DBS (posterior hypothalamus), five of them due inefficacy of NOM-S and two more directly after SPG-Rf because NOM-S did not exist then. Average of headache attack / day: 6.5, mean sc sumatriptan / day consumption: 5.7. Efficacy: 85.7%. DBS reduced NRS score (10/10 points-to-4.5/10 points) and triptans consumption (74.5%). Only one patient (14.3%) has been able to shut down the system. Complications: broken electrode-1, relocation of electrode-1.

**Conclusions.**

The 86% of our refractory chronic cluster headache patients improved their quality of life, substantially and in a sustained way, after applying this surgical algorithm sequentially. We did not register serious adverse effects or relevant surgical complications.

### P75 Spectral domain optical coherence tomography findings in RVCL-S, a monogenic vascular migraine model

#### Irene de Boer^1^, Sylvie R. Steenmeijer^2^, Nadine Pelzer^1^, Greet Dijkman^2^, Irene C. Notting^2^ , Gisela M. Terwindt^1^

##### ^1^ Department of Neurology, Leiden University Medical Center, Leiden, 2300 WB, The Netherlands; ^2^ Department of Ophthalmology, Leiden University Medical Center, Leiden, 2300 WB, The Netherlands

**Correspondence:** Irene de Boer (ideboer@lumc.nl)

**Background**

Retinal Vasculopathy with Cerebral Leukoencephalopathy and Systemic manifestations (RVCL-S) is a monogenic small vessel disease caused by mutations in *TREX1*.[1] Several organs, including the brain and retina, are affected, most likely due to endothelial dysfunction.[2] One of the striking associated neurological symptoms is migraine. Migraine has a complex genetic background, but by using monogenic, homogenous, pathophysiological models we aim to increase our knowledge of migraine pathophysiology. One of such models for studying migraine is RVCL-S. Because the retina is a peripheral extension of the brain and shares a similar embryological origin, analyzing retinal anatomy is increasingly seen as a useful biomarker for multiple neurological disorders.[3-6] Optical coherence tomography (OCT) provides a non-invasive high-resolution visualization of the optic disc and retinal layers by creating cross-sectional images.[7] The aim of this study was to investigate retinal layer pathology as a possible biomarker for the migraine model RVCL-S.

**Methods**

Seventeen *TREX1* mutation carriers and nine unrelated controls were included and examined by spectral domain optical coherence tomography (SD-OCT). Peripapillary retinal nerve fiber layer (pRNFL) thickness and total macular volume (TMV) were determined. Secondary outcomes were total macular thickness (TMT) and the thickness of individual retinal layers of the macula and peripapillary sectors of the pRNFL. SD-OCT measures of patients (n=34 eyes) and controls (n=18 eyes) were compared using generalized estimating equations (GEE) to account for intereye correlation.

**Results**

We found a decrease in pRNFL thickness (79.3 ±22.2 vs 99.5 ±11.8μm, p<0.001), and TMV (8.0 ±0.8 vs 8.6 ±0.4mm3, p=0.006) in *TREX1* mutation carriers compared to controls (Fig. 1), which may occur at a relatively young age (Fig. 2). With the exception of the temporal sector, the thickness of all peripapillary sectors were decreased in *TREX1* mutation carriers (Table 1). In *TREX1* mutation carriers the TMT (292.0 ±32.1 vs 313.8 ±15.1μm, p=0.009) and individual layers in the macular area, ganglion cell layer (29.6 ±9.5 vs 38.3 ±3.2μm, p<0.001) and inner plexiform layer (27.9 ±6.2 vs 33.1 ±2.1 μm, p=0.001), were shown to be thinner than in controls (Table 2).

**Conclusion**

RVCL-S is associated with retinal thinning in the peripapillary and macular area. SD-OCT may serve as a mirror of the brain, therefore it will be interesting to see if these retinal changes mirror disease progression cerebrally. Furthermore, these retinal measurements might improve our understanding of several diseases for which RVCL-S serves as a model, including migraine.

**Ethics Approval**

The study was approved by Leiden University Medical Center Institution’s Ethics Board, approval number: P14-299.

**Consent to publish**

Informed consent to publish has been obtained from the patients

**References**

1. Stam AH, Kothari PH, Shaikh A, Gschwendter A, Jen JC, Hodgkinson S, et al. Retinal vasculopathy with cerebral leukoencephalopathy and systemic manifestations. Brain : a journal of neurology. 2016.

2. Pelzer N, Bijkerk R, Reinders MEJ, van Zonneveld AJ, Ferrari MD, van den Maagdenberg AMJM, Eikenboom J, Terwindt GM. Circulating Endothelial Markers in Retinal Vasculopathy With Cerebral Leukoencephalopathy and Systemic Manifestations. Stroke. 2017 Dec;48(12):3301-3307.

3. Cunha LP, Almeida AL, Costa-Cunha LV, Costa CF, Monteiro ML. The role of optical coherence tomography in Alzheimer's disease. International journal of retina and vitreous. 2016;2:24.

4. Alten F, Motte J, Ewering C, Osada N, Clemens CR, Kadas EM, et al. Multimodal retinal vessel analysis in CADASIL patients. PLoS One. 2014;9(11):e112311.

5. Talman LS, Bisker ER, Sackel DJ, Long DA, Jr., Galetta KM, Ratchford JN, et al. Longitudinal study of vision and retinal nerve fiber layer thickness in multiple sclerosis. Ann Neurol. 2010;67(6):749-60.

6. Iseri PK, Altinas O, Tokay T, Yuksel N. Relationship between cognitive impairment and retinal morphological and visual functional abnormalities in Alzheimer disease. J Neuroophthalmol. 2006;26(1):18-24.

7. Drexler W, Morgner U, Ghanta RK, Kartner FX, Schuman JS, Fujimoto JG. Ultrahigh-resolution ophthalmic optical coherence tomography. Nature medicine. 2001;7(4):502-7.

8. Feng YF, Guo H, Huang JH, Yu JG, Yuan F. Retinal Nerve Fiber Layer Thickness Changes in Migraine: A Meta-Analysis of Case-Control Studies. Curr Eye Res. 2016 Jun;41(6):814-22.

9. Tan FU, Akarsu C, Güllü R. Retinal nerve fiber layer thickness is unaffected in migraine patients. Acta Neurol Scand. 2005 Jul;112(1):19-23.

10. Reggio E, Chisari CG, Ferrigno G, Patti F, Donzuso G, Sciacca G, Avitabile T, Faro S, Zappia M. Migraine causes retinal and choroidal structural changes: evaluation with ocular coherence tomography. J Neurol. 2017 Mar;264(3):494-502.

**Fig. 1 (abstract P75).**
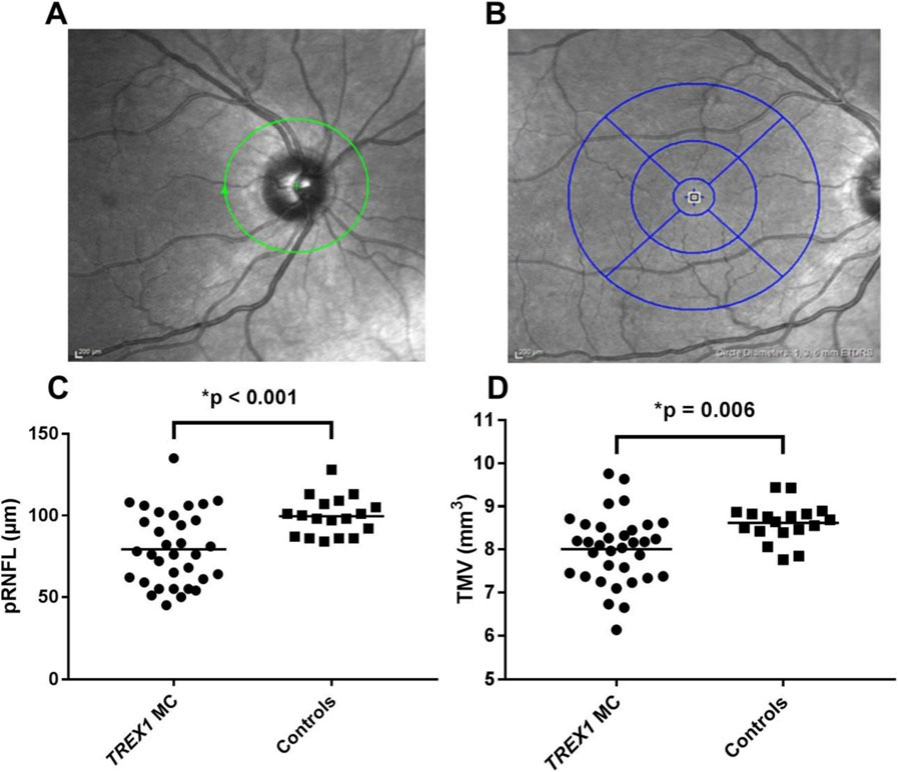
Peripapillary retinal nerve fiber layer thickness and total macular volume in *TREX1* mutation carriers. Area of measurement peripapillary retinal nerve fiber layer (pRNFL) thickness (A) and total macular volume (TMV) (B). Mean pRNFL (C) and TMV (D) were reduced in *TREX1* mutation carriers compared to controls.

**Fig. 2 (abstract P75).**
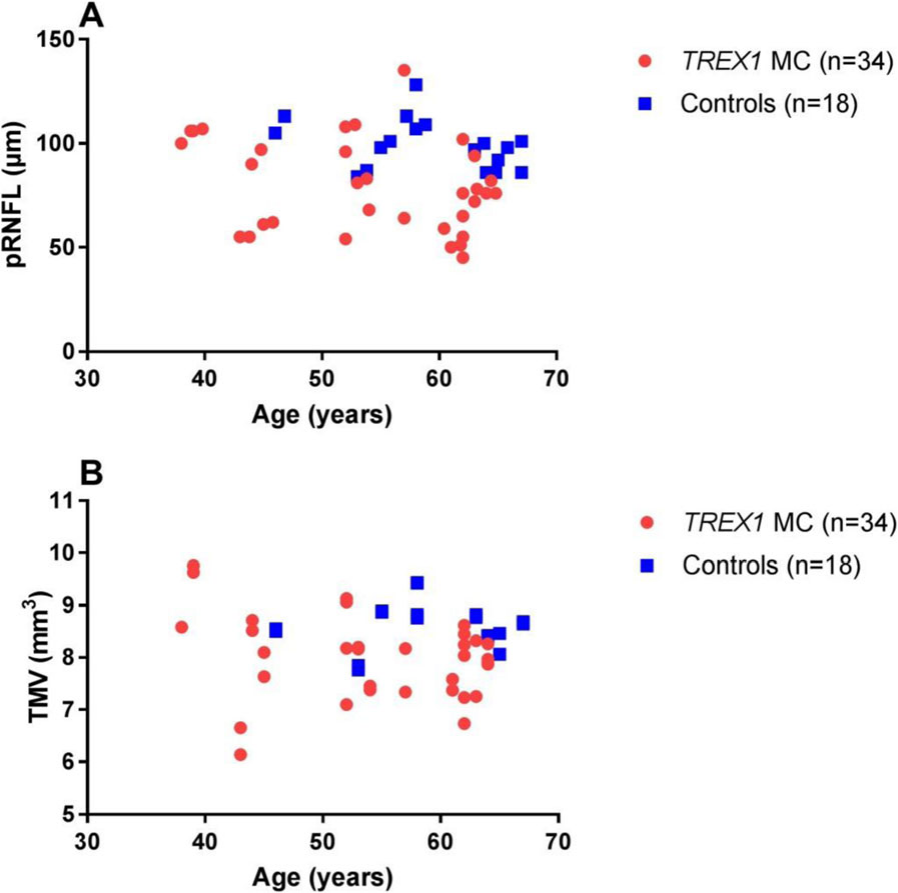
Distribution of peripapillary retinal nerve fiber layer thickness (A) and total macular volume (B) with age in *TREX1* mutation carriers and controls. Mutation carriers (MC); Peripapillary retinal nerve fiber layer (pRNFL); total macular volume (TMV).

**Table 1 (abstract P75).** Thickness of peripapillary sectors of the pRNFL in *TREX1* mutation carriers and controls

	*TREX1* MC mean ±SD (n=33^a^)	Controls mean ±SD (n=18)	Estimate	CI	P-value
Nasal sup. (μm)	84.4 **±**34.0	107.7 **±**26.2	-23.3	-42.0;-4.7	0.014*
Nasal (μm)	61.2 **±**21.4	87.7 **±**25.4	-26.5	-42.8;-10.1	0.002*
Nasal inf. (μm)	84.7 **±**34.6	118.7 **±**30.6	-34.4	-56.6;-12.2	0.002*
Temporal inf. (μm)	102.1 **±**37.9	133.8 **±**20.0	-32.6	-52.7;-12.5	0.001*
Temporal (μm)	61.7 **±**26.0	63.1 **±**10.0	-1.5	-12.5;-9.5	0.79
Temporal sup. (μm)	109.7 **±**37.4	131.1 **±**16.6	-21.6	-39.1;-4.1	0.016*

Mutation carrier (MC); standard deviation (SD); confidence interval (CI); Peripapillary retinal nerve fiber layer (pRNFL). ^a^ In one patient measurements of one eye were not possible due to the patient’s inability to focus. * p <0.05 tested by GEE

**Table 2 (abstract P75).** Thickness of the macula layers in *TREX1* mutation carriers and controls.

	*TREX1* MC mean ±SD (n=34)	Controls mean ±SD (n=18)	Estimate	CI	P-value
TMT (μm)	292.0 **±**32.1	313.8 **±**15.1	-21.8	-38.1;-5.6	0.009*
mRNFL (μm)	23.0 **±**6.3	25.7 **±**2.2	-2.7	-5.6;-0.2	0.07
GCL (μm)	29.6 **±**9.5	38.3 **±**3.2	-8.7	-13.2;-4.2	<0.001*
IPL (μm)	27.9 **±**6.2	33.1 **±**2.1	-5.1	-8.1;-2.2	0.001*
INL (μm)	33.9 **±**5.3	35.7 **±**3.5	-1.8	-4.8;-1.3	0.25
OPL (μm)	28.6 **±**2.8	28.9 **±**2.2	-0.3	-1.8;-1.2	0.70
ONL (μm)	69.8 **±**9.5	70.0 **±**8.2	-0.2	-6.3;-6.0	0.96
RPE (μm)	13.8 **±**1.5	14.0 **±**1.3	-0.2	-1.2;-0.8	0.68

*MC* Mutation carrier, *SD* standard deviation, *CI* confidence interval, *TMT* total macular thickness, *mRNFL* macular retinal nerve fiber layer, *GCL* ganglion cell layer, *IPL* inner plexiform layer, *INL* inner nuclear layer, *OPL* outer plexiform layer, *ONL* outer nuclear layer; *RPE* retinal pigment epithelium. * p <0.05 tested by GEE

### P76 Life traumatic experiences and stressful events as predictors of detoxification-therapy outcome at 6 months in chronic migraine with medication overuse

#### Bottiroli Sara^1^, Sances Grazia^1^, De Icco Roberto^1,2^, Vito Bitetto^1, 2^, Guaschino Elena^1^, Marta Allena^1^, Pazzi Stefania^1^, Giuseppe Nappi^1^, Tassorelli Cristina^1,2^

##### ^1^Headache Science Center, Mondino Foundation, Pavia, Italy; ^2^Department of Brain and Behavioral Sciences, University of Pavia, Italy

###### **Correspondence:** Bottiroli Sara (sara.bottiroli@mondino.it)

**Objectives**. Withdrawal from overused drug is the treatment of choice for MOH, reverting the headache from chronic to episodic. Many factors are involved in MOH prognosis and outcome, becoming is a topic of interest. In this study we evaluated the association between early life traumatic experiences and recent stressful events with the outcome following detoxification therapy in a 6-month follow-up in 164 subjects with medication overuse headache (MOH).

**Methods.** This study was conducted at the Mondino Foundation in Pavia, Italy. All consecutive patients with MOH undergoing an inpatient detoxification program were enrolled and followed-up in a prospective study. Diagnosis was operationally defined according to ICHD-IIIβ. The study was approved by the Ethics Committee of San Raffaele Scientific Institute (Pavia, Italy) and written informed consent was obtained from all patients. The protocol consisted in an inpatients detoxification treatment and a 6-month follow-up. Data on early life traumatic experiences – distinguished in term of physical and emotional traumas – and recent stressful events were collected by means of self-report questionnaires. Data were analyzed with univariate and multivariate logistic regressions.

**Results.** Of the 164 patients who completed the 6-month follow-up, 111 (54%) stopped overuse and their headache reverted to an episodic pattern, whereas 53 (32%) had a negative outcome given that they stopped overuse without any benefit on headache frequency or failed to stop overuse. At the univariate analysis the following variables resulted associated to the negative outcome: having experienced emotional traumas (OR 3.409; p = 0.002), having had both traumas and stressful events (OR 12.429; p < 0.001), presence of mood disorders (OR 2.373; p = 0.014), higher MIDAS scores (OR 1.015; p < 0.001), higher number of days with medication intake (OR 2.373; p = 0.014) and higher number of days with headache (OR 1.193; p = 0.002). At the multivariate analyses, having experienced both childhood traumas and recent stressful events (OR 14.229; p = 0.002) together with higher MIDAS scores (OR 1.026; p = 0.004), and presence of mood disorders (OR 8.527; p = 0.009) were prognostic for the negative outcome.

**Conclusions.** Our data suggest the synergetic impact of both childhood traumas and recent stressful events, together with other psychological variables, in determining a negative outcome after detoxification in MOH. These findings have important practical implications on how to treat these patients.

**Acknowledgements**

This study was supported by a grant of the Italian Ministry of Health to C. Mondino Foundation (RC 2014-2016).

### P77 Role of transient receptor potential (TRP) channels in a rat model of trigeminal neuropathic pain: focus on TRPA1 channels

#### Chiara Demartini^1^, Rosaria Greco^1^, Anna Maria Zanaboni^1,2^, Oscar Francesconi^3^, Cristina Nativi^3^, Cristina Tassorelli^1,2^, Kristof Deseure^4^

##### ^1^Laboratory of Neurophysiology of Integrative Autonomic Systems, Headache Science Center, IRCCS Mondino Foundation, Pavia, Italy; ^2^Department of Brain and Behavioral Sciences, University of Pavia, Pavia, Italia; ^3^Department of Chemistry ‘Ugo Schiff’, University of Florence, Florence, Italy; ^4^Department of Medicine, Laboratory for Pain Research, University of Antwerp, Wilrijk, Belgium

###### **Correspondence:** Chiara Demartini (chiara.demartini@mondino.it)

The transient receptor potential ankyrin type-1 (TRPA1) channels are known to actively participate in different pain conditions including trigeminal neuropathic pain, whose clinical treatment is still unsatisfactory. The aim of this study was to evaluate the involvement of TRPA1 channels, by means of the antagonist ADM_12, in trigeminal neuropathic pain in order to identify possible therapeutic targets. Single treatment of ADM_12 in rats 4 weeks after the chronic constriction injury of the infraorbital nerve (IoN-CCI) significantly reduced the mechanical allodynia induced in the IoN-CCI rats. Additionally, ADM_12 was able to abolish the increased levels of TRPA1, calcitonin gene-related peptide (CGRP) and substance P (SP) gene expression in trigeminal ganglia, cervical spinal cord and medulla induced in the IoN-CCI rats. By contrast no significant differences between groups were seen as regards CGRP and SP protein expression in the nucleus trigeminalis caudalis. ADM_12 also reduced TRP vanilloid type-1 (TRPV1) gene expression in the same areas after IoN-CCI. These findings show the involvement of both TRPA1 and TRPV1 channels in trigeminal neuropathic pain and, more specifically, their role in trigeminal mechanical allodynia; moreover they suggest ADM_12 as a possible tool in trigeminal neuropathic pain management.

**Ethics approval**

The study was approved by Institution‘s Ethics Board (University of Antwerp, Belgium), approval number 2017-16.

### P78 Meningeal contribution to migraine pain: a 3T magnetic resonance angiography study

#### Sabrina Khan^1^, Faisal Mohammad Amin^1^, Casper Emil Christensen^1^, Hashmat Ghanizada^1^, Samaira Younis^1^, Anne Christine Rye Andersen^1^, Patrick J H de Koning^2^, Henrik B W Larsson^3^, Messoud Ashina^1^

##### ^1^ Danish Headache Center and Department of Neurology, Rigshospitalet Glostrup, Faculty of Health and Medical Sciences, University of Copenhagen, Denmark; ^2^ Division of Image Processing, Department of Radiology, Leiden University Medical Center, Leiden, Netherlands; ^3^ Functional Imaging Unit, Department of Clinical Physiology, Nuclear Medicine and PET, Rigshospitalet, Faculty of Health and Medical Sciences, University of Copenhagen, Denmark

###### **Correspondence:** Sabrina Khan (sksabrinakhan@gmail.com)

The origin of migraine pain is unknown but possibly implicates the dura mater, which is pain sensitive in proximity to the meningeal arteries. Therefore, subtle changes in vessel caliber on the head pain side could reflect activation of dural perivascular nociceptors that leads to migraine headache. To test this hypothesis, we measured circumference changes of cranial arteries in patients with cilostazol-induced unilateral migraine without aura using 3T high-resolution magnetic resonance angiography (MRA). The middle meningeal artery (MMA) was of key interest, as it is the main supply of the dura mater. We also measured the superficial temporal (STA) and external carotid (ECA) arteries as additional extracranial segments, and the middle cerebral (MCA), the cerebral and cavernous parts of the internal carotid (ICA_cerebral_ and ICA_cavernous_), and the basilar (BA) arteries as intracranial arterial segments. MRA scans were performed at baseline, migraine onset, after sumatriptan, and ≥27 hours after migraine onset.A total of 30 patients underwent MRA scans, of which 26 patients developed unilateral attacks of migraine without aura and were included in the final analysis. Eleven patients treated their migraine with sumatriptan while the remaining 15 patients did not treat their attacks with analgesics or triptans.

At migraine onset, only MMA exhibited greater circumference increase on the pain side (0.24 ± 0.37 mm) compared to the non-pain side (0.06 ± 0.38 mm) (p=0.002). None of the remaining arteries revealed any pain-side specific changes in circumference (p>0.05), but exhibited bilateral dilation. Sumatriptan constricted all extra-cerebral arteries (p<0.05). In the late phase of migraine, we found sustained bilateral dilation of MMA.

In conclusion, onset of migraine is associated with increase in MMA circumference specific to the head pain side. Our findings suggest that vasodilation of MMA may be a surrogate marker for activation of dural perivascular nociceptors, indicating a meningeal site of migraine headache.

### P79 Sex differences in vascular responses to CGRP

#### Alejandro Labastida-Ramírez^1^, Eloísa Rubio-Beltrán^1^, A. van den Bogaerdt^2^, J. Schouten^3^, A.H. Jan Danser^1^, Carlos M. Villalón^4^, Antoinette MaassenVanDenBrink^1^

##### ^1^Division of Vascular Medicine and Pharmacology, Dept. of Internal Medicine, ^2^Heart Valve Bank Rotterdam, ^3^Deparment of Neurosurgery, Erasmus University Medical Center, P.O. Box 2040, 3000 CA Rotterdam, The Netherlands; ^4^Deparment of Pharmacobiology, Cinvestav-Coapa, Mexico City, Mexico

###### **Correspondence:** Alejandro Labastida-Ramírez (a.labastidaramirez@erasmusmc.nl)

**Background and aim:** Migraine is two to three times more prevalent in women than in men. The mechanisms underlying this high prevalence seem to be related with a cross-talk between (fluctuations of) ovarian steroid hormones and the CGRPergic system. The present study investigated the vasodilatory effects of CGRP in human isolated coronary and middle meningeal arteries of women in the pre- and postmenopausal age range and compared it to men of the same age category.

**Methods:** Human coronary arteries were obtained from “beating hearts” organ donors who died of non-cardiac disorders (females: n=19 <50 years, n=18 >50 years; males: n=15 <50 years, n=17 >50 years) and human middle meningeal arteries were obtained from dura mater of patients who underwent neurosurgery (females: n=8 <50 years, n=10 >50 years; males: n=7 <50 years, n=3 >50 years). In both arteries, concentration‑response curves to CGRP were constructed to obtain the pEC_50_ and maximum contractile response (E_max_). Since we were not able to obtain the pre- or postmenopausal status of the women included because of ethical restrictions, we classified women into groups of below and above 50 years of age, approximately corresponding to pre- and postmenopausal women. Men were divided into the same age categories.

**Results:** In human isolated coronary arteries, CGRP induced concentration-dependent dilations that were not different between men and women, either in the younger (<50 years) and older (>50 years) age categories. In contrast, in middle meningeal arteries CGRP had a significantly (p=0.01) lower maximal response (but similar pEC_50_) in young (<50 years) women (E_max_ 61±9 %) when compared with men of the same age category (E_max_ 92±5), while there was no difference between responses to CGRP in arteries obtained from older women and men.

**Conclusion:** Relaxant responses to CGRP were diminished in dural arteries of women <50 years, as compared to those in dural arteries obtained in men from the same age group. Our results suggest that (fluctuations in) ovarian sex steroid hormones could desensitize the dural CGRP receptor, either directly, or via increased dural CGRP release. Further experiments are needed to determine the causal relationship between lower responses to CGRP and cycling steroid hormones in premenopausal women.

### P80 Effect of onabotulinumtoxinA prevention on comorbidities of depression and anxiety in chronic migraine: Analysis in headache day frequency responders vs headache day frequency non-responders

#### Andrew M. Blumenfeld^1^ Stewart J. Tepper^2^ Lawrence D. Robbins^3^ Amelia Orejudos^4^ Aubrey Manack Adams^4^ Dawn C. Buse^5^ Stephen D. Silberstein^6^

##### ^1^Headache Center of Southern California, The Neurology Center, Carlsbad, CA, 92024, USA; ^2^ Neurology Department, Headache Center, Dartmouth-Hitchcock Medical Center, Lebanon, NH, 03748, USA; ^3^Robbins Headache Clinic, Riverwoods, IL, 60015, USA; ^4^Allergan plc, Irvine, CA, 92612, USA; ^5^Department of Neurology, Albert Einstein College of Medicine, Bronx, NY, 10461, USA; ^6^Department of Neurology, Jefferson Headache Center, Philadelphia, PA, 19107, USA

###### **Correspondence:** Andrew M. Blumenfeld (blumenfeld@neurocenter.com)

**Background**: Chronic migraine (CM) is comorbid with anxiety and depression. This analysis of COMPEL Study data assessed the relationship between use of onabotulinumtoxinA and depression and anxiety in people with CM who had that comorbidity and also had a ≥25% reduction in headache day frequency at week 24.

**Methods**: The 108-week, multicenter, open-label COMPEL Study (ClinicalTrials.gov, NCT01516892) enrolled adults with CM receiving onabotulinumtoxinA 155 U. Changes in depression (Patient Health Questionnaire [PHQ-9]) and anxiety (Generalized Anxiety Disorder [GAD-7]) sum scores in those with clinically significant depression (PHQ-9 ≥5) and anxiety (GAD-7 ≥10) at baseline were analyzed in those with a ≥25% reduction in headache day frequency at week 24 (i.e., “headache day reduction responders”) vs those who did not (“non-responders”). A ≥1 severity category improvement in the PHQ-9 and/or GAD-7 was considered clinically meaningful. The study received ethical approval from the Institutional Review Board at each site.

**Results:** Patients (N=715) had a mean (range) age of 43 (18–73) years, were primarily women (84.8%; 606/715), and had on average mild or worse depressive symptoms (PHQ-9 ≥5; 74.5% [529/710]) or moderate or worse anxiety (GAD-7 ≥10; 24.6% [175/711]). Mean (SD) headache day frequency at week 108 significantly decreased from baseline: 22 (4.8) to 11.3 (7.4) days (*P*<0.0001). Depressive symptoms significantly (*P*<0.001) improved in people with mild or worse depression regardless of 25% headache day reduction response (Fig. 1A), as did anxiety symptoms in those with moderate or worse anxiety (*P*<0.001, Fig. 1B); 79.8% of headache day frequency responders and 53.2% of non-responders experienced a reduction of ≥1 severity category on the PHQ-9 (Fig. 1C). 82.2% and 70.4%, respectively, experienced a reduction of ≥1 severity category on the GAD-7 (Fig. 1D).

**Conclusions**: COMPEL study results demonstrate that onabotulinumtoxinA use is associated with a reduction in symptoms of depression and anxiety among people with CM, regardless of whether onabotulinumtoxinA treatment resulted in a ≥25% reduction in headache day frequency, although the change is less robust in the <25% responder group.

**Fig. 1 (abstract P80).**
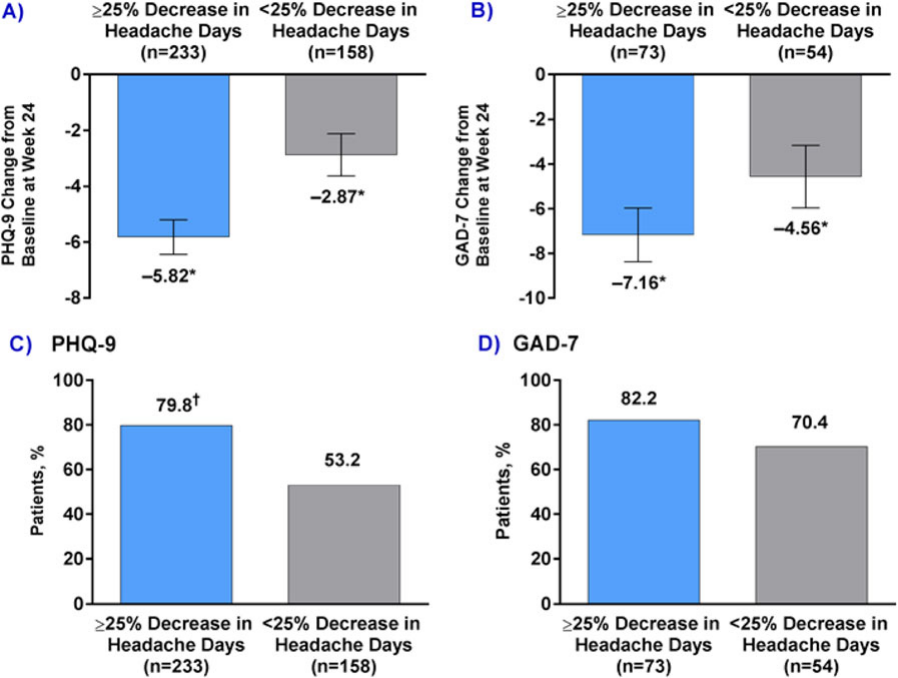
Change from baseline in (A) PHQ-9 total scores and (B) GAD-7 total scores; proportion of patients experiencing ^3^1 reduction in severity category for (C) PHQ-9 and (D) GAD-7 after treatment with onabotulinumtoxinA. GAD-7=7-item Generalized Anxiety Disorder Assessment; PHQ-9=9-item Patient Health Questionnaire. *Indicates P<0.0001 versus baseline. †Indicates P<0.001 for between group comparison

### P81 Life with migraine, effect on relationships, career and finances, and overall health and well-being: Results of the Chronic Migraine Epidemiology and Outcomes (CaMEO) study

#### Dawn C. Buse^1^, Sharron Murray^2^, Paula K. Dumas^3^, Aubrey Manack Adams^4^, Michael L. Reed^5^, Kristina M. Fanning^5^, Richard B. Lipton^1^

##### ^1^Department of Neurology, Albert Einstein College of Medicine, Bronx, NY, 10461, USA; ^2^Wenatchee, WA, 98801, USA; ^3^Migraine Again LLC, Alpharetta, GA, 30022, USA; ^4^Allergan plc, Irvine, CA, 92612, USA; ^5^Vedanta Research, Chapel Hill, NC, 27517, USA

###### **Correspodence:** Dawn C. Buse (dbuse@montefiore.org)

**Background:** Migraine can negatively affect many aspects of an individual’s life as well as the lives of people close to them. This analysis of CaMEO Study data evaluated and compared the effects of episodic (EM) and chronic migraine (CM) across a range of domains.

**Methods:** The CaMEO Study (ClinicalTrials.gov, NCT01648530) is a prospective, longitudinal, web-based survey study designed to characterize migraine impact, among other objectives, in a systematic US sample of people meeting modified *ICHD-3* criteria: 19,891 respondents meeting said criteria were invited to complete the Family Burden Module. Answers by migraine respondents (probands) relating to the impact of migraine on family life, career and finances, and overall health—including how life would be without migraine—were recast as dichotomous variables for analysis. Descriptive analysis of items stratified by EM (<14 headache days/month) and CM (≥15 headache days/month) were reported. Comparative analyses utilized Chi-square and *P* values to indicate significant differences. The study received ethical approval from the Institutional Review Board of the Albert Einstein College of Medicine.

**Results:** 13,064 respondents (EM: 11,938 [91.4%]; CM: 1,126 [8.6%]) provided valid data. Approximately 20% of respondents not currently in a relationship (n=3,512) or in a relationship but not living together (n=1,393) indicated that headaches had contributed to relationship problems. Of those in a relationship and living together (n=8,154), 49.0% agreed somewhat/completely that they would be a better partner if they did not have headaches (EM: 46.2%; CM: 78.2%; *P*<0.001); 3.2% had delayed having children/had fewer children (EM: 2.6%; CM: 9.6%; *P*<0.001). Of 13,061 individuals responding to career items, 32.7% indicated that headaches had affected ≥1 item (EM: 30.0%; CM: 58.4%). Overall, 28.9% of respondents worried about covering household expenses (EM: 26.7%; CM: 52.9%), and 32.1% about long-term financial security (EM: 29.7%; CM: 57.4%). 16.4% reported poor or fair overall health (EM: 14.2%; CM: 40.5%). Across 9 “life-with-migraine” items, 69.6% of EM respondents (CM: 87.7%) reported ≥1 area that would be “better/a lot better” if they did not have headaches.

**Conclusions:** Migraine can negatively affect many aspects of life including relationships, career and financial outcomes, and overall health. People with migraine, particularly CM, feel that headaches affect many important areas of life and perceive that life would be better/a lot better without headache. Physicians managing migraine should consider the overall burden of disease.

### P82 A multicenter, prospective, randomized, open-label study to compare onabotulinumtoxinA and topiramate for headache prevention in adults with chronic migraine: patient reported functional outcomes from the FORWARD study

#### Andrew M. Blumenfeld^1^, Atul T. Patel^2^, Aubrey Manack Adams^3^, John F. Rothrock^4^

##### ^1^Headache Center of Southern California, The Neurology Center, Carlsbad, CA, 92024, USA; ^2^Department of Rehabilitation Medicine, Kansas City Bone and Joint Clinic, Overland Park, KS, 66211, USA; ^3^Allergan plc, Irvine, CA, 92612, USA; ^4^George Washington School of Medicine, Washington, DC, 20037, USA

###### **Correspondence:** Andrew M. Blumenfeld (blumenfeld@neurocenter.com)

**Background:** Patient-reported outcomes (PROs) can be important indicators of a migraine therapy’s real world effectiveness. Safety, tolerability, and PROs of onabotulinumtoxinA (onabotA) vs topiramate (TPM) for CM are presented.

**Methods**: FORWARD, a multicenter, randomized study (ClinicalTrials.gov, NCT02191579), compared onabotA 155U every 12 wks for 3 cycles with TPM 50-100 mg/day for up to 36 wks. The primary efficacy measure was proportion of patients with ≥50% reduction in headache (HA) days/mo vs baseline at wk 32. Adverse events (AEs) were recorded. PRO measures included: Controlled Oral Word Association Test (COWAT); 9-Item Patient Health questionnaire (PHQ-9); and the Work Productivity and Activity Impairment: Specific Health Problem (WPAI-SHP) questionnaire. The protocol was approved by the Institutional Review Board at each site.

**Results**: 282 patients enrolled (onabotA, n=140; **TPM**, n=142); 148 completed treatment (onabotA, 85.7%; **TPM**, 19.7%). Primary reasons for withdrawal were lack of efficacy (onabotA, 5.0%; **TPM**, 19.0%) and AEs (onabotA, 3.6%; **TPM**, 50.7%). Using baseline observation carried forward (BLOCF) imputation, more patients on onabotA had ≥50% reduction in HA frequency vs **TPM** (40.0% vs 12.0%; *P*<0.001). Treatment-related AEs (TRAEs) were reported by 17.3% of onabotA and 69.0% of **TPM** patients. **TPM** was associated with a reduction in mean [SD] COWAT scores at wk 12 (−2.8 [5.7]), suggesting early cognitive changes in **TPM** recipients. The effect of **TPM** was potentially obscured by BLOCF methodology due to the large proportion of **TPM** withdrawals. OnabotA resulted in a small increase in mean [SD] COWAT scores at wks 12 (1.2 [8.1]) and 36 (2.8 [.8]). At wk 36, onabotA had a significantly greater effect on mean [SD] PHQ-9 scores vs **TPM** (4.4 [4.2] vs 7.1 [5.8]; estimated mean difference: –1.86 [*P*<0.001]). Both treatments were associated with a slight reduction in WPAI-SHP Absenteeism and Presenteeism scores at week 36; onabotA significantly reduced mean Work Productivity Loss scores vs TPM.

**Conclusions**: Based on TRAEs and discontinuation rates, onabotA was associated with significantly better tolerability than TPM. PRO data suggest that changes in cognition may be seen as early as wk 12 in TPM recipients. OnabotA had a more favorable effect on depressive symptoms and improved work productivity loss and impairment in patients with CM vs TPM.

### P83 Headache attributed to psychiatric disorder

#### Ana Lj Podgorac^1^, Goran Gajić ^1^, Bojana Pejušković ^1,2^, Ljubica Zamurović-Dunđerović ^1^, Vanja B Mandić-Maravić ^1^, Aleksandar Misojčić ^1^, Dušica M Lečić-Toševski ^1,2,3^

##### ^1^ Institute of Mental Health, 37 Palmoticeva St, Belgrade, Serbia; ^2^ School of Medicine University of Belgrade, Belgrade, Serbia; ^3^ Serbian Academy of Sciences and Arts Belgrade, Serbia

###### **Correspondence:** Ana Lj Podgorac (anasundic@gmail.com)

**Introduction**: Contrary to numerous studies showing a high degree of comorbidity between psychitric disorders and primary headache disorders, pointing to psychiatric disorder as a risk factor for headache progression and chronification, the number of studies that put in the spotlight headache occurring only during the psychiatric disturbance, e.g. “headache attributed to psychiatric disorder” [1,2], is significantly smaller. Literature data, limitted to case reports and few retrospective studies, point out headache attributed to psychiatric disorders as an uncommon headache syndrome with wide area of clinical presentation, differential diagnosis, clinical implications and needs for future research [2,3].

**Methods**: Hereby, we present the sample of psychiatric patients, treated for a period of one year, from January to December 2016, at the Clinical Department for Psychotic Disorders of the Institute of Mental Health, who suffered from headache. The psychiatric disorder has been diagnosed according to diagnostic criteria given in the fifth edition of the Diagnostic and Statistical Manual of Mental Disorders (DSM-5) [4],, while the headache has been diagnosed according to diagnostic criteria given in the third edition of The International Classification of Headache Disorders (ICHD-3) [2],.

**Results:** Out of 427 patients that had been treated in this period, 25 of them had headache. Majority of patients with headache (19) had major depressive disorder , recurrent in 15 patients. One patient had bipolar affective disorder. Five patients had psychotic disorder, four of them schizophrenia, and one patient presented with acute polymorphic psychotic disorder with symptoms of schizophrenia. Primary headache was present in 19 patients (migraine without aura – 6, migraine with aura – 2, chronic migraine – 2, episodic tension type headache – 3, chronic tension type headache -5). One patient had medication overuse headache. The diagnosis of headache attributed to depressive disorder was established in three patients. One patient had headache attributed to somatization disorder, and one patient had headache attributed to psychotic disorder.

**Discussion:** This observation confirmes well-known high comorbidity of psychiatric disorders and primary headache disorders, especially the chronic forms and supports the current headache classification in which the headache attributed to somatization disorder and headache attributed to psychotic disorder had been recognized. As well, by this report authors support the opinion of other authors who suggested that the headache attributed to depressive disorder, as the most common type of headache attributed to psychiatric disorders, corresponding to the additional codes in the appendix of the classification (code 12.3) could be added to the classification itself.

**Key words**: headache, psychiatric disorder

**References**:

1. Smitherman TA, Baskin SM. Headache Secondary to Psychiatric Disorders. Current Pain and Headache Reports 2008; 12: 305 – 310.

2. Headache Classification Committee of the International Headache Society (IHS). The International Classification of Headache Disorders, 3rd edition (beta version). Cephalalgia 2013; 33(9): 629–808.

3. Radat F, Milowska D, Valade D. Headaches Secondary to Psychiatric Disorders (HSPD):A Retrospective Study of 87 Patients. Headache 2011;51:789-795.

4. American Psychiatric Association. (2013). *Diagnostic and statistical manual of mental disorders* (5th ed.). Arlington, VA: American Psychiatric Publishing.

### P84 Healthcare expenditure: does society give migraine the focus and recognition patients deserve?

#### Leonhard Schaetz^1^, Parth Joshi^2^, Aoife Callan^3^, Vivek Khurana^2^, Jasper Huels^1^

##### ^1^Global Patient Access, Novartis Pharma AG, Basel, CH-4002, Switzerland; ^2^Product Life Cycle Services, NBS, Novartis Healthcare Private Limited, Hyderabad, 500081, India; ^3^Novartis Global Services Centre, Patient Access Services, Dublin, D04 A9N6, Ireland

###### **Correspondence:** Leonhard Schaetz (Leonhard.schaetz@novartis.com)

**Background**

Migraine is a painful neurological disease that is under-recognized, under-diagnosed, and under-treated worldwide [1]. Indeed the scale of the under-treatment of headache disorders prompted the World Health Organization (WHO) in 2011 to write to the world’s Ministries of Health to “illuminate the worldwide neglect of a major public-health problem, and reveal the inadequacies of responses to it” [1, 2]. To understand investment in managing migraine, we analyzed and compared healthcare expenditure on diseases with high disability, including migraine, across four countries: the United States of America (USA), Canada, Germany, and the Netherlands.

**Methods**

Diseases associated with high disability, as measured by Years Lived with Disability (YLDs), were identified from the Global Burden of Disease 2016 study for each country [3]. The top 10 diseases with high disability where health expenditure data were also available, were included in this analysis. Direct annual healthcare expenditure data per disease was sourced from a published study for the USA and government websites for Canada, Germany, and the Netherlands [4-7]. All the cost estimates were adjusted to year 2016 using healthcare specific inflation rates and were converted to US dollars using purchase power parity conversion.

**Results**

Five diseases identified as high disability burden in all four countries, included osteoarthritis, diabetes mellitus, skin and subcutaneous diseases, anxiety disorders, and migraine. Of these, and despite high disability burden, health expenditure on migraine was consistently the lowest across all countries and constituted less than 0.5% of total direct annual healthcare expenditure. Comparatively, annual healthcare expenditure on osteoarthritis was 7-times as that of migraine in the USA, 4-times in Canada, 19-times in Germany, and 24-times in the Netherlands, though the disability associated with it was much lower than migraine (Fig. 1).

**Discussion and conclusion**

The study highlights the significant underinvestment in migraine despite its high burden and relative to other high disability diseases. Such underinvestment may be correlated with major gaps in migraine management including low diagnosis rates, delays and barriers in access to specialists, and under-treatment thus contributing to the overall burden of disease on individuals and society. Literature suggests that better-quality care leads to improved outcomes for patients with migraine [8-10]. Further research is needed to expand this analysis to other countries and to seek possible solutions on how improved care can be made accessible to broader patient populations.

**Funding**

This study was funded by Novartis AG, Basel, Switzerland.

**References**

1. Steiner TJ et al. Time to act on headache disorders. The Journal of Headache and Pain. 2011; 12: 501-3

2. Steiner TJ et al. Migraine is first cause of disability in under 50s: will health politicians now take notice? The Journal of Headache and Pain. 2018; 19: 17

3. Vos T et al. Global, regional, and national incidence, prevalence, and years lived with disability for 328 diseases and injuries for 195 countries, 1990–2016: a systematic analysis for the Global Burden of Disease Study 2016. The Lancet. 2017; 390: 1211-59

4. Dieleman et al. US Spending on Personal Health Care and Public Health, 1996-2013. JAMA. 2016; 316: 2627-46

5. EBIC. Economic Burden of Illness in Canada online tool 2008. Accessed on 17 May 2018. [Available from: http://cost-illness.canada.ca/index.php

6. GBE-BUND. Cost of illness in millions of Euro for Germany 2015. Accessed on 17 May 2018. [Available from: http://www.gbe-bund.de/gbe10/trecherche.prc_them_rech?tk=19200&tk2=19400&p_uid=gast&p_aid=64133544&p_sprache=E&cnt_ut=3&ut=19460

7. RIVM. Cost of Illness in the Netherlands 2015. Accessed on 17 May 2018. [Available from: https://statline.rivm.nl/#/RIVM/nl/dataset/50040NED/table?graphtype=Table&ts=1512975518824

8. Katsarava et al. Poor medical care for people with migraine in Europe – evidence from the Eurolight study. The Journal of Headache and Pain. 2018; 19: 10

9. Soon et al. Assessment of migraineurs referred to a specialist headache clinic in Singapore: diagnosis, treatment strategies, outcomes, knowledge of migraine treatments and satisfaction. Cephalalgia. 2005; 25: 1122–32

10. Hu et al. Survey of migraineurs referred to headache specialists: care, satisfaction, and outcomes. Neurology. 2000; 55: 141–143

**Fig. 1 (abstract P84).**
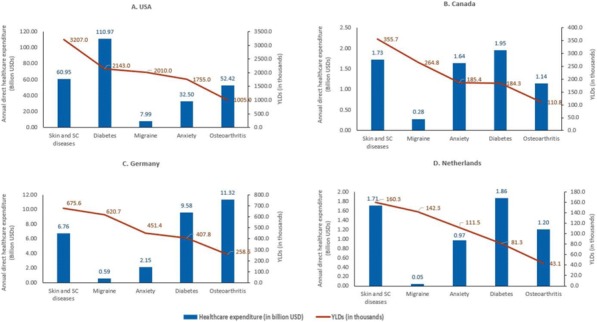
Annual direct healthcare expenditure for causes with high disability in the USA, Canada, Germany, and the Netherlands (Comparisons were made for 10 causes of high disability in each country. Data for the top five high disability diseases that were common across all four countries are presented). Abbreviations: Anxiety: Anxiety disorders; Diabetes: Diabetes mellitus; Skin and SC diseases: Skin and subcutaneous diseases; USA: United States of America; USDs: United States dollars; YLDs: Years lived with disability.

### P85 Gender differences in clinical and pharmacological response to triptans

#### Daphne S. van Casteren^1,2^, Tobias Kurth^3^, Michel D. Ferrari^2^, Gisela M. Terwindt^2,#^, Antoinette Maassen van den Brink^1,#^

##### ^1^Deparment Of Internal Medicine, Div. of Pharmacology, Erasmus Medical Center, P.O. Box 2040, 3000 CA Rotterdam, the Netherlands; ^2^Deparment Of Neurology, Leiden University Medical Center, P.O. Box 9600, 2300 RC Leiden, the Netherlands; ^3^Institute of Public Health, Charité – Universitätsmedizin Berlin, Berlin, Germany.

###### **Correspondence:** Daphne S. van Casteren (D.S.van_Casteren@lumc.nl)

^*#*^ *Shared last author*

**Objective**

To examine the effect of gender on clinical response outcomes to triptans in migraine patients and to relate these gender differences to pharmacokinetic parameters of triptans in men and women.

**Methods**

In a systematic literature search of PubMed, MEDLINE, the **Cochrane Library**, Embase and Web of Science, we identified clinical trials distinguishing clinical response to or pharmacokinetic parameters of triptans between men and women. Male-to-female pooled risk ratios (RR) for each clinical outcome were calculated using random-effects models. For the investigation of gender differences in pharmacokinetic outcomes meta-analyses were conducted using pooled ratio of means (RoM) random-effects models.

**Results**

Over 1000 publications were found by searching for publications on clinical trials with triptans, of which 190 remained after adding sex and gender related search terms. Only in 19 publications data were presented in such a way that they could be included in the meta-analysis. No gender differences were revealed for headache response after 2 hours (9 studies, male-to-female RR 1.03, 95% CI: 0.99-1.08) and pain free response after 2 hours (6 studies, male-to-female RR 1.02, 95% CI: 0.98-1.07). In contrast, females had a higher risk for headache recurrence within 24 hours or 48 hours (4 studies, male-to-female RR 0.74, 95% CI: 0.56-0.97) and a higher adverse event (AE) frequency than males (6 studies, male-to-female RR 0.82, 95% CI: 0.76-0.89).Results for Area Under the plasma drug concentration-time Curve from time zero to infinite time (AUC _0-∞_, ng/h/ml) and peak drug concentration (C_max_, ng/ml) were found only for frovatriptan, zolmitriptan and rizatriptan. Women appeared to have a higher AUC_0-∞_ (16 studies, RoM 0.64, 95% CI: 0.57-0.72) and C_max_ (18 studies, RoM 0.70, 95% CI: 0.63-0.77) than men. No statistically significant gender differences were revealed on plasma half-life Times (T_1/2_) of frovatriptan (2 studies, RoM 0.90, CI: 0.70-1.14) and zolmitriptan (8 studies, RoM 0.93, CI: 0.85-1.01).

**Conclusions**

Given the widespread use of triptans and the large amount of literature on this topic, there are remarkably few publications about gender differences in response to triptans. Based on the limited data available, we conclude that the AUC_0-∞_ and C_max_ are higher in women than in men for frovatriptan, zolmitriptan and rizatriptan, which may be an explanation for the higher AE frequency in women. This higher exposure in women (when extrapolated to the other triptans) is, however, not reflected by higher response rates to the triptans in women. Moreover, the headache recurrence rate is higher in women than in men, pointing to a potential discrepancy with the pharmacokinetic data. We hypothesise that the higher recurrence rate in women, despite their higher drug exposure, may be assigned to more persistent migraine attacks that are triggered by sex hormonal changes, such as menstrually-related migraine attacks and possibly attacks during perimenopause.

**Fig. 1 (abstract P85).**
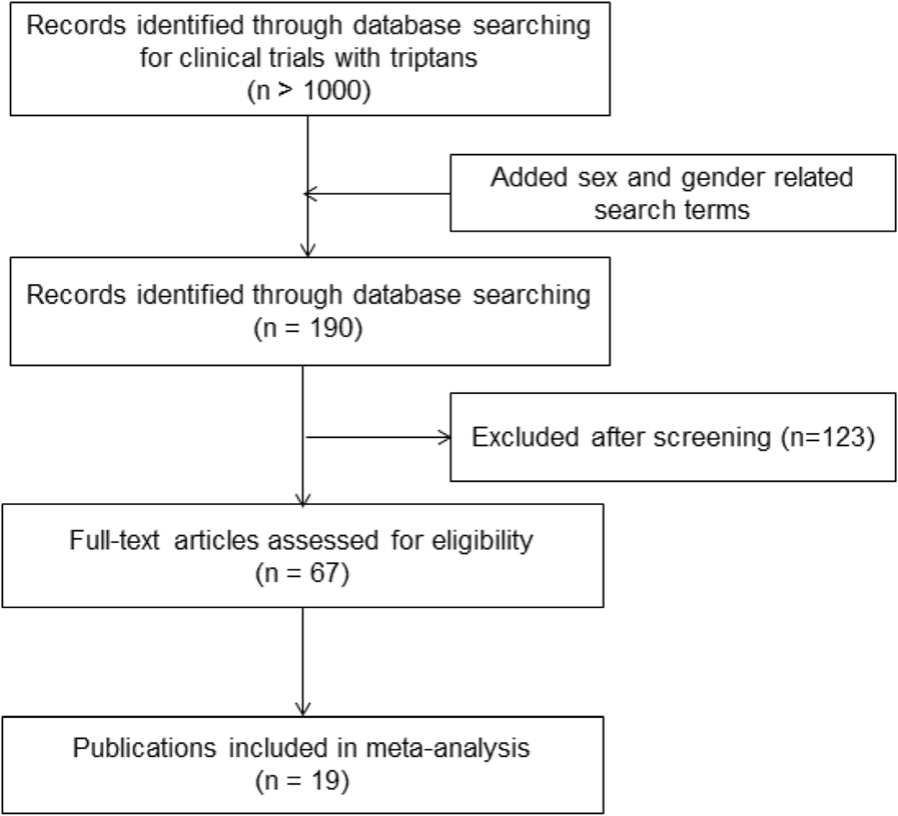
Flowchart of publication selection process.

**Fig. 2 (abstract P85).**
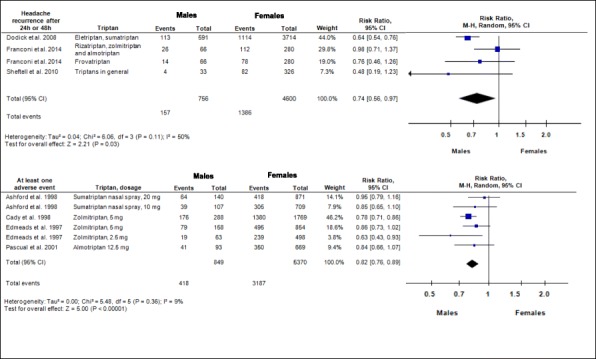
Forest plots of headache recurrence rate after 24 hours or 48 hours and the incidence of at least one adverse event after the intake of a triptan in male and female migraine patients.

**Fig. 3 (abstract P85).**
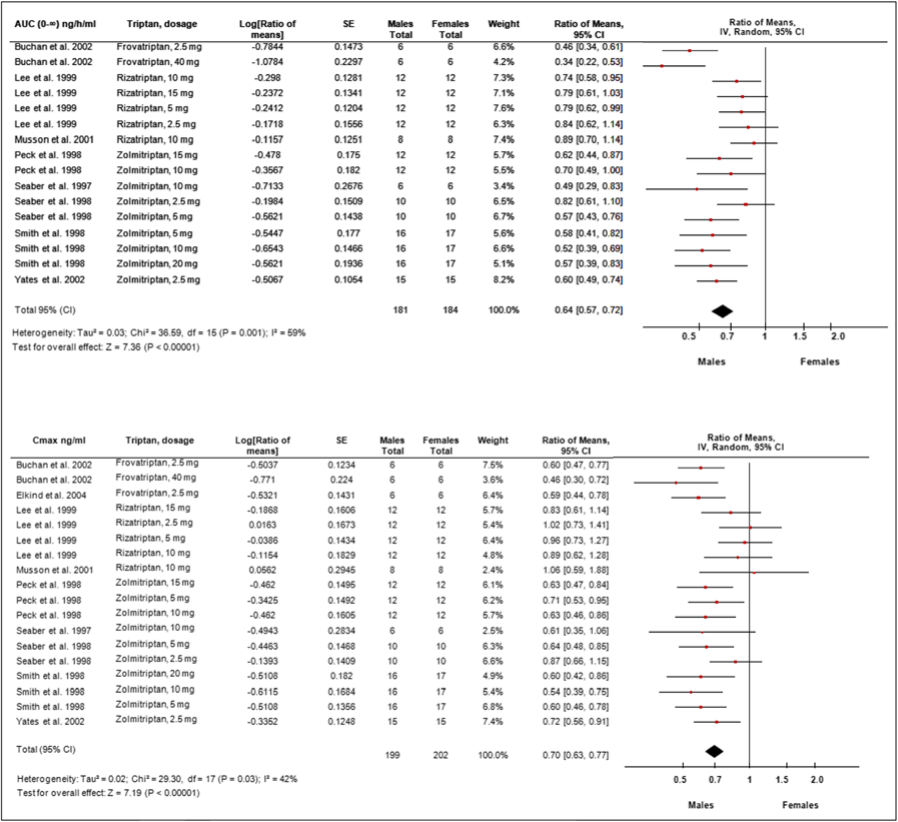
Forest plots of AUC0-∞ and Cmax after the intake of frovatriptan, rizatriptan or zolmitriptan in male and female migraine patients.

### P86 Migraine induction with calcitonin gene-related peptide in patients from erenumab trials

#### Casper Emil Christensen^&^; Samaira Younis^&^; Marie Deen; Sabrina Khan; Hashmat Ghanizada; Messoud Ashina

##### Danish Headache Center and Department of Neurology, Rigshospitalet Glostrup, Faculty of Health and Medical Sciences, University of Copenhagen, Copenhagen, Denmark

###### **Correspondence:** Messoud Ashina (ashina@dadlnet.dk)

^**&**^ These authors contributed equally to this work.

**Background:** Erenumab has recently been approved by the US Food and Drug Administration as a monoclonal antibody against the calcitonin gene-related peptide (CGRP) receptor for migraine-specific preventive treatment. Identifying those patients with the greatest potential to benefit from erenumab treatment could have a major impact on clinical practice. CGRP provokes migraine attacks and the question is whether hypersensitivity to CGRP infusion might be a predictor of erenumab efficacy, serving as a biomarker of treatment efficiency.

**Objective:** To explore a possible correlation between individual efficacy of anti-CGRP treatment and susceptibility to migraine induction by CGRP.

**Methods:** Thirteen migraine patients, previously enrolled in erenumab anti-CGRP receptor monoclonal antibody trials, received CGRP in a double-blind, placebo-controlled, randomized design to investigate their susceptibility to migraine induction. A standardized questionnaire was used to assess efficacy of antibody treatment. The patients were stratified into groups of high responders and poor responders.

**Results:** Ten high responders and three poor responders were included. CGRP induced migraine-like attacks in ten (77%) patients, whereof two were poor responders, compared to none after placebo (p=0.002). The area under the curve for headache intensity was greater after CGRP, compared to placebo, at 0–90 min (p=0.009), and 2–12 h (p=0.014). The median peak headache intensity score was 5 (5–9) after CGRP, compared to 2 (0–4) after placebo (p=0.004).

**Conclusions:** Patients with an excellent effect of erenumab are highly susceptible to CGRP provocation. A large-scale prospective CGRP provocation study in patients should confirm whether hypersensitivity to CGRP could be a biomarker for predicting antibody treatment efficacy.

**Trials Registration:** ClinicalTrials.gov identifier: NCT03481400.

**Ethics approval and consent to participate**

The study was approved by the Ethics Committee of the Capital Region of Denmark (H-16014580) and is registered at ClinicalTrials.gov (NCT03481400). All participants provided written informed consent to participate in accordance with the Declaration of Helsinki of 1964, with later revisions.

### P87 Mitochondrial DNA and migraine

#### Sigrid Børte^1,2^, Bendik S. Winswold^2,3^, Lars G. Fritsche^4,5^, Ida Surakka^6^, Jonas B. Nielsen^6^, Wei Zhou^7^, Cristen J. Willer^6,7,8^, John-Anker Zwart^1,2,3^

##### ^1^Research and Communication Unit for Musculoskeletal Health, Division of Clinical Neuroscience, Oslo University Hospital, Ullevål, Oslo, Norway; ^2^Institute of Clinical Medicine, Faculty of Medicine, University of Oslo, Oslo, Norway; ^3^Department of Neurology, Oslo University Hospital, Oslo, Norway; ^4^HUNT Research Centre, Department of Public Health and General Practice, Norwegian University of Science and Technology, 7600 Levanger, Norway; ^5^Department of Biostatistics, University of Michigan School of Public Health, Ann Arbor, MI 48109, USA; ^6^Department of Internal Medicine, Division of Cardiology, University of Michigan; Medical School, Ann Arbor, Michigan, 48109, USA; ^7^Department of Computational Medicine and Bioinformatics, University of Michigan, Ann Arbor, Michigan, 48109, USA; ^8^Department of Human Genetics, University of Michigan Medical School, Ann Arbor, Michigan, 48109, USA

###### **Correspondence:** Sigrid Børte (sigrid.borte@gmail.com)

***Background:*** Mitochondria are the main source of energy for neuronal functioning, through oxidative phosphorylation. Mutations in mitochondrial DNA and mitochondrial dysfunction have been suggested to be involved in migraine pathogenesis, partly because migraine seems to be disproportion- ally transmitted through the maternal line. However, no mitochondrial-wide association analyses have been performed yet.

***Methods:*** We used a sample of 57,924 genotyped individuals from the population-based Nord-Trøndelag Health Study (HUNT) from Norway. Mi- graine diagnoses were obtained by questionnaires. Samples were genotyped with the Illumina HumanCoreExome array,which included 369 mitochondrial variants. Variants were imputed using 2,202 sequenced individuals from the same HUNT population as reference panel, resulting in 858 mitochondrial variants for analysis. We also analysed 382,967 nuclear variants in and nearby genes coding for mitochondrial proteins. In addition, major European mito- chondrial haplogroups were assigned and analysed.

***Results:*** No single mitochondrial variants or any of the mitochondrial haplogroups were associated with migraine. One nuclear variant was signifi- cantly associated with migraine, located in the ABCD2-gene. The ABCD2- gene encodes an ATP-dependent transporter, present in the organelle mem- branes (including the mitochondrial). It is involved in transporting very long chain fatty acids and probably plays a role in protecting against oxidative stress.

***Conclusion:*** No mitochondrial single variants or haplogroups were as- sociated with migraine, indicating that inherited mutations in mitochondrial DNA does not explain the maternal-specific transmission of migraine. One significant variant in a nuclear encoded mitochondrial protein gene may sug- gest some degree of mitochondrial dysfunction in migraine, but the relevance of this variant is unclear.

### P88 Headache in Call Center Employees: an Occupational Disease

#### Ilir Alimehmeti^1^, Xhovana Kane^1^, Jera Kruja^2,3^

##### ^1^Department of Family and Occupational Health, Faculty of Medicine, University of Medicine, Tirana, Albania; ^2^Department of Neurosciences, Faculty of Medicine, University of Medicine, Tirana, Albania; ^3^Neurology Service, University Hospital Center “Mother Teresa”, Tirana, Albania

###### **Correspondence:** Jera Kruja (jkruja@gmail.com)

**Background and Aim:**

Headache is a common complaint, frequently referred as occupational. Call center employees face long hours in long and countless conversations, usually in sitting position in small personal spaces in large offices with numerous colleagues that produces additional stress and noise. Such an occupation is pursued by around 4.4% of the Albanian workforce. Thus, our aim was to assess headache severity among call center employees.

**Methods:**

During 2017, medical doctors visited a random sample of call center employees, which were invited online to answer to question pertaining to demographics, work conditions and health related issues. In detail, employees were asked about headache complaints and, if present, were invited to answer to the Migraine Disability Assessment test (MIDAS), in order to measure the impact of headache on their daily life. Moreover, data were obtained on education, marriage status, working hours, work experience, trouble with the supervisors, number of phone calls and contracts concluded, and achievement-related appraisal. In addition, depression was evaluated through the Beck’s Depression Inventory.

**Results:**

In total, 298 call center employees were enrolled, a sample composed of 183 females (61.4%) and 115 males (38.6%) with a mean age of 26.3 ± 5.6 years old, of which 255 (85.6%) were highly educated. Not surprisingly, all of them (100%) referred to suffer from some degree of headache. Mean working time was referred to be 7.74 ± 2.8 hours long. 35.9% of the sample was employed for less than a year in the call center industry, 40.6% for 1-5 years, and 21.8% for more than 5 years. Regarding to depression, 65.4% of the sample resulted in normal state, while 13.4% presented mild mood disturbance, 8.1% presented borderline clinical depression, 8.7% presented moderate depression, 2.3% presented severe depression, while 2.0% presented extreme depression. MIDAS test, headache-related disability was estimated as grade I in 16.8%, grade II in 19.1%, grade III in 30.5% and grade IV in 33.6% of the cases. In independent samples t-tests and chi-square tests, this distribution presented no statistically significant difference between age-groups, gender, education, marriage status, working hours and trouble with the supervisors. Nevertheless, more disability was positively associated with higher number of phone calls (Pearson chi-square 20.519, *p* 0.002). In regression analysis, higher MIDAS grade was positively correlate with depression (β 0.596, 95%CI 0.320-0.872, *p* <0.001) when adjusted for age, gender, working conditions and achievements.

**Conclusions:**

In our study resulted that all call center employees suffer from headache, paving the road towards regarding it as an occupational disease directly related to the number of phone calls. Moreover, 13% of the study group suffered from depression ranging from moderate to extreme, which significantly predicted higher grades of headache-related disability.

### P89 Depression is not the only cause of cognitive impairment in chronic migraine

#### Nina Latysheva^1,2*^, Diana Osipova^1^, Elena Filatova^1,2^

##### ^1^Department of Neurology, Institute for Professional Education, I.M. Sechenov First Moscow State Medical University, Moscow, Russia; ^2^Alexander Vein Headache Clinic, Moscow, Russia

###### **Correspondence:** Nina Latysheva (ninalat@gmail.com)

**Background**

Patients with various types of chronic pain frequently present with subjective cognitive complaints. Objective memory, attention and executive function deficits were demonstrated in 50–80% of patients with fibromyalgia.

This may be caused by the overlap in brain structures responsible for memory, attention, mood and chronic pain. New data is emerging on functional and structural changes in these brain areas, which may underlie cognitive impairment in depression and chronic pain. Cognitive deficits have been demonstrated in episodic migraine (EM) – both during attacks and interictally. Their severity correlated with headache frequency. The aim of this study was to evaluate subjective and objective cognitive impairment in patients with chronic migraine (CM).

**Methods**

We recruited 53 subjects with ICHD-3beta-defined CM and 22 gender- and age-matched controls with low-frequency EM (a maximum of 4 headache days per month), aged 18-59. All patients filled in the HADS anxiety and depression scale. Cognitive function was assessed with Montreal Cognitive Assessment (MoCA), Digital Symbol Substitution Test (DSST), Rey Auditory Verbal Learning Test (RAVLT) and the Perceived Deficits Questionnaire (PDQ-20). The study was approved by the Sechenov University’s Ethics Board.

**Results**

56% of CM and 9% of EM patients complained of memory impairment (p=0.00). Significant cognitive impairment was also demonstrated by PDQ-20 (24.4±12.4 vs. 18.2±7.6 in CM and EM, respectively, р=0.02). Objectively, we found a significant decrease in the 90-second DSST performance (41.6±10.0 vs. 50.6±8.9 in CM and EM, respectively, р=0.00), RAVLT total recall (32.4±12.1 vs. 38.5±12.2, respectively, р=0.04) and RAVLT learning rate (-0,7±1,6 vs. 0.14±1.0, p=0.01, respectively). 44% of CM subjects had MOCA-defined cognitive impairment, most often in attention (75%), memory/delayed recall (50%), and language (50%). Only 18% of EM patients scored lower than 26 points on the MoCA.

Depression correlated positively with RAVLT delayed recall only (r=0.78). Multiple linear regression showed that CM is an independent prognostic factor of lower DSST performance (р=0.03).

**Conclusions**

Subjective and objective memory and attention impairment is prevalent in the CM population.

As migraine becomes chronic, central sensitization and cognitive impairment become persistent and can be detected in the interictal period. Functional and structural changes in the brain observed in CM might underlie increasing cognitive impairment.

Even non-depressed CM patients need to be carefully screened for cognitive impairment.

### P90 Psychosocial risk factors for headache

#### M Zaletel, L Zaletel-Kragelj, B Žvan

##### University Clinical Center of Ljubljana, Department for vascular Neurology, Ljubljana, Slovenia

###### **Correspondence:** M Zaletel (mzaletel34@gmail.com)

**Background:** Headache is a common disabling condition related to high health system burden. It can be deleterious for psychological and social well-being. In Slovenia psychosocial factor for headache are not well established. To identify population groups at very high risk for headache and thus enable more focused prevention actions in Slovenia.

**Methods and subjects:** Data originate from the national survey carried out in 2012 which was a part of the CINDI program. A self-administered postal questionnaire were used. Multiple logistic regression was used to determine the impact of gender, age, education, employment, self-assessed social class, type of residence community, stress perception, coffer drinking behaviour, sleep behaviour on headache.

**Results:** We noticed high odds for risky stress behaviour (OR yes vs. no =1.99; *P*<0.001), sleep behaviour (OR < 6 vs. 8 hours/day = 1.23; p *P*<0.001) and coffee drinking behaviour (OR > 1cups vs. no coups/day = 1.58; p *P*<0.001) in headache subjects. In addition, we found the highest odds in women (OR women vs. men=1.99, *P*<0.001), aged 25-29 years (OR 25-29 vs. 70-74 = 6.10, *P*<0.001), participants with the lowest (OR primary vs. postgraduate =1.34, *P*=0.082). Regarding kind of work we detected higher odds in intellectual/leading positions (OR intellectual/leading positions vs. pensioners =1.39, *P*=0.014), participants self-classified in the lowest social class (OR lower vs. upper-middle = 1.65, *P=* 0.005), and in persons under 18 in household (OR yes vs. no = 1.15, *P*=0.028).

**Conclusions:** In Slovenia, intellectual/leading position women, aged 25-29 years, were identified as the largest population sub-group at high risk for frequent headache disorders with stress behaviour.

### P91 The prevalence and some risk factors chronic headache in Mongolia

#### Selenge Enkhtuya^1^, Otgonbayar Luvsannorov^1^, Byambasuren Tsenddorj^2^, Timothy J Steiner^3^

##### ^1^Mongolian National University of Medical Sciences, Department of Neurology; ^2^Mungunguur hospital; ^3^Imperial College London, London, UK

###### **Correspondence:** Selenge Enkhtuya (selenge317@gmail.com)

**Objective.** The aim of this study was to determine the 1-year prevalence and clinical characteristics of chronic headache in adult Mongolian population.

**Methods.** A cross-sectional, population-based survey consisting of semi-structured questionnaires was administered to randomly selected population aged 18–65 years, living in five geographically different regions of Mongolia using stratified multistage cluster sampling during the period from June to November 2017. The prevalence of chronic headache was calculated in the sample representing 2 million Mongolian adults. The questionnaire of primary headache was based on International Classification of Headache Disorders-III criteria.

**Results.** The surveyed totally 2043 participants; the one-year prevalence of all types of headache was 66.1%. The one-year prevalence of chronic headache was 11.2% (n=229), and 20.5% (n=47) of chronic headache declared having headache 30 days per month. About 70% (n=152) those with chronic headache also had medication overuse. Chronic tension-type headache and chronic migraine had one-year prevalence 1.3% (n=26) and 2.2% (n=45), respectively. The risk of chronic headache increased more than one fold and half when the participants were elderly participants, females, personal situation, low income and in those with low education. The most commonly overused medication was multi-therapy (acetylsalicylic acid, acetaminophen and caffeine) among the population.

**Conclusion.** The prevalence of chronic headache in Mongolia is high compare to other countries worldwide. These patients require special attention and should be offered multidisciplinary medical support.

**Keywords:** Chronic headache, Probable Medication Overuse Headache, prevalence

### P92 Symptoms of dependence in medication overuse headache: daily consumption versus days without consumption

#### J Azimova^1^, K Skorobogatykh^1^, A Amelin^2^

##### ^1^University Headache Clinic, Moscow; ^2^Pavlov First Saint Petersburg State Medical University

###### **Correspondence:** J Azimova (azimova.j@mail.ru)

**Objective**: International Classification of Headache Disorders (ICHD) defines Medication Overuse Headache (MOH) as headache occurring ≥15 d/month in a patient with a preexisting headache disorder who has regularly exceeded specific thresholds of symptomatic medication use [1]. MOH is the result of: (1) headache frequency progression or/and (2)dependence-related behavior - craving, a deficit in controlling substance intake, which is associated to orbitofrontal cortex (OFC) dysfunction [2]. Typical manifestations of addiction - craving and euphoria, are not MOH characteristics. In the present study we investigated the prevalence of addiction disorder among the chronic migraine patients

**Patients and methods**. 75 patients with chronic migraine (CM) and MOH (ICHD-3) mean age 41,1±12,8y.o. (21-65), 9 men and 66 women. We assessed the mean amount of doses of acute migraine drugs (triptans, combined analgetics) during 3 months and addiction with Leeds Dependence Questionnaire (LDQ).

**Results**. The mean amount of acute migraine drugs per month was 31,6±23,2 (mean±SD) doses per month per patient (10-90). Mean LDQ score was 13,6±6,4 (3-26). We divided the patients into two groups: with daily consumption of acute migraine drugs (group 1) – 25 patients, and the group with consumption free days (group 2) – 50 patients. The subjects in the group 1 had 30,0±0 headache days per month, 18,9±3,9 migraine days per month and consumed 59,2±21,0 doses of acute medication per month. The subjects in the group 2 had 24,6±5,9 headache days per month, 16,6±4,5 migraine days per month and consumed 17,8±4.2 doses of acute medication per month. We found that LDQ score was 17,5±4,8 in daily consumption group (group 1) and 11,7±6,3 the group with consumption free days (group 2) (p<0.0001).

**Conclusions**. Patients with MOH are heterogeneous – some of them could be addicted to acute medication, some not. Daily consumption of acute migraine drugs may be a sign dependence.

**References**:

1. Headache Classification Committee of the International Headache Society (IHS). The International Classification of Headache Disorders, 3rd edition (beta version). Cephalalgia 2013;33:629–808.
2. Radat F, Chanraud S, Di Scala G, et al. Psychological and neuropsychological correlates of dependence-related behaviour in medication overuse headaches: a one year follow-up study. J Headache Pain. 2013;14:59.

### P93 Effectiveness and safety of OnabotulinumtoxinA in the treatment of chronic migraine in patients older than 65

#### M. Castañón, E. Ameijide, J.M. Sánchez

##### Servicio de Neurología del Hospital Universitario Central de Asturias, Ausurias, Spain

###### **Corresponence:** M. Castañón (elenaviles90@hotmail.com)

**BACKGROUND**

Only two drugs have been approved for prophylactic treatment in chronic migraine, Topiramate and Onabotulinumtoxin A. Elderly population is increasing and this group is exposed to more drug interactions and greater risk of side effects. The PREEMPT study included patients aged 18-65 years old, but data are scarce in older than 65.

**METHODS**

We retrospectively reviewed medical records of patients with chronic migraine treated with Onabotulinumtoxin A. Patients older than 65 years old at first infiltration were selected. Responder was considered if reduction in headache days per month was greater than 50%.

**RESULTS**

Twelve patients (75% women), were indentified. Mean age at first infiltration was 70.08 year old (range 66-80). Infiltration was done according PREEMPT protocol and median dose was 192 u (105-240). Eight patients (66,6%), were considered responders. Nine patients (75%), reduced analgesics intake, although only one could reduced oral prophylactic treatment. Mild pain at the injection sites was the most common side effect.

**CONCLUSIONS**

Although a few patients, Onabolutinumtoxin A appears to be effective and safe also in patients older than 65 years old with chronic migraine.

### P94 Headache education and management in a multi centric study Cameroon

#### García-Azorín D^1^, Kurtis Urra M^2,^ Molina M^3^, Delgado-Suarez C^4^, Iglesias García P^4^, García-Morales I^5,^ Gonzalez-Monje M^6^

##### ^1^Hospital Clinico Universitario Valladolid, Headache Unit, Valladolid, Spain; ^2^Hospital Ruber Internacional, Movement Disorders Program, Department of Neurology, Madrid, Spain; ^3^ Hospital de Móstoles, Neurology department, Mostoles, Spain; ^4^Hospital Clinico San Carlos, Neurology, Madrid, Spain; ^5^Hospital Clínico San Carlos, Epilepsy Department, Madrid, Spain; ^6^Universidad Autónoma de Madrid, Neuroscience Department, Madrid, Spain

###### **Correspondence:** García-Azorín D (davilink@hotmail.com)

**Background:**

Headache is the main neurological condition in the high income countries, but prevalence of headache disorders is not well defined in low-income countries. In many hospitals, the absence of medical doctors obligate nurses to evaluate and treat patients. We aim to estimate the presence of headache in daily practice and evaluate education among the care providers.

**Methods:**

Observational analytic study conducted in a population of health care providers from the entire Cameroon. In October 2017, 7 Spanish neurologists organized a 4-day National Neurology Course, and one morning was dedicated to headache disorders. Before the course we performed a survey regarding sociodemographic and management variables and we evaluated the change after the course.

**Results:**

42 health care providers participated in the course. 52,4% were female with a mean age of 36,8 years. The mean number of patients consulted per week was referred of 64,3, among which, 21,1 complained of headache. The majority of participants mentioned headache as the most frequent neurological disorder in their clinics.

The concept of primary and secondary headaches was not clear: only 26,2% of participants considered migraine as a primary headache and only 12,2% tension type headache. 31,3% mentioned it specifically migraine as a secondary headache. Only 57% believed clinical history to be essential for the correct diagnosis and 40% recommended a cranial CT in every headache patient. When asked about basic headache signs and symptoms, 71,3% considered that an abnormal neurological examination could be seen in primary headaches while 50% thought fever could be a normal sign in migraine patients. Symptoms such as aggravation with movement, photophobia or nausea were considered as alarm symptoms of secondary headaches by 78,5%, 76,2% and 61,9% respectively.

Regarding etiology, 57,1% mentioned infections as the most frequent cause of headache, followed by head trauma (38,1%) and psychiatric disorders (31%). For the treatment of migraine the preferred option was paracetamol (47,6%) followed by NSAID (9,5%).

**Conclusions:**

Despite headache is the most frequent neurological condition in the Cameroonian clinical practice, many basic concepts about primary and secondary headaches are not properly understood. Identifying training gaps in developing countries is crucial in order to develop educational programs that could improve the management of those patients.

**Ethics approval and consent to participate:**

All the participants agreed to participate in the study.

### P95 Non-compliance with follow-up: analysis of two years attendance to a second level headache centre in Italy

#### Teresa Catarci, Isabella Falomi and Roberto Batistoni

##### Azienda sanitaria locale Roma1, presidio Luzzatti headache centre, Rome, Italy.

###### **Correspondence:** Teresa Catarci (teresa.catarci@aslroma1.it)

The management of headache patients is a complex process where many factors play a crucial role, as, for example, patients’ compliance with follow-up. Previously, we had monitored the first 12 months of activity of our secondary headache outpatient clinic and found a total dropout rate of 45%, where 74% missed the first follow-up visit and 60% suffered from chronic headache. In order to further study this important aspect of headache care, we decided to verify the dropout rate, after the first visit, which occurred in the following two years and possibly ascertain the motivations.

We retrospectively analysed daily worksheets and case records of the 328 consecutive patients where follow-up had been scheduled between January 2016 and end of December 2017, the first visit had to be performed within the previous 6 months. The following data were gathered: diagnosis and headache attacks frequency, language barriers and nationality, date of first visit and missed follow-up, type of withdrawal (cancellation or missed attendance), time of scheduled follow-up, time of cancellation. Phone calls have been planned in order to inquire about motivations of dropout and will be done as soon as the local Health Authorities will provide permissions. The same neurologist (TC) visited all patients but 3, seen by other substitute colleagues.

Forty-five patients did not return for follow-up (13.7%), 11 males, 34 females mean age 39.2 ± 12.5 years, 57.8% was foreigner, language barriers were present in only two. Twenty-six did not return at scheduled follow-up, 19 cancelled. Another 3 patients cancelled but no data were available.

Our second level headache centre had a dropout rate, after the first visit, of 13.7 %, similar to what we had previously found (13.9%). More than half of the patients were not Italian, but since very few language barriers were found, it is possible that in those cases a more difficult patient-doctor relationship was to be taken into account (cultural barrier). Nevertheless, a previous study performed on 316 consecutive patients of a third level headache centre in Boston, reported "dislike of the clinician and seeking care elsewhere” the main reason for non-compliance. We believe that it is critical to keep dropout rates at follow-up low, particularly in second level headache centres; also, the reasons of dropout should be investigated in order to improve headache quality of care and be considered as an important indicator.

### P96 A real-world analysis of global patient‑reported outcomes in patients with migraine

#### Janet H. Ford^1^, Sarah Cotton^2^, James Jackson^2^, Jeffrey S. Andrews^1^, Shonda A Foster^1^, Wenyu Ye, Russell M. Nichols^1^, Antje Tockhorn-Heidenreich^3^

##### ^1^Eli Lilly and Company, Indianapolis, USA; ^2^Adelphi Real World, Bollington, UK; ^3^Eli Lilly and Company, Erl Wood Manor, UK

###### **Correspondence:** Janet H. Ford (ford_janet@lilly.com)

**Background:** Preventive treatment use has demonstrated improved health-related quality of life (HRQoL) in patients with migraine. Preventive treatment is recommended for patients with migraine who experience ≥4 migraine headache days (MHDs)/month; however, many are untreated. It is important to assess if the goal of achieving <4 MHDs/month leads to better outcomes on HRQoL measures.

**Methods:** The study used a cross-sectional survey of physicians (Adelphi Migraine Disease Specific Program, 2017) and their consulting patients with migraine in the United States (US) and in five European countries (5-EU: Germany, France, UK, Italy and Spain). Country‑specific and pooled information was captured for 5-EU. Objectives were to describe and compare patient-reported outcome (PRO) measures across two subpopulations by country. This included: 1) Patients with ≥4 MHDs/month versus <4 MHDs/month (reported here), and 2) patients with ≥3 lines of migraine preventives versus 0 to 2 lines. The PRO measures included Migraine-Specific Quality of Life Questionnaire Version 2.1 (MSQ), Migraine and Disability Assessment Scale (MIDAS), EuroQoL-5D-5L (EQ-5D-5L) and Work Productivity and Activity Impairment (WPAI). Higher MSQ scores indicate better functioning, lower MIDAS scores indicate less disability and higher EQ-5D-5L scores indicate better health utility. Demographic and treatment information were also captured.

**Results:** There were no significant differences between the two subpopulations (≥4 MHDs/month versus <4 MHDs/month) for age, gender or ethnicity in any country, with the exception of the ≥4‑MHDs/month population having significantly higher proportion of females in the US (77.6% vs. 72.5%, p<0.05). Significantly higher proportion of patients with ≥4 MHDs/month were on long-term sick leave/unemployed/retired due to migraine in 5-EU (pooled result: 5.1% vs. 1.7%; p<0.01); US trends were similar, but not significant (9.6% vs. 1.6%, p>0.05). A significantly higher proportion in the ≥4‑MHDs/month group was considered refractory to preventive treatment per clinician’s opinion (US: 8.2% vs. 1.5%, 5‑EU pooled: 9.3% vs. 3.6%; p<0.01). Table 1 lists HRQoL results. A significantly greater proportion of patients with ≥4 MHDs/month had the following outcomes: moderate-to-very severe disability (MIDAS), greater functional impairment (MSQ), worse health utility (EQ-5D-5L) and 1.5 to 2 times more work/activity impairment and work absenteeism (WPAI). Similar trends of differences between the two populations were seen in individual countries of 5-EU.

**Conclusions:** These results suggest that patients with migraine who experience <4 MHDs/month experience better outcomes on PRO measures versus patients with ≥4 MHDs/month. This suggests that goals of treatment regimens for migraine should be targeted towards reducing the frequency to <4 MHDs/month.

**Ethics approval**

The DSP was conducted as a survey adhering to market research guidelines and codes of conduct according to the International Chamber of Commerce/European Society for Opinion and Marketing Research international code on observational research. Before completing the voluntary patient self-completion (PSC) form, patients were asked to provide written consent. Physicians and patients provided anonymized data. The survey was submitted to the Freiburger Ethic-Kommission International (FEKI) where approval was granted on 24^th^ October 2017 (FEKI Code 017/1763).

**Table 1 (abstract P96).** Patient‑reported outcome (PRO) measures in patients with migraine who experience ≥4 migraine headache days/month and <4 migraine headache days/month in the US and in five European countries

	US Population		5-EU Population	
	≥4 MHDs/ month	< 4 MHDs/ month	≥4 MHDs/ month	< 4 MHDs/ month
	(n=584)	(n=789)	(n=1942)	(n=2147)
Disability Category by MIDAS Value, n (%)				
Little/No	102 (41.5)	291 (69.5)	247 (35.2)	477 (56.4)
Mild	57 (23.2)	62 (14.8)	127 (18.1)	150 (17.8)
Moderate	40 (16.3)	44 (10.5)	182 (26.0)	127 (15.0)
Severe	30 (12.2)	18 ( 4.3)	101 (14.4)	77 ( 9.1)
Very severe	17 ( 6.9)	4 ( 1.0)	44 ( 6.3)	14 ( 1.7)
MSQ Domain, Mean (SD)				
Total	67.6 (21.2)	78.8 (19.1)	67.7 (18.6)	76.1 (17.4)
Role Function: Restrictive	63 (21.3)	75.6 (19.4)	64.2 (19.6)	72.4 (18.6)
Role Function: Preventive	73.5 (21.9)	82.1 (20)	72.3 (19.8)	79.9 (17.9)
Emotional	70.2 (24.9)	82 (22)	69.9 (21.8)	79.7 (19.8)
WPAI, Mean (SD)				
% work time missed due to problem	10.4 (22.8)	5.2 (18.1)	10 (21.4)	4 (13.7)
% impairment while working due to problem	36.5 (28.3)	21.7 (24.1)	34.5 (22.5)	21.9 (22)
% overall work impairment due to problem	40.3 (29.6)	22.7 (24.7)	39.7 (24.2)	23.4 (23.2)
% activity impairment due to problem	38.6 (27.4)	25.3 (24.8)	39 (23.4)	27.7 (24)
EQ-5D-5L, Mean (SD)				
EQ5D Utility Score	0.9 (0.1)	0.9 (0.1)	0.8 (0.2)	0.9 (0.2)
EQ5D Visual Analogue Scale	79.8 (14.7)	84.3 (13)	74.8 (15.8)	80.2 (15.4)

All comparisons between patients with ≥4 MHDs/month and <4 MHDs/month are statistically significant (p<0.01; Chi-square test for MIDAS value, t-test for all other comparisons). The 5-EU population consists of the combined populations from Germany, France, UK, Italy and Spain. *Abbreviation: MHD* migraine headache days.

### P97 ONAMIG: Does onabotulinumtoxinA modulate cortical excitability in chronic migraine?

#### A.Alpuente^1,2^, N.Raguer^3^, M.Torres-Ferrús^1,2^; VJ.Gallardo^2^, J.Álvarez-Sabín^1^, P.Pozo- Rosich^1^

##### ^1^Headache Unit, Neurology Department, Hospital Universitari Vall d'Hebron, Barcelona, Spain; ^2^Headache Research Group, Vall d'Hebron Research Institute (VHIR), Universitat Autònoma de Barcelona, Barcelona, Spain; ^3^Electromyography Unit, Neurophysiology Department, Vall d'Hebron University Hospital, Barcelona, Spain

###### **Correspondence:** P.Pozo- Rosich (ppozorosich@yahoo.com)

**Background:** Symptoms in migraine could be caused by changes in cortical excitability.

**Objective:** To analyze changes in cortical excitability in chronic migraine patients after onabotulinumtoxinA treatment.

**Methods:** We included patients diagnosed with CM with or without aura (ICHD-3 beta) without any preventive treatment and candidates for treatment with onabotulinumtoxinA, as well as patients with episodic migraine (EM) as controls.

We collected sociodemographic, clinical data, acute treatment intake, and disability scales. We evaluated cortical excitability using transcranial magnetic stimulation (TMS) parameters: resting motor threshold (RMT), cortical silent period (CSP) and shortinterval intracortical inhibition (SICI).

Using two-sided analysis and a level of 5% by means of t-test for independent samples and paired t-test for matched data, we analyzed clinical and neurophysiological data at baseline and after two cycles of onabotulinumtoxinA treatment (PREEMPT).

**Results:** Data was collected from 19 patients with CM and 16 controls (EM). We found a significant reduction in headache frequency after treatment with onabotulinumtoxinA (p-value=0.001). In TMS-measurements, after onabotulinumtoxinA treatment, RMT decreased (58.9±13.3% vs 55.1±11.9%, p-value=0.004), approaching control values (53.75±9.88%, p-value=0.019) and CSP increased (85.6±26.4ms vs 100.9±33.0ms, p-value=0.004) approaching control values (121.53±39.92, p=0.003). Measurements of greater inhibition in SICI predicted and improvement in frequency and intensity more than 50%.

**Conclusion:** In CM, after treatment with onabotulinumtoxinA cortical excitability changes which may indicate cortical modulation mechanisms.

**Ethics Approval**

The study was approved by CEIC institution with number MIG-ONA-2014-01.

### P98 Effectiveness of OnabotulinumtoxinA in chronic migraine. If early introduction: faster, cheaper and more satisfactory

#### Robert Belvís, Noemí Morollón, Marina Guasch, Paula Marrero, Azahara Aceituno, Carles Roig

##### Headache Unit. Dexeus University Hospital and Headache Unit. Santa Creu i Sant Pau Hospital, Barcelona, Spain

###### **Correspondence:** Robert Belvís (rbelvis@santpau.cat)

**Introduction**

Only OnabotulinumtoxinA and Topiramate have evidence I and grade A of recommendation in the treatment of chronic migraine (CM). However, many patients receive other oral drugs before starting OnabotulinumtoxinA treatment, delaying the start of onabotulinumtoxinA months or years.

**Objectives**

The aim of this study was to demonstrate that the early administration of OnabotulinumtoxinA, in the course of CM, improves the speed of its effectiveness, and reduces the number of cycles, dose and cost.

**Patients / methods**

Patients with CM (ICHD3:1.3) treated with OnabotulinumtoxinA with the PREEMPT injection paradigm in two Headache Units. We classified two groups of patients: start of OnabotulinumtoxinA at 3-9 months of the evolution of CM (early group) and at >9months (late group). Main variable: response rate (decrease>50% of days of migraine/month at 16 weeks). Secondary variables: number of cycles and dose of OnabotulinumtoxinA, percentage of completion of treatment, cost in euros, triptans consumption, and Likert satisfaction scale.

**Results**

90 patients were treated with Botox: early group-18 (average 6.2 months) and late group-72 (average 57.8 months). Overall response rate 80%: 94.4% in the early group: 76.3% in the late group (p=0.07). Early response: excellent-61.1%, good-33.3%, non-response-5.5%; vs. late: 36.1%, 38.8%, 25% (p=0.05, 0.6, 0.07, respectively). Treatment completion rate: early-50% / late-23.6% (p= 0.02); need for more than 4 cycles: early-16.6% / late-37.5% (p=0.09); maximum dose rate: early-5% / late-31.9% (p=0.02); Botox spending 1st year: early-923€ / late-1,137€ (p=0.03); reduction in average consumption of triptans: early-66.6% / late-64.7% (p=0.8); Likert satisfaction scale: early-4.5 / late-3.3 (p =0.009).

**Conclusion**

The rate of OnabotulinumtoxinA responders seems independent of the time of the chronic migraine in which treatment is started. However, the time it takes to achieve effectiveness is lower the earlier you start, so the patient receives fewer sessions and lower doses increasing their satisfaction. Additionally, the final cost of the treatment is minor.

### P99 Sickness absence and disability pension in cluster headache patients and in matched references: a population-based study

#### Anna Steinberg^1^, Pontus Josefsson^2^, Kristina Alexandersson^2^, Christina Sjöstrand^1^

##### ^1^ Division of Neurology, Department of Clinical Neuroscience, Karolinska Institutet, SE 171 77 Stockholm, Sweden; ^2^ Division of Insurance Medicine, Department of Clinical Neuroscience, Karolinska Institutet, SE 171 77 Stockholm, Sweden

###### **Correspondence:** Anna Steinberg (anna.steinberg@sll.se)

**Background**: Cluster headache, a primary neurovascular headache syndrome, is considered one of the most severe pain conditions known to humans. Nevertheless, there is little knowledge about sickness absence and disability pension among cluster headache patients.

The aim was to estimate the prevalence of cluster headache among people of working ages and to compare their rates of sickness absence and disability pension with such rates among matched references and to explore whether sociodemographic factors were associated with these rates.

**Methods**: A population-based register study of all 3240 people aged 16-64 who lived in Sweden all of 2010 and who at least once during 2001-2010 had in- or specialized outpatient care with cluster headache (ICD10 code G44.0) as the main diagnosis. They were compared with a reference group (N=16,200) matched on age, sex, type of living area, and level of education from the total population aged 16-64 years (N=5,945,895) regarding their sickness absence and disability pension in 2010. Crude and adjusted prevalence rates with 95% confidence intervals (CI) of being on full-time disability pension all 2010, part of 2010, and for having had at least one sickness absence spell >14 days, were computed for all and by different sociodemographics.

**Results**: The prevalence of cluster headache among working-aged people in Sweden was 0.054%. In 2010, sickness absence rates were 17.30% (CI: 15.93-18.68) among cluster headache patients and 9.16% (8.70-9.61) among references. In the cluster headache group a much higher rate of women had sickness absence (25.31% (CI 22.56-28.07)) compared to among men (13.38% (CI 11.87-14.89)) or full-time disability pension (women 13.17% (CI 11.17-15.17) compared to men (8.79% (CI 7.59-9.99)). Cluster headache patients >35 years had a higher rate of sickness absence and disability pension compared to references in the same age group. The difference became even more obvious in patient ages 55-64; disability pension 21.77% (18.89-24.65) compared to 11.90% (10.89-12.91). A higher rate of cluster headache patients born outside Sweden were on full-time disability pension; 13.56% (CI 10.85-16.28) compared to cluster headache patients born in Sweden; 9.51% (CI 8.39-10.63). Level of education in the cluster headache group was not associated with disability pension as much as in the reference group.

In summary, this nationwide study of working-aged people showed that cluster headache patients had sickness absence or disability pension to a much higher degree than matched references. Moreover, this varied much by sociodemographics, which needs to be addressed.

**Ethics approval**

The project was approved by the Regional Ethical Review Board in Stockholm, Sweden

### P100 Clinical features of pediatric medication overuse headache and applicability of new ICHD-3 criteria

#### Romina Moavero, Maddalena Stornelli, Laura Papetti, Barbara Battan, Samuela Tarantino, Federico Vigevano, Massimiliano Valeriani

##### Headache Center, Bambino Gesù Children’s Hospital, IRCCS, Rome, Italy

###### **Correspondence:** Romina Moavero (rominamoavero@hotmail.com)

**Objectives**. To describe the clinical phenotype of pediatric medication overuse headache (MOH) and to analyze the applicability of ICHD-3 criteria in comparison to the ICHD-2. MOH is characterized by headache occurring on ≥15 days/month in patients with pre-existing primary headache and developing as a consequence of regular overuse of symptomatic headache medication.

**Materials and Methods**. We conducted a retrospective analysis of clinical data of pediatric patients diagnosed with MOH in our Department. In all patients the clinical diagnosis of MOH was verified both according to ICHD-2 and ICHD-3 version criteria, to verify the degree of concordance.

**Results**. We identified 42 subjects diagnosed with MOH (31 F, 11 M), ranging between 8 and 17 years of age (mean 13.4 years). All patients presented with chronic migraine, with 9% fulfilling a diagnosis of migraine with aura. As for the clinical features of migraine, photo- and photophobia were both present in 81% of patients, nausea/vomiting in 30%, dizziness in 18%. Regarding the applicability of the ICHD-2 criteria, 21/42 (50%) fulfilled criterion A; 35/42 (83%) criterion B, 37/42 (88%) criterion C, and 23/42 (55%) criterion D. On the other hand, applying the criteria of ICHD-3, criterion A was fulfilled by 40/42 patients (95%), criterion B by 35/42 (83%), and criterion C by 40/42 (95%).

**Discussion**. Our data show that in comparison with ICHD-2, ICHD-3 criteria are satisfied by a higher rate of pediatric patients clinically diagnosed with MOH. The old criteria required a development or marked worsening of the headache during medication overuse, and a resolution within 2 months after medication withdrawal. Both these criteria disappeared in the new version of ICHD, being replaced by the requirement that the condition should be not better accounted by another ICHD-3 diagnosis. This means that now a diagnosis of MOH could be made in presence of a high frequency headache in a patient presenting a medication overuse, without the necessity of demonstrating a clear and direct correlation with abuse and discontinuation of symptomatic medications.

**Conclusion**. Our data show that ICHD-3 criteria allow a definite diagnosis of MOH in a higher rate of pediatric patients

### P101 C-tactile touch perception and habituation in migraineurs

#### Hanna Sophie Lapp^1^, Ilona Croy^2^, Gudrun Gossrau^1^

##### ^1^ Comprehensive Pain Center, Technische Universität Dresden, Dresden, Sachsen, 01307, Germany; ^2^ Department of Psychotherapy and Psychosomatic Medicine, Technische Universität Dresden, Dresden, Sachsen, 01307, Germany

###### **Correspodence:** Hanna Sophie Lapp (Hanna-Sophie.Lapp@uniklinikum-dresden.de)

**Background**: Migraine is characterized by sensory hypersensitivity and habituation deficits. Slow brushing over the skin activates c-tactile (CT) nerve fibres which mediate pleasant touch and analgesic effects in healthy subjects. As this function is altered in painful conditions, we aimed to examine whether the processing of CT fibres is also deranged in migraine.

**Methods**: Assessing c‐tactile function, we applied CT optimal (1cm/s, 3cm/s 10cm/s) and CT suboptimal (0,3cm/s, 30cm/s) brush stroking stimuli on both the dorsal forearm (body reference area) and the cheek as area with trigeminal sensory innervation of 53 interictal migraineurs and 53 matched healthy controls. For habituation testing, all subjects were presented 60 repeated CT optimal stimuli (3cm/s) in both test areas. The participants rated each stimulus on a 11-point visual analogue scale by intensity, pleasantness and painfulness. The study was approved by TU Dresden’s Ethics Board, approval number EK 412102017.

**Results**: Results showed no significant difference in pleasantness ratings between migraineurs and controls when assessing CT function. However, ratings decreased over time in both areas in migraineurs but not in controls during repeated stimulation (p<0.001). When comparing habituation of rarely and highly affected migraineurs, pleasantness ratings decreased in rarely affected subjects but remained stable in frequently affected subjects (p<0.001).

**Conclusion**: We conclude that there is no significant alteration of the c-tactile function in migraineurs. However, rarely affected migraineurs show a habituation deficit regarding CT processed touch whereas patients with frequent migraines show normal habituation. These findings correspond with previous research on habituation to other sensory stimuli.

### P102 Endeavor to identify associations between vaping and primary headaches

#### Marge Vaikjärv^1^, Kati Toom^2,3^, Mark Braschinsky^2,3^

##### ^1^Faculty of Medicine, University of Tartu; ^2^Headache Clinic, Tartu University Hospital ^3^Estonian Headache Society

###### **Correspondence:** Marge Vaikjärv (marge.vaikjarv@gmail.com)

**Introduction**

Smoking has been identified as one of the major risk and trigger factors of primary headaches. In recent years vaping as a socially more acceptable and presumably “healthier” form of nicotine consumption has increased in frequency. In recent studies vaping was associated with a lower and slower attainment of blood nicotine levels. The effects of vaping on human physiology, cognitive abilities and levels of different biomarkers are in the focus of many ongoing and completed studies. Although some of them bring out increase of headache frequency among vapers, associations between vaping and primary headache remains unknown.

**Objectives**

The objective of our study was to compare prevalence of primary headaches among vapers, non-smokers and smokers.

**Methods**

The data were derived from a population based survey conducted in Estonia form January 2016 till March 2017. The participants were asked about their age, sex, height and weight, daily coffee consumption, physical activity and occurrence, frequency and intensity of headaches. In order to specify the form of nicotine consumption respondents who agreed to give additional information after filling the survey, were contacted. Headache diagnoses based on given answers were determined according to ICHD-3 classification. Odd ratios (with 95% confidence interval) in all three groups for types of primary headaches (“migraine”, “tension type headache” and “others”) were calculated.

**Results**

Sample included 523 respondent, among whom 413 (79%) were non smokers, 101 (19%) smokers and 9 (2%) vapers. There was a statistically important difference in daily coffee consumption between non smokers and smokers as well as between non smokers and vapers but not between smokers and vapers. Primary headache prevalence was 82%, 77% and 78% among non smokers, smokers and vapers respectively.

**Conclusions**

The results indicated nicotine consumption to be a protective factor for primary headaches (ORs included 1) which differs from previous studies. While looking through our methodology we identified multiple probable biases which affected our results and made it impossible to give an adequate and reliable answer to the study objective. The most important ones being sampling and vaping as an form of nicotine consumption added to the original survey.

In order to study the possible connections between vaping and primary headaches data collection surveys should include vaping, and possible other nicotine consumption methods, separately from smoking.

**Ethics Approval**

The study was approved by Research Ethics Committee of the University of Tartu, approval number 252T-15.

### P103 NDPH sub-categories and treatment in pediatrics patients

#### Priyanka Shekhawat, Alex S Silver, Jessica Oppenheimer, Robert Fryer

##### Department of Child Neurology, Columbia University Medical Center - New York, USA

###### **Correspondence:** Priyanka Shekhawat (ps2761@cumc.columbia.edu)

New Daily Persistent Headache (NDPH) is a chronic non-migrainous headache disorder. As defined by the *International Classification of Headache Disorders* (ICHD-3 beta), NDPH is a headache lacking the characteristic features and is continuous and unremitting from its onset. Most patients can recall the exact moment the headache began. Rather than being a distinct disease, some experts suspect that NDPH is a syndrome having multiple underlying causes. Little information is currently available about this form of headache in the pediatric population. We are looking at the charts of patients seen in the Division of Child Neurology at Columbia University, who are of ages 5-21 and who have been diagnosed with NDPH between 1/1/2012 and 1/1/2016. Our goal is to determine if there are possible sub-categories of this disorder. A secondary goal is to characterize the range of treatments for these patients and identify the successful treatments for NDPH in children. The chart review will be complete at the time of conference.

### P104 Frequency, referral and demographic characteristics of patients with vestibular migraine from a tertiary centre

#### Isabel Luzeiro^1^, Isabel Pavão^2^

##### ^1^Centro Hospitalar da Universidade de Coimbra; ^2^University of Lisbon and Santa Maria Hospital, Lisbon

###### **Correspondence:** Isabel Luzeiro (isabeluzeiro@gmail.com)

**Background and goals**. Vestibular migraine is a recently-described entity, still classified in the Appendix of the ICHD-3 beta and of the 2018 ICHD-3 final classification. The tertiary headache clinic of the Hospital and University Centre of Coimbra receives patients from the emergency department, from the general Neurology Department and from consultations of other specialties and primary health care. Taking into account the new classification, we proposed to describe how many patients were referred to our consultation, to characterize them in demographic terms and to identify their origin.

Patients and methodology. This is a descriptive study, including all patients that were referred to our Headache Clinic in the last year.

**Results**. In a universe of 400 patients referred, 27 had vestibular migraine. Their age varies between 19 and 57 years. All of them have, at least, a level of secondary education; about 40% have a university degree. The marital status is miscellaneous. Patients referred because of a clinical hypothesis of vestibular migraine were sent by otorhinolaryngologists, with a percentage of 30%. No patients from primary care had this clinical hypothesis established. However, all patients were referred because of migraine.

**Discussion**. The percentage of patients with vestibular migraine diagnosed at our tertiary center was 6.8%, slightly higher than the results reported in literature for the general population. Age did not allow to define preferential associations for this type of pathology. All levels of education were equally affected. The analysis of these data suggests a lack of knowledge of this entity by the primary health care system. Otorhinolaryngologists seem to be the most alert physicians for this clinical entity.

### P105 Migraine comorbidity and cognitive performance in patients with focal epilepsy

#### Olivia AJ Begasse de Dhaem^1^, Chris Morrison^2^, Kimford J Meador^3^, Dale E Hesdorffer^1^, Sabrina Cristofaro^2^, Jacqueline French^2^, Mia T Minen^2^, on behalf of the HEP Investigators

##### ^1^Department of Neurology, Columbia University – New York Presbyterian Hospital, New York, New York, 10023, USA; ^2^Department of Neurology, New York University, New York, New York, 10016, USA; ^3^Department of Neurology, Stanford University, Palo Alto, California, 94304, USA

###### **Correspondence:** Olivia AJ Begasse de Dhaem (oab2109@cumc.columbia.edu)

**Background**: Migraine and epilepsy are comorbid diseases that are both associated with cognitive impairments. [1,2,3,4,5] The presence of cognitive impairment in migraine patients is thought to be independent of migraine severity, duration, or medications. [1] Using prospective data from the Human Epilepsy Project (HEP) that follows patients with newly diagnosed focal epilepsy, we will assess whether the comorbid presence of migraine affects cognitive testing scores in epilepsy patients.

**Methods**: The primary outcomes are the total and subtest differences in initial cognitive performance between epilepsy patients with migraine and epilepsy patients without migraine. Cognitive function is assessed with the Wide Range Achievement Test 4 (WRAT4) and elements of the Cogstate test battery. Logistic regression will adjust for potential confounders such as depression, anxiety, age, cardiovascular protective medications, cardiovascular disease, and history of head trauma.

**Impact**: We hypothesize that there will be significant differences in cognition between epilepsy patients with migraine compared to epilepsy patients without migraine. If this is the case, future work could evaluate whether there is an association between new migraine diagnosis at the end of the study and change in Cogstate scores (between the initial and last score). Biomarker evaluation, brain MRI, and EEG could also be considered to assess for the etiology of such differences.

Ethics Approval

The study was approved by Columbia University Institutional Review Board, AAAL5255.

Consent to publish

Informed consent was obtained from subjects or legal guardians.

Disclosures

The HEP study is supported by the Epilepsy Study Consortium (ESCI), a non-profit organization dedicated to accelerating the development of new therapies in epilepsy to improve patient care. The funding provided to ESCI to support HEP comes from industry, philanthropy and foundations (UCB Pharma, Eisai, Pfizer, Lundbeck, Sunovion, The Andrews Foundation, The Vogelstein Foundation, Finding A Cure for Epilepsy and Seizures (FACES), Friends of Faces and others).

Dr. Meador has received research support from the National Institutes of Health, the Patient-Centered Outcomes Research Institute, UCB Pharma and Sunovion Pharmaceuticals, and travel support from UCB Pharma. The Epilepsy Study Consortium pays Dr. Meador’s university for his research consultant time related to Eisai, GW Pharmaceuticals, NeuroPace, Novartis, Supernus, Upsher-Smith Laboratories, UCB Pharma, and Vivus Pharmaceuticals.

Dr. Begasse de Dhaem, Dr. Morrison, Dr. French, and Dr. Minen do not have disclosures.

The abstract only describes the protocol. However, the data analysis will be performed by the time of the European Headache Federation meeting.

References

[1] Waldie KE, Hausmann M, Milne BJ, Poulton R. Migraine and cognitive function: a life-course study. Neurology. 2002 Sep 24;59(6):904-8.

[2] Precenzano F, Ruberto M, Parisi L, Salerno M, Maltese A, Gallai B, Marotta R, Lavano SM, Lavano F, Roccella M. Visual-spatial training efficacy in children affected by migraine without aura: a multicenter study. Neuropsychiatr Dis Treat. 2017 Jan 27;13:253-258.

[3] Pellegrino Baena C, Goulart AC, Santos IS, Suemoto CK, Lotufo PA, Bensenor IJ. Migraine and cognitive function: Baseline findings from the Brazilian Longitudinal Study of Adult Health: ELSA-Brasil. Cephalalgia. 2017 Jan 1:333102417737784.

[4] Haut SR, Bigal ME, Lipton RB. Chronic disorders with episodic manifestations: focus on epilepsy and migraine. Lancet Neurol. 2006 Feb;5(2):148-157.

[5] Smith DB, Craft BR, Collins J, Mattson RH, Cramer JA. Behavioral characteristics of epilepsy patients compared with normal controls. Epilepsia. 1986 Nov-Dec;27(6):760-8.

### P106 Nosographic analysis of osmophobia and field testing of diagnostic criteria including osmophobia

#### Mona Ameri Chalmer, Thomas Folkmann Hansen, Jes Olesen, Professor

##### Department of Neurology, Danish Headache Center, Copenhagen University Hospital, Glostrup, Denmark

###### **Correspondence:** Mona Ameri Chalmer (mona.ameri.chalmer@regionh.dk)

**Introduction**

Omophobia has been suggested as an additional symptom of migraine without aura (MO) and high prevalence of osmophobia up to 50% has been reported in the literature. We conducted a nosographic study of osmophobia in all migraineurs and tension-type headache (TTH) patients and a field testing of suggested diagnostic criteria of osmophobia, presented in the appendix of the second edition of The International Classification of Headache Disorders (ICHD-2)[1] and suggested by Silva-Néto et al[2] and Wang et al[3], in MO and TTH patients (n=1,934).

**Materials and methods**

All patients were carefully phenotyped and fulfilled the ICHD-2 diagnostic criteria for migraine or TTH. Statistical analyses were performed using statistical software R. The statistical R package “Caret” was used to construct a confusion matrix and retrieve sensitivity, which was defined as the suggested criteria’s ability to correctly diagnose MO patients, and specificity, defined as the suggested criteria’s ability to not wrongly diagnose TTH patients.

**Results**

Osmophobia was present in 33.5% of patients with migraine with aura, in 36.0% of patients with MO, and in 1.2% of patients with TTH. All migraineurs with osmophobia also fulfilled the current criteria for migraine by having nausea or photophobia and phonophobia. The appendix criteria had a sensitivity of 0.96 and a specificity of 0.99 for MO, and a sensitivity of 0.65 and a specificity of 0.99 for probable MO (pMO). Both the criteria by Silva-Néto et al and Wang et al had a sensitivity of 0.98 and a specificity of 0.99 for MO, and a sensitivity of 0.66 and a specificity of 0.99 for pMO.

**Discussion**

This study demonstrates the remarkable specificity of osmophobia. The criteria by Silva-Néto et al and by Wang et al both had a higher sensitivity than the appendix criteria for MO; all three criteria had a low sensitivity for pMO. However, neither the appendix criteria nor the criteria by Silva-Néto et al or Wang et al added any extra patients that would not have been diagnosed by the current diagnostic criteria for migraine. Osmophobia is a valuable symptom that may be useful to differentiate between MO and TTH in difficult clinical cases.

**Conclusion**

Our results do not suggest that alterations of the current diagnostic criteria for MO are needed.

**Ethics Approval**

Our research group has permissions and approval from the Data Protection Agency (GLO-2010-10) and the Ethical Committee (H-2-2010-122).

**References**

1. International Headache Society (1997) The International Classification of Headache Disorders, 2nd edition. Cephalalgia 9–160 . doi: http://dx.doi.org/10.1016/B978-0-7506-3365-9.50006-7

2. Silva-Néto RP, Rodrigues ÂB, Cavalcante DC, et al (2017) Reply to the Letter to the Editor: “smell of migraine: Osmophobia as a clinical diagnostic marker.” Cephalalgia 37:907–908 . doi: 10.1177/0333102416658716

3. Wang Y-F, Fuh J-L, Chen S-P, et al (2012) Clinical correlates and diagnostic utility of osmophobia in migraine. Cephalalgia 32:1180–1188 . doi: 10.1177/0333102412461401

### P107 Living with migraine: a report from the My Migraine Voice survey

#### Audrey Craven^1^, Rebeca Quintana^2,^ Veruska Carboni^3^, Paolo Martelletti^4,5^, Michel Lanteri Minet^6^, Todd J. Schwedt^7^, Hans-Christoph Diener^8^, Annik K-Laflamme^9^, Annette Vangaa Rasmussen^10^, Elena Ruiz de la Torre^11^, Donna Walsh^12^, Simon Evans^13^, Paula Dumas^14^, Rachel Fink^9^, Angela Fiorin^9^, Stephanie Ribbe^9^, Pamela Vo^9^

##### ^1^Migraine Association Ireland, Dublin, Ireland; ^2^GFK, Madrid, Spain; ^3^GfK Health, Basel, Switzerland; ^4^Department of Clinical and Molecular Medicine, Sapienza University of Rome, Rome, Italy; ^5^EHF; ^6^Département d’Evaluation et Traitement de la Douleur, Centre Hospitalo-Universitaire de Nice, France; ^7^Neurology Arizona, Mayo Clinic, Phoenix, USA; ^8^Department of Neurology and Headache Center, University Duisburg-Essen, Germany; ^9^Novartis Pharma AG, Basel, Switzerland; ^10^Rigshospitalet Glostrup, Copenhagen, Denmark; ^11^European Headache Alliance; ^12^European Federation of Neurological Associations, Brussels, Belgium; ^13^Migraine Action, Leicester, United Kingdom; ^14^Migraine Again, USA

###### **Correspondence:** Audrey Craven (audreycraven@migraine.ie)

**Introduction**

As a pilot to the My Migraine Voice study, online bulletin boards provided insights into the impact of migraine on the lives of those affected and the coping mechanisms used.

**Objectives**

The objective of this worldwide survey was to better understand what it is like to live with migraine, as directly reported from patients across the world.

**Methods**

My Migraine Voice is a worldwide cross-sectional online survey of 11,266 individuals (31 countries in Africa, America, Asia and Europe) recruited via online panels and patient organizations. Participants were adult migraine patients who reported ≥4 MMD in the 3 months preceding survey administration, with pre-specified 90% among those having reported having used preventive migraine treatment.

**Results**

A total of 11,266 migraine individuals participated (75% female, mean age 39 years); most were in employment/students (73%), with 9% receiving disability-related allowances due to migraines. 85% of participants reported negative aspects of living with migraine (feeling helpless, depressed, not understood), sleeping difficulties (83%); 55% lived in fear of the next attack. Migraines were associated with severe pain (85%), long-lasting headaches (lasting 4 to 72 hours) (83%), sound sensitivity (81%) light sensitivity (74%; mean=19 hours/month spent in darkness). Migraine impact on professional, private or social domains was reported by 87% of participants. Over the previous 3 months, 61% had relied on external support (family/friends/anyone else) to cope with daily tasks (mean=12.8 days). Despite the negative aspects, 57% of respondents indicated >=1 positive aspect, mainly relating to learning to cope with their disease (40%), or making them responsible for their disease (13%), and being stronger as a person (11%).

**Conclusion**

This study describes the daily reality of migraine individuals, especially those with frequent attacks and who have received migraine preventive treatments. While it highlights the significant challenges and unmet needs for these individuals suffering with migraine, the positive outlook on personal growth brought from coping with the disease highlights their resilience and strength.

**Ethics approval**

Data was handled confidentially and anonymity of respondents was maintained throughout the study. Participants’ consent was obtained prior to participation in the survey.

**Funding**

This study was funded by Novartis Pharma AG, Basel, Switzerland

### P108 A model concept for assessing the cost-effectiveness of prophylactic migraine treatments

#### Ronan Mahon^1*^, Pamela Vo^2^, Philip Cooney^1^, Andrii Danyliv^1^, Aikaterini Bilitou^1^, Nagasuman Toram^3^, Sreelatha Vadapalle^3^, Jasper Huels^2^, Farooq Maniyar^4^, Peter J Goadsby^5^ Mark Sculpher^6^

##### ^1^Novartis Global Services Centre, Patient Access Services, Dublin, Ireland; ^2^Novartis Pharma AG, Basel, Switzerland; ^3^Novartis Global Services Centre, Patient Access Services, Novartis Healthcare Pvt. Ltd., Hyderabad, India; ^4^Basildon and Thurrock University Hospitals and Queen Mary University, London, England; ^5^NIHR-Wellcome Trust, King’s Clinical Research Facility, King’s College London, UK; ^6^Centre for Health Economics, University of York, Heslington, Alcuin 'A' Block, York, YO10 5DD UK

###### **Correspondence:** Ronan Mahon (ronan.mahon@novartis.com)

**Objectives**

Migraine is a distinct neurological disease ranking among the top ten leading causes of disability [1]. Erenumab is a fully human monoclonal antibody (mAb) targeting the canonical calcitonin gene-related peptide (CGRP) receptor [2]. The objective of this research was to develop an economic model in order to assess the cost-effectiveness of erenumab as a migraine prophylactic treatment compared to relevant alternatives.

**Methods**

Relevant clinical guidelines (e.g. BASH and NICE) and previous economic evaluations were researched in order to understand clinical practice and previous modelling approaches in migraine prevention. Cost- effectiveness model concepts were devised and evaluated. Key opinion leaders, within both the medical and HE&OR fields, were consulted in order to select a model structure that is clinically and economically meaningful.

**Results**

A decision-tree plus Markov structure was developed as a cost-effectiveness model for erenumab in the preventive treatment of migraine. Reflecting clinical practice, the decision-tree component represents an assessment period, allowing for treatment discontinuation based on safety and clinically relevant response criteria. The Markov component represents a post-assessment period, where treatment responders and non-responders follow distinct treatment pathways. Responders, without safety or tolerability issues, continue treatment over the post-assessment with an optional re-evaluation period which may lead to positive discontinuation, while non-responders discontinue and do not reinitiate preventive treatment. The underlying assumption of the model is that both costs and quality-adjusted life-years (QALYs) can be estimated based on monthly migraine day (MMD) frequency. Thus, each health state in the model is associated with a patient distribution across MMD frequencies.

**Conclusions**

A decision-tree plus Markov model reflecting clinical practice was constructed to assess the cost- effectiveness of erenumab in the prophylactic treatment of migraine, estimating both, migraine patients’ MMD frequencies and their response to treatment.

**Funding**

This study was funded by Novartis Pharma AG, Basel, Switzerland

References

1. Vos, T. et al. Global, regional, and national incidence, prevalence, and years lived with disability for 328 diseases and injuries for 195 countries, 1990–2016: a systematic analysis for the Global Burden of Disease Study 2016, 2017; 390; 10100: 1211-1259.
2. Goadsby, P.J., Reuter, U., Hallstrom, Y., Broessner, G., Bonner, J.H., Zhang, F., Sapra, S., Picard, H., Mikol, D., Lenz, R.A. A Controlled Trial of Erenumab for Episodic Migraine, The New England Journal of Medicine, 2017; 377: 2123-32.

### P109 Relative efficacy of outpatient infusion therapy on pediatric patients with post-concussive headaches

#### Sara J Gould^1^, Erin Mackenzie Katz^2^, Carly Ann Cignetti^2^

##### ^1^University of Alabama at Birmingham; ^2^University of Alabama at Birmingham School of Medicine

###### **Correspondence:** Sara J Gould (sgould@uabmc.edu)

**Background**:

Concussion is an increasingly common diagnosis in the pediatric population. Our center has previously reported that among children who suffer from concussion symptoms longer than 10 days, over 98% report headache as a symptom.^2^ Standard practice in post-traumatic headache management dictates that prophylactic medications should not be started within 3 weeks of the injury, due to the rapid fluctuation and resolution in symptoms that often occurs following concussion. ^3^ However, the severity of the headaches can make activities of daily living difficult or impossible for the headache sufferers. Scholastic activities can suffer due to the inability to attend school or perform coursework. Intravenous therapy has been shown to be effective in the emergency department setting for post-traumatic headaches. ^4^ Therefore, we developed an infusion clinic to serve as an abortive therapy for intractable headache symptoms following concussion.

**Methods**: We administered an intravenous (IV) cocktail consisting of ketorolac, Compazine, diphenhydramine and 20mg/kg bolus of normal saline. The infusion was administered over. Patients followed up with their providers at an appointment following the infusion. Concussion Symptom Severity Scores were documented at physician visits before and after the infusion for each patient.

**Results**: We had a total of 27 pediatric patients, age 18 years or less who received an infusion from 2016-2018. 85% (23) of patients reported that the infusion helped diminish their headache. 4% (1) said that the infusion did not help at all. 11% (3) were not asked by their physician at the follow up visit about the infusion. Average symptom severity scores before the infusion were 57.5. Following infusion, the average symptom severity score decreased to 22. Headaches were rated on a scale of 0-6 on the symptom severity score. The average severity of the headache was rated at 4 prior to the infusion and rated at 2 following infusion.

**Discussion**: Following the infusion protocol, patients ranked their headaches an average of 2 points lower on the symptom severity score. Overall symptom burden decreased at follow up visit as well, from 57 at the initial visit to 22 following the infusion.

**Conclusion**: An outpatient infusion clinic may be an effective means to control subacute post-concussive headaches. Further research with randomized controlled trials should be conducted to determine efficacy of the protocol.

**References**

1. Nonfatal traumatic brain injuries related to sports and recreation activities among persons aged </=19 years--United States, 2001–2009. MMWR Morb Mortal Wkly Rep. 2011;60:1337–42.
2. Academic Difficulty and Vision Symptoms in Children with Concussion. Swanson MW, Weise KK, Dreer LE, Johnston J, Davis RD, Ferguson D, Hale MH, Gould SJ, Christy JB, Busettini C, Lee SD, Swanson E. Optom Vis Sci. 2017 Jan;94(1):60-67
3. Part II – Management of pediatric post-traumatic headaches. Pinchefsky E, Dubrovsky AS, Friedman D, Shevell M. Pediatr Neurol. 2015 Mar;52(3):270-80
4. Intravenous migraine therapy in children with posttraumatic headache in the ED. Chan S, Kurowski B, Byczkowski T, Timm N. Am J Emerg Med. 2015 May;33(5):635-9.

### P110 Movement disorders as positive motor aura symptoms during hemiplegic migraine attacks

#### Elisa de la Cruz^1^, Geneviève Demarquay^2^, Caroline Roos^3^, Isabelle Sabatier^4^, Florence Riant^5^, Victoria Gonzàlez^1^, Anne Ducros^1^

##### ^1^Service de Neurologie, CHU Gui de Chauliac, 34090 Montpellier, France; ^2^Service de Neurologie clinique et fonctionnelle, Hôpital Pierre Wertheimer, 69667 Bron, France; ^3^Centre d’Urgences Céphalées, Hôpital Lariboisière, 75010 Paris, France; ^4^Service de Neuropédiatrie, Hôpital Femme Mère Enfant, 69677 Bron, France; ^5^Laboratoire de Génétique moléculaire, Hôpital Lariboisière, 75010 Paris, France

###### **Correspondence:** Elisa de la Cruz (elisa@sdlc.ca)

**Background:** Migraine aura includes positive and negative symptoms. Positive motor symptoms (i.e movement disorders) have not been described so far during hemiplegic migraine (HM) attacks.

**Methods:** Patients were included if they had at least 2 attacks of hemiplegic migraine, and abnormal movements during the aura. After written informed consent, patients were interviewed and examined. Testing for FHM mutations in the CACNA1A, ATP1A2, SCN1A, PRRT2 genes or for a CADASIL mutation in the NOTCH3 gene has been done previously, for diagnostic purposes.

**Results:** Seven unrelated patients (2 males, 5 females) satisfied inclusion criteria. Five had familial HM (FHM), associated with a mutation in CACNA1A in 1 patient (a deletion without known pathological significance), ATP1A2 in 2 patients, and SCN1A in 1. Relatives with FHM had no movement disorders. Two patients had sporadic HM (1 with a S218L mutation on CACNA1A gene, and 1 without identified mutation). One patient had a secondary form of HM due to CADASIL with an archetypal NOTCH3 mutation. Seven patients had typical attacks with motor deficit (n = 7) associated with sensory (n = 6), language (n = 7) and visual symptoms (n = 5), that started at a mean of 8,3 (±3,6) years old. All 7 had movement disorders which appeared as a brief (mean duration 10,8 ± 5,3 minutes) and stereotyped component of their aura, with dystonic posturing in all and choreoathetosis in 4. The patient with the S218L mutation of CACNA1A had choreoathetosis during a prolonged severe HM attack with coma. Paroxysmal movements predominantly affected one upper limb and preceded the onset of ipsilateral paresis.

**Discussion:** Dystonia and dyskinesia may occur during the aura in any variety of familial or sporadic HM, either primary due to mutations of FHM genes or secondary to CADASIL. Cortical spreading depression might induce activation of different cerebral pathways generating movement disorders and then paresis. Functional imaging during attacks might help elucidate underlying mechanisms.

**Consent for publication**: Informed consent to publish was obtained from all patients or legal tutors.

### P111 Long-term response to onabotulinumtoxin A in chronic migraine: analysis of efficacy and tolerability in a series of 40 patients

#### D García-Azorín, A Sierra, J Trigo, E Martínez-Pías, AL Guerrero

##### Headache Unit, Neurology Department. Hospital Clínico Universitario, Valladolid, Spain

###### **Correspondence:** García-Azorín D (davilink@hotmail.com)

**BACKGROUND:** The efficacy and safety of OnabotulinumtoxinA (OnabotA) in Chronic Migraine (CM) has been established both in clinical trials and in real-world setting. However, there is less information about tolerability and maintenance of efficacy in a long-term scenario. We aimed to analyze both efficacy and tolerability in a series of patients treated for a long time

**PATIENTS AND METHODS:** Patients with Chronic Migraine attended in a Headache Unit in a tertiary hospital. Treatment with OnabotA was recommended in patients non-responders to Topiramate and at least one other oral preventative, according to local guidelines. We prospectively collected demographic data and migraine characteristics from all the patients. We also recorded information about tolerability, headache days, migraine days, and the number of days on which patients used acute headache medications, in particular triptans. We specifically analyzed efficacy and tolerability in patients who had reached at least 10 OnabotA procedures according to PREEMPT protocol

**RESULTS:** We included 40 patients (34 female, 6 male), with 40.8 ± 12.3 years (16 - 69) at inclusion. Latency between migraine and CM onset and OnabotA therapy was respectively 22.7 ± 12.6 years and 26 ± 22.8 months. In 8 of these patients (20%), a decrease in the response time below 3 months was observed between the 5th and 8th procedures. This “wearing-off” response improved in most patients increasing OnabotA dose according to "follow the pain" protocol. In 12 cases (30%) an adverse effect appeared, mainly musculoskeletal pain or stiffness mainly in occipital location and, in one patient, fronto-temporal atrophy

**CONCLUSION:** Long-term decrease of efficacy and adverse effects were not rare in our series. We should be aware of this possibility after the third year of OnabotA treatment

### P112 Acute and preventive treatment patterns and associated work productivity and activity impairment among patients with migraine in Germany

#### J. Scott Andrews^1*^, Janet Ford^1^, Grazia Dell’Agnello^2^, Sarah Cotton^3^, Antje Tockhorn-Heidenreich^4^, Louise Lombard^1^, Zoe Phillips^3^, James Jackson^3^

##### ^1^Eli Lilly and Company, Indianapolis, IN, 46285, USA; ^2^Eli Lilly and Company, Florence, Italy; ^3^Adelphi Real World, Bollington, UK; ^4^ Eli Lilly and Company Limited, Windlesham, UK

###### **Correspondence:** Grazia Dell’Agnello (andrews_jeffrey_scott@lilly.com)

**Background**

Limited research exists on patients with migraine in Germany that addresses both acute and preventive treatment patterns and the productivity impact of migraine, which has been recognized as a significant contributor to societal burden. The objective of this study was to characterize treatment patterns, work productivity, and daily activity impairment associated with various levels of headache frequency among patients with migraine in Germany.

**Methods**

Data were taken from the 2017 Adelphi Real World Migraine Disease Specific Programme, a point-in-time survey of physicians and their patients with a diagnosis of migraine in Germany. Physicians (Primary care = 51, Neurologist = 40) completed patient record forms (n = 810) containing patient demographics, comorbidities, headache frequency, diagnosis, treatment practice, medication utilization, and unmet needs of current treatments. Productivity was captured via patient report on the Work Productivity and Activity Impairment Questionnaire (WPAI). Patients were stratified by the frequency of headache days (HD) experienced per month (0-3, 4-7, 8-14, 15+).

**Results**

Most patients with migraine experienced 0-3 (40%) or 4-7 (42%) HD/month. Mean duration from first migraine experienced to first diagnosis was 3.4 months. Mean duration from first diagnosis to first prescribed acute and preventive treatments was 2.2 months and 23.1 months, respectively. The majority of patients (70%, 0-3 HD; 68%, 4-7 HD; 55%, 8-14 HD; 63%, 15+HD) were prescribed acute treatment only, while 18% (0-3 HD), 25% (4-7 HD), 39% (8-14 HD), and 36% (15+HD) received preventive treatment. Sumatriptan and ibuprofen were the most commonly prescribed acute treatments across all headache frequency categories, while metoprolol and topiramate were the most frequently prescribed preventive treatments. Over the counter (OTC) medication use ranged from 17% in 0-3 HD group to 27% in 15+ HD group. Across all levels of headache frequency, at least 1 in 4 patients with migraine experienced impairment in work productivity and activity. The top physician-reported unmet needs with acute treatments included: speed of action / a faster acting drug needed, minimal / acceptable side effect profile, and minimal / no cardiovascular risk. Top unmet needs for preventives included: minimal side effects / acceptable side effect profile, effective medication, and tolerability.

**Conclusions**

Most patients in Germany are treated with acute medications only, although many experience headache frequencies indicating prevention eligibility. Notably, patients with chronic headache frequency (15+ HD) have low preventive treatment use and high combination OTC and acute prescription use. Considerable impairment in work productivity and daily activity was observed across all headache frequency groups.

### P113 Baseline demographics and disease characteristics of patients with episodic cluster headache: results from a phase 3 clinical trial

#### James M. Martinez, Jennifer N. Bardos, Tina M. Myers Oakes, Chunmei Zhou

##### Eli Lilly and Company, Indianapolis, IN, 46285, USA

###### **Correspondence:** Jennifer N. Bardos (bardos_jennifer_n@lilly.com)

**Background:** Cluster headache (CH) is a disabling primary headache disorder characterized by episodic attacks of intense unilateral headache with autonomic symptoms and/or restlessness or agitation. Patients with episodic CH (approximately 85.0% of CH patients) have cluster periods typically lasting 2-12 weeks and differ diagnostically from chronic CH patients based on duration of remissions. Increased blood levels of calcitonin gene-related peptide (CGRP) have been associated with CH, making CGRP a potential therapeutic target. The objective of this study was to assess the efficacy and safety of galcanezumab, a CGRP monoclonal antibody, in patients with episodic CH. In this abstract, we report on baseline demographics and disease characteristics of these patients.

**Methods:** This phase 3, randomized, double-blind, placebo-controlled study enrolled patients aged 18-65 years who met International Classification of Headache Disorders, 3rd edition, beta version diagnostic criteria for episodic CH and had a prior history of a cluster period that lasted ≥6 weeks. During the prospective baseline, patients were required to have a total of ≥4 attacks, with an attack frequency of at least one attack every other day but ≤8 attacks/day. Certain concomitant abortive (but not preventive) treatments were allowed. Eligible patients were randomized to galcanezumab 300 mg or placebo administered subcutaneously once monthly for 2 months. Analyses were conducted on an intent-to-treat population.

**Results:** A total of 106 patients were randomized and treated with galcanezumab 300 mg (N=49) or placebo (N=57). Overall, the patient population was predominately male (83.0%) and white (84.9%), with a mean age of 46.4 years and the majority from Europe (66.0%). Mean duration of CH illness was 16.8 years. Lifetime suicidal ideation and suicidal behavior before screening was reported by 13.2% and 0.9% of patients, respectively. Current tobacco and nicotine combined use was reported by 53.8% of patients, while 26.4% reported prior use. The most common pre-existing conditions were insomnia (10.4%), gastroesophageal reflux disease (10.4%), and hypercholesterolemia (7.6%). During the prospective baseline period, patients had an average of 17.5 CH attacks per week. Average pain severity of the CH attack was 2.5 on a 5-point scale (moderate to severe). The average weekly total of CH attack duration was 15.5 hours. The proportion of patients using oxygen and/or subcutaneous sumatriptan during the prospective baseline period was 45.3% and 56.6%, respectively.

**Conclusion:** These data build upon the existing data to provide descriptive characteristics of the episodic CH population.

**Ethics approval**

The study was approved by a central Ethics Review Board and registered on ClinTrials.gov (NCT02397473).

### P114 PrevenBox: Evaluation of concomitant use of preventive medications with OnabotulinumtoxinA in migraine

#### Marta Torres-Ferrus^1,2^; Sonia Santos Lasaosa^3^; Angel Guerrero Peral^4^; Jose M Laínez^5^; Javier Viguera Romero^6^; Ana B Gago Veiga^7^; Pablo Irimia^8^; Margarita Sanchez del Río^9^; Laila Asskour^2^; Victor J Gallardo^2^; Patricia Pozo-Rosich^1,2^

##### ^1^Headache Unit, Neurology Department, Vall d’Hebron University Hospital, Barcelona, 08035, Spain; ^2^Headache Research Group, Vall d’Hebron Research Institute, Barcelona, 08035, Spain; ^3^ Neurology Department, Hospital Clínico Universitario Lozano Blesa, Zaragoza, 5009, Spain; ^4^ Headache Unit, Hospital Clínico Universitario de Valladolid, Valladolid, 47007, Spain; ^5^ Department of Neurology, Hospital Clinico Universitario de Valencia, Universidad Católica de Valencia, Valencia, 46010, Spain; ^6^ Department of Neurology, Hospital Virgen Macarena, Sevilla, 41009, Spain; ^7^ Department of Neurology, La Princesa Health Research Institute, La Princesa University Hospital, Madrid, 28006, Spain; ^8^ Department of Neurology, Clínica Universidad de Navarra, Pamplona, 31008, Spain; ^9^ Department of Neurology, Clínica Universidad de Navarra, Madrid, 28027, Spain

###### **Correspondence:** Patricia Pozo-Rosich (ppozo@vhebron.net)

**Background**: OnabotulinumtoxinA is an effective, tolerable and safe preventive treatment for chronic migraine (CM). Other than a reduction in headache frequency or disability, in CM the withdrawal of concomitant preventive medication indicates treatment effectiveness and quality of life improvement.

**Objective**: To characterize the change in the use of oral preventive medication after treatment with OnabotulinumtoxinA in patients with migraine.

**Methods**: This is a multicentre study. We consecutively included patients with migraine (ICHD-3) that were on preventive treatment with OnabotulinumtoxinA. We retrospectively collected demographic data, diagnosis of migraine, frequency and intensity changes, number of cycle and OnabotulinumtoxinA dose. In addition, we listed the initial and current preventive treatment (number of drugs and group) and the number and cycle of medications withdrawn. We performed a univariate and logistic regression analysis.

**Results**: We included 542 patients: 87.6% women, mean age 47.6 ± 11.7 years. A 89.3% had chronic migraine and 10.8% had high frequency episodic migraine. The mean reduction in frequency after treatment was 13.4±8.2 headache days/month. At baseline, a 91.3% took other preventives and during treatment with OnabotulinumtoxinA a 58.6% withdrew at least one drug, 25.8% stopped completely all oral preventive drugs. Factors associated with withdrawal were: being male, having >50% response in frequency and intensity, the number of infiltrations and a shorter chronification period until the first OnabotulinumtoxinA administration (p <0.05). The multivariate analysis showed that a better response in intensity (OR:1.8 [1.4-2.2], p<0.001), a greater number of infiltrations (OR:1.1 [1.0-1.2], p<0.001) and a shorter chronification period (OR:0.994 [0.992-0.997], p<0.001) were predictors of withdrawal. The ROC curve, showed that 6 OnabotulinumtoxinA cycles was the cut-off point that better predicted oral preventive medication withdrawal (p <0.001).

**Conclusions**: Treatment with OnabotulinumtoxinA reduces the use of other preventive medications for migraine. The highest probability of withdrawal occurs after 6 cycles of treatment.

### P115 Onset of effect of onabotulinumtoxinA for chronic migraine treatment: analysis of PREEMPT data

#### David W. Dodick^1*^ Stephen D. Silberstein^2^ Richard B. Lipton^3^ Ronald E. DeGryse^4^ Aubrey Manack Adams^4^ Hans-Christoph Diener^5^

##### ^1^Department of Neurology, Mayo Clinic, Phoenix, AZ, 85054, USA; ^2^Department of Neurology, Jefferson Headache Center, Thomas Jefferson University, Philadelphia, PA, 19107, USA; ^3^Department of Neurology, Albert Einstein College of Medicine, Bronx, NY, 10461, USA; ^4^Allergan plc, Irvine, CA, 92612, USA; ^5^Department of Neurology, University of Duisbury-Essen, Essen, 45122, Germany

**Correspondence:** David W. Dodick (Dodick.David@mayo.edu)

**Background**: The PREEMPT trials (ClinicalTrials.gov, NCT00156910 and NCT00168428) demonstrated the efficacy and safety of onabotulinumtoxinA (onabotA) for the prevention of headache in adults with chronic migraine (CM). This analysis assessed the time to onset of treatment effects of onabotA relative to placebo (PBO) on reduction of headache days and migraine/probable migraine days per week from baseline.

**Methods**: Each PREEMPT trial included a 24-wk, double-blind, PBO-controlled phase followed by a 32-wk open-label phase. Patients were randomized to injections of onabotA (155 U to 195 U) or PBO every 12 wks for 2 cycles; followed by 3 open-label cycles of onabotA (155 U to 195 U). The primary efficacy variable for the pooled analysis was mean change from baseline in frequency of headache days per 28 days (primary endpoint, wk 24). Additional analyses included change in headache days and migraine/probable migraine days per wk vs baseline. Pooled analyses from the double-blind and open-label phases are presented. The studies were approved at each site by an institutional review board.

**Results:** 1384 adults were randomized to onabotA (n=688) or PBO (n=696). Baseline values (as assessed during wk 4 of the baseline period) were similar in both groups for mean (SD) headache days/wk (onabotA: 4.8 [1.6] days; PBO: 4.8 [1.6] days, *P*=0.70) and for migraine/probable migraine days per wk (onabotA: 4.6 [1.7]; PBO: 4.6 [1.7] days, *P*=0.72). Pooled analyses demonstrated a significant mean decrease from baseline in frequency of headache days per 28 days, favoring onabotA over PBO at the wk 24 primary endpoint (−8.4 vs −6.6; *P*<0.001) and at the end of the open label period (onabotA/onabotA: –11.7 vs PBO/onabotA: −10.8; *P*=0.02). One wk after the first treatment, onabotA reduced mean (SD) headache days by –0.9 (2.2) vs PBO (–0.7 [2.1]; *P*=0.046) and migraine/probable migraine days by –1.0 (2.4) vs PBO (0.7 [2.2]; *P*=0.031); the effect persisted from wk 3 of the first treatment cycle for both measures. OnabotA resulted in continued reduction in headache days (Fig. 1A) and migraine/probable migraine days (Fig. 1B) over 5 treatment cycles.

**Conclusions**: As early as wk 1 after the first treatment, onabotA treatment significantly reduced headache days/wk and migraine/probable migraine days/wk. This improvement persisted from wk 3 of the first treatment cycle compared with PBO. Treatment with onabotA resulted in a persistent and progressive reduction in headache days over the course of the 56-wk PREEMPT trials, indicating that peak benefit may require multiple treatments.

**Fig. 1 (abstract P115).**
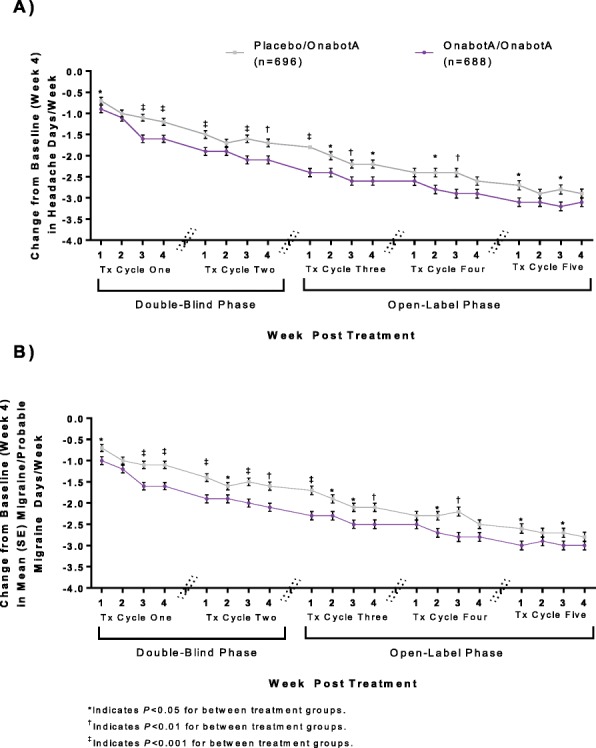
Change from baseline in A) headache days/week and B) migraine/probable migraine days/week after treatment with onabotulinumtoxinA or placebo/onabotulinumtoxinA

### P116 New daily persistent headache: A systematic review on an enigmatic disorder

#### Nooshin Yamani^1, 2^, Jes Olesen^1^

##### ^1^Danish Headache Centre and Department of Neurology, University of Copenhagen, Rigshospitalet Glostrup, Copenhagen, Denmark; ^2^ Headache Department, Iranian Center of Neurological Research, Neuroscience Institute, Tehran University of Medical Sciences, Tehran, Iran

###### **Correspondence:** Nooshin Yamani (Jes.olesen@regionh.dk)

***Background***: New daily persistent headache (NDPH) is a recognized type of primary headache disorders. Although its prevalence is rare, NDPH is important because of its persistency, therapeutic refractoriness, associated disability and psychiatric comorbidity.

***Objectives***: To provide a structured systematic review to increase the understanding of new daily persistent headache.

***Methods***: PubMed and EMBASE search was performed for papers published or e-published before February 2018 using the terms “new daily persistent headache” and “NDPH”. We also searched for other useful sources in the reference lists of the selected articles.

***Results***: New daily persistent headache is heterogeneous in presentation and may resemble migraine or tension-type headache or both. Prevalence rate of NDPH has been estimated at 0.03% to 0.1% in the general population and may be higher in children and adolescents than in adults.

The underlying pathophysiology of NDPH is unknown, but since it was demonstrated that a number of factors such as infection, stressful event or extracranial surgery might precipitate NDPH, associations have been made with the role of pro-inflammatory cytokines and cervicogenic problems in its development.

There is no well-defined strategy for treatment of NDPH based on clinical evidence and it seems best to treat NDPH based upon the prominent headache phenotype. A few treatment regimens have been used in the literature with mixed results. However, even aggressive treatment is ineffective or only partially effective.

***Conclusion***: All aspects of NDPH discussed in this review need further study. NDPH remains poorly understood but very burdensome for the individual without any efficient therapy.

**Keywords**: New daily persistent headache, NDPH, Primary headache disorders, chronic daily headache

**Table 1 (abstract P116).** Prevalence, age , sex and race distribution of NDPH in different studies.

Reference	Location	Definition criteria	Population surveyed	NDPH prevalence	Female	Male	F:M ratio	Age of onset	Race
Castillo-1995	Spain	S-L	1883 general population 22-73 yr	0.1%GP					
Li & Rozen-2002	USA	S-L	56 NDPH cases		40(71%)	16(29%)	2.5	12-78	Caucasian:87% Black:11% Hispanic:2%
Bigal-2004	USA	S-L	170 adolescent 13-17 yr with CDH 638 adult with CDH	21% CDH 10.8% CDH					
Takase- 2004	Japan	ICHD 2	30 NDPH cases of 1760 CDH	1.7% CDH	13(43%)	17(57%)	0.8	13-73	
Meineri-2004	Italy	ICHD2,S-L	18 NDPH cases of 265 CDH	6.7% CDH	11(61%)	7(39%)	1.6	13-76	
Mack-2004	USA	M-ICHD2	175 children <18 yr with CDH	23% CDH	27(67.5%)	13(32.5%)	2.1		
Kung 2006	USA	M-ICHD 2	306 children and adolescents 6-18 yr in a tertiary headache center	28% CDH	34(64.2%)	19(35.8%)	1.7		
Grande 2009	Norway	ICHD 2	30000 general population 30-44 yr	0.03% GP					
Robbins 2010	USA	M-ICHD2	71 NDPH		51(72%)	20(28%)	2.5	8-76	Cacausian:80.3% Black:5.6% Hispanic:9.9%
Prakash 2012	India	M-ICHD2	63 NDPH		36(57%)	27(43%)	1.3	18-68	
Rozen 2016	USA	ICHD-3β	97 NDPH		65(67%)	32(33%)	2	Mean: F:32.4 M:35.8	Cacausian:98% Black:1% Hispanic:1%
Uniyal 2017	India	ICHD-3β	55 NDPH		45.5%	54.5%	0.8	Mean:28.4	

*S-L* Silberstein-Lipton criteria, *ICHD* International classification of headache disorders, *M-ICHD2* modified ICHD2(NDPH according to the criteria A and B of the ICHD-2 regardless of the presence of migraine features.), *GP* general population, *CDH* chronic daily headache

**Table 2 (abstract P116).** ICHD-3 diagnostic criteria for NDPH

A. Persistent headache fulfilling criteria B and C B. Distinct and clearly-remembered onset, with pain becoming continuous and unremitting within 24 hours C. Present for >3 months D. Not better accounted for by another ICHD-3 diagnosis	

**Table 3 (abstract P116).** Clinical characteristics of patients with NDPH in various published studies.

Study	Vanst 1986	Rozen 2002	Takase 2004	Meineri 2004	Kung 2008	Robins 2010	Peng 2011	Prakash 2012	Uniyal 2017
Definition criteria		S-L	ICHD-2	ICHD-2	M-ICHD2	M-ICHD2	M-ICHD2	M-ICHD2	ICHD3β
Number of NDPH cases	45	56	30	18	53	71	92	63	55
Mean age		F:3rd decade M:5th decade	35	F:3rd decade M:4th decade	14.2 (study of pediatrics)		5^th^ decade	36.8	28.24
Female:male	1.4:1	2.5:1	0.8:1	1.6:1	1.7:1		1.3:1	1.3:1	0.8:1
Recalling time of onset	-		-						
Exact date:		80%		100%	-	42%	-	33%	20%
Month:						83 %			80%
Past history of prior headache	none	38%	7%	33%	-	25.4%	32%	54%	38.2%
Past history of episodic tension-type headache	none	19%	-	-	-	18.3%	20%	29%	29.1%
Past history of episodic migraine headache	none	2%	-	-	-	7%	12%	25%	9.1%
Location of headache	-				-				
Bilateral:		64%	86%	100%		88.7%	46.7%	83%	81.5%
Pain characteristics					-				
Throbbing	28%	55%	27%	61%		45.1%	41%	51%	41.8%
Pressing/tightening	72%	54%	90%	100%				100%	56.4%
Pain severity									
Mild:		18%	-	28%		16.9%		-	Mean Headache Score:7.5
Mod:		61%		72%		57.7%	87%		
Severe:		21%		0	10.8 d/m	23.9%			
Aggregation by physical activity	-	-	-	-	-	46.5%	57%	27%	16.4%
Associated features:									
Nausea:	55%	68%	33%	50%	39%	47.9%	35%	49%	56.4%
Vomiting:	12%	23%	-	-	-	12.7%	7%	5%	20%
Photophobia:	34%	66%	3%	27%	69%	45.1%	48%	33%	45.5%
Phonophobia:	37%	61%	-	17%	63%	40.8%	58%	19%	-
Autonomic features:	-	23%	-	-	-	21%	-	14%	-
Psychiatric comorbidity:	-	-	-	-	-	(self-reported)		(Self-reported)	
Depression:						35.2%		19%	89.1%
Anxiety:						33.8%	60.8%	16%	92.7%
Family history of headache	-	29%	-	33%		49%	47.5%	-	

**Table 4 (abstract P116).** Patients reported NDPH triggers in various published studies

Reference	Number of NDPH patients	No Triggering factor	Infection or flu-like illness	Stressful life event	Trauma /surgery	other
Mack 2004, USA	40 (pediatric NDPH)	5(12%)	17(43%)		13(33%)	5(12%) idiopathic intracranial hypertension, high altitude climbing
Takase 2004, Japan	30	24(80%)	-	6(20%)	-	
Robins2010, USA	71	38(53.5%)	10(14.1%)	7(9.9%)		6(8%) menarche, SSRI withdrawal, HPV vaccination
Peng2011, Taiwan	92	65(71%)	3(3%)	24(26%)		
Parkash2012 , India	63	29(46%)	18(29%)	5(8%)	10(16%)	9(14%) postpartum, medication overuse
Rozen2016, USA	97 Female:65 Male:34	51(53%) Female: 52% Male: 53% Mean age:30.4	21(22%) Female: 22% Male: 22% Mean age:31.8	9(9%) Female: 11% Male: 6% Mean age:28.1	9(9%) Female:9% Male:9% Mean age:63.3	7(7%) syncope, hormone, toxin and medication, cervical massage
Uniyal 2017, India	55	35(63.5%)	10(18%)	5(9.1%)	5(9.1%)	

**Table 5 (abstract P116).** Secondary mimics of NDPH

• Low or raised CSF pressure(Spontaneous CSF leak, Idiopathic intracranial hypertension, Intracranial mass lesion ) • Cerebral venous thrombosis • Cranial artery dissection • Cranial arthritis • Posttraumatic headache (subarachnoid hemorrhage, subdural hematoma,…) • Meningitis • Sphenoid sinusitis • Contact-point headache(caused by contact of intranasal structures)	

### P117 Clinical description of 135 patients with typical aura without headache and scale proposal for diagnosis in a first episode

#### Robert Belvís, Marina Guasch, Paula Marrero, Noemí Morollón, Azahara Aceituno, Carles Roig

##### Neurology department Hospital de la Santa Creu i Sant Pau, Neurology department Hospital Universitario Dexeus. Barcelona, Spain

###### **Correspondence:** Robert Belvís (rbelvis@santpau.cat)

**Introduction**

Typical aura without headache (TAWH) is a sort of migraine with aura in which aura is not followed by headache within 60 minutes. Approximately, 3-6% of patients with migraine suffer TAWH without developing any kind of pain. We clinically analyse 135 patients and propose a diagnostic scale, Migraine Aura Barcelona Scale (MABs), to diagnose it in the first episode**.**

**Patients**

We included patients with two or more episodes of TAWH according to ICHD3 criteria with normal brain MRI. We recorded prospectively its clinical features, duration and variability. With univariant statistical analysis we compare TAWH characteristics in younger and older patients (> 50 years old).

**Results**

Between 2010-2017, 352 patients with transitory visual disorders were visited. 135 of them fulfilled TAWH criteria (1.8% of migraine visits), average onset age: 18 years old. They presented 6850 auras during follow-up (average: 52 auras / patient): 51 scintillating scotoma, 31 photopsia, 22 fortification spectrum, 20 blurred vision, 42 other (non-negative phenomena: 88.1%), intraindividual variability: 22.2%, average duration: 22.05 minutes, progressive: 65.9% and black-and-white: 65.1%. In older patients, TAWH were shorter (p=0.002), less progressive (p=0.035) and they had more negative phenomena (p=0.05). 94% of patients score >3 points in MABs, which includes: age <50 years old (2-0 points), migraine history (1-0), vascular risk factors (0-1), non-negative phenomena (2-0), progressive (2-0), duration 5-60 minutes (2-0).

**Conclusions**

TAWH are less frequent in our series comparing to literature. They are more common in younger patients and have lower scores in the MAB scale when they appear in older population.

### P118 Onabotulinumtoxin A for Chronic Migraine with Medication Overuse: Preliminary Clinical Results and Changes in Catastrophising

#### Licia Grazzi^1^ Eleonora Grignani^2^, Emanuela Sansone^1^, Alberto Raggi^2^, Matilde Leonardi^2^, Domenico D’Amico^1^, Frank Andrasik^3^

##### ^1^Headache and Neuroalgology Unit, Neurological Institute "C. Besta" IRCCS Foundation, Via Celoria 11, 20133, Milan, Italy. ^2^Neurology, Public Health and Disability Unit, Neurological Institute "C. Besta" IRCCS Foundation, Milan, Italy. ^3^Department of Psychology, University of Memphis, TN

###### **Correspondence:** Licia Grazzi (licia.grazzi@istituto-besta.it)

**Introduction:** OnaBotulinumtoxin A is increasingly being shown as useful in treating chronic forms of migraine, as illustrated in the PREEMPT studies. Patients adherence to this treatment is generally high as well. Further, treatment with Onabotulinumtoxin A can also lead to corresponding improvements in various associated psychological symptoms accompanying chronic migraine such as anxiety and depression. Also reveal high levels of disability, lessened quality of life and heightened levels of catastrophising are evident in chronic migraine; the latter of which have not been well studied to date.

**Methods:** sixty patients, (7 male, 53 females), with a mean age of 46.5 ± 9.4 years and a mean age of onset of migraine of 17.3 ± 9.8years, diagnosed as Chronic Migraine with Medication Overuse (according to IHS criteria) were treated by Onabotulinumtoxin A according with the PREEMPT protocol, at the dosage of 195 UI, at the Headache Centre of the Neurological Institute C.Besta in Milan. All patients, first completed a 5-day structured day hospital withdrawal program in order to cease their overuse of symptomatic medications. All patients maintained daily headache diaries for logging key primary headache variables as well as symptomatic medications. They additionally completed secondary measures to track disability (MIDAS Questionnaire) Quality of life (HIT-6) and tendencies to catastrophise about pain (Pain Catastrophising Scale) and allodinia evaluation by ASC questionnaire.

**Results:** Twelve patients, all females, (mean age 44.3 ± 8.1 years, onset of migraine 14.2 ± 6.3 years) have completed the third session of treatment with Onabotulinumtoxin A and have provided post-treatment data. All patients reported changes in clinical indexes recorded from the daily diary (days of headache /month pre 25.6 ± 7.2 at 6months16 ± 10.3; medication/month pre 22.9 ± 8.6 at 6 months 17.4 ± 13.4). For the secondary measures of outcome, reductions were observed for catastrophizing (PCS 29 ± 9.5 at baseline vs 22.2 ± 8.9); and disability (MIDAS 75.2 ± 49.1 vs 52.4 ± 56.1); with accompanying improvements for quality of life (HIT-6: 65.6 ± 4.3 vs 60.5 ± 8.6). Although the percentage reductions for the PCS and MIDAS were sizeable, they were not statistically significant. This is likely due to the small available sample sizes for this preliminary report and the brief follow up period.

**Conclusions:** The dosage of 195 UI is well tolerated and effective as in preceding reports. The slight improvements noted for secondary measures, although not significant, are encouraging and await further replication

### P119 Mindfulness meditation for migraine in pediatric population: a pilot study

#### Licia Grazzi^1^Emanuela Sansone^1^, Raggi Alberto^2^, Eleonora Grignani^2^, Matilde Leonardi^2^, Chiara Scaratti^2^, Frank Andrasik^3^

##### ^1^Headache and Neuroalgology Unit, Neurological Institute "C. Besta" IRCCS Foundation, Via Celoria 11, 20133, Milan, Italy; ^2^Neurology, Public Health and Disability Unit, Neurological Institute "C. Besta" IRCCS Foundation, Milan, Italy; ^3^Department of Psychology, University of Memphis, TN

###### **Correspondence:** Licia Grazzi (licia.grazzi@istituto-besta.it)

**Introduction:** Chronic Migraine (CM) is a highly disabling condition characterized by at least 15 days with headache per month often associated with overuse of symptomatic drugs. It impairs patients’ emotional, social, and work/school functioning. CM prevalence is around 2% among adolescents. Also migraine at high frequency can be a problematic condition often predisposing patients to chronic forms and overuse. Many pharmacological prophylactic therapies are effective in 30-50% of cases: for this reason, the effectiveness of non-pharmacological therapies is a matter of research interest. This is of particular importance in pediatric populations, where the use of pharmacological prophylaxis should not be encouraged. Mindfulness preliminarily demonstrated a clinical advantage in in chronic pain conditions pediatric populations although few studies are available; no clinical experience in adolescent migraine. Aims of this study were to examine the feasibility, acceptability, and effectiveness of a Mindfulness-based intervention in adolescents, aged 12-17, with CM or high-frequency migraine.

**Methods:** The intervention consists of seven weekly sessions of guided mindfulness-based meditation, 45 minutes each held at Neurological Institute Besta of Milan. The group-based session aimed to teach and make direct practice with skills intended to enhance sustained nonjudgmental present moment awareness. The techniques include guided body scan, tension release, mindfulness meditation, breath-focused imagery, guided imagery and decentralization of thoughts. Participants were asked to practice these techniques at home for at least 10 min per day. The variables evaluated were: headache frequency, medication intake/month (from patients diaries); disability levels (PedMIDAS); anxiety (State-Trait Anxiety Inventory; STAI Y1-Y2); depression (Kovacs’s Children's Depression Inventory: CDI); catastrophizing attitude (Pain Catastrophizing Scale: PCS).

**Results:** Thirteen patients were enrolled. Eleven patients completed all sessions: they were regularly attended and the same was for the 10-minute home practice, which we interpreted as a marker of good compliance to the treatment and feasibility of it. The number of headache days decreased (11.1±4.25 vs 8.6±6.08) and also the medication intake (9,88±6.21 vs 7± 6.34) at the first follow up 3 months after treatment.

**Conclusion:** the overall clinical impression is that the treatment was accepted by all participants. patients that completed the seven sessions did not report any difficulty in dealing with the practice and no side effect was detected, which provides us positive feedback on the first two aims of the protocol, namely feasibility and acceptability of a Mindfulness-bases intervention for adolescents with chronic or high-frequency migraine.

### P120 Prophylactic effect of ultramicronized N-Palmitoyl Ethanol Amide (PEA) on pediatric migraine: a preliminary study

#### Giorgia Sforza^1,2*^, Laura Papetti^1^, Samuela Tarantino^1^, Barbara Battan^1^, Michela Ada Noris Ferilli ^1,3^, Romina Moavero^1,2^, Federico Vigevano^1^, Massimiliano Valeriani^1^

##### ^1^ Headache Center, Child Neurology Unit, Bambino Gesu’ Children’s Hospital, Rome, Italy; ^2^ Child Neurology and Psychiatry Unit, Tor Vergata University of Rome, Italy; ^3^Institute of Neurology, Catholic University of Sacred Heart, Rome, Italy

###### **Correspondence:** Giorgia Sforza (sforzagiorgia@gmail.com)

**BACKGROUND:**

Primary headache disorders are recognized as one of the most prevalent health problems worldwide. The prevalence of headaches during childhood have been investigated across pediatric age groups with varying estimates ranging from 3 % in school-age children to 20 % in adolescents [1,2].

Traditionally, pediatric migraine treatment includes both prophylactic therapy, aimed at reducing the severity and frequency of attacks, and acute therapy to stop the attack.

Although amitriptyline, topiramate, flunarizine and valproic acid have the most data on their use for prophylaxis in children, a serious lack of controlled studies on the pharmacological treatment of pediatric migraine still remains. Consequently, there is an urgent need for further studies in this population [3,4].

Nutraceuticals, and other supplements, may be an alternative option in treating migraine and may be offered to parents who are reluctant to start their child on a daily medication.

Palmitoyl ethanolamide (PEA) is an amide of endogenous fatty acids widely distributed in different tissues, including nervous tissues. It is emerging as a new therapeutic approach in pain and inflammatory conditions and has been reported as effective in animal models of chronic pain and inflammation, as well as in numerous clinical studies on various paininful diseases [5,6,7]. However, to date no studies have been conducted to evaluate the role of PEA in the management of migraine without aura in pediatric patients.

**OBJECTIVE:**

The aim of this preliminary open-label study was to evaluate the efficacy of chronic ultramicronized PEA (um-PEA) administration in terms of reducing the frequency and severity of migraine attacks in pediatric patients.

**METHODS:**

The study sample includes 5 patients (1 male and 4 females), ranging between 6,7 and 12,1 years of age (mean 9.4 years). They had a diagnosis of migraine according to the ICHD-3 criteria and received umPEA (600 mg/day). They were re-evaluated at 60 days after treatment onset.

**RESULTS:**

After 60 days of treatment with um-PEA, headache frequency was reduced by >50 % per month in 4 out of 5 patients and pain intensity was reduced from moderate to mild in 4 out of 5 patients.

**CONCLUSIONS:**

Our preliminary data show that um-PEA administered chronically for 60 days reduces pain intensity and the number of attacks per month in a small sample of pediatric patients. Although the small number of patients does not allow us to consider these initial results as definitely reliable, they encourage us to expand the sample.

**References:**

1)World Health Organization, Lifting the Burden. Atlas of Headache Disorders and Resources in the World 2011. A collaborative project of World Health Organization and Lifting the Burden.

Geneva: World Health Organization; 2011

2)Lewis D. Pediatric migraine. Neurol Clin. 2009;27:481–501.

3)Lewis DW, Diamond S, Scott D, Jones V. Prophylactic treatment of pediatric migraine. Headache. 2004;44(3):230–7.

4)Papetti L, Spalice A, Nicita F, Paolino MC, Castaldo R, Iannetti P, Villa MP, Parisi P. Migraine treatment in the developmental age: guidelines update. J Headache Pain. 2010;11(3):267–76.

5)Costa, B., Comelli, F., Bettoni, I., Colleoni, M., Giagnoni, G., 2008. The endogenous fatty acid amide, palmitoylethanolamide, has anti-allodynic and anti-hyperalgesic effects in a murine model of neuropathic pain: involvement of CB(1), TRPV1 and PPARgamma receptors and neurotrophic factors. Pain 139, 541-550.

6)Chirchiglia, D., Della Torre, A., Signorelli, F., Volpentesta, G., Guzzi, G., Stroscio, C.A., Deodato, F., Gabriele, D., Lavano, A., 2016. Administration of palmitoylethanolamide in combination with topiramate in the preventive treatment of nummular headache. International medical case reports journal 9, 193-195.

7)Gabrielsson, L., Mattsson, S., Fowler, C.J., 2016. Palmitoylethanolamide for the treatment of pain: pharmacokinetics, safety and efficacy. British journal of clinical pharmacology 82, 932-942.

### P121 Cerebral Hemodynamics, Patent foramen ovale and White Matter Hyperintensities in patients Migraine with aura, young stroke patients and controls

#### C Altamura^1^, CA Mallio^2^, G Lo Vullo^2^, N Brunelli^1^, M Paolucci^1^, A Cascio Rizzo^1^, G Cecchi^1^, CC Quattrocchi^2^, F Vernieri^1^

##### ^1^Headache and Neurosonology Unit, Neurology Unit, Università Campus Bio-Medico di Roma, Rome, Italy; ^2^Radiology Unit, Università Campus Bio-Medico di Roma, Rome, Italy

###### **Correspondence:** C Altamura (c.altamura@unicampus.it)

**OBJECTIVES:** Patients affected by Migraine Aura (MA) were consistently reported to present an increase in stroke risk, while if they display a higher White Matter hyperintensities (WMH) load compared with general population is still the matter of debate. Patent foramen ovale (PFO) and an impaired Cerebral Hemodynamics could at least in part account for this increased risk [1]. Our study aimed at evaluating the relationship among cerebral hemodynamics, patent foramen ovale and white matter hyperintensities in patients affected by MA, young patients with cryptogenetic stroke or motor transient ischemic attack and controls.

**METHODS** We consecutively enrolled 20 MA patients (mean age 35,8 years), 19 young (younger than 60 years) patients with cryptogenetic stroke/motor TIA (46 years) and 10 controls age-matched with MA patients (38,6 years) among subjects referred to our neurosonology lab to undergo TCD bubble test to detect PFO presence. To assess cerebral hemodynamics Breath Holding Index (BHI) [the percent increase of blood mean cerebral flow velocity after 30-second apnea] was simultaneously assessed for middle (MCA) and posterior (PCA) cerebral arteries in all subjects. Vascular risk factors were collected by medical history interviews. Brain WMH volume were obtained on axial FLAIR images using a function of OsiriX MD v.2.6 software.[2] Values of WMH area were collected separately according to the vascular territories of the MCA and PCA and eventually summed.

**RESULTS** Stroke/TIA patients presented older age (Kruskal-Wallis test p=.004), higher prevalence in PFO (95%) and dyslipidemia (Chi-squared p<.001), and higher WMH load in the MCA and PCA territories with respect to the other groups. MA patients presented higher BHI compared with the other groups in the MCA (Kruskal-Wallis test p=.013) as well as in the PCA (p=.003), and higher PFO prevalence (59%) (Chi-squared p=.011) than controls (23%). WMH load did not differ in MA patients compared with controls, while it resulted higher in Stroke/TIA patients as total load (Kruskal-Wallis test p=.038) as well as regional MCA and PCA load. Taking into account all enrolled subjects, those with PFO displayed higher WMH in the PCA territory (Mann Whitney p=.038). BHI was not related to WMH load.

**DISCUSSION** Our study supports the hypothesis that WM is preserved in MA patients. This finding can be explained by a more reactive cerebral hemodynamics (BHI) that may counteract the risk related to the higher prevalence of PFO. In our population, PFO is related to an increased WM vulnerability in the posterior circulation.

**REFERENCE**

1. Mahmoud AN, Mentias A, Elgendy AY, et al (2018) Migraine and the risk of cardiovascular and cerebrovascular events: a meta-analysis of 16 cohort studies including 1 152 407 subjects. BMJ Open 8:e020498 . doi: 10.1136/bmjopen-2017-020498

2. Altamura C, Scrascia F, Quattrocchi CC, et al (2016) Regional MRI diffusion, white-matter hyperintensities, and cognitive function in Alzheimer’s disease and vascular dementia. J Clin Neurol 12: . doi: 10.3988/jcn.2016.12.2.201

### P122 Prediction of the pterygopalatine ganglion localization in CT images

#### Joan Crespi^1,2,3^, Daniel Bratbak^2,4^, David Dodick^2,5^, Kent Are Jamtøy^2,6^, Irina Aschehoug^2^, Manjit Matharu^7^, Erling Tronvik^1,2,3^

##### ^1^Department of Neurology, St Olav’s University Hospital, Trondheim, Norway; ^2^NTNU (University of Science and Technology), Department of Neuromedicine and Movement Science, Trondheim, Norway; ^3^Norwegian Advisory Unit on Headaches, Trondheim, Norway; ^4^Department of Neurosurgery, St Olav’s University Hospital, Trondheim, Norway; ^5^Mayo Clinic, Arizona, USA; ^6^Department of maxillofacial surgery, St Olav’s University Hospital, Trondheim, Norway; ^7^National Hospital of Neurology and Neurosurgery, London, UK

###### **Correspondence:** Joan Crespi (joan.crespi@ntnu.no)

**Background**

The pterygopalatine ganglion (PPG) is a target for several headache syndromes. Most of the groups targeting the PPG do not localize it before injection and this might account for some therapeutic failures. The PPG cannot be seen in CT scans but one has to use MRI to localize it. It would be advantageous to be able to predict its localization in CT scans if MRI is not accessible or contraindicated and for those using fluoroscopy and CT-guided injections.

**Methods**

We localized the PPG in 21 Caucasian patients (21 right and 17 left ganglia; total 38) in 3T-MR images subsequently fused with CT scans. Two approaches were used to predict the position of the PPG. In the first, the coordinates of the opening of the Vidian canal (VC) and the distances to the PPG were measured in 38 sides. The average distance between VC and PPG was used to predict in CT images the first estimated localization of the PPG (PPG*). In the second approach, the coordinates (in MRIs) of the closest point to the PPG in the pterygopalatine bone, were registered (S-point). The average distance from the S-point to the PPG was calculated. This average distance, was used to calculate the coordinates of the second prediction of the PPG in CT images (PPG**) from the S-point. Finally, the distance between the PPG, as seen in MRIs, and predicted PPG*/PPG** was calculated.

**Results**

The average distance between PPG, as located in MRI-images, and PPG* (estimated in CT images, calculated from the average distance from the VC) was 1.82 mm (SD: 0.83). The average distance between PPG, as located in MRI-images, and PPG** (estimated in CT-images, calculated from the average distance from the closest point on the sphenoidal bone) was 2.09 mm (SD: 0.99).

**Conclusions**

The localization of the PPG can be accurately predicted in CT images using bony landmarks in these sample of patients.

**Key words:** pterygopalatine ganglion, sphenopalatine ganglion, headache, CT scan and MRIs.

### P123 Anxiety, depression and alexithymia in young migraine patients: which relationship with body weight?

#### Samuela Tarantino^1^, Alessandra di Stefano^2^, Laura Papetti^1^, Barbara Battan^1^, Giorgia Sfronza^3^, Federico Vigevano^1^, Simonetta Gentile^2^ and Massimiliano Valeriani^1,4^

##### ^1^Headache Center, Division of Neurology, ^2^Unit of Clinical Psychology, Ospedale Pediatrico Bambino Gesù, IRCCS, Piazza Sant’Onofrio 4, Rome, Italy; ^3^Child Neurology and Psychiatry Unit, Tor Vergata University of Rome; ^4^Center for Sensory-Motor Interaction, alborg University, Aalborg, Denmark

###### **Correspondence:** Samuela Tarantino (samuela.tarantino@opbg.net)

**Background** A growing body of literature explored the relationship between migraine and body weight. While several studies analyzed common pathophysiological mechanisms implicated in migraine and feeding regulation, data on the role of psychological factors are sparse, especially in pediatric age. Aims of the present study were to study 1) the prevalence of overweight in migraineurs children/adolescents; 2) the possible relationship between frequency of migraine and overweight; 3) the role of psychological symptoms (anxiety/depression) and emotional processing /regulating (alexithymia) on Body mass Index (BMI) in migraine patients.

**Methods** Patients were identified though a systematic review of clinical records of patients referred to our headache center from 2014 to 2017. We included a total of 122 migraineurs (m.a.12.8 ± 2.5 years; 55 M, 67 F) which were divided in two groups: Group-1, patients who underwent a screening for anxiety and depression (m.a 11.6 ± 2,6 years; M 24, F 43) and Group-2, those who were evaluated for alexithymia symptoms (m.a. 14.2 ± 1.6 years; M 31, F 24). Among the two different groups patients were divided according the BMI percentiles in “Normal weight”, “Overweight” and “Obese” (collapsed in the “Overweight” group). The psychological screening was assessed by SAFA (Anxiety-Depression scales) and TAS-20 questionnaires. The attack frequency was divided in high frequency (from weekly to daily episodes), and low frequency (≤ 3 episodes per month).

**Results** Sixty-four patients (60.7%) were classified as normal weight, 16.4% were obese and 22.9% overweight. The weight (normal or overweight) did not correlate with migraine frequency in none of the groups (respectively: Group-1: χ(2) = 0.998, p=0.317; Group-2: χ(2) = 0.151; p= 0.697). In Group-1, we found a significant higher score in “Separation anxiety” subscale (p=0.03) among overweight patients. No difference was found in SAFA-D subscales (SAFA-D Tot= 0.14) between normal and overweight groups. Analyzing Group-2, our data did not show a significant difference in alexithymia levels (TAS-20 Tot= 0.814) according the body weight. SAFA-A, SAFA-D and TAS-20 did not show any significant correlation with BMI (p>0.05).

**Conclusions** In our study, there is not a correlation between body weight and the frequency of migraine. Our results, however, suggest that overweight migraineurs patients are more prone to “separation anxiety”. In these children, food may alleviate loneliness and separation worries; on the other hand, we can suppose that overweight migraineurs patients are over-protected and pampered leading to a separation anxiety from their parents whenever a stressful situation may arise.

### P124 Health care resource use, sick-leaves and comorbidities among migraine patients in occupational healthcare

#### Minna A. Korolainen^1^, Samu Kurki^2^, Iiro Toppila^3^, Mariann Lassenius^3^, Timo Purmonen^1^ and Markku Nissilä^2^

##### ^1^Novartis Finland Oy, Espoo, Finland; ^2^Terveystalo Biobank Finland, Turku, Finland; ^3^Medaffcon Oy, Espoo, Finland

###### **Correspondence:** Minna A. Korolainen (Minna.Korolainen@novartis.com)

***Introduction and objective***: Migraine was estimated to be the leading cause of disability globally among population under 50 years of age [1]. The objective of this study was to assess how the burden of migraine impacts a Finnish occupational health care patient cohort.

***Methods:*** The study was based on a retrospective analysis of electronic medical records, collected as a part of routine clinical practice by a private health care provider Terveystalo consisting of over 150 clinics all over Finland. We gathered the epidemiological parameters, comorbidities, healthcare resource use, medication use, and sick-leaves related to migraine among 369’383 consented patients. The migraine cohorts were defined using diagnosis code G43, prescription with pre-defined ATC-codes for prophylaxis (C07-09, N03, N06, G02-03, M03AX01) and acute treatment (M01-N02BE, N02CA, N02CC, A03FA, A04AA01, H02AB) as well as “migraine” written as a free text in the electronic prescription. The inclusion period to the study and for the follow-up of patients started 1^st^ January 2012 and ended 31^st^ December 2017.

***Results:*** Of 17’623 identified migraine patients, 8899 had no prescribed treatment, 6525 had acute migraine treatment and 2199 fulfilled the defined criteria for the prophylaxis cohort. Patients in the prophylactic cohort visited the healthcare organization on average 18.4 times and were prescribed 22.5 days of sick-leave per patient-year. Moreover, numbers of visits and sick-leave days increased concomitantly with increasing prophylactic treatment lines, and were 30% higher than in migraine sufferers without prophylactic treatment. Patients with migraine receiving prophylaxis had 30-40% more concomitant diagnoses of depression (F32) and anxiety (F41) compared to those with acute or no prescribed treatment.

***Conclusions:*** This retrospective study using data from routine clinical practice showed that migraine was associated with substantial morbidity seen as an increase in migraine-related visits, sick-leaves and comorbidities. The disease burden was particularly pronounced in patients on prophylaxis compared to those with acute or no prescribed treatment for migraine.

- **References**:
- 1. Steiner et al. Migraine is first cause of disability in under 50s: will health politicians now take notice? J Headache Pain. 2018;19:17.

### P125 The Number of Active Trigger Points is Associated with Pain Features, Depression and Anxiety in Frequent Episodic, but not Chronic, Tension Type Headache

#### Stella Fuensalida-Novo^**1**^, María Palacios-Ceña^**1,2**^, Matteo Castaldo^**1**^, Kelun Wang^**1**^, Lars Arendt-Nielsen^**1**^, César Fernández-de-las-Peñas^**1,2**^

##### ^1^SMI, School of Medicine, Aalborg University, Aalborg, DENMARK; ^2^Departamento de Fisioterapia, Terapia Ocupacional, Rehabilitación y Medicina Física. Universidad Rey Juan Carlos, Alcorcón, Madrid, SPAIN

###### **Correspondence:** César Fernández-de-las-Peñas **(** cesar.fernandez@urjc.es)

**Background:** Tension type headache (TTH) is a headache condition where the referred pain elicited by active trigger points (TrPs) in the head, neck and shoulder musculature reproduces the pain features. The association between active TrPs with headache pain parameters and mood disorders has not been properly investigated in previous studies.

**Objective:** The aim of the current study was to investigate the association between the number of active and latent TrPs with headache pain features (intensity, frequency and duration) and mood disorders (anxiety and depression) in individuals with TTH grouped by the frequency of headache (frequent episodic-FETTH, chronic-CTTH)

**Methods:** Patients with TTH diagnosed by experienced neurologists according to the International Headache Classification (ICHD-III) were included. All participants read and signed a consent form prior to their participation Exclusion criteria included other primary headaches, medication overuse headache, previous whiplash or fibromyalgia. TrPs (active and latent) were bilaterally explored in the masseter, temporalis, trapezius, splenius capitis, sternocleidomastoid and suboccipital muscles by experienced physical therapists. Headache pain features. i.e., intensity, frequency and duration, were collected with a 4-weeks diary. The Hospital Anxiety and Depression Scale was used to assess the levels of anxiety (HADS-A) and depressive (HADS-D) symptoms. Spearman correlation coefficients (r_s_) were conducted to further determine the correlation between TrPs, headache pain features, depression and anxiety. The study design was approved by local Ethics Committee (URJC 23/2014, HUFA 14/104, Aalborg N20140063, CESU 5/2015).

**Results:** Two hundred and three patients (111 FETTH, 92 CTTH, age: 44±17 years) participated. Each patient with TTH exhibited 4.5±2.8 active TrPs in the cranio-cervical muscles which elicited referred pain reproduced their headache attack, and a mean of 1.6±2.2 latent TrPs. The number of active TrPs showed moderate positive associations with headache intensity (r_s_: 0.485; P<0.001), depression (r_s_:0.414; P<0.001) and anxiety (r_s_: 0.394; P=0.001) and a weak positive association with headache duration (r_s_: 0.271; P=0.004) in individuals with FETTH, but not with CTTH: the higher the number of active TrPs in the cervical musculature, the higher the headache intensity, the longer the duration of the headache attacks and the higher anxiety and depressive symptoms.

**Conclusions:** The current study supports that the number of active TrPs was associated with higher headache intensity and longer headache duration, but not with the frequency of attacks, in individual with FETTH, but not CTTH. In addition, active TrPs was also associated with mood disorders. These results support that musculoskeletal disorders, such as active TrPs, maybe more relevant in FETTH than in CTTH. Future trials should explore the relevance of proper and early treatment of TrPs in the associations observed in the current study and the prevention of chronification of TTH.

### P126 New daily persistent headache in a pediatric cohort

#### Laura Papetti, Barbara Battan, Romina Moavero, Giorgia Sforza, Samuela Tarantino, Massimiliano Valeriani

##### Headache Center Bambino Gesù Children Hospital, Rome Italy

###### **Correspondence:** Laura Papetti (laura.papetti@opbg.net)

**Introduction**

Primary new daily persistent headache (NDPH) is a rare disorder of children and adults defined by the onset of daily headaches with distinct and clearly-remembered onset, with pain becoming continuous and unremitting within 24 hours and present for >3 months. The pain lacks characteristic features, and may be migraine-like or tension-type-like, or have elements of both. Our aim was to investigate the clinical features of NDPH in a cohort of pediatric patients.

**Methods**

We retrospectively reviewed the charts of patients attending the Headache Centre of Bambino Gesú Children from the last ten years with history of persistent daily headache. The ICHD-III criteria were used for diagnosis. Statistical analysis was conducted by SPPS version 22.0 and χ2 test was used to study possible correlations between: - NDPH and population features (age and sex); - NDPH and headache qualitative features; - NDPH and response to prophylactic therapies.

**Results**

We included 377 patients with CPH (66.4% female, 33.6% male, age between 0 and 18 years). The frequency of NADPH was 13% (49/377). We did not find significant differences between the frequency of NADPH in males (42.9%) and females (57.1%). In relation to age we found that NDAPH is less common in the age group of 7-10 years (p<0.05).

Regarding the features of the pain we did not find significant differences compared to the other forms of chronic headache for the quality of pain (throbbing or gravating), and the presence of photophobia (59.2% vs 60.7%, p>0.05) and phonophobia (63.3% vs 70.1%, p>0.05). However we found a low frequency of nausea and vomiting in the NADPH population (28.6% vs 48.2%, p<0.05).

We found that 75% of patients have an onset of the symptoms in the winter months (November-February), respect the remaining months of the year when the incidence is very low (p<0.05).

Our results show that 29 (30.6%) out of 49 NADPH CPH received a prophylactic therapy. Among them, 26 patients received amitriptyline, 4 patients topiramate, one patient L-5 hydroxytryptophan, and one patient flunarizine. Positive response to therapy (reduction of attacks by at least 50% in a month) was detected in 30.6% of patients, while no outcome data were obtained from 63.3% of cases. Amitriptyline showed the highest efficacy (p<0.05).

**Conclusions**

Our results show that the incidence of NASDH in children with daily headache is 13%. In general, the onset occurs in the winter months and this is probably related to the increase in requests for school activities. Qualitative characteristics as for adults are variable, migrainous or tension type. The most effective drug is amitriptyline, although the number of patients who received other types of drugs is very low Furthermore, the number of patients for whom there is an absence of follow-up data is very high and for this reason the efficacy data are not conclusive.

### P127 Cerebral vascular reactivity in patients with chronic neck pain

#### Nevzat Uzuner^1^, Aycan Guner Ekici^2^, Gulnur Tekgol Uzuner^1^, Sacit Gulec^2^

##### ^1^Department of Algology and Neurology, Eskisehir Osmangazi University, Eskisehir, TUR; ^2^Department of Algology and Anaesthesiology, Eskisehir Osmangazi University, Eskisehir, TUR

###### **Correspondence:** Nevzat Uzuner (nevzatuzuner@gmail.com)

**Background and aims**

The cerebral hypoperfusion with amplified cervical sympathetic activation in patients with chronic neck pain (CNP) has been thought for a long time. However, the cerebral vascular reactivity (CVR) has not shown yet in such patients. We aim to show the CVR of the anterior circulation in patients with CNP.

**Methods**

Fifteen patients with CNP were subsequently entered into the study. Age and gender-matched 15 healthy subjects were served as control. All patients were treated with cervical epidural steroid (CES) injections. Bilateral blood flow velocity (BFv) measurements were done at the middle cerebral artery (MCA) during breath holding (BH) test using transcranial Doppler (TCD) before injections, after injections on the next day, and at two weeks later.

**Results**

No significant difference between BFv increases during BH test on both sides during the pre-injection, post injection and two weeks later were found. However, the reactivity times were significantly longer at the pre-injection (15.7 sec and 15.7 sec; right and left sides, respectively), and at the post-injection (15.8 sec and 15.3 sec) than those of controls (9.8 sec and 10.2 sec) (p<0.05). As a consequence, significantly lower reactivity per sec at the same periods than those of controls (p<0.05) was estimated. Nevertheless, two weeks later all parameters returned to the near normal levels.

**Conclusion**

Our study suggests longer reactivity time and lower reactivity per second was encountered in patients with CNP; tough normal cerebral VR was obtained. Although, after CES treatment reactivity parameters were near normal in all patients with significant pain relief.

### P128 Chronic migraine is much more related to cardiovascular risk factors: a clinic-based study

#### Gulnur Tekgol Uzuner^1^, Osman Ozgur Yalın^2^, Derya Uluduz^3^, Aynur Ozge^4^, Nevzat Uzuner^1^

##### ^1^Department of Neurology and Algology, Eskisehir Osmangazi University, Eskisehir, TUR; ^2^Neurology Clinic, Istanbul Education and Research Hospital, Istanbul, TUR; ^3^Department of Neurology and Algology, Istanbul University, Istanbul, TUR; ^4^Department of Neurology and Algology, Mersin University, Mersin, TUR

###### **Correspondence:** Gulnur Tekgol Uzuner (uzunergulnur@gmail.com)

**Background and purpose:** The relation between migraine and vascular risk factors is one of the curious issue. Reasons of chronification still unknown. Perhaps, age-related risk factors and factors leading to migraine progression will also change. Under these questions, our aim were to investigate whether or not there is a specific association with vascular risk factors between several age groups and different subtypes of migraine and also in their family.

**Methods**: A dataset (the Turkish Headache Database) from four tertiary headache centres in Turkey was used. This database included headache-defining features according to ICHD-II criteria based on face-to face interviews and examination by a headache specialist. Using statistical methods, vascular risk factors in MwoA, MwA and CM were compared with three age groups (under 30 years, 30-50 years and over 50 years) and also in first-degree relatives of the patients. We included 2712 patients with MwoA was 1868 (68.9%), MwA was 246 (9.1%), and CH was 598 (22.1%).

**Results**: This study show that both patients and first-degree relatives are more frequently associated with vascular risk factors in CM than episodic MwA and MwoA. MwoA has less of a relationship to vascular risk factors than MwA and CM.

**Conclusion**: Chronic migraine was associated with vascular risk factors at all ages and first-degree relatives as well. Vascular risk factors should be investigated with greater emphasis on chronic migraine.

### P129 The “pure” Trochlear Migraine : two cases and nosographic considerations

#### Luca Maria Messina^1 4^, Francesca Cardella^2^, Rosaria Roppolo^2^, Francesca Vanadia, Filippo Brighina^3^, Vincenzo Raieli^4^

##### ^1^ Child Neuropsichiatry Department - University of Palermo; ^2^ Pediatric Diabetology Unit, ISMEP - ARNAS Civico Palermo; ^3^ Department of Experimental Biomedicine and Clinical Neurosciences - University of Palermo; ^4^ Child Neuropsychiatry Unit, ISMEP - ARNAS Civico Palermo

###### **Correspondence:** Vincenzo Raieli (vinzi.raielk@inwind.it)

**Background**: Recently “Trochlear Migraine” has been described as the concurrence of strictly unilateral migraine and ipsilateral trochleodynia with migraine relief after successful treatment of trochleodynia [1]. This disorder has been interpreted like similar to “Cluster-tic syndrome” or “seizure-triggered migraine” [1]. However, in our opinion, the term is ambiguously used because it more closely defines the association between a topographical localization (orbital trochlear region) and a clinical syndrome rather than the cooccurence between two distinct disorders. In this work, we describe two pediatric migrainous children with strict trochlear localization of pain , that fits perfectly with the nosographic term.

**Case reports**:

1° case: A 12-year old male, affected by type 1 diabetes, was admitted to the Pediatric Hospital on April 2017 for severe left infraorbital pain, nausea and vomiting (five episodes in same day). He reported history of episodic headache from more than 1 year, 1-2 attacks/monthly, with the following features: gradual onset, pressure-like or pulsating quality, severe intensity, duration of some hours, alternating side (prevalent to left) , association to nausea, photophobia, phonophobia and more rarely episodes of vomiting (usually isolated). Main atypical characteristic of these attacks was the pain location, strictly limited to the unilateral superior – inner angle of orbit . The pain rarely had irradiation on the same fronto-temporal side and had selective tenderness to pressure over infratrochlear zone without additional periorbital tenderness . No swollen trochlea may be felt upon palpation. The child was not able to refer presence of pain associated with ocular movements because of severe pain. During the attacks the glycemic levels was normal.

Positive familiarity for migraine . All exams resulted negative.

2° case: A 11-year old male, admitted to our pediatric Headache center on March 2018 because he complained several migrainous attacks since two years with increasing frequency in last six months . The headache showed episodic attacks, frequency of 3-4 attacks/monthly, pulsating quality, severe intensity, duration of several hours, alternating side, association with nausea , photophobia, phonophobia, and mild unilateral cranial autonomic symptoms, no aura. Main atypical characteristic of these attacks was that several attacks reported pain location strictly limited to the unilateral superior – inner angle of orbit associated to tenderness to pressure over infratrochlear zone , without irradiation or swollen trochlea. Positive familiarity for migraine (mother). Neurological examination and instrumental exams were negative. In Table 1 are reported main clinical features of two case-reports.

**Conclusion**: Trochlear Migraine is defined like the association of two concurrent painful disorders where one influences the other [1], even if this used nosographic term seems to indicate more correctly the association of the migraine syndrome to a topographical localization, similar to the “nasal migraine” used to describe migrainous attacks strictly localized in the nasal region [2] . The possible confusion in use of this term to describe the first condition may arise in presence of subjects with migrainous pain localized in trochlear region. Our children showed migrainous attacks that fit perfectly with the term “Trochlear Migraine” if used to define relationship between topographical side and clinical features. These considerations are supported by recent reports describing patients with trochlear pain , alternating pain side and/or nausea and photophobia [3] . Probably this topographic localization of migrainous pain is less rare than is reported. In our opinion the term “Trochlear Migraine” should be reserved to a clinical migrainous syndrome strictly localized in trochlear region. Final relevant question could be if the IHS classification of primary headaches[4] based on syndromic criteria, should allow the excessive increasing descriptions of new primary headache syndromes based on particular criteria. For instance, new syndromes are defined in relation to atypical topography, (see PTH or idiopathic rhinalgia) [1,5] ; to the direction of pain, (see epicrania fugax [6]); to the size of painful area (see nummular headache[7] ; or to associated autonomic symptoms (see red ear syndrome [8]). Alternatively, a more restrictive approach could be chosen to better reallocating headaches in relation to well defined pathophysiological model of cranial pain, based on our actual scientific knowledge where the single case confirm or disconfirm the suggested model. If we consider this second hypothesis, it should be less useful to identify a disorder in relation to localization or other clinical features.

**Consent for publication**: Informed consent to publish has been obtained from this patient.

**References**

1) Yanguela J, Pareja JA, Lopez N, Sanchez Del Rio M. Trochleitis and migraine headache. Neurology 2002; 58:802–805

2) Alvarez M, Montojo T, de la Casa B, Vela L., Pareja J.A. Unilateral nasal pain with migraine features. Cephalalgia , 2013, 33:1055-58

3) Chanlalit W, Teeyapant C, Soodchuen S. Trochlear pain: clinical characteristics and treatment outcomes. J Neurol. 2017 Dec 18. doi: 10.1007/s00415-017-8713-7.

4) The International Classification of Headache Disorders, 3rd edition (beta version). Cephalalgia 2013; 33: 629–808.

5) Pareja JA, Cuadrado ML, Porta-Etessam J, et al. Idiopathic ophthalmodynia and idiopathic rhinalgia: two topographic facial pain syndromes. Headache 2010; 50:1286–1295.

6) Cuadrado ML, Guerrero AL, Pareja JA. Epicrania fugax . Curr Pain Headache Rep 2016; 20: 21

7) Schwartz DP, Robbins MS, Grosberg BM . Nummular Headache Update . Curr Pain Headache Rep . 2013, 17:340

8) Raieli V., Compagno A. Damelio M. Red ear syndrome . Curr Pain Headache Rep. 2016, 20:19

**Table 1 (abstract P129).** Trochlear migraine – two case report

Clinical features		
Sex	M	M
Age at diagnosis trochlear pain	12	11
Age at onset of migraine, y	10	10
Migraine subtype	Ep MwA	Ep MwA
Location of migraine pain	Troch.	Troch/fronto-temp
Age at onset trochlear pain	10	Not specified
Side trochlear pain	Alternanting	Alternanting
Quality troclear pain	Puls./pre	Puls
Temporal pattern of active pain period	Recurrent episodes	Recurrent episodes
Intensity of trochlear pain (not included excerbations)	7-9	7-8
Photo/phonophobia associated to trochlear pain	+	+
Nausea associated to trochlear pain	+	+
Vomiting associated to trochlear pain	+	-
Diplopia	-	-
Trigger trochlear for migraine attacks	Not applicable	Not applicable
Response to local steroid injection	Not applicable	Not applicable

### P130 Efficient therapy for treatment of migraine without aura: ergotamine based drugs or sumatriptan

#### Sinisa Miljkovic^1^, Dz. Smajlovic^2^, S. Crncevic^1^, T. Jurisic^3^, M. Tiric Campara^4^, L. Duranovic Vinkovic^5^, Z. Dostovic^2^, Z. Pasic^2^

##### ^1^University Clinical Center Banja Luka, Department of Neurology, Bosnia and Herzegovina; ^2^University Clinical Center Tuzla, Department of Neurology, Bosnia and Herzegovina; ^3^University Clinical Hospital Mostar, Department of Neurology, Bosnia and Herzegovina; ^4^University Clinical Center Sarajevo, Department of Neurology, Bosnia and Herzegovina; ^5^Cantonal Hospital “Dr. Safet Mujić“ Mostar (Regional Medical Center), Department of Neurology, Bosnia and Herzegovina

###### **Correspondence:** Sinisa Miljkovic (sinisa.miljkovic@kc-bl.com)

**Background:** Triptans, ergotamine and dihydroergotamine are the most effective medications in the treatment of acute migraine without aura. The aim of this study was to examine effectiveness of ergotamine-based fixed combination of Nomigren® (ergotamine tartrate + mecloxamine citrate + camylofin hydrochloride + caffeine + propyphenazone) over sumatriptan.

**Patients and methods:** The study was designed as randomized, double-blind, double-dummy, placebo-controlled, parallel arm, multi-center clinical trial. The study was conducted on 201 patients: 168 female and 33 male subjects from 5 different medical centres. The patients were randomized to one of the two study groups - Nomigren® or sumatriptan group. Diagnosis was based on International Headache Society Criteria (ICHD-3 Beta). The effectiveness was assessed on the basis of complete disappearance of migraine pain two hours after administration of the study medication.

**Results:** Complete reduction of migraine pain after two hours was reported in 51.12% attacks in Nomigren® group, and in 33.70% attacks in sumatriptan group (p=0.0015). Nomigren® has shown equally sustainable high efficiency through repeated migraine attacks. The therapy’s effectiveness in repeated attacks in Nomigren® group was 50.91%, and in sumatriptan group it was 23.73% (p=0,005). Reversal of photophobia, phonophobia and osmophobia was also more frequent in Nomigren® group, 51.12% vs 33.70%. A complete failure of therapy was higher in Sumtriptan in group (9 subjects) compared to Nomigren® group (2 subjects) (p=0.0006).

**Conclusion:** Nomigren® displayed better effectiveness in the complete cessation of migraine pain and accompanying symptoms compared to sumatriptan in the treatment of migraine without aura.

### P131 Analysis of Initial Non Responders to Galcanezumab in Patients with Episodic or Chronic Migraine: Results from the EVOLVE-1, EVOLVE-2, and REGAIN Randomized, Double-Blind, Placebo-Controlled Trials

#### Russell M. Nichols, Dustin Ruff, Eric Pearlman, Sheena K. Aurora

##### ^1^Eli Lilly and Company, Indianapolis, IN, USA

###### **Correspondence:** Russell M. Nichols

**Objective**: To assess response rates in adults with episodic or chronic migraine without initial response to galcanezumab and the likelihood of response with continued treatment.

**Methods**: Post hoc analyses of data from patients with *episodic* [n=879; two 6-month studies (pooled)] or *chronic* migraine (n=555; one 3-month study) treated with galcanezumab (pooled 120 or 240 mg/month) were conducted. Patients without *initial* treatment response (≥50% fewer migraine headache days [MHD] for *episodic* and ≥30% for *chronic*) at Month 1 (NR‑1) or at Months 1 and 2 (NR‑2) were examined. Patients were categorized based on the percentages of MHD improvement seen after 1 month or 1 and 2 months of treatment, as modest response (fewer MHDs by >30% to <50% for *episodic* or >10% to <30% for *chronic*), minimal/no response (≤10% fewer MHDs/ ≤10% more MHDs), or worsening response (>10% more MHDs). The percentage of patients showing good response (≥50% fewer MHDs for *episodic* or ≥30% fewer MHDs for *chronic*) or no response (≤10% fewer MHDs for *episodic* and *chronic*) during the remaining treatment period was calculated for NR-1 and NR-2 categories. Missing MHD values were imputed based on a Bayesian hierarchical regression model.

**Results**: Of *episodic* NR-1 patients with modest, minimal/no, or worsening response after 1 month of initial treatment, 62%, 34%, and 20%, respectively, achieved good response with continued treatment, while 6%, 20%, and 47%, respectively, had no response. Of *chronic* NR-1 patients with modest, minimal/no, or worsening initial treatment response, 38%, 17%, and 11%, respectively, achieved good response with continued treatment, while 30%, 53%, and 74%, respectively, had no response. Of *episodic* NR-2 patients with modest, minimal/no, or worsening response after 1 and 2 months of initial treatment, 50%, 18%, and 9%, respectively, achieved good response with continued treatment, while 4%, 30%, and 65%, respectively, had no response. Of *chronic* NR-2 patients with modest, minimal/no, or worsening initial treatment response, 35%, 13%, and 10%, respectively, achieved good response with continued treatment, while 28%, 61%, and 85%, respectively, had no response.

**Conclusions**: Galcanezumab-treated patients with episodic or chronic migraine who had not responded after 1 or 2 months of treatment appear to have a reasonable likelihood of improvement in the months following initial treatment, with a greater likelihood seen in patients that showed modest improvement with initial treatment. A small percentage of patients who experience worsening MHD following initial treatment did respond with continued dosing.

**Ethics approval**

ClinicalTrials.gov: #NCT02614183 (I5Q-MC-CGAG; EVOLVE-1) and #NCT02614196 (I5Q MC-CGAH; EVOLVE-2) and # NCT02614261 (I5Q-MC-CGAI; REGAIN)

**Trial Registration**: Each study was approved by a central Ethics Review Board and registered on ClincalTrials.gov (NCT02614183 (EVOLVE-1); NCT02614196 (EVOLVE-2); NCT02614261 (REGAIN); NCT02614287 (Study CGAJ)).

**Disclosures:**

**Russell M. Nichols, PharmD:** nichols_russell_m@lilly.com A full-time employee of Eli Lilly and Company and/or one of its subsidiaries.

**Dustin Ruff, PhD:** ruff_dustin@lilly.com A full-time employee of Eli Lilly and Company and/or one of its subsidiaries.

**Eric Pearlman, MD, PhD:** eric.pearlman@lilly.com A full-time employee of Eli Lilly and Company and/or one of its subsidiaries.

**Sheena K. Aurora, MD:** sheena.aurora@lilly.com A full-time employee of Eli Lilly and Company and/or one of its subsidiaries.

**Millie S. Hollandbeck** and **Holly Capasso-Harris** (Synchrogenix) provided writing assistance and editorial assistance.

### P132 Rapid Onset of Effect of Galcanezumab for the Prevention of Episodic Migraine: Post-hoc Analyses of Two Phase 3 Studies

#### Sheena K. Aurora, Holland C. Detke, Brian A. Millen, Hans-Peter Hundemer

##### Eli Lilly and Company, Indianapolis, IN, USA

**Objective:** The humanized monoclonal antibody galcanezumab (LY2951742), which binds to calcitonin gene-related peptide, has been evaluated for migraine prevention. The aim of these post-hoc analyses is to describe the onset of effect of galcanezumab for prevention of episodic migraine based on data from the Phase 3 clinical program.

**Methods:** These analyses were derived from patients who participated in the randomized, double-blind, placebo-controlled Phase 3 studies (EVOLVE-1 [NCT02614183] and EVOLVE-2 [NCT02614196]) that included patients aged 18-65 years with a diagnosis of episodic migraine and a history of migraine headaches for ≥1 year. A total of 1,773 (858 EVOLVE-1, 915 EVOLVE-2) patients were randomized and received either 120 mg or 240 mg galcanezumab (n=879) or placebo (n=894). Study drug was administered subcutaneously once per month for 6 months. The patient population was predominately female (83.7% EVOLVE-1, 85.4% EVOLVE-2) and white (80.4% EVOLVE-1, 70.3% EVOLVE-2) with a mean age of approximately 41 years (40.7 years EVOLVE-1, 41.9 years EVOLVE-2). On average, patients had been diagnosed with migraine approximately 20 years prior to study entry (20.1 years EVOLVE-1, 20.6 years EVOLVE-2), most patients experienced severe disability (Migraine Disability Assessment total score: 33.2 EVOLVE-1, 33.0 EVOLVE-2); the approximate mean number of baseline monthly migraine headache days (MHDs) was 9 (9.1 days EVOLVE-1 9.1 days EVOLVE-2). Patients in the 120-mg group received a 240-mg loading dose for the first month. Onset-of-effect analyses therefore evaluated the pooled galcanezumab-treated patients vs placebo as both galcanezumab groups received 240 mg in the first month. The number of weekly MHDs was modeled using a repeated measures ordinal logistic regression, and the odds ratios of having fewer MHDs for the galcanezumab group compared with the placebo group were evaluated for each week in Month 1. Onset of effect was defined as the earliest week in which a statistically significant separation between galcanezumab and placebo was observed and maintained for all remaining weeks in Month 1.

**Results**: The weekly MHD analyses showed that onset of effect occurred at Week 1 (Table 1). The odds ratio of having fewer weekly MHDs with galcanezumab vs placebo was statistically significant at Week 1 for each study and remained significant for each of Weeks 2-4.

**Conclusion:** This rapid onset of effect of galcanezumab makes it a promising medication for prevention of migraine.

**Ethics approval**

Each study was approved by a central Ethics Review Board and registered on ClincalTrials.gov (NCT02614183 (EVOLVE-1); NCT02614196 (EVOLVE-2); NCT02614287 (Study CGAJ)).

**Table 1 (abstract P132).** See text for description

	Odds ratio	95% CI	p-value
Study 1 (EVOLVE-1)			
Week 1	2.71	2.00, 3.66	<0.001
Week 2	3.08	2.27, 4.17	<0.001
Week 3	2.11	1.55, 2.86	<0.001
Week 4	1.56	1.15, 2.11	0.004
Study 2 (EVOLVE-2)			
Week 1	2.88	2.16, 3.86	<0.001
Week 2	2.76	2.07, 3.68	<0.001
Week 3	2.41	1.80, 3.22	<0.001
Week 4	2.67	1.99, 3.58	<0.001

### **P133 Migraine and cluster headache – the common link**

#### Anne Luise Vollesen^1, &^ (luisevollesen@gmail.com), Silvia Benemei^2, &^ (Italy. silvia.benemei@unifi.it), Francesca Cortese^3, &^ (francesca.cortese@hotmail.com), Alejandro Labastida-Ramírez^4, &^ (a.labastidaramirez@erasmusmc.nl), Francesca Marchese^5, &^ (marchesefrancescaa@gmail.com), Lanfranco Pellesi^6, &^ (lanfranco.pellesi@gmail.com), Michele Romoli^7, &^ (romoli.mic@gmail.com), Messoud Ashina^1^ (ashina@dadlnet.dk), Christian Lampl^8^, and on behalf of the School of Advanced Studies of the European Headache Federation (EHF-SAS)

##### ^1^Danish Headache Center and Department of Neurology, Rigshospitalet Glostrup, Faculty of Health and Medical Sciences, University of Copenhagen, Copenhagen, Denmark; ^2^Health Sciences Department, University of Florence, and Headache Centre, Careggi University Hospital, Florence; ^3^Department of Medico-Surgical Sciences and Biotechnologies, Sapienza. University of Rome, Polo Pontino, Latina, Italy; ^4^Dep Internal Medicine, Division of Vascular Pharmacology, Erasmus Medical Center, Rotterdam, The Netherlands; ^5^Child Neuropsichiatry Unit, University of Palermo, Palermo, Italy; ^6^Medical Toxicology, Headache and Drug Abuse Center, University of Modena and Reggio Emilia, Modena, Italy; ^7^Neurology Clinic, University of Perugia - S.M. Misericordiae Hospital, Perugia, Italy; ^8^Department of Neurogeriatric Medicine, Headache Medical Center Linz, Linz, Austria.

###### **Correspondence:** Christian Lampl (christian.lampl@ordensklinikum.at)

^&^ Contributed equally to the study

Although clinically distinguishable, migraine and cluster headache share prominent features such as unilateral pain, common pharmacological triggers such glyceryl trinitrate, histamine, calcitonin gene-related peptide (CGRP) and response to triptans and neuromodulation. Recent data also suggest efficacy of anti CGRP monoclonal antibodies in both migraine and cluster headache. While exact mechanisms behind both disorders remain to be fully understood, the trigeminovascular system represents one possible common pathophysiological pathway and network of both disorders. Here, we review past and current literature shedding light on similarities and differences in phenotype, heritability, pathophysiology, imaging findings and treatment options of migraine and cluster headache. A continued focus on their shared pathophysiological pathways may be important in paving future treatment avenues that could benefit both migraine and cluster headache patients.

### P134 Providing care: Cost-effective and affordable

#### Michela Tinelli (m.tinelli@lse.ac.uk)

##### Personal Social Services Research Unit, London School of Economics (LSE), Houghton Street, London WC2A 2AE, UK

**Introduction and objectives:** Headache disorders are real illnesses, often causing lifelong disabilities. Migraine, tension-type headache (TTH) and medication-overuse headache (MOH) are of major public-health importance: collectively, they are the 2nd highest cause of disability in populations throughout the world, leading to much lost productivity and very high indirect costs (>€100 billion per year in EU). The Value of Treatment (VoT) Project is a timely and ground-breaking initiative of the European Brain Council (EBC) in collaboration with the LSE, *Lifting The Burden* (LTB), European Headache Federation and other partner institutions. It argues that optimizing interventions in brain disorders can bring not only positive outcomes for patients but also economic gains for society. As part of the VoT project, the headache economic case study estimated cost/effectiveness of implementing structured headache services (SHSs) based in primary care and supported by educational initiatives aimed at both patients and health-care providers.

**Methods**: We modelled cost-effectiveness of SHSs delivering treatments for each of the headache types (migraine, TTH, MOH), with efficacy known from randomized controlled trials. Three health-care systems – of Russia, Spain and Luxemburg – brought different experiences of health service delivery and financing into the model. We made annual (short-term) and 5-year cost estimates from health-care provider and societal perspectives (2017 figures, euros). We expressed effectiveness as healthy life years (HLYs) gained, and cost-effectiveness as incremental cost-effectiveness ratios (ICERs) (cost to be invested/HLY gained).

**Results**: In short-term modelling from the health-care provider perspective, the intervention is cost effective overall and across headache types – well below WHO framework thresholds. Over 5 years, the intervention is even more cost effective. Results are consistent across health-care systems. From the societal perspective, the intervention is not only cost-effective but also cost-saving over 1 year and 5 years, for all types of headache and across health-care systems. The greater the country’s wage levels, the greater are the economic savings for society (Luxemburg > Spain > Russia).

**Discussion**: For the first time, the effectiveness and cost effectiveness of introducing hypothetical SHSs in Europe was evaluated across health-care systems. Our results study showed that such services, based in primary care and supported by patient and provider education, are effective and cost-effective solutions to headache disorders and the disability they cause. From the health-care provider perspective, cost-effectiveness is least (ICERs greatest) for TTH because of its much lower disability weight compared with those for migraine and MOH. In practice, structured headache services will not discriminate: they must manage all headache types; however, people with TTH are least likely to require them.

**Acknowledgments**

This study was part of the EBC-led Value of Treatment Project. The headache economic study team included Michela Tinelli *^1^, Timothy Steiner*^2^, Matilde Leonardi^3^, Dimos Mitsikostas^4^, Koen Paemeleire^4^, Elena Ruiz de la Torre^5,6^. ^1^The London School of Economics and Political Science; ^2^*Lifting The Burden*; ^3^European Brain Council (EBC) WHO Liaison; ^4^European Headache Federation; ^5^European Federation of Neurological Associations (EFNA); ^6^European Headache Alliance (EHA). *These authors contributed equally.

### P135 The Activity Impairment in Migraine – Diary (AIM-D): A novel migraine-specific patient-reported outcome measure to assess functioning based on activity impairment in episodic and chronic migraine patients

#### ML Cala^1^, CA Graham^1^, RB Lipton^2^, N Lyn^1^, DW Dodick^3^, C Burk^4^, JS Yu^5^, CJ Evans^1^, HN Viswanathan^5^

##### ^1^Endpoint Outcomes, Boston, MA, USA; ^2^Albert Einstein College of Medicine, Bronx, NY, USA; ^3^Mayo Clinic, Phoenix, AZ, USA; ^4^Independent consultant, Orange County, CA, USA; ^5^Allergan plc, Irvine, CA, USA

###### **Correspondence:** RB Lipton

**Objective:** To develop a patient-reported outcome (PRO) measure to assess relevant impacts of migraine in episodic migraine (EM) and chronic migraine (CM) patients for use in clinical trials of migraine preventive treatments.

**Methods:** The AIM-D was developed using an iterative instrument development process involving (1) a review of the literature and relevant PRO measures; (2) interviews with clinicians; (3) mixed concept elicitation (CE) and cognitive interviews (CIs) in 20 EM and 20 CM patients; (4) concept confirmation and item generation based on qualitative analysis and input from clinical experts and PRO measurement experts; (5) three rounds of CIs (18 EM and 20 CM patients) of the draft AIM-D; and (6) finalization based on patient input from CIs. Qualitative analysis was conducted using ATLAS.ti.

**Results:** Qualitative analysis of the mixed CE/CIs, which confirmed conceptual saturation, helped to identify impacts that were relevant to migraine patients and to develop the draft AIM-D. The AIM-D includes items that assess usual household chores, errands, leisure activities at home, leisure or social activities outside the home, strenuous physical activities, walking, body movement, bending forward, and head movement. Response options for each item range from “not difficult at all” to “I could not do it at all” on a 6-point rating scale. The novel PRO measure was developed as an electronic handheld daily diary with headache and non-headache administration options to better elicit responses from migraine patients using a 24-hour recall period. Four additional items that assess difficulty in concentrating, difficulty in thinking clearly, activity level, and activity limitation were also developed and debriefed for future evaluation of the AIM-D. Changes were made after each of the first two rounds of CIs based on patient input and informed by the experts and research team. No changes were needed after the final round. Input from CIs confirmed that instructions, recall period, items, and response options were well understood by patients.

**Conclusions:** The AIM-D followed a comprehensive instrument development process in keeping with the US FDA 2009 guidance for PROs and measures functioning based on the performance of daily activities and physical impairment related to migraine. The results of this study provide evidence of the content validity of the AIM-D for use in EM and CM patients.

**Declarations**:

MLC, CAG, NL and CJE are employees of Endpoint Outcomes, who was paid as a consultant by Allergan plc. RBL is the Edwin S. Lowe Professor of Neurology at the Albert Einstein College of Medicine in New York. He receives research support from the NIH: 2PO1 AG003949 (Program Director), 5U10 NS077308 (PI), RO1 NS082432 (Investigator), 1RF1 AG057531 (Site PI), RF1 AG054548 (Investigator), 1RO1 AG048642 (Investigator), R56 AG057548 (Investigator), K23 NS09610 (Mentor), K23AG049466 (Mentor), 1K01AG054700 (Mentor). He also receives support from the Migraine Research Foundation and the National Headache Foundation. He serves on the editorial board of Neurology, senior advisor to Headache, and associate editor to Cephalalgia. He has reviewed for the NIA and NINDS, holds stock options in eNeura Therapeutics and Biohaven Holdings; serves as consultant, advisory board member, or has received honoraria from: American Academy of Neurology, Alder, Allergan, American Headache Society, Amgen, Autonomic Technologies, Avanir, Biohaven, Biovision, Boston Scientific, Dr. Reddy’s, Electrocore, Eli Lilly, eNeura Therapeutics, GlaxoSmithKline, Merck, Pernix, Pfizer, Supernus, Teva, Trigemina, Vector, Vedanta. He receives royalties from Wolff’s Headache 7^th^ and 8^th^ Edition, Oxford Press University, 2009, Wiley and Informa. DWD Received compensation from serving on advisory boards and/or consulting within the past 5 years for: Allergan, Amgen, Novartis, Alder, Arteaus, Pfizer, Colucid, Merck, NuPathe, Eli Lilly and Company, Autonomic Technologies, Ethicon J&J, Zogenix, Supernus, Labrys, Boston Scientific, Medtronic, St Jude, Bristol-Myers Squibb, Lundbeck, Impax, MAP, Electrocore, Tonix, Novartis, Teva, Alcobra, Zosano, ZP Opco, Insys, Ipsen, Acorda, eNeura, Charleston Laboratories, Gore, Biohaven, Biocentric, Magellan, Theranica, Xenon, Dr Reddy’s/Promius Pharma, Vedanta, Electrocore, CC Ford West Group, Foresight. Dr Dodick owns equity in Epien, GBS/Nocira, Second Opinion, Healint, and Theranica. Dr Dodick has received funding for travel, speaking, editorial activities, or royalty payments from IntraMed, SAGE Publishing, Sun Pharma, Allergan, Oxford University Press, American Academy of Neurology, American Headache Society, West Virginia University Foundation, Canadian Headache Society, Healthlogix, Universal Meeting Management, WebMD, UptoDate, Medscape/WebMD, Oregon Health Science Center, Albert Einstein University, University of Toronto, Starr Clinical, Decision Resources, Synergy, MedNet LLC, Peer View Institute for Medical Education, Medicom, Medlogix, Wolters Kluwer Health, Chameleon Communications, Academy for Continued Healthcare Learning, Haymarket Medical Education, Global Scientific Communications, Miller Medical Communications, MeetingLogiX, Wiley Blackwell. Dr Dodick, through his employer, has consulting use agreements with NeuroAssessment Systems and Myndshft. He holds board of director positions with King-Devick Technologies, and Epien Inc. He holds the following Patent 17189376.1-1466:vTitle: Botulinum Toxin Dosage Regimen for Chronic Migraine Prophylaxis (no compensation). CB is a consultant for Allergan. JSY is an employee of Allergan plc and receives stock or stock options. HNV is an employed by Allergan plc, with ownership interest, including stock, stock options, patent or other intellectual property. Former employee of Amgen Inc. (until January 31, 2016), with ownership interest, including stock, stock options, patent or other intellectual property.

### P136 Epidemiology of chronic and episodic migraine in Europe

#### Hicham Benhaddi^1^, Timothy Fitzgerald^2^, Sophie McCabe^3^, Ruth Zeidman^3^

##### ^1^Teva Pharmaceuticals, Wilrijk, Belgium; ^2^Teva Pharmaceuticals, Frazer, Pennsylvania, USA; ^3^Covance Market Access, London, UK

###### **Correspondence:** Hicham Benhaddi (kgora@hcg-int.com)

**BACKGROUND:**

Migraine is a debilitating neurological disorder characterized by headaches of varying duration and intensity. Treatment options vary depending on disease severity, frequency, regional practices, and therapy availability.

**OBJECTIVE:**

To conduct a systematic literature review of the epidemiology (incidence, prevalence, mortality, and morbidity), current treatment pathways and patterns, preventive therapy guidelines, and unmet needs of chronic (CM) and episodic migraine (EM) in Europeans.

**METHODS:**

Literature searches and evidence screening were structured according to the PICOS (population, intervention, comparators, outcomes, and study types) framework. Reviews, original studies, and clinical guidelines in European adults (≥18 years) with EM (<15 headache days per month), CM (≥15 headache days with ≥8 migraine days per month), or medication overuse headache (MOH) were included. Searches focused on epidemiology, incidence, prevalence, mortality, morbidity, treatment patterns, clinical guidelines relating to preventive interventions, and unmet need (published 2007–February 1, 2018; geographical limitation: United Kingdom, France, Germany, Spain, Italy, the Netherlands, Poland, Denmark, Finland, Iceland, Norway and Sweden). Searches included: Embase, MEDLINE, and the Cochrane Library databases; specialty medicine associations; and health technology assessment agency websites.

**RESULTS:**

Analysis included 64 publications. The World Health Organization estimated 77 million migraine sufferers in Europe, with an incidence of up to 39.2 per 1000 patient-years. Migraine prevalence was higher (2–6-fold) in women than in men in all but one study, with peak prevalence at 25–55 years of age. EM was up to 15 times more prevalent than CM. MOH has an estimated prevalence of 1% in the general adult population. Preventive therapies are typically beta-blockers, antidepressants, anticonvulsants (topiramate), or in some countries, onabotulinumtoxinA (for chronic migraine), but most have no proven efficacy in CM. Guidelines vary by country, but the European Headache Federation (EHF) recommends preventive therapy for patients with ≥2 debilitating attacks per month; however, ≤13% of patients who qualify for preventive treatment actually receive it. Adherence to available preventive therapies is low, and many physicians believe that the disadvantages, including adverse events, drug dependency, and lack of sustained efficacy outweigh the benefits. To manage MOH, withdrawal of treatment is recommended.

**CONCLUSIONS**: Migraine is highly prevalent in Europe. Despite improvements in compliance with treatment guidelines, the number of patients on preventive therapies remains low, likely due to poor tolerability and efficacy of available therapies and limited access to these medications due to restrictive guidelines.

### P137 The impact of offering monthly and quarterly dosing options for a new class of migraine preventive therapy on likelihood of acceptance and adherence in adults with migraine

#### Robert Cowan^1^, Joshua Cohen^2^, Erik Rosenman^3^, Tim Fitzgerald^2^, Ravi Iyer^2^

##### ^1^Neurology, Stanford University School of Medicine, Stanford, California, 94305, USA; ^2^Teva Pharmaceutical Industries, Frazer, Pennsylvania, 19355, USA; ^3^IQVIA, Inc., Cambridge, Massachusetts, 02139, USA

###### **Correspondence:** Ravi Iyer (pjsisi1999@gmail.com)

**Background**

Migraine affects approximately 39 million people in the US^1^. A new class of migraine preventive therapy launching in 2018-2019 will provide physicians and patients with an alternate approach to preventive treatment. This study sought to understand the impact, if any, of having both monthly and quarterly dosing options on acceptance of, and adherence to, the new class of migraine preventive therapy among adults with migraine.

**Methods**

In this double-blind, observational study, 420 US adults with migraine completed a 20-minute, self-administered online survey. Respondents included 228 moderate-frequency episodic (5-9 headache days / month), 106 high-frequency episodic (10-14 headache days / month), and 86 chronic migraine patients (≥15 headache days / month). Adults with migraine were exposed to three scenarios: 1) only monthly dosing of the new class of migraine preventive therapy is available, 2) only quarterly dosing is available, and 3) both monthly and quarterly dosing are available. In each scenario, and assuming roughly equivalent efficacy regardless of dosing schedule, adults with migraine were asked their likelihood to fill the prescription (if prescribed) and their likelihood to take it consistently over one year, measured on a 7-point scale where 1 was “not at all likely” and 7 was “extremely likely”. Those that selected a 6 or 7 on the scale were classified as “likely”. At the end of the survey, respondents were then asked if they preferred either monthly or quarterly dosing for this new class of therapy. Data analysis included descriptive statistical analyses and comparison of means through ANOVA testing, with significance set at p<0.05.

**Results**

A similar proportion of adults with migraine preferred monthly (35.7%) and quarterly (39.5%) dosing regimens (24.8% had no preference). Among those who prefer monthly dosing (n=150), a greater proportion indicate they are likely to fill the prescription and remain adherent when only monthly is prescribed and available compared to when only quarterly is (77% vs. 56% p<0.001 and 80% vs. 57% p<0.001 respectively). Likewise, among those who prefer quarterly dosing (n=166), a greater proportion indicate they are likely to fill and remain adherent when only quarterly is prescribed and available compared to when only monthly is (63% vs. 55% p<0.008 and 62% vs. 54% p<0.023 respectively).

**Conclusions**

Adults with migraine are more likely to fill the new class of preventive therapy and to take it consistently over one year when presented with their preferred dosing regimen.

**References**

1. http://migraineresearchfoundation.org/about-migraine/migraine-facts/ Last accessed June 22^nd^, 2018

### P138 Prognosis of trigeminal neuralgia is very favourable when managed by specialists - a two-year prospective real-life study

#### Tone Bruvik Heinskou^1^, Stine Maarbjerg^1^, Frauke Wolfram^2^, Per Rochat^3^, Jannick Brennum^3^, Jes Olesen^1^ and Lars Bendtsen^1^

##### ^1^Danish Headache Center, Department of Neurology, Rigshospitalet Glostrup, Faculty of Health and Medical Sciences, University of Copenhagen, 2600 Glostrup, Denmark; ^2^Department of Diagnostics, Herlev Hospital, 2730 Herlev, Denmark; ^3^Department of Neurosurgery, Rigshospitalet Blegdamsvej, University of Copenhagen, 2100 Copenhagen, Denmark

###### **Correspondence:** Tone Bruvik Heinskou (tone.bruvik.heinskou@regionh.dk)

**Background:** Prognosis of trigeminal neuralgia is generally assumed to be poor with slowly deterioration over time. Prospective real-life studies of medical management of trigeminal neuralgia are almost non-existent. The aim of this prospective real-life observational study was to investigate the two-year prognosis for patients with trigeminal neuralgia treated by specialists.

**Methods:** Consecutive patients were enrolled in a structured multidisciplinary management program at a public tertiary specialist centre for headache and facial pain from May 2012 to December 2015. Optimization of medical treatment as well as non-medical treatment, i.e., physiotherapy, psychotherapy and continuous support and advice from specially trained nurses, were parts of the management program. Medically intractable patients were referred for surgery. Data were collected prospectively using standardized schemes and patient surveys.

Primary outcome was 50% reduction in overall burden of pain according to Verbal Numerical Rating Scale (VNRS), over a two-year period.

**Results:** A total of 103 medically treated patients, (72 women, 31 men), were followed for two years. Another 50 patients were treated neurosurgically before their two-year follow-up. Half of the patients (53 (51%)) had more than 50% reduction in the overall burden of pain. VNRS was reduced from 5.34 to 3.00, p < 0.01. Patients with ≥ 5 years of disease duration had 2.5 times higher odds of a good two- year outcome compared to patients with a disease duration of < 5 years, p = 0.04. There was no significant association between the primary outcome and sex, depression and/or anxiety, concomitant persistent pain or morphological changes of the trigeminal nerve. There was a significant increase in the number of patients treated with oxcarbazepine but there was no significant dosage increment of any TN drugs. Preliminary data indicate that also the surgically treated patients had a very favorable outcome.

**Conclusions:** This large prospective and systematic real-life study demonstrates, in contrary to what has previously been assumed, that TN patients enrolled in a structured medical treatment program, improve considerably over a two-year period. The improvement could be due to optimization of drug treatment, education and continuous advice and support, and natural history of the disease. The results provide hope and optimism for patients and care providers and suggest that specialist management of trigeminal neuralgia is highly rewarding.

### P139 Serum changes of apolipoproteins in Medication Overuse Headache (MOH)

#### Lanfranco Pellesi^1^, Elisa Bellei^2^, Carlo Baraldi^1^, Flavia ^1^, Simona Guerzoni^1^, Emanuela Monari^2^, Luigi Alberto Pini^1,3^

##### ^1^Medical Toxicology, Headache and Drug Abuse Centre, University of Modena and Reggio Emilia, Modena, Italy; ^2^Department of Diagnostic Medicine, Clinic and Public Health, Proteomic Lab, University of Modena and Reggio Emilia, Modena, Italy; ^3^Center for Neuroscience and Neurotechnology, University of Modena and Reggio Emilia, Modena, Italy

###### **Correspondence:** Lanfranco Pellesi (lanfranco.pellesi@gmail.com)

**Background**

Medication Overuse Headache (MOH) is a prevalent and disabling disorder resulting from the overuse of analgesic drugs, triptans or other acute headache medications. Previous proteomic studies have identified some altered proteins, including different forms of apolipoproteins, which are probably associated with the chronic painful symptom and its consequences. The aim of the study was to explore the relationship between cutaneous pain thresholds, Zung Self-Rating Depression Scale (ZUNG-D) scores, Leeds Dependence Questionnaire (LDQ) scores and serum levels of apolipoprotein A1 (APOA1) and apolipoprotein E (APOE) in patients with MOH.

**Methods**

69 patients with MOH and 42 healthy volunteers as control group were enrolled in the study between September 2016 and January 2018. To investigate skin sensitivity, Von Frey-like filaments were applied sequentially to the skin territories innervated by the divisions of the trigeminal nerve, to determine cutaneous pain thresholds. APOA1 and APOE, previously identified as potential biomarkers candidates for the pathophysiology of chronic pain, were quantified in the serum by Enzyme-linked Immunosorbent Assay (ELISA).

**Results**

Cutaneous pain thresholds were lower among patients with MOH than healthy controls. Serum APOE was significantly lower in patients with MOH, compared to healthy volunteers (*p* < 0.01), but no differences were found concerning serum APOA1 (Fig. 1). In patients with MOH, serum APOE was positively related to Body Mass Index, albumin and uric acid, whereas serum APOA1 was positively related to creatinine. Serum APOA1 and APOE did not have any relationship with cutaneous pain thresholds, ZUNG-D scores, LDQ scores and other clinical or laboratory parameters.

**Conclusions**

Serum APOE is significantly altered in patients with MOH compared to controls, but it is apparently not correlable with any aspect of the disease. APOE may play a role in the pathophysiology of MOH and the consequences associated with medication overuse; further studies are needed to deepen this finding.

**Ethics approval**

This study was performed following the Helsinki Declaration principles and approved by the local Ethical Committee (prot. 2073).

**Fig. 1 (abstract P139).**
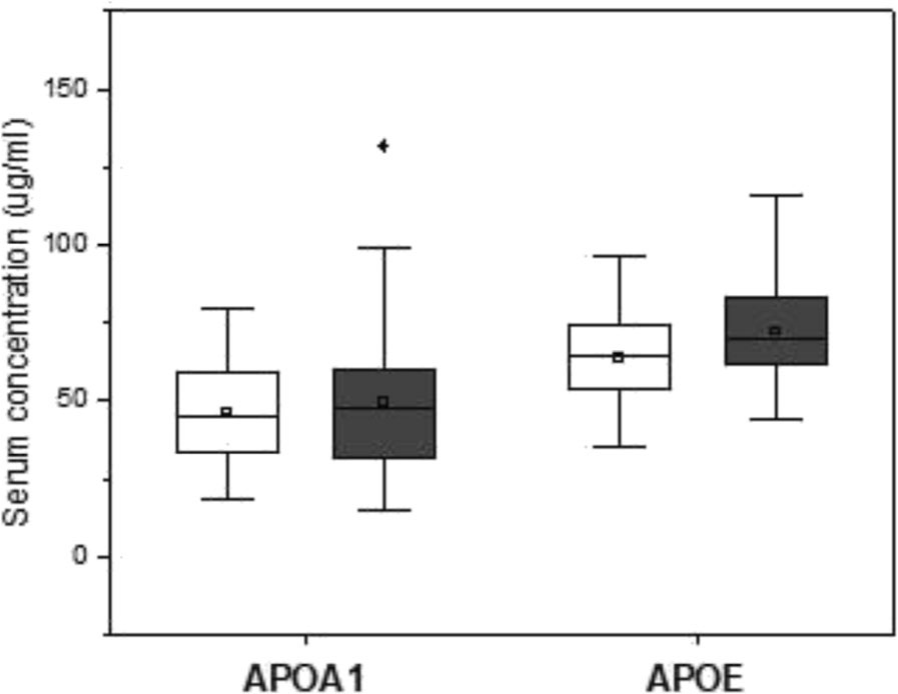
Box plot of serum APOA1 and APOE in patients with MOH (white) and healthy volunteers (grey).

### P140 Robust association between Obstructive sleep apnea (OSA) and Idiopathic intracranial hypertension (IIH) in Chronic daily headache (CDH).

#### G. Demonte^1^, L. Rapisarda^1^, F. Tosto^1^, F. Roccia^2^, M. Sturniolo^3^, A. Gambardella^3^, F. Bono^2^

##### ^1^Headache center, Institute of Neurology, Department of Medical and Surgical Sciences - Magna Graecia University – Catanzaro; ^2^Rehabilitative Cardiology - AOU Mater Domini - Catanzaro; ^3^Institute of Neurology, Department of Medical and Surgical Sciences - Magna Graecia University – Catanzaro

###### **Correspondence:** G. Demonte (g.dem.1990@gmail.com)

**Introduction**

It’s widely recognized that Obstructive sleep apnea (OSA) is a risk factor for chronic daily headache (CDH)^[1]^. However, the association between OSA and CDH with idiopathic intracranial hypertension (IIH) remains today uncertain. The aim of this study was to investigate the frequency of OSA in a population with CDH and IIH.

**Materials and methods**

In this prospective study we enrolled 49 consecutive patients with CDH and suspected IIH who attended the Institute of Neurology in Catanzaro from 2016 to 2018. All patients underwent a general and neurological examination. The frequency of headache, through a monthly headache score diary, and the effects of postural changes were recorded; moreover, we evaluated rating scales for pain (VAS), disability (MIDAS), allodynia (ASC-12); depression and anxiety (BDI-II and HAMA), and medical overuse. All patients underwent brain MRI and MR-venography and 1-hour lumbar cerebrospinal fluid pressure monitoring via a spinal puncture needle to evaluate IIH. In addition, we performed a polysomnographic evaluation with apnea-hypopnea index (AHI) and Snoring index to investigate the presence of OSA.

**Results**

We enrolled in this study 49 subjects (14 M, 35 F; age 41,3±13,2; BMI 31,2±7). 37 patients (75,5 %) were diagnosed with IIH (9 M, 28 F; age 40,6±13,8; BMI 32,3±7,2). 18 patients (36 %)

were diagnosed with OSA (8 M, 10 F; age 46,1±10,0; BMI 34,5±7,8; AHI 30,0±27,7; Snoring index 135,5±166,9); Among them, 7 subjects presented a mild OSA, 7 subject presented a moderate OSA and 4 subjects presented a severe OSA. Finally 14 patients (28,6 %) presented both IIH and OSA (6 M, 8 F; age 44,8±10,1; BMI 36,8±6,9; AHI 26,9±28,2; Snoring index 136,9±182,9). Among them, 7 subjects presented a mild OSA, 3 presented a moderate OSA and 4 presented a severe OSA.

**Discussion and conclusions**

Our data showed that the frequency of OSA in patients with IIH was of 37,8 % (14/37), with a similar representation of male and female patients (6 M, 8 F). Several mechanisms have been proposed about how OSA may cause IIH. Cerebral vasodilatation related to hypoxia and hypercapnia, increase of systemic blood pressure and reduction of cerebral venous return are considered possible causes of the increase of the intracranial pressure^[2]^. Considering that OSA represents a strong risk factor in developing CDH, our data highlight how IIH may represent an important link between these two disorders. Thus, IIH should be investigated in patients with CDH associated with OSA.

**References**

1. Stark, C.D., Stark, R.J. Sleep and chronic daily headache. Curr Pain Headache Rep. 2015;19:468

2. Wardly DE. Intracranial hypertension associated with obstructive sleep apnea: a discussion of potential etiologic factors. Med Hypotheses 2014;83:792

### P141 Loss of periventricular white matter microstructure integrity in idiopathic intracranial hypertension

#### Laura Rapisarda^1^, Giulio Demonte^1^, Federico Tosto^1^, Maria Curcio^1^, Alessia Sarica^2^, Antonio Cerasa^2^, Antonio Gambardella^1,2^, Francesco Bono^1,2^

##### ^1^Headache Center, Institute of Neurology, Department of Medical and Surgical Sciences, Magna Graecia University- Catanzaro, Italy; ^2^Neuroscience Research Center, Department of Medical and Surgical Sciences, Magna Graecia University of Catanzaro, Italy

###### **Correspondence:** Laura Rapisarda (laura_rapisarda90@yahoo.it)

**Objective**

To evaluate whether high cerebrospinal fluid (CSF) pressure causes impairment of white matter (WM) microstructural integrity in patients with idiopathic intracranial hypertension (IIH).

**Materials and methods**

We recruited 62 patients with refractory chronic headache. They were grouped in 2 groups: Group 1 included 35 patients with IIH, Group 2 included 27 non-IIH patients with refractory chronic migraine (CM) or chronic tension-type headache (CTTH). Patients underwent neurological and ophtalmological examinations, brain magnetic resonance (MRI), cerebral MR venography (MRV) and monitoring of CSF pressure through spinal lumbar puncture (LP) [1]. MRI data were acquired on a 3T unit, the protocol included: (a) 3D T1-weighted spoiled gradient echo (SPGR) sequence; (b) 3D MR venography of the brain oriented in the sagittal plane; (c) diffusion-weighted acquisition using spin-echo echo-planar imaging. Diffusion-weighted images were processed using the tools of FMRIB.

Statistical analysis was performed using the Statistical Package for Social Science software (SPSS, v20.0, Chicago, IL, USA) for Macintosh. A tract-based spatial statistics (TBSS) analysis was applied for exploring abnormalities of white matter (WM) structures, where age, gender and disease duration were added as covariates.

**Results**

Group 1, showed elevated CSF opening, mean and highest peak pressure (294±51; 305±36; 417±50 mmH_2_O), associated with abnormal pressure waves. Group 2 had normal opening, mean, highest peak pressure (166±18; 167±19; 205±25 mmH_2_O). All patients had no brain MR evidence of global or periventricular white matter hyperintensities. Compared with non-IIH patients, IIH patients showed significantly lower MD, AD and RD in several brain regions mainly in body and splenium of the corpus callosum (CC), right superior corona radiata (SCR), whereas no difference was observed in FA.

Regression analysis demonstrated a negative correlations among the average AD values and all the three CSF pressures (*p* < 0.05), particularly in forementioned areas.

**Discussion**

These results indicated that the mechanical compression produced by the repetitive abnormal CSF pressure pulsations against the wall of the lateral ventricles, leading to a dense packaging of periventricular fibers, caused the disruption of WM architecture.

This study demonstrated a disruption of the periventricular white matter microstructure on diffusion tract-based analysis in patients with IIH with and without papilloedema. For the first time, DTI metrics highlighted a subtle microstructural in vivo changes in human brain of patients with IIH.

**Conclusion**

There is an axonal loss in the periventricular WM of patients with IIH suggesting an impairment of the WM microstructural integrity correlated with high CSF pressure.

**REFERENCES**

1. Bono F, Salvino D, Tallarico T, et al. Abnormal pressure waves in headache sufferers with bilateral transverse sinus stenosis. Cephalalgia 2010;30:1419-1425.

### P142 Quality of life in the active phase of cluster headache

#### Ertsey C^1^, Dióssy M^1^, Magyar M^1,2^, Gyüre T^2^, Csépány É^1,2^, Bozsik Gy^1^

##### ^1^Dept. of Neurology, Semmelweis University, Budapest, Hungary; ^2^Szentágothai János Doctoral School of Neurosciences, Semmelweis University, Budapest, Hungary

###### **Correspondence:** Ertsey C (konfreg1@bcdtravel.hu)

**Introduction:** Cluster headache (CH), which affects 0.1% of the population, is one of the most painful human conditions: despite adequate treatment, the frequent and severe headaches cause a significant burden to the patients. According to a small number of previous studies, CH has a serious negative effect on the sufferers’ quality of life (QOL). In the current study we set out to examine the quality of life of the CH patients attending our outpatient service between 2013 and 2016, usng generic and headache-specific QOL instruments.

**Methods:** A total of 42 CH patients (16 females and 26 males; mean age: 39,1±13,5 years) completed the SF-36 generic QOL questionnaire and the headache-specific CHQQ questionnaire (Comprehensive Headache-related Quality of life Questionnaire), during the active phase of their headache. Their data were compared to those of patients suffering from chronic tension type headache (CTH) and to data obtained from controls not suffering from significant forms of headache, using Kruskal-Wallis tests.

**Results:** During the active phase of CH, the patients’ generic QOL was significantly worse than that of normal controls in 4 of the 8 domains of the SF36 instrument. Apart from a significantly worse result in the ‘Bodily pain’ SF-36 domain, there were no significant differences between the CH patients’ and the CTH patients’ results. All the dimensions and the total score of the headache-specific CHQQ instrument showed significantly worse QOL in the CH group than in the CTH group or in the control group.

**Conclusion:** Cluster headache has a significant negative effect on quality of life. The decrease of QOL experienced by the patients was better reflected by the headache-specific CHQQ instrument than the generic SF-36 instrument.

### P143 Self-detoxification from medication overuse reduces disability among patients with MOH

#### Espen Saxhaug Kristoffersen^1,2,3^, Ragnhild Berling Grande^1,4^, Kjersti Aaseth^1,3^, Michael Bjørn Russell^1,5^ and Christofer Lundqvist^1,3,5,6^

##### ^1^Head and Neck Research Group, Research Centre, Akershus University Hospital, Lørenskog; ^2^Department of General Practice, Institute of Health and Society, University of Oslo, Oslo; ^3^Department of Neurology, Akershus University Hospital, Lørenskog; ^4^The National Center for Epilepsy, Division for Surgery and Clinical Neuroscience, Oslo University Hospital, Oslo; ^5^Institute of Clinical Medicine, Campus Akershus University Hospital, University of Oslo, Nordbyhagen; ^6^Health Services Research Centre, Akershus University Hospital, Lørenskog, Norway

***Background****:* Medication-overuse headache (MOH) is a chronic headache associated with overuse of acute headache medication. It is also a major cause of headache-related disability. MOH has similarities with substance dependence disorders and withdrawal of acute headache analgesics has been demonstrated to be effective treatment. However, concerns have been raised that medication withdrawal may give patients a negative label and thus contribute to disease burden and disability.

***Objective****:* The present cohort study investigated headache-related disability among MOH patients identified in a general population sample before and after self- detoxification. In addition, possible predictors for successful outcome were investigated.

***Methods****:* This was a prospective cohort study. Participants were identified in a cross- sectional epidemiological sample of 30 000 persons aged 30–44 from the general Norwegian population. At the end of the interview, people with MOH received short oral information about their headache and the possible role of medication overuse in headache chronification. No other intervention or contact with participants was performed. A total of 108 of the 128 participants were eligible for follow-up 1.5 years later, i.e. an 84 % participation rate. Disability was assessed using the Migraine Disability Assessment (MIDAS) questionnaire.

***Results****:* People with MOH from the general population were heavily disabled at baseline with 54% in the most severe disability class (mean MIDAS score: 42.1, 95% CI 31.7; 52.6). 55% 59/108) had migraine co-occurrence and 13% (14/108) had chronic migraine. The mean number of years of preexisting chronic headache was 16 years and mean number of years of medication overuse was 9 years. 76% (82/108) of participants no longer had medication overuse at follow-up. The MIDAS score was significantly improved at follow-up (p<0.001) for those with successful self-detoxification. Co- occurrence of migraine (p=0.044) and lower headache frequency at baseline (p=0.001) increased the odds for successful self-detoxification and reversion to episodic headache. No migraine, high baseline headache frequency and high levels of psychological distress were associated with a high headache frequency at follow-up.

***Conclusion****:* MOH causes substantial disability in the general population. Self- detoxification leads to reduced headache frequency as well as reduced disability.

**Ethics approval**

The relevant regional research ethical committee approved the study and participation was by signed informed consent.

**Fig. 1 (abstract P143).**
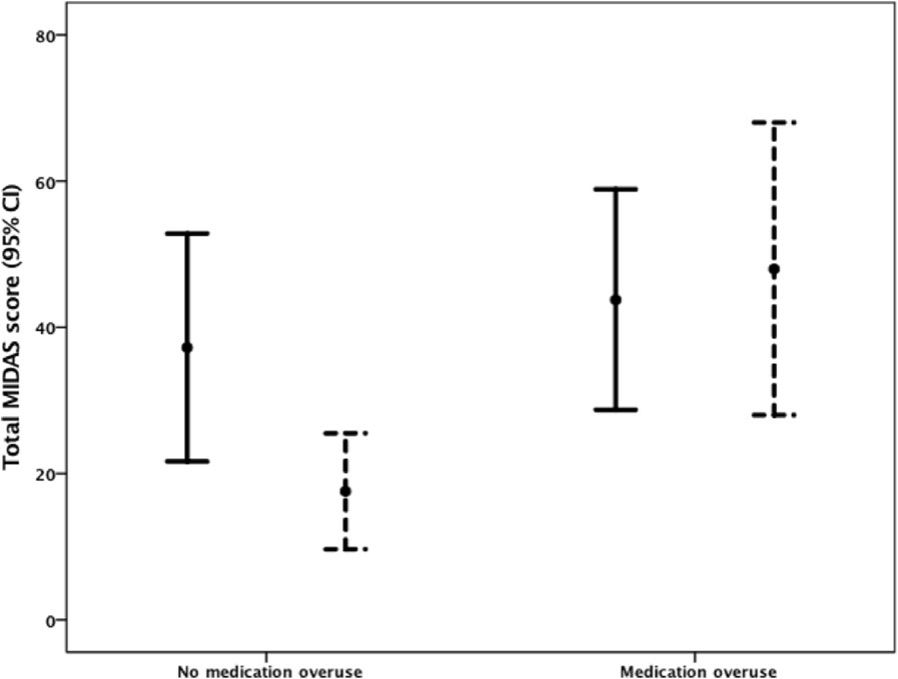
MIDAS score at baseline (lines) vs. follow-up (dashed) of participants with/without medication overuse at follow-up. (All had medication overuse at baseline.)

### P144 Effects of non-invasive vagus nerve stimulation (nVNS) on resting-state EEG and laser-evoked potentials in migraine: findings from a subgroup of patients enrolled in the randomised, sham-controlled, double-blind PRESTO study

#### Eleonora Vecchio^1^, Iege Bassez^2^, Katia Ricci^1^, Cristina Tassorelli^3,4^, Eric Liebler^5^, Marina de Tommaso^1^

##### ^1^Applied Neurophysiology and Pain Unit, SMBNOS Department, Polyclinic General Hospital, University of Bari Aldo Moro, Bari, Italy; ^2^Department of Data Analysis, Ghent University, Ghent, Belgium; ^3^Headache Science Centre, National Neurological Institute C. Mondino Foundation, Pavia, Italy; ^4^Department of Brain and Behavioral Sciences, University of Pavia, Pavia, Italy; ^5^electroCore, LLC, Basking Ridge, New Jersey, USA

###### **Correspondence:** Eleonora Vecchio (eleonora.vecchio@gmail.com)

**Background:** The randomised sham-controlled PRESTO study provided Class I evidence that for patients with an episodic migraine, non-invasive vagus nerve stimulation (nVNS; gammaCore®) significantly increases the probability of having mild pain or being pain-free 2 hours post‑stimulation [1]. These effects may be driven by multiple mechanisms including inhibition of the trigeminovascular pathway or suppression of extracellular glutamate in the central nervous system [2,3]. We aimed to investigate additional potential mechanisms of nVNS by evaluating resting-state electroencephalography (EEG) and trigeminal laser-evoked potentials (LEPs) in a subgroup of patients from PRESTO.

**Methods:** Patients from our site who were enrolled in PRESTO [1] and agreed to participate in this sub-study underwent both resting-state EEG and LEP recordings on the day of randomisation (T0) and during (T1) and after (T2) bilateral nVNS or sham stimulations. Power values for EEG frequencies between 2 and 100 Hz were measured. For the LEP recordings, 30-ms laser stimulations were applied to the right and left supraorbital zones, and the amplitudes and latencies (N1, N2, P2) and laser pain ratings were evaluated.

**Results:** Twenty patients from our PRESTO site had evaluable data (nVNS, n=10; sham, n=10). nVNS increased the slow delta-theta and alpha bands and slightly increased delta-theta and gamma power for the left vagal nerve. In the nVNS group, P2 amplitudes for the right and left trigeminal branches were smaller at T1 than at T0. Sham attenuated P2 amplitudes at both T1 and T2 for the left trigeminal branch, but these attenuations did not reach significance. Both nVNS and sham slightly reduced the N2 amplitude but did not affect N1 amplitude, latencies (N1, N2, P2), or laser pain ratings.

**Conclusions:** nVNS appears to act on cortical areas that are responsible for trigeminal pain control. Further EEG and LEP studies are warranted to identify potential clinical correlations.

**Acknowledgements**

PRESTO was sponsored by electroCore, LLC.

**Trial registration:** NCT02686034

**Ethics approval**

This sub-study was approved by the Ethical Committee of Bari Policlinico General Hospital.

**References:**

1. Tassorelli C, Grazzi L, de Tommaso M, et al. Non-invasive vagus nerve stimulation as acute therapy for migraine: the randomized PRESTO study. *Neurology*. In press.

2. Akerman S, Simon B, Romero-Reyes M. Vagus nerve stimulation suppresses acute noxious activation of trigeminocervical neurons in animal models of primary headache. *Neurobiol Dis*. 2017;102:96-104.

3. Oshinsky ML, Murphy AL, Hekierski H, Jr., Cooper M, Simon BJ. Noninvasive vagus nerve stimulation as treatment for trigeminal allodynia. *Pain*. 2014;155:1037-1042.

**Author Disclosures:**

**E. Vecchio** has nothing to disclose.

**I. Bassez** has nothing to disclose.

**K. Ricci** has nothing to disclose.

**C. Tassorelli** has received consultancy fees from Allergan S.p.A.; electroCore, LLC; Eli Lilly and Company; and Novartis AG and research grants from the European Commission and the Italian Ministry of Health. She is also a principal investigator or collaborator for RCTs sponsored by Alder BioPharmaceuticals Inc.; Eli Lilly and Company; and Teva Pharmaceutical Industries Ltd.

**E. Liebler** is an employee of electroCore, LLC, and receives stock ownership.

**M. de Tommaso** has received advisory fees from Allergan S.p.A.; Neopharmed; and Pfizer Inc.

### P145 Migraine, thrombophilic alterations, and vascular disease: results from a case-control study

#### Cinzia Cavestro^1^, Diana Degan^2^, GianmatteoMicca^3,4^, Raffaele Aloi^5^, Silvia Mandrino^1^, Carlo Di Pietrantonj^6^, Maria Cristina Frigeri^7^, Francesca Pistoia^2^, Filippo Molinari^3^, Simona Sacco^2^

##### ^1^Headache Center, Department of Neurology, ‘San Lazzaro’ Hospital, ASL CN2 Alba, Italy; ^2^Institute of Neurology, Department of Applied Clinical Sciences and Biotechnology, University of L’Aquila, L’Aquila, Italy; ^3^Main Laboratory and Hematology and Coagulation Disorders Laboratory, ASL CN2 Alba, Italy; ^4^Main Laboratory and Hematology and Coagulation Disorders Laboratory, ‘Santa Croce’ Hospital, Cuneo, Italy; ^5^Occupational Medicine Service, ASL CN2, Alba, Italy; ^6^Regional Service of Epidemiology, Alessandria, Italy; ^7^Administrative Medical Office, ‘San Lazzaro’ Hospital, ASL CN2, Alba, Italy

###### **Correspondence:** Cinzia Cavestro (cicaves@alice.it); Simona Sacco (simona.sacco@univaq.it)

**Background**. The association between migraine and increased risk of ischemic vascular events has been broadly investigated and supported [1]. Besides, available data indicate that migraine is comorbid with antiphospholipid antibodies (aPLs) positivity [2], whereas data referring to the comorbidity between migraine and other thrombophilic alterations are not conclusive. Accordingly, we investigated the association between migraine and thrombophilic alterations and examined if the presence of those alterations was associated with vascular events in migraine patients.

**Methods**. We designed a cross-sectional, case-control study. We included consecutive outpatients diagnosed with migraine referring to a tertiary Headache Center. Migraine patients were matched to headache-free control subjects. All participants were evaluated for free protein S anticoagulant, functional protein C anticoagulant, homocysteine, and antiphospholipid antibodies (aPLs). Past history of ischemic stroke (IS) or transient ischemic attack (TIA), coronary heart disease, and peripheral venous thrombosis was ascertained.

**Results**. We included 329 migraine patients and 329 control subjects (mean age 41 years, 77% women in both groups). As reported in Table 1, we identified an association of migraine with aPLs positivity (OR=2.6, 95% CI 1.5-4.7, p=0.001) and with free protein S deficiency (OR=4.7, 95% CI 1.6-14.0, p=0.002). We also found that in migraine patients, aPLs positivity was independently associated with both IS or TIA (OR=5.6, 95% CI 1.5-20.4, p=0.009) and coronary heart disease (OR=27.6, 95% CI 1.4-531.1, p=0.028) whereas free protein S deficiency was associated with IS or TIA only (OR=14.3, 95% CI 2.8-74.4, p=0.002).

**Discussion**. Our study provides some innovative results. To our best knowledge, the association between migraine and protein S deficiency was previously investigated by a single pilot studyinvolving migraine with aura participants only [3] that demonstrated a non-significant trend toward the association, possibly because of the low number of participants. We also found an association between migraine and aPLs positivity, in line with most of the available data.Our study was not adequately powered to assess the association between selected migraine subtypes (with and without aura, episodic and chronic) and thrombophilic alterations.

**Conclusion**. Our study indicates that migraine is comorbid with free protein S deficiency and aPLs positivity and that those factors are associated with the occurrence of vascular events in migraine patients, suggesting that in migraine-prone subjects, comorbid thrombophilic defects may favor migraine attacks occurrence and concur to ischemic events.

**Ethics Approval**

We obtained the approval of the Regional Health Office for targeted Research and local General Board Committee for this study (Protocol No 474281). Informed consent was obtained from all participants.

**References**

1. Sacco S, Ornello R, Ripa P, Tiseo C, Degan D, Pistoia F, Carolei A. Migraine and risk of ischaemic heart disease: a systematic review and meta-analysis of observational studies. Eur J Neurol 2015;22:1001-1011.

2. Cavestro C, Micca G, Molinari F, et al. Migraineurs show a high prevalence of antiphospholipid antibodies. J Thromb Haemost. 2011;9:1350-1354.

3. D'Amico D, Moschiano F, Leone M,Ariano C, Ciusano E, Erba N, Crazzi L, Ferraris A, Schieroni F, Bussone G. Genetic abnormalities of the protein C system: shared risk factors in young adults with migraine with aura and with ischemic stroke? Cephalalgia1998;18:618-621.Cephalalgia1998;18:618-621.

**Table 1 (abstract P145).** Risk of ischemic stroke or transient ischemic attack, heart disease, and peripheral venous thrombosis in migraine patients (multivariate analysis)

	IS or TIA			Coronary heart disease			Peripheral venous thrombosis		
	OR	95% CI	p	OR	95% CI	p	OR	95% CI	p
Age	1.0	0.9-1.0	0.187	-	-	-	1.0	0.9-1.0	0.516
Arterialhypertension	3.1	0.7-13.4	0.127	3.7	0.2-81.4	0.407	3.9	1.0-14.4	0.047
Diabetesmellitus	1.2	0.1-13.6	0.903	1.9	0.1-318.8	0.801	-	-	-
Hypercholesterolemia	1.4	0.3-6.9	0.705	8.0	0.4.174.2	0.187	-	-	-
BMI	0.9	0.8-1.0	0.041	0.8	0.5-1.1	0.141	0.9	0.8-1.0	0.080
aPLspositivity	5.6	1.5-20.4	0.009	27.6	1.4-531.3	0.028	3.4	0.9-12.3	0.063
Free protein S deficiency	14.3	2.8-74.4	0.002	6.2	0.1-1298.5	0.502	-	-	-

*Abbreviations*: *IS* ischemic stroke, *TIA* transient ischemic attack, *aPLs* antiphospholip antibodies, *BMI* body mass index, *OR* odds ratio, *CI* confidence interval

### P146 Healthcare Resource Utilisation in Patients Treated with OnabotulinumtoxinA for Chronic Migraine: The REPOSE study

#### Katja Kollewe^1^, Angela Antonakakis^2^, Katherine Sommer^3^, Justin S. Yu^4^

##### ^1^Hannover Medical School, Hannover, Germany; ^2^Schmerzzentrum Rhein-Main, Frankfurt, Germany; ^3^Allergn plc, Marlow, Buckinghamshire, UK; ^4^Allergan plc, Irvine, CA, USA

**Objective:** Chronic migraine (CM) has been associated with substantial disability, healthcare resource utilization (HRU), and economic burden. The objective of this analysis was to assess HRU in patients treated with onabotulinumtoxinA for CM in a European sample, and with a focus on German patients.

**Methods:** The REPOSE study is a 2-year multicentre (78 sites across 7 European countries), prospective, non-interventional, observational, open-label study, which aimed to describe the real-world use of onabotulinumtoxinA in adult patients with CM. Patients prescribed onabotulinumtoxinA for CM were enrolled and received onabotulinumtoxinA approximately every 12 weeks according to the physician’s discretion, and guided by the Summary of Product Characteristics. HRU, including visits to a healthcare professional (HCP) for any reason, accident and emergency (A&E) visits for any reason, and hospital admittance for headache, were collected at baseline (occurrence within the previous 3 months) and at each treatment session (since the last visit).

**Results:** In total, 641 patients were enrolled in this study, with 633 patients receiving ≥1 dose of onabotulinumtoxinA for CM. Patients were on average 45 years of age, 85% female, and 60% of patients (n=377) were from Germany. Among all patients, HCP visits at baseline were recorded in 45.8% of patients, with reductions from baseline observed at each follow-up session (range: 12.5-20.8%).

Similarly, A&E visits (baseline: 6.3% of patients) and hospital admittance for headache (6.0%) were reduced compared to baseline at each follow-up session (A&E range: 1.0-2.4%; hospital admittance range: 0.4-1.7%). Within the German subgroup, similar trends were observed, where HCP visits decreased from baseline (35.8% of patients) at each subsequent treatment session (range: 13.4-7.4%). In addition, A&E visits (baseline: 2.1% of patients) and hospital admittance for headache (baseline: 4.2%) were reduced from baseline at each follow-up session (A&E range: 0.0-0.8%; hospital admittance range: 0.7-2.1%).

**Conclusions:** Real-world findings from REPOSE demonstrate that treatment of CM with onabotulinumtoxinA is associated with a reduction in HRU, including HCP visits (for any reason), A&E visits (for any reason), and hospital admittance (for headache). Similar trends in HRU reductions were observed between the total sample and the German subgroup. Combined, these data support the long- term benefits associated with the use of onabotulinumtoxinA for CM in clinical practice.

**Declarations:** KK has received travel grants and honoraria for lectures from Allergan, Ipsen, Merz, and Biogen. AA has no disclosures. KS and JSY are employees of Allergan plc and receives stock or stock options.

### P147 Proposed new diagnostic criteria for chronic migraine

#### Mona Ameri Chalmer^1^, Thomas Folkmann Hansen^1^, Elena Lebedeva^2^, David Dodick^3^, Jes Olesen^1^

##### ^1^Department of Neurology, Danish Headache Center, Copenhagen University Hospital, Glostrup, Denmark; ^2^Department of Neurology and Neurosurgery, Urals State Medical University, Russia; ^3^Mayo Clinic, Scottsdale, AZ, USA

###### **Correspondence:** Mona Ameri Chalmer (mona.ameri.chalmer@regionh.dk)

**Introduction**

There are a number of weaknesses with the current diagnostic criteria for chronic migraine (CM): The criteria are complex, they include a mixture of migraine and tension-type-like headaches, and they do not account for patients who have a high frequency of migraine but no other headaches. We suggest classifying CM as simply ≥ 8 migraine days per month disregarding the request for ≥ 15 days with any kind of headache per month to better reflect the suffering of the patients.

**Materials and methods**

We compared patients with CM according to the ICHD-3 criteria[1] with patients who had ≥ 8 or more migraine days per month but not 15 days with headache (proposed CM-CM). Patients were recruited from tertiary referral clinics in Denmark and the United States as well as a student population from Russia. All were carefully phenotyped. Furthermore, data from extensive Danish registries were used to compare the socioeconomic impact of chronic migraine in these two groups.

**Results**

The number of migraine patients with proposed CM-CM was almost two fold higher in all three datasets (Table 1). There was no significant difference in the demographic profile between the patients in the two groups. No difference between the number of lifelong attacks (p>0.3) and number of attacks in the previous year. The prevalence of comorbidities was the same for the two groups. Proposed CM-CM patients purchased more triptans than CM patients (p=0.01) (Fig. 1). The self-reported effect of triptans was the same for the two groups (p=1). There was no difference in early pension (p>0.1) or sickness benefit (p>0.2) between the two groups from 1987-2007 (Table 2).

**Conclusions**

Patients who suffer from ≥ 8 migraine days per month but not 15 days with headache do not differ from patients with CM in their demographic profile, attack frequency, comorbidities, self-reported effect of triptans and prophylactic drugs; they tend to even purchase more triptans than CM patients and are equally as disabled by their migraine attacks measured on socioeconomic parameters such as early pension and sickness benefit. We propose to classify CM as simply ≥ 8 migraine days per month and to disregard the request for ≥ 15 days with any kind of headache per month. This will better reflect the suffering of the patients; make more and newer drugs available for this severely affected group of patients; make the diagnostic criteria simpler; and finally create a more homogenous patient group for clinical trials.

**Ethics Approval**

Our research group has permissions and approval from the Data Protection Agency (GLO-2010-10) and the Ethical Committee (H-2-2010-122).

**References**

1. (2018) Headache Classification Committee of the International Headache Society (IHS) The International Classification of Headache Disorders, 3rd edition. Cephalalgia 38:1–211. doi: 10.1177/0333102417738202

**Table 1 (abstract P147).** Number of migraine patients

	Danish Headache Center	The Mayo Clinic	Russian students
CM	174	T.b.c.	17
pCM	350	T.b.c.	46
pCM-CM	176	T.b.c.	29

*pCM* proposed chronic migraine, *CM* chronic migraine, *T.b.c* To be collected

**Table 2 (abstract P147).** Social parameters 1987-2007

	CM	pCM-CM
Early retirement pension	17 (12.6%)	15 (10.6%)
Sickness benefit	113 (83.7%)	113 (80.1%)

*pCM* proposed chronic migraine, *CM* chronic migraine

**Fig. 1 (abstract P147).**
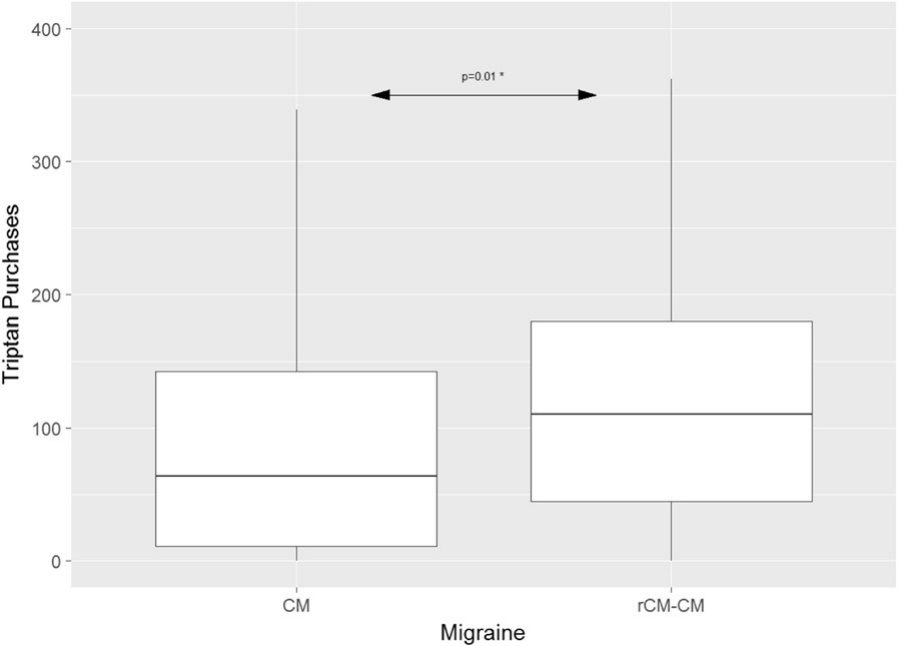
Distribution of triptan purchases. pCM: proposed chronic migraine, CM: chronic migraine

### P148 Safety and efficacy of cervical 10 kHz cervical spinal cord stimulation (SCS) for the management of refractory chronic migraine: a prospective, proof-of-concept open label study

#### Lambru Giorgio^1^, Al-Kaisy Adnan^1,2^, Palmisani Stefano^1,2^, Carganillo Roy^2^, Wesley Samuel^2^, Pang David^2^

##### ^1^The Headache Centre, Guy’s and St Thomas’ NHS Foundation Trust, London, UK; ^2^Pain and Neuromodulation Academic Research Centre, Guy’s and St Thomas’ NHS Foundation Trust, London, UK

###### **Correspondence:** Lambru Giorgio (giorgiolambru@gmail.com)

**Summary**

There is a significant unmet need for safe and effective therapies for the management of refractory chronic migraine. Neurostimulation therapies targeting peripheral structures, such as occipital nerve stimulation showed disappointing therapeutic effects in large clinical trials [1]. High frequency (10 kHz) spinal cord stimulation (HF-SCS) has shown promising efficacy in a recent prospective study conducted in subjects with chronic migraine and medication overuse [2].

**Aim of the investigation**

The aim of this study was to assess safety, tolerability and efficacy of 10 kHz cervical spinal cord stimulation (SCS) in chronic migraine subjects refractory to conventional medical therapies. The study design is a single-center, open label, prospective, feasibility study.

**Methods**

Twenty adults diagnosed with refractory chronic migraine were enrolled. Medication overuse headache and severe depression were considered exclusion criteria. All subjects were implanted with spinal cord stimulator (Senza System, Nevro Corp, Redwood City, CA); no stimulation trial was performed. Stimulating leads were positioned in the epidural space with the distal tip at the C2 vertebral level. All subjects completed a daily headache diary and headache-related disability scores for the full duration of the study. Safety and effectiveness outcomes, including change in headache days, migraine days, responder rate, change in abortive treatments intake and change in quality of life measures were captured up to 12 months post-device activation. Here we present the interim six month results.

**Results**

Baseline data was available for 19 subjects (43 ± 10 years; 84% female), who had failed an average of 11.7 ± 3.2 preventive treatments at the time of recruitment. The average number of headache days at baseline was 23.3 ± 5.2 days of which 21.6 ± 6.6 days were migraine days. The average migraine-specific quality of life (MSQ) score at baseline was 32.0 ± 15.7. Compared to baseline, the average reduction in headache days at 12 weeks was 3.9 days, which increased to 4.9 days at week 24. The average reduction in migraine days at 12 weeks was 6.2 days/month, which increased to 7.2 days at weeks 24. Thirty-eight percent of the subjects at week 12 and 43% at week 24 obtained a 30% reduction in headache days. Forty-four percent of subjects at week 12 and 50% of subjects at week 24 reported a reduction of more than 30% in migraine days compared to baseline. In 50% of the subjects, the chronic migraine reverted to being episodic in pattern (<15 headache days/month) at week 24. We observed an improvement in the MSQ score of an average of 17.2 points at 24 weeks (50.8 ± 23.4). At week 24, five subjects reported pain at the site of implant, four reported musculoskeletal pain in the cervical and shoulder area, one experienced slight lead movement. No subjects required any further surgical procedure.

**Conclusion**

This interim analysis suggests that HF-SCS may offer a potentially safe and effective therapeutic option for medically refractory chronic migraine. These efficacy outcomes seem similar to those reported for ONS. However the population treated in this trial was significantly more refractory than the one included in the ONS studies. Furthermore, the lack of implant-related additional surgery at six month follow-up suggests that this therapy may offer a safer profile to ONS.

**References**

1. Dodick DW, Silberstein SD, Reed KL et al (2015) Safety and efficacy of peripheral nerve stimulation of the occipital nerves for the management of chronic migraine: long-term results from a randomized, multicenter, double-blinded, controlled study. Cephalalgia 35:344–358

2. Arcioni R, Palmisani S, Mercieri M et al (2016) Cervical 10 kHz spinal cord stimulation in the management of chronic, medically refractory migraine: A prospective, open-label, exploratory study. Eur J Pain 20:70–78

### P149 Is pituitary screening necessary in cluster headache?

#### Lou Grangeon^1,2^, Emer O’Connor^1^, Daisuke Danno^1^, Thanh Mai Pham Ngoc^3^, Indran Davagnanam^4^, Manjit Matharu^1^

##### ^1^Headache Group, UCL Institute of Neurology and The National Hospital for Neurology and Neurosurgery, Queen Square, London; ^2^Department of Neurology, Rouen University Hospital, 76031, Rouen, France; ^3^Mathematics Institute of Orsay, Paris Sud University, CNRS, 91405 Orsay, France; ^4^Department of Brain Repair and Rehabilitation, UCL Institute of Neurology, Queen Square, London

###### **Correspondence:** Manjit Matharu (m.matharu@uclmail.net)

**Background:**

Cluster headache (CH), one of the most painful conditions known to humans, can occur secondary to pituitary disease. To date, it remains unclear as to whether a higher prevalence of pituitary tumours exists in CH patients and, as a result, if pituitary imaging is required in the diagnostic assessment of CH patients. The aim of this study was to determine the incidence of pituitary adenomas in CH patients and to identify clinical predictors of pituitary adenomas in CH patients.

**Methods:**

A retrospective study was conducted of all consecutive patients diagnosed with CH between 2007 and 2017 in a headache center. Data including demographics, attack characteristics, response to treatments and routine pituitary function tests were recorded. Univariate and multivariate analysis using random forests were used to analyse the data.

**Results:**

718 CH patients attended the headache clinic; 643 underwent a standard MRI scan of whom 376 also underwent a dedicated pituitary MRI. Pituitary adenomas occurred in 17 of 376 patients (4.52%). Non-functioning microadenomas (n=13) were the most common abnormality reported. Two patients, one of whom lacked the symptoms of pituitary disease, required treatment for their pituitary lesion. No statistical difference was found between patients with pituitary adenoma and with normal pituitary MRI in terms of demographic, clinical characteristic or response to treatment. Systematic pituitary MRI scanning only benefited a single patient in the entire cohort.

**Conclusion:**

The incidence of pituitary adenomas in CH is similar to that reported in the general population thereby precluding an over-representation of pituitary lesions in CH. We conclude that the diagnostic assessment of CH patients should not include routine pituitary screening. Only patients with standard brain MRI findings or symptoms suggestive of a pituitary disorder require pituitary imaging.

**Classification of evidence:**

This study provides Class IV evidence that routine dedicated pituitary MRI scans are not indicated in CH patients

**Fig. 1 (abstract P149).**
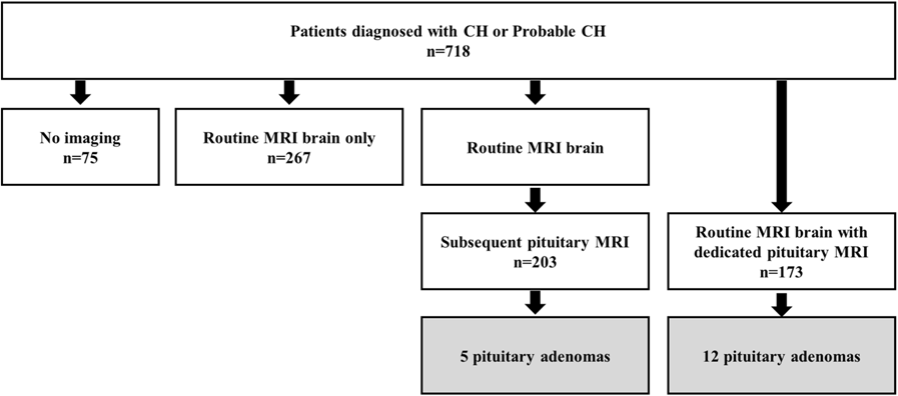
Flow diagram showing patient disposition throughout the trial

**Fig. 2 (abstract P149).**
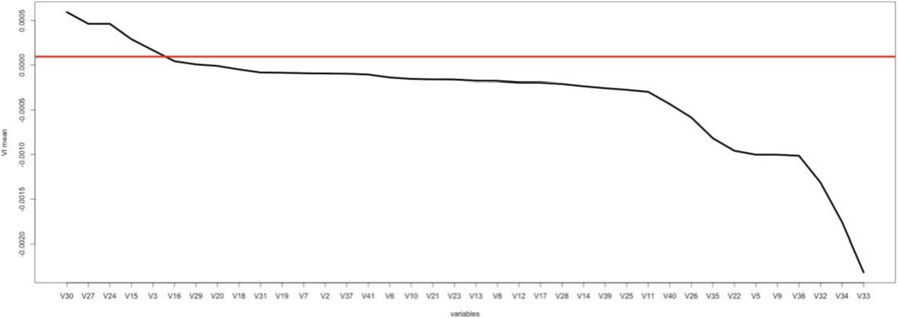
Multivariate analysis using random forest showing sorted variable importance (VI) mean VI (on the X-axis) of all variables recorded , in descending order (on the y-axis). The red line is the threshold value. Only five items were above the line but with minor significance, of no clinical relevance (VI mean < 0.002). *Variables legend: V2: Current age, V3: Gender, V5: Age of Onset, V6: Episodic CH, V7: Chronic CH, V8: Probable CH , V9: Strictly Unilateral, V10: Side Variable, V11: Bilateral, V12: Site RetroOrbital,V13: Site Orbital, V14: Site Frontal, V15: Site Temporal, V16: Site Parietal, V17: Site Vertex, V18: Site Occiput, V19: Site Nasal, V20: Absence of dysautonomic feature, V21: Ptosis, V22: Eye Oedema, V23: Conjonctival injection, V24: Miosis, V25: Lacrimation, V26: Nasal Blockage, V27: Rhinorrea, V28: Sweating, V29: Facial Flush, V30: Aural Fullness, V31: Restlessness, V32: Average attack frequency per day, V33: Minimum attack frequency per day V34: Maximum attack frequency per day, V35: Average Attack Duration, V36: Minimum Attack Duration, V37: Maximum Attack Duration, V39: Intractable to Acute treatment, V40: Intractable to Preventive treatment, V41: Follow-up duration*

### P150 The impact on physician prescribing of monthly and quarterly dosing options for a new class of migraine preventive therapy

#### Robert Cowan^1^, Erik Rosenman^3^, Tim Fitzgerald^2^, Joshua Cohen^2^, Ravi Iyer^2^

##### ^1^Teva Pharmaceutical Industries, Frazer, Pennsylvania, 19355, USA; ^2^Neurology, Stanford University School of Medicine, Stanford, California, 94305, USA; ^3^IQVIA, Inc., Cambridge, Massachusetts, 02139, USA

###### **Correspondence:** Robert Cowan

**Background**

Migraine affects approximately 39 million people in the US^1^. A new class of migraine preventive therapy launching in 2018-2019 will provide physicians and patients with an alternate approach to preventive migraine treatment. This study sought to understand the impact, if any, of having both monthly and quarterly dosing options available on physician intent to prescribe this new class of migraine preventive therapy.

**Methods**

In this double-blind, observational study, 406 US physicians currently treating adult migraine patients completed a 20-minute online survey. Respondents were selected using customized screening criteria and included 166 general practice, internal medicine or family practice physicians, 95 general neurologists, 85 pain specialists, and 60 headache specialists. Physicians allocated the proportion of their migraine patients who would receive preventive therapies, including the new class of migraine preventive therapy, in each of three scenarios: 1) only monthly dosing of the new class of therapy is available, 2) only quarterly dosing is available, and 3) both monthly and quarterly dosing are available. In each scenario, physicians provided the percent of patients who would likely receive each type of preventive therapy across three migraine patient types: moderate-frequency episodic (5-9 headache days / month), high-frequency episodic (10-14 headache days / month), and chronic (≥15 headache days / month). In the survey, receiving a therapy was defined as physicians prescribing the therapy and patients accepting (filling) the prescription. Physicians reviewed scenarios in a randomized order. Data analysis included descriptive statistical analyses and ANOVAs, assuming significance at p<0.05.

**Results**

The availability of both monthly and quarterly dosing yielded a statistically significant increase (p<0.001) in the proportion of patients expected to receive the new class of migraine preventive therapy: moderate-frequency episodic patients were more likely to receive the new class of therapy when both dosing options are available (35.3%) than when monthly only (26.1%) or quarterly only (25.8%) is available; high-frequency episodic patients were more likely to receive the new class of therapy when both dosing options are available (42.9%) than when monthly only (33.9%) or quarterly only (33.5%) is available; and chronic patients were more likely to receive the new class of therapy when both dosing options are available (49.1%) than when monthly only (39.6%) or quarterly only (39.3%) is available.

**Conclusions**

Physicians showed equal preference for monthly and quarterly dosing options, while the availability of both dosing options increased the proportion of patients likely to receive this new class of migraine preventive therapy.

**References**

1. http://migraineresearchfoundation.org/about-migraine/migraine-facts/ Last accessed June 22^nd^, 2018

### P151 A multicentre, double-blind, randomised, sham-controlled study of non-invasive vagus nerve stimulation (nVNS) for the preventive treatment of episodic migraine: the PREMIUM trial

#### Hans-Christoph Diener^1^, Peter J. Goadsby^2^, Messoud Ashina^3^, Mohammad Al-Mahdi Al-Karagholi^3^, Alexandra Sinclair^4^, Dimos Mitsikostas^5^, Delphine Magis^6^, Patricia Pozo-Rosich^7,8^, Pablo Irimia Sieira^9^, Miguel J.A. Làinez^10^, Charly Gaul^11^, Nicholas Silver^12^, Jan Hoffmann^13^, Eric Liebler^14^, Michel D. Ferrari^15^

##### ^1^Klinik für Neurologie, Universitätsklinikum Essen, Essen, Germany; ^2^NIHR-Wellcome Trust King's Clinical Research Facility, King's College London, UK; ^3^Danish Headache Center, Rigshospitalet Glostrup, University of Copenhagen, Denmark; ^4^Metabolic Neurology, Institute of Metabolism and Systems Research, University of Birmingham, Birmingham, UK; ^5^1st Neurology Department, Aeginition Hospital, National and Kapodistrian University of Athens, Athens, Greece; ^6^CHR de la Citadelle, University of Liège, Liège, Belgium; ^7^Headache Clinic, Neurology Department, Hospital Universitari Vall d'Hebron, Barcelona, Spain; ^8^Headache Research Group, Vall d’Hebron Research Institute, Universitat Autonoma de Barcelona, Barcelona, Spain; ^9^Clinica Universidad de Navarra, Pamplona, Spain; ^10^Catholic University of Valencia, University Clinic Hospital, Valencia, Spain; ^11^Migraine and Headache Clinic, Königstein, Germany; ^12^The Walton Centre, Liverpool, UK; ^13^Department of Systems Neuroscience, University Medical Center Hamburg-Eppendorf, Hamburg, Germany; ^14^electroCore, LLC, Basking Ridge, New Jersey, USA; ^15^Leiden University Center, Leiden, The Netherlands

###### **Correspondence:** Hans-Christoph Diener (h.diener@uni-essen.de)

**Background:** Novel preventive migraine therapies such as non-invasive vagus nerve stimulation (nVNS; gammaCore®) would be welcome due to the low adherence, adverse events (AEs), and limited efficacy of traditional options. The aim of the randomised, double-blind, sham-controlled PREMIUM trial was to evaluate the efficacy, safety, and tolerability of preventive nVNS for episodic migraine.

**Methods:** In PREMIUM, patients from 22 European sites entered a 4-week observational run-in period (no study treatment), a 12-week double-blind period of randomised nVNS or sham, and a 24-week open-label period of nVNS. Patients were instructed to administer two 120-second stimulations bilaterally to the neck 3 times daily (TID). Abortive migraine medication was permitted if needed, but adjunctive preventive migraine medication was not allowed until week 24. The *intent-to-treat (ITT) population*, defined as enrolled patients who received ≥1 treatment in the double-blind period, was the primary analysis set for efficacy. Upon observation of suboptimal rates of adherence to the TID treatment protocol in the ITT population, a *modified intent-to-treat (mITT) population* was defined as those with >67% adherence per month for evaluation in a post hoc analysis. Safety was evaluated in all enrolled patients.

**Results:** Mean reductions in number of migraine days from the run-in period to the last 4 weeks of the double-blind period (primary end point) were –2.26 days (nVNS; n=165) and –1.80 days (sham; n=167) (*p*=0.146). Percentages of patients with a ≥50% reduction in number of migraine days were 31.9% (nVNS) and 25.0% (sham) (*p*=0.186). Findings were similar for headache and acute medication days. For the mITT population, significant benefits of nVNS (n=138) vs sham (n=140) were seen for reductions in number of migraine days (–2.27 vs –1.53 days; *p*=0.043), headache days (–2.85 vs –1.99 days; *p*=0.045), and acute medication days (–1.94 vs –1.14 days; *p*=0.039). Device-related AEs in the nVNS group were mostly mild, with application site pain being the most common.

**Conclusions:** nVNS demonstrated consistent but nonsignificant preventive benefits over sham in episodic migraine, with sham providing a higher response than anticipated. Patients with >67% adherence experienced statistically significant benefits of nVNS. The role of adherence and the sham response warrants further evaluation to better understand the clinical utility of preventive nVNS for migraine.

**Acknowledgements**

PREMIUM was sponsored by electroCore, LLC. We present this abstract on behalf of the PREMIUM Study Group.

**Trial registration:** NCT02378844

**Ethics approval**

PREMIUM was approved by the local ethics committee for each study site.

**Author Disclosures:**

**H.-C. Diener** has received honoraria for participation in clinical trials and for contributions to advisory boards and oral presentations sponsored by 3M Medica; Addex Pharma; Adler; Allergan; Almirall; Amgen; AstraZeneca; Autonomic Technologies; Bayer; Berlin-Chemie; Boehringer Ingelheim; Bristol-Myers Squibb; Chordate Medical; Coherex Medical; CoLucid Pharmaceuticals; electroCore, LLC; Eli Lilly and Company; GlaxoSmithKline; Grünenthal; Janssen-Cilag; Johnson & Johnson; Labrys Biologics; La Roche; Medtronic; Menarini; Minster Pharmaceuticals; MSD; NeuroScore; Novartis; Pfizer; Pierre Fabre; Sanofi; Schaper and Brümmer; St. Jude Medical; Vital; and Weber & Weber. Dr. Diener has also received research funding from Allergan; Almirall; AstraZeneca; Bayer; electroCore, LLC; GlaxoSmithKline; Janssen-Cilag; MSD; and Pfizer. He has received additional research support from the European Union; the German Ministry of Education and Research; and the German Research Council. Dr. Diener has no ownership interests and does not own any pharmaceutical company stocks.

**P.J. Goadsby** has received grants and personal fees from Allergan; Amgen; and Eli Lilly and Company. He has also received personal fees from Akita Biomedical; Alder Biopharmaceuticals; Autonomic Technologies; Avanir Pharmaceuticals; Cipla Ltd; CoLucid Pharmaceuticals, Inc.; Dr. Reddy's Laboratories; electroCore, LLC; eNeura; Journal Watch; *Medico-Legal Journal*; Novartis; Oxford University Press; Pfizer Inc; Promius Pharma; Quest Diagnostics; Scion; Teva Pharmaceuticals; Trigemina, Inc.; and Up-to-Date. In addition, Dr. Goadsby has a patent for magnetic stimulation for headache pending assigned to eNeura.

**M. Ashina** has received honoraria for contributions to advisory boards and oral presentations sponsored by Allergan; Alder; Amgen; Eli Lilly and Company; Novartis; and Teva Pharmaceuticals. Dr. Ashina has been the primary investigator for the Amgen 20120178 (Phase 2), 20120295 (Phase 2), 20130255 (OLE), and 20120297 (Phase 3) trials and for the Alder ALD403-CLIN-001 (Phase 3), Amgen PAC1 20150308 (Phase 2a), and electroCore GM-11 trials. Dr. Ashina has no ownership interests and does not own any pharmaceutical company stocks.

**M. Al-Mahdi Al-Karagholi** has received travel grants from electroCore, LLC.

**A. Sinclair** is funded by an NIHR Clinician Scientist Fellowship (NIHR-CS-011-028) and by the Medical Research Council, UK (MR/K015184/1).

**D. Mitsikostas** has received advisory fees, honoraria, research grants, or travel grants from Allergan; Amgen; Biogen; Cefaly; electroCore, LLC; Eli Lilly and Company; Merz Pharma; Novartis; Roche; Sanofi Genzyme; and Teva Pharmaceuticals.

**D. Magis** has received travel grants from electroCore, LLC; is a consultant for Novartis Belgium; and is an associate editor for *Cephalalgia*.

**P. Pozo-Rosich** has received honoraria as a consultant and speaker for Allergan; Almirall; Chiesi; Eli Lilly and Company; Novartis; and Teva Pharmaceuticals. Dr. Pozo-Rosich does not own stocks from any pharmaceutical company.

**P. Irimia Sieira** has received honoraria from Allergan; Novartis; and Teva Pharmaceuticals. Dr. Irimia has no ownership interests and does not own any pharmaceutical company stocks.

**M.J.A. Làinez** has received advisory fees, speaker fees, research grants, and/or research support from ATI Pharma; Allergan; Amgen; Boehringer Ingelheim; electroCore, LLC; Eli Lilly and Company; Lupin Pharmaceuticals; Medtronic; Novartis; Otsuka; Roche; and Teva Pharmaceuticals.

**C. Gaul** has received honoraria from Allergan; Bayer; Boehringer Ingelheim; Desitin Arzneimittel; electroCore, LLC; Eli Lilly and Company; Grünenthal; Hormosan Pharma; Novartis; Ratiopharm; Reckitt Benckiser Group; and Teva Pharmaceuticals. He has no ownership interests and does not own any pharmaceutical company stocks.

**N. Silver** has received honoraria from Allergan; electroCore, LLC; Eli Lilly and Company; Novartis; and Teva Pharmaceuticals and investigator fees paid to the Walton Centre.

**J. Hoffmann** has consulted for and/or served on advisory boards for Allergan; Autonomic Technologies Inc. (ATI); Chordate Medical AB; Hormosan Pharma; Novartis; and Teva Pharmaceuticals. He received honoraria for speaking from Allergan; Chordate Medical AB; Novartis; and Teva Pharmaceuticals.

**E. Liebler** is an employee of electroCore, LLC, and receives stock ownership.

**M.D. Ferrari** has received consultancy fees from Medtronic and research support from the Netherlands Organisation for Scientific Research (NWO); the European Community; ZonMw; and the Dutch Heart Foundation. Dr. Ferrari is a member of the Editorial Board for *Cephalalgia*.

### P152 Post hoc analysis of the effects of more frequent non-invasive vagus nerve stimulation (nVNS) for patients with chronic cluster headache from the PREVA trial

#### Charly Gaul^1^, Eric Liebler^2^, Candace McClure^3^, Annelie Andersson^2^, Andreas Straube^4^

##### ^1^Migraine and Headache Clinic, Königstein, Germany; ^2^electroCore, LLC, Basking Ridge, NJ, USA; ^3^North American Science Associates Inc., Minneapolis, MN, USA; ^4^Ludwig Maximilian University of Munich, Munich, Germany

###### **Correspondence:** Charly Gaul (c.gaul@migraene-klinik.de)

**Background:** The randomised controlled PREVA trial demonstrated that non-invasive vagus nerve stimulation plus standard of care (nVNS+SoC) significantly decreased the number of weekly attacks compared with SoC alone in patients with chronic cluster headache [1]. We conducted a post hoc analysis of the effects of more frequent nVNS use (through optional complementary acute treatment) in enhancing the preventive effect of nVNS.

**Methods:** In PREVA [1], the nVNS+SoC group received preventive nVNS; 3 additional stimulations per attack were permitted as acute treatment. The median percentage of attacks per patient that were acutely treated was 76.9%, and the median average number of daily acute stimulations per patient was 8.2. The mean reduction in weekly attack frequency from baseline to the randomised period was compared for subgroups that fell above and below these median values and the SoC-alone group using linear regression with the Bonferroni method to adjust for multiple comparisons.

**Results:** Acute nVNS was reported for 1138/1673 attacks (68%) in the randomised period. The average decrease in weekly attack frequency was significantly greater for patients who used acute nVNS for ≥76.9% of their attacks (–8.5 attacks/week; n=22) than for the SoC-alone group (–2.1 attacks/week; n=47; *p*<0.01). Those who used nVNS for <76.9% of their attacks had a less-pronounced decrease (mean, –3.7 attacks/week; n=22; *p*=1.00). The mean reduction in weekly attack frequency was greater for patients who used ≥8.2 daily stimulations (–6.3 attacks/week; n=23) than for those who used <8.2 daily stimulations (–5.9 attacks/week; n=21); differences vs the SoC-alone group were not significant after adjustment for multiple comparisons (*p*=0.15 and *p*=0.25, respectively).

**Conclusions:** More frequent nVNS use (for ≥76.9% of attacks) provided a significantly better response than less frequent use. In this analysis, the effects of nVNS on attack prevention appeared to be more closely related to the frequent treatment of attacks than to the use of more (≥8.2) stimulations per day. These findings support more frequent acute nVNS treatment of attacks to enhance the preventive effects of nVNS on chronic cluster headache and that a dose-response analysis may be warranted.

**Acknowledgements**

PREVA was sponsored by electroCore, LLC.

**Trial registration:** NCT01701245

**Ethics approval**

PREVA was approved by the local ethics committee for each study site.

**Reference**

1. Gaul C, Diener H-C, Silver N, et al. Non-invasive vagus nerve stimulation for PREVention and Acute treatment of chronic cluster headache (PREVA): a randomised controlled study. *Cephalalgia*. 2016;36(6):534-546.

**Author Disclosures:**

**C. Gaul** has received honoraria from Allergan plc; Bayer AG; Boehringer Ingelheim GmbH; Desitin Arzneimittel GmbH; electroCore, LLC; Eli Lilly and Company; Grünenthal GmbH; Hormosan Pharma GmbH; Novartis Pharma AG; Ratiopharm GmbH; and Reckitt Benckiser Group plc. He has no ownership interests and does not own any pharmaceutical company stocks.

**E. Liebler** is an employee of electroCore, LLC, and receives stock ownership.

**C. McClure** is an employee of North American Science Associates Inc.

**A. Andersson** is an employee of electroCore, LLC, and receives stock ownership.

**A. Straube** has received honoraria from Allergan plc; Boehringer Ingelheim GmbH; Desitin Arzneimittel GmbH; electroCore, LLC; Hormosan Pharma GmbH; Novartis Pharma AG; and Teva GmbH. He has also received research grants from the Else Kröner-Fresenius Foundation; the German Council of Science and Humanities; the German Secretary of Education; and the Ludwig Maximilian University.

### P153 IMPACT OF MEDICAL CARE ON SYMPTOMATIC DRUG CONSUMPTION AND QUALITY OF LIFE IN HEADACHE: a one-year population study

#### Antonaci F^1^, Cotta Ramusino M^1^, Perini G^1^, Vanacore N^2^, Costa A^1^

##### ^1^Department of Brain and Behavioral Sciences, C. Mondino National Institute of Neurology Foundation, IRCCS, University of Pavia, Pavia, Italy; ^2^National Centre for Epidemiology, Surveillance, and Health Promotion, National Institute of Health, Rome, Italy

###### **Correspondence:** Antonaci F (fabio.antonaci@unipv.it)

**Background**: Headache is one of the most common painful syndrome and can be responsible for high disability. It is a widespread disorder both in episodic and chronic form. Chronic headache often leads to a high use/overuse of symptomatic drugs (1); indeed, medication overuse headache (MOH) occurs in over half of chronic headache patients (2), with significant management difficulties.

**Objective**: to provide data about symptomatic drug (NSAIDs and triptans) consumption in an outpatient population of the Health District of Pavia, and describe how the clinical picture may change after being taken over by headache experts.

**Materials and Methods:** 276 patients using symptomatic drug for headache were recruited in 32 pharmacies. A telephonic interview was carried out in 199 of them. Data collection included sociodemographic characteristics and features of headache and drug consumption/abuse. Patients underwent 4 visits: a baseline visit (T0) and 3 follow-up visits performed by a neurologist at 3, 6 and 12 months (T3, T6 and T12, respectively). During each visit, patients underwent a complete neurological assessment and received therapeutic adjustments aimed at obtaining a proper management of headache.

**Results**: Patients with chronic migraine or MOH were only 8% at the telephonic interview compared to 17% at the frontal visit (T0). After 12 months of follow-up, we observed a significant decrease in the frequency of attacks (T0: 8.5±8.8/month vs T12: 2.1±1.8/month; p<0.01), and a concomitant reduction of the days/month of headache (T0: 10.7±9.0 vs T12: 3.6±4.6; p<0.01). With respect to the single attacks, a decrease in the duration was reported (T0: 33.8±30.2 hrs vs T12: 9.3±18.5 hrs; p<0.001) , while pain intensity remained unchanged (T1: 4.9±2.3 vs T12: 4.3±2.9 NRS). The improvement of headache management resulted in both a significant decrease in the analgesic consumption per month (T0: 11.6±16.4 vs T12: 3.8±5.9 doses/month; p<0.01), and an increase in the quality of life, scored by MIDAS (T0: 22.6±29.3 vs T12: 16.8±35.1; p<0.05), and in the self-rated quality of the received treatment, scored by HURT (T0: 9.7±5.0 vs T12: 5.5±5.5; p<0.05).

**Conclusion**: This study shows that a proper medical management is more effective than self-treatment of headache, resulting in lower disability and improved quality of life within a few months from taking-over by headache specialists.

RC MINSAL 2013-2015 IRCCS MONDINO

**References:**

(1) Ghiotto N, et al. Medication overuse headache and applicability of the ICHD-II diagnostic criteria: 1-year follow-up study (CARE I protocol). Cephalalgia. 2009; 29(2):233-43.

(2) Westergaard ML, et al. Prevalence of chronic headache with and without medication overuse: associations with socioeconomic position and physical and mental health status. Pain. 2014 Oct;155(10):2005-13.

### P154 Habituation determined by algometry as a marker of response in Chronic Migraine

#### García-Azorín D, Ruiz-Piñero M, Sierra A, Martinez B, Guerrero Peral AL

##### Headache Unit, Clinico University Hospital. 47005. Valladolid, Spain

###### **Correspondence:** García-Azorín D (davilink@hotmail.com)

**INTRODUCTION**:

Lack of habituation is one of the pathophysiological hallmarks of chronic migraine, nevertheless it has not been used in clinical practice to date, being determined in the experimental setting with neurophysiological parameters. Pressure pain thresholds change in repeated measures can be used to demonstrate habituation, showing increased threshold in the healthy population and a lack of habituation in migraneurs. We aim to evaluate if the normalization of the pattern is associated with clinical improvement.

**METHODS**:

We conducted a prospective study in patients with Chronic Migraine (CM) diagnosis according to International Headache Society criteria. We included patients with indication to receive type A Onabotulinumtoxin (OnabotA) according to national guidelines. We conducted a pressure pain threshold (PPT) determination with algometer in 21 cranial points according to 10:20 system and in 12 additional extra cranial points. We took 3 consecutive measures with a 1 second interval, evaluating if subsequent determinations showed a higher or a lower value, showing habituation or lack of habituation. We studied patients prior to the administration of OnabotA and one month after, including clinical and sociodemographic variables.

**RESULTS**:

We included 49 patients, 91,8% of them female. Mean age was 43,8 and mean age of onset of migraine was 19,1. They suffered chronic migraine since a mean of 38,73 months at the moment of OnabotA treatment. Only 8,1% patients showed habituation in the basal evaluation, being present in 34,7% after OnabotA administration. We did not find differences in number of migraine days, analgesic or triptan uptake in the basal evaluation. Patients that showed habituation had a higher reduction in migraine days (12,9 vs 7,1, p=0,08) and analgesic consumption (11,6 vs 4,1, p=0,03). Patients that showed clinical improvement after OnabotA treatment showed an increase of average PPT (+0,15 vs -0,03, p=0,05).

**CONCLUSIONS**:

Presence of habituation after OnabotA treatment was associated with clinical improvement in CM patients. Pressure pain threshold determination with algometer could be considered in clinical practice as a potential biomarker of response.

### P155 Study on the phenotypic characterization of delayed alcohol induced headache: series of 1184 sufferers

#### García-Azorín D, Aparicio Cordero L, Sierra Mencía A, Guerrero Peral AL.

##### Headache Unit, Hospital Clínico Universitario, 47005. Valladolid, Spain.

###### **Correspondence:** García-Azorín D (davilink@hotmail.com)

**INTRODUCTION**:

Delayed alcohol induced headache is one of the commonest secondary headaches[1]. Its phenotype has not been studied so far. Many pathophysiological mechanisms have been proposed, but this is not yet clarified [2, 3]. We aimed to describe its phenotype and to correlate it with pathophysiology.

**METHODS**:

Observational descriptive study conducted through an anonymous on-line survey. An e-mail was sent to the local university students, who responded during headache. We addressed demography, consumption pattern and clinical characteristics. Study was approved by the institutional ethics board.

**RESULTS**:

1184 subjects fulfilled the survey, with a median age of 22. 57.7% were female. Headache started immediately after waking up the next day in 78.7% of the participants. Mean length was 6.7 hours and average intensity was 5,4 on a 0-10 scale (Fig. 1). Mean intensity was higher in patients with usual headache in comparison with subjects without headache (5.7 vs 5.1, p<0,001). We found correlation between the intensity of headache and the type of alcohol consumed, being higher in subjects who drank wine, followed by spirits and beer (p<0.001, p=0.001 and p=0.02, respectively). Topography was holocraneal in 84.6% with a frontal predominance in 42.9% (Fig. 2). Most frequent quality was oppressive (60.4%) and throbbing in 38.8%. 61.1% of participants complained about phonophobia, 43.1% of photophobia, 29.1% of osmophobia, 50.9% mentioned vegetative symptoms and 82,9% aggravation by physical activity. Presence of some trigeminoautonomal symptoms was described in 29.8% participants. 66.3% of responders described an orthostatic pattern worsening when becoming upright and 59.5% felt alleviation when lying down.

**Conclusions**:

In our sample we found that alcohol induced headaches howed an overlapping phenotype between a Migrainous headache and an intracranial hypotension pattern. This could be explained by both vasodilation and dehydration induced by alcohol.

**REFERENCES**:

1. Rasmussen BK, Olesen J. Symptomatic and non-symptomatic headaches in a general population. Neurology 1992; 42: 1225-31.

2. Penning R, van Nuland M, Fliervoet LA, Olivier B, Verster JC. The pathology of alcohol hangover. Curr Drug AbuseRev 2010 Jun; 3 (2): 68-75.

3. Dueland A. Headache and Alcohol. Headache 2015; 55 (7): 1045-1049.

**Fig. 1 (abstract P155).**
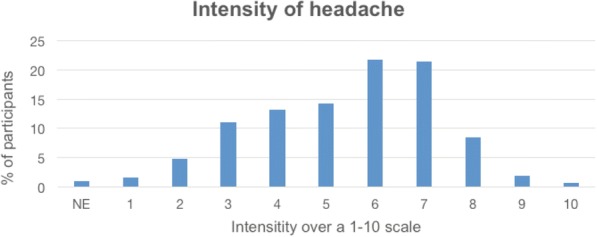
Intensity of headache presented in % of participants in a 1-10 scale

**Fig. 2 (abstract P155).**
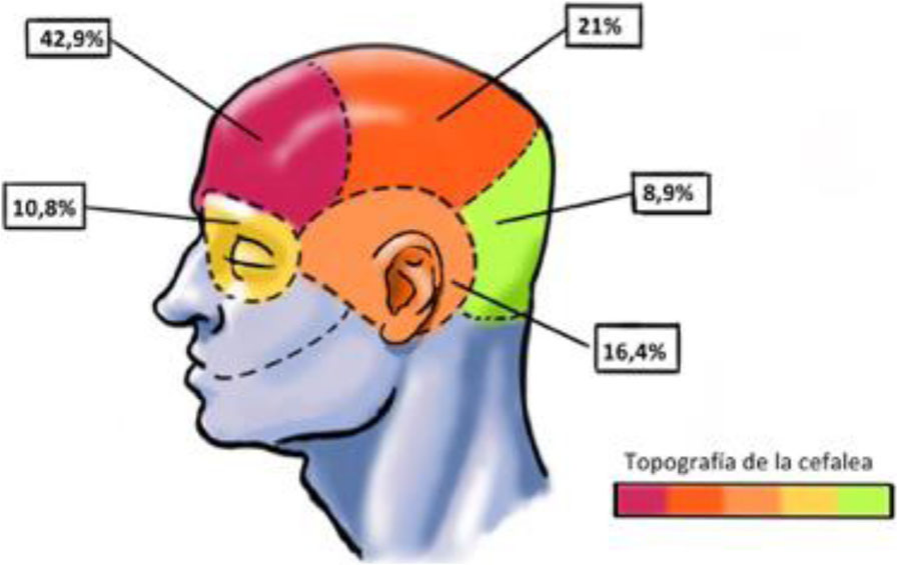
Topography of headache

### P156 Ibuprofen and not paracetamol as first line analgesic for acute migraine due to its interaction with TRPA1 in the cinnamaldehyde target engagement biomarker model

#### Dorien Bamps, Laura Macours, Linde Buntinx, Jan de Hoon

##### Center for Clinical Pharmacology, Department of Pharmaceutical and Pharmacological Sciences, KU Leuven, Belgium

###### **Correspondence:** Dorien Bamps (dorien.bamps@kuleuven.be)

**Background**: In case of an acute migraine headache, many patients rely on over-the-counter analgesics. Both ibuprofen and paracetamol are cornerstone therapies, without consensus on which one is the most effective drug. A recent overview of systematic reviews and meta-analyses directly compared ibuprofen and paracetamol at standard doses and concluded that ibuprofen was superior in relieving headache pain at two hours [1]. However, to date justification is lacking as the underlying mechanism of action remains elusive. This study aimed to assess the effect of ibuprofen and paracetamol on TRPA1, an ion channel implicated in the pathophysiology of migraine with migraine triggers such as acrolein and umbellulone as known TRPA1 agonists.

**Methods**: Sixteen healthy male volunteers were included to evaluate the effect of ibuprofen (600 mg) and paracetamol (1 g) on TRPA1 in a randomized, double blind, cross-over study. At Tmax of the administered drugs, a 10% cinnamaldehyde (CA) solution was applied topically to the volar surface of subjects’ forearms in order to induce vasodilation via TRPA1. At baseline and at 10, 20, 30, 40 and 60 minutes post application, the CA-induced dermal blood flow (DBF) response was measured using laser Doppler imaging (LDI). The area under the curve over a 60 minutes period (AUC_0-60min_) following administration of ibuprofen, paracetamol or no drug was analyzed using one-way ANOVA with post-hoc Bonferroni correction.

**Results**: As shown in figure 1, paracetamol had no significant effect on the CA-induced DBF response compared to no drug treatment, while after ibuprofen administration this DBF response was clearly reduced (p < 0.001 versus no treatment).

**Conclusion**: Using the TRPA1 target engagement biomarker model, we demonstrated that the non-selective COX-inhibitor ibuprofen reduces the DBF increase induced by CA. This possibly elucidates why ibuprofen was found superior in reducing headache pain compared to paracetamol. Furthermore, these results highlight TRPA1 as a potential target for the development of new migraine treatments.

**Ethics Approval**

Approval was obtained from the Ethics Committee of the University Hospitals Gasthuisberg, Leuven, Belgium (S57829) as well as the Federal Agency for Health and Medicinal Products (FAHMP) of Belgium (EudraCT 2014-004736-19).

**References**:

[1] Moore RA et al. Overview review: Comparative efficacy of oral ibuprofen and paracetamol (acetaminophen) across acute and chronic pain conditions. Eur. J. Pain 2015;19:1213–1223.

**Fig. 1 (abstract P156).**
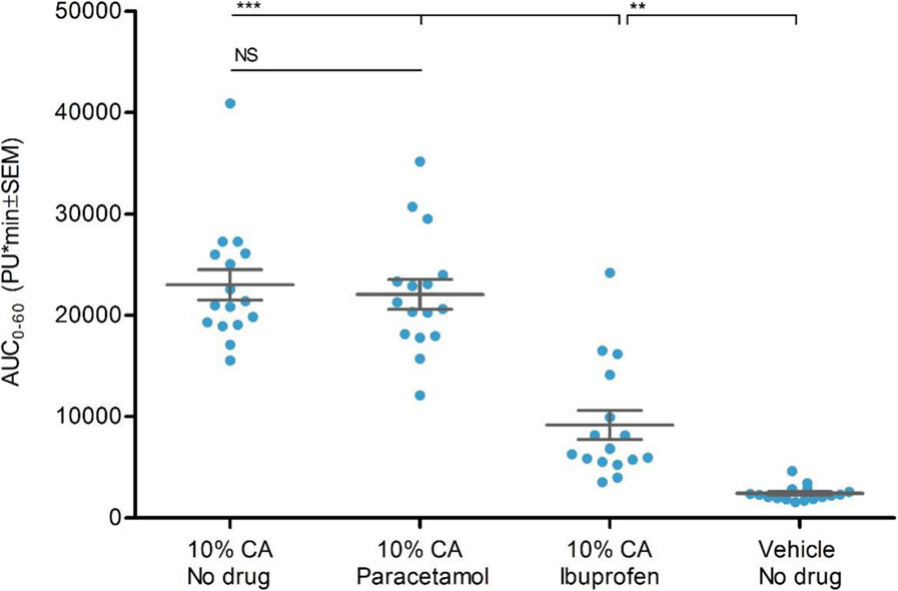
Influence of paracetamol and ibuprofen on the CA-induced DBF response (n = 16). One-way ANOVA with post-hoc Bonferroni NS: Non-significance (p > 0.05), ** : p < 0.01, *** : p < 0001

### P157 Efficacy and safety of external trigeminal neurostimulation with the Cefaly® device in chronic migraine: an open trial

#### Jean Schoenen^1^, Marius Birlea^2^

##### ^1^Headache Research Unit. CHR Citadelle. University of Liège. Belgium; ^2^University of Colorado Anschutz Medical Campus, Department of Neurology, Aurora, USA

###### **Correspondence:** Jean Schoenen (jschoenen@uliege.be)

***Background***:

External trigeminal neurostimulation (eTNS) with the Cefaly® device has proven efficacy for the prevention of episodic migraine [1]. In the PREMICE trial [1] patients with more than 6 migraine attacks per month benefited most from eTNS. Imaging studies suggest that eTNS may act by modulating activity in anterior cingulate cortex, an area also involved in chronic pain [2]. Given the poor efficiency of available treatments, we felt therefore that a trial of eTNS with the Cefaly® would be of interest in chronic migraine.

***Study protocol*** (ClinicalTrials.gov NCT02342743):

Seventy-three patients (18-65 years) fulfilling ICHD3 criteria for chronic migraine were included in a single centre, prospective, open trial and applied the Cefaly® device once or twice per day at home (20 min, 60Hz, 16mA) for 84 days after a 28-day baseline period. Headache parameters were monitored with paper diaries. The primary outcome measures were mean change from baseline in headache days and acute medication intake. The secondary outcome measures were mean change in migraine days and moderate/severe headache days, in monthly cumulative headache hours on headache days, average headache intensity, and 50% responder rate for migraine days.

***Results***:

Fifty-eight patients (24 with permanent headache) were included in the treatment phase and available for the ITT analysis (Fig. 1), 47 for the PP analysis.

In ITT, the two primary outcome measures showed a significant reduction after ENT: headache days decreased by 16.21% (p<0.001) and acute medication use by 8.11 units or 30.80% (p<0.001) (Fig. 1).

For PP, frequency of headache days was reduced from 22.66 to 19.02 days (-18.81%, p<0.001) and acute anti-migraine medication intake from 29.53 to 19.61, -9.92, p<0.001). The 50% responder rate was 18.97% overall, but 29.41% in patients with non-permanent headaches (n= 34). All secondary outcome measures also improved significantly after ENT, both in ITT and PP.

Patients with permanent headache (n=24) only had modest improvements although they used the device more often (153.78 sessions) than those with non-permanent headache (127.75).

In terms of safety, 78 AEs and 2 SAEs were reported, but only 2 AEs were related to the device: forehead skin irritation and burning sensation, which were minor and fully reversible.

***Conclusion***:

eTNS with Cefaly® appears to be an effective, safe and rather inexpensive prophylactic treatment for chronic migraine, indicating that a randomized sham-controlled or comparative trial is worthwhile. Its effect size is close to that reported for onabotulinum toxin A or topiramate. As for the latter, patients with permanent headache are poor responders and should be explored separately.

***References***

1. Schoenen J, Vandersmissen B, Jeangette S, Herroelen L, Vandenheede M, Gérard P, Magis D. Migraine prevention with a supraorbital transcutaneous stimulator: A randomized controlled trial. Neurology 2013, 80: 697-704

2. Schoenen J, Coppola G. Efficacy and mode of action of external trigeminal neurostimulation in migraine. Exp Rev Neurotherap 2018 (in press).

**Fig. 1 (abstract P157).**
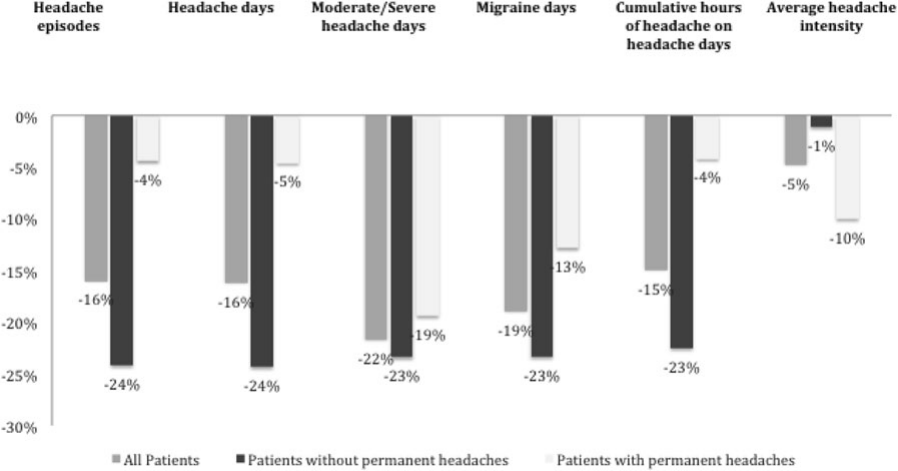
See text for description.

### P158 Abortive home treatment of migraine with external trigeminal neurostimulation using the Cefaly® device: a pilot trial

#### Jean Schoenen^1^, Joseph Mann^2^

##### ^1^Headache Research Unit. CHR Citadelle. University of Liège, Belgium; ^2^Rochester Clinical Research Center , NY, USA

###### **Correspondence:** Jean Schoenen (jschoenen@uliege.be)

***Background*** **:**

Safety and efficacy of external trigeminal nerve stimulation (eTNS) with the Cefaly® Acute device for the acute treatment of migraine have been demonstrated in previous in-hospital clinical trials that led to FDA approval in November 2017 (1,2). However, no data are available for patients using the device at home to treat a migraine attack. The main objective of this study was to obtain efficacy data for e-TNS in the abortive treatment of migraine using a protocol design similar to the one used in trials of triptans and other new abortive migraine medications.

***Study protocol*** *(*ClinicalTrials.gov identifier NCT03217968):

This was a single center, open-label trial conducted at the Rochester Clinical Research Center (NY, USA). Subjects aged (18 to 65 years) suffering from 2-8 attacks per month of migraine with or without aura (ICHD-3, codes 1.1 & 1.2.1) were recruited to treat with a 2-hour eTNS session a single migraine attack of moderate or severe intensity (grades 2 or 3 on the “Glaxo” scale) that started less than 4 hours before. Primary outcome measures were freedom of pain and of most bothersome migraine-associated symptom (MBS) at 2 hours. Secondary outcome measures were pain relief, absence of symptoms at 2 hours, sustained 2-24 hours pain freedom and use of rescue medication, Subjects were included in a modified ITT analysis (mITT) if they treated a qualifying migraine attack and filled in a headache diary at baseline and at 2 hours.

***Results***:

Fifty-nine subjects were included in the study and 48 subjects were eligible for the mITT analysis. After 2 hours of eTNS, 35.4% of subjects were pain-free and 60.4% were MBS-free, 70.8% had pain relief and 45.8% had no migraine-associated symptom. Half of subjects took rescue medication between 2 and 24 hours and 2-24h sustained pain freedom was achieved by 25% of subjects (Table 1). Regarding safety, 15 patients (25%) reported at least one AE, all minor and fully reversible, mainly forehead paraesthesia (13,6%).

***Conclusion*** **:**

This study shows that eTNS with the Cefaly® Acute is effective, well tolerated and safe for the abortive treatment of migraine attacks. Efficacy data are similar to those usually reported in triptan trials, but tolerability of e-TNS is better. A large multicenter, randomized, sham-controlled trial is needed to further confirm these data.

**References**:

1) Chou DE, Gross GJ, Casadei CH, Yugrakh MS. External trigeminal nerve stimulation for the acute treatment of migraine: open-label trial on safety and efficacy. Neuromodulation 2017; 20(7):678-683.

2) Chou DE, Yugrakh MS, Gross G, Winegarner D, Rowe V, Kuruvilla D. Acute treatment of migraine with e-TNS: a multi-center, double-blind, randomized, sham-controlled trial. Cephalalgia. 2017;37:323.

**Table 1 (abstract P158).** outcome after 2h of eTNS with the Cefaly® for the treatment of an attack in 45 migraine patients. Results are shown in % (numbers)

Primary outcomes	
Pain Freedom at 2 hours	**35.56%** (16)
MBS Freedom at 2 hours	**57.78%** (26)
Secondary outcomes	
Pain Relief at 2 hours	68.89% (31)
Absence of symptoms at 2 hours	44.44% (20)
Use of rescue medication	51.11% (23)
Sustained pain freedom at 24 hours	26.67% (12)

### P159 Paroxismal body pain without migraine; corpalgia

#### Levent Ertuğrul İnan^1^, F. Figen Ayhan^2^, F. Ayşen Eren^3^

##### ^1^Neurology and Algology Department, Ankara Training and Research Hospital of Ministery of Health, Ankara, Turkey; ^2^Physical Medicine Rehabilitation Department, Ankara Training and Research Hospital of Ministery of Health, Ankara, Turkey; ^3^Algology Department, Ankara Training and Research Hospital of Ministery of Health, Ankara, Turkey.

###### **Correspondence:** Levent Ertuğrul İnan; F. Ayşen Eren (aysendilbaz@gmail.com)

**Introduction:**

Migraine may have various symptoms such as atypical pain. Some pain states without headache have been associated with migraine like abdominal migraine and recurrent limb pain. Migrainous corpalgia is a migraine-related spontaneous body pain and may be associated with central sensitization. Here we present a case with paroxysmal recurrent pain attacks that start from the upper right and lower extremities and spread to the whole body, but not accompanied by headache.

**Case:**

A 41-year-old female patient was admitted to our outpatient clinic with complaints of pain and numbness in the whole body. The pain was present for about four years and has been spreading to the right upper limb and then the whole body starting from the right lower extremity for the last two years. The pain episodes were lasting for two days with a visual analog scale 8/10, and were repeating once or twice a month. According to the patients description the pain episodes had concomitant dizziness but headache, nausea or vomiting were absent. The patient did not describe morning stiffness or loss of strength.

For the medical treatment duloxetine 30 mg was given to the patient and the dose was increased up to 60 mg. The treatment was beneficial for the first six months, but the pain repeated despite the treatment. The patient participated in psychotherapy sessions 1-2 times a month for the last one year. Musculoskeletal system and neurological examinations were natural. There was no abnormality in routine blood tests. Brain MRI, EEG, EMG and SEP were within normal limits, cervical vertebral MRI was normal except minimal bulging at C3-4 level. The patient was started on 5 mg of Flunarizine. A month later, 50% reduction in the duration and severity of the pain was detected. The dose of flunarizine was increased to 10 mg and the patient was observed to have no pain at the end of 1 month follow-up.

**Conclusion:**

Corpalgia is defined as body pain. The condition is presented as migrainous corpalgia when it is accompanied by migraine. Except for migraine, there is no corpalgia case presence reported. The reported patient had migraine-like paroxysmal feature and progression, such as the spread of aura, but the complaints were not accompanied by headache. Another important point is the response of the patient to the given migraine prophylaxis agent; flunarizine. As a result, paroxysmal body pain which is treated by migraine prophylactic agent will open new horizons in pain physiopathology.

**Consent for publication:** Informed consent to publish has been obtained from this patient.

### P160 PREVALENCE AND CLINICAL PROFILE OF MIGRAINE WITH AURA IN A COHORT OF YOUNG PATIENTS WITH MINOR STROKE: A RETROSPECTIVE ANALYSIS.

#### Claudia Altamura, Angelo Cascio Rizzo, Matteo Paolucci, Nicoletta Brunelli, Gianluca Cecchi, and Fabrizio Vernieri

##### Headache and Neurosonology Unit, Clinical Neurology, Università Campus Bio-Medico di Roma, Rome, Italy

###### **Correspondence:** Claudia Altamura (c.altamura@unicampus.it)

**BACKGROUND** Patients with Migraine Aura (MA) display an increased risk for stroke at least in part explained by paradoxical embolism via Patent Foramen Ovale (PFO). Other pathogenetic mechanisms have been also involved to explain stroke occurrence in MA patients: increased prevalence of thrombophilia, cerebral hemodynamic impairment, and predisposition to cervical artery dissection. We aimed at exploring if stroke patients with MA presented peculiar clinical profiles.

**METHODS** We retrospectively enrolled 74 patients searching the clinical electronic dossiers of our Hospital for “stroke” diagnosis at the discharge of in patients younger than 55-year-old from November 2007 to March 2018. Reviewing the medical files, we collected demographic characteristics, vascular risk factors, comorbidity with MA, the results of PFO detection tests (via Transcranial Doppler and/or Echocardiography) and thrombophilic screening (factor II, factor V, MTHFR genotype, LAC, antiphospholipid antibodies, blood levels of homocysteine, C protein, S protein and ATIII). Stroke severity, etiology (according to ASCOD classification) and vascular territory were also defined.

**RESULTS** All patients (mean age at stroke onset was 46.6 ys (SD 8.2), 63.5% were male) had suffered for minor stroke (NIHSS<2). In 54% of cases, stroke etiology corresponded “other causes”. In 59.5 % of cases, stroke involved Middle Cerebral Artery territory. Prevalence of MA comorbidity in our cohort was 9.5%. These subjects all presented visual aura, with onset at 13.7 ys (SD 4.6) and a very wide frequency of MA attacks per year (min 1 max 180). Patients with MA were more frequently female (Chi-square p=0.04) and presented a higher prevalence of PFO (Chi-square p=0.001) and severe right-left shunts (Chi-square p=0.016) compared with the other stroke patients. No difference in stroke vascular territory and etiology, the prevalence of vascular risk factors (including estrogens), homocysteinemia and prothrombotic conditions was observed.

**CONCLUSIONS** Our study supports the hypothesis that PFO represents a relevant pathogenetic mechanism subtending the increase in stroke risk observed especially in female MA patients. In our patients, first MA attack occurred at a young age. Although the screening for PFO detection is not advisable in all MA patients, future studies are warranted to circumstantiate a subset of patients where it can have a clinical value.

### P161 The increase of subcutaneous abdominal and body fat is a characteristic associated to allodynia in migraineurs

#### Mínguez-Olaondo A.^1^, Romero S.^2,3^, Frühbeck G.^2,3^, Martínez-Vila E.^1^, Irimia P.^1^

##### ^1^Neurology, Clínica Universidad de Navarra, Pamplona; ^2^Endocrinology, Clínica Universidad de Navarra; ^3^CIBER Fisiopatología de la Obesidad y Nutrición (CIBERobn), ISCIII

###### **Correspondence:** Irimia P (pirimia@unav.es)

**BACKGROUND**

Cutaneous allodynia is a common feature that accompanies migraine attacks and is considered a clinical marker of central sensitization. Allodynia is one of the factors associated to migraine chronification, and is also associated with the presence of phono and photophobia. Obesity is a risk factor for chronification and excess body fat could release inflammatory mediators facilitating central sensitization.

**OBJECTIVES**

To compare the clinical and anthropometric characteristics that are associated with allodynia in patients with migraine and determine if the increase in body fat correlates with the presence of cutaneous allodynia.

**METHODS**

Prospective descriptive study of patients with chronic migraine (CM) and episodic migraine (EM) diagnosed in outpatient clinic. Cutaneous allodynia was studied with the Allodynia Symptoms Check-List 12 scale (ASC-12). Body composition was measured with ViScan TANITA® and Bod Pod Cosmed USA®. We also studied the relation of allodynia with the frequency and severity of migraine attacks, the presence of migrainous features (such as photophobia, and phonophobia), the lifestyle (sedentary or active), the body mass index (BMI) and duration of the disease. Chi2 was performed for percentages, T Student for means and Mann-Whitney U for nonparametrics values. Calculations are made using version 15.0.1 of SPSS® (SPSS, Chicago, USA). The study was approved by the Research Ethics Committee of Clínica Universidad de Navarra (approval no. 109/2015). Informed consent to publish has been obtained from patients.

**RESULTS**

We included 80 patients with migraine with chronic and EM according to International Classification of Headache Disorders criteria. Mean age was 39 years (18-65 years), of whom 65 (81.3%) were women. 65% of patients from our sample had allodynia. Patients with allodynia had higher truncal fat 36,06 vs 31,32 (p=0,023) and higher amount of body fat 33,03 vs 29,82 (p=0,028). The presence of allodynia was also associated with older age 41 vs 36 (p=0,03), sedentary lifestyle 76,2% vs 23,8% (p=0,027), migraine frequency 8,71 vs 4,78 days (p=0,03) and migraine severity 7,14 vs 5,93 (p=0,05), but not with the duration of the disease.

**CONCLUSIONS**

Cutaneous allodynia affects 65% of migraineurs in the population and is associated with an increase of the subcutaneous abdominal and body fat. Age, frequency and severity of migraine attacks are also associated with allodynia.

### P162 CLINICAL PROFILE OF MIGRAINE WITH AURA AND PATENT FORAMEN OVALE: A RETROSPECTIVE ANALYSIS.

#### Claudia Altamura, Matteo Paolucci, Nicoletta Brunelli, Gianluca Cecchi, Angelo Cascio Rizzo, and Fabrizio Vernieri

##### Headache and Neurosonology Unit, Clinical Neurology, Università Campus Biomedico di Roma

###### **Correspondence:** Claudia Altamura (c.altamura@unicampus.it)

**BACKGROUND** The physio-pathological relationship between patent foramen ovale (PFO) and Migraine with Aura (MA) is the matter of a long-standing debate. Nevertheless, MA patients display an increased risk for stroke at least in part explained by paradoxical embolism. The aim of our study was to explore if peculiar attack clinical features, baseline characteristics and family history relate to the presence of PFO in MA patients.

**METHODS** We retrospectively enrolled 102 patients searching the clinical electronic dossiers of our Headache Center for MA patients who had also undergone Transcranial Doppler (TCD) with microbubbles for the detection of PFO. We collected MA clinical features (number of attacks per year, aura duration, family history of MA, trigger factors, age onset, aura type) and the presence of vascular risk factors at MA onset. We also recorded echocardiography findings for the detection of inter-atrial septal aneurysm (ISA), and the results for thrombophilia screening. PFO test results were stratified for severity. The study received approval from the local Ethical Committee (Prot: 5.18 TS ComEt CBM).

**RESULTS** Our results showed that patients with PFO presented the first MA attack at younger age (Mann Whitney test, p<.0001) compared with patients without PFO. Besides, PFO severity was associated with progressively younger age onset (Spearman Rho -481, p<.0001). Similarly, patients with ISA presented younger age onset than patients without ISA (Mann Whitney test, p=.023). Also family history for MA was associated with PFO (Chi-squared p=.02). Aura duration and frequency was not associated with PFO. PFO was not associated with presence or kind of trigger factors neither with aura type. Age onset was not influenced by the presence of thrombophilia or other vascular factor at onset, not even in the PFO+ group.

**DISCUSSION** Our findings show that younger MA age onset and family history for MA are associated with the presence of PFO. One possible interpretation is that PFO lowers “MA threshold” or facilitates MA occurrence, anticipating the onset in predisposed subjects. This effect seems not to be mediated by thrombophilia as in patients with PFO the presence of thrombophilia was not associated to a younger MA onset. On the other hand, the wider we observed right-left shunt, the younger was the age onset. This finding suggests that PFO is not merely a bystander in MA physiopathology as the amount of micro-emboli or other substances bypassing the pulmonary filter through PFO makes a difference in the disease history.

### P163 The Association Between Low Carbohydrate Diet Score and Migraine

#### Soodeh Razeghi Jahromi^1,2^, Zeinab Ghorbani^3,2^, Mansoureh Togha^2^, Azita Hekmatdoost^1^, Faezeh Khorsha^2,3^, Boshra Torkan^2^, Nasim Rezaeimanesh^1,2^

##### ^1^Department of Clinical Nutrition and Dietetics, Faculty of Nutrition and Food Technology, Shahid Beheshti University of Medical Sciences, Tehran, Iran; ^2^Headache Department, Iranian Center of Neurological Research, Neuroscience Institute, Tehran University of Medical Sciences, Tehran, Iran; ^3^School of Nutritional Sciences and Dietetics, Tehran University of Medical Sciences, Tehran, Iran.

###### **Correspondence:** Mansoureh Togha (toghae@tums.ac.ir)

**Introduction**

The high fat, high protein and low carbohydrate diet (LCD) is widely used for seizure and epilepsy treatment. Due to the suggested underlying relationship between migraine and epilepsy, LCD might have some effects on migraine headache too. But to date, there are a few researches that have addressed the association between LCD and migraine. Thus, we aimed to investigate the relationship between total and animal or plant based LCD scores and migraine.

**Methods**

The case group were 534 migraineurs who were diagnosed based on ICHD III criteria. Control subjects consisted of 741 healthy individuals who were randomly selected from general population. After obtaining demographic and anthropometric data, a validated 168-item semi-quantitative food frequency questionnaire (FFQ) was applied for assessing dietary intakes. The total LCD score calculated according to dietary macronutrients intakes including carbohydrate, protein, fat and also animal fat, animal protein, vegetable plant and plant protein intake, expressed as a percentage of energy. The LCD scores increase with higher intakes of macronutrients, except for carbohydrate.

**Results**

Overall, about 62.1% of healthy subjects (mean age=43.66 years) and 92.5% of migraineurs(mean age=36.27 years) were women. Multiple regression models adjusted for age, gender, BMI and energy intake revealed a marginally significant 35% reduced odds of migraine for the subjects in the second quintile relative to those in the lowest quintile of total LCD score whereas the odds of migraine for those in the 4^th^ and 5^th^ quintiles increased significantly [(OR=0.65,95%CI=0.42-1.00 for the 2^nd^quintile); (OR=0.95,95%CI=0.63-1.44 for the 3^rd^quintile); (OR=2.04,95%CI=1.34-3.12 for the 4^th^quintile);(OR=3.32,95%CI=2.15-5.13 for the 5^th^quintile); compared to the lowest quintile as reference; P-for-trend=0.00].

Regarding animal based LCD score, the mentioned regression models resulted in roughly 2-7 fold higher odds of developing migraine for subjects in the fourth and fifth quintile of this score than those in the first quintile [(OR=2.67,95%CI=1.75-4.08 for the 4^th^quintile);(OR=7.19,95%CI=4.61-11.21 for the 5^th^quintile); P-for-trend=0.00]. In contrast, it was highlighted that the participants who had higher scores of plant based LCD had lower risk for migraine [(OR=0.44,95%CI=0.30-0.65 for the 2^nd^quintile);(OR=0.59,95%CI=0.40-0.86 for the 3^rd^quintile); (OR=0.83,95%CI=0.54-1.28 for the 4^th^quintile);(OR=0.67, 95%CI=0.45-1.01 for the 5^th^quintile); compared to the lowest quintile as reference; P-for-trend=0.001]

**Conclusion**

These findings highlight the protective role of a low carbohydrate diet based on plant sources of fat and protein on migraine headache. Conversely, it was shown that consuming a low carbohydrate diet, but rich in animal fat and animal protein could highly increase the risk of migraine in susceptible subjects.

### P164 The expanding burden of Idiopathic Intracranial Hypertension

#### Susan P Mollan^1,2^, Magda Aguiar^3^, Felicity Evison^4^, Emma Frew^5^, Alexandra J Sinclair ^1,2,6^

##### ^1^Birmingham Neuro-Ophthalmology Unit, University Hospitals Birmingham NHS Foundation Trust, B15 2TH, United Kingdom (UK); ^2^Metabolic Neurology, Institute of Metabolism and Systems Research, University of Birmingham, Edgbaston, B15 2TT, UK; ^3^Health Economics Unit, Institute of Applied Health Research, University of Birmingham, Birmingham, B12 2TT, UK; ^4^Department of Informatics, University Hospitals Birmingham NHS Trust, Queen Elizabeth Hospital Birmingham, Mindelsohn Way, Edgbaston, Birmingham, B15 2WB, UK; ^5^Centre for Endocrinology, Diabetes and Metabolism, Birmingham Health Partners, B15 2TH, UK; ^6^Department of Neurology, University Hospitals Birmingham NHS Foundation Trust, B15 2TH, UK.

###### **Correspondence:** Susan P Mollan (susan.mollan@uhb.nhs.uk)

**Objective**

To quantify the hospital burden and health economic impact of Idiopathic Intracranial Hypertension.

**Methods**

Hospital Episode Statistics (HES) national data was extracted between 1^st^ January 2002 and 31^st^ December 2016. All those within England with a diagnosis of Idiopathic Intracranial Hypertension were included. Those with secondary causes of raised intracranial pressure such as tumours, hydrocephalus and cerebral venous sinus thrombosis were excluded.

**Results**

23,182 new IIH cases were diagnosed. 52% resided in the most socially deprived areas (quintiles 1 and 2). Incidence rose between 2002 to 2016 from 2.3 to 4.7 per 100,000 in the general population. Peak incidence occurred in females aged 25 (15.2 per 100,000). 91.6% were treated medically, 7.6% had a cerebrospinal fluid diversion procedure, 0.7% underwent bariatric surgery and 0.1% had optic nerve sheath fenestration. Elective caesarean sections rates were significantly higher in IIH (16%) compared to the general population (9%), p<0.005. Admission rates rose by 442% between 2002 and 2014, with 38% having repeated admissions in the year following diagnosis. Duration of hospital admission was 2.7 days (8.8 days for those having CSF diversion procedures). Costs rose from £9.2 to £50 million per annum over the study period with costs forecasts of £462 million per annum by 2030.

**Conclusion**

IIH incidence is rising (by greater than 100% over the study), highest in areas of social deprivation and mirroring obesity trends. Re-admissions rates are high and growing yearly. The escalating population and financial burden of IIH has wide reaching implications for the health care system.

### P165 The Number of Active and Latent Trigger Points is Associated with Restricted Cervical Range of motion in Tension Type Headache

#### María Palacios-Ceña^1,2^, Stella Fuensalida-Novo^1^, Matteo Castaldo^1^, Kelun Wang^1^, Lars Arendt-Nielsen^1^, César Fernández-de-las-Peñas^1,2^

##### ^1^SMI, School of Medicine, Aalborg University, Aalborg, DENMARK; ^2^Departamento de Fisioterapia, Terapia Ocupacional, Rehabilitación y Medicina Física. Universidad Rey Juan Carlos, Alcorcón, Madrid, SPAIN

###### **Correspondence:** María Palacios-Ceña; César Fernández-de-las-Peñas (cesar.fernandez@urjc.es)

**Background:** Tension type headache (TTH) is a headache where the presence of trigger points (TrPs) can play a relevant role. There is evidence supporting that referred pain elicited by active TrPs reproduces the pain features in patients with TTH. Additionally, these patients also exhibit several musculoskeletal disorders in the cervical spine, e.g., restricted cervical range of motion. The association between active TrPs and restricted cervical range of motion has not been previously investigated.

**Objective:** The aim of the current study was to investigate the association between the number of active and latent TrPs with cervical range of motion in individuals with TTH.

**Methods:** Patients with TTH diagnosed by experienced neurologists according to the International Headache Classification (ICHD-III) were included. All participants read and signed a consent form prior to their participation Exclusion criteria included other primary headaches, medication overuse headache, previous whiplash or fibromyalgia. TrPs (active and latent) were bilaterally explored in the masseter, temporalis, trapezius, splenius capitis, sternocleidomastoid and suboccipital muscles by experienced physical therapists. Pain-free cervical range of motion was assessed in flexion, extension, lateral-flexion and rotation with a Cervical Range of motion (CROM) device. Spearman correlation coefficients (r_s_) were conducted to further determine correlations between the TrPs and cervical range of motion. The study design was approved by local Ethics Committee (URJC 23/2014, HUFA 14/104, Aalborg N20140063, CESU 5/2015).

**Results:** One hundred and seventy patients (mean age: 45±15 years) with TTH and a headache frequency of 17±9 days per month participated. Each individual with TTH exhibited 4.7±3.0 active TrPs in the cranio-cervical muscles which referred pain evoked by their palpation reproduced their headache, as well as a mean of 1.5±2.3 latent TrPs. The number of active TrPs showed weak negative associations with cervical flexion (r_s_: -0.189; P=0.014), extension (r_s_: -0.221; P=0.004) and lateral-flexion (r_s_: -0.161; P=0.04) but not with the rotation. Further, the number of latent TrPs showed moderate negative associations with cervical flexion (r_s_: -0.438; P<0.001), extension (r_s_: -0.417; P<0.001), lateral-flexion (r_s_: -0.420; P<0.001) but weak association with rotation (r_s_: -0.292; P<0.001): the higher the number of active or latent TrPs in the cervical musculature, the lower cervical range of motion.

**Conclusions:** The current study supports that the number of active and latent TrPs was associated with a restricted range of motion in individuals with TTH. In fact, latent TrPs were more relevant for cervical range of motion, supporting the potential role of latent TrPs in musculoskeletal disorders such as restricted range of motion in TTH sufferers. Future trials should explore the relevance of proper treatment of TrPs in the associations observed in the current study.

### P166 Evaluation of the 6-Item Identify Chronic Migraine (ID-CM) Screener in a Large Medical Group

#### Jelena M. Pavlovic^1^, Justin S. Yu^2^, Stephen D. Silberstein^3^, Michael L. Reed^4^, Steve H. Kawahara^5^, Robert P. Cowan^6^, Firas Dabbous^7^, Karen L. Campbell^2^, Riya Pulicharam^5^, Hema N. Viswanathan^2^, Richard B. Lipton^8^

##### ^1^Montefiore Medical Center, Bronx, NY, USA; ^2^Allergan plc, Irvine, CA, USA; ^3^Jefferson Headache Center, Philadelphia, PA, USA; ^4^Vedanta Research, Chapel Hill, NC, USA; ^5^DaVita Medical Group, El Segundo, CA, USA; ^6^Stanford University School of Medicine, Stanford, CA, USA; ^7^Independent Consultant, La Jolla, CA, USA; ^8^Albert Einstein College of Medicine, Bronx, NY, USA

**Objective**

The objective of this analysis was to assess the sensitivity and specificity of the 6-item Identify Chronic Migraine (ID-CM) screener.

**Methods**

Patients included in this analysis were enrolled in a large medical group and had at least 1 medical claim with an ICD-9/10 code for migraine in the 12-month pre-screening period. The 12-item ID-CM screener was then administered by e-mail, in person, or over the telephone to all patients enrolled into the study. The 12-item ID-CM screener is based primarily on 30-day patient recall and consists of questions that assess headache (HA) frequency, HA symptoms, medication use for HA, interference with activities due to HA, and planning disruption due to HA. The 6-item ID-CM screener is based on a subset of the 12- item version and consists only of the questions that assess HA frequency and HA symptoms.

Additionally, a Semi-Structured Diagnostic Interview (SSDI) was administered by telephone to a subset of eligible patients by a physician trained to reliably administer the tool. The SSDI assesses HA symptoms, frequency, disability, and medication use based on 30-day and 90-day patient recall and served as the study gold standard for determining CM status. Migraine patients that were not administered the SSDI were excluded from this analysis. Additionally, migraine patients that had a medical claim with an ICD-9/10 code for CM in the 12-month pre-screening period or a migraine-related onabotulinumtoxinA claim in the 12-month pre-enrollment period were excluded.

**Results**

The analysis of the 6-item ID-CM screener included 109 patients with a migraine diagnosis who completed the ID-CM screener and the SSDI. The average (standard deviation) age of the patient sample was 48.7 (14.5) years and 91.7% of them were female. Using the SSDI as the diagnostic gold standard for CM, the 6-item ID-CM screener had a sensitivity of 70.8% (46/65) and a specificity of 93.2% (41/44).

**Conclusions**

Optimal treatment of CM requires an accurate diagnosis of the disease. Based on the SSDI as the gold standard for CM diagnosis, the 6-item ID-CM screener demonstrated acceptable sensitivity and good specificity in determining CM status. The results support the real-world validity of the 6-item ID-CM screener and its use as a simple yet accurate tool to identify CM patients.

**Disclosures**:

JMP received honoraria from Allergan and the American Headache Society. JSY is an employee of Allergan plc and receives stock or stock options. SDS is a consultant and/or advisory panel member, Dr. Stephen Silberstein receives honoraria from Alder Biopharmaceuticals; Allergan, Inc.; Amgen; Avanir Pharmaceuticals, Inc.; eNeura; ElectroCore Medical, LLC; Labrys Biologics; Medscape, LLC; Medtronic, Inc.; Neuralieve; NINDS; Pfizer, Inc.; and Teva Pharmaceuticals. His employer receives research support from Allergan, Inc.; Amgen; Cumberland Pharmaceuticals, Inc.; ElectroCore Medical, Inc.; Labrys Biologics; Eli Lilly and Company; Merz Pharmaceuticals; and Troy Healthcare. SDS has no disclosures. MLR Vedanta has received research funding from Allergan, Amgen, CoLucid, Dr. Reddy’s Laboratories, Endo Pharmaceuticals, GlaxoSmithKline, Merck & Co., Inc., NuPathe, Novartis, and Ortho-McNeil, via grants to the National Headache Foundation. Vedanta Research has received funding directly from Allergan for work on the CaMEO Study.

### P167 HEADACHE IN PHARMACY : OBSERVATIONAL STUDY OF PHARMACOLOGICAL HABIT OF CEPHALALGIC PATIENT

#### Giorgio Dalla Volta^1^, Giorgio Zanchin^2^, Maria Pia Prudenzano^3^, Lidia Savi^4^

##### ^1^Headache Center of Neurological Unit of Istituto Clinico Citta’ di Brescia, Brescia; ^2^Headache Centre, Department of Neurosciences, Padua University, Padua; ^3^Headache Centre. Department of Basic Medical Sciences, Neuroscience and Sense Organs. University of Bari; ^4^Headache Center of Lugano

###### **Correspondence:** Giorgio Dalla Volta (dalla@numerica.it)

Noting that the pharmacy is the place of first access of the cephalalgic patient (as emerged from more studies done in the territory) even more than the general practitioner, that is consulted only once the patient realize that headache is a disease , with the aim of informing the patient and making the pharmacist to feel responsible to spread the concept of headache = disease in the clients / patients who address him , we have organized an observational pilot study under the aegis of the SISC in collaboration with the Pharmacists' Orders in some Italian provinces.

The involvement of the pharmacist in these studies can help to spread the knowledge of the risk inherent in the abuse of fans / triptans with the purpose that the patient talks with his doctor or consult a Headache Center.

The study provided for the delivery in 200 pharmacies distributed in the province of Brescia, Padua, Bari and Turin of 10,000 cards that the pharmacist had to fill out by asking for the information necessary ,to the first 50 customers who went to the pharmacy requesting an analgesic / tripthane / nutraceutical ,signaling headache as the reason of the purchase.

The data contained in the data sheet referred to the data of population (age and sex), how many patients were followed by the attending physician or a specialist or in a headache center or how many were self-curing, if they were following a preventive or only symptomatic therapy, if they were using a nutraceutical and in the case as prevention or only as symptomatic. We know that 45% of patients suffering from headaches use nutraceuticals of their choice without communicating the fact to their doctor or the specialist, and this fact must emerge to help the pharmacist handle these requests and the specialist to integrate these therapies with conventional drugs.

The study also allowed us to provide a concrete idea on how many patients go to the pharmacy for the symptom headache, a result very different from the epidemiological data reported in the population studies.

### P168 The Italian chROnic migraiNe (IRON) Registry: first report from 28 headache centers

#### Piero Barbanti^1^, Luisa Fofi, Gabriella Egeo, Cinzia Aurilia, Sabina Cevoli^2^, Giulia Pierangeli^2^, Paola Torelli^3^, Giancamillo Manzoni^3^, Cecilia Camarda^4^, Licia Grazzi^5^, Domenico D’Amico^5^, Fabrizio Vernieri^6^, Patrizia Balsamo^7^, Fabio Frediani^8^, Giacomo D'Arrigo^8^, Paola Di Fiore^8^, Cinzia Finocchi^9^, Francesco Perini^10^, Florindo d'Onofrio^11^, Francesco Bono^12^, Antonio Russo^13^, Roberto De Simone^14^, Guido Ferra^14^, Vincenzo Busillo^15^, Antonio Carnevale^16^, Francesco Pierelli^17^, Cherubino Di Lorenzo^17^, Fabio Valguarnera^18^, Renata Rao^19^, Salvatore Caratozzolo^19^, Stefano Messina^20^, Bruno Colombo^21^, Alfonso Coppola^22^, Gabriella Turano^23^, Giovanni Battista Allais^24^, Chiara Lia^25^, Ottavio Di Marco^26^, Marcella Curone^27^, Gennaro Bussone^27^, Vincenzo Tullo^27^, Maria Gabriella Saracco^28^, Nicola Vanacore^29^

##### ^1^IRCCS S.Raffaele, Unità per la cura e la ricerca su cefalee e dolore, Roma; ^2^IRCCS Istituto delle Scienze Neurologiche Bologna; ^3^Dipartimento di Neuroscienze, Centro Cefalee, Parma; ^4^UO di Neuologia e Patologie Cognitive. AOU Policlinico "P. Giaccone", Palermo; ^5^Fondazione IRCCS Ist. Nazionale Neurologico "Carlo Besta", Milano; ^6^Università Campus Bio-Medico, Roma; ^7^Poliambulatorio ASL5 Ghilarza; ^8^ASST Santi Paolo Carlo, UOC Neurologia e Stroke Unit Centro Cefalee, Milano; ^9^Dip. Scienze Neurologiche Oftalmologia e genetica, Cl. Neurologica II, Genova; ^10^S. Bortolo Vicenza – Neurologia; ^11^A.O.Rilievo Nazionale "S.G.Moscati", Avellino; ^12^Policlinico Mater Domini, Clinica Neurologica, Catanzaro; ^13^SUN - II Divisione di Neurologia, Napoli; ^14^Università Federico II, Dip. Scienze Neuologiche, Napoli; ^15^Ospedale Maria SS Addolorata - Div. Neurologica, Salerno; ^16^UOC Neurologia, Ospedale San Filippo Neri -ASL Roma 1; ^17^Centro per la cura dell’emicrania e delle cefalee, ICOT, Latina; ^18^Dip. Neurologia, C.Cefalee ASL 3 genovese, Ospedale Sestri Ponente "Padre Antero Micone", Genova; ^19^Ospedali Civili, U.O. I Neurologia, Brescia; ^20^IRCCS Ist. Auxologico Italiano - Università degli Studi di Milano, UO Neurologia, Milano; ^21^IRCCS Ospedale San Raffaele, Milano; ^22^A.O. "G.Salvini" - U.O. Neurologia, Garbagnate Milanese; ^23^S.C. Neurologia, Ospedale Regina Montis Regalis, Cuneo; ^24^Centro Cefalee della Donna - Dip. di discipline Ginecologiche e Ostetriche, Ospedale Sant’Anna, Torino; ^25^Ospedale Regionale della Valle d'Aosta, S.C. di Neurologia, Centro Cefalee Regionale; ^26^Ospedale SS Trinità, Frosinone; ^27^Casa di Cura Igea, Milano; ^28^Azienda Sanitaria Locale di Asti, Ospedale Cardinal Massaia, Soc. Neurologia; ^29^Centro Nazionale di Epidemiologia, Sorveglianza e Promozione della Salute, Istituto Superiore di Sanità, Roma

###### **Correspondence:** Piero Barbanti (piero.barbanti@sanraffaele.it)

**Background**

A national chronic migraine (CM) registry may improve disease knowledge and management, favor tailored treatment, rationalize resource allocation and improve economic sustainability of modern innovative treatments.

**Methods**

Since 1^st^ March 2018, twenty-eight Italian headache centers are actively participating to the Italian CM registry project. All consecutive patients affected by CM are being screened by specifically trained neurologists with face-to-face interviews using a shared, dedicated web-based database, created and approved by all participants in a series of preliminary meetings supervised by the Italian National Institute of Health. The database comprises 6 domains including 150 items detailing 1) life-style, behavioral and socio-demographic factors; 2) migraine features before chronicization; 3) chronic migraine characteristics and comorbidities; 4) healthcare resource use and socio-economic benefits; 5) MIDAS; 6) HIT-6. The protocol was approved by the institutional review board at IRCCS San Raffaele Pisana.

**Results**

At the date of 31^st^ May 2018, twenty-one headache centers have enrolled 161 CM patients.

*CM patient characteristics:* F/M 122/39; mean age 46.4±14.2 yrs; mean monthly headache days 26.8±15; medication overuse 80.7%; MIDAS 81.6±78.4; HIT-6 67.9±5.9; mean age at chronification 34.8 ±14 yrs; positive CM family history 16.2%; unilateral pain 44.6%; allodynia 55.4%; cephalalgophobia 58.5%. Most common comorbidities were psychiatric (50%), endocrinological (15.4%) and gastrointestinal (13.8%) disorders and hypertension (11.5%). Poor therapeutic compliance was present in 24.6% patients and only 45.4% used a headache diary.

*Previous episodic migraine characteristics:* without aura 88.5%, with aura 6.15%, with and without aura 5.4%; mean monthly frequency <4 days: 48.6%, 4-9 days: 34.6%, 10-14 days: 16.9%; duration <24 h: 60%, 25-48 h: 18.5%, 49-72 h: 13.1%, >72 h; 8.5%; unilateral pain: 48.5%. Only 11.5% of CM patients had used prophylaxis when their migraine was episodic.

*Healthcare and resource use:* age at first headache center consultation: 36.04±14.03 yrs; headache centers consulted: none 10%, <2: 48%; 3-8: 42%. Previous hospitalization for headache: 43.1%. ED access during the previous year: 25.4%. Mean diagnostic procedures per patient: 2.95±7.7. Procedures were improper or inadequate in 48.5% of cases, mostly loaded on the national health system (80.8%), usually prescribed by specialists (45.4%) or GPs (24.6%). Number of different specialists consulted per patient: 7.31±15.7.

**Conclusions**

The newly established Italian CM registry is providing useful clinical, epidemiological and healthcare/resource use insights, shedding lights on numerous neglected clinical governance areas. The registry will be of help in moving from clinical empiricism to precision medicine.

### P169 Spontaneous intracranial hypotension in a patient with meningeal diverticula: a case report

#### Torrente A., Davì M., Pilati L., Portera E., Scardina S., Di Marco S., Gangitano M., Cosentino G., Fierro B., Brighina F.

##### Department of Experimental Biomedicine and Clinical Neurosciences (BioNeC), University of Palermo, Palermo, Italy

**Objective:** spontaneous intracranial hypotension (SIH) can sometime represent a puzzling clinical condition due to missing causal factors and poor response to treatment. Here we describe a SIH case where cerebrospinal fluid (CSF) leaks follow to the uncommon cause of meningeal diverticula^[1]^.

**Materials:** a 54-years-old-woman who works as a caregiver came to our attention for a symptomatology characterized by heavy headache localized on the vertex that started after long lasting upright position and that improved with rest. During last two months the pain has begun costant, associated with vertigo and non responsive to rest or symptomatic drugs.

**Methods:** the patient was admitted to our Unit and remained for 9 days. During such period she underwent medical therapy with intravenous hydratation, 5% glucose solution and 8 mg of desametasone per day. We performed a cerebral CT scan and cerebral and cervico-dorsal contrast-enhanced magnetic resonance imaging (MRI). Moreover, the patient underwent a Neuro-surgical consult.

**Results:** CT scan showed intracranial hypotension suggestive signs and the diagnosis was confirmed by cerebral MRI scan, showing pachimeningeal thickening and contrast enhancement and slight caudal sliding of the cerebellar tonsils and diencephalic structures, associated to smaller ventricles. Cervico-dorsal MRI scan revealed the causal factor, highlighting the presence of 5 meningeal diverticula turning outwards at the level of right D9- D10 and D11-D12 and left D8-D9, D9-D10 and D10-D11 intervertebral foramen (the biggest 12 mm). The patient response to medical therapy was good and led to symptoms regression, making not necessary other therapeutic approaches, such as epidural blood patch or surgical intervention. One month after discharge the patient referred no headache, but sporadic vertigo that she controlled using antivertiginous drugs.

**Discussion:** spontaneous intracranial hypotension has several causes, one of them is represented by cerebrospinal leaks. Meningeal diverticula represent a possible, even if very rare, cause of cerebrospinal leaks and, in our case, such link was probably at the origin of our patient symptoms and imaging signs. Medical treatment succeded in soothing patient’s headache and vertigo, so, in future, it will be useful to reconsider the need for surgical intervention in patients with meningeal diverticula.

**Conclusions:** Meningeal diverticula, even if rare, are possible causes of SIH and medical treatment could be effective in such cases, since surgery is not always needed.

**Consent for publication**: the patient signed a written informed consent for the publication of this case report.

[1] W.I. Schievink, M.M. Maya, C. Louy, F.G. Moser, J. Tourje. Diagnostic Criteria for Spontaneous Spinal CSF Leaks and Intracranial Hypotension. AJNR Am J Neuroradiol 29:853–56, May 2008.

### P170 Estimation of the Economic Burden and Labor Impact of Migraine in Spain: Results from the Spanish Atlas of Migraine Survey 2018

#### Pablo Irimia^1^, Marco Garrido-Cumbrera^2,3^, Sonia Santos-Lasaosa^4^, Isabel Colomina^5^, Francisco Javier León^6^, Carles Blanch^7^, Patricia Pozo-Rosich^8,9^

##### ^1^Clínica Universidad de Navarra, Pamplona; ^2^Health & Territory Research, Sevilla; ^3^Universidad de Sevilla, Sevilla; ^4^Hospital Clínico de Zaragoza; ^5^AEPAC, Madrid; ^6^UGC Zaidín-Sur, Granada; ^7^Novartis, Barcelona; ^8^Headache Unit, Neurology Department, Hospital Vall d’Hebron, Barcelona; ^9^Headache Research Group. Vall d’Hebron Institut de Recerca (VHIR), Universitat Autonoma of Barcelona

###### **Correspondence:** Pablo Irimia (pirimia@unav.es)

**Objectives:** To estimate the average annual cost per patient and the impact on work t of migraine in Spain.

**Material and Method:** This is a prospective, online, anonymous, cross-sectional survey, conducted between June and September 2017, promoted by the Spanish Association of Patients with Headache (AEPAC) within the framework of the Spanish Atlas of Migraine 2018. People who completed the survey answered questions in relation to their migraine. A distinction was made between chronic migraine (CM) and episodic migraine (EM), considering the monthly headache days declared by patients. The economic burden of migraine was evaluated: direct health costs (including visits to specialists, medical tests, emergency visits, hospital admissions and medication), indirect costs (lost labor productivity), and those assumed by the migraineur. The labor consequences of migraine over the last year were analyzed. Chi-square and Mann- Whitney tests were used as contrast tests. Ethics Approval: A central ethics review board approved the study design.

**Results:** 1,281 people with migraine participated in the survey, 34.2% with CM, 88.2% women, with an average age of 37.3 (SD 11.5). The direct health costs for the last year were estimated at €3,847.29 for CM and €964.19 for EM (p<0,001). The costs assumed by the patient in the last year were €1,609.89 for CM and €878.04 for EM (p<0.001). The indirect cost was estimated at €7,464.83 for CM and €3,199.15 for EM (p <0.001). The total average cost per patient/year rised to €12,922.01 for CM and €5,041.41 for EM (p<0.001). Regarding the job status: 62.2% with EM and 49.0% with CM were working, 2.6% with EM and 9.1% with CM were on sick leave and 12.2% with EM and 16.8% with CM were unemployed (p <0.05). In the last year, because of migraine, 17.8% of patients with EM and 27.2% with CM (p<0.01) requested days of leave or leave of absence, and reduced their working hours 8.5% with EM and 11.1% CM (p=0.270). Labor efficiency was reduced in 61.1% of patients with EM and 65.7% with CM (p=0.257).

**Conclusion:** Migraine represents an important economic burden in Spain, particularly in patients with CM. Migraine causes important productivity losses resulting from absenteeism, presentism, decreasing the working hours and the probabilities to keep working, and its impact is significantly greater in CM.

### P171 Chromig10: evolution of migraine after 10 years

#### E. Caronna^1^, E. Fonseca Hernández^1^, VJ. Gallardo^2^, JB. Gomez^2^, A. Alpuente^1,2^, M. Torres-Ferrús^1,2^, P. Pozo- Rosich^1,2^

##### ^1^Headache Unit, Neurology Department, Vall d’Hebron Hospital, Barcelona, 08035, Spain; ^2^Headache Research Group, Vall d’Hebron Research Institute, Barcelona, 08035, Spain

###### **Correspondence:** P. Pozo- Rosich (ppozo@vhebron.net)

**Objective**

To analyse the evolution of a cohort of patients with migraine after 10 years, focusing on prognostic factors of improvement and worsening.

**Methods**

Cross-sectional analysis of the cohort of migraine patients from the *Chromig* study after 10 years. Using an online survey we collected demographic data, comorbidities, characteristics, treatment, impact of migraine (HIT-6, MIDAS, BDI, SF-36) and subjective impression of evolution. Initial and after-10-years data was compared. A reduction ≥25% in headache days/month was considered as improvement. A comparative study was carried out to identify predictors of improvement or worsening.

**Results**

Data was collected from 380/1109 patients (34.3%): 77.1% women; mean age 49.2 ± 10.5 years; 73.9% initial diagnosis of episodic migraine (EM). After 10 years: patients have more arterial hypertension (6.1% *vs.* 13.2%), less anxiety (57.5% *vs.* 22.9%) and depression (36.1% *vs.* 13.9%) (p<0.05). A 20.8% of women have gone through menopause. There is 66.8% reported subjective improvement of their migraine. Mean frequency of headache improves (9.6±8.5 *vs.* 2.9±4.2 headache-days/month p<0.001), 80.7% decrease in frequency ≥25%, (from these ten years ago, 43.3% had low-frequency EM; 24.6% high-frequency EM; 23.9% chronic migraine; 8.2% chronic daily headache (p<0.001)), which correlates with a lower proportion of high- frequency EM (22.1% *vs.* 7.7%), chronic migraine (15.9% *vs.* 2.6%) and chronic daily headache (6.6% 0 *vs.* 0.3%) (p <0.001); as well as with an improvement in impact (HIT-6) and quality of life (SF-36) (p<0.05). The factors associated with improvement are: baseline frequency >10 days/month, change in routines and menopause onset (p<0.05). In the multivariate analysis, the factor independently associated with improvement is the baseline frequency >10 days/month (OR:1.268, p=0.005). The factor associated with no improvement after 10 years is going through mourning (p<0.05). As additional results of the analysis, we observed a reduction in the use of preventive treatment (48.7% *vs.* 23.5%) and an increase in monotherapy (42.2% *vs.* 72.7%) (p<0.001). 44.4% of patients don’t have a medical follow-up for their migraine.

**Conclusion**

After 10 years, patients with migraine improve, especially those who 10 years ago had a high-frequency of headache days/month. Other than the natural pathophysiology of migraine, the factors which correlate with this improvement are a change in life habits and the onset of menopause.

### P172 WHOLE GRAIN CEREAL CONSUMPTION REDUCES MIGRAINE RELATED DISABILITY

#### Claudia Altamura, Gianluca Cecchi, Giorgia Botti, Maria Bravo, Paola Di Caprio, Nicoletta Brunelli, Matteo Paolucci, Manon Khazrai and Fabrizio Vernieri

##### Headache and Neurosonology Unit, Neurology Unit, Università Campus Bio-Medico di Roma, Rome, Italy

###### **Correspondence:** Claudia Altamura (c.altamura@unicampus.it)

**Introduction**

Diet has been often implied in Migraine pathophysiology. [1] Recently the Agricultural Department of United States published a renewed version of food pyramid: the Healthy eating Plate.[2] This study aimed at evaluating the effect of the education on the Healthy eating Plate on migraine frequency and disability.

**Methods**

We enrolled 148 consecutive Migraine patients (mean age 41 ys SD 13.2, 86% female) referring to our Headache Centre. At baseline, all patients underwent anthropometric assessment and filled a Frequency Food Questionnaire (FFQ) to assess their dietary habit months and clinical scales (MIDAS e HIT-6) in the previous three. All patients received prophylactic treatment indications as appropriate. After two months (T2) patients underwent anthropometric assessment, filled a Frequency Food Questionnaire (FFQ) and disability scales. They were educated about the healthy eating plate advices. Patients requiring change in the preventive therapy were considered dropouts. Three months later (T5), the enrolled patients returned for the control visit and underwent again the assessments. The study was approved by our Local Ethical committee (prot 6.18TS ComET CBM)

**Results**

The analysis of baseline data showed average BMI 24.6 ±4 and Waist Circumference 83cm; mean headache days per month 9.3 ± 7, abortive drugs per month 9.2±7. We observed a correlation between sweet confectionery products intake and MIDAS-tot (rho di Spearman= 0.189, p= 0.021) and HIT6 (Spearman’s rho=0.231, p=0.005). The intake of rice was related with MIDAS-B (Spearman’s rho=0.223, p=0.01). Likewise the consumption of potatoes had was related with MIDAS- B (Spearman’s rho=0.216, p=0.022) and HIT6 (Spearman’s rho=0.200, p=0.034). At t5, we observed a correlation between increase in whole grain cereals intake and a decrease in disability scales (Spearman’s rho= -0.660 and -0.438; p= <0.0001 and p=0.029 for MIDAS tot and Hit-6 respectively). Variation in MIDAS tot was related with changes in refined cereals intake (Spearman’s rho=0.446, p=0.014). No relation was observed for weight change.

**Discussion and Conclusion**

This study showed for the first time that a healthy diet may be a real help for migraine prevention. In particular, the shift from refined to whole cereals seems to play the relevant role independently of weight loss. The pathogenetic mechanism explaining the beneficial effect of whole grain cereal on migraine is possibly based on a more steady glycemic control. Alternatively, we can speculate that a high fibre diet can modify gut microbiota and in turn have a detrimental effect on migraine.

**References**

1. Di Lorenzo C, Coppola G, Sirianni G, et al (2015) Migraine improvement during short lasting ketogenesis: a proof-of-concept study. Eur J Neurol 22:170–7 . doi: 10.1111/ene.12550

2. Il Piatto del Mangiar Sano (Italian). https://www.hsph.harvard.edu/nutritionsource/healthy-eating-plate/translations/italian/

3. Dalcara T. How does fasting trigger migraine? A hypothesis. Curr Pain Headache Rep. 2013;17(10):368)

### P173 The Association Between Dietary Tryptophan Intake and Migraine

#### Soodeh Razeghi Jahromi^1,2^, Mansoureh Togha^2^, Zeinab Ghorbani^2,3^, Hossein Ansari^4^, Azita Hekmatdoost^1^, Faezeh Khorsha^2,3^, Pedram Shirani^1^, Morvarid Nourmohammadi^2^

##### ^1^Department of Clinical Nutrition and Dietetics, Faculty of Nutrition and Food Technology, Shahid Beheshti University of Medical Sciences, Tehran, Iran; ^2^Headache Department, Iranian Center of Neurological Research, Neuroscience Institute, Tehran University of Medical Sciences, Tehran, Iran; ^3^School of Nutritional Sciences and Dietetics, Tehran University of Medical Sciences, Tehran, Iran; ^4^Department of Neurology, University of California San Diego (UCSD), USA

###### **Correspondence:** Hossein Ansari (headache@hansari.com)

**Background**

Migraineurs have been identified to have chronically decreased serotonin levels which markedly increases during ictal periods. Also, increased sensitivity to serotonin agonists might occur during attacks probably due to a defect in serotonin metabolism. Tryptophan is a precursor of serotonin. Once tryptophan concentrations diminished, it results in a short-term drop in neural serotonin level. However, much uncertainty still exists about the association between tryptophan and migraine. Regarding the importance of adequate tryptophan intake in regulating serotonin hemostasis and subsequent effects on migraine attacks, we designed the current study to assess the relationship between dietary tryptophan intake and migraine headache risk.

**Methods**

The migraine group (n=550, diagnosed according to the ICHDIII criteria) were recruited from a tertiary headache clinic. The control subjects consisted of 741 healthy volunteers who were randomly selected from general population. After collecting demographic and anthropometric data, a validated 168-item semi-quantitative food frequency questionnaire (FFQ) was used for dietary intake assessments. Multiple regression models were applied in order to explore the relationship between migraine and dietary tryptophan intake.

**Results**

The mean(SD) of the age of participants in the controls and migraine group was 43.83(14.50) and 36.21(9.85) years, respectively. Also, the mean(SD) BMI of controls and cases were about 27.71(4.57) and 25.99(4.79) kg/m^2^, respectively. The multiple regression models were adjusted for age, sex, BMI and dietary intakes of energy, food groups(g/d) such as total grains, vegetables, fruits, fish and poultry, red and processed meat and nuts groups and also intake of a number of nutrients including animal based protein(g/d), plant based protein(g/d), total fat(g/d), saturated fat(g/d), unsaturated fat(g/d), and cholesterol(mg/d). the models showed significant inverse association between tryptophan intake and migraine risk [(OR in the 3^rd^ quartile=0.31;95%CI= 0.13- 0.72) (OR in the 4^th^ quartile= 0.19;95%CI=0.05-0.66) with the first quartile as reference;p-for-trend=0.001].

**Conclusion**

To the best of our knowledge, this is the first relatively large population based investigation of the migraine headache risk according to dietary tryptophan intake. Our results showed that subjects who had a median intake of 0.90-1.15 grams of tryptophan per day had a reduced odds of developing migraine by approximately 69-81%, relative to those consumed ≤0.57 g/d. These findings therefore highlighted that considering enough intake of tryptophan rich foods such as milk, poultry, egg, seafood, soybeans, salmon within a healthy diet could lead to attenuating the odds of migraine among susceptible subjects.

### P174 Serum Vitamin B12 and Methyl-malonic Acid Status in a group of Migraine Patients Compared to Healthy Controls: A Case-Control Study

#### Mansoureh Togha^1^, Soodeh Razeghi Jahromi^2,1^, Zeinab Ghorbani^1,3^, Fahimeh Martami^1,2^, Maryam Seifishahpar^1,2^.

##### ^1^Headache Department, Iranian Center of Neurological Research, Neuroscience Institute, Tehran University of Medical Sciences, Tehran, Iran; ^2^Department of Clinical Nutrition and Dietetics, Faculty of Nutrition and Food Technology, Shahid Beheshti University of Medical Sciences, Tehran, Iran; ^3^School of Nutritional Sciences and Dietetics, Tehran University of Medical Sciences, Tehran, Iran

###### **Correspondence:** Mansoureh Togha (toghae@tums.ac.ir)

**Background**

Vitamin B12 is involved in scavenging against NO and prevention of hyperhomocysteinemia, the two factors which are proposed to be probably implicated in migraine pathogenesis. Thus, in the current study we aimed at evaluating the serum vitamin B12 and its most sensitive and specific biomarker, methyl-malonic acid (MMA) status in a group of migraine patients compared to healthy controls.

**Methods**

Seventy migraine patients (34 chronic and 36 episodic migraineurs) and 70 sex- age matched control subjects were enrolled in this case control study from April to September 2017. Patients were diagnosed based on an expert headache specialist-neurologist examination according to the International Headache Society criteria (ICHD-IIIβ). Migraine characteristics include the number of headache attacks, severity of headaches (from 0 to 10), and duration of each attack in hours were recorded based on a 30-day headache diary. The serum vitamin B12 and MMA levels were measured with ELISA and using commercially available test kits. The study protocol was approved by the ethics committee of the Tehran University of Medical Sciences (ethics board approval code= IR.TUMS.IKHC.REC.1396.2468).

**Results**

The serum levels of B12 were found to be significantly lower in migraine patients than in control subjects(584.08± 300.20 vs. 750± 350.91pg/ml;P=0.007); whereas migraineurs had higher levels of MMA than control participants(2.171± 1.90 vs. 2.07± 2.05μg/dL;P=0.02). In the fully adjusted regression models, those in the highest vs. the lowest serum B12 quartile had 80% decrease in the odds of having migraine(OR= 0.20, 95% CI= 0.05-0.73;P for trend= 0.008)]; while, patients in the highest quartile of MMA had more than 5 times increased risk of developing migraine(OR= 5.44, 95%CI= 1.49-19.87;P for trend= 0.002). There was no association between serum B12 levels and headache characteristics.

**Conclusion**

Taken together, these results suggest that increasing level of serum B12 was accompanied by roughly 80% decrease in the odds of developing migraine. In addition, it was shown that participants with higher MMA levels, that considered as lower functional activity of B12, had about 4-to-5 fold higher odds of having migraine.

### P175 PRELIMINARY EFFICACY STUDY IN PROPHYLAXES OF EPISODIC TENSION CEPHALA AND HEMICRANIA WITHOUT AURA USING A COMBINATION OF MAGNESIUM, L-TRIPTOFANO, BOSWELLIA SERRATA CASPEROME®, NIACINA, RIBOFLAVINA AND VITAMIN D COMPARED WITH AMITRIPTILINE

#### L. Balzano^1^, B. Ciccone^2^

##### ^1^Neurologist – Clinic for the diagnosis and treatment of headaches – Torre del Greco (NA) ASL NA3 SUD; ^2^Neurophysiopathologist - Clinic for the diagnosis and treatment of headaches ATHENA Saviano (NA)

###### **Correspondence:** L. Balzano (neurogino@yahoo.it)

**INTRODUCTION**

Open-label efficacy study in prophylaxis therapy using Magnesium 225mg, L-Tryptophan 150mg, Boswellia Serrata Casperome® 100mg, Niacin 16mg, Riboflavin 1.4mg, Vitamin D 10mcg (Normorelax® = NRX) to Amitriptyline (AM) in patients with CTE and ESA (1-2-3). Outcomes of th study are: pain modulation (NRS scale), monthly attacks number and monthly analgesic-triptans consumption.

**MATERIALS AND METHODS**

200 patients with CTE and ESA using ICHD-II were selected: 100 CTE and 100 ESA.

50 CTE assuming NRX (two tablets per day) compared to 50 assuming AM (20 mg evening). 50 ESA assuming NRX, compared to 50 assuming AM. Results were evaluated at T1 (60 days) and T2 (120 days).

The longitudinal variatons of the three outcomes were analyzed through the GEE (Generalized Estimating Equations) modeling in order to check the correlation induced by the repeated measures. In all the Group factor , the time induced by the repeated measures. In all the models the Group factor, the time factor (as a categorical variable) and their interaction were included as predictors.

**RESULTS**

Both groups show statistically significant changes from T0 to T2 for all the outcomes considered. In CTE patients of NRX and AM group results are, respectively : (Table 1 and Fig. 1)

NRS reduces by 2.4 (p <0.001) and by 3.5 (p <0.001) points, attacks number reduces from 9.5 to 5.7 (p <0.001) and from 9.6 at 4.7 (p <0.001); analgesics frequency is reduced by an average of 3.1 (p <0.001) and 4.9 (p <0.001). Patients percentage showing a reduction in attacks frequency ≥ 50% from baseline is 24% in NRX and 40% in AM group.

In ESA patients in NRX and AM group, results are, respectively: (Table 2 and Fig. 2)

NRS reduces by 3.3 (p <0.001) and by 3.7 (p <0.001) points; attacks number reduces from 9.7 to 5.2 (p <0.001) and from 9.3 to 4.2 (p <0.001); analgesics frequency is reduced by an average of 4.9 (p <0.001) and 7.2 (p <0.001); patients percentage showing a reduction in attacks frequency ≥ 50% from baseline is 40% in NRX and 60% in AM group.

**DISCUSSION AND CONCLUSIONS**

Results confirm the improvement of all the outcomes in patients treated with NRX.

The greater treatment efficacy with AM compared to NRX is confirmed; there is no statistically significant difference in patients with ESA vs CTE in monthly attacks reduction, with NRX advantage for no side effects and greater patient compliance.

**References**

1. Ciccone B, D'Otolo G and Balzano L, Efficay of Oral Supplement Compared with Amitriptyline in the Prophylaxis of Episodic Tension Type Headache and Migraine without Aura Current Neurology and Neuroscience An open access journal, Vol 1 (1): 1-2, Feb 2018

2. LEONE M. et al., A review of the treatment of the primary headaches, in the Italian Journal of Neurological Science, 16 (1995), 577-586.

3. SILBERSTEIN S.D., Practice parameter: Evidence-based guidelines for migraine headache (an evidence-based review): Report of the Quality Standards Subcommittee of the American Academy of Neurology, in Neurology, 55, 2000.

**Table 1 (abstract P175).**
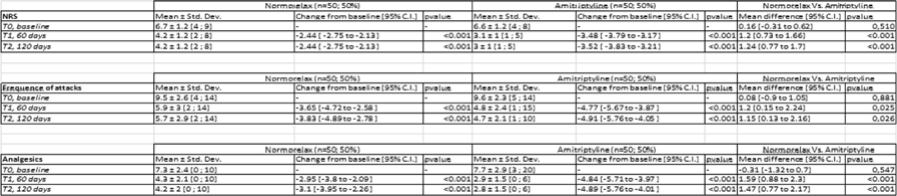
CTE

**Table 2 (abstract P175).**
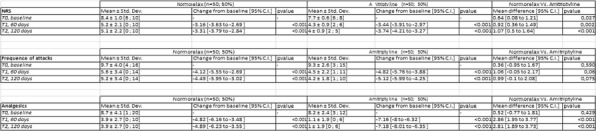
ESA

**Fig. 1 (abstract P175).**
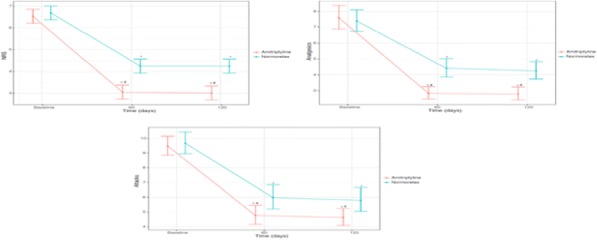
CTE

**Fig. 2 (abstract P175).**
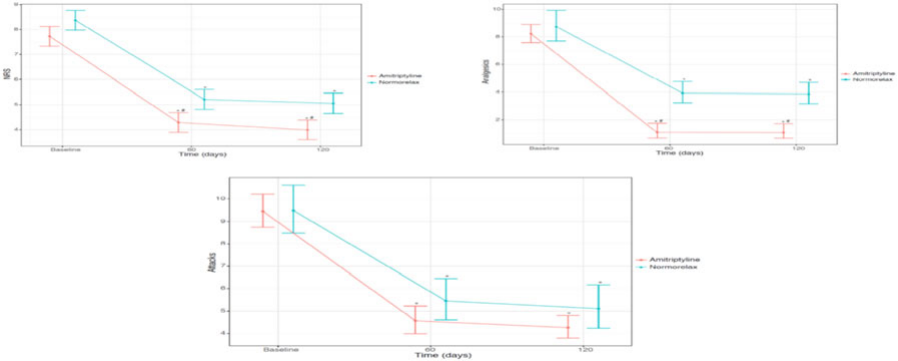
ESA

## SISC Poster Presentation

### P176 Efficacy in high frequency migraine with aura prevention of a combination of Tanacethum Parthenium, 5 - hydroxy tryptophan and magnesium (Aurastop^©^)

#### Giorgio Dalla Volta, Paola Zavarise, Gaelle Ngonga, Danilo Fontana

##### Headache Center of Neurological Unit of Istituto Clinico Citta' di Brescia, Brescia

###### **Correspondence:** Giorgio Dalla Volta (dalla@numerica.it)

**Background**: Each component of the novel phytotherapic combination of Tanacethum Parthenium (150 mg), 5-hydroxy tryptophan (20 mg) and magnesium (185 mg) (Aurastop^©^) acts on a different target among the main mechanisms involved in the pathophysiology of migraine and of the aura itself : sensitization of trigeminal vascular system, central sensitization and activation of the "migraine generator" located in the brainstem, through glutammate and kynurenine pathways and the cortical spreading depression [1,2,3]. Aim of this study is to test the effectiveness of Aurastop^©^ in the prophylaxis of migraine with aura with high frequency

**Materials and methods**: 18 patients (F: n=10, M: n=8, mean age: 28 ) presenting with an ICHD-3 beta diagnosis of migraine with aura (MWA ) with a frequency of more than 5 attacks of migraine with aura per month since at least 6 months, were enrolled in the survey and treated with Aurastop^©^ twice a day for a period of 3 months. Diary cards were filled in during a 3-months period prior the beginning of the survey and during the 3-months duration of the study. The reduction of MWA attacks per month was assessed as the primary end-point; the reduction of the duration and disability of the aura and of the intensity of the headache were considered as secondary end-points.

**Results**: A statistically significant reduction of MWA attacks /month was observed: more than 95% of the patients referred a reduction >50% of the frquency. Moreover, a sensible reduction of the duration and disability of the aura phenomena was reported by more than 90% of the patients and, in the 60% of the patients also a reduction of the intensity of the headache. No side effects were reported. The efficacy started to appear during the first month of intake and was maintained during the three months of therapy .

**Conclusion**: In this observational open study, Aurastop^©^ appears to be effective and safe as a preventive treatment of MWA in the patients with a high frequency of attacks.

**References**

1. Curto M, Lionetto L, Negro A, Capi M, Fazio F, Giamberardino MA, Simmaco M, Nicoletti F, Martelletti P. Altered kynurenine pathway metabolites in serum of chronic migraine patients. J Headache Pain. 2015; 17: 47.

2. Geppetti P, Bernabei S, De Cesaris F. CGRP receptors and TRP channels in migraine. J Headache Pain. 2015; 16(Suppl 1): A21.

3. Diener HC, Pfaffenrath V, Schnitker J, Friede M , Henneicke-von Zeppelin HH. Efficacy and safety of 6,25 mg t.i.d feverfew CO2-extract ( MIG-99) in migraine prevention – a randomized , double blind, multicenter, placebo controlled study. Cephalalgia. 2005; 25: 1031-41.

### P177 Quantitative analysis of perfusion Computed Tomography images increases the evidence of hypoperfusion during migrainous aura

#### Laura D’Acunto^1^, Antonio Granato^1^, Miloš Ajčević^1^, Giovanni Furlanis^1^, Maja Ukmar^2^, Irene Zorzenon^2^, Mariana Ridolfi^1^, Paolo Manganotti^1^

##### ^1^Department of Medical, Technological and Translational Sciences, Headache Centre, University of Trieste, Italy; ^2^Department of Radiology, Cattinara Hospital, University of Trieste, Italy

###### **Correspondence:** Laura D’Acunto (laura.d.acun@gmail.com)

**Background:** Perfusion computed tomography (PCT) represents a rapid and practical technique for assessment of salvageable tissue and infarct core in acute stroke imaging [1,2]. Perfusion patterns found during migraine with aura are controversial, in fact normal, hypo- and hyperperfusion were reported, though perfusion measurements were not always performed in the acute phase of the aura [3-6]. Aim of the present study is to demonstrate that an ad hoc quantitative analysis of PTC images may detect perfusion anomalies in migrainous aura that are not highlighted in the routine PCT images analysis.

**Patients and Methods:** Patients who presented a focal neurological deficit compatible with migraine with aura were enrolled. All the patients performed PTC during migrainous aura and no perfusion abnormality was found at first visual assessment. For each patient, a cerebral region of interest (RoI) was placed by two blinded neuro-radiologists according to the symptoms of the patient. As MTT maps are the most reliable in analysis of hypoperfusion [7], a quantitative analysis of mean transit time (MTT) maps on the RoI, voxel per voxel, by a semi-automatic algorithm was made. Data were compared with the mirrored RoI in the unaffected hemisphere (mRoI) (Fig. 1). Demographic data, characteristics of headache, and asymmetry of MTT between RoI and mRoI [ΔMTT=(MTT RoI – MTT mRoI)/MTT mRoI*100)] were evaluated. Each patient provided a written consent that allowed the analysis of data for research purposes.

**Results:** Six patients were enrolled (4 F, 2 M, mean age 43±23 y). PTC was performed after 70±15 minutes from symptoms onset. All patients were migraineurs, two of them already suffered with migraine with aura. Characteristics of migrainous aura are shown in Table 1, 50% of patients had headache at the onset of aura. In all patients, MTT values increased in RoI compared to mRoI (mean ΔMTT=19.9% [1.8-60.4%]), without effect of time of PTC performance (Table 1).

**Conclusions:** An ad hoc quantitative analysis of PTC images during migrainous aura detects an increase of MTT in cerebral RoI that corresponds to hypoperfusion that was not highlighted in the routine PCT images analysis. The use of this quantitative analysis in clinical practice can reduce the percentage of migrainous aura false negatives.

**References**

[1] Krishnan P, Murphy A, Aviv RI. CT-based Techniques for Brain Perfusion. Top Magn Reson Imaging. 2017;26(3):113-119.

[2] Furlanis G, Ajčević M, Stragapede L, et al. Ischemic Volume and Neurological Deficit: Correlation of Computed Tomography Perfusion with the National Institutes of Health Stroke Scale Score in Acute Ischemic Stroke. J Stroke Cerebrovasc Dis. 2018. Doi:10.1016/j.jstrokecerebrovasdis.2018.04.003

[3] Nieuwkamp DJ, van der Schaaf IC, Biessels GJ. Migraine aura presenting as dysphasia with global cognitive dysfunction and abnormalities on perfusion CT. Cephalalgia 2010;30:1007-9.

[4] Hansen JM, Schytz HW, Larsen VA, Iversen HK, Ashina M. Hemiplegic Migraine Aura Begins With Cerebral Hypoperfusion: Imaging in the acute phase. Headache, 2011;51:1289-1296.

[5] Shah L, Rana S, Valeriano J, Scott TF. Reversible CT perfusion abnormalities in patient with migraine variant: a two phase process. Clin Neurol Neurosurg. 2013;115(6):830-832

[6] Ridolfi M, Granato A, Polverino P, et al. Migrainous aura as stroke-mimic: The role of perfusion-computed tomography. Clin Neurol Neurosurg. 2018;166:131-135

[7] Floery D, Vosko MR, Fellner FA, et al. Acute-onset migrainous aura mimicking acute stroke: MR perfusion imaging features. AJNR Am J Neuroradiol. 2012;33(8):1546-1552

**Fig. 1 (abstract P177).**
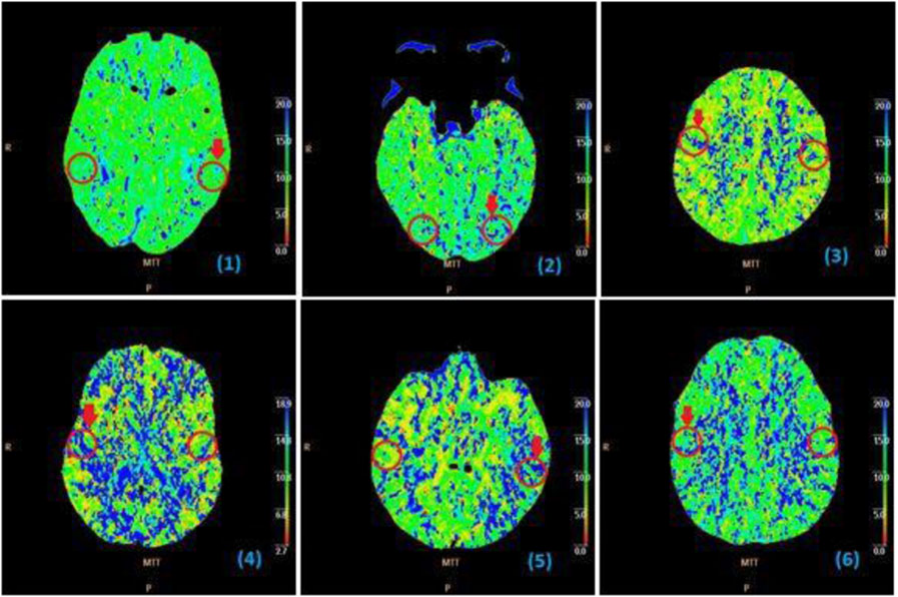
Perfusion Computed Tomography (Mean Transit Time images) performed during migrainous aura of the six patients. Circles with red arrows= symptomatic area; circles without red arrows= unaffected control area. Symptomatic area size= 1470 voxel

**Table 1 (abstract P177).** Clinical features of the six patients with migrainous aura

Patients	Age	Gender	History of migraine	Symptoms	Headache at the onset of symptoms	NIHSS	Time to PTC after symptoms onset	Symptoms duration	ΔMTT (%)
1	52	F	Yes, without aura	Right hemiparaestesia and dysphasia	Yes	2	60 min	2 hours	1,8
2	83	F	Yes, with aura	Aphasia	Yes	4	60 min	2 hours	13
3	49	F	Yes, without aura	left hemiparesis left sensitive-motor	No	3	90 min	2 hours	8,4
4	28	M	Yes, with aura	hemisyndrome and left homonymous hemianopsia	No	7	90 min	24 hours	30,2
5	21	M	Yes, without aura	Aphasia	Yes	1	60 min	2 hours	60,2
6	26	F	Yes, without aura	left hemiparesis	No	3	60 min	10 hours	6,1

(NIHSS=National Institutes of Health Stroke Scale; ΔMTT=(MTT RoI –MTT mRoI)/MTT mRoI * 100)

### P178 Cerebello-thalamo-cortical inhibitory activity impairment in patients with chronic migraine

#### Gianluca Coppola^1^, Clarissa Elizabeth Centurioni^1^, Chiara Abagnale^1^, Vincenzo Maria Parisi^2^, Mariano Serrao^1^, Cherubino Di Lorenzo^3^, Francesco Pierelli^1,4^

##### ^1^Sapienza University of Rome Polo Pontino, Department of Medico-Surgical Sciences and Biotechnologies, Latina, Italy; ^2^G.B. Bietti Foundation IRCCS, Research Unit of Neurophysiology of Vision and Neurophthalmology, Rome, Italy; ^3^Don Carlo Gnocchi Onlus Foundation, Milan, Italy; ^4^IRCCS-Neuromed, Pozzilli (IS), Italy

###### **Correspondence:** Gianluca Coppola (gianluca.coppola@gmail.com)

**Background**

Chronic migraine (CM) patients with or without medication overuse often present structural changes within the cerebellum. In healthy humans, the cerebellum exerts a suppressive effect on the contralateral motor cortical response, through a cerebello-thalamo-cortical pathway. Here, we investigate the cerebello-thalamo-cortical functioning in patients affected from chronic migraine.

**Materials and methods**

We recruited 19 patients affected from CM (11 without and 8 with medication overuse), and we compared them to a group of 18 healthy volunteers (HVs). After a conditioning single pulse high-voltage electrical stimulation delivered over the posterior surface of the mastoid processes (anode placed on the right side, and the cathode on the left), a TMS pulse was delivered over the contralateral motor cortex with 5, 7, 10, and 15 ms interstimulus interval (ISI) in random order. Five stimuli were delivered at each ISI. Motor cortical excitability changes were evaluated by amplitude changes of EMG responses from the first dorsal interosseous muscle to motor cortical stimulation. Furthermore, we have tested correlations of neurophysiological parameters with CM clinical features.

**Results**

In HVs, suppression occurs at an ISI of 5 ms (-19.4%) and lasts a few milliseconds. In CM patients, conditioning electric stimulation over the cerebellum did not reduce the size of MEPs to test TMS of the motor cortex at conditioning-test intervals of 5 ms (+3.0%, p=0.022). In CM patients, the percentage of MEP suppression at 5 ms ISI negatively correlated with number of tablets taken per month (r= -0.473, p= 0.04).

**Conclusions**

We found neurophysiological evidence for cerebello-thalamo-cortical inhibitory pathway malfunctioning in CM, a chronic head pain condition where an abnormal structure of the cerebellum had been previously reported. Overall, we reason that abnormal macrostructural and functional patterns in the cerebellum might be involved in cue-elicited acute medication craving. Whether these functional abnormalities are due to primary abnormal cerebellar inhibitory dysfunction or are secondary to a disrupted cerebellar-thalamic-cortical connectivity, remains to be determined.

### P179 Transcranial direct current stimulation as add-on therapy in the detoxification of patients with chronic migraine and medication overuse headache: clinical and EEG findings

#### Roberto De Icco^1,2^, Cristina Tassorelli^1,2^, Irene De Paoli^1^, Raffaele Manni^3^, Michele Terzaghi^3^, Riccardo Cremascoli^2,3^, Giorgio Sandrini^1,2^, Grazia Sances^1^

##### ^1^Headache Science Center, IRCCS Mondino Foundation; ^2^Department of Brain and Behavioral Sciences, University of Pavia; ^3^Unit of Sleep Medicine and Epilepsy, IRCCS Mondino Foundation

###### **Correspondence:** Roberto De Icco (rob.deicco@gmail.com)

**Objectives**: Transcranial direct current stimulation (tDCS) has been tested with encouraging results in the management of different painful conditions such as fibromyalgia, trigeminal neuralgia, and migraine.

Chronic migraine with medication overuse (CM+MO) represents a challenging condition where discontinuation of overused drugs is effective in a fairly high percentage of patients, but burdened by relapse into the previous situation.

The aim of this study is to evaluate the efficacy of anodic tDCS as an add-on therapy to the conventional treatment in patients with CM+MO.

**Methods**: We enrolled twenty patients (16 females, age range 32-65) with CM+MO among those hospitalized at the C. Mondino Foundation of Pavia for a detoxification program. They were randomly assigned to 2 groups: subjects in the tDCS-Group underwent 5 daily sessions of anodic tDCS on motor primary area M1 (contralateral to the most affected side of pain or on the right side in case of bilateral pain); subjects in the Sham-Group underwent 5 daily sessions of sham stimulation.

Patients underwent EEG recordings immediately before and after the first session of tDCS (T0 and T1), immediately before and after the fifth session of tDCS (T2 and T3) and 1 month later (T4).

**Results**: The two study groups were comparable at baseline. In the tDCS-Group we found a significant reduction in the number of headache days/month, days and drug doses/month at T4 when compared to baseline (p=0.001, p=0.001, p=0.002, respectively). In the Sham-Group, only the reductions in days and drug doses/month were significantly reduced at T4 (p=0.002 and p=0.001, respectively). Indeed the number of headache days /month at T4 was significantly lower in the tDCS-Group respect to Sham-Group (p=0.032). The percentage of patients with a 50% or more reduction in headache days/month at T4 was 70% in the tDCS-Group and 12.5% in the Sham-Group (p=0.025).

As regards EEG data, we observed an increase in the alfa power spectrum in the tDCS-Group in the occipital and frontal regions. This increase was significant at T1 and T3 when compared to T0 and it was also significantly more marked when compared to Sham-Group at T1, T2 and T3.

**Conclusion**: Our findings suggest the clinical efficacy of anodic tDCS as an add-on to a detoxification protocol in patients with CM+MO. The observed potentiation of alfa rhythm after anodic tDCS may be explained by the clinical improvement, however it is tempting to hypothesize that it could reflect a modulatory effect on the pain matrix.

### P180 Ultrasound biomicroscopy detects secondary hemicrania continua attributed to hidden narrow angle glaucoma

#### A. Granato, A. Dinoto, C. Bertolotti, D. Stokelj, L. Antonutti, P. Manganotti

##### Department of Medical, Technological and Translational Sciences, Headache Centre, University of Trieste, Italy

###### **Correspondence:** A. Granato (antonio_granato@hotmail.com)

**Background**

Hemicrania continua (HC) is coded in ICHD-3 as a primary headache in the Trigeminal Autonomic Chephalalgias. Although many ophtalmological conditions may be related to HC, no case of secondary HC attributed to narrow angle glaucoma has been described yet.

**Case report**

A 39-years old Caucasian woman with history of low frequency migraine without aura accessed to the Emergency Department (ED) for the sudden onset of right frontal and retro-orbital continuous headache, with 6-7 daily exacerbations, which were associated with omolateral ptosis and lacrimation. Head CT scan was normal. Neurological examination evidenced right eyelid ptosis and conjunctival injection during pain exacerbations (Fig. 1). Because of unresponsiveness to analgesics, she was admitted to the Neurology Unit. Prednisone, Verapamil, Acetaminophen, transdermic Fentanyl and Diazepam were not effective. HC was suspected and intravenous Indomethacin 50 mg four times a day was started with only partial reduction of the intensity of continuous pain and of the number of daily exacerbations. Topiramate and Celecoxib were also added with no significant effect. During the hospitalization, the patient reported a slight loss of visual acuity in right eye. Therefore she performed brain MRI, ocular tonometry, fundoscopic examination and visual evoked potential which were normal. To rule out intermittent glaucoma, an ultrasound biomicroscopy was performed and bilateral narrowing of anterior chamber due to plateau iris was found. She was treated with bilateral YAG-laser iridotomy and she markedly improved. Continuous headache with neurovegetative symptoms and daily exacerbations disappeared (Fig. 1). Prophylactic therapy was then slowly interrupted. At a fourth-month follow-up visit she complained only her usual episodic migraine she already suffered.

**Conclusions**

Ultrasound biomicroscopy may be a useful and safe tool to detect hidden narrow angle glaucoma as a possible cause of secondary hemicrania continua, when fundoscopic examination and ocular tonometry are normal.

Consent for publication: The patient provided a written consent that allowed the analysis of data and images for research purposes.

**Fig. 1 (abstract P180).**
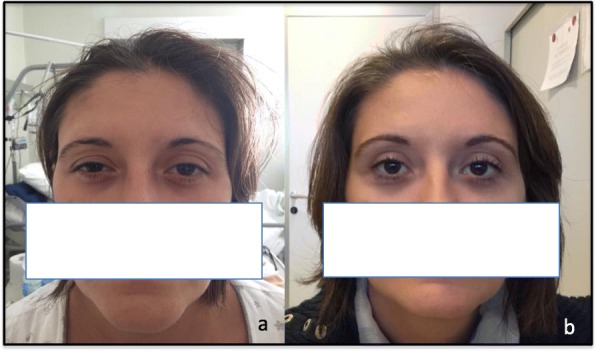
Right eyelid ptosis and conjunctival injection during pain exacerbations (a); normal eyes after bilateral YAG-laser iridotomy (b)

### P181 Analysis of Injection Site Reactions across Four Placebo Controlled Trials of Erenumab for Migraine Prevention

#### Julio Pascual^1^, David Doležil^2^, Brendan Davies^3^, Hernan Picard^4^, Frank Hong^5^, Feng Zhang^4^, Fei Xue^4^, Dan Mikol^4^, Jan Klatt^5^

##### ^1^Service of Neurology, University Hospital Marqués de Valdecilla and IDIVAL, Santander, Spain; ^2^DADO MEDICAL sro, Prague Headache Center, Prague, Czech Republic; ^3^Department of Neurology, Royal Stoke University Hospital, Stoke-on-Trent, UK; ^4^Amgen Inc., Thousand Oaks, CA, USA; ^5^Novartis Pharma AG, Basel, Switzerland

*Presented by Laura Bartolini – Medical Department Novartis Farma, Italy*

**Objective:** To assess the frequency of injection site reaction-related adverse events (ISR-AEs) observed in erenumab clinical trials in subjects with either episodic or chronic migraine.

**Background** Erenumab is a fully human monoclonal antibody that selectively inhibits the calcitonin gene-related peptide (CGRP) receptor and is under investigation for migraine prevention.

**Design/Methods** Data were obtained from four randomized, placebo-controlled trials (clinicaltrials.gov: NCT01952574, NCT02066415/NCT02174861, NCT02456740, and NCT02483585). Analysis was performed for the 12-week double-blind placebo-controlled treatment period (DBTP; erenumab and placebo) and the entire erenumab exposure period (EEP), including the open-label extension phase (erenumab only). AEs were graded according to the Common Terminology Criteria Version 4.03. The studies were approved by national Competent Authorities.

**Results** Over the 12-week DBTP, incidence of ISR-AEs was 3.2%, 5.6%, and 4.5% in the placebo, erenumab 70 mg, and 140 mg groups, respectively. Incidence of injection site pain, erythema and pruritus was comparable in the placebo, erenumab 70 mg, and erenumab 140 mg groups. Over the EEP, which extended erenumab exposure to median 46 weeks (mean 47 weeks, range 0–159), incidence of ISR-AEs was 6.1% and 4.2% in the erenumab 70 mg and 140 mg groups, respectively. Most ISR-AEs were mild (Grade 1). Moderate ISR-AEs (Grade 2) were injection site erythema (n=4, 0.2%), injection site pain (n=3, 0.1%), and injection site reaction, injection site induration, and injection site urticaria (n=1 each, <0.1%). There were no ISR-AEs of Grade >2, and no serious ISR-AEs. Across 2519 subject-years of erenumab exposure, one subject discontinued due to injection site pain, one due to injection site rash, and one due to injection site urticaria.

**Conclusion** ISR-AEs occurred in a small proportion of subjects treated with either dose of erenumab, with little change over time. Most ISR-AEs were mild and did not require discontinuation.

**Acknowledgements**

This study was supported by Amgen Inc. This study has been first presented at the 70th Annual American Academy of Neurology Meeting (Los Angeles, 21-27 April, 2018).

### P182 "Fusion Imaging” to identify Periaqueductal Gray Matter (PAG) in a Medication Overuse Headache (MOH) patient

#### Elena Guaschino^1,2^, Natascia Ghiotto^1,2^, Cristina Tassorelli^2,3^, Ana Bacila^4^, Daniele Bosone^1^, Grazia Sances^2^

##### IRCCS Mondino Foundation, Pavia; ^1^Neurovascular Ambulatory, Clinical Neurophysiology Unit; ^2^Headache Science Centre; ^3^Department of Brain and Behavioral Sciences, University of Pavia, Italy; ^4^Neuroradiology Department

###### **Correspondence:** Elena Guaschino; Natascia Ghiotto (natascia.ghiotto@mondino.it)

**Background**

The image fusion process is defined as gathering all the important information from multiple images and their inclusion into fewer images, usually a single one, that is more informative than any single source image. Fusion techniques allow direct and immediate comparison of ultrasound images with previously recorded radiological images.

The use of this technique in Neurosonology is recent and two authors have applied it to describe and identify intracranial vessels (1). Transcranial B-mode sonography (TCS) of brain parenchyma is increasingly used as a diagnostic tool for diagnosis and treatment monitoring of neurological diseases, particularly movement disorders.

In our Neurosonology Unit in collaboration with the Headache Science Centre, we evaluate the reproducibility of digitized TCS image analysis to identify the possible hyperechogenicity of PAG in migraine patients, as marker of metals deposition (2). PAG is an essential component in the pain modulation network and its dysfunction can play a role in the genesis of MOH (3). MRI studies suggested that an alteration of PAG iron homeostasis is present in migraine patient and this evidence correlates with the duration of the disease (4). Furthermore, MRI studies show PAG volume gain in MOH compared with normal controls (5) and decrease of grey matter in the midbrain only in MOH patients that have responded to detoxification (6). These data would suggest that PAG volume changes are an important imaging variable in MOH patients.

**Results**

Using the Esaote's Mylab Twice UltraNeuroNav system, we try to detect the PAG and then fuse echografic and MRI images. Our Institution Ethics Committee approved the study.

We examined a 64-year-old female patient with a long history of migraine without aura and MOH from 15 years. She first underwent TCS to evaluate the echogenicity of the transtemporal window; subsequently, a brain MRI was performed. Lastly, the ultrasound exam was recorded and superimposed on the MRI images.

The fusion of the ultrasound planes and the MRI sequences allowed us to establish whether the area we recognized as PAG on real-time US actually corresponded to the gray periacqueductale in the MRI images.

In our case, the juxtaposition of images confirmed that the hyperechogenic zone of the MRI corresponded effectively to the PAG.

**Conclusions**

These findings warrant further investigation and may be useful for lowering the cost of studying the pathways of nociception in MOH using TCS.

**References**

1. Malferrari et al, “Virtual navigator study. Subset of preliminary data about venous circulation” Proc 16 th Meeting of European Society of Neurosonology and Cerebral Hemodynamics, Munich, 2011; 67

2. Tepper SJ Tapper et Al., Iron deposition in pain-regulatory nuclei in episodic migraine and chronic daily headache by MRI. Headache. 2012 Feb;52(2):236-43

3. Guaschino E. Hyperechogenicity of the periaqueductal gray in chronic migraine and episodic migraine as a potential marker of progressive dysfunction: preliminary results with transcranial sonography. J Headache Pain. 2015 Dec;16(Suppl 1):A61

4. Welch KM et al., Periaqueductal gray matter dysfunction in migraine: cause or the burden of illness?, 2001 Jul-Aug;41(7):629-37

5. Chen Z et al., Volume gain of periacqueductal gray in medication-overuse headache. The journal of headache and pain 2017; 18:12

6. Riederer F. Decrease of Gray Matter Volume in the Midbrain is Associated with Treatment Response in Medication-Overuse Headache: Possible Influence of Orbitofrontal Cortex. The Journal of Neuroscience, September 25, 2013; 33(39):15343–15349

### P183 Early BoNT-A treatment after controlled withdrawal in Medication Overuse Headache: selection criteria for an optimal cost-benefit ratio

#### V. Rebecchi, L. Princiotta Cariddi, A.M Clerici, M. Mauri

##### Headache Center - Neurology and Stroke Unit, Osp. Di Circolo - University of Insubria, Varese, Italy

###### **Correspondence:** L. Princiotta Cariddi (luciapc86@hotmail.it)

**Background**

Chronic Migraine (CM) is a disabling condition: it is estimated to affect approximately 2% of the general population with a considerable burden for both the individuals affected and the healthcare system. Managing CM-MOH, particularly the long- lasting form, implies structured and expensive treatment protocols, mainly based on a preliminary detoxification program followed by reprophilaxis and -eventually - long-term BoNT-A treatment according to PREEMPT paradigm.

Previous studies from our group (V. Rebecchi L. P., 2016) (L. Princiotta Cariddi, 2017) have shown that a profile grouping CM-MOH patients on the daily profile of drug abuse intake is an important tool to predict the response to detoxification and to reprophilaxis.

Our observations have also demostrated that the percentage of long-term response to treatment programs for CM-MOH increases with the growing accuracy of patient’s selection and the optimal set-up of protocols; furthermore controlled withdrawal is an important step of our treatment protocols, but without a significant influence on the response to the successive BoNT-A treatment (V. Rebecchi L. P., 2017).

**Results**

The selection of patients is the key for the successful use of BoNT-A in the management of CM. From the analysis of our data of a series n.78 consecutives cases with a follow-up at least 12 months (L. Princiotta Cariddi, 2017) (V. Rebecchi L. P., 2017) (V. Rebecchi L. P., 2016) we have identified possible predictors of long-term efficacy of controlled withdrawal program followed by start of BONT-A treatment, also combined with long-term supportive care. These predictors may allow the reduction of treatment failures (i.e drop-out from follow-up recurrence of drug overuse) and the identification of the cases in need of an early start of BONT-A treatment (within 4-6 weeks after the withdrawal period). This soubgroup of patients is chactacterized by a longer exposure to medication overuse at observation, a specific profile of daily intake of overused medications and also by one or more previous failures after controlled withdrawal in their personal history.

**Conclusions**

In the perspective of limited resources it is necessary to increase the accuracy in the selection of those patients with CM in need of been admitted to expensive and long-term treatment protocols, particularly those with long-lasting MOH and previous treatment failures (drop-out from protocols/short term recurrence of overuse). Our results support the role of some prognostic variables to be considered in order to reduce the risk of failure, particularly in long term follow-up. Early of BoNT-A is trongly indicated for a well identified subgroup of CM-MOH patients with a long cumulative exposure to drug abuse and higher number of previous failure from therapeutic protocols.

**References**

[1] L. Princiotta Cariddi, V. R. (2017). How the therapeutic response to BoNT-A in CM-MO is increased through adjustements of treatment protocols and more accurate evaluation and management of patients.

[2] V. Rebecchi, L. P. (2016). Chronic Headache with Medication Overuse: Usefulness of structured evaluation and patients' subgrouping in therapeutic perspective. *Journal of Headache Pain*.

[3] V. Rebecchi, L. P. (2017). Chronic Headaches with medication overuse (MOH): no significat influence of preliminary controlled withdrawal programs on the response to Onabotulinumtoxin A prophylaxis.

### P184 Efficacy of Erenumab for the Treatment of Patients With Chronic Migraine With and Without Aura

#### Messoud Ashina^1^, David Dodick^2^, Peter J Goadsby^3^, David Kudrow^4^, Uwe Reuter^5^, Stewart J Tepper^6^, Sunfa Cheng^7^, Dean K Leonardi^7^, Robert A Lenz^7^, Daniel D Mikol^7^

##### ^1^Danish Headache Center, Rigshospitalet Glostrup, Faculty of Health and Medical Sciences, University of Copenhagen, Copenhagen, Denmark; ^2^Mayo Clinic, Scottsdale, AZ, USA; ^3^NIHR-Wellcome Trust King’s Clinical Research Facility, Kings College London, London, UK; ^4^California Medical Clinic for Headache, Santa Monica, CA, USA; ^5^Charité Universitätsmedizin Berlin, Berlin, Germany; ^6^Geisel School of Medicine at Dartmouth, Hanover, NH, USA; ^7^Amgen Inc., Thousand Oaks, CA, USA

Presented by Annalea Conte – Medical Department Novartis Farma Italy

**Objective**

Report erenumab efficacy in patients with chronic migraine (CM) with and without aura.

**Material**

Subgroups with CM with and without aura from a phase 2 study (NCT02066415) were assessed.

**Methods**

Data from patients randomized to placebo or erenumab (70mg or 140mg QM) for 12 weeks, and with ≥1 migraine with or without aura during baseline (duration: 4 weeks) were used to assess changes in monthly migraine days (MMD), acute migraine–specific medication days (MSMD), and ≥50% responder rates (RR; patients achieving ≥50% reduction in MMD). Data based on history of aura were also analyzed. *P*-values are without multiplicity adjustment.

The study was approved by national Competent Authorities.

**Results**

Of 667 patients randomized, 49% (n=328) had ≥1 migraine with aura. Mean (SD) respective baseline MMD in patients with aura were 18.6 (4.5), 18.5 (4.1), and 18.1 (4.7) in placebo, 70mg, and 140mg groups (without: 17.8 [4.9], 17.5 [4.5], and 17.5 [4.7]). Erenumab 70mg or 140mg reduced MMD vs placebo in patients with aura (LS mean [SE]: −6.6 [0.7]; *P=*0.009 and −7.1 [0.6]; *P*<0.001, respectively, vs −4.5 [0.5]) and without (−6.6 [0.6]; *P*<0.001 and −6.1 [0.6]; *P=*0.002, respectively, vs −3.8 [0.5]). MSMD was also reduced with erenumab 70mg or 140mg vs placebo in patients with aura (−2.8 [0.4]; *P=*0.010 and −4.0 [0.4]; *P*<0.001, respectively, vs −1.5 [0.3]) and without (−4.0 [0.4]; *P*<0.001 and −4.3 [0.4]; *P*<0.001, respectively, vs −1.7 [0.4]). Compared with placebo, ≥50% RR was higher with erenumab 70mg and 140mg in patients with aura (23% vs 41%, OR [95% CI]: 2.5 [1.4, 4.4]; *P*=0.003 and 40%, 2.5 [1.4, 4.5]; *P*=0.002, respectively) and without (24% vs 39%, 2.0 [1.1, 3.5]; *P=*0.020 and 42%, 2.2 [1.3, 4.0]; P=0.006, respectively).

**Discussion**

Results based on history of aura showed a similar pattern.

**Conclusions**

These data indicate that erenumab has similar efficacy in patients with migraine with and without aura in terms of MMD, MSMD, and ≥50% RR.

**Acknowledgements**

This study was supported by Amgen Inc. This study has been first presented at the 70th Annual American Academy of Neurology Meeting (Los Angeles, 21-27 April, 2018).

### P185 Efficacy and safety of erenumab in episodic migraine patients with 2–4 prior preventive treatment failures: Results from the Phase 3b LIBERTY study

#### Uwe Reuter^1^, Peter J. Goadsby^2^, Michel Lanteri-Minet^3^, Michel Ferrari^4^, Shihua Wen^5^, Jan Klatt^6^

##### ^1^Deprtment of Neurology, Charité Universitätsmedizin Berlin, Berlin, Germany; ^2^NIHR-Wellcome Trust Kings Clinical Research Facility, King’s College London, London, UK; ^3^Department of Pain-CHU Nice, FHU Inov-Côte Azur University, Nice, France; ^4^Department of Neurology, Leiden University Medical Centre, Leiden, The Netherlands; ^5^Novartis Pharmaceutical Corporation, East Hanover, NJ, USA; ^6^Novartis Pharma AG, Basel, Switzerland

*Presented by Marco Andrè Bassano – Medical Department Novartis Farma, Italy*

**Objective:** To assess the efficacy and safety of erenumab in patients with episodic migraine who have failed 2–4 prior preventive migraine treatments (PMTs).

**Material and Methods:** LIBERTY (NCT03096834) was a 12-week, double-blind, randomized study. Patients (N=246) were randomized (1:1) to receive erenumab 140mg and placebo. The primary endpoint was the proportion of patients achieving ≥50% reduction in mean monthly migraine days (MMDs) during Weeks 9-12 (Month 3). Secondary endpoints included change from baseline to Month 3 in MMDs and monthly acute migraine-specific medication days (MSMDs) and safety/tolerability. The study was approved by national Competent Authorities.

**Results:** At baseline, proportion of patients who failed 2, 3, and 4 prior PMTs were 38.6%, 37.8%, and 22.8%, respectively. The mean (SD) MMDs and MSMDs were 9.3 (2.64) and 4.6 (2.89), respectively. At week 12, the proportion of patients achieving ≥50% reduction in MMD was higher in those treated with erenumab 140mg vs placebo (30.3% vs 13.7%; OR [95% CI]: 2.73 [1.43, 5.19]; p=0.002). At week 12, there were greater reductions in MMDs and MSMDs with erenumab 140mg vs placebo (mean difference [95% CI] in MMD: −1.61 [−2.70,−0.52]; p=0.004; mean difference (95% CI) in MSMD:−1.73 [−2.46,−1.01]; p<0.001). Safety and tolerability profile of erenumab was comparable to placebo. No patients in the erenumab group discontinued due to adverse events.

**Discussion:** Erenumab is a fully human monoclonal antibody that inhibits the canonical CGRP receptor. Clinical studies have demonstrated the efficacy and safety of erenumab in patients with episodic and chronic migraine. Current oral preventive therapies are associated with low adherence rates due to the lack of efficacy and/or poor tolerability. It is therefore important to assess the safety and efficacy of erenumab in patients who have failed multiple therapies. LIBERTY is the first migraine prevention trial of its kind conducted specifically in patients who have failed multiple prior preventive therapies, and have a high unmet need for additional treatment options. The study met its primary endpoint of percentage of patients on erenumab achieving at least a 50% reduction of migraine days versus placebo, and all secondary endpoints.

**Conclusion:** These results confirm the efficacy and safety of erenumab in this first dedicated study of a difficult to treat population with 2–4 prior preventive migraine treatment failures.

**Acknowledgements**

This study was supported by Novartis Pharma AG, Basel, Switzerland. This study has been first presented at the 70th Annual American Academy of Neurology Meeting (Los Angeles, 21-27 April, 2018).

### P186 Case Report: Chronic Migraine successfully treated with Cannabinoids

#### E. Dini, M. Cafalli, C. De Luca, F. Baldacci, S. Gori, U. Bonuccelli

##### Neurology Unit, Department of Clinical and Experimental Medicine, University of Pisa, Pisa, Italy

###### **Correspondence:** E. Dini (dini.elisa87@gmail.com)

**Introduction**. The use of Cannabis sativa plant for medical purposes dates back to ancient times. Due to their strong analgesic action, cannabinoids are still used for symptomatic and prophylactic treatment in many pain conditions. Phytocannabinoids such as THC (Δ-9-tetrahydrocannabinol) and CBD (cannabidiol), are effective in reducing pain and inflammatory damages, causing also plastic changes in brain areas implicated in pain transmission^1^. Empirical evidences suggest the utility of cannabinoids in treating migraine, but no controlled trials are available^2^.

**Case Report.** A 48-year-old man presented last July to headache outpatient clinic. He referred, since he was a young boy, recurring headache with unilateral, switching side, pulsating pain, associated with nausea/vomiting, photophobia and phonophobia; the pain exacerbates with routine physical activity. In his past medical history, he reported Chronic lymphocytic leukemia in hematological follow-up. A recent brain MRI scan was performed and resulted normal. The neurological examination of the patient was also normal. For the past two years the headache frequency has been >20 days/month, with indomethacin overuse. Along this period, the patient underwent prophylactic treatment with propranolol and flunarizine, both ineffective. Detoxification with dexamethasone was prescribed, along with prophylactic treatment with amitriptyline up to 30 mg/days. This therapy, although well tolerated, resulted ineffective in reducing headache frequency and intensity, so it was discontinued by the patient after 3 months. In November he started a prophylactic treatment, prescribed by a specialist from Analgesic Treatment Centre, with a galenic formulation of THC 6.5% and CBD 8%, 10 drops orally bid, with reduction in migraine frequency: 0-1 days/month in the last 4 months, as he reported during the last month headache outpatient clinic evaluation.

**Discussion.** Cannabinoids are a promising weapon in treating migraine, due to their analgesic, antiemetic and anti-inflammatory action. Supporting literature is limited to case reports and laboratory studies^1^. Further researches, especially randomized clinical trials, are needed to determine specific posology and prescription^3^, since low doses of cannabinoids lower migraine pain, while higher doses exacerbate it. Moreover, an important question to investigate in cannabinoids use for chronic pain syndromes, is the development of physical reliance and tolerance. CBD, unlike THC, does not have psychoactive effects, so a path for CBD-based drugs should also be explored. Formal approval of cannabinoids drugs for a certain number of medical conditions, may encourage further scientific research to establish effectiveness and safety of these compounds in migraine treatment, to uncover potential therapeutic effects still unknown.

**Consent for publication:** Informed consent was obtained from the patient for publication.

**References**

1. P. Leimuranta, L. Khiroug and R. Giniatullin. Emerging role of (Endo)Cannabinoids in migraine. Frontiers in Pharmacology, 2018 9:420 (1-7).

2. R. Greco and C. Tassorelli. Endocannabinoids and migraine. Cannabinoids in Neurologic and Mental Disease, 2015, chapter 7; pages 173-189.

3. S. Maione, B. Costa, V. Di Marzo. Endocannabinoids: a unique opportunity to develop multitarget analgesics. Pain. 2013; 154(Suppl 1):87–93.

### P187 Abnormal intracortical facilitation pattern in episodic migraine without aura: results of a paired-pulse TMS study

#### S. Ferlisi, G. Cosentino, W. Capitano, S. Di Marco, L. Pilati, S. Scardina, A. Torrente, G. La Bianca, B. Fierro, F. Brighina

##### Department of Experimental Biomedicine and Clinical Neurosciences (BioNeC), University of Palermo

###### **Correspondence:** S. Ferlisi (fransalvo1@gmail.com)

**Introduction**: Paired-pulse TMS paradigms can be used to test connectivity within the primary motor cortex in human subjects. [1]Aim of the present study was to provide additional information on short intracortical inhibition (SICI), long intracortical inhibition (LICI) and intracortical facilitation (ICF) using different intensities of the test stimulus (TS) in patients suffering from migraine without aura (MwoA).

**Methods**: We enrolled 24 patients suffering with episodic MwoA and 24 healthy subjects. Both patients and controls were randomly assigned to two groups: the first group underwent assessment of SICI and LICI, whilst in the second group we evaluated ICF. We assessed SICI, LICI and ICF at three different suprathreshold intensities of the TS (110%, 130% and 150% of the resting motor threshold). Interstimulus intervals (ISI) of 2 ms and 100 ms were used for testing SICI and LICI respectively, whilst ICF was carried out by using 10 msISI.[2]

**Results**: When testing ICF, maximum increase in conditioned MEP amplitude was observed in migraineurs at the lower stimulation intensity of the TS. This intensity was indeed unable to induce significant facilitation in the healthy subjects, where maximum facilitation was observed at the higher stimulation intensities. No significant differences were observed between patients and healthy subjects as regards SICI and LICI.

**Conclusion**: Our results strengthen the notion of altered tuning of cortical excitability in migraine. [3] In particular, we provide evidence of hyperresponsivity of the glutamatergic intracortical circuits that could be revealed only by using a low stimulation intensity.

**References**

[1] Ziemann U, Rothwell JC, Ridding MC. Interaction between intracortical inhibition and facilitation in human motor cortex. J Physiol. 1996;496 (Pt 3):873-81.

[2] Sanger TD, Garg RR, Chen R.Interactions between two different inhibitory systems in the human motor cortex.J Physiol. 2001;530(Pt 2):307-17

[3] Cosentino G, Fierro B, Vigneri S, Talamanca S, Palermo A, Puma A, Brighina F. Impaired glutamatergic neurotransmission in migraine with aura? Evidence by an input-output curves transcranial magnetic stimulation study. Headache. 2011;51(5):726-33.

### P188 Migraine with aura: a great mimicker or a misleading herald?

#### Alberto Terrin^1^, Anna Maria Basile^2^, Federico Mainardi^3^, Giorgio Zanchin^1^, Ferdinando Maggioni^1^

##### ^1^Headache Centre of the Veneto Region, Department of Neuroscience, University of Padova, Padova, Italy; ^2^Stroke Unit, St. Anthony Hospital, Padua, Italy; ^3^Headache Centre. Hospital SS. Giovanni and Paolo, Venice, Italy

###### **Correspondence:** Alberto Terrin (alberto.terrin89@gmail.com)

**Background**

Migraine with aura (MA) represents one of the most challenging differential diagnosis in front of a patient complaining about acute neurological symptoms. Distinguishing it from an acute ischemic stroke is not always possible in the setting of the Emergency Department, where the well-known adagio “time is brain” keeps a pivotal role. A recent revision of scientific literature about the so-called stroke mimics reaffirmed the safety of rt-PA administration in patients with MA [1].

**Case report**

A 76 years-old woman come to our attention with a history of MA (scintillating scotomas) since her thirties. In the last 15 years, she experienced recurrent episodes defined as transient ischemic attacks or minor strokes, characterized by dysarthria, aphasia, left or right body paresthesias and alternating side hemiparesis; their duration ranged from 30 minutes to more than 48 hours; their frequency progressively increased along with years. Thrombolytic treatment was administered in two occasions, once causing parenchymal hematoma. However, brain MRI scans never showed a recent ischemic lesion in the DWI sequence. Again, she came to our attention complaining about the sudden onset of paresthesias and numbness in the right arm and in the perioral region, followed by non-fluent aphasia. A migrainous headache had started a few hours before. 60 minutes after the symptoms’ onset, the neurological evaluation revealed a NIHSS of 5, while the urgent CT brain scan was unremarkable. She rapidly developed a right hemiplegia (NIHSS 20). An acute-phase EEG (Fig. 1), a DWI-negative brain MRI (Fig. 2), a PET-MRI (Fig. 3), the relatives’ report of a cognitive impairment and the almost complete resolution of her symptoms within 96 hours composed the puzzle. A genetic analysis revealed a mutation in the gene NOTCH3 (Ch. 19q12), confirming the clinical suspect of cerebral autosomal dominant arteriopathy with subcortical infarcts and leukoencephalopathy (CADASIL) [2].

**Discussion**

MA may mimic a stroke causing an improper thrombolytic treatment. Scientific literature advocates the safety of this improper administration, in front of a diagnostic doubt [1]. At the same time, MA can be a misdiagnosis in front of an acute ischemic stroke. Our case report makes the puzzle even more complex. MA, also with hemiplegic features [2-3], may herald the clinical and neuroradiological pattern of a clinically composite genetic pathology, as CADASIL [4]. Hemiplegic auras can hide small-vessel strokes, as shown in our case, with a stepwise progression in disability. In these cases, thrombolytic treatment can be dangerous, causing intracranial hemorrhage.

**Consent for publication:** Informed consent to publish has been obtained from this patient.

**References**

1. Terrin A, Toldo G, Ermani M et al. When migraine mimics stroke: A systematic review. Cephalalgia. 2018 Jan 1:333102418767999.

2. Burkett JG, Dougherty C. Recognizing CADASIL: a Secondary Cause of Migraine with Aura. Curr Pain Headache Rep. 2017 Apr;21(4):21.

3. Schon F, Martin RJ, Prevett M et al. "CADASIL coma": an underdiagnosed acute encephalopathy. J Neurol Neurosurg Psychiatry. 2003 Feb;74(2):249-52.

4. Dichgans M, Holtmannspötter M, Herzog J et al. Cerebral microbleeds in CADASIL: a gradient-echo magnetic resonance imaging and autopsy study. Stroke. 2002 Jan;33(1):67-71.

**Fig. 1 (abstract P188).**
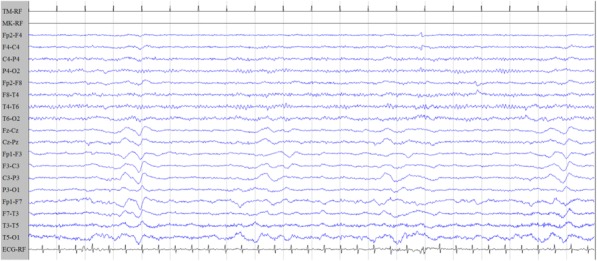
Acute-phase EEG showing a clearly asymmetrical pattern, with slow waves in the left hemisphere together with a minor amplitude of the tracks respect to the contralateral ones

**Fig. 2 (abstract P188).**
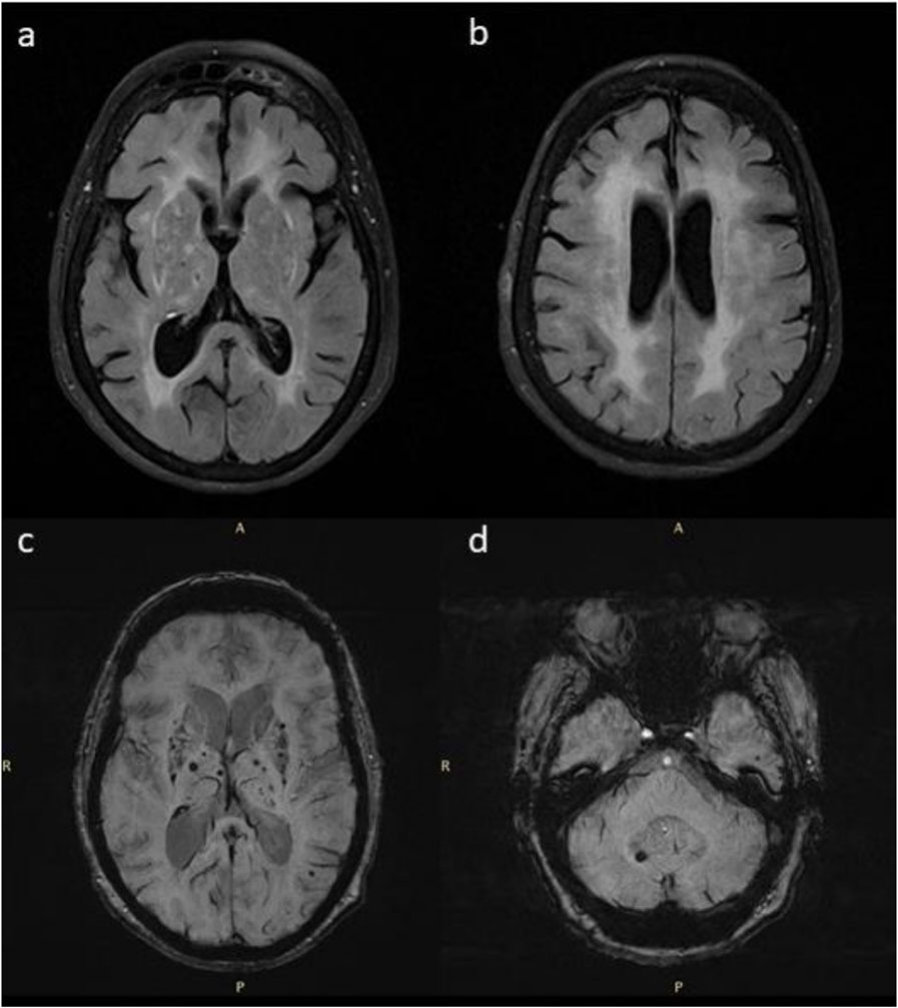
brain MRI scans: a-b) axial FLAIR sequences, showing diffuse white matter hyperintensity, including external capsules. C-d) Susceptibility-weighted imaging (SWI) showing hemosiderin-depositions in the basal ganglia (thalami included) and in the right cerebellum

**Fig. 3 (abstract P188).**
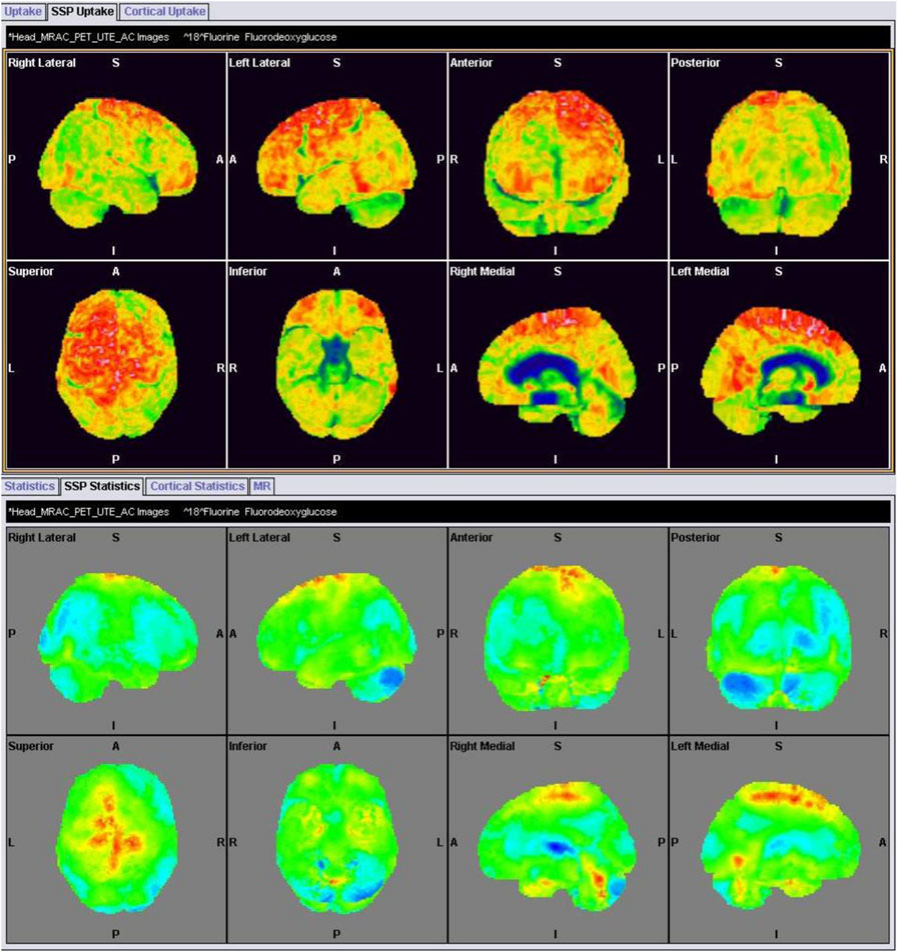
PET-MRI showing a right parieto-temporal and frontal hypo-metabolism; a bilateral hypo-metabolism of cingulate cortex is visible, as well.

### P189 Central and peripheral FAAH inhibition and migraine pain: potential mechanisms and targets in an animal model

#### Rosaria Greco^1^, Chiara Demartini^1^, Anna Maria Zanaboni^1,2^, Elena Tumelero^1^, Alessandra Misto^3^, Angelo Reggiani^3^, Daniele Piomelli^4^, Cristina Tassorelli^1,2^

##### ^1^Laboratory of Neurophysiology of Integrative Autonomic Systems, Headache Science Centre, IRCCS Mondino Foundation, Pavia, Italy; ^2^Department of Brain and Behavioral Sciences, University of Pavia, Italy; ^3^Dept. of Drug Discovery and Development, Istituto Italiano di Tecnologia, Genova, Italy; ^4^Department of Anatomy and Neurobiology, University of California, Irvine, CA, USA

###### **Correspondence:** Rosaria Greco (rosaria.greco@mondino.it)

**Background** - Fatty-acid amide hydrolase (FAAH) is an intracellular enzyme that catalyzes the cleavage of several endogenous fatty acid amides, including the endocannabinoid anandamide (AEA). In a previous study we reported that inhibition of peripheral FAAH, with the consequent increase in peripheral levels of AEA, reduces hyperalgesia as well as c-Fos expression in the nucleus trigeminalis caudalis (NTC) and the locus coeruleus in a well-known animal model of migraine based on nitroglycerin (NTG) administration.

**Methods -** Here, we tested the potential anti-hyperalgesic effect of URB597 (2mg/Kg, i.p), a global FAAH inhibitor , in male Sprague-Dawley rats made hyperalgesic by NTG injection (10mg/kg, i.p.) during orofacial formalin test. We also evaluated mRNA levels of candidate mediators of migraine pain in areas involved in trigeminal nociception and c-Fos protein expression in brain nuclei involved in migraine pain. Finally, we also tested the effects of ARN14633 (1mg/Kg, i.p), a new global FAAH inhibitor, with a better plasma metabolic stability compared to the URB597.

**Results -** URB597 administered 1h before NTG significantly inhibited NTG-induced hyperalgesia in Phase II of orofacial formalin test. Additionally, it decreased nNOS, CGRP and cytokines gene expression in peripheral and central tissues, while reverting NTG-induced c-Fos immunoreactivity in the NTC. No change was instead observed when URB597 was administered 3 hours after NTG. Interestingly, ARN14633 administered 3 hours after NTG significantly inhibited NTG-induced hyperalgesia and reduced cytokines gene expression in medulla, cervical spinal cord and trigeminal ganglion.

**Conclusion -** The findings confirm that inhibition of FAAH activity represents a promising target for modulating the pathways that are likely involved in migraine pain.

**Ethics approval**

The study was approved by the Italian Ministry of Health (Document number 1239/2015PR) and supported by a grant of the Italian Ministry of Health (Ricerca Finalizzata, RF-2013- 02355704 ) to the IRCCS Mondino Foundation.

### P190 Possible effects, side-effects and adverse events of a selective cannabinoid (6% THC/7.5% CBD) in refractory chronic migraine. 2013-2018 pilot data

#### Maria Nicolodi^1^, Maria Stella Pinnarò^1,2^, Vanessa Sandoval^1^, Anna Torrini^1^

##### ^1^Foundation Prevention and Therapy Primary Pain and Headache, Florence 50125, IT; ^2^Department Neuroscience, University of Florence, Florence 50139 Florence, IT

###### **Correspondence:** Maria Nicolodi (info@fondazionesicuterinicolodi.it); Maria Stella Pinnarò

**Background**: Laboratory data enlighten capacity of cannabinoids to control neurogenic pain [1].

Migraine sufferers refractory to conventional therapies as topiramate and amitriptyline, volunteered a treatment with selective cannabinoids.

**Method**: optimal THC-CBD dosage is chosen as previously indicated [2]. Dosage= 200 mg/day/orally=13mg THC+15mg CBD.

Volunteers: Migraine sufferers (n=311) showing 15/18 migraine days/monthly. Exclusion criteria: Systemic, organic, psychiatric diseases, addiction to recreational drugs.

Group A (n=211; 112 males, 99 females, mean age 35.7±16.3SD) used R/cannabis flos (Bediol 6%THC-7,5%-HPLC verified). Control group consisted of 120 volunteers (62 males, 58 females, mean age 42,7±14,1SD) who choose to re-test amitriptyline 50 mg/day/orally. Drugs were increased starting from 20 and 10 mg, respectively.

**Design**: 15 days run-in, 3-months treatment with 6%THC-7.5%CBD or amitriptyline. Perspective observation started in 2013- 52 weeks minimum period. Observations performed in agreement with Helsinki Declaration (FE 11/0/2013)

**Results:**

Possible Effects and Adverse Events: THC/CBD vs. Amitriptyline (Group 1)

Decrease of migraine day versus run-in: THC-CBD -47.05±6.8SD migraine hours/month vs. Amitriptyline= -17.6± 10.9SD p>00001

Table of Side effects

Serious adverse events or Deaths n= 0 THC-CBD n= 0 Group 1

Discontinuations due to adverse events n= 1 THC-CBD n =1 Group 1

Moderate Withdrawal or need to increase dosage n= 1 THC-CBD n=1 Group1 resolution in 10 days

Sexual functioning – sexual performances flow chart- n=0 THC-CBD n=1 Group 1

Changes in blood pressure n=0 THC-CBD, n=0 Group 1

Cardiopalmus n= 0 THC-CBD, n= 4 Group 1

Hearth rate changes ECG documentation n=0 THC-CBD n=1 Group 1

Nausea = 1 THC-CBD n=3 Group 1

Stomachache n=0 THC-CBD n=2 Group 1

Gut pain/dysfunction n=5 Group 1

Confusion n= 3 THC-CBD n=2 Group 1

Somnolence n= 1 THC-CBD n=12 Group 1

Fatigue n= 1 THC-CBD n=13 Group 1

Sleep disturbances n=1 THC-CBD n=0 Group 1

Memory (Randt Memory Battery)

amelioration n= 2 THC-CBD n=0 Group 1

worsening n= 2 THC-CBD n= 2 Group 1

General well being n= 79 THC-CBD n=1 Group1

**Conclusion**

Selected cannabinoid in selected population seemingly does not induce heavy side effects when compared to amitriptyline. Cannabinoids might be of interest in chronic migraine therapy

**References**

1.Nicolodi M, Sandoval V. Therapeutic use of cannabinoids -Dose finding, pilot data of effects in chronic migraine and cluster headache. Proceedings EAN Congress, Amsterdam 24-27 June 2017.

2. Russo E. Cannabinoids in the management of difficult to treat pain Ther Clin Risk Manag. 2008 Feb; 4(1): 245–259.

### P191 Physical Therapy and Onabotulinumtoxin A in chronic migraine: a three-arm randomized perspective study

#### A. Granato^1^, M. Deodato^2^, UG. Sisto^1^, C. Borgino^2^, L. D’Acunto^1^, P. Manganotti^1^

##### ^1^Department of Medical, Technological and Translational Sciences, Headache Centre, University of Trieste, Italy; ^2^Department of Experimental and Clinical Medicine and Experimental and Clinical Neurosciences, Physiotherapy, University of Trieste, Italy

###### **Correspondence:** A. Granato (antonio_granato@hotmail.com)

**Introduction**

Efficacy of physical therapy (PT) in chronic migraine (CM) is controversial, on the contrary Onabotulinumtoxin A (BoNT-A) has proven to be effective. Aim of the study is to evaluate the efficacy of PT in monotherapy or in combination with BoNT-A in the treatment of CM.

**Material and methods**

We perform a perspective three-arm randomized study of patients suffering from CM admitted to the Headache Centre of the University of Trieste. After one-month observation period (baseline), all the patients were treated with BoNT-A, PT or combined therapy BonT-A *plus* PT (CT). BoNT-A was administered following the standardized Botox protocol. PT consists in exercises and manual treatment on trigger points, reinforcement, postural modification, postural advice and relaxation training. Number of responders (>50% reduction of headache days), headache days (HD), headache hours (HH), symptomatic drug intake (SDI), disability (MIDAS) were analysed in baseline and at the three-month follow-up visit.

**Results**

We enrolled 34 patients (29 F, 5 M; mean age 53 [24-79] years), 14 patients were treated with BoNT-A, 10 patients with CT, 10 patients with PT. All the three groups of had a significant reduction of number of HD (BoNT-A: Baseline= 23 [11-30] vs Three-month visit= 15 [4-30] (p=0.001); CT: Baseline= 22 [13-30] vs Three-month visit= 15 [3-29] (p=0.002); PT: Baseline= 20 [16-30] vs Three-month visit= 12 [3-28] (p=0.002)), SDI (BoNT-A: Baseline=20 [6-57] vs Three-month visit=12 [0-21] (p=0.02); CT: Baseline=25 [11-41] vs Three-month visit=12 [1-41] (p=0.02); PT: Baseline=22 [12-45] vs Three-month visit=17 [2-43] (p=0.03)), and MIDAS (BoNT-A: Baseline= 103 [62-210] vs Three-month visit= 57 [10-179] (p<0.001); CT: Baseline= 86 [35-152] vs Three-month visit= 60 [12-187] (p=0.04); PT: Baseline= 93 [17-180] vs Three-month visit= 42 [2-109] (p=0.002)). HH reduced only in the group of CT (Baseline=213 [71-487] vs Three-month visit=124 [28-417] (p=0.02)). Number of responders and reduction of HD, SDI and MIDAS were comparable in three groups (p=NS). Each patient provided a written consent that allowed the analysis of data for research purposes.

**Conclusions**

Our data evidenced that physical therapy is effective in treating chronic migraine. Combined treatment of BoNT-A and physical therapy increases the reduction of the overall headache hours.

### P192 Visual cortical hyperactivity to sound induced flash illusions in pediatric migraine

#### Salvatore Di Marco^1^, Alice Pavone^1^,Giuseppe Cosentino^1^, Laura Pilati^1^, Angelo Torrente^1^, Simona Maccora^1^, Giuseppe Santangelo^2^, Vincenzo Raieli^2^, Serena Scardina^1^, Brigida Fierro^1^, Filippo Brighina^1^

##### ^1^Department of Experimental Biomedicine and Clinical Neuroscience (BioNeC), University of Palermo; ^2^Child Neuropsychiatry Unit, Di Cristina Hospital, ARNAS CIVICO Palermo

###### **Correspondence:** Salvatore Di Marco (dimarcosal@gmail.com)

**Background:** The number of sound-induced flash illusions (SIFI) perceived are related with the level of visual cortex (V1) excitability [1]. We found a V1 hyperexcitability in adults migraneurs in response to SIFI [2]. Moreover, healthy children see more SIFI than adults [3].

**Aim:** To test the V1 excitability in children with migraine through SIFI.

**Participants:** Twenty-six migraineours children (examinated interictally) and fifteen healthy children with no familiarity for migraine were evaluated.

**Methods:** Different combinations of visual (flash) and sound (beep) stimuli are presented: multiple flashes with a single beep cause perception of less flashes (fusion illusion) while multiple beeps and single flash, induce perception of more flashes (fission illusion). Each combination was randomly presented and the subject had to indicate the number of the flashes seen.

**Results:** Children with migraine do not differ from age matched control in the illusory percept, but they perceive more flashes in multiple flash trials(p<.05), that was confirmed by the variable mean isolated flash perception (MIFP) (p<.0052) .

**Conclusions:** Migraine children have an increased ability to perceive flashes, even outside migraine attack, that reveal a hyper-functional visual cortex in migraine also in pediatric age. SIFI can be used in pediatric migraine for testing the responsivity of V1.

**References**

1. Bolognini N, Rossetti A, Casati C, Mancini F, Vallar G. Neuromodulation of multisensory perception: a tDCS study of the sound-induced flash illusion. Neuropsycologia2011, 49:231-237.

2. Brighina F, Bolognini N, Cosentino G, Maccora S, Paladino P, Baschi R, Fierro B. Visual cortex hyperexcitability in migraine in response to sound-induced flash illusions.Neurology 2015 84: 2057-2051.

3. Nava E, Pavani F. Changes in Sensory Dominance During Childhood: Converging evidence From the Colavita Effect and the Sound induced Flash Illusion. Child Development. 2013, 84: 604-616.

### P193 Clinical features of pediatric medication overuse headache and applicability of new ICHD-3 criteria

#### Romina Moavero, Maddalena Stornelli, Laura Papetti, Barbara Battan, Samuela Tarantino, Federico Vigevano, Massimiliano Valeriani

##### Headache Center, Bambino Gesù Children’s Hospital, IRCCS, Rome, Italy

###### **Correspondence:** Romina Moavero (rominamoavero@hotmail.com)

**Objectives**. To describe the clinical phenotype of pediatric medication overuse headache (MOH) and to analyze the applicability of ICHD-3 criteria in comparison to the ICHD-2. MOH is characterized by headache occurring on ≥15 days/month in patients with pre-existing primary headache and developing as a consequence of regular overuse of symptomatic headache medication.

**Materials and Methods**. We conducted a retrospective analysis of clinical data of pediatric patients diagnosed with MOH in our Department. In all patients the clinical diagnosis of MOH was verified both according to ICHD-2 and ICHD-3 version criteria, to verify the degree of concordance.

**Results**. We identified 42 subjects diagnosed with MOH (31 F, 11 M), ranging between 8 and 17 years of age (mean 13.4 years). All patients presented with chronic migraine, with 9% fulfilling a diagnosis of migraine with aura. As for the clinical features of migraine, photo- and photophobia were both present in 81% of patients, nausea/vomiting in 30%, dizziness in 18%. Regarding the applicability of the ICHD-2 criteria, 21/42 (50%) fulfilled criterion A; 35/42 (83%) criterion B, 37/42 (88%) criterion C, and 23/42 (55%) criterion D. On the other hand, applying the criteria of ICHD-3, criterion A was fulfilled by 40/42 patients (95%), criterion B by 35/42 (83%), and criterion C by 40/42 (95%).

**Discussion**. Our data show that in comparison with ICHD-2, ICHD-3 criteria are satisfied by a higher rate of pediatric patients clinically diagnosed with MOH. The old criteria required a development or marked worsening of the headache during medication overuse, and a resolution within 2 months after medication withdrawal. Both these criteria disappeared in the new version of ICHD, being replaced by the requirement that the condition should be not better accounted by another ICHD-3 diagnosis. This means that now a diagnosis of MOH could be made in presence of a high frequency headache in a patient presenting a medication overuse, without the necessity of demonstrating a clear and direct correlation with abuse and discontinuation of symptomatic medications.

**Conclusion**. Our data show that ICHD-3 criteria allow a definite diagnosis of MOH in a higher rate of pediatric patients

### P194 C677T Methylenetetrahydrofolate reductase homozygous mutation: vitamin supplement in migraineur children

#### Elisabetta Tozzi, Giulia Iapadre, Luca Zagaroli, Agnese Onofri

##### Neuropsychiatric Clinic- Headache Center- San Salvatore Hospital -University L'Aquila Italy

###### **Correspondence:** Elisabetta Tozzi (elisabetta.tozzi@univaq.it)

**Background** TheMTHFR variant C677T has been associated with an increased genetic risk in migraine susceptibility, particularly for migraine with aura. Individuals with the homozygous state for this mutation show higher levels of plasma homocysteine (Hcy) [1]

A**im of study** is to evaluate if vitamin B9, B6 and B12 supplement in that homozygous condition in Migraine without and with aura ( MwoA and MA) is able to decrease the headache disability.

**Materials and Methods**

The open-label study evaluating clinical trial concern 236 children,aged 6-17 years, admitted to the Headache Regional Centre and recruited according to temporal criteria by observations sequentially during the years 2015, 2016 and 2017.These underwent to baseline plasma Hcy assessment (chemiluminescent immunoenzymatic assay) and screening for MTHFR ( Real-Time Polymerase Chain Reaction ). 31 patients with MTHFR homozygous mutation were selected and underwent clinical evaluation of migraine characteristics (frequency, severity of pain and use of acute treatment) at baseline and after a 12 month-period of daily supplement of vitamins B9 (2 mg) B6 (25 mg), B12 (400 mcg). In 20 patients with hyper Hcy (>95° percentile for age) the second collection of blood sample was obtained for Hcy assessment. Diagnosis of headache was made according to ICHD-III criteria. Informed consent was obtained for participation in the study by the parents of the children. Statistical analysis is made by Wilcoxon test(ANOVA).

**RESULTS**

20 children suffer from MwoA and 11 from MA. 86% of children aged >10 years had significantly higher Hcy values compared with controls (p <0.0001). 21 patients (70%), 14 females and 7 males have Hcy values > 95°percentile. Among this cohort the diagnosis was MwoA in 13 and MA in 8 patients. The values of Hcy after 12 months of vitamin supplement were reduced in the normal range for age. Hcy levels were strictly related to the age with a regression index of 0.557 (p <0.05). In all the sample the vitamin supplementation was effective in producing significant reduction in Hcy levels (p=0.0001) and a significant improvement of migraine disability in terms of frequency (p<0.0001), severity of pain (p<0.0003) and use of acute treatment (p<0.0001).

**Conclusion:** We underline the efficacy of vitamin supplement in lowering Hcy plasma levels and migraine disability in this condition not infrequent.

**References**

1. Menon S et al The effect of 1 mg folic acid supplementation on clinical outcomes in female migraine with aura patients. 2016; J Headache Pain.17(1):60.

### P195 The use of nutraceuticals in childhood and adolescence headaches

#### Agnese Onofri, Martina Mazzilli, Cristina Gammella, Giulia Iapadre, Elisabetta Tozzi

##### Neuropsychiatric Clinic- Headache Center- San Salvatore Hospital -University L'Aquila Italy

###### **Correspondence:** Agnese Onofri; Elisabetta Tozzi (elisabetta.tozzi@univaq.it)

**Background** Recently the use of nutraceuticals in prophylaxis and attack therapy of headaches is spreading both in adulthood and in childhood age [1].

**Aim of study** is to evaluate and to compare various nutraceuticals in the headaches prophylaxis

**Materials and methods** 99 children, 6-17 years, F 44 and M 55, suffering from Primary Headaches and admitted to Headache Center in the years 2016- 2017 are the sample. 7 patients were excluded because they did not adhere to the study due to lack of therapeutic compliance and because they did not return to clinical controls. The patients referred to the Headache center are selected consecutively. The open-label study evaluating clinical trial concerns the evaluation of the following parameters: headache diagnosis (ICHD-III,2013), migraine index: $\mathrm{MI}=\frac{n^{{}^{\circ}} crisis\ X\  pain\ intensity/ month}{30\kern0.75em days}$ ;EEG characteristics , the prophylaxis and attack therapies at time zero and after 6 and 12 months of therapy. Prophylaxis therapy = Mg citrate, Mg oxide and Mg aspartate(compound n° 1) vs Bisglycinate Mg + L-Tryptophan + Niacin + B2 Vit + D Vit(compound n° 2) and vs Oxide Mg + Partenium + Andrographis paniculata + coenzyme Q10, B2 Vit(compound n° 3) and vs Pidolate Mg + Ginkgo Biloba Phytosome, B2 Vit and Coenzyme Q10(compound n° 4). Attack therapy: Paracetamol, Ibuprofen, ketoprofen, Indomethacin. Informed consent was obtained for participation in the study by the parents of the children. Statistical analysis is made by Kruskal –Wallis test and analysis post hoc Conover .

**Results**: 22 F and 24 M suffer from Migraine without aura (MwoA), 9 F and 12 M From Migraine with Aura (MA), 11 F and 14 M from Tension Type Frequent Headache (TTH). The therapy as with compound n° 1 and as with compounds n° 2,3,4 is effective in reducing MI and reduces the use of attack therapy in all the cases very significantly (P = 0.00067). In MwoA (p = 0.000796) and to a lesser extent, in MA (P = 0.00442), the compound n° 1 is less effective than compounds n° 2 , 3 and 4. The compound 4 is not significantly effective. In TTH, compound 1 is less effective than compound 3 alone.

**Conclusion:** the use of nutraceuticals appears to be effective and also encouraging as it is well accepted by parents and children themselves.

**Reference:**

1. Orr SL .The Evidence for the Role of Nutraceuticals in the Management of Pediatric Migraine: a Review. 2018; Curr Pain Headache Rep.4;22(5):37

### P196 Changes on cervical range of motion in migraine patients after a strain counterstrain treatment

#### Valerio Giustino^1,2^, Valentina Contrò^3^, Jessica Brusa^1,2^, Patrizia Proia^3^, Giuseppe Messina^1,2,3^, Filippo Brighina^4^

##### ^1^Posturology and Biomechanics Laboratory Research Unit, University of Palermo, Palermo, Italy; ^2^Posturalab Italia Research Institute, Palermo, Italy; ^3^Department of Psychological, Pedagogical and Educational Sciences, University of Palermo, Palermo, Italy; ^4^Department of Experimental Biomedicine and Clinical Neurosciences (BioNeC), University of Palermo, Palermo, Italy

###### **Correspondence:** Valerio Giustino (valeriogiustino@msn.com)

**Background**

Migraine is a chronic neurological disorder that negatively influence the quality of life. This disabling neurologic condition is associated with irritation of head and neck sensory nerves. The aim of this study was to evaluate any changes on cervical range of motion (ROM) before and after a strain counterstrain (SCS) treatment, an indirect osteopathic manipulative technique that provides passive positioning to relieve tender points palpation pain, in migraine patients.

**Materials and Method**

A repeated measures within-subjects design was adopted for the study on a number of 25 participants affected by migraine (age: 43,24±15,61 yrs; height: 165,28±8,92 cm; weight: 70,2±18,99 kg) to measure the effects on cervical range of motion (ROM) before (T0) and after (T1) a SCS treatment. The SCS treatment provided a passive 90-second for tender point in the three most painful points, assessed with Visual Analog Scale (VAS). In the two identical testing sessions each participant, seated in a chair, performed neck movements on the three planes until the maximal ROM measured via a non-invasive technique using a wireless computer-aided accelerometer (Moover®; Sensor Medica®; Guidonia Montecelio, Roma, Italia) positioned medially of the frontal bone of the skull and above the bridge of the nose and fastened around the head via a strap. The evaluation provided three different and consecutive movements: maximal left and right rotation (LRR), maximal left and right lateral flexion (LRLF), maximal flexion-extension (FE) movements. All assessments were performed three times and the average values for each were used for statistical analysis.

**Results**

A paired-sample *t*-tests, with alpha level set at *p*<0.05, was executed for comparisons T0 and T1. Our results showed a significant increase for the left rotation in the pre-post interaction (*p*<0.05). No significant difference was shown between T0 and T1 for the other parameters (*p*>0.05).

**Conclusions**

It is known that migraine is associated with muscle pain in the neck region and patient who suffered of this disorder adopt an antalgic posture modifying the cranio-cervical posture. This condition causes asymmetrical muscular tensions. A possible explanation for the significant improvement on cervical rotation in patients with migraine may be explained because the SCS treatment technique decreased muscle stiffness caused by the irritation of head and neck sensory nerves. This treatment might have a positive impact on cervical mobility and involve an improvement on physical daily tasks in people who suffer of this disorder. Further investigation be conducted so as to confirm the hypothesis.

***References***

1. Blaschek A, Milde-Busch A, Straube A, Schankin C, Langhagen T, Jahn K, Schröder SA, Reiter K, von Kries R, Heinen F. Self-reported muscle pain in adolescents with migraine and tension-type headache. *Cephalalgia.* 2012;32(3):241-9.

2. Fernández-de-Las-Peñas C, Cuadrado ML, Pareja JA. Myofascial trigger points, neck mobility and forward head posture in unilateral migraine. *Cephalalgia.* 2006;26(9):1061-70.

**Ethics approval and consent to participation**

The study was approved by the ethics committee of the University of Palermo in conformity with criteria for the use of persons in research as defined by the Declaration of Helsinki.

All subjects provided written consent prior to participating in the study by undersigning an institutionally approved informed consent form.

### P197 The role of metacognitive executive dysfunctions in medication-overuse headache

#### Milena Zucca, Elisa Rubino, Alessandro Vacca, Flora Govone, Annalisa Gai, Paola De Martino, Silvia Boschi, Maria T. Giordana, Innocenzo Rainero

##### Neurology I, Department of Neuroscience “Rita Levi Montalcini”, University of Turin**,** Italy

###### **Correspondence:** Milena Zucca, Elisa Rubino (milena.zucca@unito.it)

**Background:**

Medication-overuse headache (MOH) has been characterized by headache occurring more than 15 days per month and by regular overuse of symptomatic medications. A poor decision making, associated to a fronto-striatal circuit dysfunction, has been described in patients with MOH. [1]. In literature, it was suggested that decision making is a complicated behavioral task that involves metacognitive executive functions in term of both the ability to evaluate one’s on-going cognitive activities (*monitoring*) and to use this metacognitive knowledge to regulate an on-going cognitive activity (*control*). At present, no data on metacognitive functions in adult MOH patients are available. The aim of our study was to analyze the role of metacognitive executive dysfunctions in patients with MOH.

**Material & Methods:**

Sixty-five patients were recruited at the Headache Center, Department of Neuroscience “Rita Levi Montalcini”, University of Torino: 37 patients [M/F = 9/28; mean age= 46.05 ± 11.32 years] fulfilled the criteria for MOH and 28 [M/F= 9/19; mean age= 45.11±12.18 years] for episodic migraine (EM)according to ICHD-3 [2]. Twenty-nine healthy controls (HC) [M/F= 12/17; age ±SD= 42.86±14.78 yrs] were also included in the study. Metacognitive executive functions were assessed using the metacognitive version of the Wisconsin Card Sorting Test (M-WCST) [3]. M-WCST is designed to yield also measures of free choice response that depend on the patient’s metacognitive knowledge. Different metacognitive indexes were achieved: accuracy score (AS); free-choice improvement (FCI); global monitoring (GM); monitoring resolution (MR); control sensitivity (CS) and monetary gains (MG).All statistical analyses were performed with SPSS version 21.0.Metacognitive aspects were compared between groups using ANOVA test with Bonferroni correction.

**Results:**

Patients with MOH showed worse performances respect toHC in M-WCST indexes such as: AS (p<.001), FCI (p=.02), GM (p<.001), MG (p=.001), and CS (p<.001). We also found statistically significant differences between MOH and EM in the same metacognitive indices, as AS (p<.001), FCI (p=.015), GM (p=.002), MG (p=.002), and CS (p<.001). Intriguingly, age and duration the disease did not influence the metacognitive performance.

**Conclusions:**

In conclusion our MOH patients showed a worse performance in metacognitive executive functions, especially in the term of control sensitivity. We can speculate that an impairment of the control process explain the reduction of strategic decision making and predisposes these subjects to a relapse of chronic substance abuse. Further studies would be helpful to shed light on the etiopathological role of metacognitive dysfunctions in dependency-like behaviors associated to chronic migraine.

**References:**

1. Biagianti, B., Grazzi, L., Gambini, O., Usai, S., Muffatti, R., Scarone, S., &Bussone, G. (2012) Orbitofrontaldysfunction and medicationoveruse in patients with migraine. Headache,52 (10), 1511–1519. doi: 10.1111/j.1526-4610.2012.02277.x

2. Headache Classification Committee of the International Headache Society. The International Classification of Headache Disorders, 3rd edition (betaversion). Cephalalgia. 2013;33:629-808.

3. Koren D, Seidman LJ, Goldsmith M, Harvey PD. Real-world cognitive– and metacognitive– dysfunction in schizophrenia: A new approach for measuring (and remediating) more “right stuff”. Schizophrenia Bulletin. 2006; 32(2), 310–326.

### P198 Aortic pulse wave velocity in children with migraine: a case- control study

#### Laura Pilati^1^, S. Di Marco^1^, A. Pavone^1^, S. Scardina^1^, G. Cosentino^1^, G. Mule'^2^, V.Raieli^3^, M.Gangitano^1^, B. Fierro^1^, F. Brighina^1^

##### ^1^Department of Experimental Biomedicine and Clinical Neurosciences (BioNeC) - University of Palermo – Palermo; ^2^Biomedical Department of Internal Medicine, and Specialist (DIBIMIS) - University of Palermo – Palermo; ^3^Child Neuropsichiatry unit, Di Cristina Hospital, ARNAS CIVICO Palermo

###### **Correspondence:** Laura Pilati (laura.pilati.91@gmail.com)

**Background:** Migraine has been associated with increased risk of cardiovascular (CV) accident like angina and myocardial infarction(1) . Vascular changes in migraineur traditionally may prevail in cranial blood district, but more likely it is a generalized vascular phenomenon.(2) Previous studies showed an increased aortic pulse wave velocity (aPWV), a direct measure of aortic stiffness and an independent predictor of stroke and CV disease, in young and middle-aged migraineurs(3) Here we hypothesized that, if associated with the pathogenetic bases of the disease, increased aPWV should be appreciable also in migraineurs children.

**Materials and Methods: :** We studied 10 children with migraine without aura (age 12,9±1,9 years, 7 male and 3 female, blood pressure 123,30±13,6mm Hg) and 6 age-, sex-, and blood pressure–matched healthy control children. In all participants, aortic PWV and aortic augmentation index were measured using an oscillometric technique**.**

**Results:** Children with migraine without aura had a higher a PWV (5.65 ± 0.60 vs 4.65 ± 0.42 m×s−1, *p* < 0.002) and aortic augmentation (15.07 ± 13.97 vs 3.00 ± 7,48 *p* < 0.037) than matched control children. While other variables potentially able to influence aortic distensibility, such as mean brachial arterial pressure and age, are not significantly different between the two groups. Similarly, no significant differences emerged as regards heart rate, diastolic arterial pressure and humeral differential and systolic and aortic pulsation.**.**

**Conclusion:** The present study showed higher aPWV in children affected by migraine without aura with respect to healthy controls. This result support the data of higher aPWV in migraine(3) and suggest that an increased aortic stiffness is already present in childhood. So, whatever the role played by such vascular alteration, it’s likely to be associated with basic mechanisms of migraine and could represent a new way to shed light on the complex yet unsolved pathophysiological network of the disease.

**References:**

1). Schurks M, Rist PM, Bigal ME, Buring JE, Lipton RB, Kurth T. Migraine and cardiovascular disease: systematic review and meta-analysis. BMJ 2009;339:b3914.

2). Silberstein SD. Migraine pathophysiology and its clinical implications. Cephalalgia 2004;24(suppl 2):2–7.

3).Schillaci G, Sarchielli P, Corbelli I, Pucci G, Settimi L, Mannarino MR, Calabresi P, Mannarino E. Aortic stiffness and pulse wave reflection in young subjects with migraine: A case-control study. Neurology. 2010 Sep 14;75(11):960-6.

### P199 Efficacy of KUZIK® in the prophylaxis of migraine without aura: a case report

#### E. Pucci^1,2^, R. Galante^3^

##### ^1^Headache Science Center – IRCCS Mondino Foundation, Pavia, Italy; ^2^Department of Brain and Behavioral Sciences, University of Pavia, Pavia, Italy; ^3^Gam Farma Srl. Milan

###### **Correspondence:** E. Pucci (ennio.pucci@mondino.it)

**Background**

A migraine without aura is the most common type of migraine headache ( about 60% to 80% of all migraines). In recent years there is growing interest in the use of nutraceuticals for the prevention of migraine, as there are no specific drugs for this indication. A new kudzu based food supplement (Kuzik) , thanks to its mechanism of action, has attracted our interest in this use.

**Materials and Methods**

25 year old woman. Familial family history of headache (paternal line). Onset of school age. Gradual increase after the menarche. Diagnosis according to the criteria ICHD-III beta-version: migraine without aura.Frequency: 4 crisis / month. VAS (Visual Analogue Scale): 7. Duration of the crisis: 24-72 hours. Disabling crisis on the first day of the menstrual cycle. Triggering factors: menstruation, ovulation, psycho-physical stress. drugs used as needed: fans with partial benefit. No preventive therapies.Total days / month with migraine: 10. Total number of symptomatic administrations:12. The patient performed complementary therapy with Kuzik 2 tablets in the morning on a fast.

**Results**

Kuzik was well tolerated: on a numerical scale from 1 to 10 the average score was 8 (range 6 -10); patient compliance was optimal, no side effects related to the product were reported, and no weight gain occurred in the patient. As far as efficacy is concerned, at the end of the treatment all the parameters analyzed were significantly improved: the average number of seizures was 2 (range 0-4), days with headache 4 (range 0-12) and medium VAS 3 (range 0-7).It should be noted that there was no disabling crisis at the beginning of menstruation and that there was a reduction, above all, of the intensity of the crises, associated with frequency and duration.

**Conclusions**

Our study has clearly demonstrated the benefits, safety, and good tolerability of Kuzik in the prophylaxis of migraine without aura and the use of this medication should be taken in consideration as a good practice in this setting of patients. Data from our study are encouraging to be confirmed by further investigations.

**Consent for publication:** Informed consent was obtained from the patient for publication.

**References**

Sewell RA et al. Response to Cluster Headache to Kudzu. Headache 2009; 49: 98-105.

### P200 Anxiety, depression and alexithymia in young migraine patients: which relationship with body weight?

#### Samuela Tarantino^1^, Alessandra di Stefano^2^, Laura Papetti^1^, Barbara Battan^1^, Giorgia Sforza, Federico Vigevano^1^, Simonetta Gentile^2^ and Massimiliano Valeriani^1,4^

##### ^1^Headache Center, Division of Neurology, Ospedale Pediatrico Bambino Gesù, IRCCS, Piazza Sant’Onofrio 4, Rome, Italy; ^2^Unit of Clinical Psychology, Ospedale Pediatrico Bambino Gesù, IRCCS, Piazza Sant’Onofrio 4, Rome, Italy; ^3^Child Neurology and Psychiatry Unit, Tor Vergata University of Rome, Rome, Italy; ^4^Center for Sensory-Motor Interaction, Aalborg University, Aalborg, Denmark

###### **Correspondence:** Samuela Tarantino (samuela.tarantino@opbg.net)

**Background** A growing body of literature explored the relationship between migraine and body weight. While several studies analyzed common pathophysiological mechanisms implicated in migraine and feeding regulation, data on the role of psychological factors are sparse, especially in pediatric age. Aims of the present study were to study 1) the prevalence of overweight in migraineurs children/adolescents; 2) the possible relationship between frequency of migraine and overweight; 3) the role of psychological symptoms (anxiety/depression) and emotional processing /regulating (alexithymia) on Body mass Index (BMI) in migraine patients.

**Methods** Patients were identified though a systematic review of clinical records of patients referred to our headache center from 2014 to 2017. We included a total of 122 migraineurs (m.a.12.8 ± 2.5 years; 55 M, 67 F) which were divided in two groups: Group-1, patients who underwent a screening for anxiety and depression (m.a 11.6 ± 2,6 years; M 24, F 43) and Group-2, those who were evaluated for alexithymia symptoms (m.a. 14.2 ± 1.6 years; M 31, F 24). Among the two different groups patients were divided according the BMI percentiles in “Normal weight”, “Overweight” and “Obese” (collapsed in the “Overweight” group). The psychological screening was assessed by SAFA (Anxiety-Depression scales) and TAS-20 questionnaires. The attack frequency was divided in high frequency (from weekly to daily episodes), and low frequency (≤ 3 episodes per month).

**Results** Sixty-four patients (60.7%) were classified as normal weight, 16.4% were obese and 22.9% overweight. The weight (normal or overweight) did not correlate with migraine frequency in none of the groups (respectively: Group-1: χ(2) = 0.998, p=0.317; Group-2: χ(2) = 0.151; p= 0.697). In Group-1, we found a significant higher score in “Separation anxiety” subscale (p=0.03) among overweight patients. No difference was found in SAFA-D subscales (SAFA-D Tot= 0.14) between normal and overweight groups. Analyzing Group-2, our data did not show a significant difference in alexithymia levels (TAS-20 Tot= 0.814) according the body weight. SAFA-A, SAFA-D and TAS-20 did not show any significant correlation with BMI (p>0.05).

**Conclusions** In our study, there is not a correlation between body weight and the frequency of migraine. Our results, however, suggest that overweight migraineurs patients are more prone to “separation anxiety”. In these children, food may alleviate loneliness and separation worries; on the other hand, we can suppose that overweight migraineurs patients are over-protected and pampered leading to a separation anxiety from their parents whenever a stressful situation may arise.

### P201 Does a dedicated social network help specialists’ training in the management of headaches? Comparison with other networks - a four-year experience

#### Laura Silvestri^1^, Carmela Loiacono^1^, Luca Messina^1^, Marco Micale^1^, Edvige Correnti^1^, Francesca Marchese^1,2^, Francesca Vanadia^2^, Filippo Brighina^3^, Vincenzo Raieli^2^

##### ^1^Child Neuropsychiatry School – University of Palermo; ^2^UO NPI- P.O. Cristina - ARNAS Civico Palermo; ^3^Department of Experimental Biomedicine and Clinical Neurosciences - University of Palermo

###### **Correspondence:** Vincenzo Raieli (vinzi.raielk@inwind.it)

**Background :** It is very important for specialists to have an adequate training for the management of headaches [1-3]. The increasing development of digital instruments (digital platforms, web sites , social networks etc) seems to be a great help, but is it really true? Our recent study [4] has pointed out that, after two years of experience, a digital platform does not stimulate the active management of headaches. Some possible explanations may be : 1) unlike the common thought, a social network on headaches does not affect the interested scientific community ; 2) the increasing use of smartphones and WhatsApp groups makes the use of a PC platform more obsolete and less attractive ; 3) more time is needed to become familiar with these new technologies ; 4) a digital platform dedicated to Headache is probably less interesting than platforms dedicated to other disorders.

To verify these hypotheses we have monitored the activity on the Headache’s digital platform in the last two years compared to the two previous years.

**Methods** : A digital platform has been active online since 1 October 2014. The platform is easily accessible for doctors via free registration. It is divided in two sections ( pediatric headaches and adult headaches). The uploaded resources consist of many different materials concerning headaches . Furthermore, members can discuss clinical cases . We have also created a WhatsApp group to alert members about each new contribution and to encourage real-time communication. From 1 January 2018 an app to access the platform from smartphone is available.

**Results:** After two years, platform’s members were 37 (14 in common for the two sections), while at the fourth year they are 67 (44 in both sections). 74 resources were uploaded during the first two years and other 64 during the other two (almost 90% only by one member ), while in the first two years the downloads were 486 (average: 13,1 downloads per member)active members were 22/37 (59.5%), 5 clinical cases were included in the platform ; in the second two years the downloads were 940 (average: 14 downloads per member), active members were 49/67 (71%), no further clinical cases were submitted. WhatsApp group’s members are 58, however 30/58 have participated actively with more than two interventions . The smartphone app has been downloaded by 16 members (16/58 ). Finally in (Table 1) is summarized the activity of other digital platforms dedicated to other clinical disorders (number of the members , uploads and downloads).

**Discussion**: After 4 years the monitoring of the platform activity shows that social networks dedicated to headaches are appreciated by the scientific community because we had an increase of about 80% of subscriptions to the platform , however the activity does not appear to increase significantly . These data confirm the limits in the efficacy of these digital instruments in the headache training for specialists. The fourth hypothesis is not supported by data coming from other platforms (see Table 1). To increase the use of these instruments and to ameliorate the specialists’ training is probably necessary to make their use mandatory or to encourage it through institutional and economical credits.

**References**

1. Brighina F. , Raieli V. , La Pegna G., F. Lanaia F.(2006) Disability and social impact of headaches and migraine. The role of information and cooperation among patient, general practitioner and specialist: A project of the SISC Sicilia. Giornale delle Cefalee 2:10-12.

2. Raieli,V. ,Compagno A., Puma D., La Vecchia M., Pandolfi E., La Franca G., Ragusa D. (2010) Headache: What do children and mothers expect from paeditricians? Headache 50: 2, 290-300;

3. World Health Organization (2011). Atlas of headache disorders and resources in the world. 2011 **ISBN**: 9789241564212

4. Raieli V, Correnti E, Sandullo A, Romano M, Marchese F, Loiacono C, Brighina .F. Effectiveness of a digital platform for sharing knowledge on headache management: a two-year experience. Funct Neurol. 2018;33:51-55.

**Table 1 (abstract P201).** See text for description

Total 48 groups	Downloads	Discussions	members	active members
JBOARD CLUB AlgologyGroups: 8	84	180	84	37
JBOARD CLUB Dermatology Groups: 3	13	8	33	16
JBOARD CLUB Hematology Groups: 4	19	1	82	19
JBOARD CLUB Digestive Endoscopy Groups: 1	0	3	17	8
JBOARD CLUB Gastroenterology Groups: 13	260	11	183	70
JBOARD CLUB Immunology Groups: 1	0	0	5	2
JBOARD CLUB Infettivology Groups : 2	0	0	3	2
JBOARD CLUB Nefrology Groups : 1	0	1	19	2
JBOARD CLUB Neurology Groups: 3	1437	54	121	98
JBOARD CLUB Oncology Groups: 8	163	9	86	42
JBOARD CLUB Psichiatry Groups : 2	32	1	201	45
Total	2008	268	834	341

### P202 VITAMIN D SERUM LEVEL IN HEADACHE PATIENTS: PRELIMINARY ANALYSIS

#### Vincenzo Pizza^1^, Silvana Montella^1^, Domenico Cassano^2^, Maria Cristina Lerza^3^, Vincenzo Busillo^3^

##### ^1^Neurology Unit, S. Luca Hospital, Vallo della Lucania (SA); ^2^60 District, Nocera Inferiore; ^3^Neurology Unit, Maria SS Addolorata Hospital, Eboli, ASL SALERNO, Italy

###### **Correspondence:** Vincenzo Pizza (vpizza@libero.it)

**Introduction:** headache pathogenesis is complex and still to be still to be clarified. Some nutrients, such as the Vitamin D, have a debated role in the development and chronicity of different headache types, in adults and children [1]. Decreased Vitamin D concentration was associated with chronic tension-type headache [2]. In another case series the Authors did not find any association between migraine and vitamin D status neither between severity of headaches and 25(OH)D level [3]. Interestingly, Vitamin D showed to have no role in other types of pain, for example chronic low back pain [4], while its contribute in pain deriving from tunnel carpal syndrome is still controversial [5]. Recent findings suggest a role of low vitamin D levels for raised central sensitivity, able to increase pain processing upon mechanical stimulation in chronic pain patients [6].

**Objective:** our aim is to determine 25(OH)D level in our headache patients and correlate them to age, headache type, headache duration

**Material and Methods:** we collected, in a small preliminary analysis, 25(OH)D level in 21 consecutive patients belonging to our headache centre, in San Luca Hospital, Vallo Della Lucania. 25-Hydroxy vitamin D [25(OH)D] plasma levels were measured by MicroVue 25-OH Vitamin D EIA. Fifteen patients were women, 6 were male; fourteen were diagnosed with migraine without aura, 3 had tension-type headache, 1 had cluster headache, 3 fulfilled criteria both for migraine without aura and tension-type headache; mean age was 38.6±17.5 years; mean disease duration was 10.4±8.6 years.

**Results:** Mean vitamin D serum level is 20.9±9 ng/ml, denoting a state of insufficiency. Among our 21 patients, only 2 have serum level above 30 ng/ml (sufficiency); most patients (16 out of 21) show serum level among 10 and 30 ng/ml, while in 3 patients serum level is less than 10 ng/ml. All these last three patients were diagnosed with migraine without aura.

**Conclusion:** Vitamin D insufficiency is a frequent finding in our headache patients, but we cannot postulate a definite relationship between vitamin D deficiency and headache. We suggest that this conclusion needs to be supported with randomised clinical studies containing a larger number of samples and controls. Moreover, intervention studies are required to find out if supplementation of vitamin D is effective in patients with different headache types.

**References**:

1. Donmez A, Orun E, Sonmez FM. Vitamin D status in children with headache: A case-control study. Clin Nutr ESPEN. 2018 Feb;23:222-227.

2. Prakash S, Rathore C, Makwana P, Dave A, Joshi H, Parekh H. Vitamin D Deficiency in Patients With Chronic Tension-Type Headache: A Case-Control Study. Headache. 2017 Jul;57(7):1096-1108.

3. Zandifar A, Masjedi SS, Banihashemi M, Asgari F, Manouchehri N, Ebrahimi H, Haghdoost F, Saadatnia M. Vitamin D status in migraine patients: a case-control study. Biomed Res Int. 2014;2014:514782.

4. Heuch I, Heuch I, Hagen K, Mai XM, Langhammer A, Zwart JA. Is there an association between vitamin D status and risk of chronic low back pain? A nested case-control analysis in the Nord-Trøndelag Health Study. BMJ Open. 2017 Nov 25;7(11):e018521.

5. Demiryurek BE, Sentürk A. Correlation of vitamin D levels with electrophysiological findings and pain in the patients with Carpal Tunnel Syndrome. Ideggyogy Sz. 2017 Sep 30;70(9-10):315-320.

6. Von Känel R, Müller-Hartmannsgruber V, Kokinogenis G, Egloff N. Vitamin D and central hypersensitivity in patients with chronic pain. Pain Med. 2014 Sep;15(9):1609-18.

### P203 Prescholar headaches and red flags in the Emergency Department

#### Flavia Drago^1^, Edvige Correnti^1^, Francesca Marchese^1^, Luca Maria Messina^1^, Marco Micale^1^, Lucia Rocchitelli^1^*,* Francesca Vanadia^2^, Vincenzo Raieli^2^

##### ^1^Child Neuropsichiatry Dept.- University of Palermo; ^2^Child Neuropsichiatr Unit, ISMEP- ARNAS Civico Palermo

**Introduction:** The role of red flags list in pediatric headaches is uncertain to establish if to perform neuroimaging studies in emergency department, except for some specific signs, such as papilledema or motor paralysis [1]. There are not studies specifically dedicated to prescholar headaches even if in this age the secondary headaches increase more than other pediatric ages. This study aims at revealing and verifying the relationship between the presence of red flags and neuroimaging abnormalities in prescholar headaches**.**

**Methods:** we have collected clinical data of 281 children (121 males and 160 females) aged from 1 to 7 years old admitted in emergency department (24,3% of total access for headache) from October 2015 to December 2017. We used a predetermined list of red flags (acute onset, associated symptoms, abnormal neurologic examination, resistance to analgesic therapy and others) and evaluate the number of children underwent computed tomography (CT).

**Results**: we found that 154/281 (54,8%) showing one or more red flags at the access to hospital (frequently nausea and vomiting), 97/154 (59,1%) of these was investigated with CT. Eighty-four of them showed positive CT for incidental benign abnormalities (ptosis of cerebellar tonsils, sinusitis, arachnoid cysts or non-specific abnormalities) ten of them showed negative CT, while just three patients (1,06%), with more than one red flags at the access, showed altered CT for dangerous anomalies.

**Conclusions:** Our study shows that prescholar headaches are not infrequent in Emergency department and several children (almost 50%) present one or more red flags. However the sensitivity of red flags remains low in unraveling dangerous neuroimaging abnormalities. In the setting of a normal neurologic examination or in presence of aspecific red flags neuroimaging can be deferred in most pediatric patients.

**REFERENCES**

1.Roser T, Bonfert M, Ebinger F, Blankenburg M, Ertl-Wagner B, Heinen F. Primary versus secondary headache in children: a frequent diagnostic challenge in clinical routine. Neuropediatrics. 2013,44:34-9

**Table 1 (abstract P203).** More significant anomalies

Ptosis of cerebellar tonsil	Sinusitis	Arachnoid cysts	Expansion of liquor spaces	Cerebrovascular anomalies	Pineal calcifications	Intracranial tumors	Haemorrhage	Negative TC
42	10	3	18	13	11	2	1	10

### P204 Tecnostress and chronic headaches for the use of drugs: wash-out combined with enzymatic add-on therapy

#### Ennio Pucci^1^, Silvano Cristina^1^, Marcello Imbriani^3^, Giuseppe Taino^4^

##### ^1^Headache Science Center – IRCCS Mondino Foundation, Pavia, Italy; ^2^Department of Brain and Behavioral Sciences, University of Pavia, Pavia, Italy; ^3^Department of Public Health, Experimental and Forensic Medicine - University of Pavia, Pavia, Italy; ^4^U.O. Hospital Occupational Medicine - IRCCS "S. Maugeri" Foundation, Pavia, Italy

###### **Correspondence:** Ennio Pucci (ennio.pucci@mondino.it)

**Introduction**: Work and the working environment have a determining function in the development of symptoms which, in turn, can determine the onset of some headache as well as increase the frequency and / or intensity of pre-existing forms . Techno-stress is counted among the new occupational diseases according to the 2007 Guariniello ruling. This term includes various addictions: videodependence, internet addiction disorder, social network mania, information overload, multitasking, cybersex addiction, email addiction. Coined by the psychologist Graig Broad, it manifests itself with numerous symptoms: headache, hypertension, anxiety, panic attacks, decreased concentration, gastrointestinal disorders and cardiovascular diseases, depression, loss of desire up to behavioral changes and relational isolation. Always approached to managers, it is now also popular among workers of other risk categories such as call center operators, accountants, networkers, journalists, advertisers and financial analysts.

Nomophobia is the uncontrolled fear of being disconnected from the mobile phone network.

Headache, on the other hand, is one of the most common symptoms found in medical practice, frequent in individuals of working age, is a common cause of absence from work and reduced production yield and as we have seen is described together with psychological distress in all situations above described.To establish what role the work has in the onset of the headache and what consequences it has on work efficiency, is a reason for growing interest in scientific research.A headache is defined as chronic when the patient suffers for 15 days / month for 3 months. Patients often suffer from headache every day. Subjects with chronic headache do not constitute a homogeneous group with regard to type and intensity of symptoms. Furthermore, the diagnosis escapes the criteria proposed by the ICHD-III beta-version because it presents with intermediate symptoms in different forms. The abuse of analgesics in chronic headache always becomes a contributing factor and proper therapy requires an initial detoxification period. Therapy can be pharmacological and not pharmacological. In this case the cyto-enzymatic complementary therapy for the polypharmacy patient wash-out strategies is taken into consideration.

The Project consider an enzymatic nutritional supplements and drugs withdrawal. The problem of the wash out of previous therapies represents a potential obstacle to the establishment of rational therapeutic strategies in chronic headache patients: with the help of new biodynamic preparations, which can favorably alter the "enzyme soil", it is considered possible set up rational therapeutic strategies.

### P205 Atypical facial pain after a nasal trauma

#### M. Puma^1^, B. Petolicchio^1,2^, A. Viganò^1^, V. Di Piero^1,2^

##### ^1^Department of Human Neuroscience, Sapienza University of Rome, 00185 - Rome, Italy; ^2^“Enzo Borzomati” Pain Medicine Unit – University Hospital Policlinico Umberto I, 00161-Rome, Italy

###### **Correspondence:** M. Puma (martapuma@hotmail.it)

**Background**

External nasal pain is a difficult and misdiagnosed condition, often disabling and refractory to the antalgic treatment. The reason is the complexnasal sensory innervations. It may have various etiologies, both local (e.g.infectious, neoplastic or traumatic rhinopaty) and central, such us trigeminal neuralgia, nasociliary neuralgia, nasal migraine and idiopathic rhinalgia. In literature have been described few cases of post traumatic nasal pain syndrome[1,2], that is nasal pain occurring immediately after a trauma to the nasal pyramid which persists through time. We report the case of a young woman suffering from a nasal pain that occurred one month later after a facial trauma.

**Case Report**

Our patient is a 34 year-old woman with a history of episodic migraine without aura who was injured striking her face against the corner of a closet. A month after the trauma, she developed pain to the right *radix nasi*and medial omolateral periorbital region. The pain was daily, persistent and continuous, severe in intensity and stabbing/burning. No associated autonomic symptoms were reported andour patient’s pain did not meet the criteria for nasociliary neuralgia. The pain referred involved the nasal skin portion supplied by infratrochlear and anteriorethmoidal nerves, therefore the painful area did not show the typical topography of a non traumatic neuralgia affecting one of the terminal branches of the trigeminal nerve [3,4]. Neurological examination and MRI were normal. This pain was resistant to common analgesic medications (NSAID, paracetamol) and showed only a partial and unsatisfactory improvement from pregabalin and amitriptyline, which are effective in treatment of neuropathic pain. Instead, it seems to have had a satisfactory response to topical corticosteroid (intranasal fluticasone spray): at 6 months follow-up the patient referred symptom resolution.

**Conclusions**

In this case, the failure of general analgesic therapy suggests a sort of post-traumatic neuropathic pain. However, modulating therapy alone (pregabalin plus amitripriline) was not sufficient to provide a complete relief.Interestingly, the addition of a topical corticosteroid was effective, so suggesting that a combination of drugs that act on various aspects of pain, neuropathic processes as well as inflammatory response, could be a valuable therapeutic strategy in post-traumatic nasal pain.

**References**

1. Rozen T. Post‐Traumatic External Nasal Pain Syndrome (a Trigeminal Based Pain Disorder). Headache. 2009, 49(8): 1223-1228

2. Golding-Wood D.G., Brookes G.B. Post-traumatic external nasal neuralgia--an often missed cause of facial pain?Postgrad Med J. 1991,67(783): 55-56

3. García‐Moreno H, et al. External nasal neuralgia: A neuropathic pain within theterritory of the external nasal nerve.Headache. 2015,55(9): 1259-1262

4. Pareja J.A., Pareja J, Yangüela J. Nummular headache, trochleitis, supraorbital neuralgia, and other epicranial headaches and neuralgias: the epicranias.J Headache Pain. 2003, 4(3): 125-131

**Consent for publication**: A written informed consent was obtained from the patient.

### P206 Prophylactic effect of ultramicronized N-Palmitoyl Ethanol Amide (PEA) on pediatric migraine: a preliminary study

#### Giorgia Sforza^1,2^, Laura Papetti^1^, Samuela Tarantino^1^, Barbara Battan^1^, Michela Ada Noris Ferilli^1,3^, Romina Moavero^1,2^, Federico Vigevano^1^, Massimiliano Valeriani^1^

##### ^1^Headache Center, Child Neurology Unit, Bambino Gesu’ Children’s Hospital, Rome, Italy; ^2^Child Neurology and Psychiatry Unit, Tor Vergata University of Rome, Italy; ^3^Institute of Neurology, Catholic University of Sacred Heart, Rome, Italy

###### **Correspondence:** Giorgia Sforza (sforzagiorgia@gmail.com)

**Background:**

Primary headache disorders are recognized as one of the most prevalent health problems worldwide. The prevalence of headaches during childhood have been investigated across pediatric age groups with varying estimates ranging from 3 % in school-age children to 20 % in adolescents [1,2].

Traditionally, pediatric migraine treatment includes both prophylactic therapy, aimed at reducing the severity and frequency of attacks, and acute therapy to stop the attack.

Although amitriptyline, topiramate, flunarizine and valproic acid have the most data on their use for prophylaxis in children, a serious lack of controlled studies on the pharmacological treatment of pediatric migraine still remains. Consequently, there is an urgent need for further studies in this population [3,4].

Nutraceuticals, and other supplements, may be an alternative option in treating migraine and may be offered to parents who are reluctant to start their child on a daily medication.

Palmitoyl ethanolamide (PEA) is an amide of endogenous fatty acids widely distributed in different tissues, including nervous tissues. It is emerging as a new therapeutic approach in pain and inflammatory conditions and has been reported as effective in animal models of chronic pain and inflammation, as well as in numerous clinical studies on various paininful diseases [5,6,7]. However, to date no studies have been conducted to evaluate the role of PEA in the management of migraine without aura in pediatric patients.

**OBJECTIVE:**

The aim of this preliminary open-label study was to evaluate the efficacy of chronic ultramicronized PEA (um-PEA) administration in terms of reducing the frequency and severity of migraine attacks in pediatric patients.

**METHODS:**

The study sample includes 5 patients (1 male and 4 females), ranging between 6,7 and 12,1 years of age (mean 9.4 years). They had a diagnosis of migraine according to the ICHD-3 criteria and received umPEA (600 mg/day). They were re-evaluated at 60 days after treatment onset.

**RESULTS:**

After 60 days of treatment with um-PEA, headache frequency was reduced by >50 % per month in 4 out of 5 patients and pain intensity was reduced from moderate to mild in 4 out of 5 patients.

**CONCLUSIONS:**

Our preliminary data show that um-PEA administered chronically for 60 days reduces pain intensity and the number of attacks per month in a small sample of pediatric patients. Although the small number of patients does not allow us to consider these initial results as definitely reliable, they encourage us to expand the sample.

**References:**

1) World Health Organization, Lifting the Burden. Atlas of Headache Disorders and Resources in the World 2011. A collaborative project of World Health Organization and Lifting the Burden.

Geneva: World Health Organization; 2011

2) Lewis D. Pediatric migraine. Neurol Clin. 2009;27:481–501.

3) Lewis DW, Diamond S, Scott D, Jones V. Prophylactic treatment of pediatric migraine. Headache. 2004;44(3):230–7.

4) Papetti L, Spalice A, Nicita F, Paolino MC, Castaldo R, Iannetti P, Villa MP, Parisi P. Migraine treatment in the developmental age: guidelines update. J Headache Pain. 2010;11(3):267–76.

5) Costa, B., Comelli, F., Bettoni, I., Colleoni, M., Giagnoni, G., 2008. The endogenous fatty acid amide, palmitoylethanolamide, has anti-allodynic and anti-hyperalgesic effects in a murine model of neuropathic pain: involvement of CB(1), TRPV1 and PPARgamma receptors and neurotrophic factors. Pain 139, 541-550.

6) Chirchiglia, D., Della Torre, A., Signorelli, F., Volpentesta, G., Guzzi, G., Stroscio, C.A., Deodato, F., Gabriele, D., Lavano, A., 2016. Administration of palmitoylethanolamide in combination with topiramate in the preventive treatment of nummular headache. International medical case reports journal 9, 193-195.

7) Gabrielsson, L., Mattsson, S., Fowler, C.J., 2016. Palmitoylethanolamide for the treatment of pain: pharmacokinetics, safety and efficacy. British journal of clinical pharmacology 82, 932-942.

### P207 The attention to social and health problems of headaches by central and peripheral structures of social and health services

#### Gregorio Iannone^1^, Carlo Piccolini^2^

##### ^1^Deputy coordinator S.I.S.C. Umbria - Marche – Abruzzo Already responsible for the center - headaches company hospital "Santa Maria" Terni; ^2^A.O”S.Maria” Neuroscience and neurorehabilitation Department Terni Italy

###### **Correspondence:** Gregorio Iannone (iannonegregorio45@gmail.com)

The author reviews the evolution of the concept of social disease with regard to headache and migraine, in particular to chronic forms. It examines the European directives and their implementation by the Italian governments, especially the regional ones that have the best skills in the field. They carry out evaluations on the need for new frameworks for all types of pain and recognition in the categories of disability.

### P208 The Neurologist of Territory and headache: critical issues and perspectives

#### Domenico Cassano (info@domenicocassano.it)

##### Headache Centre, ASL SA, Campania, Distretto 60, Via S. Giordano, 7, 84014 Nocera Inf. (SA)

Headaches represent about 25% of medical consults in a Territorial neurological office, in front of 10% of non-traumatic headaches referring to an Emergency Department. Aim of this presentation is to emphasize the central role of Territorial neurologist in headache management, the potential resources, critical issues and future perspectives.

Many are efforts performed in this field by AINAT, the national association of Territorial neurologists, founded about 20 years ago, tending to manage and to improve quality of life of headache patients: from a constant updating for the “headache expert neurologist” to many other initiatives (meetings, dedicated centres, etc).

Main goal is the organization of a combined and multidisciplinary headache network, for specific areas, in order to ensure continuity in the different levels of care.

### P209 Headaches, TTH and Migraine

#### G.Andrisani^1^, G. Andrisani^2^

##### ^1^Studio Dentistico Andrisani, Matera, Italy; ^2^Tandzorg Delft Centrum, Tandarts Praktijk, Delft, Netherland

Cephalgic patients Have Cerebral Cortex (CC) hyperexcitability (CH) due at the high number of unspecific synapses. In the TTH, after an injury or damage in the cervical cranial district that does not resolve spontaneously and in the right time frame, an increase in excitability is observed in the Caudal Nucleus of Trigeminal (NCT) neurons. Among the various causes, particularly important is sleep bruxism which, by activating theTrigeminal mesencephalic nucleus, activates ARAS nuclei and, therefore, the entire CC. The resulting cortical hyperactivity is due to the activation of a large number of glutamatergic synapses per neuron. This results in a large influx of Na+ and Ca++ ions in their cytoplasm and a large outflow of K+, H+ and ATP that is able, physiologically, to activate, the meningeal perivasal nerve endings, coming from the ganglion of Gasser (GSG), that bring the algic information to the NCT. We have central sensitization and resulting hyperalgesia. In the NCT we find c-fos. Moreover, the efferent branches of GSG, are able to conduct the signal in an antidromic way and to release CGRP, SP and NGF that are powerful inflammatory agents that induce mast cell degranulation, release of bradykinin and NO that generates vasodilation which causes the classic pulsating pain of TTHs. In the migraine with aura (MA), because of the aforementioned CH, at some point somewhere, in the CC the UA becomes so high as to generate a cortical decoding of the nerve transmission, nonspecific, non-sense in that part of the CC. The "non sense" encodings activate protective mechanisms that block the neuronal transmission blocking the UA and which manifests itself with a depressive alteration of the EEG tracing, the Cortical Spreading Depression (CSD). The VLPO / MnPO GABA provokes a temporary functional block of the ARAS nuclei, causing a functional block of nonspecific synapses of cortical neurons; as a computer reset, but the excess of K + in the intercellular matrix can activate the Microglia (Natural Immunity, or Innate) cause, at the cortical level, as in the TTH, vasoconstriction / vasodilation / extravasation ... .. pai: the MA. If the microglial reaction does not activate we will have the Aura but not the pain. .

Migraine without Aura (MO) are essentially menstrual migraine and even in this case they do not differ much from the previous ones because they respond well to the same therapies as MA (NSAIDs, Triptans etc.), which means that the basic mechanism is same: vasoconstriction, reactive vasodilation and extravasation, CGRP etc. In these patients, the menstruation, this must be considered, to all intents and purposes, a large wound, with a large production of Prostaglandins and varius inflammation mediators (hence the pain in the lower abdomen). For some reason, endogenous or exogenous, at the time of menstruation estrogen and / or progesterone levels are not low, The "uterine wound", with large amounts of Prostaglandins (and not only) and high levels of estrogen and Progesterone, all lead to CH and headache (Migraine or TTH) with the mechanisms desc previously ruled.

### P210 Hopelessness in Migraine: a new psychological marker for disease evolution and response to treatment?

#### R. Lo Baido^1^, M. S. Epifanio^2^, N. Bellavia^2^, M. Piombo^2^, A. Torrente^3^, B. Fierro^3^, S. La Grutta^2^, F. Brighina^3^

##### ^1^Dipartimento di Biopatologia e Biotecnologie Mediche (DiBiMed); ^2^Dipartimento di Scienze Psicologiche, Pedagogiche e della Formazione (SPPF); ^3^Dipartimento di Biomedicina sperimentale e neuroscienze cliniche (BioNeC)

*Objectives*

Migraine is a common disabling primary headache disorder, afflicting in average 12-15 percent of the global population. The relationship among psychological aspects in migraine, has received increasing interests in literature [1]. The aim of the present study is to evaluate the correlation between emotional regulation, psychological markers of depression, anxiety and migraine, in order to determine the impact on the prognosis and quality of life.

*Subject and methods*

80 (67 F) consecutive outpatients affected by episodic (EM) or chronic migraine (CM) [2], admitted for the first visit at the ‘Headache Center’ at Policlinico ‘Paolo Giaccone’ in Palermo, between October 2017 and June 2018, were enrolled in this study. Participation was obtained through a written informed consent.

All patients underwent clinical neurological examination and test batteries exploring specific psychological aspects and disease-related disability. Alexithymia was measured using the Toronto Alexithymia Scale (TAS-20), a measure of deficiency in understanding, processing and describing emotions. The Beck Hopelessness Scale (BHS), was used mainly to evaluate three major aspects of hopelessness, which is a factor of the depressive condition: feelings about the future, loss of motivation and expectations. Anxiety was measured using the Beck Anxiety Inventory (BAI). Patients underwent also SF-12, a multipurpose short-form generic measure of health status; Brief Pain Inventory (BPI), a 9 item self-administered questionnaire to evaluate pain severity and impact on daily functioning; Headache Impact Test (HIT-6) to measure headache impact on daily functioning (at job, school, home and social situations).

*Results*

No significant differences emerged between EM and CM patients as concerns demographic features. Alexithymia measures showed no significant changes between EM and CM. Differently, CM patients presented significantly higher scores, as compared to EM group, in measures concerning the construct of hopelessness.

*Discussion and conclusions*

The study provides important informations about the relationship between migraine and more specific depression factors, like hopelessness, particularly in CM patients. Further studies in greater patients series and with prospective design will help better defining the role of such factor as prognostic marker for disease evolution and response to treatments (risks of non-compliance).

*References*

1. de Andrade-Vieira RV, Vieira DC, Barbosa Gomes W, Gauer G(2013) Alexithymia and its impact on quality of life in a group of Brazilian women with migraine without aura*, J Headache Pain,* 14:18.

2. Headache Classification Committee of the International Headache Society. The international classification of headache disorders, 3^rd^ edition (beta version)(2013). *Cephalalgia* 33(9):629–808.

### P211 Selected cannabinoids and cutaneous allodynia in chronic refractory migraine

#### Maria Nicolodi^1^, Maria Stella Pinnarò^1 2^, Vanessa Sandoval^1^

##### ^1^Foundation Prevention and Therapy Primary Pain and Headache, Florence 50125, Italy; ^2^Department Neuroscience, University of Florence,Florence 50139 Florence, Italy

###### **Correspondence:** Maria Nicolodi (info@fondazionesicuterinicolodi.it)

**Background** Allodynia is a feature of central neurogenic-neuropathic pain. Cannabis psychoactive constituent Δ9-tetrahydrocannabinol (THC) has efficacy against neurogenic chronic pain; however, its effect is hampered by side effects. Co-administration with cannabidiol (CBD) might enhance the analgesic actions while limiting side-effects. Here an observation with the end-point of testing the anti-allodynic effect of the therapy.

**Procedure** A 15-days treatment with 400 mg /day/oral 6%THC-7.5%CBD has been administered to 68 chronic migraine sufferers (33 males, 35 female, mean age 38.2±11.9SD), refractory to conventional therapies and ailed by unbearable allodynia state. Inclusion criteria: Absence of systemic or psychiatric diseases, no addiction to recreational drugs. We previously indicated 200 mg cannabis flos 22% THC/orally as a therapeutic dose [1]. Here, we used 400mg R/cannabis flos 6%THC-7.5%CBD.

Control Group: 55 age-sex matched volunteers showing overlapping number of migraine hours/monthly, defined refractory to conventional therapies in other centres. Controls choose to re-test amitriptyline 50 mg/day/orally in a 15 days treatment. No patient used acute migraine treatments during the 3 days before allodynia evaluation and test-day. Allodynia measurements: a) brushing VAS 0-10, b) 12 items allodynia checklist [2]. Allodynia evaluations: after 2 weeks run-in; following treatment, 3 hours after drug intake. Observation in agreement with Helsinki Declaration (FE 013/17).

**Results** Allodynia: 6%THC-7.5%CBD fixed combination induceed 20 fold higher anti-allodynic effect versus amitriptyline short treatment: THC-CBD run-in value 16.5±4.6 SD versus post-treatment 8.1±4.9SD p>0.00001, amitriptyline run–in value versus post-treatment values: 16.1±4.6 SD versus 12.07±4.02SD, p>0.002. Brush Test: mean VAS (0-10) temples/forehead 8.4±1.2SD after run–in, 2.2±1.6SD after THC-CBD, 6.8±3.4SD after amitriptyline p>0.00001.

Side Effects of 6%THC-7.5%CBD: Somnolence: n=1, amitriptyline group n=2, withdrawal at discontinuation n=0.

**Conclusion**

Bediol-induced anti-allodynic effect was higher then amitriptyline-induced effect in checklist (50% decrease) and in brushing tests of cephalic area (75%). The effect was not associated with clear cannabinoid side-effects. Higher doses of THC might induce greater anti-allodynic effect.

**References**

1.Nicolodi M, Sandoval V. Therapeutic use of cannabinoids - Dose finding, pilot data of effects in chronic migraine and cluster headache. Proceedings EAN Congress, 24-27 June 2017.

2. Lipton RB, Bigal ME, Ashina S, Burstein R, Silberstein S, Reed ML, Serrano D, Stewart WF. Cutaneous allodynia in the migraine population. Ann Neurol. 2008 Feb;63(2):148-58.

### P212 Follow-up study of 17-25 years on subtypes of headache in a population children below 6 years old

#### Lucia Rocchitelli^1^, Francesca Marchese^1^, Marco Micale^1^, Carmela Loiacono^1^, Francesca Vanadia^2^, Filippo Brighina^3^, Salvatore Mangano^1^, Vincenzo Raieli^2^

##### ^1^Child Neuropsichiatry Department - University of Palermo; ^2^Child Neuropsychiatr Unit, ISMEP - ARNAS Civico Palermo; ^3^Department of Experimental Biomedicine and Clinical Neurosciences - University of Palermo

###### **Correspondence:** Lucia Rocchitelli (luciana.91@hotmail.it)

**Background**: Although primary headaches are common neurological disorders in pediatric population, they have received poor attention in children under the age of 6 years [1]. Some recent studies have shown the increasing prevalence of primary headaches in this age. The prospective follow-up researches are very rare, both in relation to the temporal course of migraine in school-age children and adolescents and to its clinical course in adulthood. Furthermore the samples are small and their studied follow up periods are brief. For these reasons we have conducted a follow-up study on our cephalalgic population considering only the preschool age [2].

**Methods**: We have conducted an interview on our previously studied preschooler cephalalgic population [2]. The survey included 13 questions. The secondary headaches were excluded from the sample. The ethical committee approved the research project and informed consent was firmed.

**Results**: Our sample counted 23 children (11 males, 12 females, mean age 21.2, range 17-26). Follow-up was mean age 17,4 (range 15,5-21,1 ys) , 16/23 (69.5%) patients still suffer from headache. At first admission 17 migrainous children (13 continued to suffer from migrainous attacks at follow-up); 3 primary stabbing headaches (in 2 children the preexisting headaches were transformed into migraine); 3 tension headache type (2 in remission and one transformed into migraine). Finally we observed the presence of allodynia in 56% (9/16) and of cranial autonomic symptoms 38% (6/16) in persistent migraineurs.

**Discussion**: There are very few and short follow-up studies of preschooler headaches [3]. Overall, migraineurs had poorer prognosis than other primary headaches and a negative predictive factor for the persistence of headache was vomiting during the attacks. Our study is the first with more than ten years follow-up in pediatric population and it confirms the poorer prognosis of preschooler migraine. The study confirms more prevalence of allodynia in follow-up of prescholar migraine headaches [4] suggesting that the presence of allodynia during the painful attacks may be a negative predictive factors for the persistence of migraine. Our sample has the limit to be small and with high escape rate to follow-up (about 70%), however the escape rate is lower in migrainous sample (46%), similarly to other study [3].

Surely other studies with a wider sample and a longer follow up are needed, possibly including a prospective design and a multifactorial analysis with the aim of an early and more appropriate management in order to avoid chronic headaches.

**References**

9) Abu-Arafeh I, Howells R. Primary headaches in children under the age of 7 years. Curr Pain Headache Rep. 2014; 18:401-8

10) Raieli V, Eliseo M, Pandolfi E, La Vecchia M, La Franca G, Puma D, et al. Recurrent and chronic headaches in children below 6 years of age. J Headache Pain. 2005;6:135-42

11) Virtanen R, Aromaa M, Rautava P, Metsähonkala L, Anttila P, Helenius H, et al. Changing headache from preschool age to puberty. A controlled study. Cephalalgia. 2007 27:294-303

12) Raieli V, Trapolino D, Giordano G, Spitaleri C, Consolo F, Santangelo G, Buffa ,D, Vanadia F, D'Amelio M. Juvenile migraine and allodynia: results of a retrospective study. Headache. 2015 Mar;55(3):413-8.

### P213 Optimization of Outpatient Appointment Scheduling in Cephalalgic Day Service

#### Rosario Iannacchero^1^, Domenico Conforti^2^, Giuseppe Ielpa^3^, Maria Carmela Groccia^2^, Rosita Guido^2^

##### ^1^Regional Headache Centre, Neurology Division, "Pugliese-Ciaccio" Hospital, Catanzaro, Italy; ^2^Department of Mechanical Energy Management Engineering, University of Calabria, Rende, Italy;^3^Department of Mathematics and Computer Science, University of Calabria, Rende, Italy

###### **Correspondence:** Rosario Iannacchero (centrocefaleeaopc@gmail.com)

**Introduction.** Effective resource utilization represents a strategic objective of every health care system. Day Service is an operational model, to deliver diagnostic tests and therapeutic treatments,with a prioritization, that can tackle the hinder of long waitings, minimize the risk of diagnosis uncertainty, and avoid inappropriate hospitalization, or limit patient access to the Emergency Department. This model is well suited for cephalalgic patients, who do not require hospitalization, but are often prone to inappropriate diagnosis and long waits for clinical service delivery. This work focuses on a solution for Cephalalgic Patient Appointment Scheduling, based on mathematical model, to manage outpatient Day Service.

**Materials and Methods.** Day Service is an organizational model based on one-day admission, suitable for tailoring clinical services on patients needs. This model applies well to clinical domains where multidisciplinary diagnostic tests and therapies are needed. Clinical service delivery is planned in advance, after a baseline visit, and patients are queued and scheduled in function of a priority, based on their severity level. A diagnosis should be devised in a short period of time (tipically one month). We start from previous works related to Day Service in Cephalalgic Networks [1] and other clinical sectors [2] operations management, we tailor a model of the Patient Appointment Scheduling problem, with the aim to possibly schedule all patients prescribed exams and tests, taking into account of the capacity of the health provider to deliver the clinical services, patient priority, service level agreements. the model is implemented in a declarative flavour, with the help of Answer Set Programming, an innovative approach emerging from the Artificial Intelligence sector.

**Results**. Patient Appointment Scheduling has been validated within the Regional Headache Centre, Neurology Division, "Pugliese-Ciaccio" Hospital, Catanzaro, Italy, over 500 prescriptions of clinical service delivered for 220 patients.

**Conclusions**. Day Service represents an innovative and efficient health care model, an Appointment scheduling systems represent a method to manage patient's waiting lists, and it may be adopted in many health care systems. Answer Set Programming is suitable to flexibly prototype service operations requirements.

**References**

1. Conforti, D., Groccia, M.C., Corasaniti, B., Guido, R., and Iannacchero, R.: “Calabria cephalalgic network”: innovative services and systems for the integrated clinical management of headache patients”, EHMTI-0172, The Journal of Headache and Pain 15 (1), 1-1, 2014.

2. Ielpa, G., Guido R., Conforti D.: Outpatient Day Service Operations: a Case Study within Rheumatology Diseases Management. Proceedings of the International Conference on Health Care Systems Engineering, HCSE 2017, Florence, Italy. Springer (2018):269-279.

- **References**

1. Rozen T. Post‐Traumatic External Nasal Pain Syndrome (a Trigeminal Based Pain Disorder). Headache. 2009, 49(8): 1223-1228

2. Golding-Wood D.G., Brookes G.B. Post-traumatic external nasal neuralgia--an often missed cause of facial pain? Postgrad Med J. 1991, 67(783): 55-56

3. García‐Moreno H, et al. External nasal neuralgia: A neuropathic pain within the territory of the external nasal nerve. Headache. 2015, 55(9): 1259-1262

4. Pareja J.A., Pareja J, Yangüela J. Nummular headache, trochleitis, supraorbital neuralgia, and other epicranial headaches and neuralgias: the epicranias. J Headache Pain. 2003, 4(3): 125- 131

