# Correction to: 12th European Headache Federation Congress jointly with 32nd National Congress of the Italian Society for the Study of Headaches

**DOI:** 10.1186/s10194-018-0947-y

**Published:** 2018-12-06

## Correction

After publication of this supplement [1], it was brought to our attention that e-mail errors were apparent in the following abstracts. This has now been included in this correction.

ᅟ

**O24**

**The economic and humanistic burden of episodic and chronic migraine in Europe**

ᅟ

Hicham Benhaddi^1*^, Timothy Fitzgerald^2^, SophieMcCabe^3^, Ruth Zeidman^3^

^1^ Teva Pharmaceuticals, Wilrijk, Belgium

^2^ Teva Pharmaceuticals, Frazer, Pennsylvania, USA

^3^ Covance Market Access, London, UK

* Correspondence: Hicham Benhaddi (Hicham.Benhaddi@tevaeu.com).

ᅟ

**The correspondence e-mail address contained an error. The correct e-mail address has now been included in this correction.**

ᅟ

**BACKGROUND:** Migraine is a debilitating neurological disorder characterized by attacks that may last 4–72 hours, with a high burden in Europeans.

**OBJECTIVE:** This systematic literature review examined the clinical, humanistic, and economic burden associated with chronic and episodic migraine (CM and EM, respectively) in Europe.

**METHODS:** Literature searches and evidence screening were structured according to the PICOS (population, intervention, comparators, outcomes, and study types) framework. Reviews and original observational studies in adults (≥18 years) with EM (<15 headache days per month) or CM (≥15 headache days with ≥8 migraine days per month) were included. Searches focused on resource utilization, treatment costs, productivity, quality of life (including generic and migraine-specific instruments and functioning), and utility outcomes (published 2007–February 1, 2018; geographical limitation: United Kingdom, France, Germany, Spain, Italy, the Netherlands, Poland, Denmark, Finland, Iceland, Norway and Sweden). Searches included: Embase, MEDLINE, and the Cochrane Library databases; specialty medicine associations; and health technology assessment agency websites.

**RESULTS:** Analysis included 68 publications. Data from the World Health Organization indicated that in Europe, migraine burden weighed higher than that of epilepsy, multiple sclerosis, and Parkinson’s disease. Up to 57% of individuals with migraine report severe disability, and many find treatments ineffective. Nausea and/or vomiting occurred in up to 74% of individuals with migraine. Depression and/or anxiety occur up to three times more often in individuals with migraine than in the general population. People with migraine have reported poorer health-related quality of life than those without migraine, which worsens with increasing migraine attack frequency. Europeans with migraine perceive that it has a negative impact on work (up to 76%), family situations, leisure time, studies, sexual life, social position, love, financial situation, career, and friendships. The prevalence of migraine is highest for men and women during their peak years of economic productivity (ages 25–55 years). In Europe, the estimated total annual cost was up to €111 billion (2008–2009), of which 72%–98% was indirect costs (two-thirds of indirect costs due to reduced productivity). Annual direct costs for CM were up to four times higher than those for EM.

**CONCLUSIONS:** This research demonstrates that migraine has a substantial humanistic and economic burden on Europeans and affects all aspects of life.

ᅟ

**O38**

**Impact of fremanezumab on response rates, acute medication use, and disability in patients with episodic migraine who have failed at least one prior migraine preventive medication**

Paul K. Winner^1*^, Rashmi B. Halker Singh^2^, Joshua M. Cohen^3^, Ronghua Yang^3^, Paul P. Yeung^3^, Verena Ramirez Campos^4^

1 Premiere Research Institute, West Palm Beach, Florida, USA

2 Mayo Clinic, Phoenix, Arizona, USA

3 Teva Pharmaceuticals, Frazer, Pennsylvania, USA

4 Teva Pharmaceuticals, Buenos Aires, Argentina

* Correspondence: Paul K. Winner (pwinner777@aol.com).

ᅟ

**The correspondence e-mail address contained an error. The correct e-mail address has now been included in this correction.**

ᅟ

**BACKGROUND:** Preventive medication is recommended for episodic migraine (EM) patients with ≥4 headache days per month. Fremanezumab, a fully humanized monoclonal antibody (IgG2a) that selectively targets calcitonin gene-related peptide (CGRP), is efficacious in preventing EM, but its effectiveness in patients who failed previous preventive medications is unknown.

**OBJECTIVE:** To assess the effects of fremanezumab on response rates, acute headache medication use, and disability in EM patients who failed at least one prior preventive migraine medication.

**METHODS:**

In this Phase 3, multicenter, randomized, double-blind, placebocontrolled study, patients were randomized 1:1:1 to receive subcutaneous injections of fremanezumab quarterly (675 mg at baseline and placebo at Weeks 4 and 8), fremanezumab monthly (225 mg at baseline, Weeks 4 and 8), or placebo (at baseline, Weeks 4 and 8) over a 12-week treatment period. Analyses were performed in patients who failed at least one prior preventive migraine medication (due to lack of efficacy or intolerability) using Cochran–Mantel–Haenszel test or an analysis of covariance model. Endpoints included the proportion of patients with ≥50% reduction in the monthly average number of migraine days, mean change from baseline in the monthly average number of days of acute headache medication use, and mean change from baseline in the Migraine Disability Assessment (MIDAS) score during the 12-week treatment period.

**RESULTS:** The subgroup who failed at least one prior migraine preventive therapy included 58 fremanezumab quarterly, 65 fremanezumab monthly, and 63 placebo patients. A greater proportion of patients who received fremanezumab had a ≥50% reduction in the monthly average number of migraine days during the treatment period (quarterly: 38%, *P*=0.0100; monthly: 43%, *P*=0.0010) compared with placebo (17%). Fremanezumab significantly reduced from baseline the monthly average number of days of any acute headache medication use during the treatment period (quarterly [least-squares mean change ± standard error]: –3.1±0.5 days; monthly: –3.4±0.5 days) compared with placebo (–1.1±0.5 days; both, *P*<0.0001). Fremanezumab significantly improved disability from baseline, based on the change in MIDAS score during the treatment period (quarterly: –24.5±3.7, P=0.0006; monthly: –26.8±3.7, P<0.0001) compared with placebo (–11.1±3.4).

**CONCLUSIONS:** Among EM patients who failed at least one prior preventive migraine medication, fremanezumab treatment was efficacious, reduced acute headache medication use, and improved disability, with effect sizes greater than those seen in the overall trial population.

**TRIAL REGISTRATION:** ClinicalTrials.gov, NCT02629861

**ETHICS APPROVAL**

The study was approved by relevant independent ethics committees or institutional review boards, according to national or local regulations.

ᅟ

**O40**

**Efficacy of fremanezumab in migraine patients who have failed at least one prior migraine preventive medication**

ᅟ

Peter McAllister^1*^, David W. Dodick^2^, Joshua M. Cohen^3^, Ronghua Yang^3^, Paul P. Yeung^3^, Verena Ramirez Campos^4^

1 New England Institute for Neurology and Headache, Stamford, Connecticut, USA

2 Department of Neurology, Mayo Clinic, Phoenix, Arizona, USA

3 Teva Pharmaceuticals, Frazer, Pennsylvania, USA

4 Teva Pharmaceuticals, Buenos Aires, Argentina

* Correspondence: Peter McAllister (peter@neinh.com).

ᅟ

**The correspondence e-mail address contained an error. The correct e-mail address has now been included in this correction.**

ᅟ

**BACKGROUND:** Fremanezumab, a fully humanized monoclonal antibody (IgG2a) that selectively targets calcitonin gene-related peptide (CGRP), has been shown to be effective in the prevention of chronic migraine (CM) and episodic migraine (EM).

**OBJECTIVE:** To assess the efficacy of fremanezumab in migraine patients who failed at least one prior preventive migraine medication.

**METHODS:** Fremanezumab was studied in two Phase 3, multicenter, randomized, double-blind, placebo-controlled, parallel-group trials. Patients with CM or EM (confirmed during a 28-day pre-treatment baseline period) received subcutaneous injections of fremanezumab quarterly (675 mg at baseline and placebo at Weeks 4 and 8), monthly (CM: 675 mg at baseline and 225 mg at Weeks 4 and 8; EM: 225 mg at baseline and Weeks 4 and 8), or placebo (at baseline and Weeks 4 and 8) over a 12-week treatment period, with a final evaluation 4 weeks after the last dose of the study drug. Mean changes from baseline in the monthly average number of headache days of at least moderate severity or the monthly average number of migraine days during the 12-week treatment period were assessed in patients who failed at least one prior migraine preventive medication due to lack of efficacy or intolerability. Analyses were performed in the intent-to-treat population using an analysis of covariance (ANCOVA) model.

**RESULTS:** In CM patients, fremanezumab yielded greater reductions in the monthly average number of headache days of at least moderate severity (quarterly [n=130] [least-squares mean change ± standard error]: –4.0±0.47, *P*<0.0001; monthly [n=141]: –4.6±0.46, P<0.0001) compared with placebo (n=136; –1.9±0.49). There were similar reductions in the monthly average number of migraine days (quarterly: –4.2±0.55, *P*=0.005; monthly: –4.8±0.53, *P*<0.0001) compared with placebo (–2.4±0.56). In EM patients, fremanezumab yielded greater reductions in the monthly average number of headache days of at least moderate severity (quarterly [*n*=58]: –3.0±0.51, *P*<0.0001; monthly [n=65]: –3.2±0.49, *P*<0.0001) compared with placebo (*n*=63; –0.7±0.47). There were similar reductions in the monthly average number of migraine days (quarterly: –3.3±0.61, *P*=0.0015; monthly: –3.8±0.59, *P*<0.0001) compared with placebo (–1.3±0.57). *P*-values stated are compared with placebo.

**CONCLUSIONS:** Fremanezumab was efficacious in migraine patients who failed at least one prior migraine preventive medication, a potentially difficultto-treat population. Effect sizes in this subgroup were greater than those in the overall trial population.

**TRIAL REGISTRATION**: ClinicalTrials.gov NCT02621931 and NCT02629861

**ETHICS APPROVAL**

The study was approved by all relevant independent ethics committees or institutional review boards, according to national or local regulations.

ᅟ

**P3**

**The impact of fremanezumab on symptoms associated with migraine in patients with episodic migraine**

ᅟ

Jan L. Brandes^1*^, Paul P. Yeung^2^, Ernesto Aycardi^2^, Ronghua Yang^2^, Yuju Ma^2^, Joshua M. Cohen^2^

1 Nashville Neuroscience Group, Vanderbilt University, Department of Neurology, Nashville, Tennessee, USA

2 Teva Pharmaceuticals, Frazer, Pennsylvania, USA

* Correspondence: Jan L. Brandes (jbrandes1@msn.com)

**The correspondence e-mail address contained an error. The correct e-mail address has now been included in this correction.**

ᅟ

**OBJECTIVES:** Non–headache symptoms (nausea, vomiting, photophobia and phonophobia) are included in the International Classification of Headache Disorders, third edition (beta version) (ICHD-3 beta) criteria for migraine. Fremanezumab, a fully humanized monoclonal antibody (IgG2a) that selectively targets calcitonin gene-related peptide (CGRP), reduced the number of migraine days in EM patients. We assessed the effect of fremanezumab on nausea or vomiting, and photophobia and phonophobia in EM patients.

**METHODS:** In this multicenter, randomized, double-blind, placebocontrolled, Phase 3 study, patients with EM were randomized 1:1:1 to receive subcutaneous fremanezumab quarterly (675 mg at baseline, placebo at Weeks 4 and 8), fremanezumab monthly (225 mg at baseline, Weeks 4 and 8), or placebo over a 12-week treatment period. Exploratory endpoints included mean change from baseline in the monthly average number of days with nausea or vomiting, and days with photophobia and/or phonophobia during the 12-week period after the first dose of study drug. Analyses were performed in the full analysis set (all randomized patients who received ≥1 dose of study drug and had ≥10 days of post-baseline efficacy assessments on the primary endpoint). The data were analyzed using both the analysis of covariance approach, with baseline number of days with nausea or vomiting, or photophobia and phonophobia, and years since onset of migraines as covariates, and the Wilcoxon rank-sum test.

**RESULTS:** Fremanezumab treatment yielded greater reductions from baseline in the monthly number of days with nausea or vomiting during the 12-week treatment period (quarterly [least-squares mean ± standard error]: –1.9±0.19 days, *P*=0.0314; monthly: –2.1±0.19 days, *P*=0.0008) compared with placebo (–1.4±0.19 days). Reductions in nausea or vomiting were seen as early as Week 4 (quarterly: –1.7±0.21 days, P=0.0046; monthly: –1.9±0.21 days, *P*=0.0002) compared with placebo (–1.0±0.21 days). Fremanezumab treatment also yielded greater reductions from baseline in the number of days with photophobia and phonophobia during the 12-week treatment period (quarterly: –2.2±0.21 days, *P*=0.0038; monthly: –2.4±0.21 days, *P*=0.0001) compared with placebo (–1.5±0.21 days). Significant reductions in days with photophobia and phonophobia were seen as early as Week 4 (quarterly: –2.0±0.23 days, *P*=0.0003; monthly: –2.2±0.23 days, *P*<0.0001) compared with placebo (–1.0±0.23 days).

**CONCLUSIONS:** In patients with EM, fremanezumab treatment rapidly improved non–head pain symptoms associated with migraine, including nausea or vomiting, and photophobia and phonophobia.

**TRIAL REGISTRATION:** ClinicalTrials.gov, NCT02629861

**ETHICS APPROVAL**

The study was approved by all relevant independent ethics committees or institutional review boards, according to national or local regulations.

ᅟ

**P4**

**The impact of fremanezumab on symptoms associated with migraine in patients with chronic migraine**

ᅟ

Peter McAllister^1*^, Paul P. Yeung^2^, Ernesto Aycardi^2^, Ronghua Yang^2^, Yuju Ma^2^, Joshua M. Cohen^2^

1 New England Institute for Neurology and Headache, Stamford, Connecticut, USA

2 Teva Pharmaceuticals, Frazer, Pennsylvania, USA

* Correspondence: Peter McAllister (peter@neinh.com)

ᅟ

**The correspondence e-mail address contained an error. The correct e-mail address has now been included in this correction.**

ᅟ

**OBJECTIVES:** The International Classification of Headache Disorders, third edition (beta version) (ICHD-3 beta) criteria for migraine include nausea, vomiting, photophobia, and phonophobia symptoms. Fremanezumab, a fully humanized monoclonal antibody (IgG2a) that selectively targets calcitonin gene-related peptide (CGRP), reduced the frequency and severity of headaches in patients with chronic migraine (CM). We assessed the effect of fremanezumab versus placebo on nausea or vomiting, and photophobia and phonophobia, in patients with CM.

**METHODS:** In this multicenter, randomized, double-blind, placebocontrolled, Phase 3 study, patients with CM were randomized 1:1:1 to receive subcutaneous injections of fremanezumab quarterly (675 mg at baseline, placebo at Weeks 4 and 8), fremanezumab monthly (675 mg at baseline, 225 mg at Weeks 4 and 8), or placebo (at baseline, Weeks 4 and 8) over a 12-week treatment period. Exploratory endpoints included the mean change from baseline in the monthly average number of days with nausea or vomiting, and days with photophobia and phonophobia during the 12-week period after the first dose of study drug. Analyses were performed in the full analysis set (all randomized patients who received ≥1 dose of study drug and had ≥10 days of post-baseline efficacy assessments on the primary endpoint) using analysis of covariance (with baseline number of days with the symptom, and years since onset of migraines as covariates) and the Wilcoxon rank-sum test.

**RESULTS:** Fremanezumab treatment yielded greater reductions from baseline in the monthly number of days with nausea or vomiting during the 12-week treatment period (quarterly [least-squares mean ± standard error]: –3.3±0.29 days, *P*=0.0009; monthly: –3.2±0.28 days, *P*=0.0019) compared with placebo (–2.2±0.29 days). Significant reductions in nausea or vomiting were seen as early as Week 4 (quarterly: –3.2±0.30 days, *P*<0.0001; monthly: –2.9±0.29 days, P=0.0014) versus placebo (–1.9±0.29 days). Fremanezumab treatment also yielded greater reductions from baseline in the number of days with photophobia and phonophobia during the 12-week treatment period (quarterly: –3.5±0.32 days, *P*=0.0025; monthly: –3.7±0.32 days, *P*=0.0001) versus placebo (–2.4±0.32 days). Reductions in days with photophobia and phonophobia were seen as early as Week 4 (quarterly: –3.5±0.33 days, *P*<0.0001; monthly: –3.5±0.32 days, *P*<0.0001) versus placebo (–2.1±0.33 days).

**CONCLUSIONS:** Fremanezumab treatment rapidly improved non–head pain symptoms associated with migraine, including nausea or vomiting, and photophobia and phonophobia, in patients with CM.

**TRIAL REGISTRATION:** ClinicalTrials.gov, NCT02638103

**ETHICS APPROVAL**

The study was approved by all relevant independent ethics committees or institutional review boards, according to national or local regulations.

ᅟ

**P5**

**Long-term impact of fremanezumab on response rates, acute headache medication use, and disability in patients with episodic migraine: interim results of a 1-year study**

ᅟ

Jan L. Brandes^1*^, Paul P. Yeung^2^, Joshua M. Cohen^2^, Sanjay K. Gandhi^2^, Timothy Fitzgerald^2^, Ronghua Yang^2^, Yuju Ma^2^, Ernesto Aycardi^2^

1 Nashville Neuroscience Group, Nashville, Tennessee

2 Teva Pharmaceuticals, Frazer, Pennsylvania, USA

* Correspondence: Jan L. Brandes (jbrandes1@msn.com)

ᅟ

**The correspondence e-mail address contained an error. The correct e-mail address has now been included in this correction.**

ᅟ

**BACKGROUND:** Fremanezumab, a fully humanized monoclonal antibody (IgG2a) that selectively targets calcitonin gene-related peptide (CGRP), has demonstrated efficacy in preventing episodic migraine (EM) in 3-month studies; this analysis evaluates its long-term effects.

**OBJECTIVE:** To investigate the long-term effect of fremanezumab on response, acute headache medication and disability in adults with EM.

**METHODS:** This 52-week, multicenter, randomized, double-blind, parallel-group study evaluated the long-term safety, tolerability and efficacy of fremanezumab in adults with migraine; disability was assessed using the Migraine Disability Assessment (MIDAS). Most patients rolled over from a pivotal EM study, but some patients enrolled directly into this long-term study. Patients were assigned to one of two subcutaneous dose groups: (1) monthly dosing: 225 mg doses of fremanezumab every month, or (2) quarterly dosing: 675 mg doses of fremanezumab every 3 months. Percentage of patients achieving ≥50% reduction in monthly average number of migraine days, the mean change from baseline in the monthly number of days of use of any acute headache medications, and the mean change from baseline in MIDAS score were assessed for both doses.

**RESULTS:** This study enrolled 780 EM patients. The mean change in monthly number of migraine days from baseline to Month 1 was – 4.6 days for the monthly treatment group and –4.9 days for the quarterly group. The proportion of patients achieving ≥50% reduction in monthly average number of migraine days at Month 6 was 61% with monthly dosing, and 65% with quarterly dosing. The mean change in monthly number of days of use of any acute headache medications from baseline to Month 6 in patients with EM was –4.1 days in the monthly group and –4.3 days in the quarterly group. The change from baseline in the MIDAS disability score in patients with EM was similar in both treatment groups at Month 6; disability scores decreased by 27.1 and 27.3 at Month 6 in the monthly and quarterly treatment groups, respectively. For a subset of patients who completed the entire 12-month treatment period, data available at the cutoff date indicated that the response achieved at Month 6 was maintained throughout the treatment period.

**CONCLUSION:** Efficacy and disability data from this interim analysis indicated that the efficacy observed at Month 1 was maintained during the remainder of the study.

**TRIAL REGISTRATION:** ClinicalTrials.gov, NCT02638103

**ETHICS APPROVAL**

The study was approved by all relevant independent ethics committees or institutional review boards, according to national or local regulations.

ᅟ

**P6**

**Overview of fremanezumab pooled safety data from placebocontrolled phase 2 and 3 studies**

ᅟ

Stephen D. Silberstein^1*^, Nicola Faulhaber^2^, Xiaoping Ning^3^, Paul P. Yeung^3^, Jimmy Schiemann^3^, Ronghua Yang^3^, Yuju Ma^3^, Ernesto Aycardi^3^

1 Thomas Jefferson University, Philadelphia, Pennsylvania, USA

2 Teva Pharmaceuticals, Ulm, Germany; 3Teva Pharmaceuticals, Frazer, Pennsylvania, USA

* Correspondence: Stephen D. Silberstein (Stephen.Silberstein@jefferson.edu)

ᅟ

**The correspondence e-mail address contained an error. The correct e-mail address has now been included in this correction.**

ᅟ

**BACKGROUND:** Fremanezumab, a fully humanized monoclonal antibody (IgG2a) that selectively targets calcitonin gene-related peptide (CGRP), has been shown to be effective in the prevention of episodic migraine (EM) or chronic migraine (CM).

**OBJECTIVE:** To summarize the safety profile of fremanezumab based on all placebo-controlled studies in patients with migraine.

**METHODS**: Fremanezumab has been studied in four placebocontrolled studies in patients with migraine, including two Phase 2 and two Phase 3 studies. Each study was a 16-week, multicenter, randomized, double-blind, placebo-controlled, parallel-group study to compare the efficacy, safety, and tolerability of fremanezumab and placebo in adults with EM or CM. The studies evaluated fremanezumab at the proposed subcutaneous doses of 225 mg monthly (CM patients received a starting dose of 675 mg), 675 mg quarterly, and at two higher doses (675 mg monthly and 900 mg monthly) for 3 months.

**RESULTS:** Most patients who received fremanezumab (N=1702) or placebo (N=861) were female (87%), with mean age of 41.4 years (range = 18 to 70 years), respectively. Serious adverse events (AEs) and AEs leading to discontinuation occurred infrequently, with similar incidences in patients who received fremanezumab (1% and 2%, respectively) versus patients who received placebo (2% for both subsets). The most common AEs in the placebo-controlled studies were injection-site reactions, including induration and erythema, which tended to be transient, mild and slightly more frequent in patients who received fremanezumab versus those given placebo. Upper respiratory tract infection and nasopharyngitis, both reported with similar incidence in patients who received either fremanezumab or placebo, were the next most frequently reported AEs. Cardiovascular AEs occurred infrequently and with a similar incidence in both fremanezumab and placebo groups. No signal for hepatoxicity was observed. No anaphylaxis or severe hypersensitivity occurred, and only three patients (two on placebo, and one on fremanezumab) had AEs of drug hypersensitivity of mild or moderate severity. None of these events was serious, and all resolved with steroid and/or antihistamine treatment. Incidence of antidrug antibody (ADA) formation was low, and there were no AEs related to ADA or neutralizing antibody development.

**CONCLUSION:** Four placebo-controlled studies demonstrate that fremanezumab, at the proposed monthly and quarterly dose regimens, is an efficacious and generally safe and well-tolerated preventive therapy.

**TRIAL REGISTRATION:** ClinicalTrials.gov, NCT02621931, NCT02629861, NCT02021773, NCT02025556

**ETHICS APPROVAL**

The study was approved by all relevant independent ethics committees or institutional review boards, according to national or local regulations.

ᅟ

**P7**

**Reversion of patients with chronic migraine to an episodic migraine classification with fremanezumab treatment**

ᅟ

Joshua M. Cohen^1*^; Kristen Bibeau^1^; Maja Galic^2^; Michael J. Seminerio^1^; Verena Ramirez Campos^3^; Rashmi B. Halker Singh^4^; Jessica Ailani^5^

1 Teva Pharmaceuticals, Frazer, Pennsylvania, USA

2 Teva Pharmaceuticals, Amsterdam, The Netherlands

3 Teva Pharmaceuticals, Buenos Aires, Argentina

4 Mayo Clinic, Phoenix, Arizona, USA

5 Medstar Georgetown University Hospital, Washington, District of Columbia, USA

* Correspondence: Joshua M. Cohen (Joshua.Cohen05@tevapharm.com)

ᅟ

**The correspondence e-mail address contained an error. The correct e-mail address has now been included in this correction.**

ᅟ

**OBJECTIVE:** To evaluate the effect of fremanezumab on reversion from chronic migraine (CM) to episodic migraine (EM).

**BACKGROUND:** CM and EM are clinically, functionally, and anatomically differentiated, with evidence suggesting that they may be separate conditions. Furthermore, patients with CM usually have more comorbid conditions and more-frequent medication overuse, which complicates their clinical management. Fremanezumab, a fully humanized monoclonal antibody (IgG2a) that selectively targets calcitonin gene-related peptide (CGRP), has demonstrated efficacy in migraine prevention.

**DESIGN/METHODS:** In this Phase 3, multicenter, randomized, doubleblind, placebo-controlled, parallel-group study, adults with prospectively confirmed CM (≥15 headache days and ≥8 migraine days per month) were randomized 1:1:1 to subcutaneous injections of fremanezumab quarterly (675 mg at baseline; placebo at Weeks 4 and 8), fremanezumab monthly (675 mg at baseline; 225 mg at Weeks 4 and 8), or matching placebo over a 12-week treatment period. Post hoc analyses evaluated the proportion of patients who reverted from CM to EM, defined as patients who had ≥15 headache days per month at baseline (28-day pre-treatment period) and then had <15 headache days per month in all 3 months of the treatment period.

**RESULTS:** In an analysis of the 1130 CM patients randomized in this trial (quarterly, N=376; monthly, N=379; placebo, N=375), significantly more fremanezumab-treated patients reverted from having ≥15 headache days per month at baseline to <15 headache days per month in Months 1, 2, and 3 (quarterly: 121 patients [32%]; monthly: 133 patients [35%]) than those who received placebo (86 patients [23%]; both, *P*≤0.002). On average, these fremanezumab-treated patients had 18–19 headache days per month at baseline and showed reductions to 6–9 headache days during any month in the treatment period, representing up to an approximately 70% reduction in headache days.

**CONCLUSIONS:** Along with its efficacy as a migraine preventive treatment, fremanezumab demonstrated the potential benefit for reversion from CM to EM.

**TRIAL REGISTRATION:** ClinicalTrials.gov, NCT02621931, NCT02629861

**ETHICS APPROVAL**

The study was approved by all relevant independent ethics committees or institutional review boards, according to national or local regulations.

**Disclosures:**

**Joshua M. Cohen**: Employee of Teva Pharmaceuticals.

**Kristen Bibeau**: Former employee of Teva Pharmaceuticals.

**Maja Galic**: Employee of Teva Pharmaceuticals.

**Michael J. Seminerio**: Employee of Teva Pharmaceuticals.

**Verena Ramirez Campos**: Employee of Teva Pharmaceuticals.

**Rashmi B. Halker Singh**: Received honoraria from Current Neurology and Neuroscience Reports, MedLink, and Amgen.

**Jessica Ailani**: Received honoraria from Allergan (speaking/consulting), Avanir (speaking), Eli Lilly (speaking/advisory board), Teva Pharmaceuticals (advisory board), Promius (speaking), Current Pain and Headache Reports (section editor), Theranica (clinical trials).

ᅟ

**P8**

**Efficacy of fremanezumab in patients with chronic migraine with or without concomitant use of preventive medication**

ᅟ

Peter J. Goadsby^1*^, David W. Dodick^2^, Stephen D. Silberstein^3^, Paul P. Yeung^4^, Tricia Blankenbiller^4^, Xiaoping Ning^4^, Ronghua Yang^4^, Yuju Ma^4^, Ernesto Aycardi^4^, Marcelo E. Bigal^4^

1 NIHR-Wellcome Trust King’s Clinical Research Facility, King’s College, London, UK

2 Mayo Clinic, Phoenix, Arizona, USA

3 Jefferson Headache Center, Thomas Jefferson University, Philadelphia, Pennsylvania, USA

4 Teva Pharmaceuticals, Frazer, Pennsylvania, USA

* Correspondence: Peter J. Goadsby (Peter.Goadsby@ucsf.edu)

ᅟ

**The correspondence e-mail address contained an error. The correct e-mail address has now been included in this correction.**

ᅟ

**OBJECTIVE:** To investigate the efficacy of fremanezumab in chronic migraine (CM) patients with or without concomitant use of preventive medication.

**BACKGROUND:** Some patients with CM may take more than one preventive medication. Fremanezumab, a fully humanized monoclonal antibody (IgG2a) that selectively targets calcitonin gene-related peptide (CGRP), has demonstrated efficacy in migraine prevention.

**DESIGN/METHODS:** In this Phase 3, randomized, double-blind, placebo-controlled, parallel-group study, eligible patients with prospectively confirmed CM (≥15 headache days and ≥8 migraine days per month) were randomized 1:1:1 to receive subcutaneous injections of fremanezumab quarterly (675 mg at baseline; placebo at Weeks 4 and 8), fremanezumab monthly (675 mg at baseline; 225 mg at Weeks 4 and 8) or placebo at each time point over a 12-week treatment period. Changes from baseline were assessed in the monthly average number of headache days of at least moderate severity, and in migraine days in patients with or without concomitant preventive medication.

**RESULTS:** Analyses included 239 patients receiving one concomitant preventive medication (quarterly, *N*=77; monthly, N=85; placebo, N=77) and 882 patients receiving none (quarterly, N=298; monthly, N=290; placebo, N=294). During the 12-week treatment period, fremanezumab reduced from baseline the mean number of monthly headache days of at least moderate severity versus placebo in patients receiving concomitant preventive medication (quarterly: –3.8±0.61; monthly: –4.5±0.57; placebo: –2.5±0.61), reaching significance with monthly dosing (*P*=0.003). Reductions were also significant for fremanezumab quarterly and monthly in those not receiving concomitant preventive medication (quarterly: –4.6±0.33; monthly: –4.9±0.33; placebo: –2.7±0.33; both, *P*<0.0001). These reductions were observed as early as 4 weeks after initiation of fremanezumab monthly in patients receiving concomitant preventive medication (*P*=0.028); similarly early reductions occurred with fremanezumab monthly and quarterly in patients not receiving concomitant preventive medication (*P*<0.0001). There were also fewer migraine days with both fremanezumab regimens.

**CONCLUSIONS:** Fremanezumab demonstrated efficacy in patients with CM, regardless of concomitant preventive medication use.

**TRIAL REGISTRATION:** ClinicalTrials.gov, NCT02621931

**ETHICS APPROVAL**

The study was approved by all relevant independent ethics committees or institutional review boards, according to national or local regulations.

**Disclosures:**

**Peter Goadsby**: Personal fees from Teva Pharmaceuticals during the conduct of the study. He receives personal fees from Akita Biomedical, Alder Biopharmaceuticals, Avanir Pharma, Cipla Ltd, Dr. Reddy's Laboratories, ElectroCore LLC, Novartis, Pfizer Inc, Quest Diagnostics, Scion, MedicoLegal, UptoDate, and Oxford University Press. He receives grants and personal fees from Allergan, Amgen, Eli Lilly and Company, and eNeura Inc. He receives personal fees and other from Trigemina Inc. He reports work, personal fees from Journal Watch, and Massachusetts Medical Society, outside the submitted work. He has a patent on magnetic stimulation for headache licensed to eNeura.

**David W. Dodick**: Provides consultation to Acorda, Allergan, Amgen, Alder, Dr. Reddy’s, Merck, Promius, eNeura, Eli Lilly & Company, Insys, Autonomic Technologies, Teva, Xenon, Tonix, Trigemina, Boston Scientific, GBS, Colucid, Zosano, Laydenburg Thalmann, Biocentric, Biohaven, Magellan, Charleston Laboratories, Pfizer. Royalties: Oxford University Press and Cambridge University Press (Book Royalty). He receives editorial/honoraria from UpToDate. He receives honoraria/publishing or honoraria/royalties from Chameleon Communications, Medscape, WebMD, Academy for Continued Healthcare Learning, Haymarket Medical Education, Global Scientific Communications, HealthLogix, Academy for Continued Healthcare Learning, MeetingLogiX, Wiley Blackwell, Oxford University Press, Cambridge University Press. Stock/options: GBS/Nocira, Epien, and Mobile Health. He has a consulting use agreement with NAS. He has a board position at King-Devick Inc.

**Stephen D. Silberstein**: Provides consultation to Alder, Allergan, Amgen, Avanir, Curelater Inc., Depomed, Dr. Reddy’s Laboratories, Ensured Inc., ElectroCore Medical LLC, INSYS Therapeutics, Lilly USA LLC, Supernus Pharmaceuticals Inc., Teva Pharmaceuticals, Theranica, and Trigemina Inc.

**Paul P. Yeung**: Employee of Teva Pharmaceuticals.

**Tricia Blankenbiller**: Former employee of Teva Pharmaceuticals.

**Xiaoping Ning**: Employee of Teva Pharmaceuticals.

**Ronghua Yang**: Employee of Teva Pharmaceuticals.

**Yuju Ma**: Employee of Teva Pharmaceuticals.

**Ernesto Aycardi**: Former employee of Teva Pharmaceuticals.

**Marcelo E. Bigal**: Former employee of Teva Pharmaceuticals.

ᅟ

**P10**

**Efficacy of fremanezumab in patients with chronic migraine and comorbid moderate to moderately severe depression**

ᅟ

Joshua M. Cohen^1*^, Paul P. Yeung^1^, Ernesto Aycardi^1^, Marcelo E. Bigal^1^, Ronghua Yang^1^, Kristen Bibeau^1^, Maja Galic^2^, Michael J. Seminerio^1^, Richard B. Lipton^3^, Dawn C. Buse^3^

1 Teva Pharmaceuticals, Frazer, Pennsylvania, USA

2 Teva Pharmaceuticals, Amsterdam, The Netherlands

3 Albert Einstein College of Medicine and Montefiore Medical Center, Bronx, New York, USA

* Correspondence: Joshua M. Cohen (Joshua.Cohen05@tevapharm.com)

ᅟ

**The correspondence e-mail address contained an error. The correct e-mail address has now been included in this correction.**

ᅟ

**OBJECTIVE:** To evaluate the efficacy of fremanezumab on migraine symptoms and depression in patients with chronic migraine (CM) and comorbid moderate to moderately severe depression.

**BACKGROUND:** Depression is common in CM and contributes to the already substantial burden of disease. Fremanezumab, a fully humanized monoclonal antibody (IgG2a) that selectively targets calcitonin gene-related peptide (CGRP), has demonstrated efficacy in migraine prevention.

**DESIGN/METHODS:** In this Phase 3, multicenter, randomized, doubleblind, placebo-controlled, parallel-group study, eligible patients aged 18–70, with prospectively confirmed CM (≥15 headache days and ≥8 migraine days per month) were randomized 1:1:1 to receive subcutaneous injections of fremanezumab quarterly (675 mg at baseline; placebo at Weeks 4 and 8), fremanezumab monthly (675 mg at baseline; 225 mg at Weeks 4 and 8), or matching placebo over a 12-week treatment period. Post hoc analyses evaluated changes in headache and migraine frequency and depression in patients with moderate to moderately severe depression (score of 10–19 on the 9-item Patient Health Questionnaire [PHQ-9]) at baseline.

**RESULTS:** Almost 20% (219/1130) of randomized patients had moderate to moderately severe depression at baseline (quarterly, *n*=74; monthly, *n*=88; placebo, *n*=57). As in the overall study population, fremanezumab-treated patients in this subgroup had significant reductions from baseline in the mean number of monthly headache days of at least moderate severity (quarterly: –5.4±0.79; monthly: –5.6±0.75) versus those who received placebo (–2.2±0.84) during the 12-week treatment period (both, *P*<0.001), with effects observed as early as Week 4 (*P*<0.0001). Similar treatment differences were observed for change in the mean number of migraine days (*P*<0.001). Fremanezumab also reduced the mean PHQ-9 score from baseline to Week 12 (quarterly: –10.5±0.68; monthly: –9.5±0.63) versus placebo (–8.7±0.71); the quarterly group reached significance (*P*<0.05).

**CONCLUSIONS:** Fremanezumab demonstrated efficacy in preventive treatment of CM in patients with comorbid moderate to moderately severe depression, reducing migraine and headache frequency and improving depression.

**TRIAL REGISTRATION:** ClinicalTrials.gov, NCT02621931

**ETHICS APPROVAL**

The study was approved by all relevant independent ethics committees or institutional review boards, according to national or local regulations.

**Disclosures:**

**Joshua M. Cohen**: Employee of Teva Pharmaceuticals.

**Paul P. Yeung**: Employee of Teva Pharmaceuticals.

**Ernesto Aycardi**: Former employee of Teva Pharmaceuticals.

**Marcelo E. Bigal**: Former employee of Teva Pharmaceuticals.

**Ronghua Yang**: Employee of Teva Pharmaceuticals.

**Kristen Bibeau**: Former employee of Teva Pharmaceuticals.

**Maja Galic**: Employee of Teva Pharmaceuticals.

**Michael J. Seminerio**: Employee of Teva Pharmaceuticals.

**Richard B. Lipton**: Consultant to Teva Pharmaceuticals.

**Dawn C. Buse**: Consultant to Amgen, Allergan, Avanir, Biohaven, Eli Lilly and Promeius.

ᅟ

**P11**

**Achievement of response over time with fremanezumab in the treatment of chronic and episodic migraine**

ᅟ

Stephen D. Silberstein^1*^, Richard B. Lipton^2^, Merle L. Diamond^3^, Joshua M. Cohen^4^, Ronghua Yang^4^, Bo Jiang^4^

1 Jefferson Headache Center, Thomas Jefferson University, Philadelphia, Pennsylvania, USA

2 Albert Einstein College of Medicine, Bronx, New York, USA

3 Diamond Headache Clinic, Chicago, Illinois, USA

4 Teva Pharmaceuticals, Frazer, Pennsylvania, USA

* Correspondence: Stephen D. Silberstein (Stephen.Silberstein@jefferson.edu)

**The correspondence e-mail address contained an error. The correct e-mail address has now been included in this correction.**

ᅟ

**OBJECTIVES:** The long-term efficacy of monoclonal antibodies that selectively target calcitonin gene-related peptide (CGRP) in patients with early treatment failure is not well characterized. Based on data from Phase 3 trials in episodic (EM) and chronic migraine (CM) of fremanezumab, a fully humanized monoclonal antibody (IgG2a) that selectively targets CGRP, we assessed long-term treatment response rates in patients with early treatment failure.

**METHODS:** This multicenter, randomized, double-blind, parallelgroup, long-term study, included patients who completed either 12-week Phase 3 study (HALO CM or HALO EM). Patients continued on treatment from the 12-week studies, receiving either subcutaneous fremanezumab quarterly (675 mg every 3 months), fremanezumab monthly (CM: 675 mg at baseline and 225 mg every month; EM: 225 mg every month) over a 12-month treatment period. The percentage of patients with a reduction in migraine days (response rates) >40% at Months 6 and 9 among patients with low response rates (<40%) at Month 1 was assessed in patients who received active treatment in the 12-week studies.

**RESULTS:** CM patients with <20% reduction in migraine days at Month 1 had >40% response rates of 29% (58/197) at Month 6 and 43% (35/81) at Month 9. Patients with <20% reduction at Month 3 had >40% response rates of 18% (32/176) at Month 6 and 30% (21/69) at Month 9. Patients with <40% reduction at Month 1 had >40% response rates of 36% (99/272) at Month 6 and 51% (55/108) at Month 9. Patients with <40% reduction at Month 3 had >40% response rates of 28% (72/253) at Month 6 and 41% (41/101) at Month 9. EM patients with <20% reduction in migraine days at Month 1 had >40% response rates of 53% (53/100) at Month 6 and 62% (26/42) at Month 9. Patients with <20% reduction at Month 3 had >40% response rates of 41% (34/83) at Month 6 and 47% (15/32) at Month 9. Patients with <40% reduction at Month 1 had >40% response rates of 57% (92/162) at Month 6 and 63% (45/72) at Month 9. Patients with <40% reduction at Month 3 had >40% response rates of 46% (62/135) at Month 6 and 61% (33/54) at Month 9.

**CONCLUSIONS:** Failure to achieve an early response to fremanezumab does not predict failure at later time points.

**TRIAL REGISTRATION:** ClinicalTrials.gov, NCT02638103

**ETHICS APPROVAL**

The study was approved by all relevant independent ethics committees or institutional review boards, according to national or local regulations.

**P12**

**The impact of fremanezumab on medication overuse in patients with chronic migraine**

ᅟ

Stephen D. Silberstein^1*^, Sait Ashina^2^, Zaza Katsarava^3^, Kristen Bibeau^4^, Michael J. Seminerio^4^, Danielle E. Harlow^4^, Joshua M. Cohen^4^

1 Jefferson Headache Center, Thomas Jefferson University, Philadelphia, Pennsylvania, USA

2 Beth Israel Deaconess Medical Center Comprehensive Headache Center, Harvard Medical School, Boston, Massachusetts, USA

3 University of Essen, Unna, Germany

4 Teva Pharmaceuticals, Frazer, Pennsylvania, USA

* Correspondence: Stephen D. Silberstein (Stephen.Silberstein@jefferson.edu)

ᅟ

**The correspondence e-mail address contained an error. The correct e-mail address has now been included in this correction.**

ᅟ

**OBJECTIVES:** Overuse of acute or symptomatic headache medications (triptans, ergot derivatives, opioids, and combination analgesics) can cause medication overuse headache (MOH), which often accompanies chronic migraine (CM). Fremanezumab, a fully humanized monoclonal antibody (IgG2a) that selectively targets calcitonin gene-related peptide (CGRP), reduced the frequency and severity of headaches in CM patients. We assessed the effect of fremanezumab on medication overuse and acute headache medication use in CM patients.

**METHODS:** In this multicenter, randomized, double-blind, placebocontrolled, Phase 3 study, CM patients CM were randomized 1:1:1 to receive subcutaneous fremanezumab quarterly (675 mg at baseline, and placebo at Weeks 4 and 8), fremanezumab monthly (675 mg at baseline, and 225 mg at Weeks 4 and 8), or placebo over a 12-week treatment period. We assessed the proportion of patients who reverted from overusing medications at baseline (use of acute headache medication on ≥15 days, use of migraine-specific acute medication on ≥10 days, or use of combination medications for headache on ≥10 days during the 28-day baseline period) to not overusing medications at Week 12, and the change from baseline in the number of days of acute headache medication use among these patients. Analyses were performed in the full analysis set (all randomized patients who received ≥1 dose of study drug and had ≥10 days of post-baseline efficacy assessments on the primary endpoint).

**RESULTS:** Among patients with medication overuse at baseline (quarterly n=201; monthly n=198; placebo n=188), more fremanezumabtreated patients reported no medication overuse during the 12-week treatment period (quarterly: 111/201 patients [55%], *P*=0.0389; monthly: 120/198 patients [61%], *P*=0.0024) than those who received placebo (87/188 patients [46%]). This response was seen as early as Week 4 (quarterly: 102/201 patients [51%], *P*=0.0091; monthly: 107/198 patients [54%], *P*=0.0014; vs placebo: 73/188 patients [39%]). Among patients who responded (quarterly *n*=111; monthly *n*=120; placebo *n*=87), the baseline number of days with medication overuse was similar across treatment groups (quarterly [mean ± standard error]: 16.6±0.32 days; monthly: 16.7±0.33 days; placebo: 16.6±0.35). Within this population, fremanezumab treatment reduced the days of acute headache medication use over the treatment period (quarterly: –9.0±0.41 days, *P*=0.0017; monthly: –8.9±0.41 days, *P*=0.0040) versus those who received placebo (–7.1±0.46 days).

**CONCLUSIONS:** Fremanezumab treatment was associated with reduced overuse of acute medications and fewer days using acute medications.

**TRIAL REGISTRATION:** ClinicalTrials.gov, NCT02621931

**ETHICS APPROVAL**

The study was approved by all relevant independent ethics committees or institutional review boards, according to national or local regulations.

ᅟ

**P136**

**Epidemiology of chronic and episodic migraine in Europe**

ᅟ

Hicham Benhaddi^1*^, Timothy Fitzgerald^2^, Sophie McCabe^3^, Ruth Zeidman^3^

1 Teva Pharmaceuticals, Wilrijk, Belgium

2 Teva Pharmaceuticals, Frazer, Pennsylvania, USA

3 Covance Market Access, London, UK

* Correspondence: Hicham Benhaddi (Hicham.Benhaddi@tevaeu.com)

ᅟ

**The correspondence e-mail address contained an error. The correct e-mail address has now been included in this correction.**

ᅟ

**BACKGROUND:** Migraine is a debilitating neurological disorder characterized by headaches of varying duration and intensity. Treatment options vary depending on disease severity, frequency, regional practices, and therapy availability.

**OBJECTIVE:** To conduct a systematic literature review of the epidemiology (incidence, prevalence, mortality, and morbidity), current treatment pathways and patterns, preventive therapy guidelines, and unmet needs of chronic (CM) and episodic migraine (EM) in Europeans.

**METHODS:** Literature searches and evidence screening were structured according to the PICOS (population, intervention, comparators, outcomes, and study types) framework. Reviews, original studies, and clinical guidelines in European adults (≥18 years) with EM (<15 headache days per month), CM (≥15 headache days with ≥8 migraine days per month), or medication overuse headache (MOH) were included. Searches focused on epidemiology, incidence, prevalence, mortality, morbidity, treatment patterns, clinical guidelines relating to preventive interventions, and unmet need (published 2007–February 1, 2018; geographical limitation: United Kingdom, France, Germany, Spain, Italy, the Netherlands, Poland, Denmark, Finland, Iceland, Norway and Sweden). Searches included: Embase, MEDLINE, and the Cochrane Library databases; specialty medicine associations; and health technology assessment agency websites.

**RESULTS:** Analysis included 64 publications. The World Health Organization estimated 77 million migraine sufferers in Europe, with an incidence of up to 39.2 per 1000 patient-years. Migraine prevalence was higher (2–6-fold) in women than in men in all but one study, with peak prevalence at 25–55 years of age. EM was up to 15 times more prevalent than CM. MOH has an estimated prevalence of 1% in the general adult population. Preventive therapies are typically betablockers, antidepressants, anticonvulsants (topiramate), or in some countries, onabotulinumtoxinA (for chronic migraine), but most have no proven efficacy in CM. Guidelines vary by country, but the European Headache Federation (EHF) recommends preventive therapy for patients with ≥2 debilitating attacks per month; however, ≤13% of patients who qualify for preventive treatment actually receive it. Adherence to available preventive therapies is low, and many physicians believe that the disadvantages, including adverse events, drug dependency, and lack of sustained efficacy outweigh the benefits. To manage MOH, withdrawal of treatment is recommended.

**CONCLUSIONS:** Migraine is highly prevalent in Europe. Despite improvements in compliance with treatment guidelines, the number of patients on preventive therapies remains low, likely due to poor tolerability and efficacy of available therapies and limited access to these medications due to restrictive guidelines.

ᅟ

**P137**

**The impact of offering monthly and quarterly dosing options for a new class of migraine preventive therapy on likelihood of acceptance and adherence in adults with migraine**

ᅟ

Robert Cowan^1^, Joshua Cohen^2^, Erik Rosenman^3^, Tim Fitzgerald^2^, Ravi Iyer^2*^

1 Neurology, Stanford University School of Medicine, Stanford, California, 94305, USA

2 Teva Pharmaceutical Industries, Frazer, Pennsylvania, 19355, USA

3 IQVIA, Inc., Cambridge, Massachusetts, 02139, USA

* Correspondence: Ravi Iyer (Ravi.Iyer01@tevapharm.com)

ᅟ

**The correspondence e-mail address contained an error. The correct e-mail address has now been included in this correction.**

ᅟ

**Background:** Migraine affects approximately 39 million people in the US1. A new class of migraine preventive therapy launching in 2018-2019 will provide physicians and patients with an alternate approach to preventive treatment. This study sought to understand the impact, if any, of having both monthly and quarterly dosing options on acceptance of, and adherence to, the new class of migraine preventive therapy among adults with migraine.

**Methods:** In this double-blind, observational study, 420 US adults with migraine completed a 20-minute, self-administered online survey. Respondents included 228 moderate-frequency episodic (5-9 headache days / month), 106 high-frequency episodic (10-14 headache days / month), and 86 chronic migraine patients (≥15 headache days / month). Adults with migraine were exposed to three scenarios: 1) only monthly dosing of the new class of migraine preventive therapy is available, 2) only quarterly dosing is available, and 3) both monthly and quarterly dosing are available. In each scenario, and assuming roughly equivalent efficacy regardless of dosing schedule, adults with migraine were asked their likelihood to fill the prescription (if prescribed) and their likelihood to take it consistently over one year, measured on a 7-point scale where 1 was “not at all likely” and 7 was “extremely likely”. Those that selected a 6 or 7 on the scale were classified as “likely”. At the end of the survey, respondents were then asked if they preferred either monthly or quarterly dosing for this new class of therapy. Data analysis included descriptive statistical analyses and comparison of means through ANOVA testing, with significance set at *p*<0.05.

**Results:** A similar proportion of adults with migraine preferred monthly (35.7%) and quarterly (39.5%) dosing regimens (24.8% had no preference). Among those who prefer monthly dosing (*n*=150), a greater proportion indicate they are likely to fill the prescription and remain adherent when only monthly is prescribed and available compared to when only quarterly is (77% vs. 56% *p*<0.001 and 80% vs. 57% *p*<0.001 respectively). Likewise, among those who prefer quarterly dosing (*n*=166), a greater proportion indicate they are likely to fill and remain adherent when only quarterly is prescribed and available compared to when only monthly is (63% vs. 55% *p*<0.008 and 62% vs. 54% *p*<0.023 respectively).

**Conclusions**

Adults with migraine are more likely to fill the new class of preventive therapy and to take it consistently over one year when presented with their preferred dosing regimen.

**References**

1. http://migraineresearchfoundation.org/about-migraine/migraine-facts/ Last accessed June 22nd, 2018

## References

[1] Benhaddi H (2018). 12th European Headache Federation Congress jointly with 32nd National Congress of the Italian Society for the Study of Headaches. J Headache Pain.

